# Diagnostic Performance of a Locally Deployed Vision Language Model for Bone Tumor Diagnosis Using Smartphone-Captured Images: Exploratory Retrospective Study

**DOI:** 10.2196/99757

**Published:** 2026-09-21

**Authors:** Yin Shi, Zhi-Chao Tong

**Affiliations:** 1 Department of Orthopedic Oncology Honghui Hospital, Xi'an Jiaotong University Xi’an, Shaanxi Province China

**Keywords:** vision language models, bone tumors, retrieval-augmented generation, RAG, artificial intelligence hallucination, clinical decision support, prompt engineering

## Abstract

**Background:**

Vision language models (VLMs) show promise in medical imaging, yet their performance on high-noise smartphone-captured images—common in primary care referrals—remains untested. Furthermore, it remains controversial whether retrieval-augmented generation (RAG) using external expert guidelines actually improves diagnostic accuracy for rare bone tumors.

**Objective:**

This study aims to evaluate the diagnostic efficacy of a locally deployed, open-source VLM (Qwen3-VL) on high-noise bone tumor images. Specifically, we investigated how clinical persona prompts and RAG integration were associated with diagnostic performance and observed error patterns during multimodal reasoning.

**Methods:**

This retrospective study included 42 patients with biopsy-proven primary bone tumors and tumor-like lesions. To simulate real-world conditions, we captured the original DICOM (Digital Imaging and Communications in Medicine) images from a monitor using a handheld smartphone without stabilization, organically capturing ambient glare and Moiré patterns typical of real-world teleconsultations. Using a 2×2 factorial design, we compared the diagnostic performance of the base model versus the RAG-integrated model under 2 distinct system personas: “radiologist” and “orthopedic oncologist.” Primary outcomes were top-1 and top-3 diagnostic accuracy. We used the McNemar test for paired comparisons of diagnostic correctness before and after RAG integration, and conducted an exploratory analysis of AI hallucinations using model-generated reasoning traces.

**Results:**

Without RAG, the top-3 accuracy showed no significant difference between the radiologist and orthopedic oncologist personas (15/42, 36% vs 14/42, 33%; *P*=.76). After RAG integration, top-1 accuracy in the radiologist persona decreased from 12 (29%, 95% CI 16%-45%) to 6 (14%, 95% CI 5%-29%; *P*=.03). In the orthopedic oncologist persona, no significant change was observed in top-1 or top-3 accuracy (*P*=.65 and *P*>.99, respectively). Exploratory review of model-generated reasoning traces identified several text-associated diagnostic error patterns, including demographic anchoring, trauma-related masking, and shifts toward rare diagnoses following RAG retrieval.

**Conclusions:**

In this exploratory study, adding the evaluated RAG configuration did not improve diagnostic accuracy and was associated with text-related diagnostic errors under smartphone-captured degraded imaging conditions. The findings suggest that persona design may influence the robustness of VLM responses to retrieved information, but this observation requires validation in larger, multimodel studies. Future studies should evaluate whether targeted visual fine-tuning on representative real-world degraded medical images can improve robustness under such conditions.

## Introduction

### The Bigger Picture

Artificial intelligence has achieved remarkable milestones in medical imaging, yet a critical “last-mile” bottleneck remains: the gap between pristine laboratory data and the messy reality of clinical practice. In resource-limited settings, such as primary care facilities and remote clinics in Western China, expert teleconsultations rarely rely on high-resolution DICOM (Digital Imaging and Communications in Medicine) transfers. Instead, they heavily depend on smartphone photos of computer screens, organically plagued by ambient glare and Moiré patterns. Furthermore, deploying advanced AI typically requires specialized computer science expertise, creating an adoption barrier for frontline doctors. This study breaks from traditional benchmarks by having a practicing orthopedic oncologist—without specialized AI programming training—deploy and stress-test an open-source vision language model (VLM) locally. By exposing the model to authentic, high-noise clinical images, we reveal critical vulnerabilities, such as retrieval-augmented generation (RAG)–induced “text-induced visual misinterpretations,” providing an essential preliminary warning for safely integrating AI into grassroots health care.

### Background

VLMs have demonstrated substantial potential in the radiological analysis of common orthopedic diseases. However, primary bone tumors and tumor-like lesions remain diagnostically challenging due to their low incidence, diverse histological subtypes, and complex, often nonspecific, imaging manifestations [1]. Although histopathological biopsy remains the gold standard for definitive diagnosis, the formulation of initial clinical management plans relies heavily on the comprehensive assessment of patient history and multimodal imaging (radiographs, computed tomography [CT], and magnetic resonance imaging [MRI]) [2].

In current hierarchical health care systems and teleconsultation settings, regional orthopedic oncology experts frequently provide preliminary diagnostic advice via online platforms or instant messaging applications (eg, WeChat) based on sparse medical histories and imaging data provided by primary care physicians or patients [3]. Due to cross-institutional data silos, hardware limitations, and strict privacy regulations, sharing original high-resolution DICOM images is often impractical. Consequently, consultation materials typically consist of “screen-captured” photos taken with smartphones by junior physicians who lack specialized musculoskeletal oncology experience. These physicians subjectively select what they perceive to be the most representative image slices. Such smartphone-captured images are inevitably degraded by inconsistent device resolutions, environmental glare, and Moiré patterns [4]. The poor image quality, combined with the absence of preliminary reports from specialized musculoskeletal radiologists, significantly complicates the expert consultation process.

Recent advancements in large language models (LLMs) and domain-specific VLMs have demonstrated transformative potential across broad medical domains, including radiology and clinical reasoning [5,6]. To further bridge knowledge gaps in specialized fields, RAG has increasingly been adopted to connect LLMs with authoritative clinical guidelines, thereby reducing general open-domain hallucinations [7]. Integrating domain-specific VLMs to assist in interpreting these suboptimal images could substantially empower primary care and junior physicians, thereby reducing the risks of missed diagnoses and misdiagnoses of rare bone tumors [8]. Nevertheless, directly processing sensitive patient histories and medical images through commercial cloud-based LLMs poses severe data breach risks and ethical concerns [9]. Therefore, the localized, private deployment of high-performance VLMs with robust image parsing and logical reasoning capabilities (such as Qwen3-VL) in a completely offline environment presents an ideal solution to balance patient data security with efficient clinical decision support [10].

### Objectives

Therefore, this study aims to evaluate the diagnostic efficacy of an open-source, reasoning-enabled vision language model Qwen3-VL when processing high-noise, smartphone-captured bone tumor images from real-world clinical scenarios. Furthermore, we investigate how two distinct prompt engineering strategies influence the model’s diagnostic behavior.

Specific objectives include:

1. Persona Constraint Effect: To evaluate the differences in extracting differential diagnostic features and the resilience against confounding medical histories when the model operates under a specialized “orthopedic oncologist” persona versus a conventional “radiologist” persona.
2. The RAG Trade-Off (Impact of RAG): To investigate whether RAG—using authoritative international imaging texts and clinical guidelines—effectively bridges the model’s knowledge gaps regarding rare diseases during multimodal visual reasoning tasks. Alternatively, we examine whether cross-lingual and cross-dimensional information retrieval inadvertently provokes a “cognitive conflict” between text priors and actual visual features.

Ultimately, this study seeks to establish evidence-based best practices for the secure, localized clinical deployment of multimodal LLMs in diagnosing bone tumors and other rare diseases.

## Methods

### Sample Set Construction

We used a retrospective study design, consecutively enrolling 42 patients admitted to the Orthopedic Oncology Department at Xi'an Honghui Hospital between October 2023 and March 2026. Inclusion criteria were as follows: (1) diverse age representation; (2) availability of preoperative x-ray, CT, or MRI scans accessible via Xi'an Honghui Hospital picture archiving and communication system (PACS) monitors; and (3) a definitive histopathological diagnosis confirmed by surgical biopsy [11]. Notably, obtaining a complete trimodality imaging suite (x-ray, CT, and MRI) for every single patient is exceptionally rare in routine orthopedic practice, especially for benign lesions. This rigorous inclusion criterion ensured that the model’s visual reasoning was evaluated against a high-dimensional, comprehensive clinical ground truth, compensating for the relatively small cohort size. To prevent the model from developing an overwhelming demographic anchoring bias, we intentionally excluded bone metastasis cases, which have a high incidence but lack specific morphological features of primary tumors. Consequently, the cohort focused exclusively on primary bone tumors and tumor-like lesions. The original input images for all 42 cases are provided in Multimedia Appendices 1-5.

The pathological distribution of the 42 included cases was as follows: epidermoid cyst (n=1), aneurysmal bone cyst (n=2), chondroblastoma (n=1), non-ossifying fibroma (n=1), giant cell tumor of bone (GCTB; n=5), simple bone cyst (n=1), intraosseous ganglion cyst (n=1), intraosseous hemangioma (n=2), osteosarcoma (n=13), osteochondroma (n=3), fibrous dysplasia (n=4), osteoid osteoma (n=1), liposclerosing myxofibrous tumor (n=1), enostosis/bone island (n=1), enchondroma (n=2), and undifferentiated pleomorphic sarcoma (n=3).

### Hardware Setup and Image Acquisition

To rigorously simulate the high-noise image conditions typical of primary care referrals and teleconsultations, we standardized the hardware variables.

#### Model Inference Terminal

To reflect portable teleconsultation needs, we used a consumer-grade, high-performance laptop (ASUS Tianxuan Air, AMD Ryzen AI Max+ 392, 64GB RAM, 1TB SSD) rather than a fixed high-end computing workstation. We allocated a fixed 32GB of RAM for model loading.

#### Capture Device and Conditions

A single orthopedic oncology specialist manually photographed all images using a standard smartphone (VIVO iQOO 12, 16GB+1TB, 50-megapixel primary camera). We strictly avoided stabilizing equipment like tripods to replicate a typical, handheld clinical capture environment [4,12].

#### Display and Noise Control

Ambient lighting was fixed to standard clinic overhead fluorescent lights. The display terminal was a Lenovo ThinkVision T2014 LCD monitor (1600×900 resolution) routinely used in our department. We maintained a shooting distance of 30-40 cm. *Specific setting*: Rather than artificially injecting digital noise, our goal was to organically capture the degradation inherent in real-world teleconsultation workflows. The images were captured directly from standard clinical liquid crystal display (LCD) monitors without any attempt to optimize lighting, clean screens, or eliminate reflections. This authentically preserved environmental glare, display artifacts, and Moiré patterns, accurately reflecting the suboptimal and highly noisy visual materials typically forwarded by primary care physicians.

#### Image Selection Principle

To prevent context window overflow from excessively long sequence stacks, the specialist subjectively selected 3 to 5 slices exhibiting the most specific lesional characteristics from each patient’s x-ray, CT, and MRI series. All patient privacy information was redacted prior to capture.

### Model Deployment and Software Environment

#### Test Model

We selected Qwen3-VL (specifically, the 32B-Thinking 4-bit quantized checkpoint: Qwen3VL-32B-Thinking-Q4_K_M), an open-source VLM equipped with native chain-of-thought reasoning capabilities [10].

#### Operating Framework

The back-end inference service was hosted locally via LM Studio (version 0.4.6; Element Labs). Front-end prompt interactions and RAG knowledge base management were facilitated by AnythingLLM (v1.11.0-2; Mintplex Labs).

#### Operator Profile

To objectively simulate grassroots adoption, the entire hardware deployment, prompt engineering, and RAG configuration were independently executed by a frontline orthopedic oncology clinician. No computer scientists or AI engineers were involved in optimizing the back-end code. This setup strictly evaluated the “out-of-the-box” clinical utility of locally deployed VLMs.

#### Parameter Control

To objectively assess the model’s out-of-the-box performance in real-world diagnostic support scenarios and avoid human-induced bias from excessive hyperparameter tuning, we locked the inference temperature at the system default of 0.7 across all testing batches. This setting preserved a reasonable degree of generative diversity required for internal consistency testing.

### RAG Knowledge Base Construction and Preprocessing

To ensure the external knowledge retrieved during the RAG phase aligned with the highest global academic consensus in orthopedics, we constructed a highly specialized, English-only gold-standard knowledge base. This repository comprised the following two components, which were uniformly mounted to both the RAG-radiologist and RAG-ortho oncologist test groups.

#### Gold-Standard Imaging Monograph: Diagnostic Imaging: Musculoskeletal Non-Traumatic Disease (2022 Edition, Elsevier) [13]

To prevent context confusion during the model’s retrieval process, we performed professional manual “noise reduction” preprocessing prior to database construction. Specifically, we cropped and discarded the first 139 pages covering various degenerative osteoarthropathies, retaining all subsequent chapters (particularly Section 8, covering adult and pediatric infections/acute osteomyelitis, which frequently mimic bone tumors). This step aimed to improve the retriever’s recall precision for differential features of tumors and tumor-like lesions.

#### Global Clinical Practice Consensus Guidelines: The 2026 NCCN Clinical Practice Guidelines in Oncology: Bone Cancer [14]

This guideline provided the model with authoritative epidemiological priors and clinical diagnostic weighting.

To ensure the full reproducibility of the RAG pipeline, the local AnythingLLM framework was configured with precise hyperparameters. Document vectorization was performed using the built-in all-MiniLM-L6-v2 embedding model. The English reference texts were segmented using a maximum chunk size of 1000 tokens with a chunk overlap of 20 tokens. During the retrieval phase, the system was configured to inject a maximum of 4 context snippets (top-*k*=4) into the prompt per query, applying a minimum similarity score threshold of 0.25. No secondary reranking mechanism was used.

Regarding cross-lingual retrieval (Chinese clinical prompts querying English knowledge bases), no explicit external translation API was used. Instead, we relied on the native multilingual semantic alignment capabilities of the embedding model and the underlying Qwen3-VL reasoning engine. All retrieved context passages and the model’s corresponding reasoning steps were fully logged via the back-end interface for subsequent qualitative analysis.

### Model Intervention Grouping and 2×2 Factorial Design

We designed a 2×2 factorial evaluation test focusing on the combination of “minimal history text + captured images” for the same cohort of 42 cases. The experiment included 2 independent intervention dimensions: “clinical persona” and “RAG,” resulting in 4 groups:

- Group A1: Base-Radiologist: Used the pure base model without an external knowledge base. The system prompt established the persona as a “chief physician of radiology at a provincial tertiary hospital.” This group assessed the model’s baseline interpretative ability based purely on visual morphological features.
- Group A2: RAG-Radiologist: Built upon the A1 persona by activating the RAG function via AnythingLLM, mounting the aforementioned authoritative texts as a local knowledge base [15].
- Group B1: Base-Ortho Oncologist: Used the pure base model without an external knowledge base. The system prompt established the persona as an “orthopedic oncology consultation expert at a provincial tertiary hospital.” This evaluated whether a specialized clinical persona prompts the model to assign higher clinical reasoning weight to textual features such as patient age, disease duration, and trauma history.
- Group B2: RAG-Ortho Oncologist: Built upon the B1 persona by activating RAG and mounting the same local knowledge base.

### Prompt Engineering and Task Instruction Control

To ensure structured, clinically applicable outputs while mitigating human-induced bias, we designed a standardized, minimalist zero-shot prompt framework (Table 1) [16]. This framework consisted of three core modules:

1. System Persona: Strictly defined the model as either an “orthopedic radiology” or “orthopedic oncology” consultation expert, instructing it to be proficient in differentiating various bone diseases.
2. Input Variables: Standardized the ingestion of patient demographic characteristics (gender, age), a minimal medical history, and the anatomical lesion location.
3. Output Structure Constraints: Required the model to sequentially provide an objective “imaging description,” a “top-3 differential diagnosis” ranked by probability, and “clinical recommendations.”

Note, the underlying chain-of-thought reasoning logs were automatically generated by the Qwen3-VL model’s native thinking mechanism and fully retained via the back-end interface for subsequent qualitative analysis. No mandatory constraints were forced onto the reasoning process within the prompts, maximizing the natural and objective nature of the testing task.

**Table 1:** Prompt engineering and task instruction framework.

Module	Original Chinese input	English translation
System prompt	身份设定：你是一名省级三甲医院[骨科影像学/骨肿瘤外科]会诊专家（精通各类骨肿瘤与骨病影像鉴别）。请基于提供的X线、CT及MRI影像和极简病史，给出影像学研判。	*Persona Setting*: You are a consultation expert in [Orthopedic Radiology / Orthopedic Oncology] at a provincial tertiary hospital, proficient in the imaging differential diagnosis of various bone tumors and bone diseases. Please provide an imaging assessment based on the provided x-ray, CT^a^, and MRI^b^ images, along with a brief medical history.
Patient data	病变位置：[填入部位] 患者信息：[性别] [年龄] [简要病史及外伤史]	*Lesion Location*: [Insert location] *Patient Info*: [Gender], [Age], [Brief medical history]
Output requirements	要求： 1. **影像学描述**：客观描述你观察到的核心影像学特征。 2. **鉴别诊断**：结合病史与影像，按可能性从大到小，列出最可能的 3 个具体疾病诊断。 3. **临床建议**：给出下一步的具体诊疗或处置建议。	*Requirements*: Imaging Description: Objectively describe the core imaging features you observe. Differential Diagnosis: Based on the history and images, list the top 3 most likely specific disease diagnoses in descending order of probability. Clinical Recommendations: Provide specific recommendations for the next step in diagnosis or management.

^a^CT: computed tomography.

^b^MRI: magnetic resonance imaging.

### Statistical Analysis

All statistical analyses in this study were performed using Jamovi software (version 2.6.44). Categorical variables, such as the top-1 and top-3 diagnostic accuracies of the models, were presented as frequencies and percentages. Given the paired design of this study (ie, repeated evaluations of the same 42 cases across different clinical personas and RAG intervention strategies), the McNemar test was used to compare the differences in diagnostic accuracy. These comparisons included evaluations between different clinical personas (eg, base-radiologist vs base-ortho oncologist) as well as within the same persona before and after RAG integration. All statistical tests were 2-sided, and a *P* value <.05 was considered statistically significant.

Diagnostic correctness was evaluated by a senior orthopedic oncologist. A “top-1” match was defined as the model’s first predicted diagnosis conceptually matching the biopsy-proven histological ground truth (including clinically accepted synonyms). A “top-3” match was defined as the ground truth appearing anywhere within the model’s top 3 ranked predictions. Diagnostic synonyms and hierarchical diagnostic relationships (eg, secondary aneurysmal bone cyst vs primary) were adjudicated clinically by the senior orthopedic oncologist according to accepted diagnostic terminology.

### Reporting Guideline

This study was reported in accordance with the STARD-AI (Standards for Reporting of Diagnostic Accuracy Studies–Artificial Intelligence) guideline.

### Ethical Considerations

This retrospective study was conducted in accordance with the principles of the Declaration of Helsinki and received formal approval from the Clinical Research Ethics Committee of Xi’an Honghui Hospital (approval no. 2026-KY-067-01). Written informed consent was obtained from all participating patients or their legal guardians. All patient identifiers were strictly redacted prior to analysis to protect participant privacy. No financial compensation was provided to participants as this was a retrospective study.

## Results

### Baseline Characteristics

The final cohort comprised 42 patients with biopsy-proven primary bone tumors and tumor-like lesions. Table 2 summarizes the baseline characteristics of the study population. The mean age was 36.9 (SD 20.3) years, with an approximately balanced sex distribution. Lesions were predominantly located in the long bones of the lower extremities (femur, tibia, and fibula), accounting for 32 (76%) of the cases. Regarding tumor biological behavior, benign or tumor-like lesions constituted 26 (62%) of the cohort, while malignant tumors (primarily osteosarcoma) comprised the remaining 16 (38%). Furthermore, 5 (12%) of the patients reported a definitive history of recent trauma. Detailed case-by-case data and supplementary information are provided in Multimedia Appendix 6.

**Table 2:** Baseline characteristics of the study population (n=42).

Characteristics			Values
Age (years), mean (SD)			36.9 (20.3)
Gender, n (%)			
	Male	20 (48)	
	Female	22 (52)	
Anatomical location, n (%)			
	Lower extremity (femur/tibia/fibula)	32 (76)	
	Upper extremity (humerus/radius/ulna)	6 (14)	
	Hands and feet	3 (7)	
	Axial skeleton (pelvis)	1 (2)	
History of trauma, n (%)			
	Yes	5 (12)	
	No	37 (88)	
Tumor behavior, n (%)			
	Benign or tumor-like lesions	26 (62)	
	Malignant	16 (38)	

### Diagnostic Performance Across Personas and RAG Integration

Table 3 summarizes the diagnostic accuracies and McNemar test results across all intervention groups. For the base models operating without RAG, the top-3 diagnostic accuracy showed no significant difference between the radiologist persona (group A1) and the orthopedic oncologist persona (group B1) (36% vs 33%, *P*=.76). However, integrating the RAG knowledge base precipitated different performance shifts between the two personas. After RAG integration, top-1 accuracy in the radiologist persona decreased from 29% to 14% (*P*=.03). Specifically, Table 4 details the exact McNemar contingency matrix, revealing 7 discordant pairs where the base model was correct but the RAG model was incorrect, compared to only 1 pair where the RAG model successfully corrected the base model’s error. Conversely, in the orthopedic oncologist persona, no significant change was observed in top-1 or top-3 accuracy following RAG integration (*P*=.65 and *P*>.99, respectively).

**Table 3:** Impact of retrieval-augmented generation (RAG) integration on diagnostic accuracy across different clinical personas (n=42).

Persona and metrics				Base model, n (%, 95% CI)		RAG model, n (%, 95% CI)		*P* value (McNemar test)
Radiologist								
	Top-1 accuracy		12 (29%, 95% CI 16%-45%)		6 (14%, 95% CI 5%-29%)		.03^a^	
	Top-3 accuracy		15 (36%, 95% CI 22%-52%)		10 (24%, 95% CI 12%-39%)		.13	
Ortho oncologist								
		Top-1 accuracy	8 (19%, 95% CI 9%-34%)		7 (17%, 95% CI 7%-31%)		.65	
		Top-3 accuracy	14 (33%, 95% CI 20%-50%)		14 (33%, 95% CI 20%-50%)		>.99	

^a^Statistically significant (*P*<.05). *P* values indicate the difference in diagnostic accuracy before and after RAG integration within the same persona, calculated using the standard McNemar test. The baseline top-3 accuracy between the base-radiologist and base-ortho oncologist showed no significant difference (*P*=.76).

**Table 4:** McNemar contingency table for the radiologist persona before and after RAGa integration (n=42).

	RAG correct	RAG incorrect
Base correct	5	7
Base incorrect	1	29

^a^RAG: retrieval-augmented generation.

### Exploratory Analysis of Model-Generated Diagnostic Error Patterns

To investigate the underlying logical flaws exhibited by the VLMs during multimodal diagnostics, we qualitatively analyzed the model-generated reasoning traces of typical severe misdiagnoses (Table 5). Exploratory review identified three recurring error patterns: (1) demographic overanchoring bias induced by patient age and epidemiological probabilities (eg, case 11), (2) premature visual closure masked by confounding textual inputs such as a history of trauma (eg, case 27), and (3) RAG-associated shifts toward malignant differentials (eg, case 3). The full LLM-generated outputs and reasoning traces for all 42 cases are detailed in Multimedia Appendices 7 and 8.

**Table 5:** Exploratory analysis of model-generated diagnostic error patterns.

Case info	Ground truth	AI diagnosis	Observed diagnostic error pattern
*Case 11*: 47 years old, female, right humerus, no trauma	Simple bone cyst with hemorrhage (benign)	All Groups: bone metastasis, osteosarcoma	*Demographic Anchoring Bias*: The AI overrelied on the statistical prior that “middle-aged patients with bone lesions have a high probability of metastasis” and did not appropriately account for the relatively benign morphological margins visible in the images.
*Case 27*: 15 years old, male, right femur, trauma (1 day)	Osteosarcoma (malignant)	Base/RAG^a^-Radiologist: Salter-Harris II fracture, epiphyseal separation Base/RAG-Ortho Oncologist: correctly identified osteosarcoma with pathological fracture	*Premature Closure via Trauma Masking*: The radiologist persona prematurely anchored its diagnosis on the mechanical trauma history, misinterpreting the malignant lesion as a benign sports injury. Conversely, the orthopedic persona successfully resisted this text bias, demonstrating the clinical resilience of specialized prompts.
*Case 3*: 6 years old, female, left tibia, no trauma	Aneurysmal bone cyst	Base Model: bone cyst, non-ossifying fibroma (benign cluster) RAG Model: Ewing sarcoma, acute osteomyelitis	*RAG-Associated Diagnostic Shift*: While the base model correctly confined its differential to benign entities based on visual features, the RAG integration retrieved aggressive pediatric textbook priors (Ewing sarcoma), overriding visual intuition and coinciding with a marked shift toward malignant diagnoses.

^a^RAG: retrieval-augmented generation.

## Discussion

### Overall Diagnostic Performance and the Effect of Clinical Personas

The primary and most counterintuitive finding of this study is that in complex medical image diagnostics, integrating an external knowledge base via RAG did not improve the VLM’s accuracy. Instead, paradoxically, it was associated with lower diagnostic accuracy in specific contexts [17,18]. This aligns with recent findings in information retrieval, which suggest that in highly specialized reasoning tasks, the retrieved guideline text coincided with a shift in the model’s final differential diagnosis away from the visually supported diagnosis [19]. Macroscopic data revealed that RAG integration precipitated a statistically significant drop in the radiologist persona’s top-1 accuracy (from 29% to 14%, *P*=.03).

The comparison of the two clinical personas suggested that the effect of RAG integration was not uniform across prompting conditions. While RAG integration was associated with a marked decrease in top-1 accuracy for the radiologist persona, the orthopedic oncologist persona demonstrated greater diagnostic stability, maintaining a stable top-3 accuracy (33%) regardless of RAG integration (*P*>.99). This finding should be interpreted cautiously because the study was exploratory and involved a single model and a small cohort.

One possible explanation is that the two system prompts may have encouraged different approaches to integrating clinical history and imaging findings. Purely radiological prompts may condition the model to engage in superficial “visual captioning,” making its output more susceptible to being influenced by retrieved text. Conversely, the orthopedic oncologist prompt inherently embeds complex clinical reasoning priorities (such as “ruling out malignancies”), which may contribute to the observed diagnostic stability. However, because the prompts were not systematically ablated and no independent measure of reasoning quality was available, the present study cannot determine whether the observed difference was caused by the persona specification itself. This finding therefore supports further investigation of prompt-conditioned robustness rather than establishing a definitive protective effect of a specific clinical persona [20].

### RAG-Associated Diagnostic Shifts and Rare-Disease Errors

An in-depth analysis of the model-generated reasoning traces revealed that the RAG mechanism frequently triggers “rare-disease hallucinations” and “overdifferential traps” during complex multimodal tasks. For instance, in case 4, the RAG-ortho oncologist misdiagnosed a chondroblastoma with a secondary aneurysmal bone cyst as an adamantinoma. In case 6, the RAG-radiologist misidentified a GCTB as intraosseous hydroxyapatite deposition disease. In case 12, the RAG-ortho oncologist concocted a diagnosis of brown tumor secondary to hyperparathyroidism for a simple intraosseous ganglion cyst. Furthermore, in case 29, the RAG-ortho oncologist erroneously attributed an osteochondroma to Maffucci syndrome or neurofibromatosis type 1 (NF1)-related calcific lesions.

Taking case 4 as a prime example, the patient’s true pathology was a femoral chondroblastoma accompanied by an aneurysmal bone cyst. However, the RAG-ortho oncologist model output a highly improbable final diagnosis of “adamantinoma” (Figures 1 and 2).

**Figure 1:**
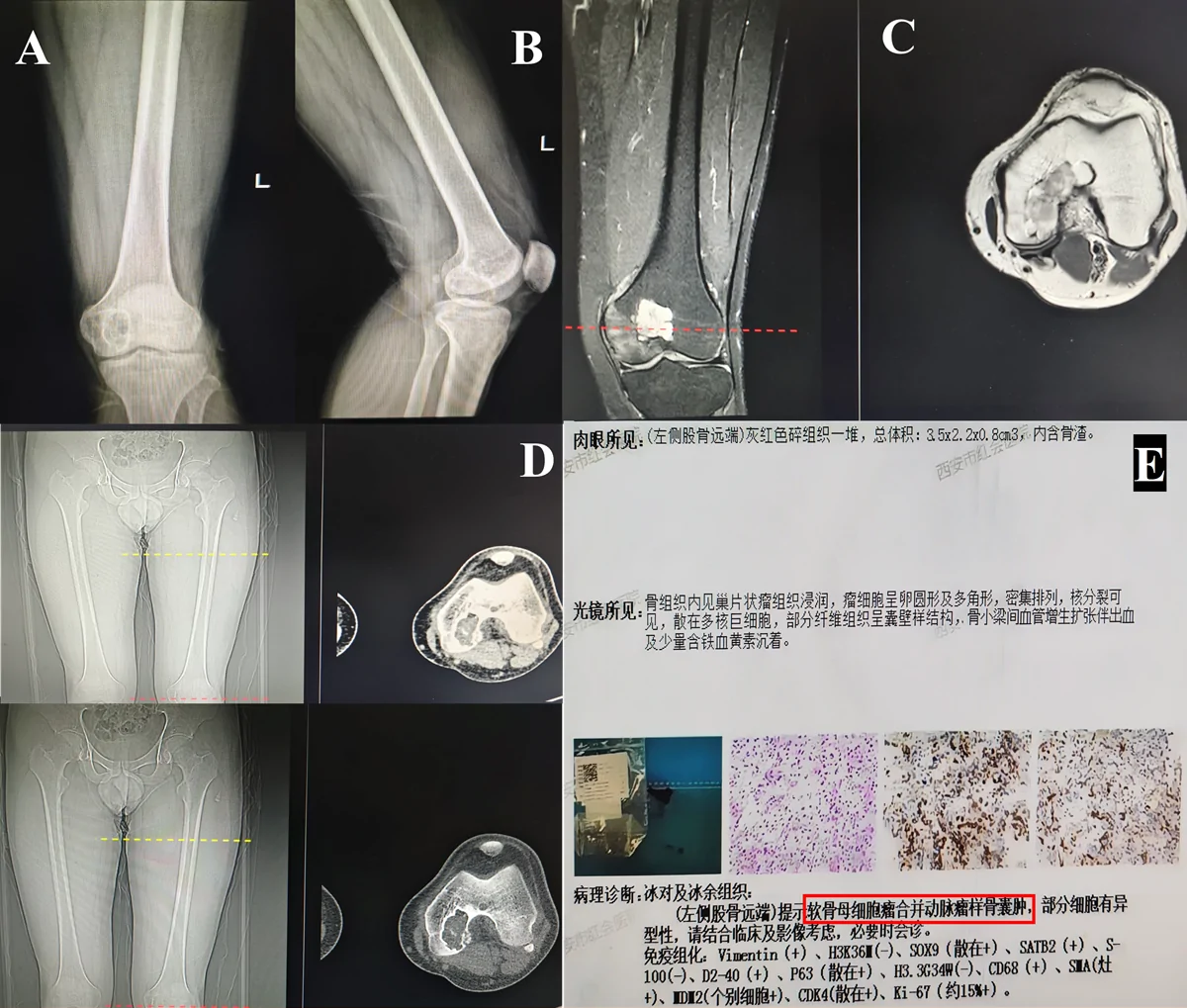
Ground truth and pathological confirmation of the distal femur lesion (chondroblastoma with secondary aneurysmal bone cyst): (A) anteroposterior radiograph, (B) lateral radiograph, (C) magnetic resonance imaging (MRI), (D) computed tomography (CT), and (E) histopathological examination results.

**Figure 2:**
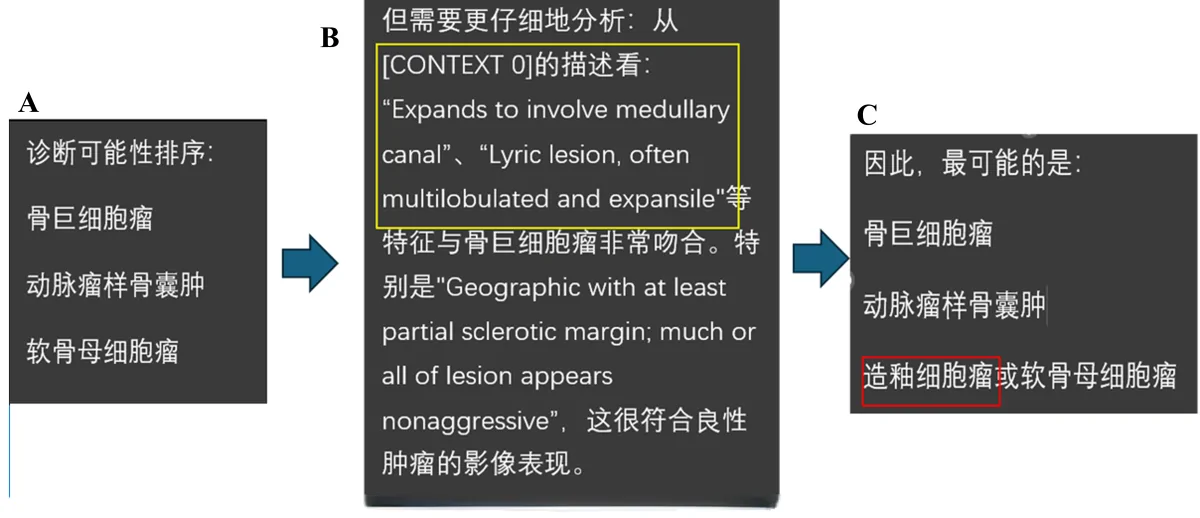
Artificial intelligence hallucination triggered by text-over-vision conflict: (A) initial visual-based intuition accurately identifying the lesion (chondroblastoma); (B) retrieval-augmented generation (RAG) integration injecting text priors, causing internal cognitive conflict; and (C) final output overriding objective visual features with a rare-disease hallucination (adamantinoma).

Epidemiological evidence establishes that long-bone adamantinoma is an exceedingly rare, low-grade malignant tumor, accounting for merely 0.1% to 0.4% of all primary bone tumors, and exhibits strict site specificity (80%-85% occur exclusively in the tibial diaphysis) [21,22]. Review of the reasoning traces revealed that the retrieved text coincided with a change in the model’s differential diagnosis. Despite earlier reasoning steps that had correctly identified chondroblastoma as a leading possibility based on visual features, the model elevated adamantinoma—an exceptionally rare tumor that mismatches the anatomical site (femur) in this case—into the top-3 diagnoses, while the visually supported chondroblastoma was dropped. This example illustrates a possible text-associated diagnostic shift, where the final diagnostic response became less consistent with the salient imaging features. However, the underlying computational mechanism cannot be definitively determined from the generated reasoning traces alone.

Consequently, we postulate that to uphold the complex persona of an “orthopedic oncology consultation expert,” the LLM attempted to demonstrate “professionalism” by encompassing a broader spectrum of rare differential diagnoses.

### Clinical Prior Anchoring and Imaging Feature Misinterpretation: The Influence of Trauma History

Do multimodal LLMs genuinely possess “vision-dominated” feature extraction capabilities when processing medical images, or are they merely engaging in “text-based probabilistic guessing”? Our data yields a sobering answer. Across all bone tumor cases accompanied by a brief but definitive history of trauma (cases 27, 38, 39, and 41), the 4 AI expert models almost uniformly ranked “fracture” or related traumatic injuries as the primary diagnosis. Furthermore, traumatic lesions predominantly occupied the second and third differential slots.

The stark contrast between case 27 and other typical osteosarcoma cases lacking a trauma history highlights a text-associated imaging misinterpretation. In case 27 (a 15-year-old male with a definitive trauma history), the patient’s true pathology was osteosarcoma complicated by a pathological fracture. Crucially, the MRI revealed a highly conspicuous, massive soft-tissue mass measuring approximately 7.3 × 3.9 × 12.9 cm (Figure 3).

**Figure 3:**
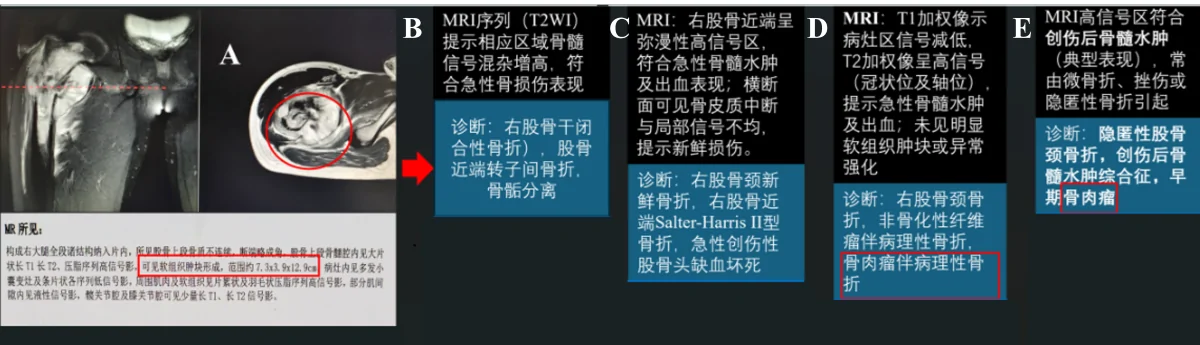
Trauma-induced visual blindness and diagnostic anchoring (case 27). (A) Ground truth MRI of a 15-year-old male demonstrating a massive (7.3 × 3.9 × 12.9 cm) soft-tissue mass and aggressive bone destruction (osteosarcoma). (B) Radiologist persona (base model) ignoring the massive tumor and anchoring on trauma. (C) Radiologist persona (RAG model) missing the tumor entirely due to trauma bias. (D) Orthopedic oncologist persona (base model) exhibiting text-associated diagnostic interference. (E) Orthopedic oncologist persona (RAG model) retaining the tumor in the differential despite trauma bias. MRI: magnetic resonance imaging; RAG: retrieval-augmented generation.

Yet, confronted with such a glaring malignant space-occupying lesion, the RAG-radiologist model remained completely mired in the cognitive trap of a “traumatic fracture.” It even exhibited a severe “reverse hallucination,” wherein the model deduced imaging features from its text-based diagnostic assumption: it forcibly misinterpreted the massive soft-tissue mass as “acute bone marrow edema and hemorrhage.” Further anchored by the patient’s age label of “15 years,” it fabricated the illusion of a “Salter-Harris type II fracture (physeal injury)” out of thin air. The base-radiologist fared no better, erroneously deriving conclusions of a “greenstick fracture” and “epiphyseal separation.”

In contrast, although the orthopedic oncologist persona (with or without RAG) also suffered severe interference from the trauma history, its internal instruction weighting to “rule out tumors” forced the model to reluctantly retain “bone tumor with pathological fracture” within its differential diagnosis. Nevertheless, it remained functionally blind to the obvious soft-tissue mass on the MRI, treating the malignant diagnosis merely as a probabilistic defensive strategy based on age demographics rather than genuine visual recognition.

### Epidemiological Probabilities Overriding Vision: Demographic Anchoring and Diagnostic “Text-Induced Visual Misinterpretations”

Beyond trauma history, patient demographics (particularly age and sex) constitute another fiercely stubborn cognitive anchor for VLMs. In standard clinical decision-making, it is a routine pathway to include metastatic bone disease in the differential diagnosis when an older adult presents with osteolytic lesions [23]. However, our findings indicate that VLMs suffer from severe overanchoring to this epidemiological probability. This bias becomes glaringly obvious in middle-aged and older patients presenting with classically benign imaging features. For biopsy-confirmed benign cases exhibiting geographic bone destruction with well-defined margins and sclerotic rims (eg, cases 11, 12, 13, 14, and 37), all 4 models almost uniformly ranked “bone metastasis” as their top diagnosis.

Take case 11 (a 47-year-old female with a bleeding simple bone cyst) as an example. Her x-ray and CT scans displayed an exceptionally regular cystic lesion, entirely lacking any aggressive malignant signs (Figure 4).

**Figure 4:**
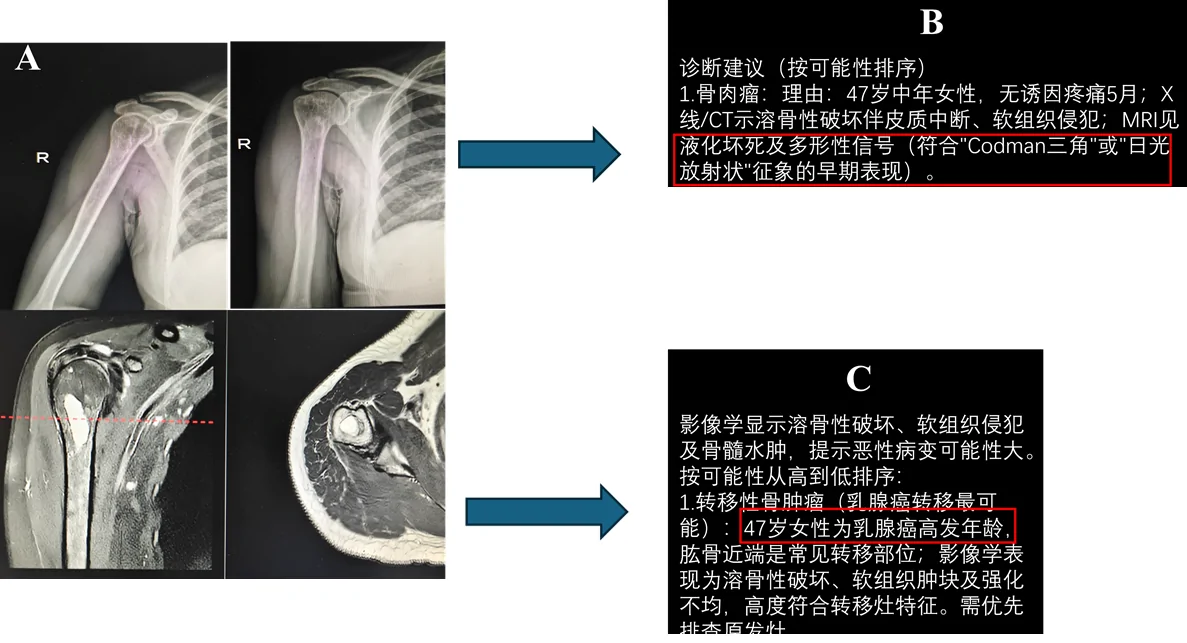
Reverse hallucination driven by epidemiological probabilities (case 11). (A) Ground truth imaging of a 47-year-old female showing a benign simple bone cyst with well-defined margins. (B) Radiologist persona hallucinating aggressive features (“Codman triangle”) to justify a highly improbable osteosarcoma diagnosis in an older adult. (C) Orthopedic oncologist persona blindly anchoring on the “47-year-old female” tag to force a breast cancer metastasis diagnosis without primary tumor history.

Yet, upon detecting the “47-year-old” tag, the model instantly triggered the preset logic of “older adult + bone destruction = metastasis.” To justify this conclusion, it incorrectly described malignant features, describing ill-defined margins and soft-tissue infiltration. This phenomenon demonstrates that the model does not perform independent pathophysiological deductions based on visual cues. Instead, when visual features clash with high-frequency text priors (age probability), the final diagnostic output appeared to be more strongly influenced by the textual demographic prior. In clinical practice, this behavior of “forcibly modifying visual perception using epidemiological probabilities” can easily precipitate the overtreatment of benign lesions.

Furthermore, the models exhibited a strong tendency toward “overextrapolation” driven by textual stereotypes. For instance, based solely on the patient’s sex (female) and lacking any history of a primary tumor, both the RAG and base orthopedic oncologist groups asserted with absolute certainty that “breast cancer bone metastasis is the most likely.” Meanwhile, the radiologist groups mechanically regurgitated the standard boilerplate of “breast/lung cancer metastasis.” This highlights that the text co-occurrence frequency of “osteolytic lesion in an older female” and “breast cancer bone metastasis” is exceptionally high within the training corpora. Consequently, the model’s final output appeared to place greater weight on the textual demographic information than on the salient visual features. Essentially, the model’s output did not reflect the actual cyst margins visible on the images. Instead, the final diagnostic output appeared to be strongly influenced by demographic information and was accompanied by imaging descriptions that were inconsistent with the observed features.

This exposes a core deficiency in current VLMs: confirmation bias [24]. Recent studies evaluating foundation models in electronic health records have increasingly warned about their systemic vulnerability to demographic anchoring, where models erroneously propagate demographic-based medical biases rather than analyzing individualized patient data [25,26]. Our findings vividly illustrate this risk in multimodal settings: Once the model locks onto a high-probability diagnosis via textual prompts (such as age, sex, or trauma history), the final diagnostic response becomes less consistent with the salient imaging features, pivoting instead to searching for textual evidence to fit the preset probabilistic answer. This suggests that, in atypical clinical scenarios, VLM responses may be substantially influenced by superficial medical history information, potentially increasing the risk of diagnostic error.

### Textbook Bias and False-Negative Traps: Atypical Demographics Masking Malignant Visual Features

Demographic anchoring does not merely result in an overestimation of malignancies (such as the aforementioned overdiagnosis of metastases) in older adults; equally alarming, it triggers severe “false-negative” missed diagnoses when malignant tumors present at an atypical age of onset.

The cohort of 13 osteosarcoma cases (cases 15-27) vividly exposes this vulnerability. When patient data perfectly align with the standard textbook profile—“10 to 20 years old, male, lesion located in the femur or tibia” (eg, cases 15, 18, 19, 24, 25, and 26)—all 4 models achieved exceptionally high diagnostic hit rates. However, the moment a patient’s epidemiological characteristics deviate from this pre-established track, the models frequently failed to identify even the most conspicuous malignant imaging signs.

Case 22 (a 42-year-old male with osteosarcoma of the distal radius and ulna) serves as a quintessential example. Radiographically, this case exhibited flagrant, aggressive bone destruction at the distal radius and ulna, accompanied by the formation of a massive soft-tissue mass (Figure 5).

**Figure 5:**
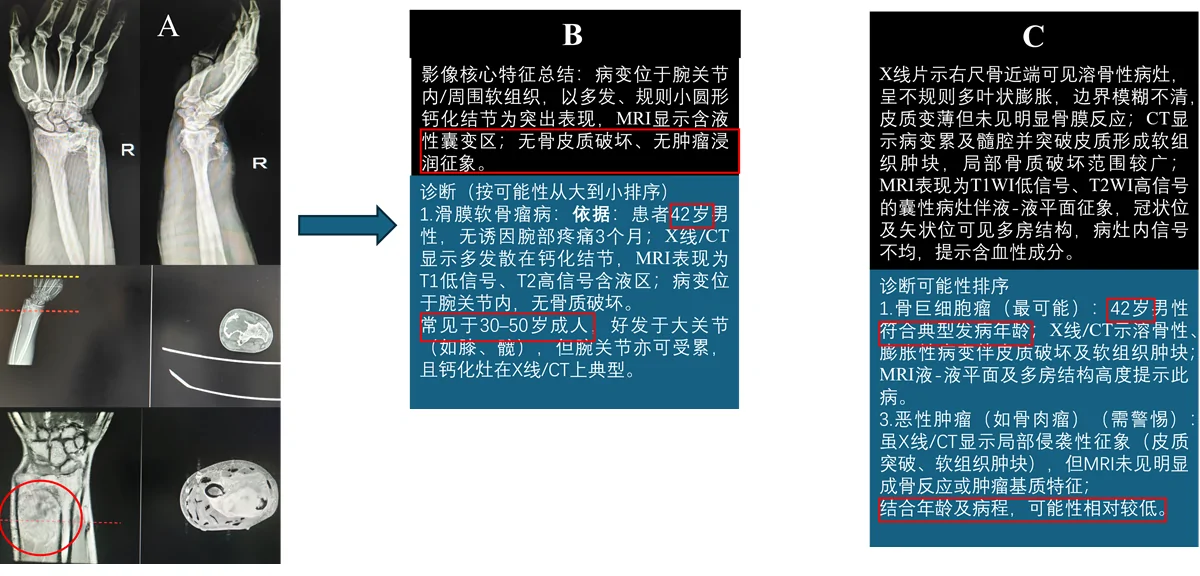
Textbook bias leading to a severe false-negative trap (case 22). (A) Ground truth imaging of a 42-year-old male demonstrating aggressive bone destruction and a massive soft-tissue mass (osteosarcoma). (B) Radiologist persona completely ignoring the tumor and hallucinating benign features to justify synovial chondromatosis. (C) Orthopedic oncologist persona recognizing aggressive features but downgrading the true malignancy because the patient’s age (42 years old) deviates from the classic textbook profile (10-20 years old) for osteosarcoma.

Notably, after processing the “42 years old” demographic tag and performing a knowledge retrieval, the RAG-radiologist model yielded “synovial chondromatosis”—a strictly benign condition—as its primary diagnosis. Even more absurdly, to align with the retrieved text, the model suffered a severe visual hallucination in its imaging description. It described incorrect features—such as “multiple small round/oval high-density opacities, scattered distribution, without bone destruction”—that directly contradicted the actual images.

Conversely, although the orthopedic oncologist personas (both base and RAG) successfully recognized critical aggressive visual features—such as “lesion involving the medullary cavity and breaking through the cortex to form a soft-tissue mass”—their final diagnostic reasoning was nevertheless hijacked by the “42-year-old” age prior. Since 20 to 40 years is the peak incidence age for GCTB, the models rigidly superimposed these malignant destructive signs onto GCTB, crowning it as the absolute first diagnosis. As for the true malignancy (osteosarcoma), the models merely relegated it to the third position with a dismissive caveat: “Considering the patient’s history and age, this is less likely.”

This stark contrast profoundly unmasks the imbalanced weighting logic internal to multimodal LLMs: the model is not “interpreting images to deduce a diagnosis,” but rather “using epidemiological tags to frame a presumptive diagnosis, and subsequently cherry-picking imaging fragments to endorse it.” If a patient fails to conform to classic epidemiological profiles, the model frequently underestimates the severity of the malignant lesion, going so far as to mischaracterize visual features to maintain its internal logical consistency. In the complexities of real-world clinical practice, this observed pattern may pose a potential clinical risk if similar behavior occurs in real-world diagnostic settings.

### Temporal Anchoring and Retrieval-Associated Diagnostic Errors

In the clinical differential diagnosis of bone tumors, disease duration often provides vital clues. However, when processing temporal adverbials, large models exhibit a highly mechanistic “time-axis cognitive rigidity.” In real-world clinical settings, many benign or borderline bone tumors (such as aneurysmal bone cysts or non-ossifying fibromas) frequently trigger sudden, acute, and severe pain due to intralesional hemorrhage or micropathological fractures [27]. Yet, multimodal large models evidently lack the capacity for such dynamic pathophysiological deduction. Their underlying logic crudely and absolutely equates a “disease duration of less than 1 week” with an “acute infection or fresh trauma.”

Rather than being mitigated, this misdiagnosis triggered by short disease duration devolved into a severe retrieval-associated diagnostic error following the introduction of RAG. This study provided the RAG models with *Diagnostic Imaging: Musculoskeletal Non-Traumatic Disease* as a reference knowledge base, initially anticipating that the models would use its differential diagnosis sections to better rule out infections. The outcome was entirely the opposite. When confronted with tumor patients presenting with a disease duration of less than 1 week (eg, cases 3, 5, and 16), the base models rarely misdiagnosed them as acute osteomyelitis. In stark contrast, the RAG expert models frequently and assertively nominated “acute osteomyelitis” as the core diagnosis.

Case 3 further illustrates how temporal text can disproportionately influence the differential diagnosis. The patient, a 6-year-old female, was diagnosed with an aneurysmal bone cyst of the left tibia, with a brief medical history indicating only “pain without obvious cause for 5 days” (Figure 6). Within the model-generated reasoning traces of the RAG-radiologist, the model referenced retrieved descriptions of pediatric osteomyelitis, explicitly citing its diagnostic rationale: “The history of unexplained pain for 5 days aligns with the progression timeline of a bacterial infection.” To forcibly cater to this retrieved textual time-axis, the model did not appropriately account for the unambiguously cystic tumor features on the images, rigidly misinterpreting them as “diffuse bone marrow edema.”

**Figure 6:**
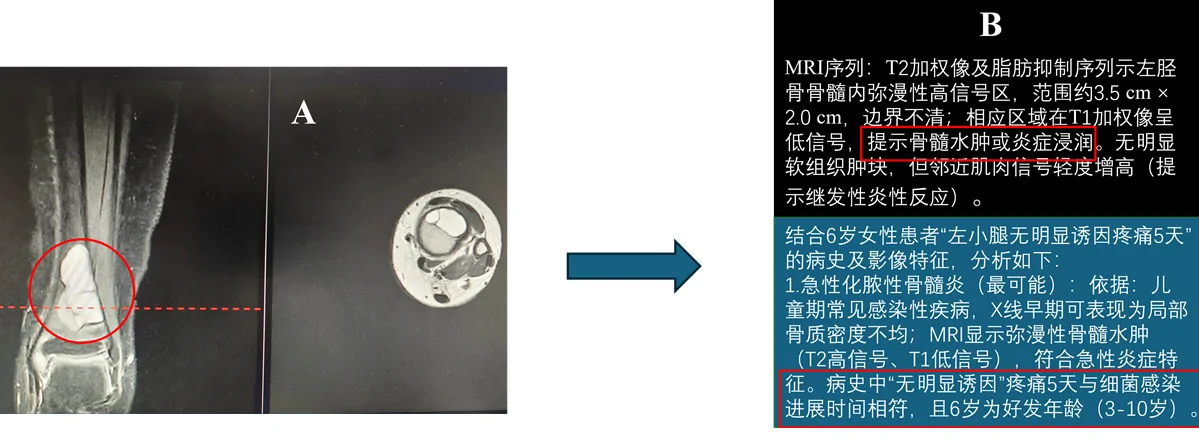
Temporal anchoring and retrieval-associated diagnostic error (case 3). (A) Cystic imaging features of the left tibial lesion in a 6-year-old female with aneurysmal bone cyst. (B) The retrieval-augmented generation–radiologist reasoning trace linked the 5-day history to an infectious differential and subsequently described imaging findings as “diffuse bone marrow edema.”.

This phenomenon highlights a critical limitation of the RAG mechanism in medical diagnostics: overfitting and context stripping. Upon receiving a strong prompt like “short disease duration,” the RAG system prioritizes retrieving chapters containing the keyword “acute” (eg, acute suppurative osteomyelitis). Subsequently, to ensure its output appears superficially plausible, the language model’s (LLM) generative logic forcibly binds the extraordinarily thin temporal clue (5 days) to the high-weight retrieved infection text. This demonstrates that in the absence of deep pathophysiological causal reasoning, blindly augmenting large models with external textbooks easily triggers severe, dogmatic misdiagnoses.

### Modality Imbalance: The “Shortcut Trap” Between High-Noise Images and Pristine Text

All the severe misdiagnoses triggered by text priors inevitably raise a core question regarding the underlying architecture: Why do multimodal large models rely so heavily on text, even to the point of turning a blind eye to flagrant malignant imaging features (such as a massive 13 cm soft-tissue mass)?

Our experimental design provides a “real-world” answer to this question. To maximally simulate the actual pain points of primary care hospitals and teleconsultations, this study deliberately used high-noise images obtained via “smartphone screen captures” (incorporating Moiré patterns, screen glare, and local distortion). Under such intensely challenging visual inputs, the challenging visual conditions may have increased the models’ reliance on structured textual information.

The observed errors may reflect a mismatch between degraded visual information and the retrieved textual context. In the evaluated setting, the model’s diagnostic responses sometimes appeared more strongly influenced by relatively structured clinical history or retrieved textual information than by salient imaging features [28]. However, because the study did not include clean-image controls or direct measurements of multimodal attention, the relative contribution of visual degradation, retrieval content, and model-level fusion mechanisms cannot be determined.

### Limitations

This study has several limitations. First, as a single-center retrospective study, the sample size is relatively small (42 biopsy-confirmed cases). Although the cohort covers an exceptionally rich spectrum of bone tumors, larger-scale, multicenter validation remains necessary. Second, the selection of 3-5 representative slices was performed by a single orthopedic oncology specialist and was not blinded or independently adjudicated, which may have introduced selection bias and limits generalizability to routine referral workflows. Third, no blinded human reader or contemporary VLM comparator was included, limiting the interpretation of absolute diagnostic accuracy. Fourth, because only smartphone-captured degraded images were evaluated, the study cannot determine whether the observed errors were solely attributable to image degradation itself. Fifth, we tested a single open-source VLM (Qwen3-VL); future research must conduct benchmarking across multiple models [29]. Sixth, regarding the RAG pipeline, querying an English-only knowledge base with Chinese clinical prompts relied entirely on the native cross-lingual semantic alignment of the embedding model, which may introduce subtle retrieval mismatches. Finally, because this was an exploratory study, we did not apply multiplicity corrections across all subgroup comparisons. The reported *P* values should therefore be interpreted as nominal, and the possibility of type I error inflation should be considered.

### Conclusions

In conclusion, in this exploratory study of 42 biopsy-confirmed bone tumors and tumor-like lesions—diagnoses that inherently rely on minute radiographic features [30,31]—the evaluated RAG configuration did not improve diagnostic accuracy under smartphone-captured degraded imaging conditions. A nominally significant decrease in top-1 accuracy was observed for the radiologist persona, whereas no significant change was observed for the orthopedic oncologist persona. Exploratory review of model-generated reasoning traces identified several text-associated diagnostic error patterns, although the underlying computational mechanisms could not be established. These findings suggest that retrieval configuration and clinical persona may influence VLM behavior under degraded visual conditions, but require validation in larger, multicenter, and multimodel studies. Future work should evaluate whether targeted visual fine-tuning using representative real-world degraded medical images can improve robustness in such settings [29].

## Data Availability

The datasets generated and analyzed during this study are not publicly available because they contain clinical and medical imaging data subject to patient privacy and institutional data protection requirements. Data may be available from the corresponding author upon reasonable request, subject to institutional and ethical approval.

## Appendix

Multimedia Appendix 1 Original input images, part 1.

Multimedia Appendix 2 Original input images, part 2.

Multimedia Appendix 3 Original input images, part 3.

Multimedia Appendix 4 Original input images, part 4.

Multimedia Appendix 5 Original input images, part 5.

Multimedia Appendix 6 Detailed case-by-case data.

Multimedia Appendix 7 Large language model outputs, part 1.

Multimedia Appendix 8 Large language model outputs, part 2.

Multimedia Appendix 9 STARD-AI checklist.

## Abbreviations

CT: computed tomography
DICOM: Digital Imaging and Communications in Medicine
GCTB: giant cell tumor of bone
LCD: liquid crystal display
LLM: large language model
MRI: magnetic resonance imaging
NCCN: National Comprehensive Cancer Network
PACS: picture archiving and communication system
RAG: retrieval-augmented generation
STARD-AI: Standards for Reporting of Diagnostic Accuracy Studies–Artificial Intelligence
VLM: vision language model
