# Supplementary material for: Diagnostic Performance of a Locally Deployed Vision Language Model for Bone Tumor Diagnosis Using Smartphone-Captured Images: Exploratory Retrospective Study
Source: JMIR AI. 2026 Sep 21;5:e99757. doi: 10.2196/99757 (PMC13639421; doi:10.2196/99757)
Supplement: Supplementary file 1 [file ai_v5i1e99757_app6.docx]

**Multimedia Appendix 6.** Detailed case-by-case data.

| **Patient ID** | **Gender** | **Age** | **Lesion Location** | **Trauma History (Y/N)** | **Duration** | **Pathology (Ground Truth)** | **Base-Radiologist Top-3** | **RAG-Radiologist Top-3** | **Base-Ortho Oncologist Top-3** | **RAG-Ortho Oncologist Top-3** |
| --- | --- | --- | --- | --- | --- | --- | --- | --- | --- | --- |
| 1 | F | 62 | Right middle finger | N | 4 years | Epidermoid cyst | Enchondroma, Degenerative joint disease, Osteochondroma | Synovial chondromatosis, Tendon calcification, Degenerative osteophytes with loose bodies | Osteochondroma, Enchondroma, Chondroblastoma | Calcific tendinitis of extensor tendon, Interphalangeal osteoarthritis, Gouty tophi |
| 2 | F | 11 | Left fibula | N | 6 months | Aneurysmal bone cyst (ABC) | Non-ossifying fibroma, Bone cyst, Eosinophilic granuloma | Osteosarcoma, Ewing sarcoma, Aneurysmal bone cyst | Osteosarcoma, Ewing sarcoma, Aneurysmal bone cyst | Bone cyst, Aneurysmal bone cyst, Giant cell tumor of bone |
| 3 | F | 5 | Left tibia | N | 5 days | Aneurysmal bone cyst (ABC) | Bone cyst, Non-ossifying fibroma, Giant cell tumor of bone | Acute osteomyelitis, Non-ossifying fibroma, Eosinophilic granuloma | Non-ossifying fibroma, Bone cyst, Giant cell tumor of bone | Ewing sarcoma, Non-ossifying fibroma, Acute osteomyelitis |
| 4 | M | 23 | Left femur | N | 2 years | Chondroblastoma with secondary ABC | Chondroblastoma, Osteochondroma, Chondrosarcoma | Synovial chondromatosis, Multiple osteochondromas, Osteosarcoma | Chondroblastoma, Giant cell tumor of bone, Non-ossifying fibroma | Giant cell tumor of bone, Aneurysmal bone cyst, Adamantinoma |
| 5 | F | 22 | Left tibia | N | 2 days | Non-ossifying fibroma (NOF) | Giant cell tumor of bone, Aneurysmal bone cyst, Osteosarcoma | Osteosarcoma, Ewing sarcoma, Acute osteomyelitis | Giant cell tumor of bone, Aneurysmal bone cyst, Chondrosarcoma | Enchondroma, Eosinophilic granuloma, Non-ossifying fibroma |
| 6 | F | 25 | Right fibula | N | 1 month | Giant cell tumor of bone (GCTB) | Giant cell tumor of bone, Chondroblastoma, Non-ossifying fibroma | Synovial chondromatosis, Intraosseous hydroxyapatite deposition disease, Osteochondritis | Aneurysmal bone cyst, Bone cyst, Giant cell tumor of bone | Osteoid osteoma, Osteochondroma, Non-ossifying fibroma |
| 7 | F | 63 | Left tibia | N | 4 months | Giant cell tumor of bone (GCTB) | Enchondroma, Bone metastasis, Fibrous dysplasia | Bone cyst, Clear cell chondrosarcoma, Intraosseous lipoma | Bone metastasis, Multiple myeloma, Chondrosarcoma | Bone cyst, Aneurysmal bone cyst, Bone metastasis |
| 8 | F | 57 | Left tibia | N | 1 month | Giant cell tumor of bone (GCTB) | Giant cell tumor of bone, Bone metastasis, Bone cyst | Giant cell tumor of bone, Chondrosarcoma, Bone metastasis | Breast cancer bone metastasis, Multiple myeloma, Dedifferentiated chondrosarcoma | Synovial chondromatosis, Chondrosarcoma, Multiple osteochondromas |
| 9 | M | 31 | Right tibia | N | 20 days | GCTB with secondary ABC | Giant cell tumor of bone, Aneurysmal bone cyst, Chondroblastoma | Enchondroma, Chondrosarcoma, Giant cell tumor of bone | Osteosarcoma, Ewing sarcoma, Giant cell tumor of bone | Giant cell tumor of bone, Aneurysmal bone cyst, Osteosarcoma |
| 10 | F | 32 | Right tibia | N | 6 months | Giant cell tumor of bone (GCTB) | Giant cell tumor of bone, Aneurysmal bone cyst, Chondroblastoma | Giant cell tumor of bone, Aneurysmal bone cyst, Osteosarcoma | Osteosarcoma, Chondrosarcoma, Giant cell tumor of bone | Aneurysmal bone cyst, Giant cell tumor of bone, Osteosarcoma |
| 11 | F | 47 | Right humerus | N | 5 months | Simple bone cyst (SBC) | Osteosarcoma, Chondrosarcoma, Bone metastasis | Osteosarcoma, Bone metastasis, Giant cell tumor of bone | Bone metastasis, Chondrosarcoma, Aneurysmal bone cyst | Bone metastasis, Chondrosarcoma, Multiple myeloma |
| 12 | F | 56 | Right humerus | N | 2 days | Intraosseous ganglion cyst | Bone metastasis, Multiple myeloma, Acute osteomyelitis | Synovial chondromatosis, Chondrosarcoma, Synovitis with secondary calcification | Bone metastasis, Chondrosarcoma, Giant cell tumor of bone | Giant cell tumor of bone, Chondroblastoma, Brown tumor secondary to hyperparathyroidism |
| 13 | M | 44 | Right femur | N | 2 months | Intraosseous hemangioma | Bone metastasis, Chondrosarcoma, Chronic osteomyelitis | Bone metastasis, Giant cell tumor of bone, Intracortical hydroxyapatite deposition disease | Osteosarcoma, Chondrosarcoma, Bone metastasis / Chronic osteomyelitis | Synovial chondromatosis, Aggressive chondroma, Bone island |
| 14 | F | 73 | Right femur | N | 1 month | Intraosseous hemangioma | Bone cyst, Aneurysmal bone cyst, Bone metastasis | Bone cyst, Aneurysmal bone cyst, Bone metastasis | Bone cyst, Bone metastasis, Aneurysmal bone cyst | Bone metastasis, Multiple myeloma, Giant cell tumor of bone |
| 15 | M | 14 | Left femur | N | 1 month | Osteosarcoma | Osteosarcoma, Ewing sarcoma, Non-ossifying fibroma | Osteosarcoma, Ewing sarcoma, Langerhans cell histiocytosis | Osteosarcoma, Ewing sarcoma, Giant cell tumor of bone | Non-ossifying fibroma, Aneurysmal bone cyst, Osteosarcoma |
| 16 | F | 64 | Right tibia and fibula | N | 2 days | Spindle cell tumor (suspected low-grade osteosarcoma) | Bone metastasis, Pleomorphic osteosarcoma, Giant cell tumor of bone | Bone metastasis, Osteosarcoma, Malignant giant cell tumor of bone | Bone metastasis, Multiple myeloma, Chronic osteomyelitis | Bone metastasis, Chondrosarcoma, Acute osteomyelitis |
| 17 | F | 6 | Left tibia | N | 6 months | Osteosarcoma | Eosinophilic granuloma, Non-ossifying fibroma, Chronic osteomyelitis | Non-ossifying fibroma, Chronic osteomyelitis, Osteoid osteoma | Ewing sarcoma, Osteoblastoma, Malignant fibrous histiocytoma | Ewing sarcoma, Osteosarcoma, Aneurysmal bone cyst |
| 18 | M | 16 | Right femur | N | 1 month | Osteosarcoma | Osteosarcoma, Ewing sarcoma, Giant cell tumor of bone | Giant cell tumor of bone, Aneurysmal bone cyst, Chondrosarcoma | Osteosarcoma, Ewing sarcoma, Giant cell tumor of bone | Osteosarcoma, Ewing sarcoma, Giant cell tumor of bone |
| 19 | M | 19 | Left femur | N | 3 months | Osteosarcoma | Giant cell tumor of bone, Aneurysmal bone cyst, Chondrosarcoma | Giant cell tumor of bone, Aneurysmal bone cyst, Osteosarcoma | Osteosarcoma, Ewing sarcoma, Giant cell tumor of bone | Osteosarcoma, Ewing sarcoma, Giant cell tumor of bone |
| 20 | M | 42 | Right humerus | N | 8 months | Osteosarcoma | Chondrosarcoma, Osteosarcoma, Bone metastasis | Synovial chondromatosis, Bone infarct with secondary calcification, Bone metastasis | Chondrosarcoma, Osteosarcoma, Giant cell tumor of bone | Giant cell tumor of bone, Chondrosarcoma, Malignant transformation of ABC |
| 21 | M | 15 | Left femur | N | 2 months | Osteosarcoma | Bone island, Aneurysmal bone cyst, Chondrosarcoma | Giant cell tumor of bone, Osteosarcoma, Aneurysmal bone cyst | Osteosarcoma, Ewing sarcoma, Non-ossifying fibroma | Non-ossifying fibroma, Langerhans cell histiocytosis, Giant cell tumor of bone |
| 22 | M | 42 | Right radius and ulna | N | 3 months | Osteosarcoma | Giant cell tumor of bone, Chondrosarcoma, Aneurysmal bone cyst | Synovial chondromatosis, Post-traumatic synovial chondromatosis, Chondrosarcoma | Giant cell tumor of bone, Chondrosarcoma, Aneurysmal bone cyst | Giant cell tumor of bone, Aneurysmal bone cyst, Malignant tumor (e.g., Osteosarcoma) |
| 23 | M | 65 | Left tibia | N | 1 month | Small cell osteosarcoma | Bone metastasis, Chronic osteomyelitis, Dedifferentiated osteosarcoma | Enchondroma, Chondroblastoma, Osteochondroma | Bone metastasis, Chronic osteomyelitis, Giant cell tumor of bone | Synovial chondromatosis, Chondrosarcoma, Multiple osteochondromas |
| 24 | M | 15 | Left femur | N | 1 month | Osteosarcoma | Osteosarcoma, Ewing sarcoma, Langerhans cell histiocytosis | Non-ossifying fibroma, Aneurysmal bone cyst, Bone cyst | Osteosarcoma, Ewing sarcoma, Non-ossifying fibroma | Osteosarcoma, Ewing sarcoma, Malignant transformation of NOF |
| 25 | M | 15 | Right femur | N | 7 months | Osteosarcoma | Osteosarcoma, Ewing sarcoma, Malignant fibrous histiocytoma | Synovial chondromatosis, Giant cell tumor of bone, Osteochondroma | Osteosarcoma, Ewing sarcoma, Aneurysmal bone cyst | Osteosarcoma, Malignant fibrous histiocytoma, Giant cell tumor of bone |
| 26 | F | 12 | Right femur | N | 1 month | Osteosarcoma | Osteosarcoma, Ewing sarcoma, Non-ossifying fibroma | Non-ossifying fibroma, Aneurysmal bone cyst, Giant cell tumor of bone | Osteosarcoma, Ewing sarcoma, Aneurysmal bone cyst | Osteosarcoma, Ewing sarcoma, Aneurysmal bone cyst |
| 27 | M | 15 | Right femur | Y | 1 day | Osteosarcoma | Closed fracture of right femoral shaft, Intertrochanteric fracture, Epiphyseal separation (physeal injury) | Right femoral neck fracture, Salter-Harris II fracture, Early acute traumatic avascular necrosis | Right femoral neck fracture, NOF with pathological fracture, Osteosarcoma with pathological fracture | Occult femoral neck fracture, Post-traumatic bone marrow edema, Osteosarcoma |
| 28 | F | 59 | Left knee | N | Mass for 7 years, enlarging over 4 months | Osteochondroma | Lipoma, Schwannoma, Hemangioma | Popliteal cyst, Schwannoma, Lipoma | Lipoma, Schwannoma, Liposarcoma | Giant cell tumor of bone, Chondrosarcoma, Aneurysmal bone cyst |
| 29 | F | 36 | Right humerus | N | 30 years | Osteochondroma | Multiple osteochondromas, Chondrosarcoma, Fibrous dysplasia with calcification | Hereditary multiple exostoses, Chondrosarcoma, Polyostotic fibrous dysplasia | Multiple enchondromatosis, Maffucci syndrome, NF1-related calcific lesions | Synovial chondromatosis, Multiple osteochondromas, Chondrosarcoma |
| 30 | M | 26 | Right fibula/tibia/distal femur | N | 20 years | Osteochondroma | Multiple osteochondromas, Osteochondroma, Chondrosarcoma | Osteochondroma, Chondroblastoma, Fibrous dysplasia | Osteochondroma, Chondrosarcoma, Giant cell tumor of bone | Multiple osteochondromas, Fibrous dysplasia, Enchondroma |
| 31 | M | 26 | Right femur | Y | 1 week | Fibrous dysplasia | Osteosarcoma, Ewing sarcoma, Acute osteomyelitis | Osteosarcoma, Giant cell tumor of bone, Parosteal osteosarcoma | Giant cell tumor of bone, Aneurysmal bone cyst, Chondroblastoma | Giant cell tumor of bone, Aneurysmal bone cyst, Chondrosarcoma |
| 32 | M | 48 | Right femur | N | Hip pain for 2 years, worsening for 2 months | Fibrous dysplasia | Giant cell tumor of bone, Bone metastasis, Osteosarcoma | Chondrosarcoma, Giant cell tumor of bone, Bone metastasis | Osteosarcoma, Bone metastasis, Giant cell tumor of bone | Giant cell tumor of bone, Chondrosarcoma, Bone metastasis |
| 33 | M | 54 | Right fibula | N | 2 months | Fibrous dysplasia | Bone metastasis, Giant cell tumor of bone, Non-ossifying fibroma | Fibrous dysplasia, Aneurysmal bone cyst, Chondrosarcoma | Bone metastasis, Giant cell tumor of bone, Osteosarcoma | Giant cell tumor of bone, Aneurysmal bone cyst, Bone metastasis |
| 34 | F | 62 | Right humerus | N | 1 week | Fibrous dysplasia | Bone metastasis, Multiple myeloma, Giant cell tumor of bone | Bone metastasis, Multiple myeloma, Chronic osteomyelitis | Osteosarcoma, Bone metastasis, Giant cell tumor of bone | Synovial chondromatosis, Multiple enchondromatosis, Calcific tendinitis with secondary changes |
| 35 | M | 16 | Left femur | N | 1 year | Osteoid osteoma | Osteosarcoma, Ewing sarcoma, Aneurysmal bone cyst | Aneurysmal bone cyst, Giant cell tumor of bone, Non-ossifying fibroma | Osteosarcoma, Ewing sarcoma, Aneurysmal bone cyst | Aneurysmal bone cyst, Fibrous dysplasia, Chronic osteomyelitis |
| 36 | F | 37 | Right femur | N | 10 years | Liposclerosing myxofibrous tumor (LSMFT) | Fibrous dysplasia, Chronic osteomyelitis, Chondroblastoma | Synovial chondromatosis, Fibrous dysplasia, Osteochondroma | Giant cell tumor of bone, Aneurysmal bone cyst, Bone cyst | Giant cell tumor of bone, Aneurysmal bone cyst, Chronic osteomyelitis |
| 37 | F | 52 | Left femur | N | 1 month | Enostosis (Bone island) | Osteosarcoma, Bone metastasis, Giant cell tumor of bone | Bone metastasis, Chondrosarcoma, Giant cell tumor of bone | Bone metastasis, Chondrosarcoma, Giant cell tumor of bone | Chondrosarcoma, Giant cell tumor of bone, Bone metastasis |
| 38 | F | 41 | Right middle finger | Y | Pain for 9 hours after a fall | Enchondroma | Greenstick fracture of proximal phalanx, MCP joint subluxation, Flexor tendon contusion with periosteal reaction | Fracture of proximal phalanx base, Bone contusion with bone marrow edema, Collateral ligament injury | Closed fracture of proximal phalanx, Pathological fracture secondary to benign tumor, PIP joint dislocation with soft tissue contusion | Acute comminuted fracture of proximal phalanx, MCP joint dislocation with soft tissue injury, Extensor tendon contusion or partial tear |
| 39 | F | 66 | Left little finger | Y | Pain and limited mobility for 3 days after minor exertion | Enchondroma | Stress fracture, Osteoporosis-related microfracture, Acute osteomyelitis | Fatigue fracture of proximal phalanx base, Partial tear of extensor tendon with inflammation, Acute osteoarthritis exacerbation with bone contusion | Fracture of distal phalanx, Osteoporotic stress fracture, Enchondroma with pathological fracture | Stress fracture of phalanx, Traumatic incomplete fracture, Intraosseous ganglion cyst |
| 40 | F | 63 | Left ilium | N | 40 days | Undifferentiated pleomorphic sarcoma (UPS) | Bone metastasis, Multiple myeloma, Osteosarcoma | Bone metastasis, Multiple myeloma, Primary plasmacytoma | Bone metastasis, Multiple myeloma, Primary plasmacytoma | Bone metastasis, Multiple myeloma, Osteosarcoma |
| 41 | M | 54 | Right femur | Y | 8 hours | Undifferentiated sarcoma | Acute traumatic fracture of femoral shaft, Osteoporotic pathological fracture, Pathological fracture secondary to osteosarcoma | Comminuted traumatic fracture of femoral shaft, Osteoporotic femur fracture, Post-traumatic bone marrow edema with soft tissue injury | Comminuted fracture of femoral shaft, Osteoporotic fracture, Pathological fracture (Bone metastasis) | Pathological fracture of femoral shaft, Pathological fracture secondary to synovial chondromatosis, Bone island/Fibrous dysplasia with acute trauma |
| 42 | M | 18 | Left tibia | N | 9 months | Undifferentiated sarcoma | Osteosarcoma, Ewing sarcoma, Giant cell tumor of bone | Osteosarcoma, Ewing sarcoma, Aneurysmal bone cyst | Osteosarcoma, Ewing sarcoma, Malignant fibrous histiocytoma | Non-ossifying fibroma, Aneurysmal bone cyst, Low-grade chondroblastoma |
