# Supplementary material for: Diagnostic Performance of a Locally Deployed Vision Language Model for Bone Tumor Diagnosis Using Smartphone-Captured Images: Exploratory Retrospective Study
Source: JMIR AI. 2026 Sep 21;5:e99757. doi: 10.2196/99757 (PMC13639421; doi:10.2196/99757)
Supplement: Supplementary file 2 [file ai_v5i1e99757_app9.docx]

**STARD-AI Checklist**

| **Section and Topic** | | **No.** | | **STARD-AI Item** | | **Reported on page** | |  |
| --- | --- | --- | --- | --- | --- | --- | --- | --- |
| **Title or abstract** | |  | |  | |  | |  |
|  | | 1 | | Identification as a study reporting AI-centred diagnostic accuracy and reporting at least one measure of accuracy within title or abstract | | Title / Abstract | |  |
| **Abstract** | |  | |  | |  | |  |
|  | | 2 | | Structured summary of study design, methods, results and conclusions (for specific guidance, please see STARD for Abstracts) | | Abstract | |  |
| **Introduction** | |  | |  | |  | |  |
|  | | 3 | | Scientific and clinical background, including the intended use of the index test, whether it is novel or an established index test, and its integration into an existing or new workflow, if applicable | | Introduction- Background | |  |
|  |  | 4 | | Study objectives and hypotheses | | Introduction - Objectives | |  |
| **Methods** | |  | |  | |  | |  |
| Study design | | 5 | | Whether data collection was planned before the index test and reference standard were performed (prospective study) or after (retrospective study) | | Methods - Sample Set Construction | |  |
| Ethics | | 6 | | Formal approval from an ethics committee. If not required, justify why | | Methods - Ethics Statement | |  |
| Participants | | 7 | | Eligibility criteria: listing separate inclusion and exclusion criteria in the order that they are applied at both participant level and data level | | Methods - Sample Set Construction | |  |
|  |  | 8 | | On what basis potentially eligible participants were identified (such as symptoms, results from previous tests, inclusion in registry) | | Methods - Sample Set Construction | |  |
|  |  | 9 | | Where and when potentially eligible participants were identified (setting, location, and dates) | | Methods - Sample Set Construction | |  |
|  |  | 10 | | Whether participants formed a consecutive, random, or convenience series | | Methods - Sample Set Constructio | |  |
| Dataset | | 11 | | Source of the data and whether it has been routinely collected, specifically collected for the purpose of the study or acquired from an open-source repository | | Methods - Sample Set Construction | |  |
|  | | 12 | | Who undertook the annotations for the dataset (including experience levels and background) and how (within the same clinical context or in a post-hoc fashion), if applicable | | Methods - Statistical Analysis | |  |
|  |  | 13 | | Devices (manufacturer, model) that were used to capture data; software (with version number) used to engineer the index test, highlighting the intended use | | Hardware Setup / Model Deployment | |  |
|  |  | 14 | | Data acquisition protocols (e.g. contrast protocol or reconstruction method for medical images) and details of data preprocessing in sufficient detail to allow replication | | Methods - Hardware Setup | |  |
| Test methods | | 15a | | Index test, in sufficient detail to allow replication | | Methods - Model Deployment / Prompt Engineering | |  |
|  | | 15b | | How the index test was developed, including any training, validation, testing and external evaluation, detailing sample sizes, when applicable | | N/A (Pre-trained open-source model used) | |  |
|  |  | 15c | | Definition of and rationale for test positivity cut-offs or result categories of the index test, distinguishing pre-specified from exploratory | | Methods - Statistical Analysis | |  |
|  |  | 15d | | The specified end user of the index test and the level of expertise required of users | | Methods - Model Deployment | |  |
|  |  | 16a | | Reference standard, in sufficient detail to allow replication | | Methods - Sample Set Construction / Statistical Analysis | |  |
|  |  | | 16b | | Rationale for choosing the reference standard (if alternatives exist) | | Introduction - Background | |
|  |  |  | 16c | | Definition of and rationale for test positivity cut-offs or result categories of the reference standard, distinguishing pre-specified from exploratory | | Methods - Statistical Analysis | |
|  |  |  | 17a | | Whether clinical information and reference standard results were available to the performers or readers of the index test | | Methods - Prompt Engineering | |
|  |  |  | 17b | | Whether clinical information and index test results were available to the assessors of the reference standard | | N/A (Routine biopsy ground truth) | |
|  | Analysis | | 18 | | Methods for estimating or comparing measures of diagnostic accuracy | | Methods - Statistical Analysis | |
|  |  |  | 19 | | How indeterminate index test or reference standard results were handled | | N/A | |
|  |  |  | 20 | | How missing data on the index test and reference standard were handled | | N/A | |
|  |  |  | 21 | | Any analyses of variability in diagnostic accuracy, distinguishing pre-specified from exploratory | | Results - Exploratory Analysis | |
|  |  |  | 22 | | Intended sample size and how it was determined | | N/A (Exploratory convenience sample) | |
|  |  |  | 23 | | Details of any performance error analysis, and algorithmic bias and fairness assessments if undertaken | | Results - Exploratory Analysis | |
|  | **Results** | |  | |  | | | |
|  | Participants and dataset | | 24 | | Flow of participants, using a diagram | | N/A (Textual description provided) | |
|  |  |  | 25 | | Baseline demographic, clinical and technical characteristics of training, validation and test set, if applicable | | Results - Baseline Characteristics / Table 2 | |
|  |  |  | 26a | | Distribution of severity of disease in those with the target condition | | Results - Baseline Characteristics | |
|  |  |  | 26b | | Distribution of alternative diagnoses in those without the target condition | | Results - Baseline Characteristics | |
|  |  |  | 27 | | Time interval and any clinical interventions between index test and reference standard | | N/A | |
|  |  |  | 28 | | Whether the datasets represent the distribution of the target condition that one would expect from the intended use population | | Discussion - Limitations | |
|  |  |  | 29 | | For external evaluation on an independent dataset, an assessment of how this differs from the training, validation and test sets | | N/A | |
|  | Test results | | 30 | | Cross tabulation of the index test results (or their distribution) by the results of the reference standard | | Results - Table 4 | |
|  |  |  | 31 | | Estimates of diagnostic accuracy and their precision (such as 95% confidence intervals) | | Results - Table 3 | |
|  |  |  | 32 | | Any adverse events from performing the index test or the reference standard | | N/A | |
|  | **Discussion** | |  | |  | | | |
|  |  | | 33 | | Study limitations, including sources of potential bias, statistical uncertainty, and generalisability | | Discussion - Limitations | |
|  |  |  | 34 | | Implications for practice, including the intended use and clinical role of the index test | | Discussion - Conclusions | |
|  |  |  | 35 | | Ethical considerations and adherence to ethical standards associated with the use of the index test and issues of fairness | | Methods – Ethics Statement | |
|  | **Other information** | |  | |  | | | |
|  |  | | 36 | | Registration number and name of registry | | N/A | |
|  |  |  | 37 | | Where the full study protocol can be accessed | | N/A | |
|  |  |  | 38 | | Sources of funding and other support; role of funders | | Funding Statement | |
|  |  |  | 39 | | Commercial interests, if applicable | | Conflicts of Interest | |
|  |  |  | 40a | | Availability of datasets and code; detailing any restrictions on their reuse and repurposing | | Data Availability | |
|  |  |  | 40b | | Whether outputs are stored, auditable and available for evaluation, if necessary | | Data Availability | |
