# Supplementary material for: Diagnostic Performance of a Locally Deployed Vision Language Model for Bone Tumor Diagnosis Using Smartphone-Captured Images: Exploratory Retrospective Study
Source: JMIR AI. 2026 Sep 21;5:e99757. doi: 10.2196/99757 (PMC13639421; doi:10.2196/99757)
Supplement: Supplementary file 8 [file ai_v5i1e99757_app7.zip › Case_11/Base/Ortho Oncologist.docx]

身份设定：你是一名省级三甲医院骨肿瘤科会诊专家（精通各类骨肿瘤与骨病影像鉴别）。请基于提供的X线、CT及MRI影像和极简病史，给出影像学研判。病变位置：右肱骨 患者信息：女性 47岁 右上臂近端无诱因疼痛5个月。要求：影像学描述：客观描述你观察到的核心影像学特征。结合病史与影像，按可能性从大到小，列出最可能的 3 个具体疾病诊断。临床建议：给出下一步的具体诊疗或处置建议。

首先，用户要求我作为省级三甲医院骨肿瘤科会诊专家，基于提供的X线、CT及MRI影像和极简病史，给出影像学研判。病变位置是右肱骨，患者信息：女性47岁，右上臂近端无诱因疼痛5个月。

关键点：

- 影像学描述：客观描述观察到的核心影像学特征。
- 结合病史与影像，按可能性从大到小列出最可能的3个具体疾病诊断。
- 临床建议：给出下一步的具体诊疗或处置建议。

现在，分析提供的图像：

1. **X线图像（前两张）**：
   - 第一张和第二张是右肱骨的X光片。标记为"R"表示右侧。
   - 在X光片上，可以看到右肱骨近端（靠近肩关节处）有异常。在第一张图中，有一个区域看起来像模糊或不规则，可能表明病变。第二张图更清晰，显示了肱骨头和颈部的细节。
   - 从图像看，在肱骨近端似乎有一个溶骨性病变（bone destruction），可能伴有轻微膨胀。但需要仔细观察。
2. **CT图像（第三张和第四张）**：
   - 第三张：左侧是X光，右侧是CT横断面。在CT上，可以看到右肱骨近端有一个病灶，显示为低密度区（溶骨性），可能有不规则边缘。
   - 第四张：类似，但CT图像更清晰地显示了病变的细节。在横断面上，病变区域似乎有破坏性变化，可能涉及髓腔。
3. **MRI图像（第五张）**：
   - 左侧是冠状位或矢状位，右侧是轴位。
   - 在MRI上，病变区域显示为高信号（T2加权），表明有液体或软组织成分。在T1加权可能低信号，但这里没有指定序列；从图像看，在左侧图中，有一个明亮的区域（可能T2高信号），表示水肿、肿瘤或炎症。
   - 病变似乎累及骨髓，并延伸到周围软组织。

**核心影像学特征总结：**

- **X线**：右肱骨近端见溶骨性病变，边界不清，可能有轻度膨胀。无明显骨膜反应（但需要确认）。
- **CT**：显示骨质破坏，低密度区，边缘不规则，可能有软组织肿块。
- **MRI**：T2加权高信号，提示病变内含液体或活跃的肿瘤过程；累及骨髓和周围软组织。

病史：女性47岁，无诱因疼痛5个月。这提示慢性过程，非急性外伤。

**可能疾病诊断（按可能性从大到小）：**

1. **原发性骨肿瘤**：
   - 骨肉瘤？但骨肉瘤更常见于青少年，年龄不符。
   - 软骨肉瘤：47岁女性，肱骨近端是软骨肉瘤的常见部位。软骨肉瘤常表现为溶骨性病变，MRI显示高信号（由于软骨基质）。
   - 骨巨细胞瘤：但骨巨细胞瘤多见于20-40岁，47岁稍大；通常发生在骨端，可能有膨胀。
2. **转移性肿瘤**：
   - 乳腺癌、肺癌等常见癌症的转移。患者47岁女性，无其他病史，但需要考虑。疼痛5个月，慢性。
   - 骨转移常表现为溶骨性病变（如乳腺癌），MRI显示高信号。
3. **良性肿瘤或病变**：
   - 动脉瘤样骨囊肿：但多见于青少年；可有膨胀、液体-液平面在MRI上。
   - 骨髓炎：但无感染史，X线可能更显示骨质破坏和反应性改变，这里似乎没有明显骨膜增生。

结合病史：

- 47岁女性，疼痛5个月，慢性。
- 影像学：溶骨性病变，累及骨髓和软组织，MRI高信号。

最可能诊断（基于常见疾病）：

1. **转移性肿瘤**：特别是乳腺癌、甲状腺癌等的骨转移。因为47岁女性，乳腺癌是常见原发灶；影像显示溶骨性破坏。
2. **软骨肉瘤**：原发骨肿瘤，肱骨近端好发于中老年人。
3. **多发性骨髓瘤**：但通常为多发性病变，这里只提右肱骨，可能单发；但骨髓瘤可有溶骨性破坏。

需要确认：

- 骨肉瘤在40+岁不常见，所以可能性低。
- 骨巨细胞瘤：年龄稍大（30-50岁），但47岁仍可能；通常边界较清晰，这里似乎模糊。

**最可能的3个诊断：**

1. **转移性骨肿瘤**（如乳腺癌转移）—— 最常见于中年女性。
2. **软骨肉瘤** —— 原发恶性肿瘤，好发部位。
3. **骨巨细胞瘤** —— 但年龄稍大；或考虑其他。

标准排序：

- 对于47岁女性，无诱因疼痛5个月，右肱骨近端病变：转移性肿瘤可能性最高（尤其乳腺癌），然后是软骨肉瘤，再是良性如动脉瘤样骨囊肿或其他。

**临床建议：**

- 下一步应进行全身检查以寻找原发灶。
  - 具体：全身影像学评估，如PET-CT或全身骨扫描。
  - 血液检查（如血钙、碱性磷酸酶）。
  - 活检：穿刺活检明确病理诊断。因为影像不能确诊。

**结构化回答：**

1. 影像学描述：客观描述核心特征。
2. 可能疾病：3个，按可能性排序。
3. 临床建议：具体步骤。

确保语言专业、准确。

**影像学研判报告**

**身份设定**：省级三甲医院骨肿瘤科会诊专家（基于X线、CT及MRI影像与极简病史分析）

**影像学描述**

- **X线特征**：右肱骨近端（肩关节下1/3处）可见不规则溶骨性破坏区，边界模糊不清，局部骨质呈“虫蚀样”缺损；无明显骨膜反应或Codman三角征；周围软组织未见显著肿胀。
- **CT特征**：横断面显示病灶为低密度区（CT值约20-40 HU），累及髓腔并突破皮质，边缘呈“地图样”不规则破坏；无明确骨硬化或钙化；相邻软组织内见低密度肿块影，提示软组织侵犯。
- **MRI特征**：T2加权序列病灶呈高信号（与周围肌肉对比明显），内部可见液-液平面及分叶状结构；T1加权序列呈低信号，增强扫描示病灶不均匀强化，累及骨髓腔并延伸至周围软组织。

**结合病史与影像的3个最可能疾病诊断（按可能性从大到小）**

1. **转移性骨肿瘤（乳腺癌骨转移最可能）**：47岁女性为乳腺癌高发人群；无诱因慢性疼痛5个月，符合转移灶进展特点；影像表现为溶骨性破坏、软组织侵犯及MRI高信号（提示肿瘤活性），与乳腺癌转移典型特征一致。
2. **软骨肉瘤（中度恶性）**：好发于40-60岁成人，肱骨近端为常见部位；X线/CT示边界不清的溶骨性病变、MRI显示软骨基质高信号及液-液平面，符合软骨肉瘤影像学表现。
3. **动脉瘤样骨囊肿（继发性）**：虽多见于青少年，但47岁女性中可发生；X线/CT示膨胀性溶骨破坏、MRI显示特征性液-液平面及高信号区，结合慢性疼痛病史，需考虑继发于其他肿瘤（如软骨肉瘤）的动脉瘤样变。

**临床建议**

1. **紧急病理确诊**：立即行右肱骨近端穿刺活检（CT引导下），获取组织标本进行病理学检查及免疫组化分析，明确良恶性及原发灶性质。
2. **全身评估**：
   - 行全身骨扫描（ECT）或PET-CT，排查多发性转移灶；
   - 实验室检查：血清肿瘤标志物（CEA、CA153）、生化全套（含碱性磷酸酶及钙离子），重点关注乳腺癌相关指标。
3. **多学科会诊**：若活检确诊为转移瘤，需联合乳腺外科/肿瘤内科制定治疗方案；若原发骨肿瘤，则转骨肿瘤外科行保肢手术或放化疗。

*注：本研判基于所提供影像及病史，最终诊断以病理结果为准。建议24小时内启动上述检查。*
