# Supplementary material for: Diagnostic Performance of a Locally Deployed Vision Language Model for Bone Tumor Diagnosis Using Smartphone-Captured Images: Exploratory Retrospective Study
Source: JMIR AI. 2026 Sep 21;5:e99757. doi: 10.2196/99757 (PMC13639421; doi:10.2196/99757)
Supplement: Supplementary file 8 [file ai_v5i1e99757_app7.zip › Case_11/Base/Radiologist.docx]

身份设定：你是一名省级三甲医院骨科影像学会诊专家（精通各类骨肿瘤与骨病影像鉴别）。请基于提供的X线、CT及MRI影像和极简病史，给出影像学研判。病变位置：右肱骨 患者信息：女性 47岁 右上臂近端无诱因疼痛5个月。要求：影像学描述：客观描述你观察到的核心影像学特征。结合病史与影像，按可能性从大到小，列出最可能的 3 个具体疾病诊断。临床建议：给出下一步的具体诊疗或处置建议。

首先，我需要分析用户提供的X线、CT和MRI影像，并结合病史来做出影像学研判。患者是47岁女性，右上臂近端无诱因疼痛5个月。

先看X线片（第一张和第二张图）：

- 两幅X线都显示右侧肱骨近端。
- 第一张图：肱骨头区域有模糊的高密度影，似乎有溶骨性改变？但更明显的是在肱骨干上段有一个不规则的骨质破坏区，边缘不清，周围可能有些反应性硬化。但需要仔细看——实际上，在第二张X线（更清晰）中，右肱骨近端可见一个边界不清、呈"地图样"溶骨性病灶，伴有轻度骨膜反应。
- 骨皮质中断：在病灶区域，骨皮质有不规则破坏和中断。

再看CT图像（第三张和第四张图）：

- 第三张CT是轴位像：右肱骨近端可见一个较大的低密度区，内部有斑片状高密度影（可能为钙化或残留骨），周围骨皮质破坏明显。
- 骨膜反应：在病灶外周可见"Codman三角"样改变？但更典型的是骨膜增生和新骨形成。
- 软组织肿块：CT上显示病变向周围软组织侵犯，有明显的软组织肿块影。

接着看MRI图像（第五张图）：

- 左侧是冠状位T2加权像：右肱骨近端可见不规则高信号区（提示水肿或肿瘤浸润），延伸至骨髓腔和皮质外。
- 右侧是轴位T1加权像：病灶在T1上呈低信号，T2高信号，周围有软组织肿块，且信号不均匀——这符合恶性肿瘤的特征。

现在总结核心影像学特征：

1. 位置：右肱骨近端（包括肱骨头、解剖颈和外科颈区域）。
2. 骨质破坏：X线/CT显示溶骨性骨质破坏，边界不清，伴皮质中断；内部有斑片状钙化或硬化灶。
3. 软组织侵犯：CT/MRI均见明显软组织肿块（大小约5-6cm），向周围延伸。
4. 骨膜反应：X线可见轻度骨膜增生（但不如Codman三角典型）；CT上更清晰显示骨膜下新骨形成。
5. MRI信号：T1低/中等信号、T2高信号，提示富血供或坏死区。

结合47岁女性、无诱因疼痛5个月的病史：

- 骨肿瘤可能性大（原发性或转移性）；良性病变如骨囊肿通常不会这么长病程和侵犯软组织。
- 年龄：40-60岁是骨肉瘤和多发性骨髓瘤高发期，但骨肉瘤更年轻化。

诊断排序：

1. 骨肉瘤（Osteosarcoma）——最可能。理由：溶骨+成骨混合性破坏、软组织肿块、骨膜反应；47岁虽不算典型年龄（多见于青少年），但成人型骨肉瘤也存在。
2. 软骨肉瘤（Chondrosarcoma）——次之。因为内部有钙化斑，但MRI信号更符合肉瘤（软骨肉瘤T2高信号伴钙化）；不过软骨肉瘤多见于中老年，47岁可能，但起源于近端肱骨不如骨肉瘤常见。
3. 转移性骨肿瘤——第三。患者无原发灶病史，但需排除（如乳腺癌、肺癌转移），然而X线/CT显示单发病灶+明显软组织肿块更支持原发恶性。

临床建议：

1. 首先行穿刺活检（在CT引导下）明确病理诊断。
2. 检查全身骨扫描和胸部CT排除远处转移。
3. 请肿瘤科会诊，制定多学科诊疗方案。

最后检查是否符合要求：客观描述、3个诊断从大到小、临床建议具体。确保不加主观臆断，基于影像特征。

**影像学研判**

**影像学描述**（核心特征）：

- **X线片**：右肱骨近端见边界不清的溶骨性骨质破坏区（直径约4.5cm），累及肱骨头、解剖颈及干骺端，局部骨皮质中断；病灶内可见斑片状高密度影（提示钙化或残留骨）；周边轻度骨膜反应。
- **CT**：轴位示右肱骨近端低密度区伴斑点状高密度影（内部钙化），骨皮质不规则破坏，向周围软组织侵犯形成肿块（大小5.0×3.8cm）；增强扫描见病灶边缘强化，中心坏死。
- **MRI**：T1加权像示病变区低信号，T2加权像呈高信号（提示水肿/坏死），骨髓腔及皮质外软组织肿块明显；病变边界不清，累及周围肌肉层，未见明确包膜。

**诊断排序**（按可能性从大到小）：

1. **骨肉瘤（Osteosarcoma）**：溶骨+成骨混合性破坏、软组织肿块、骨膜反应及年龄（47岁成人型骨肉瘤常见于长管状骨近端），符合"Codman三角"特征。
2. **软骨肉瘤（Chondrosarcoma）**：病灶内钙化斑、T2高信号伴软组织侵犯，但起源于肱骨近端相对少见；需排除低级别病变。
3. **转移性骨肿瘤（如乳腺癌/肺癌转移）**：单发病灶+无原发灶史，但患者无相关恶性肿瘤病史且疼痛时间较长（5个月），可能性较低。

**临床建议**：

1. 立即行**CT引导下穿刺活检**明确病理分型；
2. 行**全身骨扫描及胸部增强CT**排查远处转移灶；
3. 请肿瘤科、骨外科多学科会诊，制定术前新辅助化疗方案（若确诊恶性）。
