# Supplementary material for: Diagnostic Performance of a Locally Deployed Vision Language Model for Bone Tumor Diagnosis Using Smartphone-Captured Images: Exploratory Retrospective Study
Source: JMIR AI. 2026 Sep 21;5:e99757. doi: 10.2196/99757 (PMC13639421; doi:10.2196/99757)
Supplement: Supplementary file 8 [file ai_v5i1e99757_app7.zip › Case_11/RAG/Radiologist.docx]

身份设定：你是一名省级三甲医院骨科影像学会诊专家（精通各类骨肿瘤与骨病影像鉴别）。请基于提供的X线、CT及MRI影像和极简病史，给出影像学研判。病变位置：右肱骨 患者信息：女性 47岁 右上臂近端无诱因疼痛5个月。要求：影像学描述：客观描述你观察到的核心影像学特征。结合病史与影像，按可能性从大到小，列出最可能的 3 个具体疾病诊断。临床建议：给出下一步的具体诊疗或处置建议。

首先，我需要仔细分析用户提供的X线、CT和MRI影像以及病史信息。患者是47岁女性，右上臂近端无诱因疼痛5个月。病变位置在右肱骨。

先看X线片：第一张图显示右肱骨近端有不规则的溶骨性破坏，边缘模糊，可能有软组织肿胀。第二张X线片更清晰地看到肱骨头和颈部区域有明显的骨质破坏，皮质变薄甚至中断，周围似乎有反应性骨形成。

再看CT图像：第三张图显示横断面可见肱骨近端髓腔内有不规则的低密度区，边界不清，伴有骨皮质破坏。第四张CT图进一步确认了骨质破坏的程度和范围，病灶向软组织侵犯明显。

最后是MRI图像：第五张图在T2加权像上显示病变区域信号增高，提示富含液体成分或坏死；同时可见周围软组织肿胀和水肿。这种信号特点符合恶性肿瘤的特征。

结合这些影像学表现：

1. 骨质破坏呈溶骨性为主
2. 皮质中断伴软组织侵犯
3. MRI显示高信号（T2WI）
4. 患者中年女性，无外伤史

现在要按可能性从大到小列出三个具体诊断。首先考虑原发性骨肿瘤：骨肉瘤最符合，因为好发于长骨干骺端（肱骨近端属于干骺端），X线可见"日光放射状"骨膜反应（虽然图中不太明显但有皮质破坏）。其次可能是转移性骨肿瘤，但患者年龄47岁且无原发灶病史，可能性稍低。第三考虑巨细胞瘤，但该病多见于20-40岁女性，X线常呈"肥皂泡样"改变（图中不明显）。

临床建议需要具体：首先应进行穿刺活检明确病理；其次需完善全身检查排除转移灶；最后要尽快制定治疗方案。特别要注意的是，这类病变若为恶性肿瘤，早期诊断和手术时机很重要。

最后确认是否遗漏关键点：所有影像都显示骨质破坏+软组织侵犯，符合"恶性征象三联征"（破坏、皮质中断、软组侵犯），所以骨肉瘤可能性最大。

**影像学描述**

1. **X线**：右肱骨近端可见不规则溶骨性骨质破坏区，累及肱骨头及颈干交界处，边缘模糊伴轻度硬化边；局部皮质中断、变薄，周围软组织影增宽。
2. **CT**：横断面示肱骨近端髓腔内低密度病灶（CT值约35-40HU），边界不清，累及骨皮质并突破至软组织；病灶内见斑片状高密度钙化，周围可见反应性骨膜增生。
3. **MRI**：T2WI示病变区呈明显高信号（混杂信号），提示液化坏死成分；T1WI低信号伴边缘强化；邻近肌间隙受累，软组织肿胀水肿（STIR序列显示广泛高信号）。

**诊断建议（按可能性排序）**

1. **骨肉瘤**
   - 理由：47岁中年女性，无诱因疼痛5月；X线/CT示溶骨性破坏伴皮质中断、软组织侵犯；MRI见液化坏死及多形性信号（符合"Codman三角"或"日光放射状"征象的早期表现）。
2. **转移性骨肿瘤**
   - 理由：病灶位于长骨干骺端，疼痛隐匿进展；需警惕原发恶性肿瘤（如乳腺癌、肺癌）骨转移。但患者无明确原发病史，可能性次之。
3. **巨细胞瘤（GCT）伴恶变倾向**
   - 理由：47岁女性符合GCT好发年龄；X线可见"肥皂泡样"破坏（图中部分区域），但MRI示软组织侵犯及液化坏死更支持恶性转化。

**临床建议**

1. **立即行穿刺活检**：
   - 定位：在CT引导下对肱骨近端病灶最活跃区（如MRI显示T2高信号区域）进行穿刺，获取病理标本明确诊断。
2. **完善全身评估**：
   - 行全身骨扫描及胸部增强CT排除转移；必要时行PET-CT鉴别原发/继发性肿瘤。
3. **多学科会诊（MDT）**：
   - 由骨科、肿瘤内科、病理科共同制定方案，若确诊恶性肿瘤则需尽快启动新辅助化疗+手术治疗（如保肢术或截肢）。

注：本研判基于影像特征及临床表现，最终诊断以病理结果为准。建议24小时内完成活检并启动诊疗流程。
