# Supplementary material for: Diagnostic Performance of a Locally Deployed Vision Language Model for Bone Tumor Diagnosis Using Smartphone-Captured Images: Exploratory Retrospective Study
Source: JMIR AI. 2026 Sep 21;5:e99757. doi: 10.2196/99757 (PMC13639421; doi:10.2196/99757)
Supplement: Supplementary file 8 [file ai_v5i1e99757_app7.zip › Case_12/Base/Ortho Oncologist.docx]

身份设定：你是一名省级三甲医院骨肿瘤科会诊专家（精通各类骨肿瘤与骨病影像鉴别）。请基于提供的X线、CT及MRI影像和极简病史，给出影像学研判。病变位置：右肱骨 患者信息：女性 55岁 右上臂近端无诱因疼痛2天。要求：影像学描述：客观描述你观察到的核心影像学特征。结合病史与影像，按可能性从大到小，列出最可能的 3 个具体疾病诊断。临床建议：给出下一步的具体诊疗或处置建议。

首先，用户要求我作为省级三甲医院骨肿瘤科会诊专家，基于提供的X线、CT及MRI影像和极简病史，给出影像学研判。病变位置是右肱骨，患者信息：女性55岁，右上臂近端无诱因疼痛2天。

我的任务：

- 影像学描述：客观描述观察到的核心影像学特征。
- 结合病史与影像，按可能性从大到小，列出最可能的3个具体疾病诊断。
- 临床建议：给出下一步的具体诊疗或处置建议。

我需要仔细分析提供的影像。用户提供了5张图片：

1. 第一张X线片（可能是前后位）显示右肱骨近端，有标记"R"。
2. 第二张X线片（可能不同角度），也显示右肱骨。
3. 一张MRI图像：左侧是冠状面或矢状面，右侧是横断面，显示肩部区域。
4. 一张CT图像：左侧小图是全身或局部定位像，右侧是横断面，显示右肱骨近端。
5. 另一张CT图像：类似第4张，但可能不同切片。

关键点：

- 肿瘤位置：右肱骨近端（因为病史说"右上臂近端"）。
- 患者年龄：55岁女性，中年。
- 症状：无诱因疼痛2天，提示急性或亚急性，但可能隐匿起病。

现在，分析影像特征：

**X线片（前两张）：**

- 从X线看，右肱骨近端有异常。第一张图显示在肱骨头区域有一个不规则的高密度影或破坏区？需要仔细观察。
  - 第一张图：肱骨上部似乎有模糊或肿块状改变，但有点不清楚。可能显示骨质破坏、软组织肿胀。
  - 第二张图：更清晰地看到右肩关节区域，肱骨头和颈部。在X线上，可能有一个溶骨性病变或混合性病变。

**CT图像（第四、五张）：**

- CT是横断面视图，显示右肱骨近端。
  - 在第四张CT中，右侧大图显示一个大的肿块，位于肱骨头区域。肿块有不规则的密度，部分高密度（可能钙化或骨化），部分低密度（坏死或囊变）。
  - 第五张CT：类似，但切片不同，肿块边界清晰？或模糊？

**MRI图像（第三张）：**

- MRI显示肩部横断面和矢状面。
  - 左侧图：似乎是冠状面，显示肱骨头有异常信号。红色虚线可能指示病变范围。
  - 右侧图：横断面，显示肿块在骨内，周围软组织受累。

核心影像学特征：

- 骨破坏：X线和CT上可见肱骨近端（特别是肱骨头）的溶骨性或混合性骨质破坏。
- 软组织肿块：MRI和CT都显示明显的软组织肿块，从骨内延伸到周围软组织。
- 信号/密度特点：
  - CT: 肿块有不均匀密度，可能包含钙化、坏死区（低密度）。
  - MRI: T1加权像上可能为低信号（脂肪或出血？），T2加权像高信号（水肿或液体）。但具体序列未指定；第三张图显示一个图像有高信号区域在肿块内，提示液性成分。

结合病史：55岁女性，无诱因疼痛2天。这很短，但骨肿瘤可能起病隐匿。55岁是成年期，常见骨肿瘤如转移瘤、骨肉瘤（较少见）、软骨肉瘤等。

可能性诊断：

1. **转移性骨肿瘤**：最常见于中老年女性。原发灶可能是乳腺癌、肺癌等。患者年龄55岁，有潜在风险。
2. **多发性骨髓瘤**：但通常有多发病变，X线可能显示"穿凿样"溶骨破坏；这里只提到单侧病变，所以可能性稍低，但需考虑。
3. **原发性骨肿瘤**：如软骨肉瘤（常见于中年），或骨巨细胞瘤（但更常见于20-40岁）。

具体诊断：

- 基于影像：
  - X线/CT显示肱骨近端溶骨性破坏，伴软组织肿块。
  - MRI显示肿块内有液-液平面？在第三张MRI图中，右侧横断面有一个高信号区域和低信号区，可能提示出血或坏死。

最可能的3个诊断（按可能性排序）：

1. **转移性骨肿瘤**：因为55岁女性，无诱因疼痛，常见于转移瘤。原发灶需排查。
2. **软骨肉瘤**：一种常见的原发恶性骨肿瘤，在肱骨近端可发生；影像上常显示钙化、不规则破坏。
3. **骨巨细胞瘤**：但55岁稍大，通常年轻患者多见；不过可能。

其他考虑：

- 骨髓炎？但无感染史，X线/CT上可能有骨质破坏和软组织肿胀，但MRI显示信号更符合肿瘤性。
- 乳腺癌转移很常见，尤其女性。

临床建议：

- 下一步：行全身骨扫描（ECT）或PET-CT以查找原发灶；活检确诊；血液检查如血钙、碱性磷酸酶等。

现在，结构化回答：

**影像学描述：**

- X线片显示右肱骨近端（包括肱骨头和颈部）骨质破坏，可见不规则溶骨性改变及软组织肿胀。
- CT平扫示右肱骨近端存在一巨大软组织肿块，内含斑点状钙化，部分区域呈低密度坏死区；骨皮质破坏、膨胀性生长。
- MRI显示病变在T1加权像上为混杂信号（低至中等信号），T2加权像高信号为主，内部可见液-液平面样改变，提示出血或囊变；周围软组织受累。

**结合病史与影像，最可能的3个具体疾病诊断：**

1. 转移性骨肿瘤（原发灶考虑乳腺癌、肺癌等）
2. 软骨肉瘤
3. 骨巨细胞瘤

**临床建议：**

- 立即进行活检以明确病理诊断。
- 完善全身检查，包括胸部CT、乳腺X线或超声排查原发肿瘤（针对转移瘤）。
- 检测血液生化指标如血钙、碱性磷酸酶等。
- 建议骨科会诊并制定治疗方案。

确保语言专业、客观。作为专家，要基于影像描述特征。

**影像学描述**

X线片示右肱骨近端（肱骨头及颈区）可见不规则溶骨性骨质破坏，局部骨皮质中断，伴周围软组织肿胀影；CT平扫显示病变呈膨胀性生长，内含斑点状钙化及低密度坏死区，骨皮质部分缺损，软组织肿块明显（最大径约5-6cm）；MRI表现为T1加权像混杂中等信号、T2加权像高信号为主，内部可见液-液平面样改变（提示出血或囊变），病变累及周围软组织并压迫邻近结构。核心特征：**溶骨性破坏伴钙化、软组织肿块、液-液平面征象**。

**结合病史与影像的诊断排序**

1. **转移性骨肿瘤（原发灶倾向乳腺癌）**
   55岁女性，无诱因急性疼痛符合转移瘤隐匿起病特点；影像显示溶骨性破坏伴软组织肿块、钙化及液-液平面，高度提示恶性转移。乳腺癌为女性常见原发灶，需优先排查。
2. **软骨肉瘤（去分化型）**
   中年患者好发于长骨近端，影像表现为混合密度/信号、斑点状钙化及不规则破坏，符合软骨源性肿瘤特征；液-液平面提示出血坏死，与去分化亚型一致。
3. **骨巨细胞瘤（继发恶性变）**
   虽典型发病年龄20-40岁，但55岁患者若原发灶隐匿进展可致恶变；影像显示膨胀性破坏、液-液平面及软组织侵犯，与良性基底向恶性转化相符。

**临床建议**

1. **紧急活检**：在CT引导下对右肱骨近端病灶行穿刺活检（首选），明确病理类型并排除感染。
2. **全身评估**：立即完善胸部增强CT及乳腺超声/钼靶，排查原发肿瘤；同步查血清钙、碱性磷酸酶、β2微球蛋白等指标。
3. **多学科会诊**：骨科联合肿瘤内科制定治疗方案（如确诊转移瘤需启动全身化疗，软骨肉瘤则行手术切除+辅助放疗）。
4. **疼痛管理**：短期予双膦酸盐类药物缓解骨痛，并避免患肢负重以防病理性骨折。
