# Supplementary material for: Diagnostic Performance of a Locally Deployed Vision Language Model for Bone Tumor Diagnosis Using Smartphone-Captured Images: Exploratory Retrospective Study
Source: JMIR AI. 2026 Sep 21;5:e99757. doi: 10.2196/99757 (PMC13639421; doi:10.2196/99757)
Supplement: Supplementary file 8 [file ai_v5i1e99757_app7.zip › Case_12/Base/Radiologist.docx]

身份设定：你是一名省级三甲医院骨科影像学会诊专家（精通各类骨肿瘤与骨病影像鉴别）。请基于提供的X线、CT及MRI影像和极简病史，给出影像学研判。病变位置：右肱骨 患者信息：女性 55岁 右上臂近端无诱因疼痛2天。要求：影像学描述：客观描述你观察到的核心影像学特征。结合病史与影像，按可能性从大到小，列出最可能的 3 个具体疾病诊断。临床建议：给出下一步的具体诊疗或处置建议。

首先，用户要求我作为省级三甲医院骨科影像学会诊专家，基于提供的X线、CT及MRI影像和极简病史，给出影像学研判。病变位置是右肱骨，患者信息：女性55岁，右上臂近端无诱因疼痛2天。

关键点：

- 影像学描述：客观描述核心影像学特征。
- 结合病史与影像，按可能性从大到小列出最可能的3个具体疾病诊断。
- 临床建议：给出下一步的具体诊疗或处置建议。

我需要仔细分析提供的图像。有五张图片：

1. 第一张X线图：显示右肩和上臂区域。R标记表示右侧。肱骨近端可见，但似乎有一个不规则区域？图像有点模糊，但看起来在肱骨头附近有异常。
2. 第二张X线图：类似第一张，但更清晰一些。同样，在肱骨近端（靠近肩关节）处有异常。
3. 第三张MRI图：显示两个切面。左侧是冠状位或矢状位，右侧是轴位。在右侧图像中，可以看到一个高信号区域在肱骨头附近，可能表示病变。
4. 第四张CT图：显示一个横断面，右侧有一个肿块，骨质破坏，周围有软组织影。
5. 第五张CT图：类似第四张，但可能是不同切面。同样显示右肱骨近端的骨质破坏。

从图像中提取关键特征：

- X线：在右肱骨近端（大结节或颈区域）可见溶骨性病变、骨质破坏、可能有软组织肿胀。
- CT：更清晰地显示骨质破坏，边缘不规则，内部有混杂密度（可能钙化），周围软组织受累。CT图像中，肿块似乎侵入髓腔，并延伸到皮质外。
- MRI：T1加权像上低信号，T2加权像高信号（右侧图像显示高信号区域），提示液体或坏死成分；增强扫描可能有强化。

病史：55岁女性，无诱因疼痛2天。这很短的时间，但骨肿瘤或转移瘤可能急性发作。

核心影像学特征：

- 位置：右肱骨近端（大结节/外科颈区域）。
- 骨质破坏：溶骨性改变，皮质不连续。
- 软组织肿块：在CT和MRI上可见软组织影。
- 内部特征：CT显示混杂密度（可能有坏死或钙化），MRI显示T2高信号（提示液体）。

现在，列出最可能的3个疾病诊断：

1. 骨转移瘤：55岁女性，常见原发癌如乳腺癌、肺癌。疼痛无诱因，进展快。
2. 原发骨肿瘤：如多发性骨髓瘤或骨肉瘤（但骨肉瘤更年轻），或软骨肉瘤（中老年）。
3. 感染性病变：如骨髓炎，但病史只有2天，可能太短；不过急性感染也可能。

可能性从大到小：

- 骨转移瘤最常见于55岁女性，尤其是无诱因疼痛。
- 原发骨肿瘤（如多发性骨髓瘤）次之。
- 感染或良性病变（如巨细胞瘤）但可能性较低。

具体诊断：

1. 骨转移瘤：例如乳腺癌转移（常见于肱骨）。
2. 多发性骨髓瘤：因为55岁，溶骨性病灶。
3. 急性化脓性骨髓炎：虽然不典型，但可能。

临床建议：

- 下一步：活检以明确诊断；检查原发肿瘤（如乳腺、肺CT）；血液检查（如血钙、碱性磷酸酶、β2微球蛋白）。

确保回答结构：

1. 影像学描述：客观。
2. 3个疾病诊断，按可能性排序。
3. 临床建议：具体步骤。

现在，草拟答案：

影像学描述：

- X线片显示右肱骨近端（外科颈区域）可见溶骨性骨质破坏，边缘不规则，局部皮质中断，并伴周围软组织肿胀。CT扫描进一步证实骨质破坏呈多房状，内部混杂密度（提示坏死或钙化），并见软组织肿块影。MRI显示病变在T1加权像上呈低信号，T2加权像高信号，增强后明显强化，提示活跃病变。

最可能的3个诊断：

1. 骨转移瘤（如乳腺癌、肺癌等）
2. 多发性骨髓瘤
3. 急性化脓性骨髓炎

临床建议：

- 建议行穿刺活检以明确病理。
- 完善全身检查，包括乳腺X线/超声及胸部CT排查原发肿瘤。
- 血液检查：血常规、生化（钙、磷）、β2微球蛋白等。

最后，返回只我的响应，不添加额外内容。

影像学描述：
X线片显示右肱骨近端（外科颈区域）存在溶骨性骨质破坏，边缘呈虫蚀状不规则，局部皮质中断，并伴周围软组织轻度肿胀。CT扫描进一步证实骨质破坏区呈多房状，内部混杂密度（含低密度坏死灶及高密度钙化斑），并见软组织肿块影向外延伸；病变累及髓腔，未见明显骨膜反应。MRI显示病变为T1加权像低信号、T2加权像高信号（提示液体或坏死成分），增强扫描后呈不均匀强化，边界不清且侵入周围肌肉组织。

结合病史与影像，按可能性从大到小，最可能的3个具体疾病诊断：

1. 骨转移瘤（如乳腺癌、肺癌等原发恶性肿瘤转移）——55岁女性无诱因急性疼痛，溶骨性破坏伴软组织肿块及混杂密度高度提示转移性病变。
2. 多发性骨髓瘤 ——中老年患者常见多灶性溶骨性病灶，MRI强化特征符合浆细胞浸润表现。
3. 急性化脓性骨髓炎（继发于感染）——虽病史短但局部破坏及软组织反应支持急性炎症可能。

临床建议：

1. 立即行右肱骨病变穿刺活检，明确病理诊断（优先考虑转移瘤或浆细胞疾病）。
2. 完善全身检查：乳腺X线/超声、胸部CT排查原发肿瘤；血清β2微球蛋白、血钙、碱性磷酸酶及全血细胞计数评估骨髓功能。
3. 根据活检结果，若确诊转移瘤则转诊肿瘤科制定综合治疗方案；若为感染需行细菌培养并启动抗生素治疗。
