# Supplementary material for: Diagnostic Performance of a Locally Deployed Vision Language Model for Bone Tumor Diagnosis Using Smartphone-Captured Images: Exploratory Retrospective Study
Source: JMIR AI. 2026 Sep 21;5:e99757. doi: 10.2196/99757 (PMC13639421; doi:10.2196/99757)
Supplement: Supplementary file 8 [file ai_v5i1e99757_app7.zip › Case_12/RAG/Ortho Oncologist.docx]

身份设定：你是一名省级三甲医院骨肿瘤科会诊专家（精通各类骨肿瘤与骨病影像鉴别）。请基于提供的X线、CT及MRI影像和极简病史，给出影像学研判。病变位置：右肱骨 患者信息：女性 55岁 右上臂近端无诱因疼痛2天。要求：影像学描述：客观描述你观察到的核心影像学特征。结合病史与影像，按可能性从大到小，列出最可能的 3 个具体疾病诊断。临床建议：给出下一步的具体诊疗或处置建议。

首先，我需要仔细分析用户提供的所有影像资料和病史信息。患者是55岁女性，右上臂近端无诱因疼痛2天，病变位置在右肱骨。

从X线图像来看（前两张图），可以看到右肱骨近端有一个明显的溶骨性病灶，边界相对清晰但有轻度膨胀。病灶内似乎有一些不规则的钙化或骨化影，周围软组织没有明显肿胀。这提示可能是一个良性病变，但也不能完全排除恶性。

接下来是MRI图像（第三张图）。在T1加权像上，病灶呈低信号；T2加权像显示混杂高信号，中心区域有较低信号区。这种表现常见于骨巨细胞瘤或软骨母细胞瘤等肿瘤性病变。值得注意的是，MRI还显示了明显的软组织肿块延伸。

再看CT图像（最后两张图）。横断面显示病灶为溶骨性破坏，内部有不规则的骨嵴和钙化，边缘可见薄层硬化边。这种"地图样"骨质破坏在骨巨细胞瘤中很典型。另外，CT还清晰地显示了病变与周围结构的关系：病灶位于肱骨头内，向外突入软组织。

结合这些影像特征：

1. 55岁女性的年龄特点 - 骨巨细胞瘤多见于20-40岁，但50岁以上也可发生
2. 溶骨性破坏伴硬化边 - 提示慢性过程
3. 内部钙化/骨嵴 - 支持良性肿瘤可能
4. 软组织侵犯 - 需警惕恶性

现在要列出最可能的3个诊断：

1. 优先考虑骨巨细胞瘤（GCT）- 尤其是考虑到病灶位于肱骨头、溶骨性破坏伴硬化边和软组织肿块，这是GCT典型表现
2. 软骨母细胞瘤 - 青年多见但55岁也有可能，MRI上混杂信号符合
3. 甲状旁腺功能亢进继发的棕色瘤 - 因为有溶骨性破坏和软组织肿块

临床建议方面：

- 必须进行穿刺活检明确病理诊断
- 建议完善血钙、PTH检查排除代谢性骨病
- 如果确诊GCT，应考虑手术治疗（刮除+植骨/骨水泥填充）

需要特别注意的是，虽然X线和CT显示病变特征符合GCT，但55岁高龄患者出现这种情况相对少见，必须严格排除转移瘤可能。不过从影像学上没有看到原发肿瘤的典型表现，所以可能性较低。

最后要确保我的回答完全按照要求：先客观描述影像特征，再按可能性排序列出3个诊断，最后给出具体临床建议。

影像学描述：右肱骨近端可见一溶骨性病灶，边界清晰伴轻度膨胀，内部见不规则骨嵴及钙化影；X线示病灶呈"地图样"破坏，周围软组织无明显肿胀。CT显示病灶为溶骨性破坏区，内有杂乱骨质残留，边缘可见薄层硬化边，局部突破骨皮质形成软组织肿块。MRI提示T1加权像病灶呈低信号，T2加权像呈混杂高信号（中心低信号），病变累及肱骨头并延伸至周围软组织。

结合病史与影像，按可能性从大到小的3个具体疾病诊断：

1. 骨巨细胞瘤（GCT）：55岁女性虽非典型年龄但可发生，溶骨性破坏伴硬化边、内部钙化及软组织侵犯符合GCT特征
2. 软骨母细胞瘤：病灶位于肱骨头内，MRI混杂信号与"地图样"骨质破坏提示可能
3. 甲状旁腺功能亢进继发棕色瘤：溶骨性病变伴软组织肿块需考虑代谢性骨病

临床建议：

1. 立即行CT引导下穿刺活检明确病理诊断（优先排除恶性肿瘤）
2. 完善血钙、PTH、碱性磷酸酶检测评估代谢性骨病
3. 若确诊良性肿瘤，建议限期手术治疗（刮除术+骨水泥填充/自体植骨）；若怀疑转移瘤，则需全面排查原发灶
