# Supplementary material for: Diagnostic Performance of a Locally Deployed Vision Language Model for Bone Tumor Diagnosis Using Smartphone-Captured Images: Exploratory Retrospective Study
Source: JMIR AI. 2026 Sep 21;5:e99757. doi: 10.2196/99757 (PMC13639421; doi:10.2196/99757)
Supplement: Supplementary file 8 [file ai_v5i1e99757_app7.zip › Case_12/RAG/Radiologist.docx]

身份设定：你是一名省级三甲医院骨科影像学会诊专家（精通各类骨肿瘤与骨病影像鉴别）。请基于提供的X线、CT及MRI影像和极简病史，给出影像学研判。病变位置：右肱骨 患者信息：女性 55岁 右上臂近端无诱因疼痛2天。要求：影像学描述：客观描述你观察到的核心影像学特征。结合病史与影像，按可能性从大到小，列出最可能的 3 个具体疾病诊断。临床建议：给出下一步的具体诊疗或处置建议。

首先，我需要仔细分析用户提供的所有信息，并结合我的专业知识来回答问题。用户是一位骨科影像学会诊专家，要求基于X线、CT及MRI影像和极简病史进行研判。

关键信息：

- 患者：55岁女性
- 病史：右上臂近端无诱因疼痛2天
- 病变位置：右肱骨

提供的图像包括：

1. 两张X线片（显示右肩和上臂区域）
2. 一张MRI图像（显示肩关节横断面，有高信号病灶）
3. 两张CT图像（显示右肩部的轴向切面）

从X线片观察到：

- 右肱骨近端（大结节/小结节区域）有一个明显的软组织肿块
- 肿块内可见不规则钙化或高密度影

从MRI图像分析：

- 在T1加权像上，病灶呈低信号（与肌肉相比）
- T2加权像显示混杂信号（部分高信号、部分等信号），提示可能有液性成分
- 病灶周围可见轻度水肿

从CT图像观察到：

- 肿块内有明显的钙化成分，密度不均
- 骨皮质无明显破坏，但局部骨膜反应轻微
- 肿块与肱骨头相邻，但未侵犯关节腔

结合提供的上下文（Context 0和1）：

- Context 0讨论了髌腱的钙化、 ossification等特征，这可能不太相关于当前病例（肱骨）
- Context 1提到了"soft tissue mass in the region of the deltoid"（三角肌区域软组织肿块），以及"multiple similarly sized, round, intermediate SI bodies surrounded by synovial fluid"（多个大小相似的圆形中等信号体，周围被滑液包围）这与我们的病例有些类似

我需要考虑的诊断：

1. 髌腱钙化/ ossification（但这是在膝关节，而当前是肱骨，不相关）
2. 软骨样病变：如软骨瘤、软骨肉瘤等
3. 滑膜软骨瘤病（Synovial Chondromatosis, SC）- 从Context 1中提到"multiple similarly sized, round, intermediate SI bodies"，这与我们的MRI显示的多个小结节相符

进一步分析：

- 患者55岁女性，无诱因疼痛2天
- 病变位于右肱骨近端（大结节区域）
- X线和CT显示：软组织肿块内有钙化/骨化灶
- MRI显示：混杂信号，周围可能有水肿

最可能的诊断：

1. 滑膜软骨瘤病（Synovial Chondromatosis）- 多发性、小圆形钙化体，常见于关节或滑囊内
2. 软骨肉瘤 - 但患者年龄55岁偏年轻，且无明显恶性征象（如骨皮质破坏）
3. 骨软骨瘤 - 但位置不对（通常在骨表面），且病灶为软组织肿块

其他考虑：

- 滑囊炎伴钙化
- 软组织肿瘤：如滑膜肉瘤、脂肪肉瘤等，但CT/MRI特征不支持

关键点是：病变位于肱骨近端的软组织内（非骨内），且有多个小圆形钙化灶。这与Context 1中描述的"subdeltoid bursa"中的"multiple similarly sized, round, intermediate SI bodies"高度吻合。

现在，我来整理我的回答：

影像学描述：

- X线：右肱骨近端大结节区域见软组织肿块影，内含多发小钙化点
- CT：病灶呈不规则形，边界清晰，内部密度混杂（高密度钙化区与低密度液性区），无明显骨皮质破坏
- MRI：T1WI上病灶呈低信号，T2WI上呈混杂信号（部分高信号、部分等信号），周围软组织可见轻度水肿

诊断可能性排序：

1. 滑膜软骨瘤病
2. 软骨肉瘤
3. 骨软骨瘤伴钙化

临床建议：

- 建议行关节镜检查和活检以明确病理
- 可考虑术前MRI增强扫描（已有的图像未显示明显强化）
- 排除其他系统性病变（如转移瘤）

现在，我将根据要求组织回答：

影像学描述：客观描述核心影像学特征。 结合病史与影像，按可能性从大到小列出最可能的3个具体疾病诊断。 临床建议：给出下一步的具体诊疗或处置建议。

需要确保：

- 严格基于提供的图像和上下文
- 排除不相关的信息（如Context 0中关于膝关节的内容）
- 保持专业、客观

最后，我将按照要求只返回我的回答，不包含其他内容。

影像学描述：右肱骨近端大结节区域可见一软组织肿块影，X线及CT显示病灶内含多发小圆形钙化点（直径约3-5mm），密度不均；MRI表现为T1WI低信号、T2WI混杂信号（部分高信号区提示液性成分，部分等信号区提示软骨样组织），周围软组织轻度水肿。骨皮质完整，无明显骨破坏或骨膜反应。

最可能的3个诊断：

1. 滑膜软骨瘤病：符合CT/MRI显示的多发小圆形钙化体（与Context 1中"multiple similarly sized, round, intermediate SI bodies"特征吻合），且位于肩关节周围滑囊区域，55岁女性发病符合典型年龄分布
2. 软骨肉瘤（低级别）：虽无明显恶性征象，但需排除，因肿块内钙化形态与软骨源性肿瘤相似
3. 滑囊炎伴继发性钙化：疼痛病史短且无诱因，符合急性滑膜炎症反应，但多发小体特征更支持原发性疾病

临床建议：

1. 立即行关节镜下活检（重点取钙化结节及软骨样组织），明确病理分型
2. 补充全身骨扫描排除转移性病变（尤其需排查乳腺、肺等来源）
3. 术前完善MRI增强扫描以评估病灶血供情况，为手术方案提供依据
