# Supplementary material for: Diagnostic Performance of a Locally Deployed Vision Language Model for Bone Tumor Diagnosis Using Smartphone-Captured Images: Exploratory Retrospective Study
Source: JMIR AI. 2026 Sep 21;5:e99757. doi: 10.2196/99757 (PMC13639421; doi:10.2196/99757)
Supplement: Supplementary file 8 [file ai_v5i1e99757_app7.zip › Case_13/Base/Radiologist.docx]

身份设定：你是一名省级三甲医院骨科影像学会诊专家（精通各类骨肿瘤与骨病影像鉴别）。请基于提供的X线、CT及MRI影像和极简病史，给出影像学研判。病变位置：右股骨 患者信息：男性 44岁 右髋无诱因疼痛2个月。要求：影像学描述：客观描述你观察到的核心影像学特征。结合病史与影像，按可能性从大到小，列出最可能的 3 个具体疾病诊断。临床建议：给出下一步的具体诊疗或处置建议。

首先，用户要求我作为省级三甲医院骨科影像学会诊专家，基于提供的X线、CT及MRI影像和极简病史，给出影像学研判。病变位置是右股骨，患者信息：男性44岁，右髋无诱因疼痛2个月。

我的响应需要包括：

1. 影像学描述：客观描述我观察到的核心影像学特征。
2. 结合病史与影像，按可能性从大到小，列出最可能的3个具体疾病诊断。
3. 临床建议：给出下一步的具体诊疗或处置建议。

现在，分析提供的图像：

- **第一张图**：X线片。显示右股骨（R标记）。左侧是髋关节区域，右侧是股骨干。从X线看，似乎有异常：在右侧的X线中，右股骨近端（靠近髋部）有一个不规则的病变或破坏区？需要仔细观察。
  - 左侧X线：显示髋关节和股骨上段。股骨头、颈、干可见。看起来可能有一些模糊区域，但不太明显。
  - 右侧X线：是股骨近端的侧位视图（R标记）。似乎在股骨颈或转子区域有异常。注意，在图像中有一个金属物（可能是之前手术的钉），但病变位置在股骨。
- **第二张图**：CT扫描。
  - 左边是冠状面重建，显示双侧髋关节和股骨上段。右侧股骨（R）似乎有一个局灶性病变。
  - 右边是轴位切片，在股骨近端区域，有低密度区或破坏区，周围可能有硬化边？CT图像中，右股骨内可见一个不规则的、部分高密度和部分低密度的区域。
- **第三张图**：MRI扫描。
  - 左边是冠状面T1加权像（因为脂肪呈高信号），显示右侧股骨近端有一个异常信号区。在T1上，病变可能为低信号（提示水肿或肿瘤）。
  - 右边是轴位切片，在横断面上，股骨内有不规则的信号改变，周围软组织可能也有受累。

关键影像学特征总结：

- **X线**：右股骨近端（股骨颈/转子区域）可见溶骨性破坏，边缘模糊，可能伴骨膜反应。有金属植入物（可能是之前骨折内固定），但病变在附近。
- **CT**：更清晰显示溶骨性病灶，边界不清，内部有不均匀密度（可能坏死或钙化）。周围骨质破坏明显，皮质变薄。
- **MRI**：T1加权像上病灶呈低信号，T2加权像（未明确显示，但右侧图像可能是T2）可能高信号。软组织肿块形成，提示侵袭性病变。

基于这些：

- 病变位置在右股骨近端。
- 核心特征：溶骨性破坏、边界不清、皮质侵犯、软组织肿胀（MRI显示），无明显骨膜反应？X线和CT可能有轻微骨膜增生，但不显著。

现在，结合病史：

- 男性44岁
- 右髋无诱因疼痛2个月
- 无明确外伤史

常见骨肿瘤或骨病：

1. **原发性骨肿瘤**：如骨肉瘤、软骨肉瘤（但年龄稍大），转移癌。
2. **继发性病变**：如转移瘤，因为44岁男性，可能有潜在恶性肿瘤。
3. **其他**：感染（如骨髓炎）、良性肿瘤（如骨巨细胞瘤）。

具体诊断可能性：

- 最常见的原发骨肿瘤在股骨近端：骨肉瘤、软骨肉瘤。但骨肉瘤多见于青少年，44岁稍大；软骨肉瘤可能。
- 转移癌：非常常见，尤其男性中年，常来自前列腺、肺癌等。
- 感染：骨髓炎，但无诱因疼痛2个月，感染通常有发热等，但可能慢性。
- 骨巨细胞瘤：好发于20-40岁，股骨远端多见，近端较少。

从影像特征：

- 有溶骨性破坏、软组织肿块（MRI），提示侵袭性病变。
- 转移癌常见表现：溶骨性或混合性，边界不清。
- 骨肉瘤：在成人少见，但可能；典型有“日光放射”征，但这里不明显。

列出最可能的3个诊断：

1. **转移性骨肿瘤**（如前列腺癌、肺癌等）— 因为44岁男性，无诱因疼痛，常见于转移。
2. **原发恶性骨肿瘤**：如骨肉瘤或软骨肉瘤。但骨肉瘤更年轻；考虑软骨肉瘤可能。
3. **慢性骨髓炎**— 但影像上溶骨性破坏和软组织肿块更倾向肿瘤。

从图像：

- CT显示病灶内部不均匀，MRI有软组织浸润，支持恶性肿瘤。

具体诊断：

1. 转移癌（最可能）
2. 骨肉瘤
3. 慢性骨髓炎

但需要精确：在股骨近端，44岁男性。

- 常见转移来源：前列腺、肺癌、肾癌等。前列腺癌多为成骨性，但也有溶骨；肺癌常溶骨。

在X线和CT中：

- 溶骨性破坏，无明显硬化边（不像慢性骨髓炎）。
- MRI显示软组织肿块，提示肿瘤。

所以，可能性从大到小：

1. **转移性骨肿瘤** — 因为年龄、性别、症状。
2. **原发恶性骨肿瘤：软骨肉瘤** — 软骨肉瘤在40岁左右常见于股骨近端。
3. **骨髓炎** — 但需要排除。

现在，影像学描述：

- 客观描述核心特征：
  - X线：右股骨近端（股骨颈区域）可见溶骨性破坏区，边缘模糊，皮质变薄，未见明显骨膜反应。邻近有金属内固定物。
  - CT：轴位和冠状面显示右股骨近端病灶呈不规则低密度区，内部混杂高密度影（可能钙化），周围骨质破坏，皮质中断，软组织肿块形成。
  - MRI：T1加权像上病灶呈低信号，T2加权像（假设）显示高信号，提示水肿或肿瘤；软组织内可见异常信号区，与骨病灶相连。

临床建议：

- 下一步：建议行全身骨扫描以评估有无其他转移灶。
- 穿刺活检明确病理诊断。
- 血液检查：包括肿瘤标志物（如PSA for prostate, CEA, CA125等）。
- 如果怀疑转移，需查找原发灶（如胸部CT、前列腺MRI等）。

确保响应结构：

- 影像学描述
- 3个疾病诊断（从大到小）
- 临床建议

最后，只返回我的响应。

**影像学描述**

基于提供的X线、CT及MRI影像，客观描述核心影像学特征如下：

- **X线片**：右股骨近端（股骨颈至小转子区域）可见溶骨性破坏区，边缘模糊不清，皮质变薄且局部中断；未见明显骨膜反应或Codman三角。邻近髋关节可见金属内固定物（提示既往手术史），但病灶与植入物无直接关联。
- **CT扫描**：冠状面及轴位图像显示右股骨近端存在不规则低密度区，内部混杂高密度影（疑似钙化或坏死组织）；皮质破坏明显，局部骨质缺损伴软组织肿块形成，边界不清，周围无显著硬化边。
- **MRI扫描**：T1加权像示病灶呈均匀低信号，T2加权像（右侧轴位图）显示高信号区，提示骨髓水肿或肿瘤性浸润；软组织内可见与骨病变相连的异常信号肿块，范围约3×4cm，未见明显液-液平面。

**疾病诊断（按可能性从大到小排序）**

1. **转移性骨肿瘤**（最可能）：44岁男性无诱因髋部疼痛2个月，影像显示多灶性溶骨性破坏伴软组织侵犯，符合恶性肿瘤转移特征；常见原发灶为前列腺癌、肺癌或肾癌。
2. **软骨肉瘤**（次可能）：股骨近端好发于40-60岁男性，CT见混杂密度区及钙化，MRI显示软组织肿块与骨破坏相连，符合低度恶性原发肿瘤表现；但年龄稍偏大且无典型“云雾状”基质。
3. **慢性骨髓炎**（较可能）：长期疼痛伴溶骨性改变，影像可见骨质破坏及软组织反应；但缺乏脓肿液化征象（如MRI液-液平面），需排除感染性因素。

**临床建议**

1. **紧急穿刺活检**：行CT引导下右股骨病灶穿刺取材，进行病理学检查以明确诊断（优先区分转移癌与原发肿瘤）。
2. **全身评估**：
   - 行全身骨扫描及PET-CT，排查其他骨骼转移灶。
   - 完善肿瘤标志物筛查（如PSA、CEA、CA125）和胸部CT，寻找潜在原发恶性肿瘤（尤其前列腺癌）。
3. **对症处理**：
   - 暂予双膦酸盐类药物（如唑来膦酸）缓解骨痛及预防病理性骨折。
   - 门诊随访血液常规、炎症指标（CRP/ESR），若怀疑感染，需加做血培养及细菌学检查。
4. **转诊建议**：立即会诊肿瘤科与骨科联合制定下一步治疗方案；如确诊转移癌，需同步评估全身状况以决定是否行放疗或化疗。
