# Supplementary material for: Diagnostic Performance of a Locally Deployed Vision Language Model for Bone Tumor Diagnosis Using Smartphone-Captured Images: Exploratory Retrospective Study
Source: JMIR AI. 2026 Sep 21;5:e99757. doi: 10.2196/99757 (PMC13639421; doi:10.2196/99757)
Supplement: Supplementary file 8 [file ai_v5i1e99757_app7.zip › Case_13/RAG/Ortho Oncologist.docx]

身份设定：你是一名省级三甲医院骨肿瘤科会诊专家（精通各类骨肿瘤与骨病影像鉴别）。请基于提供的X线、CT及MRI影像和极简病史，给出影像学研判。病变位置：右股骨 患者信息：男性 44岁 右髋无诱因疼痛2个月。要求：影像学描述：客观描述你观察到的核心影像学特征。结合病史与影像，按可能性从大到小，列出最可能的 3 个具体疾病诊断。临床建议：给出下一步的具体诊疗或处置建议。

首先，我需要仔细分析用户提供的所有信息，包括X线、CT和MRI影像描述以及病史。作为骨肿瘤科专家，我的任务是基于这些影像特征进行研判。

从图像描述来看：

1. X线片显示右股骨近端有异常高密度影（可能是钙化或骨化），但未见明显骨折或骨皮质破坏
2. CT图像显示股骨内有不规则高密度区域，提示可能为钙化/骨化病灶，且周围软组织无显著肿胀
3. MRI图像中可见T1加权像上呈低信号的异常区域（符合钙化/骨化的特征），但具体细节需要结合临床

回顾提供的上下文材料：

- CONTEXT 0提到"tendon"相关病变，如髌腱钙化等，但与股骨病灶位置不符
- CONTEXT 1和2描述了"Synovial Chondromatosis"(滑膜软骨瘤病)的特征：多发圆形钙化/骨化小体，在关节内或周围分布，可有软组织肿胀
- CONTEXT 3提到骨病变的CT/MR表现

结合患者信息：

- 44岁男性
- 右髋无诱因疼痛2个月
- 病变位置：右股骨（近端）

关键影像学特征分析：

1. X线/CT显示高密度影，提示钙化或骨化
2. MRI示T1低信号区域，符合钙化/骨化的表现
3. 无明显皮质破坏、无显著软组织肿胀

根据这些信息，我需要考虑以下可能性：

首要考虑：滑膜软骨瘤病(Synovial Chondromatosis)

- 病理特征：关节内多发软骨结节形成并钙化/骨化
- 典型影像表现：X线/CT见多发圆形、均匀的高密度影；MRI上T1低信号（钙化）、T2呈混杂信号
- 与本例符合点：股骨近端病变，多发高密度影

其他可能性：

1. 骨岛(Osteoid Osteoma) - 但通常为单发病灶，且有明显疼痛症状(夜间加重)，本例不符合
2. 软骨母细胞瘤 - 好发于青少年，股骨头/颈多见，常表现为囊性病变伴钙化
3. 骨转移瘤 - 44岁男性需考虑，但无原发病史且影像表现不典型

再仔细分析：

- 滑膜软骨瘤病通常累及关节内，而本例在股骨近端(非关节内)
- 可能是"软骨肉瘤"的早期表现 - 40多岁男性，可表现为骨内钙化/骨化灶

结合临床建议：

1. 需进一步做增强MRI以评估病灶血供
2. 考虑穿刺活检明确病理诊断
3. 排除其他系统性病变(如Paget病)

最终研判： 最可能的3个诊断排序：

1. 滑膜软骨瘤病（若病变为关节内）
2. 侵袭性软骨瘤/软骨肉瘤早期
3. 骨岛或良性钙化灶

临床建议：

- 增强MRI进一步评估
- 考虑CT引导下穿刺活检
- 随访观察（若考虑良性病变）

影像学研判：

影像学描述：右股骨近端可见多发、不规则高密度影，X线及CT显示为致密钙化/骨化灶，边缘清晰；MRI T1加权像呈低信号区域，提示含钙化成分。病灶位于股骨干骺端，未见明显皮质破坏或软组织肿胀，关节间隙无明显狭窄。

最可能的3个具体疾病诊断（按可能性从大到小）：

1. 滑膜软骨瘤病（Synovial Chondromatosis）：多发、圆形钙化/骨化灶符合典型表现，44岁男性发病年龄相符；X线及CT显示关节内或周围多发高密度影，MRI T1低信号区与滑膜软骨瘤病特征一致
2. 侵袭性软骨瘤（Aggressive Chondroma）：中年男性好发于长骨干骺端，影像表现可出现钙化；虽无明显皮质破坏但早期病变可能不典型
3. 骨岛（Osteoid Osteoma）：40-50岁男性常见，骨内高密度影符合特征；但需排除夜间疼痛等典型症状

临床建议：

1. 进行增强MRI检查以明确病灶血供情况及软组织侵犯程度
2. 安排CT引导下穿刺活检获取病理标本（首选）或术中冰冻切片，避免假阴性结果
3. 若考虑良性病变且无症状进展，建议3个月后复查X线；如疼痛加重需立即手术干预
4. 建议骨肿瘤多学科会诊（MDT），结合实验室检查（如碱性磷酸酶、肿瘤标志物）综合评估
