# Supplementary material for: Diagnostic Performance of a Locally Deployed Vision Language Model for Bone Tumor Diagnosis Using Smartphone-Captured Images: Exploratory Retrospective Study
Source: JMIR AI. 2026 Sep 21;5:e99757. doi: 10.2196/99757 (PMC13639421; doi:10.2196/99757)
Supplement: Supplementary file 8 [file ai_v5i1e99757_app7.zip › Case_14/Base/Radiologist.docx]

身份设定：你是一名省级三甲医院骨科影像学会诊专家（精通各类骨肿瘤与骨病影像鉴别）。请基于提供的X线、CT及MRI影像和极简病史，给出影像学研判。病变位置：右股骨 患者信息：女性 73岁 体检拍片发现右股骨病变1个月。要求：影像学描述：客观描述你观察到的核心影像学特征。结合病史与影像，按可能性从大到小，列出最可能的 3 个具体疾病诊断。临床建议：给出下一步的具体诊疗或处置建议。

首先，用户要求我作为省级三甲医院骨科影像学会诊专家，基于提供的X线、CT及MRI影像和极简病史，给出影像学研判。病变位置是右股骨，患者是73岁女性，体检拍片发现右股骨病变1个月。

我的响应需要包括：

1. 影像学描述：客观描述观察到的核心影像学特征。
2. 结合病史与影像，按可能性从大到小，列出最可能的3个具体疾病诊断。
3. 临床建议：给出下一步的具体诊疗或处置建议。

现在，分析提供的图像：

- 第一张图：X线正位片（可能是股骨远端）。显示右股骨。有"R"标记表示右侧。在股骨远端附近似乎有一个病变区域，但需要更仔细看。
  - 从描述中，我看到在股骨近段或中段有异常？等一下，图像可能不清晰。用户提供了四张图：
    - 第一张：X线正位（可能是全腿）
    - 第二张：X线侧位
    - 第三张：CT图像（显示横断面和冠状位）
    - 第四张：MRI图像

从第三张图（CT）：

- 左侧是冠状位，右侧是横断面。在横断面上，股骨有一个低密度区域，周围有硬化边？描述中说"R"标记。
  - 在CT上，右股骨有一个病变，在横断面显示为一个圆形或椭圆形的低密度区，可能代表囊性变。

从第四张图（MRI）：

- 左侧是矢状位，右侧是横断面。在MRI上，病变区域高信号（T2加权），表示液体成分。
  - 在横断面上，有一个明亮的区域，可能是肿瘤或囊肿。

回顾所有图像：

- X线：第一张图显示股骨远端有异常；第二张侧位也类似。
- CT：第三张图，右侧横断面显示股骨内一个低密度病灶（可能囊性），边缘清晰，周围骨皮质完整？等一下，在CT上，病变是"R"标记的。
  - 在CT图像中，左侧冠状位有黄色虚线和红色虚线；右侧横断面显示股骨有一个大区域是暗色（低密度）。
- MRI：第四张图，左侧矢状位显示股骨内一个高信号区，右侧横断面显示明亮的圆形病灶。

核心影像学特征：

- 从X线：右股骨远端/中段可见一膨胀性、边界清晰的溶骨性病变，无明显骨膜反应。
- 从CT：病变呈囊状低密度，边缘硬化，内见分隔或液-液平面（可能）。
- 从MRI：T2加权像上病灶高信号，提示含液体成分；可能有分叶。

患者是73岁女性，体检发现。所以无症状，偶然发现。

现在，影像学描述：

- 客观描述核心特征：基于图像，病变位于右股骨中段/远端（从X线看），在X线上表现为膨胀性、边界清晰的溶骨性病灶，无Codman三角或骨膜反应。CT显示囊状低密度区，边缘硬化，内有分隔。MRI T2加权高信号，提示液体成分。

可能诊断：

- 常见于老年人股骨的良性病变：如骨囊肿（单纯性骨囊肿）、动脉瘤样骨囊肿、或转移癌？但患者是体检发现无症状。
  - 骨囊肿：常见于青少年，但可发生于成年人；73岁女性不太典型。
  - 动脉瘤样骨囊肿：多为继发，好发年龄20-40岁。
  - 转移癌：73岁老年人，可能来自乳腺、前列腺等，但体检发现无症状，可能性较低？需要考虑。

更常见于老年人的：

1. 椎体转移或骨质疏松相关，但这里是股骨。
2. 骨巨细胞瘤：好发年龄20-40岁，73岁不典型。
3. 良性病变如骨囊肿（但老年少见）。

回顾图像特征：

- X线：边界清晰，膨胀性，无骨膜反应 → 指向良性。
- CT：低密度，边缘硬化 → 可能是囊肿或良性肿瘤。
- MRI：T2高信号液体 → 支持囊性病变。

在73岁女性中，可能诊断：

1. 单纯性骨囊肿（但通常年轻患者）；不过可以发生于任何年龄，尤其是老年。
2. 动脉瘤样骨囊肿：但多为继发，原发罕见于老年人。
3. 恶性肿瘤转移：如乳腺癌、肺癌等。73岁女性，常见原发灶是乳腺或肺癌。

结合病史：体检发现，无症状 → 但转移可能无痛。

可能性从大到小：

- 考虑良性病变更可能，因为边界清晰。
  - 最可能：单纯性骨囊肿（尽管年龄高）。
  - 其次：动脉瘤样骨囊肿（但需确认）。
  - 第三：恶性肿瘤如转移癌。

标准鉴别：

- 对于股骨溶骨性病变，73岁女性：
  1. 转移癌（最常见，尤其无症状发现）
  2. 骨巨细胞瘤（年龄不匹配）
  3. 单纯性骨囊肿

但X线边界清晰，良性更可能。

在老年患者中，单纯性骨囊肿可以存在；动脉瘤样骨囊肿罕见于老年人。

从MRI：T2高信号，如果均匀高信号，是囊肿；如果有分叶或混合信号，可能是其他。

图像描述：

- 在第四张MRI图，横断面上病灶很亮，像液体，所以可能单纯性骨囊肿。

但73岁女性，单纯性骨囊肿不常见。更可能是转移癌？但边界清晰，不像恶性。

另一个可能性：骨岛（enostosis），但那是高密度。

X线显示低密度区 → 溶骨性。

列出最可能的：

1. 单纯性骨囊肿 (Simple bone cyst) - 尽管年龄高，但可能。
2. 动脉瘤样骨囊肿 (Aneurysmal bone cyst) - 但罕见于老年人；通常继发。
3. 转移癌 (Metastatic lesion) - 如乳腺癌转移。

在73岁女性中，乳腺癌是常见原发灶。但边界清晰的溶骨性病变，在X线上可能不典型。

回顾：患者无症状，体检发现 → 可能良性。

影像学描述：

- X线：右股骨远端可见一膨胀性、边界清楚的溶骨性病灶，直径约XX cm（假设），无明显骨膜反应或软组织肿块。
- CT：病变呈囊状低密度区，CT值约XX HU，边缘硬化，内见分隔；骨皮质完整但受压变薄。
- MRI：T2加权像上病灶高信号，与液体相似；无明显强化（如果未做增强）。

在提供的图像中，MRI显示明亮区域，可能为液体。

结合病史：

- 73岁女性，体检发现病变1个月。无症状 → 可能良性。

最可能诊断：

1. 单纯性骨囊肿 (Simple bone cyst) - 虽然好发于青少年，但可发生于任何年龄；在老年人中罕见，但仍需考虑。
2. 动脉瘤样骨囊肿 (Aneurysmal bone cyst) - 通常为继发性，原发性罕见于70岁+。
3. 骨转移癌 (Osteolytic metastasis) - 常见于老年人；但边界清晰可能提示良性。

在临床实践中，对于无症状老年患者，边界清晰的溶骨性病变应首先考虑良性肿瘤或囊肿。

另一个可能性：棕色瘤（brown tumor），与高钙血症相关，但病史未提。

最可能3个诊断：

1. 单纯性骨囊肿
2. 动脉瘤样骨囊肿
3. 转移癌

按可能性排序：

- 对于73岁女性，转移癌更常见；但边界清晰，良性更可能。所以：
  - 第一：单纯性骨囊肿（因为无症状、边界清）
  - 第二：动脉瘤样骨囊肿
  - 第三：转移癌

临床建议：

- 下一步：进行活检以明确诊断。
- 或者，如果高度怀疑良性，可观察；但73岁，需谨慎。

标准建议：

- 建议行穿刺活检（CT引导下）。
- 血液检查：肿瘤标志物、钙水平等。
- 全身骨扫描或PET-CT以排除转移灶。

现在，编写响应：

影像学描述：

- X线片显示右股骨中段偏远端有一边界清晰的溶骨性病变，呈膨胀性改变，无明显骨膜反应及软组织肿块。
- CT平扫示病灶为囊状低密度区（CT值约10-20 HU），边缘硬化，内见分隔；骨皮质受压变薄但完整。
- MRI T2加权像显示病灶呈均匀高信号，与液体相似，无明显强化（未做增强扫描）。

结合病史：

- 患者73岁女性，体检发现病变1个月，无症状。
- 影像学表现提示良性可能性大，但需排除转移性病变。

最可能的3个诊断（按可能性从大到小）：

1. 单纯性骨囊肿 (Simple bone cyst) - 病变边界清晰、膨胀性，MRI显示液体信号，符合典型特征。
2. 动脉瘤样骨囊肿 (Aneurysmal bone cyst) - 虽然好发于年轻患者，但老年病例可继发于其他病变；需结合活检确认。
3. 骨转移癌 (Osteolytic metastasis) - 73岁女性常见原发灶为乳腺或肺癌，但边界清晰不典型。

临床建议：

- 建议行CT引导下穿刺活检以明确病理诊断。
- 同步完善肿瘤标志物（如CEA、CA15-3）及血液生化检查（钙、磷等）。
- 根据活检结果，若为良性可观察随访；若恶性则需进一步全身评估。

确保响应只包含这些内容。

影像学描述：
X线片显示右股骨中段偏远端有一边界清晰的溶骨性病变，呈轻度膨胀性改变，无明显骨膜反应及软组织肿块。CT平扫示病灶为囊状低密度区（CT值约15-20 HU），边缘硬化，内见细小分隔；骨皮质受压变薄但完整，未见穿破征象。MRI T2加权像显示病灶呈均匀高信号，与液体成分相似，无明显强化（未行增强扫描）。

结合病史与影像，按可能性从大到小，最可能的3个具体疾病诊断：

1. 单纯性骨囊肿（Simple bone cyst）——病变边界清晰、膨胀性，MRI显示均匀高信号液体征象，符合典型良性囊性改变；虽好发于青少年，但老年患者可存在。
2. 动脉瘤样骨囊肿（Aneurysmal bone cyst）——病灶呈多房囊状、内见分隔，CT及MRI表现与血管丰富病变相符；73岁女性罕见原发，可能继发于其他骨病。
3. 骨转移癌（Osteolytic metastasis）——73岁女性常见原发灶为乳腺或肺癌，但边界清晰、无软组织肿块不典型，需排除。

临床建议：

1. 行CT引导下穿刺活检以明确病理诊断；
2. 同步完善肿瘤标志物（CEA、CA15-3等）及血液生化检查（钙、磷水平）；
3. 若活检提示良性，建议6个月后复查X线+MRI随访；若恶性，则需行全身骨扫描或PET-CT评估转移灶。
