# Supplementary material for: Diagnostic Performance of a Locally Deployed Vision Language Model for Bone Tumor Diagnosis Using Smartphone-Captured Images: Exploratory Retrospective Study
Source: JMIR AI. 2026 Sep 21;5:e99757. doi: 10.2196/99757 (PMC13639421; doi:10.2196/99757)
Supplement: Supplementary file 8 [file ai_v5i1e99757_app7.zip › Case_14/RAG/Radiologist.docx]

身份设定：你是一名省级三甲医院骨科影像学会诊专家（精通各类骨肿瘤与骨病影像鉴别）。请基于提供的X线、CT及MRI影像和极简病史，给出影像学研判。病变位置：右股骨 患者信息：女性 73岁 体检拍片发现右股骨病变1个月。要求：影像学描述：客观描述你观察到的核心影像学特征。结合病史与影像，按可能性从大到小，列出最可能的 3 个具体疾病诊断。临床建议：给出下一步的具体诊疗或处置建议。

首先，用户要求我作为省级三甲医院骨科影像学会诊专家，基于提供的X线、CT及MRI影像和极简病史进行研判。患者是73岁女性，体检拍片发现右股骨病变1个月。

关键部分：

- **影像学描述**：客观描述观察到的核心影像学特征。
- **诊断列表**：按可能性从大到小列出最可能的3个具体疾病诊断。
- **临床建议**：给出下一步的具体诊疗或处置建议。

我需要分析提供的四张图片：

1. 第一张：X线片（正位），显示右股骨。有“R”标记，表明是右侧。在股骨干近端区域有一个模糊的病变，看起来像一个不规则的透亮区或密度改变。
2. 第二张：X线片（侧位），同样显示右股骨。在股骨干处有一个类似病变，可能更清晰地显示了病变的位置和形态。
3. 第三张：CT图像。左侧是冠状面重建图，右侧是轴位切片。在股骨中段，有一个圆形的低密度区（黑色区域），周围有硬化边或反应性改变。黄色虚线标记位置。
4. 第四张：MRI图像。左侧是矢状面，右侧是轴位。在股骨内有一个高信号病变，在T2加权像上明显亮白。

回顾病史：

- 患者73岁女性。
- 体检发现右股骨病变1个月（无症状）。

现在，分析影像学特征：

**X线片：**

- 正位和侧位显示在股骨干中段有一个边界相对清晰的透亮区（低密度影），大小约2-3cm。周围有轻微硬化边，但不明显。没有明显的骨膜反应或软组织肿块。

**CT图像：**

- 轴位切片显示一个圆形、均匀的低密度病变，位于股骨髓腔内。
- 病变边界清晰，周围有薄层硬化边缘（在CT上表现为高密度环）。
- 没有明显的皮质破坏或软组织侵犯。

**MRI图像：**

- 轴位T2加权像显示病变呈均匀高信号（亮白），表明含液成分。
- 矢状面也显示病变为圆形、边界清晰的高信号区，周围骨髓无明显水肿。

核心影像学特征总结：

- 位置：右股骨干中段。
- 形态：圆形/类圆形病变，大小约2-3cm（估计）。
- X线：透亮区（低密度），边界较清，轻微硬化边。
- CT：均匀低密度，清晰边缘，周围薄层硬化环。
- MRI：T2高信号，表明液体成分；无明显骨髓水肿或软组织受累。

结合病史：

- 患者73岁女性，老年患者。股骨病变在体检中偶然发现，无症状。
- 常见于老年人的良性骨肿瘤包括骨囊肿、骨巨细胞瘤（但骨巨细胞瘤多见于20-40岁）、转移性肿瘤等。

可能诊断：

1. **单纯性骨囊肿**：常见于儿童和青少年，但也可发生在成人。在老年患者中较少见，但仍有可能。影像上表现为边界清晰的透亮区，MRI T2高信号。
   - 但在73岁女性，单纯性骨囊肿可能性较低，因为多发生于10-20岁。
2. **动脉瘤样骨囊肿**：通常在青少年和年轻成人中发生（10-30岁），但偶尔可发生在老年人。影像上为膨胀性病变，CT显示液-液平面，MRI有分层信号。
   - 从描述看，没有提到液-液平面；这里似乎是均匀高信号。
3. **骨转移瘤**：在73岁女性中很常见，尤其是如果原发肿瘤未知（如乳腺癌、肺癌等）。但病史说体检发现，无其他症状。影像上，转移灶常为溶骨性，边界不清，但有些可有硬化边。
   - 然而，这里病变边界清晰，周围有硬化环，不像典型的转移瘤（通常边界模糊）。
4. **骨巨细胞瘤**：好发于20-40岁，常见于股骨远端。在73岁老年人中罕见。
5. **骨纤维结构不良**：可发生在任何年龄，但多见于青少年。影像上为磨玻璃样改变，边界不清。
   - 这里不是典型的磨玻璃。
6. **良性肿瘤如软骨母细胞瘤**：罕见，好发于20-40岁。
7. **老年性退行性病变**：如骨岛、骨内生软骨瘤等。但影像特征不符合。

重新思考：

- 病变边界清晰，CT显示低密度区有硬化边，MRI T2高信号。
- 在老年人中，一个常见的良性病变是**骨囊肿**（包括单纯性骨囊肿），但年龄不典型。
- 另一个重要考虑：**骨转移瘤**。虽然转移灶通常边界不清，但在某些情况下（如溶骨性转移伴反应性硬化）可有清晰边缘。
  - 但患者无原发肿瘤病史，体检发现。

另一个可能性：

- **内生软骨瘤**：好发于手足短管状骨，但也可发生在长骨。在股骨中段少见；影像上为透亮区，常含钙化点（但这里没有提到）。
  - 在CT和MRI中，病变均匀低密度/高信号，无钙化。
- **骨淋巴瘤**：罕见，但可表现为溶骨性破坏，边界不清。

更可能的诊断：

1. **单纯性骨囊肿**：尽管年龄大，但有时可在成人发生。老年患者中骨囊肿较少见，但可能。
2. **动脉瘤样骨囊肿**：在老年人中少见，但偶尔发生；MRI T2高信号符合。
3. **转移性肿瘤**：考虑到73岁女性，无症状，体检发现。许多癌症如乳腺癌可转移到股骨。

但影像特征：

- 边界清晰、硬化边提示良性，而转移瘤通常边界不清（除非是反应性改变）。
- 在CT上，低密度区有硬化环，更支持良性病变。

参考标准知识：

- **单纯性骨囊肿**：儿童和青少年常见；成人中可复发或持续存在。影像：X线透亮区，边界清；CT均匀低密度；MRI T1低信号、T2高信号。
- 在73岁女性，这不太典型，但可能。

另一个常见于老年人的病变：

- **骨髓纤维化**：但通常不表现为局灶性囊肿样改变。

或许考虑：

- **慢性骨脓肿（Brodie脓肿）**：但无感染史；影像上为透亮区伴硬化边。
  - 但患者无症状，体检发现。

最可能的3个诊断（按可能性从大到小）：

1. **单纯性骨囊肿**：尽管年龄高，但影像特征匹配。在成人中，尤其老年人，骨囊肿可存在；X线、CT和MRI符合。
2. **动脉瘤样骨囊肿**：虽然年轻患者更常见，但老年也可发生；T2高信号支持。
3. **转移性肿瘤（如乳腺癌）**：考虑到年龄和性别，需排除。但边界清晰可能不典型。

在73岁女性中：

- 乳腺癌是常见癌症，可转移到骨。
- 影像上，转移灶通常为多发、溶骨性，边界不清；但单发病变也可发生，尤其如果反应性硬化。

从提供的图像看：

- X线：病变边界清晰（有硬化边）。
- CT：均匀低密度，周围硬化环。
- MRI：T2高信号，无水肿。

这更符合良性囊肿而非转移瘤。

其他可能性：

- **骨内腱鞘囊肿**：但罕见于股骨；通常发生在关节附近。

最终诊断列表（基于经验）：

1. **单纯性骨囊肿**（最可能） - 因为影像特征典型。
2. **动脉瘤样骨囊肿**（次之）
3. **转移性肿瘤**（需排除，但可能性较低）

在老年人中：

- 骨囊肿罕见；更常见的是其他病变。

另一个想法：**老年性骨髓炎或慢性骨脓肿**？但无症状，体检发现，不太可能。

或许考虑**骨岛**，但骨岛是致密的，这里不是透亮区。

回顾CT图像：

- 第三张图显示在股骨中段有一个大的低密度区域（黑色），周围有高密度环（硬化边）。
- 这很像一个囊肿。

MRI：T2高信号，表明液体内容物。

所以，最可能诊断是良性囊性病变。

具体疾病：

1. **单纯性骨囊肿**（Solitary bone cyst）
2. **动脉瘤样骨囊肿**（Aneurysmal bone cyst） - 但通常有液-液平面；这里似乎均匀高信号。
3. **内生软骨瘤**（Enchondroma） - 但常含钙化点，在CT上应可见，而图像中无描述。

在第四张MRI图，病变是均匀的T2高信号，没有分层。

因此：

- 第1：单纯性骨囊肿
- 第2：动脉瘤样骨囊肿（如果考虑液-液平面可能被遗漏）
- 但更可能：**转移性肿瘤**

我需要基于年龄和性别。

73岁女性，股骨病变：

- 常见原因：原发骨肿瘤罕见；更可能是转移灶或良性病变。
- 在体检中发现无症状的病变更支持良性。

在医学文献中：

- 70岁以上患者，孤立性骨病变最常见是转移瘤（50%以上），其次是良性肿瘤如囊肿、骨巨细胞瘤等。

但影像上边界清晰提示良性。

标准方法：

- 如果病变边界清楚、硬化边、无软组织侵犯，则更可能良性；如果边界不清、皮质破坏则恶性。

这里：边界清，有硬化边（CT显示周围高密度环），所以良性可能性大。

在73岁女性中：

1. **单纯性骨囊肿** - 尽管年龄高，但可发生。
2. **动脉瘤样骨囊肿**
3. **内生软骨瘤** - 但在股骨长干骨少见；多见于手足骨。

另一个常见良性病变：**骨纤维结构不良**（Fibrous dysplasia），但影像上为磨玻璃状，边界不清，这里不匹配。

所以：

1. 单纯性骨囊肿
2. 动脉瘤样骨囊肿
3. 转移性肿瘤

但转移性肿瘤在边界清晰时可能性较低；可能需要考虑其他。

或许**骨巨细胞瘤** - 但在73岁罕见，且好发于远端股骨（这里位置是骨干）。

病变位置：从X线看，在股骨干中段。股骨巨细胞瘤多见于远端（膝关节附近），但有时可发生在近端或骨干。

在第二张X线图，似乎靠近膝关节区域？但描述说"右股骨"，病史提到"体检拍片发现右股骨病变1个月"，没有指定位置。从图像看，在股骨中下段。

在第三张CT，黄色虚线标记位置；在第四张MRI，红色虚线标记。

大致位置：股骨干中段到远端。

对于73岁女性：

- 骨囊肿可能性低（50%以上发生于20岁以下）。
- 更可能考虑转移或良性病变。

**关键点：** 在老年人，孤立性骨病变需排除转移瘤。但影像特征支持良性。

最可能的三个诊断：

1. **单纯性骨囊肿** - 由于边界清晰、T2高信号。
2. **动脉瘤样骨囊肿**
3. **慢性骨脓肿（Brodie脓肿）**

但无感染史，可能性低。

另一个：**骨淋巴瘤** - 罕见。

基于常见情况：

1. 转移性肿瘤
2. 单纯性骨囊肿
3. 动脉瘤样骨囊肿

但影像不支持转移（边界清晰）。

我查一下标准知识。

在临床实践中，对于无症状老年患者，孤立性溶骨性病变伴硬化边：

- 最可能：良性骨肿瘤如骨囊肿或内生软骨瘤。
- 但年龄高，需考虑转移。

然而，在CT和MRI上，均匀T2高信号更支持囊肿而非转移（转移通常异质）。

所以：

1. **单纯性骨囊肿** - 尽管年龄不典型
2. **动脉瘤样骨囊肿**
3. **转移性肿瘤**

按可能性排序：在73岁女性中，转移更常见，但影像特征不符合；因此可能良性病变更可能。

或许诊断为：

1. 单纯性骨囊肿 (40%)
2. 动脉瘤样骨囊肿 (30%)
3. 转移性肿瘤 (30%)

但需要具体疾病名称。

列出三个：

1. 简单骨囊肿（Solitary bone cyst）
2. 动脉瘤样骨囊肿（Aneurysmal bone cyst）
3. 骨转移瘤（如乳腺癌）

临床建议：

- 下一步：由于患者无症状，但73岁，需排除恶性肿瘤。
- 建议：进行全身骨扫描或PET-CT以评估是否有其他病灶；如果单发，则行活检以明确诊断。

具体：

1. 病理活检（穿刺活检）以确诊
2. 全身检查排除原发肿瘤

但患者体检发现，无症状，可能先做进一步影像。

根据指南：对于孤立性骨病变，在老年人中，推荐进行活检或全身评估。

现在，写响应：

**影像学描述：**

- X线：右股骨干中段可见一圆形、边界清晰的透亮区（直径约2.5cm），周围见薄层硬化边。无骨膜反应及软组织肿块。
- CT：轴位图像显示病变呈均匀低密度，边界锐利，周围有环状硬化带；冠状面重建示病变位于髓腔内，范围局限。
- MRI：T2加权像（轴位）表现为明显高信号病灶，边界清晰；矢状面示病变圆形、无骨髓水肿及软组织侵犯。

**诊断列表（按可能性从大到小）：**

1. 单纯性骨囊肿
2. 动脉瘤样骨囊肿
3. 骨转移瘤（考虑乳腺癌等原发肿瘤）

**临床建议：**

- 建议行全身骨扫描或PET-CT检查以排除多发性病变及寻找潜在原发灶。
- 如单发病变，推荐经皮穿刺活检获取病理诊断，明确良恶性。
- 根据活检结果制定进一步治疗方案（如良性则随访；恶性需综合治疗）。

确保响应只包含这些部分，并且是中文。

影像学描述：
X线片显示右股骨干中段可见一圆形、边界清晰的透亮区，直径约2.5cm，周围见薄层硬化边，无骨膜反应及软组织肿块。CT轴位图像示病变呈均匀低密度，边界锐利，周围有环状硬化带；冠状面重建证实病变位于髓腔内，范围局限，未突破皮质。MRI T2加权像（轴位）表现为明显高信号病灶，边界清晰，无骨髓水肿及软组织侵犯；矢状面示病变呈圆形，内部信号均匀。

结合病史与影像，按可能性从大到小列出最可能的3个具体疾病诊断：

1. 单纯性骨囊肿（Solitary bone cyst）—— 73岁女性虽年龄偏高，但影像特征典型：边界清晰、硬化边、T2高信号提示液性内容物，符合良性囊性病变。
2. 动脉瘤样骨囊肿（Aneurysmal bone cyst）—— 老年患者中罕见，但MRI T2高信号及CT低密度表现可支持；需排除液-液平面（本例未明确显示）。
3. 骨转移瘤（如乳腺癌转移）—— 73岁女性为骨转移高危人群，但影像边界清晰、硬化边明显，与典型溶骨性转移不符；需优先排查原发肿瘤。

临床建议：

1. 行全身骨扫描或PET-CT检查，以排除多发性病变及寻找潜在原发灶（尤其乳腺癌等常见恶性肿瘤）。
2. 如单发病变且无症状，推荐经皮穿刺活检获取病理诊断，明确良恶性。
3. 根据活检结果制定方案：若为良性则定期随访；若为恶性需多学科会诊并行综合治疗（如放疗、化疗或手术）。
