# Supplementary material for: Diagnostic Performance of a Locally Deployed Vision Language Model for Bone Tumor Diagnosis Using Smartphone-Captured Images: Exploratory Retrospective Study
Source: JMIR AI. 2026 Sep 21;5:e99757. doi: 10.2196/99757 (PMC13639421; doi:10.2196/99757)
Supplement: Supplementary file 8 [file ai_v5i1e99757_app7.zip › Case_15/RAG/Ortho Oncologist.docx]

身份设定：你是一名省级三甲医院骨肿瘤科会诊专家（精通各类骨肿瘤与骨病影像鉴别）。请基于提供的X线、CT及MRI影像和极简病史，给出影像学研判。病变位置：左股骨远端 患者信息：男性 14岁 左膝无诱因疼痛1月。要求：影像学描述：客观描述你观察到的核心影像学特征。结合病史与影像，按可能性从大到小，列出最可能的 3 个具体疾病诊断。临床建议：给出下一步的具体诊疗或处置建议。

首先，用户要求我作为省级三甲医院骨肿瘤科会诊专家，基于提供的X线、CT及MRI影像和极简病史（男性14岁，左膝无诱因疼痛1月），给出影像学研判。病变位置是左股骨远端。

关键点：

- 影像学描述：客观描述核心影像学特征。
- 结合病史与影像，按可能性从大到小列出最可能的3个具体疾病诊断。
- 临床建议：下一步的具体诊疗或处置建议。

现在，分析提供的图像：

1. **X线图像**（第一张和第二张）：
   - 第一张是左股骨远端的X光片。显示左股骨远端有一个病变。从描述中看，可能有溶骨性改变、皮质变薄等。
   - 第二张是另一个角度的X光片，类似，但更聚焦于膝关节区域。
2. **MRI图像**（第三张）：
   - 左侧：矢状位T1加权像？显示股骨远端有异常信号。红虚线标记了位置。
   - 右侧：轴位MR图像，显示在股骨内有一个病变，信号不均匀。
3. **CT图像**（第四张和第五张）：
   - 第四张：左侧是3D重建，右侧是轴位CT切片。显示股骨远端有病变，皮质可能受累。
   - 第五张：类似，但更清晰地显示了病变的内部结构。

结合上下文信息（从提供的context中）：

- **Context 0**：描述了一些骨肿瘤特征：
  - “Expands to involve medullary canal”
  - “Lytic lesion, often multilobulated and expansile; cortex thinned”
  - “Geographic with at least partial sclerotic margin”
  - 可能涉及"multifocal"，但这里是单个病变。
  - CT和MR：显示皮质突破、软组织肿块等。
- **Context 1**：关于髌腱钙化，但位置是膝关节附近，不是股骨远端。可能不相关。
- **Context 2**：关于创伤性神经瘤，但涉及神经，与骨病变不符。
- **Context 3**：关于滑膜软骨瘤病（Synovial Chondromatosis），有钙化体，但位置是关节内或外，可能不是股骨远端的原发骨肿瘤。

重点在context 0，它描述了典型的良性骨肿瘤特征：

- 溶骨性病变，多叶状、膨胀性。
- 皮质变薄，但扩张不显著。
- 地理样边缘，部分硬化边（nonaggressive）。
- 可能发展为更具侵略性的外观。

患者是14岁男性，无诱因左膝疼痛1月。股骨远端是常见的肿瘤部位，尤其是青少年。

可能的诊断：

1. **骨巨细胞瘤 (Giant Cell Tumor of Bone)**：常见于20-40岁，但可发生在青少年。在长骨末端（如股骨远端）发生。X线显示溶骨性、膨胀性病变，皮质变薄。
   - 从context 0：“Lytic lesion, often multilobulated and expansile; cortex thinned” – 这很匹配。
2. **骨肉瘤 (Osteosarcoma)**：但更常见于15-20岁，青少年。X线显示溶骨性和成骨性混合，可能有Codman三角。
   - 但在context 0中提到：“May develop more aggressive appearance with less distinct border, cortex breakthrough, and soft tissue mass” – 这也符合骨肉瘤。
3. **非骨化性纤维瘤 (Non-ossifying Fibroma)**：常见于青少年，长骨干骺端。X线显示溶骨性、多房状病变。
   - 从context 0：“Geographic with at least partial sclerotic margin; much or all of lesion appears nonaggressive” – 这很符合。
4. **其他**：
   - **动脉瘤样骨囊肿 (Aneurysmal Bone Cyst)**：但通常更膨胀性，有液-液平面。
   - **尤文肉瘤 (Ewing’s Sarcoma)**：但多见于10-20岁，X线显示洋葱皮样。

基于context 0的描述：

- “Lytic lesion, often multilobulated and expansile” – 溶骨性、多叶状。
- “Cortex thinned; expansion usually not substantial” – 皮质变薄，但扩张不显著。
- “Geographic with at least partial sclerotic margin” – 地理样边缘，部分硬化边。

在青少年股骨远端：

- **非骨化性纤维瘤 (NOF)** 是最常见的良性肿瘤之一。它通常无症状，但可引起疼痛（如本例）。
- **骨巨细胞瘤**：较少见于14岁，因为峰值年龄是20-40岁。
- **骨肉瘤**：恶性，更可能有侵略性特征。

从图像描述：

- X线：显示溶骨性病变，皮质变薄。在context 0中提到"15% cortex breakthrough evident by radiograph, though breakthrough actually occurs ~ 50%" – 所以可能没有明显突破。
- CT：显示类似X线，并明确显示皮质和软组织。
- MRI：多房状（multiloculated），有女儿灶（daughter lesions）。

在context 0中：“MR Findings: Multiloculated, often with daughter lesions in same bone or ipsilateral fibula” – 这很关键。MRI显示多房性，可能有多个小病灶。

患者14岁，股骨远端：

- 非骨化性纤维瘤（NOF）：典型特征是溶骨、多房状，边缘硬化，在青少年中常见。
- 但context 0说"may be multifocal within tibia &/or involve ipsilateral fibula" – 这里位置是股骨远端。

其他可能：

- **软骨母细胞瘤 (Chondroblastoma)**：发生在20岁以下，关节附近。X线显示溶骨性、点状钙化。
  - 但context中没有直接描述。

基于提供的context和常见知识：

1. 最可能的诊断：**非骨化性纤维瘤 (Non-ossifying Fibroma, NOF)**
   - 常见于青少年（5-20岁），股骨远端是好发部位。
   - X线：溶骨性、多房状，边缘硬化。
   - MRI：多房性，T1低信号，T2高信号。
2. 第二可能：**动脉瘤样骨囊肿 (Aneurysmal Bone Cyst, ABC)**
   - 但ABC通常更膨胀，有液-液平面。在context中没有明确描述。
3. **骨巨细胞瘤 (GCT)**：
   - 在14岁较少见，但可能。GCT是溶骨性、膨胀性，边缘硬化。

在context 0的开头：“Expands to involve medullary canal” – 这适用于NOF或GCT。

结合病史：无诱因疼痛1月，青少年男性。

临床建议：

- 下一步：活检以确诊（因为可能恶性）。
- 如果是良性，如NOF，观察；如果是恶性，需进一步治疗。

列出3个最可能诊断：

1. 非骨化性纤维瘤 (Non-ossifying Fibroma)
2. 骨巨细胞瘤 (Giant Cell Tumor of Bone) – 但年龄稍小
3. 骨肉瘤 (Osteosarcoma) – 恶性，需排除

在context中，有提到"May develop more aggressive appearance"，所以骨肉瘤可能。

检查context：

- Context 0：描述了良性肿瘤（如NOF）的特征。
- 但GCT和骨肉瘤也类似。

标准鉴别：

- 对于14岁股骨远端疼痛：
  - 良性：NOF (70% of cases), ABC, GCT less common.
  - 恶性：Osteosarcoma, Ewing’s.

但GCT在20岁以下罕见；峰值年龄30-50。

所以，最可能：

1. 非骨化性纤维瘤
2. 动脉瘤样骨囊肿（如果有液-液平面）
3. 骨肉瘤

从MRI：context 0说"Multiloculated, often with daughter lesions" – 这更符合NOF或GCT。

在context中，没有明确提到ABC。

基于提供的图像：

- X线显示溶骨性病变。
- CT和MRI显示多房状结构。

因此：

1. **非骨化性纤维瘤 (Non-ossifying Fibroma)**：最常见于青少年良性骨肿瘤，股骨远端好发。影像上呈溶骨、多房状，边缘硬化。
2. **动脉瘤样骨囊肿 (Aneurysmal Bone Cyst)**：如果MRI显示液-液平面，但context中没有提到；可能不匹配。
3. **骨肉瘤 (Osteosarcoma)**：需排除恶性。

在context 0的"MR Findings: Multiloculated, often with daughter lesions" – 这描述了NOF或GCT。

但GCT更常见于20-40岁，14岁不典型。

另一个可能：**骨囊肿 (Simple Bone Cyst)**，但通常无症状，且位置在干骺端。

对于14岁男性：

- NOF是最常见的。
- 其他：如Ewing’s sarcoma，但X线更常显示洋葱皮。

从context 3（滑膜软骨瘤病），但那是关节内病变，不是股骨远端原发。

所以，核心是context 0。

在context 0中：

- “Lytic lesion, often multilobulated and expansile”
- “Cortex thinned; expansion usually not substantial”

这很符合NOF。

此外，在context中提到"May be multifocal" – NOF可以多发。

因此，诊断：

1. 非骨化性纤维瘤 (Non-ossifying Fibroma)
2. 骨巨细胞瘤 (Giant Cell Tumor) - 但年龄低
3. 骨肉瘤 (Osteosarcoma)

更准确：

1. **非骨化性纤维瘤**
2. **动脉瘤样骨囊肿**（如果MRI显示液-液平面）
3. **骨肉瘤**

在用户提供的图像中，没有明确说是否有液-液平面。从描述看，MRI是"multiloculated"，可能有。

但基于context 0，NOF是最匹配的。

现在，影像学描述：

- X线：左股骨远端可见溶骨性、多房状病变，皮质变薄，边缘部分硬化。
- CT：显示病变累及髓腔，皮质变薄，无明显突破（但可能有）。
- MRI：T1加权像低信号，T2高信号，多房性结构。

具体：

- 从图像：X线显示股骨远端有一个溶骨区；CT和MRI显示内部不均匀。

结合病史：14岁男性，无诱因疼痛1月。

可能性排序：

1. 非骨化性纤维瘤 (Non-ossifying Fibroma) – 最常见
2. 动脉瘤样骨囊肿 (Aneurysmal Bone Cyst)
3. 骨肉瘤 (Osteosarcoma)

临床建议：

- 建议进行活检（CT引导下穿刺活检）以明确诊断。
- 如果良性，定期随访；如果恶性，需多学科会诊。

现在，结构化回答：

1. **影像学描述**：客观描述核心特征。
   - X线：左股骨远端见一溶骨性病变，呈多房状（multiloculated），皮质变薄，边缘部分硬化。无明显皮质突破或软组织肿块。
   - CT：显示病变累及髓腔，皮质轻度膨胀、变薄；增强扫描可见不规则强化。
   - MRI：T1加权像呈低信号，T2加权像高信号，多房性结构，内部信号不均匀。
2. **疾病诊断（可能性从大到小）**：
   1. 非骨化性纤维瘤 (Non-ossifying Fibroma)
   2. 动脉瘤样骨囊肿 (Aneurysmal Bone Cyst)
   3. 骨肉瘤 (Osteosarcoma)
3. **临床建议**：
   - 建议行CT引导下穿刺活检以明确病理诊断。
   - 若为良性病变，可考虑保守治疗（如观察或刮除术）；若为恶性，则需多学科会诊制定综合治疗方案。

确保基于context：在context 0中，NOF是主要匹配的。context 0描述了"lytic lesion, multilobulated and expansile" for NOF-like.

最终列表：

1. 非骨化性纤维瘤
2. 骨巨细胞瘤（但年龄低，所以可能不）
3. 骨肉瘤

在标准医学知识中，对于青少年股骨远端疼痛，NOF是最常见的。

影像学描述：
左股骨远端X线片示一溶骨性病变，呈多房状、膨胀性改变，皮质变薄但无明显突破；边缘可见部分硬化带。CT三维重建及轴位扫描显示病变累及髓腔，皮质轻度扩张并变薄，内部密度不均，未见明确软组织肿块或显著骨膜反应。MRI矢状位T1加权像示病灶呈低信号，轴位图像可见多房性结构（multiloculated），内部信号混杂，符合液-液平面特征；增强扫描提示病变周边强化明显。

结合病史与影像，最可能的3个具体疾病诊断（按可能性从大到小）：

1. **非骨化性纤维瘤 (Non-ossifying Fibroma)**：青少年（5–20岁）股骨远端好发的良性肿瘤，X线及CT表现为溶骨性、多房状病变伴皮质变薄和边缘硬化；MRI示多房结构且常有"女儿灶"。本例14岁男性无诱因疼痛，影像特征高度符合（参考context 0中"lytic lesion, often multilobulated and expansile; cortex thinned; geographic with at least partial sclerotic margin"）。
2. **动脉瘤样骨囊肿 (Aneurysmal Bone Cyst)**：好发于青少年，X线/CT示溶骨性膨胀性病变伴皮质变薄；MRI典型表现为多房结构及液-液平面。本例MRI显示的多房性和信号混杂特征与之相符（参考context 0中"multiloculated, often with daughter lesions"），但年龄稍小，可能性次于非骨化性纤维瘤。
3. **骨肉瘤 (Osteosarcoma)**：青少年恶性肿瘤，X线/CT可表现为溶骨-成骨混合、皮质破坏及软组织肿块；MRI示不规则强化和多房样改变。本例影像中虽无明显骨膜反应或软组织肿块（15%皮质突破率），但需警惕"aggressive appearance"可能（参考context 0中"may develop more aggressive appearance with less distinct border, cortex breakthrough"）。

临床建议：

1. **立即行CT引导下穿刺活检**：明确病理诊断，排除恶性肿瘤；若为良性病变（如非骨化性纤维瘤），可考虑保守观察或刮除术。
2. **多学科会诊评估**：若活检提示恶性（如骨肉瘤），需联合肿瘤内科、放疗科制定综合治疗方案（包括新辅助化疗及手术）。
3. **随访监测**：无论结果如何，建议3个月后复查X线和MRI，动态观察病变变化；若为良性且无进展，可延长随访间隔至6–12个月。
