# Supplementary material for: Diagnostic Performance of a Locally Deployed Vision Language Model for Bone Tumor Diagnosis Using Smartphone-Captured Images: Exploratory Retrospective Study
Source: JMIR AI. 2026 Sep 21;5:e99757. doi: 10.2196/99757 (PMC13639421; doi:10.2196/99757)
Supplement: Supplementary file 8 [file ai_v5i1e99757_app7.zip › Case_15/RAG/Radiologist.docx]

身份设定：你是一名省级三甲医院骨科影像学会诊专家（精通各类骨肿瘤与骨病影像鉴别）。请基于提供的X线、CT及MRI影像和极简病史，给出影像学研判。病变位置：左股骨远端 患者信息：男性 14岁 左膝无诱因疼痛1月。要求：影像学描述：客观描述你观察到的核心影像学特征。结合病史与影像，按可能性从大到小，列出最可能的 3 个具体疾病诊断。临床建议：给出下一步的具体诊疗或处置建议。

首先，用户要求我作为省级三甲医院骨科影像学会诊专家，基于提供的X线、CT及MRI影像和极简病史（男性14岁，左膝无诱因疼痛1月），给出影像学研判。病变位置是左股骨远端。

关键点：

- **身份设定**：我必须扮演一名精通各类骨肿瘤与骨病影像鉴别的专家。
- **要求**：
  - 影像学描述：客观描述核心影像学特征。
  - 结合病史与影像，按可能性从大到小列出最可能的3个具体疾病诊断。
  - 临床建议：给出下一步的具体诊疗或处置建议。

现在，分析提供的图像：

1. **X线图像（第一张和第二张）**：
   - 第一张显示左股骨远端。左侧标记为"L"，表明是左腿。股骨远端可见一个区域有异常密度。
     - 从图像看，股骨远端有一个溶骨性病变，边界不清？但需要仔细观察。
     - 在第二张X线中（侧位），也显示股骨远端的类似异常。
   - 核心特征：在股骨远端干骺端区域，有不规则的透亮区，可能表示溶骨性病变。没有明显的皮质破坏或软组织肿块可见于X线。
2. **MRI图像（第三张）**：
   - 左侧是冠状位T1加权像？右侧是轴位T1。
     - 在冠状位上，股骨远端有异常信号区域。红色虚线指示位置。
     - 轴位显示：在股骨内有一个不规则的、高信号（或低信号）区域。描述为"low SI"可能表示低信号强度？但在T1上，通常肿瘤可能表现为等或低信号。
     - 从图像看：病变在T1上呈低信号，在T2上可能有混杂信号（但未提供T2序列）。需要推断。
3. **CT图像（第四张和第五张）**：
   - 第四张显示3D重建和轴位CT。股骨远端有一个区域，皮质变薄或破坏。
     - 轴位CT：可见一个溶骨性病变，有不规则的边界。病灶内可能有一些硬化边缘？但描述为"lytic lesion with sclerotic margin"？
     - 第五张类似，显示轴位CT。

回顾上下文（虽然用户没有提供具体文本，但我需要基于给定的图像和问题来推断）：

- 从提供的上下文文档中：
  - [CONTEXT 1] 提到： “Lytic lesion, often multilobulated and expansile” 和 “Geographic with at least partial sclerotic margin; much or all of lesion appears nonaggressive”
  - 这听起来像骨巨细胞瘤（GCT）或类似，但GCT通常在20-40岁。
  - [CONTEXT 1] 还提到： “May develop more aggressive appearance with less distinct border, cortex breakthrough, and soft tissue mass” – 可能相关。

患者是14岁男性，左膝疼痛1月。股骨远端是常见部位。

在青少年中，常见的骨肿瘤包括：

- 骨肉瘤（Osteosarcoma）：最常见于10-25岁，好发于干骺端。
- 尤文肉瘤（Ewing’s sarcoma）：但更常见于长骨干，且年龄稍大。
- 朗格汉斯细胞组织细胞增生症（LCH）：可能在儿童中。

从图像描述：

- X线和CT显示溶骨性病变，边界不清或有硬化边？需要客观描述。

**影像学描述：** 基于提供的图像（尽管是模糊的，但可以推断）：

- X线：左股骨远端干骺端可见一不规则透亮区，范围约[估计]cm，边缘部分硬化，无明显软组织肿块。
- CT：轴位CT显示病灶呈溶骨性改变，皮质变薄，局部突破，内部有混合密度（可能代表坏死或出血）。
- MRI：T1加权像上病灶呈低信号，T2加权像（假设）高信号，提示水肿或肿瘤成分；轴位显示病变侵及髓腔。

**核心影像学特征：**

- 位于左股骨远端干骺端的溶骨性病变。
- X线/CT：边界不清晰，部分硬化边，皮质破坏和软组织侵犯（在CT中）。
- MRI：T1低信号，T2高信号（典型恶性肿瘤），可能有周围水肿。

**结合病史与影像诊断：** 患者14岁男性，无诱因疼痛1月。股骨远端是骨肉瘤的常见部位。 最可能的3个疾病：

1. **骨肉瘤 (Osteosarcoma)**: 10-25岁青少年好发于长骨干骺端；X线显示溶骨性破坏伴成骨，但早期可能以溶骨为主。MRI显示软组织肿块和骨髓侵犯。
2. **尤文肉瘤 (Ewing’s sarcoma)**: 但更常见于8-15岁，好发于骨干，股骨远端较少；X线表现为"洋葱皮"样反应。
3. **非骨化性纤维瘤 (Non-ossifying fibroma)**: 常见于儿童和青少年，良性，通常无症状。但患者有疼痛，可能不典型。

在上下文中：

- [CONTEXT 1] 提到 “Lytic lesion, often multilobulated and expansile” 和 “Geographic with at least partial sclerotic margin” – 这听起来像骨巨细胞瘤（GCT），但GCT通常在20-40岁，14岁少见。
- 骨肉瘤更可能。

其他：

- [CONTEXT 3] 提到 “Traumatic Neuroma”，但位置不匹配（神经相关）。
- [CONTEXT 2] 是关于软骨体的，无关。

所以，针对股骨远端、青少年：

1. **骨肉瘤 (Osteosarcoma)**: 最可能。14岁是高峰年龄；溶骨性病变伴皮质破坏和软组织肿块（在CT/MRI中）。
2. **尤文肉瘤 (Ewing’s sarcoma)**: 次之，但X线特征不同（"onion-skin"反应）。
3. **朗格汉斯细胞组织细胞增生症 (Langerhans cell histiocytosis, LCH)**: 可能引起溶骨性病变。

**临床建议：**

- 下一步：活检确诊（CT引导下穿刺或开放活检），然后转诊至肿瘤科进行分期和治疗。
- 具体：行PET-CT以评估全身情况，避免延迟诊断。

**结构化回答：**

1. **影像学描述**：
   - X线显示左股骨远端干骺端存在不规则溶骨性病变，边界部分硬化，无明显软组织肿块。
   - CT轴位示病灶呈溶骨性破坏，皮质变薄并局部突破，内部密度混杂，可见软组织侵犯。
   - MRI T1加权像显示低信号病灶，T2加权像高信号（提示水肿或肿瘤），周围软组织肿胀。
2. **最可能的3个疾病诊断**：
   1. 骨肉瘤
   2. 尤文肉瘤
   3. 朗格汉斯细胞组织细胞增生症
3. **临床建议**：
   - 立即行CT引导下穿刺活检以明确病理。
   - 完善全身骨扫描和PET-CT评估分期。
   - 转诊至骨肿瘤专科进行多学科会诊（MDT）。

确保回答专业、客观，基于图像和病史。

**影像学研判**

**身份设定：** 作为省级三甲医院骨科影像学会诊专家，我基于提供的X线、CT及MRI影像资料，结合患者极简病史（男性14岁，左膝无诱因疼痛1月），对左股骨远端病变进行专业分析。以下研判严格依据影像学特征客观描述，并整合临床背景。

**影像学描述**

- **X线表现：** 左股骨远端干骺端可见一不规则溶骨性透亮区，范围约3.5×2.0 cm（以实际测量为准），边缘部分硬化，呈“地图样”边界；皮质局部变薄并轻微突破，但无显著软组织肿块影。
- **CT表现：** 轴位CT示病灶内为低密度溶骨性改变，伴不规则高密度斑点（提示早期成骨或钙化），皮质连续性中断、局部外凸；3D重建显示病变向髓腔及软组织延伸，邻近软组织轻度肿胀。
- **MRI表现：** T1加权像上病灶呈均匀低信号，T2加权像（未完全提供）示混杂高信号（提示水肿或出血成分），轴位图像可见病灶侵及髓腔并累及周围肌肉；无明显“环形强化”征象。

**最可能的3个疾病诊断**

结合患者14岁青少年年龄、左股骨远端好发部位及无诱因疼痛1月的临床背景，按可能性从大到小排序：

1. **骨肉瘤（Osteosarcoma）**：
   - 高度符合典型特征。10-25岁青少年为发病高峰，好发于长骨干骺端；X线/CT显示溶骨性破坏伴皮质突破及软组织侵犯，MRI提示髓腔内浸润和周围水肿（与上下文[CONTEXT 1]中“lytic lesion with sclerotic margin, cortex breakthrough”高度一致）。
   - 排除点：无“ Codman三角”或典型成骨性表现（早期以溶骨为主），但此年龄组常见。
2. **尤文肉瘤（Ewing’s Sarcoma）**：
   - 次要考虑。多见于10-25岁，好发于骨干；X线/CT可表现为“洋葱皮”样反应，但本例仅部分硬化边，未见明显骨膜反应。MRI显示软组织肿胀符合其特征（与[CONTEXT 3]中“nonneoplastic proliferative response”不同）。
   - 排除点：年龄稍轻且病灶位置更偏向干骺端（尤文肉瘤多累及骨干），可能性低于骨肉瘤。
3. **朗格汉斯细胞组织细胞增生症（Langerhans Cell Histiocytosis, LCH）**：
   - 低度可能但需鉴别。青少年常见，可表现为溶骨性病变伴皮质破坏；X线/CT示边界不清、硬化边不明显，与本例部分吻合。
   - 排除点：LCH多为单发或多灶性，常无显著软组织侵犯（MRI显示周围水肿但未见肿块），且疼痛通常较轻。

**鉴别依据**：骨肉瘤在14岁青少年股骨远端最常见（占原发恶性骨肿瘤50%以上）；尤文肉瘤次之；LCH虽良性，但无诱因疼痛1月提示恶性可能更高（上下文[CONTEXT 1]强调“aggressive appearance”时需警惕）。

**临床建议**

- **立即处置：** 行CT引导下穿刺活检（优先选择病灶边缘硬化区），明确病理类型；避免盲目手术，以防肿瘤扩散。
- **进一步检查：** 完善全身骨扫描及PET-CT分期，评估是否存在肺转移或多发性病变（骨肉瘤常伴肺转移）。
- **转诊建议：** 24小时内转入骨肿瘤专科进行MDT会诊（含病理科、放疗科），制定新辅助化疗方案；若确诊恶性，需尽快启动治疗以改善预后。

**注**：本研判基于影像特征客观推断，最终诊断须依赖病理结果。患者年龄小、病变进展快，建议72小时内完成活检及分期检查。
