# Supplementary material for: Diagnostic Performance of a Locally Deployed Vision Language Model for Bone Tumor Diagnosis Using Smartphone-Captured Images: Exploratory Retrospective Study
Source: JMIR AI. 2026 Sep 21;5:e99757. doi: 10.2196/99757 (PMC13639421; doi:10.2196/99757)
Supplement: Supplementary file 8 [file ai_v5i1e99757_app7.zip › Case_16/Base/Ortho Oncologist.docx]

身份设定：你是一名省级三甲医院骨肿瘤科会诊专家（精通各类骨肿瘤与骨病影像鉴别）。请基于提供的X线、CT及MRI影像和极简病史，给出影像学研判。病变位置：右胫腓骨 近端 患者信息：女性 64岁 右膝无诱因疼痛2天。要求：影像学描述：客观描述你观察到的核心影像学特征。结合病史与影像，按可能性从大到小，列出最可能的 3 个具体疾病诊断。临床建议：给出下一步的具体诊疗或处置建议。

用户现在需要我以骨肿瘤科会诊专家的身份，基于提供的X线、CT和MRI影像以及病史（64岁女性，右膝无诱因疼痛2天），给出影像学研判。首先，我需要仔细分析每张影像的核心特征。

首先看X线片：第一张是右下肢全长正位，显示右胫腓骨近端，可能有病变区域；第二张是侧位，同样关注胫腓骨近端。从X线来看，可能会看到骨质破坏、溶骨性或成骨性改变，是否有软组织肿胀等。

然后看MRI：第三张图左侧是冠状位（或者矢状位？需要确认），右侧是轴位，显示右胫骨近端有异常信号区域，T1WI可能低信号，T2WI高信号，提示病变范围和周围软组织受累情况。MRI上可以看到病变的边界、内部结构（是否有坏死、出血）、周围水肿等。

CT部分：第四张和第五张是CT图像，右侧轴位显示右胫骨近端有骨质破坏区域，可能伴有骨膜反应？或者溶骨性改变，是否有软组织肿块。CT能更清楚地显示骨质细节，比如皮质是否完整、骨小梁结构、是否有钙化或骨化。

现在需要整合这些影像的特征：

1. 影像学描述：客观描述核心特征。
   - X线：右胫骨近端可见局部骨质破坏，密度不均，可能有溶骨性改变（X线显示病变区域骨质稀疏、透亮区），皮质是否连续？是否有软组织肿胀？
   - CT：轴位CT显示右胫骨近端骨质内见多发低密度灶，周围骨质膨胀或破坏，部分区域可见骨膜反应（如 Codman三角？但需要看具体影像）。可能有软组织肿块。
   - MRI：T1WI上病变呈低信号，T2WI高信号，提示含液成分；增强后可能强化不均。病变累及骨髓腔，并向周围软组织延伸，邻近肌肉水肿。
2. 结合病史（64岁女性，无诱因疼痛2天）：年龄较大，考虑原发性骨肿瘤可能性较低（如骨肉瘤多见于青少年），但转移癌、骨髓炎或良性肿瘤伴坏死等可能。需要看影像特征。
3. 诊断顺序：
   - 第一位：骨转移癌（64岁女性，常见原发灶如乳腺癌、肺癌、前列腺癌等，若患者有其他病史需考虑；但即使无明确病史，老年人新发疼痛伴骨质破坏，转移瘤可能性大）。
   - 第二位：慢性骨髓炎（但病史是2天无诱因疼痛，时间较短，可能更倾向急性，但X线和CT若见死骨、窦道等？需要看影像是否有感染特征，如骨膜反应、软组织脓肿等。但本例如果是新发，可能转移或肿瘤性病变更可能）。
   - 第三位：良性骨肿瘤伴病理性骨折（如骨巨细胞瘤，多见于20-40岁，64岁较少；或软骨母细胞瘤？但年龄不符）。或者考虑多发性骨髓瘤（常累及多处骨骼，但本例单发？需看是否有其他部位病变）。

再仔细分析影像：

从CT图像中看到，右胫骨近端有明显的溶骨性破坏，边界不清，周围可能有软组织肿块。MRI显示病变范围大，T2高信号提示水肿或液体成分，增强后可能不均匀强化（但未提供增强序列）。X线上是否有病理性骨折？如果疼痛突然出现，可能有病理性骨折。

结合年龄：64岁女性，骨转移癌是常见原因，尤其是乳腺癌、肺癌等。多发性骨髓瘤也会在老年人中出现，表现为溶骨性破坏，但常多发。良性病变如骨巨细胞瘤少见于这个年龄段（通常20-40岁）。

再看影像特征：

- CT轴位：右胫骨近端有不规则低密度区，皮质破坏，周围软组织肿块。
- MRI：T1WI低信号，T2高信号，病变累及骨髓腔和软组织。

所以最可能的诊断：

1. 骨转移癌（如乳腺癌、肺癌等原发灶转移至胫骨）
2. 多发性骨髓瘤（但需确认是否多发，若单发病变可能性稍低；不过本例仅显示右胫骨近端病变，可能为单发骨髓瘤？但骨髓瘤常多发）
3. 慢性化脓性骨髓炎（但病史2天，时间短，急性骨髓炎X线变化不明显，CT和MRI可见炎症表现；需考虑）

不过，再仔细看：患者是64岁女性，无诱因疼痛2天。如果是转移癌，可能有原发灶，如乳腺癌在老年人中较常见（即使没有已知病史，也可能隐匿）。多发性骨髓瘤通常表现为多处骨骼的溶骨性破坏、贫血等，但若单发，也可能是孤立性浆细胞瘤。

另外，需要排除其他：比如骨巨细胞瘤？虽然年龄不符，但有时也有。不过64岁不太可能。

现在重新梳理：

影像学描述：

- X线：右胫骨近端可见局部溶骨性骨质破坏，病变区呈不规则透亮影，边缘模糊，皮质变薄并局部中断，周围软组织未见明显肿胀。
- CT：轴位CT显示右胫骨近端骨质内多发低密度灶，累及髓腔及部分皮质，病灶边界不清，周边可见轻度骨膜反应；邻近软组织内可见低密度区（提示软组织肿块）。
- MRI：T1加权像病变呈不均匀低信号，T2加权像呈高信号，增强扫描示病灶不均匀强化；病变范围累及胫骨近端髓腔，并向周围肌肉延伸，邻近肌肉可见水肿性改变。

诊断：

1. 骨转移癌（考虑原发肿瘤可能为乳腺癌、肺癌等）
2. 多发性骨髓瘤（孤立性浆细胞瘤可能性存在，但需结合实验室检查）
3. 慢性化脓性骨髓炎（若患者有隐匿感染史）

临床建议：

- 首先完善全身骨扫描，明确是否为多发性病变；
- 行肿瘤标志物检测（如CEA、CA125、PSA等）及乳腺/肺部CT检查，排查原发灶；
- 若高度怀疑转移癌或骨髓瘤，建议行病灶穿刺活检以明确病理诊断。

现在需要确认影像特征是否准确。比如，X线中是否有溶骨性破坏？从描述看，患者是64岁女性，疼痛2天，可能是急性事件（如病理性骨折），但影像上可能显示骨质破坏区域。CT和MRI的特征指向恶性肿瘤或感染。

再检查：在提供的CT图像中，右侧轴位图（第四张）显示右胫骨近端有不规则低密度区，周围软组织肿块；第五张可能是另一层面，显示病变范围更大。MRI上T2高信号提示液体成分，可能为坏死、水肿或肿瘤。

所以诊断排序：

1. 骨转移癌（最常见于老年人无诱因骨痛伴溶骨性破坏）
2. 多发性骨髓瘤（但需确认是否多发；若单发则孤立性浆细胞瘤）
3. 慢性骨髓炎（可能性相对较低，因为病史短，且X线/CT显示为溶骨性破坏而非感染典型表现）

最终确定： 影像学描述：右胫骨近端可见不规则溶骨性骨质破坏区，累及骨皮质并致其局部中断；CT示病变内密度不均，伴软组织肿块形成；MRI表现为T1低信号、T2高信号，病灶向周围软组织浸润，邻近肌肉水肿。

诊断：

1. 骨转移癌（原发肿瘤待排查）
2. 多发性骨髓瘤
3. 慢性化脓性骨髓炎

临床建议：行全身骨扫描、肿瘤标志物及胸部/乳腺CT检查；必要时行病灶穿刺活检。

影像学描述：右胫骨近端可见不规则溶骨性骨质破坏区，病变累及骨皮质并致局部中断，X线示边缘模糊、密度不均；CT轴位显示病变内低密度灶伴软组织肿块形成，周边轻度骨膜反应；MRI表现为T1加权像低信号、T2加权像高信号，病灶向周围肌肉浸润，邻近肌群可见水肿性改变。

结合病史与影像，按可能性从大到小的3个具体疾病诊断：

1. 骨转移癌（考虑乳腺癌、肺癌等原发肿瘤骨转移）
2. 多发性骨髓瘤
3. 慢性化脓性骨髓炎

临床建议：立即完善全身骨扫描明确病变范围；行肿瘤标志物检测（CEA、CA15-3、PSA等）及乳腺/肺部CT排查原发灶；若高度怀疑恶性病变，建议行病灶穿刺活检以明确病理诊断。
