# Supplementary material for: Diagnostic Performance of a Locally Deployed Vision Language Model for Bone Tumor Diagnosis Using Smartphone-Captured Images: Exploratory Retrospective Study
Source: JMIR AI. 2026 Sep 21;5:e99757. doi: 10.2196/99757 (PMC13639421; doi:10.2196/99757)
Supplement: Supplementary file 8 [file ai_v5i1e99757_app7.zip › Case_16/RAG/Ortho Oncologist.docx]

身份设定：你是一名省级三甲医院骨肿瘤科会诊专家（精通各类骨肿瘤与骨病影像鉴别）。请基于提供的X线、CT及MRI影像和极简病史，给出影像学研判。病变位置：右胫腓骨 近端 患者信息：女性 64岁 右膝无诱因疼痛2天。要求：影像学描述：客观描述你观察到的核心影像学特征。结合病史与影像，按可能性从大到小，列出最可能的 3 个具体疾病诊断。临床建议：给出下一步的具体诊疗或处置建议。

首先，用户要求我作为省级三甲医院骨肿瘤科会诊专家，基于提供的X线、CT及MRI影像和极简病史，给出影像学研判。病变位置是右胫腓骨近端，患者信息：女性64岁，右膝无诱因疼痛2天。

关键点：

- 影像学描述：客观描述观察到的核心影像学特征。
- 结合病史与影像，按可能性从大到小，列出最可能的3个具体疾病诊断。
- 临床建议：给出下一步的具体诊疗或处置建议。

我需要仔细分析提供的影像：

1. **X线图像**：
   - 第一张图：显示右膝区域。R标记表示右侧。胫骨和腓骨近端可见。似乎有异常，比如骨质破坏或肿块。
   - 第二张图：另一个角度的X线，可能显示胫骨远端（因为膝盖附近）。但病变位置是胫腓骨近端，所以这可能是膝关节区域。
2. **MRI图像**：
   - 第三张图：显示MRI扫描。左侧是冠状位或矢状位，右侧是横断面。有红色虚线标记，可能指示病变位置。在胫骨近端（因为膝盖附近），有一个高信号区，表示软组织肿块或骨髓水肿。
3. **CT图像**：
   - 第四张图：显示CT扫描。左侧是三维重建，右侧是横断面。有红色虚线标记，在右胫骨区域，似乎有骨质破坏和软组织肿块。
   - 第五张图：另一个CT视图，类似，但更清晰地显示病变。

从所有影像中提取核心特征：

- **X线**：
  - 右胫腓骨近端（即靠近膝关节处）可见不规则的骨质破坏区域。
  - 骨皮质可能有中断或侵蚀。
  - 没有明显的骨折线，但可能有溶骨性改变。
- **CT**：
  - 在横断面和三维视图中，显示胫骨近端有明显的骨质破坏（低密度区）。
  - 病变区域有软组织肿块延伸到周围。
  - 骨皮质不连续，可能有穿孔。
  - 软组织内可见钙化或骨化（在CT中高密度点）。
- **MRI**：
  - 在T2加权像上，病变区域显示高信号，表示液体或水肿。
  - 病变累及骨髓，并向周围软组织扩展。
  - 可能有分叶状边缘和不规则边界。

结合病史：64岁女性，右膝无诱因疼痛2天。这很短暂，但可能是急性发作。由于是肿瘤相关，可能不是新发的，而是症状突然出现。

核心影像学特征：

- 骨质破坏：溶骨性病变，累及胫骨近端。
- 软组织肿块：延伸到周围软组织。
- 无明显骨折，但有骨皮质侵蚀。
- MRI显示高信号，提示炎症或肿瘤活动。
- CT显示钙化/骨化成分。

现在，列出最可能的3个疾病诊断（按可能性从大到小）：

1. **原发性骨肿瘤**：如骨肉瘤、软骨肉瘤等。但患者64岁，骨肉瘤更常见于青少年；软骨肉瘤在老年人中更常见。
   - 骨肉瘤：好发于长骨干骺端，胫骨近端是常见部位。但年龄偏大（64岁），骨肉瘤多见20-30岁。
   - 软骨肉瘤：好发于40岁以上，尤其是中老年，常累及骨盆、股骨和肱骨；胫骨也可发生。
2. **转移性肿瘤**：由于患者年龄大（64岁），原发癌症转移到骨骼很常见。肺癌、乳腺癌等是常见来源。
   - 骨质破坏、软组织肿块符合转移灶特征。
3. **其他**：如感染（骨髓炎）或良性肿瘤，但疼痛仅2天，可能不是慢性过程；不过急性期可能快速进展。
   - 骨髓炎：通常有发热等全身症状，但病史中没提；影像上可能显示骨质破坏和软组织肿胀。

可能性排序：

- 最可能：转移性肿瘤（因为年龄大，常见）。
- 第二：原发恶性肿瘤如软骨肉瘤（胫骨近端）。
- 第三：感染或良性病变，但不太符合急性疼痛2天；不过需考虑。

具体疾病诊断：

1. 转移性骨肿瘤（例如来自乳腺癌、肺癌等）
2. 软骨肉瘤
3. 骨髓炎

检查影像细节：

- CT显示有钙化/骨化成分，这更符合软骨肉瘤或转移灶。
- MRI高信号：在T2加权像上，软骨肉瘤常有高信号；转移性肿瘤也可能。

根据标准知识：

- 软骨肉瘤：好发于40岁以上，50%发生在股骨、胫骨和肱骨。影像上可见钙化/骨化。
- 骨肉瘤：更年轻，但64岁罕见；可能有Codman三角等特征，但这里没看到明显。

在提供的CT图像中（第四张图），横断面显示病变内有高密度点（钙化），这支持软骨肉瘤或转移性肿瘤（如乳腺癌转移常有钙化）。

病史：无诱因疼痛2天。如果是急性，可能不是原发肿瘤的典型表现；但恶性肿瘤可以突然引起症状。

最可能诊断：

1. 转移性骨肿瘤（鉴于年龄和常见性）
2. 原发软骨肉瘤
3. 骨髓炎（但需排除）

临床建议：下一步诊疗。

- 建议行活检以明确病理。
- 进一步检查原发病灶（如全身CT、PET-CT）。
- 血液检查：肿瘤标志物等。

结构化回答：

1. 影像学描述：客观描述核心特征。
2. 诊断列表：3个疾病，按可能性排序。
3. 临床建议：具体步骤。

最终输出应只包含我的响应内容，不加额外信息。

影像学描述：
X线显示右胫骨近端（膝关节水平）存在溶骨性骨质破坏区，骨皮质中断、局部膨胀，伴软组织肿胀影；CT三维重建及横断面示胫骨近端骨质明显破坏（低密度区），病灶内可见斑片状钙化/骨化成分，周围软组织肿块形成，骨皮质不连续并穿孔；MRI冠状位及横断面T2加权像显示病灶呈高信号，累及骨髓腔并向周缘软组织浸润性扩展，边界不清。

结合病史（64岁女性，右膝无诱因疼痛2天）与影像特征：

1. **转移性骨肿瘤**（最可能）：年龄大、溶骨性破坏伴钙化/骨化及软组织肿块，符合常见原发恶性肿瘤（如乳腺癌、肺癌等）的骨转移表现。
2. **软骨肉瘤**（次可能）：好发于中老年，胫骨近端为常见部位；影像上病灶内钙化/骨化、浸润性生长及软组织侵犯与典型特征相符。
3. **急性骨髓炎**（较可能）：无诱因疼痛2天提示急性炎症过程；溶骨破坏和软组织肿胀可符合，但需排除慢性感染背景。

临床建议：

1. 立即行穿刺活检（CT引导下）明确病理诊断，并送肿瘤标志物检测；
2. 完善全身检查（包括胸部CT、PET-CT及血液学指标）以排查原发灶或转移范围；
3. 暂时避免负重，给予抗炎镇痛治疗并监测症状变化，待病理结果后制定靶向/放化疗方案。qwen3vl-32b-thinking · 431.605s (4.03 tok/s) · Mar 25, 8:25 PM
