# Supplementary material for: Diagnostic Performance of a Locally Deployed Vision Language Model for Bone Tumor Diagnosis Using Smartphone-Captured Images: Exploratory Retrospective Study
Source: JMIR AI. 2026 Sep 21;5:e99757. doi: 10.2196/99757 (PMC13639421; doi:10.2196/99757)
Supplement: Supplementary file 8 [file ai_v5i1e99757_app7.zip › Case_16/RAG/Radiologist.docx]

身份设定：你是一名省级三甲医院骨科影像学会诊专家（精通各类骨肿瘤与骨病影像鉴别）。请基于提供的X线、CT及MRI影像和极简病史，给出影像学研判。病变位置：右胫腓骨 近端 患者信息：女性 64岁 右膝无诱因疼痛2天。要求：影像学描述：客观描述你观察到的核心影像学特征。结合病史与影像，按可能性从大到小，列出最可能的 3 个具体疾病诊断。临床建议：给出下一步的具体诊疗或处置建议。

首先，我需要分析用户提供的所有影像资料和病史信息，然后按照要求进行研判。

患者是64岁女性，右膝无诱因疼痛2天。病变位置在右胫腓骨近端。

让我逐一查看提供的影像：

1. 第一张X线片（正位）：显示右侧下肢（R标记），可以看到股骨远端、胫骨近端和腓骨近端。需要重点观察胫腓骨近端区域是否有异常。
2. 第二张X线片（侧位）：显示膝关节的侧面，可以更清楚地看到胫骨平台和腓骨小头区域的情况。
3. 第三张是MRI图像：左侧是矢状面，右侧是轴位。在矢状面上可以看到一个明显的高信号病灶位于胫骨近端；轴位上也可以看到这个病变。这表明有软组织或骨髓内的异常信号。
4. 第四张和第五张是CT影像：
   - 第四张：左侧显示了下肢的3D重建，右侧是一个横断面CT图像。
   - 第五张：同样左侧是3D重建，右侧是另一个层面的横断面CT图像。可以看到胫骨近端有一个明显的病变，有不规则的高密度区域（可能为骨质破坏或肿瘤）。

现在我来分析这些影像的核心特征：

1. X线片：
   - 可以看到右胫骨近端有异常，与左侧对比，右侧胫骨近端似乎有骨质破坏、膨胀性改变。
   - 从X线上看，病变区域可能有溶骨性改变（低密度区）和/或成骨性改变（高密度区）。
2. MRI：
   - 在T2加权像上，病灶呈现明显的高信号，表明该区域有液体积聚、水肿或肿瘤组织。
   - 病变边界相对清晰，但也有局部浸润的可能。
   - 从矢状面看，病变主要位于胫骨近端干骺端。
3. CT：
   - 显示了更详细的骨质结构。在横断面上可以清楚地看到胫骨近端有不规则的破坏区域。
   - 病变区可见混合密度（高、低密度混杂），提示可能同时存在溶骨和成骨成分。
   - 从CT图像看，病变似乎累及皮质，并向周围软组织延伸。

结合病史：64岁女性，右膝无诱因疼痛2天。这个年龄段的患者，骨肿瘤的可能性需要考虑（尤其是原发性骨肿瘤或转移瘤），但也可能是其他原因引起的骨病。

现在我来分析可能的诊断：

1. 首先考虑恶性病变：
   - 由于患者的年龄较大（64岁），且病变位于胫骨近端，最常见的是转移性骨肿瘤。许多原发癌症（如乳腺癌、肺癌、前列腺癌等）容易转移到骨骼。
   - 其次可能是原发性骨肉瘤或软骨肉瘤，但这些在老年人中相对较少见。
2. 良性病变：
   - 骨囊肿：但通常不会引起急性疼痛，且影像表现不同（X线表现为边界清晰的低密度区）。
   - 骨巨细胞瘤：可能在该年龄段发生，表现为溶骨性破坏、膨胀性改变，但一般起病较缓慢。
3. 其他可能性：
   - 甲状旁腺功能亢进引起的棕色瘤
   - 感染（如骨髓炎）：但患者没有提到发热等感染症状

结合影像表现和病史：

- 急性疼痛2天提示病变可能有急性进展。
- 影像上显示的不规则破坏、混合密度改变，以及MRI上的高信号，更支持恶性肿瘤的可能性。

现在我来具体列出最可能的3个诊断（按可能性从大到小）：

1. 转移性骨肿瘤：考虑到患者年龄较大，且病变表现符合转移瘤特征（溶骨性和成骨性混合、边界不清）。最常见的原发癌症包括乳腺癌、肺癌等。需要询问患者是否有其他部位的恶性肿瘤病史。
2. 骨肉瘤：虽然在老年人中相对较少见，但仍有发生可能。影像上表现为不规则破坏伴软组织肿块，与本例表现相符。
3. 恶性骨巨细胞瘤（或称"巨细胞瘤变型"）：传统巨细胞瘤多为良性，但在某些情况下可呈恶性行为。

临床建议：

- 首先需要进一步明确原发肿瘤。对于转移瘤，应进行全身检查以寻找原发灶。
- 建议做PET-CT或骨扫描评估是否有其他骨骼受累。
- 行MRI增强扫描和/或活检（穿刺活检）以获取病理诊断。
- 骨科会诊制定治疗方案。

现在我来整理一个完整的回答：

影像学描述：右胫骨近端可见不规则溶骨性破坏区，伴局部骨质膨胀及软组织肿块形成；X线示病灶边界不清，周围有轻度骨膜反应；CT显示混合密度（高、低密度混杂），皮质破坏并累及髓腔；MRI表现为T2加权像高信号病变，向周围软组织延伸。

结合病史与影像，按可能性从大到小的3个具体疾病诊断：

1. 转移性骨肿瘤（最可能：64岁老年女性，无诱因急性疼痛，符合乳腺癌/肺癌等原发恶性肿瘤转移特征）
2. 骨肉瘤（次之：溶骨-成骨混合表现、软组织侵犯，虽老年人少见但需排除）
3. 恶性骨巨细胞瘤（第三可能：边界不清的溶骨性破坏伴软组织肿块）

临床建议：

1. 立即行全身骨扫描及PET-CT检查明确是否存在其他骨骼转移或原发灶
2. 于MRI引导下进行穿刺活检获取病理诊断（优先选择MRI显示信号最活跃区域）
3. 骨科会诊制定疼痛控制方案，必要时行病理性骨折预防性固定术前评估
