# Supplementary material for: Diagnostic Performance of a Locally Deployed Vision Language Model for Bone Tumor Diagnosis Using Smartphone-Captured Images: Exploratory Retrospective Study
Source: JMIR AI. 2026 Sep 21;5:e99757. doi: 10.2196/99757 (PMC13639421; doi:10.2196/99757)
Supplement: Supplementary file 8 [file ai_v5i1e99757_app7.zip › Case_17/Base/Ortho Oncologist.docx]

身份设定：你是一名省级三甲医院骨肿瘤科会诊专家（精通各类骨肿瘤与骨病影像鉴别）。请基于提供的X线、CT及MRI影像和极简病史，给出影像学研判。病变位置：左胫骨 患者信息：女性 6岁 左小腿无诱因疼痛6个月。要求：影像学描述：客观描述你观察到的核心影像学特征。结合病史与影像，按可能性从大到小，列出最可能的 3 个具体疾病诊断。临床建议：给出下一步的具体诊疗或处置建议。

首先，我需要分析用户提供的X线、CT和MRI影像以及病史信息。患者是6岁女性，左小腿无诱因疼痛6个月，病变位置在左胫骨。

从X线图像看（第一张和第二张图），左胫骨中段有明显的骨质破坏区域，表现为不规则的透亮区，并伴有骨膜反应（如Codman三角或日光射线征）。但具体细节需要更仔细观察。由于是6岁儿童，在这个年龄段常见的原发性恶性肿瘤包括尤文肉瘤和骨母细胞瘤等。

接下来看CT图像（第三张图），显示了横断面影像，可以看到胫骨内有不规则的溶骨性破坏，病灶中心密度稍高，周围可见软组织肿块。这种表现提示可能是恶性病变。

再看MRI图像（第四张图），在T1加权像上呈低信号，在T2加权像上呈混杂信号，并且有明显的强化，这支持了肿瘤的活性和血供丰富。同时可以看到病灶向周围软组织侵犯的情况。

结合患者的年龄（6岁）和症状（无诱因疼痛6个月），我需要考虑几种可能性：

1. 尤文肉瘤：这是儿童最常见的原发性骨恶性肿瘤之一，好发于长管状骨的干骺端。影像上常表现为溶骨性破坏、骨膜反应及软组织肿块。
2. 骨母细胞瘤：虽然多见于青少年，但6岁也有发生可能。它通常为良性，但可引起疼痛和局部肿胀。
3. 恶性纤维组织细胞瘤：相对少见，但在儿童中也可能出现。

需要排除其他可能性如骨髓炎（但病史无感染迹象）或转移癌（儿童原发肿瘤较少见）。因此，按可能性排序：

1. 尤文肉瘤
2. 骨母细胞瘤
3. 恶性纤维组织细胞瘤

临床建议方面，应该进行活检以明确病理诊断，并考虑进一步的全身检查如PET-CT来评估是否有转移。同时需要多学科会诊制定治疗方案。

影像学描述：
左胫骨中段可见不规则溶骨性破坏区，边界模糊，伴轻度骨膜反应及软组织肿块影（X线/CT）；MRI显示病灶T1WI呈低信号、T2WI混杂高信号，增强扫描明显强化，累及髓腔并突破皮质向周围软组织浸润。

最可能的3个具体疾病诊断：

1. 尤文肉瘤（儿童长骨恶性肿瘤首选考虑）
2. 骨母细胞瘤（良性但可引起疼痛的骨源性肿瘤）
3. 恶性纤维组织细胞瘤（少见，需排除其他恶性病变）

临床建议：

1. 立即行CT引导下穿刺活检明确病理分型；
2. 补充全身骨扫描及PET-CT评估转移情况；
3. 多学科会诊制定化疗+手术方案（尤文肉瘤需新辅助化疗）。
