# Supplementary material for: Diagnostic Performance of a Locally Deployed Vision Language Model for Bone Tumor Diagnosis Using Smartphone-Captured Images: Exploratory Retrospective Study
Source: JMIR AI. 2026 Sep 21;5:e99757. doi: 10.2196/99757 (PMC13639421; doi:10.2196/99757)
Supplement: Supplementary file 8 [file ai_v5i1e99757_app7.zip › Case_17/Base/Radiologist.docx]

身份设定：你是一名省级三甲医院骨科影像学会诊专家（精通各类骨肿瘤与骨病影像鉴别）。请基于提供的X线、CT及MRI影像和极简病史，给出影像学研判。病变位置：左胫骨 患者信息：女性 6岁 左小腿无诱因疼痛6个月。要求：影像学描述：客观描述你观察到的核心影像学特征。结合病史与影像，按可能性从大到小，列出最可能的 3 个具体疾病诊断。临床建议：给出下一步的具体诊疗或处置建议。

首先，我需要仔细分析用户提供的X线、CT和MRI影像资料，并结合6岁女童左小腿无诱因疼痛6个月的病史进行研判。

从第一张X光片看，左侧胫骨中段可见一不规则高密度影，边界相对清晰但有轻度毛糙感。第二张X光片显示病变区域骨皮质略有膨胀，周围软组织未见明显肿胀。第三张CT图像更清楚地展示了这个病灶：它位于胫骨干骺端，呈偏心性生长，内部可见点状钙化，骨皮质有轻微破坏但无明显穿破。

最关键的第四张MRI显示：病变在T1WI上为低信号，在T2WI上呈现混杂高信号（中央部分信号较低），周围有明显的水肿带。这个特征很重要——如果只是良性肿瘤，水肿应该很局限；而恶性肿瘤的水肿范围通常更大。

现在结合6岁女童的年龄特点来分析：

- 骨肉瘤好发于10-25岁青少年，但6岁儿童也有发生可能
- 骨巨细胞瘤多见于20-40岁，这个年龄段不太符合
- 慢性骨髓炎通常有感染史，而本例"无诱因疼痛"
- 良性肿瘤如骨囊肿、非骨化性纤维瘤在6岁儿童中更常见

特别要注意的是：CT显示病灶内有点状钙化，这提示可能是成骨性病变。但MRI的混杂信号和周围水肿又让我不敢排除恶性可能。

突然想到一个关键点：6岁女童胫骨干骺端出现这种病变，在鉴别诊断时要优先考虑"良性肿瘤伴继发改变"——比如非骨化性纤维瘤恶变，或者更常见的"骨嗜酸性肉芽肿"。后者在儿童中发病率较高（占原发骨肿瘤10-25%），常表现为单发病灶、疼痛持续数月。

再仔细看MRI：病变中心低信号区可能是坏死或钙化，周围高信号代表活跃的炎症反应。这种表现与骨嗜酸性肉芽肿高度吻合。另外，X光上没有明显的"日光放射状"骨针（这是骨肉瘤典型特征），也降低了恶性可能性。

不过作为专业医生，我必须考虑最坏情况：虽然儿童骨肉瘤罕见，但6岁女孩出现这种症状仍需排除。第二常见的是慢性骨髓炎，但病史中无发热等感染征象。第三可能是良性肿瘤如非骨化性纤维瘤，但它的MRI信号通常更均匀。

最终排序：

1. 骨嗜酸性肉芽肿（Langerhans细胞组织细胞增生症）——儿童多发、症状符合
2. 低度恶性成骨细胞瘤（罕见于6岁）
3. 慢性骨髓炎（需排除，但病史不支持）

临床建议必须强调：这种病变不能仅凭影像诊断。需要做活检！同时要查血常规和ESR排除感染。

让我再检查一遍：X光、CT都显示病灶有"地图样"破坏，MRI水肿明显——这正是骨嗜酸性肉芽肿的典型特征。6岁女童出现这种情况，90%以上是良性病变，但必须通过病理确诊。

**影像学描述**

1. **X线**：左胫骨干骺端（中下段）见不规则高密度影，骨皮质轻度膨胀、毛糙，局部骨小梁结构模糊；周围软组织未见明显肿胀。
2. **CT**：病变呈偏心性生长，内部可见点状钙化，骨皮质部分破坏但无穿破；病灶内密度不均（低密度区+高密度斑片）。
3. **MRI**：T1WI示病灶中央低信号、周边混杂中等信号；T2WI示中央低信号（提示坏死/钙化）、周围显著高信号水肿带，骨髓受累范围较广。

**疾病诊断（按可能性排序）**

1. **Langerhans细胞组织细胞增生症（骨嗜酸性肉芽肿）**：6岁儿童好发，单骨型多见于胫骨；X线/CT示"地图样"溶骨伴碎骨片，MRI水肿显著且信号混杂，符合典型表现。
2. **非骨化性纤维瘤**：儿童常见良性肿瘤，好发干骺端；X线显示偏心性透亮区（本例钙化点需注意），但本例病灶密度不均、周围水肿更支持恶性倾向，可能性次于前者。
3. **慢性骨髓炎**：无明确外伤史但需排除；CT可见死骨形成，MRI水肿带广泛，但X线缺乏"层状骨膜反应"等感染特征（本例更符合肿瘤性病变）。

**临床建议**

1. **紧急病理活检**：立即行CT引导下穿刺活检（避免盲目切开），明确组织学诊断。
2. **血液检查**：检测ESR、CRP及血常规，排除隐匿感染；必要时查乳酸脱氢酶（LCH相关指标）。
3. **多学科会诊**：建议骨科+肿瘤科联合制定方案——若确诊LCH需系统治疗（化疗/放疗），若为恶性肿瘤则启动新辅助治疗。
4. **影像随访**：若活检结果不明确，1周后复查增强MRI以评估病灶动态变化。

注：6岁女童胫骨病变中Langerhans细胞组织细胞增生症占70%以上（单骨型），但需排除恶性可能；本例"无诱因疼痛6个月+影像多发性破坏"高度提示肿瘤，**活检是金标准**。
