# Supplementary material for: Diagnostic Performance of a Locally Deployed Vision Language Model for Bone Tumor Diagnosis Using Smartphone-Captured Images: Exploratory Retrospective Study
Source: JMIR AI. 2026 Sep 21;5:e99757. doi: 10.2196/99757 (PMC13639421; doi:10.2196/99757)
Supplement: Supplementary file 8 [file ai_v5i1e99757_app7.zip › Case_17/RAG/Radiologist.docx]

身份设定：你是一名省级三甲医院骨科影像学会诊专家（精通各类骨肿瘤与骨病影像鉴别）。请基于提供的X线、CT及MRI影像和极简病史，给出影像学研判。病变位置：左胫骨 患者信息：女性 6岁 左小腿无诱因疼痛6个月。要求：影像学描述：客观描述你观察到的核心影像学特征。结合病史与影像，按可能性从大到小，列出最可能的 3 个具体疾病诊断。临床建议：给出下一步的具体诊疗或处置建议。

首先，我需要仔细分析用户提供的X线、CT及MRI影像资料，并结合病史（6岁女性，左小腿无诱因疼痛6个月）进行研判。

从图像中观察到的核心特征：

1. X线片显示：左胫骨近端（上段）存在一不规则高密度影，边界相对清晰但有轻微毛糙。周围软组织未见明显肿胀或异常钙化。
2. CT横断面图：病变区域呈混杂密度，以高密度为主，内部可见斑点状低密度区，骨皮质局部增厚、膨胀性改变，无明确骨膜反应。
3. MRI图像（T1WI和T2WI）：病灶在T1加权像上呈等信号/稍低信号，在T2加权像上呈现混杂高信号，中心有低信号区。周围软组织未见明显水肿或异常强化。

结合6岁儿童的年龄特点及无诱因长期疼痛的临床表现：

- 该年龄段常见骨病包括：良性肿瘤（如骨样骨瘤、非骨化性纤维瘤）、感染性疾病（如慢性骨髓炎）以及罕见的恶性肿瘤。
- 病变位于胫骨干，符合非骨化性纤维瘤好发部位；但需排除其他可能。

最可能的3个具体疾病诊断：

1. 非骨化性纤维瘤：6岁儿童多见于长管状骨（尤其胫骨），X线表现为边界清晰的透亮区伴硬化边，CT/MRI可见混杂密度/信号。本例虽有高密度影但可能为钙化灶。
2. 骨样骨瘤：典型" nidus "在X线上呈小圆形致密影（本例更接近此表现），MRI显示周围反应性水肿明显（本例未见显著软组织水肿）。
3. 慢性骨髓炎：儿童可因隐匿感染引起，但通常有发热等急性症状且影像上可见死骨、窦道形成。

临床建议：

- 优先进行病理活检明确诊断
- 建议完善血常规+CRP及ESR检测评估炎症反应
- 推荐行全身骨扫描排除多发性病变

需要特别注意：从提供的参考文献中，我看到关于"tendon"的描述（如CT显示钙化或骨化）以及"speckled intermediate SI filling defects"等特征。但本例是胫骨病变而非肌腱/滑膜问题，因此这些信息可能不直接相关。

影像学研判：
**影像学描述**：左胫骨干中上段可见一混杂密度病灶（X线及CT），以高密度为主伴斑点状低密度区；CT显示骨皮质局部增厚、膨胀性改变，无明确骨膜反应；MRI示T1WI等/稍低信号、T2WI混杂高信号伴中心低信号区，周围软组织未见显著水肿。

**最可能的3个诊断（从大到小）**：

1. **非骨化性纤维瘤**：6岁儿童胫骨干好发，X线呈边界清晰透亮区伴硬化边（本例高密度影考虑钙化灶），CT/MRI混杂密度/信号符合特征。
2. **慢性骨髓炎**：长期疼痛、病灶内死骨形成（低密度区）及骨皮质反应性增厚，但儿童隐匿感染需排除。
3. **骨样骨瘤**：典型"nidus"高密度影与本例类似，但MRI未见显著周围水肿（提示非急性期），可能性稍低。

**临床建议**：

1. 紧急行穿刺活检明确病理类型；
2. 完善血常规、CRP及ESR检测评估炎症指标；
3. 推荐全身骨扫描排查多发性病变（尤其非骨化性纤维瘤常为多灶性）；
4. 暂不建议手术，待病理结果后制定治疗方案。
