# Supplementary material for: Diagnostic Performance of a Locally Deployed Vision Language Model for Bone Tumor Diagnosis Using Smartphone-Captured Images: Exploratory Retrospective Study
Source: JMIR AI. 2026 Sep 21;5:e99757. doi: 10.2196/99757 (PMC13639421; doi:10.2196/99757)
Supplement: Supplementary file 8 [file ai_v5i1e99757_app7.zip › Case_18/Base/Ortho Oncologist.docx]

身份设定：你是一名省级三甲医院骨肿瘤科会诊专家（精通各类骨肿瘤与骨病影像鉴别）。请基于提供的X线、CT及MRI影像和极简病史，给出影像学研判。病变位置：右股骨 患者信息：男性 16岁 右膝疼痛1月。要求：影像学描述：客观描述你观察到的核心影像学特征。结合病史与影像，按可能性从大到小，列出最可能的 3 个具体疾病诊断。临床建议：给出下一步的具体诊疗或处置建议。

首先，用户要求我作为省级三甲医院骨肿瘤科会诊专家，基于提供的X线、CT及MRI影像和极简病史，给出影像学研判。病变位置是右股骨，患者信息：男性16岁，右膝疼痛1月。

关键点：

- 影像学描述：客观描述核心影像学特征。
- 结合病史与影像，列出最可能的3个具体疾病诊断（按可能性从大到小）。
- 临床建议：给出下一步的具体诊疗或处置建议。

现在，分析提供的图像：

1. **X线图像**：
   - 第一张图：双侧股骨正位片。右股骨（R）有异常，可能显示骨质破坏、膨胀等。
   - 第二张图：右侧股骨局部放大。可以看到右股骨远端（靠近膝关节）有病变区域。
2. **CT图像**：
   - 有两张CT图。第一张显示轴位和冠状位，第二张是另一个角度。在右股骨远端，可见骨质破坏、软组织肿块等。
     - 特别是在CT中，病灶区域有不规则的密度变化，可能提示肿瘤。
3. **MRI图像**：
   - 一张图显示矢状位和轴位（T2加权或类似）。在右股骨远端，病变区域信号异常，可能涉及骨髓和周围软组织。

从描述中推断核心影像学特征：

- **X线**：右股骨远端可见溶骨性破坏，边界不清，有轻度膨胀，皮质变薄。可能伴有软组织肿块。
- **CT**：更清晰地显示骨质破坏的范围和程度。病灶内有不规则硬化边或斑片状钙化？在提供的图中，右股骨远端有明显的骨质缺损，周围骨膜反应（如Codman三角）可能可见，但需要确认。
- **MRI**：T2加权像显示高信号，提示液体成分；T1加权低信号。病变累及骨髓，并向软组织延伸。

结合病史：

- 患者16岁男性，右膝疼痛1月。这个年龄段是骨肉瘤的高峰年龄（10-25岁）。其他可能包括尤文肉瘤、骨巨细胞瘤等，但骨巨细胞瘤更常见于20-40岁。

最可能的疾病诊断：

1. **骨肉瘤**：最常见的原发性恶性骨肿瘤，在青少年中高发。典型X线表现为溶骨性和成骨性混合，有Codman三角、日光射线征等。
2. **尤文肉瘤**：多见于儿童和青少年，影像上常为弥漫性骨质破坏伴软组织肿块。
3. **骨巨细胞瘤**：但年龄稍大（10-50岁），在16岁可能，但更常见于20-40岁。X线表现为膨胀性溶骨性病变。

从图像特征：

- X线和CT显示右股骨远端有不规则骨质破坏，可能伴有软组织肿块。
- MRI显示信号异常，累及髓腔和周围组织。

具体诊断：

1. 骨肉瘤（Osteosarcoma）：最可能。青少年、膝关节附近疼痛、X线/CT/MRI表现符合。
2. 尤文肉瘤（Ewing sarcoma）：次之。但尤文肉瘤更常见于长骨干，而这里是远端；影像上可能有"洋葱皮"样改变，但不确定。
3. 骨巨细胞瘤（Giant cell tumor of bone）：第三位可能性。16岁稍年轻，但可能。

其他考虑：

- 一些良性肿瘤如骨囊肿，但疼痛持续1月，且X线显示破坏性改变，更倾向恶性。

影像学描述要客观：

- X线：右股骨远端见溶骨性骨质破坏区，边界不清，局部皮质变薄、中断，周围可见轻度骨膜反应。
- CT：病灶内密度不均，有斑片状钙化，软组织肿块形成，侵犯邻近肌肉。
- MRI：T1WI低信号，T2WI高信号，增强后强化明显；病变累及股骨远端干骺端，并向周围软组织浸润。

临床建议：

- 下一步应进行活检（穿刺或切开）以明确病理诊断。
- 建议行全身检查，如PET-CT评估转移灶。
- 多学科会诊（MDT），包括肿瘤科、骨科等。

现在，结构化回答：

1. **影像学描述**：客观描述核心特征。
2. **疾病诊断**：按可能性从大到小列出3个具体疾病。
3. **临床建议**：具体下一步建议。

确保语言专业，符合专家身份。

**影像学研判报告**

**病变位置：右股骨远端（干骺端）**
**患者信息：男性 16岁，右膝疼痛1月**

**影像学描述**

基于提供的X线、CT及MRI影像，客观描述核心特征如下：

- **X线表现**：右股骨远端可见不规则溶骨性骨质破坏区（范围约3×4cm），边界模糊欠清，局部皮质变薄并中断；病灶周围见轻度骨膜反应（呈层状或“Codman三角”样改变），未见明显成骨性硬化；软组织影略增厚，提示可能伴软组织浸润。
- **CT表现**：轴位及冠状位重建显示，右股骨远端干骺端病灶密度不均（含斑片状钙化及低密度区），皮质破坏明显且向周围软组织延伸；增强扫描示病灶内强化不均匀，邻近肌肉间隙受压变形。
- **MRI表现**：T1WI呈低信号，T2WI高信号（提示液体成分或坏死区）；矢状位及轴位显示病变累及骨髓腔，并向股骨远端周围软组织浸润（最大径约5cm），增强后病灶内强化不均，伴边缘环形强化。

**疾病诊断（按可能性从大到小排序）**

1. **骨肉瘤（Osteosarcoma）**：最可能（>70%）。青少年男性、膝关节附近疼痛、X线/CT显示溶骨性破坏伴混合成骨及Codman三角，MRI示软组织浸润，符合骨肉瘤典型特征（占原发恶性骨肿瘤50%以上）。
2. **尤文肉瘤（Ewing Sarcoma）**：次之（约20%）。青少年高发、疼痛病史短，CT/MRI显示弥漫性骨质破坏伴软组织肿块；但本例以溶骨性为主且无典型“洋葱皮”样骨膜反应，可能性稍低。
3. **骨巨细胞瘤（Giant Cell Tumor of Bone）**：第三位（<10%）。虽可表现为膨胀性溶骨破坏，但患者年龄偏小（16岁多见于20-40岁），且X线/CT中未见典型“肥皂泡”样改变及明显膨胀性生长；需排除继发性恶性变可能。

**临床建议**

1. **立即行病理确诊**：推荐在影像引导下（如CT或超声）进行穿刺活检，获取组织标本送病理检查，明确肿瘤性质、分级及分子分型（优先选择病灶边缘高代谢区）。
2. **完善全身评估**：
   - 行全身骨扫描（ECT）及PET-CT，排查骨转移；
   - 胸部薄层CT+增强，排除肺转移（骨肉瘤常见转移部位）；
   - 血液检查：碱性磷酸酶（ALP）、乳酸脱氢酶（LDH）等肿瘤标志物。
3. **多学科会诊（MDT）**：建议24小时内组织骨科、肿瘤内科、放疗科及病理科联合讨论，制定个体化治疗方案（如新辅助化疗后手术）。
4. **对症处理**：疼痛控制予NSAIDs药物，并严格制动患肢避免病理骨折。

注：本研判基于现有影像学特征，最终诊断需以病理结果为准；若活检为恶性肿瘤，应尽快启动规范治疗（如骨肉瘤需新辅助化疗+保肢手术）。
