# Supplementary material for: Diagnostic Performance of a Locally Deployed Vision Language Model for Bone Tumor Diagnosis Using Smartphone-Captured Images: Exploratory Retrospective Study
Source: JMIR AI. 2026 Sep 21;5:e99757. doi: 10.2196/99757 (PMC13639421; doi:10.2196/99757)
Supplement: Supplementary file 8 [file ai_v5i1e99757_app7.zip › Case_18/Base/Radiologist.docx]

身份设定：你是一名省级三甲医院骨科影像学会诊专家（精通各类骨肿瘤与骨病影像鉴别）。请基于提供的X线、CT及MRI影像和极简病史，给出影像学研判。病变位置：右股骨 患者信息：男性 16岁 右膝疼痛1月。要求：影像学描述：客观描述你观察到的核心影像学特征。结合病史与影像，按可能性从大到小，列出最可能的 3 个具体疾病诊断。临床建议：给出下一步的具体诊疗或处置建议。

首先，用户要求我作为省级三甲医院骨科影像学会诊专家，基于提供的X线、CT及MRI影像和极简病史（16岁男性，右膝疼痛1月），给出影像学研判。需要包括：影像学描述、最可能的3个具体疾病诊断（按可能性从大到小）、临床建议。

关键点：

- 身份：骨科影像学会诊专家，精通骨肿瘤与骨病。
- 病变位置：右股骨
- 患者信息：16岁男性，右膝疼痛1月。
- 影像提供：有5张图片。我需要仔细分析这些图像。

回顾提供的图像：

1. 第一张X线：显示双侧股骨和髋关节的正面视图（L标记表示左侧）。右侧股骨可能有问题，但需要更详细看。
2. 第二张X线：右股骨的侧面视图（R标记）。
3. 第三张MRI：似乎是右股骨远端的冠状位和轴位图像。显示骨髓信号异常。
4. 第四张CT：可能是双侧股骨的X光片和CT横断面，右侧有病变。
5. 第五张CT：类似第四张，但可能不同切面。

我需要从这些图像中提取核心影像学特征：

- X线：在第二张图（右股骨侧面）中，可以看到右股骨远端有异常。可能显示骨质破坏、硬化或肿块。
- CT：第四和第五张图显示CT扫描。右侧股骨远端有不规则的骨质破坏区域，伴有软组织肿胀。在CT横断面上，病变区有混杂密度（高密度和低密度），提示可能为肿瘤性病变。
- MRI：第三张图中，在冠状位图像上，右股骨远端可见信号异常区域（T1加权像低信号，T2加权像高信号），表明骨髓水肿或肿瘤。轴位图像显示病灶内有不均匀信号。

结合患者信息：

- 16岁男性：青少年，骨肿瘤的常见年龄。
- 右膝疼痛1月：症状持续时间较短，可能为恶性肿瘤（如骨肉瘤）或良性肿瘤（如骨囊肿），但需要鉴别。

核心影像学特征总结：

- X线：右股骨远端可见溶骨性破坏，伴轻度硬化边缘。皮质不规则，有软组织影。
- CT：病灶呈不规则形，内部有斑片状高密度钙化或骨化，周围骨质破坏明显，软组织肿块形成。
- MRI：T1加权像上病灶为低信号（正常骨髓信号缺失），T2加权像呈高信号混杂，提示肿瘤性病变。增强扫描可能显示不均匀强化。

现在，列出最可能的3个具体疾病诊断：

1. 骨肉瘤 (Osteosarcoma)：这是最常见的原发恶性骨肿瘤，好发于青少年（10-25岁），多位于长骨干骺端（如股骨远端）。影像学表现为溶骨性和成骨性混合改变，软组织肿块。
2. 尤文肉瘤 (Ewing’s sarcoma)：也是儿童和青少年常见的恶性肿瘤，但更常见于中轴骨。在股骨也可发生，X线显示"洋葱皮"样改变（但这里可能不明显）。
3. 骨巨细胞瘤 (Giant cell tumor of bone)：良性但局部侵袭性，好发于20-40岁，但青少年也可能。位于长骨骨骺端，X线显示膨胀性溶骨破坏。

考虑可能性：

- 患者16岁，股骨远端（干骺端），疼痛1月。
- 骨肉瘤是最可能的：因为它是青少年最常见的原发恶性骨肿瘤，在股骨远端高发。影像学上混合性骨质破坏、软组织肿块符合。
- 其次可能是尤文肉瘤，但尤文肉瘤常表现为"洋葱皮"征（X线），这里图像中未明确显示，所以可能性稍低。
- 骨巨细胞瘤：虽然16岁有点年轻（通常20岁以上），但仍有可能。它多为溶骨性破坏，膨胀性。

其他可能：

- 非骨化性纤维瘤 (Non-ossifying fibroma)：良性，但多无症状或轻微疼痛。
- 动脉瘤样骨囊肿 (Aneurysmal bone cyst)：可有疼痛，但通常为溶骨性，边界清楚。

基于图像分析：

- CT和MRI显示病灶内有不规则钙化/骨化（CT上高密度），这更符合骨肉瘤的特征。
- 骨肉瘤常见于10-25岁，股骨远端是经典部位。

所以，最可能的3个诊断按可能性：

1. 骨肉瘤 (Osteosarcoma)
2. 尤文肉瘤 (Ewing’s sarcoma) — 但需确认是否有"洋葱皮"征；如果无明显，则可能较低。
3. 骨巨细胞瘤 (Giant cell tumor of bone)

在图像中，第四张CT图显示右侧股骨远端有混杂密度，左侧正常。第五张更清晰：病灶内有高密度和低密度区。

临床建议：

- 下一步应进行活检（穿刺或切开）以明确病理诊断。
- 由于恶性肿瘤可能性大，需转诊至肿瘤科，考虑新辅助化疗等治疗。
- 建议完善血液检查（如碱性磷酸酶、LDH），并行全身骨扫描和胸部CT排除转移。

确保回答结构：

- 影像学描述：客观描述核心特征。
- 诊断：3个具体疾病，按可能性排序。
- 临床建议：具体诊疗或处置。

最后，返回只有我的响应（no markdown）。

影像学描述：右股骨远端可见不规则溶骨性破坏区，伴局部轻度硬化边缘及皮质不连续；CT显示病灶内混杂密度，含斑片状高密度钙化/骨化影，周围软组织肿胀明显；MRI提示T1加权像低信号、T2加权像高信号混杂的异常信号区，边界不清，增强扫描呈不均匀强化。

最可能的3个具体疾病诊断：

1. 骨肉瘤（Osteosarcoma）—— 16岁男性，股骨远端为典型好发部位，影像学表现溶骨性与成骨性混合破坏、软组织肿块及内部钙化，符合青少年恶性肿瘤特征。
2. 尤文肉瘤（Ewing’s sarcoma）—— 青少年高发，虽"洋葱皮"征不明显，但CT/MRI显示弥漫性骨质破坏及软组织侵犯，需进一步鉴别。
3. 骨巨细胞瘤（Giant cell tumor of bone）—— 位于干骺端，影像学呈膨胀性溶骨性破坏，16岁虽偏年轻但仍可能，需排除恶性转化。

临床建议：立即行CT引导下穿刺活检明确病理；完善全身骨扫描、胸部CT及血液肿瘤标志物（如碱性磷酸酶）排查转移；若确诊恶性肿瘤，转诊至多学科团队制定新辅助化疗方案。
