# Supplementary material for: Diagnostic Performance of a Locally Deployed Vision Language Model for Bone Tumor Diagnosis Using Smartphone-Captured Images: Exploratory Retrospective Study
Source: JMIR AI. 2026 Sep 21;5:e99757. doi: 10.2196/99757 (PMC13639421; doi:10.2196/99757)
Supplement: Supplementary file 8 [file ai_v5i1e99757_app7.zip › Case_19/Base/Ortho Oncologist.docx]

身份设定：你是一名省级三甲医院骨肿瘤科会诊专家（精通各类骨肿瘤与骨病影像鉴别）。请基于提供的X线、CT及MRI影像和极简病史，给出影像学研判。病变位置：左股骨 患者信息：男性 19岁 左大腿无诱因疼痛3个月。要求：影像学描述：客观描述你观察到的核心影像学特征。结合病史与影像，按可能性从大到小，列出最可能的 3 个具体疾病诊断。临床建议：给出下一步的具体诊疗或处置建议。

首先，用户要求我作为省级三甲医院骨肿瘤科会诊专家，基于提供的X线、CT及MRI影像和极简病史，给出影像学研判。病变位置是左股骨，患者信息：男性19岁，左大腿无诱因疼痛3个月。

我的响应需要包括三个部分：

1. 影像学描述：客观描述观察到的核心影像学特征。
2. 结合病史与影像，按可能性从大到小，列出最可能的3个具体疾病诊断。
3. 临床建议：给出下一步的具体诊疗或处置建议。

现在，分析提供的影像：

- 第一张图是X线片（可能是前后位），显示左股骨。有"L"标记表示左侧。我看到股骨干有一个区域似乎有异常，比如骨质破坏或肿块。从图像中看，股骨远端附近有模糊的区域，可能指示病变。
- 第二张图也是X线片（侧位？），显示膝关节和股骨。同样，左股骨上有不规则区域。
- 第三张图是CT扫描：左侧显示双下肢对比（可能是冠状面或矢状面），右侧有轴向切片。在左侧图像中，红色虚线标记了病变位置；在右侧的CT切片上，可以看到股骨内有一个高密度区域，周围可能有软组织肿胀。
- 第四张图是另一个CT扫描：类似第三张，但显示不同的切片或序列。轴向切片显示股骨内的病变，看起来更清晰。
- 第五张图是MRI：左侧是矢状面或冠状面，右侧是轴向T1/T2加权图像。在轴向图像中，股骨内有一个高信号区域（可能表示水肿或肿瘤），周围有软组织受累。

关键影像学特征：

- 从X线：左股骨骨干有溶骨性破坏，边界不清，可能伴有骨膜反应（但不明显）。
- 从CT：病变位于股骨中段，显示骨质破坏、皮质中断，内部有不规则的高密度影（可能是肿瘤基质或钙化），周围软组织肿块。
- 从MRI：T2加权像上病变为高信号，提示富含液体成分；T1加权可能低信号。病变侵犯骨髓和软组织，显示明显的软组织肿胀。

结合病史：

- 患者19岁男性，无诱因左大腿疼痛3个月。
- 年龄：19岁是骨肉瘤的高发年龄（10-25岁）。
- 症状：无诱因疼痛，逐渐加重，符合恶性肿瘤的表现。

可能的诊断：

1. 骨肉瘤 (Osteosarcoma)：最常见于青少年和年轻成人，好发于长骨干骺端。影像上表现为溶骨性破坏、Codman三角、日光放射状骨针等。CT显示骨质破坏和软组织肿块；MRI显示侵犯范围。
2. 尤文肉瘤 (Ewing sarcoma)：也常见于青少年，但更倾向于骨干。影像上类似，但可能有更明显的骨膜反应（洋葱皮样）。
3. 骨巨细胞瘤 (Giant cell tumor of bone)：好发于20-40岁，多在骨端。但患者19岁，也可能；影像上为膨胀性溶骨性病变。

按可能性排序：

- 由于患者年龄19岁，无诱因疼痛3个月，左股骨病变（干骺端或骨干？从图像看，似乎更靠近骨干），最可能的是骨肉瘤。
- 其次是尤文肉瘤。
- 骨巨细胞瘤也可能，但通常在20岁以上。

确认影像特征：

- X线：溶骨性破坏，边缘不清，无明显Codman三角（但需仔细看）。
- CT：显示骨质破坏、软组织肿块；高密度影提示肿瘤基质。
- MRI：T1低信号、T2高信号，符合恶性肿瘤。

列出3个最可能的诊断：

1. 骨肉瘤 (Osteosarcoma)
2. 尤文肉瘤 (Ewing sarcoma)
3. 恶性纤维组织细胞瘤 (Malignant fibrous histiocytoma) 或其他，但骨巨细胞瘤可能性较低；或许考虑转移癌？但19岁无原发肿瘤史。

更准确：

1. 骨肉瘤
2. 尤文肉瘤
3. 软骨肉瘤 (Chondrosarcoma) — 但软骨肉瘤更常见于成人，且好发于骨盆；在股骨也可能。

标准排序：对于青少年股骨病变：

1. 骨肉瘤
2. 尤文肉瘤
3. 恶性淋巴瘤或转移癌（罕见）

但患者无其他症状，所以优先考虑原发骨肿瘤。

临床建议：

- 下一步应进行活检以明确诊断。
- 建议：经皮穿刺活检或开放活检；同时检查全身情况（如PET-CT、X线胸片）排除转移。
- 转诊至骨肿瘤专科治疗，包括化疗等多学科诊疗。

现在，结构化响应：

1. 影像学描述：客观描述核心特征。基于所有影像。
2. 诊断列表：3个具体疾病，按可能性排序。
3. 临床建议：具体步骤。

确保语言专业、简洁。

**影像学描述**

基于提供的X线、CT及MRI影像（左股骨），观察到以下核心影像学特征：

- **X线片**：左股骨干中段可见不规则溶骨性破坏区，边界模糊，局部皮质中断；无明显Codman三角或日光放射状骨针，但病变区域周围有轻度软组织肿胀影。
- **CT扫描**：轴向切面显示病变为偏心性、多房性骨质破坏灶（直径约3.5 cm），内部见斑片状高密度钙化/骨化成分；皮质局部缺损，周围软组织呈浸润性肿块（增强后轻度强化）；未见明显骨膜反应。
- **MRI扫描**：T1加权像示病变为低信号，T2加权像呈不均匀高信号（提示富含液体成分），病变向髓腔内扩展并累及周围软组织；脂肪抑制序列显示病变边缘不清，伴邻近肌肉水肿信号，符合恶性肿瘤浸润特征。
  综上，影像表现为**溶骨性骨质破坏、皮质中断、软组织肿块形成及多模态信号不均**，提示侵袭性骨源性疾病。

**诊断可能性排序（结合19岁男性、无诱因疼痛3个月）**

1. **骨肉瘤 (Osteosarcoma)**：最符合。患者为青少年高发年龄段（10-25岁），股骨干中段溶骨性破坏伴软组织肿块及钙化，MRI信号不均支持肿瘤基质形成；X线/CT无典型Codman三角可能因早期病变所致。
2. **尤文肉瘤 (Ewing Sarcoma)**：次之。青少年常见原发恶性骨肿瘤，影像表现类似（溶骨性破坏、软组织肿块），但CT/MRI中钙化较少见；本例高密度影更倾向骨肉瘤，需活检鉴别。
3. **骨巨细胞瘤 (Giant Cell Tumor of Bone)**：相对可能性低。虽好发于20-40岁，但19岁患者可发生；影像表现为膨胀性溶骨、皮质变薄（本例更偏侵袭），且无典型“肥皂泡”样改变，需排除恶性转化。

**临床建议**

1. **立即行病理活检**：推荐在CT引导下经皮穿刺活检（避开大血管神经），获取组织标本进行免疫组化及分子检测以明确诊断；若穿刺失败，则开放手术活检。
2. **全身分期评估**：
   - 完善胸部高分辨CT（排除肺转移）；
   - 全身骨扫描或PET-CT（评估远处骨转移）。
3. **多学科会诊**：转至骨肿瘤专科，制定个体化方案（如确诊为骨肉瘤，则需新辅助化疗+手术切除+辅助化疗）；同时监测血清碱性磷酸酶及乳酸脱氢酶水平。
4. **患者管理**：避免剧烈活动以防病理性骨折，疼痛控制以非甾体抗炎药为主，并安排3日内复诊。
