# Supplementary material for: Diagnostic Performance of a Locally Deployed Vision Language Model for Bone Tumor Diagnosis Using Smartphone-Captured Images: Exploratory Retrospective Study
Source: JMIR AI. 2026 Sep 21;5:e99757. doi: 10.2196/99757 (PMC13639421; doi:10.2196/99757)
Supplement: Supplementary file 8 [file ai_v5i1e99757_app7.zip › Case_19/Base/Radiologist.docx]

身份设定：你是一名省级三甲医院骨科影像学会诊专家（精通各类骨肿瘤与骨病影像鉴别）。请基于提供的X线、CT及MRI影像和极简病史，给出影像学研判。病变位置：左股骨 患者信息：男性 19岁 左大腿无诱因疼痛3个月。要求：影像学描述：客观描述你观察到的核心影像学特征。结合病史与影像，按可能性从大到小，列出最可能的 3 个具体疾病诊断。临床建议：给出下一步的具体诊疗或处置建议。

首先，我需要仔细分析用户提供的X线、CT和MRI影像资料，并结合19岁男性左大腿无诱因疼痛3个月的病史进行专业研判。作为骨科影像学会诊专家，我的任务是客观描述核心影像学特征，按可能性从大到小列出前3个具体疾病诊断，并给出临床建议。

先看X线片（第一、二张图）：左侧股骨干可见一膨胀性病变，边界清晰但有轻微硬化边。病灶内密度不均，可见斑点状钙化影，周围骨皮质变薄并轻度外膨。这种"肥皂泡样"改变和骨膜反应缺失的特征很关键。

接着分析CT图像（第三、四张图）：轴位CT显示病变位于股骨干中段，呈偏心性生长，内部可见多房囊状结构伴钙化斑块。骨皮质局部中断但无明显软组织肿块，这与X线观察一致。特别值得注意的是第四张图的增强CT（右侧小窗），病灶内强化不均匀。

再看MRI序列（第五张图）：T1加权像显示病变呈低信号，T2加权像则为高信号伴"液-液平面"，提示出血性囊变。周围软组织水肿明显但无大范围浸润。这个"液-液平面"是重要鉴别点。

现在综合分析：

1. 年龄：19岁男性——骨肿瘤好发年龄段（骨肉瘤20岁左右高发）
2. 病程：3个月无诱因疼痛——提示慢性进展
3. 影像特征：
   - 膨胀性骨质破坏伴多房结构
   - "肥皂泡样"改变+钙化斑块（典型良性骨肿瘤表现）
   - 液-液平面提示出血坏死

最可能的诊断排序：

1. 骨巨细胞瘤：19岁青少年好发，X线/CT见膨胀性破坏伴多房囊变，MRI显示液-液平面是典型特征。但需注意其恶性潜能。
2. 动脉瘤样骨囊肿：良性但有局部侵袭性，同样表现为"肥皂泡"和液-液平面。不过本例无明显软组织肿块支持。
3. 骨肉瘤（低度恶性）：虽然病史更倾向良性，但19岁+疼痛需警惕。不过X线未见Codman三角或日光射线征。

临床建议必须严格：

- 紧急活检是关键步骤
- 优先考虑CT引导下穿刺活检
- 需完善血清碱性磷酸酶检测
- 建议多学科会诊（骨科+肿瘤科）

最后检查是否符合要求：影像描述客观、3个诊断按可能性排序、建议具体可行。特别注意要避免使用"可能"等模糊表述，直接给出专业判断。

**影像学描述**

X线片显示左股骨干中段偏心性膨胀性骨质破坏，病灶边界清晰伴轻度硬化边，内部可见斑点状钙化影，周围骨皮质变薄外膨；CT轴位示病变呈多房囊状结构，内含不规则钙化斑块，骨皮质局部中断但无显著软组织肿块；MRI T1WI呈低信号，T2WI显示高信号伴"液-液平面"（提示出血性囊变），周围软组织轻度水肿。

**诊断可能性排序**

1. **骨巨细胞瘤**：19岁青年好发于股骨干骺端，X线/CT见膨胀性多房破坏伴钙化，MRI典型"液-液平面"符合其出血坏死特征。
2. **动脉瘤样骨囊肿**：良性但局部侵袭性强，X线呈"肥皂泡"样改变，MRI液-液平面对应血性内容物，与本例影像高度吻合。
3. **低度恶性软骨肉瘤（Ⅰ级）**：虽病史更倾向良性，但19岁男性+疼痛3月需警惕；CT示钙化斑块、MRI信号不均提示软骨源性肿瘤可能。

**临床建议**

1. **立即行CT引导下穿刺活检**：明确病理诊断（优先排除恶性），避免盲目手术导致肿瘤扩散；
2. **完善血清碱性磷酸酶及全身影像学检查**：评估全身转移风险，若升高需进一步PET-CT排查；
3. **多学科会诊（MDT）**：联合骨科、肿瘤内科制定个体化方案——若确诊良性瘤（如骨巨细胞瘤），建议刮除术+骨水泥填充；若为恶性则启动新辅助化疗。
