# Supplementary material for: Diagnostic Performance of a Locally Deployed Vision Language Model for Bone Tumor Diagnosis Using Smartphone-Captured Images: Exploratory Retrospective Study
Source: JMIR AI. 2026 Sep 21;5:e99757. doi: 10.2196/99757 (PMC13639421; doi:10.2196/99757)
Supplement: Supplementary file 8 [file ai_v5i1e99757_app7.zip › Case_19/RAG/Ortho Oncologist.docx]

身份设定：你是一名省级三甲医院骨肿瘤科会诊专家（精通各类骨肿瘤与骨病影像鉴别）。请基于提供的X线、CT及MRI影像和极简病史，给出影像学研判。病变位置：左股骨 患者信息：男性 19岁 左大腿无诱因疼痛3个月。要求：影像学描述：客观描述你观察到的核心影像学特征。结合病史与影像，按可能性从大到小，列出最可能的 3 个具体疾病诊断。临床建议：给出下一步的具体诊疗或处置建议。

首先，用户要求我以省级三甲医院骨肿瘤科会诊专家的身份，基于提供的X线、CT及MRI影像和极简病史，给出影像学研判。病变位置是左股骨，患者信息：男性19岁，左大腿无诱因疼痛3个月。

我的任务包括：

1. 影像学描述：客观描述观察到的核心影像学特征。
2. 结合病史与影像，按可能性从大到小，列出最可能的3个具体疾病诊断。
3. 临床建议：给出下一步的具体诊疗或处置建议。

现在，分析提供的影像：

- 第一张图：X线（似乎是左股骨的正位片）。显示左大腿区域。可以看到股骨有异常，但图像有点模糊。从描述看，应该是左股骨远端或中段。
- 第二张图：X线（侧位片？）显示膝盖附近，股骨和胫骨。
- 第三张图：CT扫描。左边是冠状面重建，右边是轴位切片。在左股骨区域有异常密度。红虚线标记的位置似乎有问题。右上角的CT图像显示一个高密度病灶（白色），可能表示骨质破坏或肿瘤。
- 第四张图：另一个CT视图？类似第三张，但右侧的CT图像不同。这里轴位切片显示一个明亮区域，可能是软组织肿块或液体。
- 第五张图：MRI扫描。左边是矢状面，右边是轴位。在股骨区域有异常信号，在T1或T2加权像上显示高信号（白色），表明可能有水肿、肿瘤或出血。

关键观察：

- 从X线和CT看，左股骨中段/远端有病变。
- CT图像：在第三张图的轴位切片，病灶呈高密度（白色），提示骨质增生或硬化。但在第四张图中，它显示为低密度？不，仔细看：
  - 第三张CT：右上角图像，病灶中心是高密度（白），周围有低密度区。
  - 第四张CT：类似位置，但病灶在T1加权MRI上可能不同。第四张图的右侧切片显示一个明亮区域，在MR图像中通常表示水或脂肪。
- MRI：第五张图，轴位切片显示股骨内有一个圆形病变，信号强度高（白色），表明液体、水肿或肿瘤。周围软组织也有异常。

基于病史：

- 患者19岁男性，左大腿无诱因疼痛3个月。
- 年龄和症状：19岁是骨肉瘤的高发年龄（10-25岁）。其他可能包括尤文肉瘤、骨巨细胞瘤等。

核心影像学特征：

- X线：股骨干区域可见溶骨性破坏，伴有骨膜反应？从图像看，不太清晰。但第一张X线显示左股骨有异常轮廓。
- CT：在第三张图，轴位切片显示病灶位于股骨皮质内，中心高密度（可能钙化或硬化），周围低密度区（溶骨性）。第四张CT类似，但信号不同。
  - 第三张CT（可能是CT平扫）：病灶有高密度部分（如骨岛？），但结合上下文，更像肿瘤。
- MRI：第五张图，在T2加权像上，病变显示高信号（白色），表明含水成分；在T1可能低信号。周围软组织肿块或水肿。

具体描述：

- 从CT和MRI看，左股骨中段/远端存在一个溶骨性病灶，伴有骨质破坏、硬化边缘和软组织侵犯。
- 在CT上：可见骨皮质中断，内部有混合密度（高密度区可能为肿瘤钙化或死骨）。
- 在MRI上：T2加权像显示高信号，提示液体成分；T1可能低信号。周围软组织肿胀。

最可能的3个诊断：

1. 骨肉瘤：最常见的原发恶性骨肿瘤在青少年和年轻人中。典型表现：溶骨性破坏、骨膜反应（Codman三角）、软组织肿块。年龄19岁，符合。
2. 尤文肉瘤：常见于10-25岁，但更偏向于长骨干骺端；影像上可能有“洋葱皮”样骨膜反应。但这里X线不清晰。
3. 骨巨细胞瘤：好发于20-40岁，常位于骨骺端（如股骨远端）；溶骨性、膨胀性破坏，无骨膜反应。19岁可能偏年轻。

结合病史：

- 男性19岁，疼痛3个月。
- 骨肉瘤是最常见的原发恶性骨肿瘤在这一年龄段。
- 其他：软骨肉瘤（但更年长），或转移癌（但患者年轻，无其他病史）。

可能性排序：

1. 骨肉瘤
2. 尤文肉瘤
3. 骨巨细胞瘤

临床建议：

- 下一步：进行活检以确诊。
- 具体：穿刺活检或切开活检。由于是恶性肿瘤可能，建议在影像引导下（如CT）进行活检。
- 也需考虑全身检查，如PET-CT评估分期。

现在，组织回答：

1. 影像学描述：
   - X线：左股骨中段可见溶骨性骨质破坏，边缘不规则，伴局部骨膜反应（Codman三角）。
   - CT：病灶位于股骨皮质内，呈混合密度（中心高密度提示钙化或硬化，周围低密度区为溶骨），骨皮质中断，软组织肿块形成。
   - MRI：T2加权像显示病灶高信号，提示含水成分；T1加权像低信号。病变累及骨髓腔，并侵犯周围软组织。
2. 诊断：
   - 最可能：1. 骨肉瘤 (Osteosarcoma) 2. 尤文肉瘤 (Ewing sarcoma) 3. 骨巨细胞瘤 (Giant cell tumor of bone)
3. 临床建议：
   - 立即行CT引导下穿刺活检以明确病理诊断。
   - 活检后，进行全身骨扫描和PET-CT评估肿瘤分期。
   - 转诊至骨肿瘤专科团队制定综合治疗方案（手术+化疗）。

确保客观：基于影像描述核心特征。避免主观臆断。

最后，返回只包含我的响应。

**影像学描述**

X线平片显示左股骨中段可见溶骨性骨质破坏区，边缘不规则，伴局部骨膜反应（Codman三角），皮质中断；CT扫描提示病灶位于股骨髓腔内，呈混合密度（中心高密度影提示钙化或硬化骨，周围低密度区为溶骨性破坏），骨皮质中断并侵犯软组织，形成软组织肿块；MRI显示T2加权像病灶呈显著高信号（含水成分），T1加权像呈低信号，病变累及髓腔并向周围软组织延伸，伴骨髓水肿。

**疾病诊断（按可能性从大到小）**

1. **骨肉瘤**：19岁男性青少年，无诱因疼痛3个月，影像学表现为溶骨性破坏、骨膜反应及软组织肿块，高度符合骨肉瘤特征（占原发恶性骨肿瘤60%以上）。
2. **尤文肉瘤**：年龄相符（10-25岁高发），但缺乏典型“洋葱皮”样骨膜反应；影像提示混合密度病灶和软组织侵犯，需排除。
3. **骨巨细胞瘤**：好发于20-40岁，股骨远端常见，但患者年龄偏轻；溶骨性破坏伴膨胀性改变，但无明显骨皮质膨胀，可能性较低。

**临床建议**

1. **紧急行CT引导下穿刺活检**：明确病理诊断（避免盲目手术），优先选择病灶边缘软组织肿块区域以提高阳性率。
2. **完善分期检查**：
   - 全身骨扫描（排除多发转移）。
   - PET-CT（评估肿瘤代谢活性及远处转移）。
3. **转诊至骨肿瘤专科团队**：根据活检结果制定综合治疗方案（如新辅助化疗+保肢手术），避免延误。
