# Supplementary material for: Diagnostic Performance of a Locally Deployed Vision Language Model for Bone Tumor Diagnosis Using Smartphone-Captured Images: Exploratory Retrospective Study
Source: JMIR AI. 2026 Sep 21;5:e99757. doi: 10.2196/99757 (PMC13639421; doi:10.2196/99757)
Supplement: Supplementary file 8 [file ai_v5i1e99757_app7.zip › Case_19/RAG/Radiologist.docx]

身份设定：你是一名省级三甲医院骨科影像学会诊专家（精通各类骨肿瘤与骨病影像鉴别）。请基于提供的X线、CT及MRI影像和极简病史，给出影像学研判。病变位置：左股骨 患者信息：男性 19岁 左大腿无诱因疼痛3个月。要求：影像学描述：客观描述你观察到的核心影像学特征。结合病史与影像，按可能性从大到小，列出最可能的 3 个具体疾病诊断。临床建议：给出下一步的具体诊疗或处置建议。

首先，用户要求我作为省级三甲医院骨科影像学会诊专家，基于提供的X线、CT及MRI影像和极简病史进行研判。病变位置是左股骨，患者是19岁男性，无诱因疼痛3个月。

关键点：

- **身份设定**：我是骨科影像学专家，精通骨肿瘤与骨病的影像鉴别。
- **要求**：
  - 影像学描述：客观描述核心影像学特征。
  - 结合病史与影像，列出最可能的3个具体疾病诊断（按可能性从大到小）。
  - 临床建议：给出下一步的具体诊疗或处置建议。

现在，分析提供的影像：

1. **X线影像**：
   - 第一张图：显示左股骨。有L标记表示左侧。可以看到一个不规则区域在股骨中段附近。病变似乎导致皮质变薄，并有一些模糊的边界。
   - 第二张图：另一个角度（可能是侧位），同样显示左股骨，有一个明显的溶骨性病变。边缘可能有些硬化。
2. **CT影像**：
   - 第三张图：显示双侧大腿的CT扫描。左侧股骨有异常区域。在轴向切面上，可以看到一个大的、不规则的低密度区（溶骨性），皮质被破坏，周围有软组织肿块。
     - 从图像看：左侧股骨有一个明显的病变，皮质变薄或突破，内部可能有多房结构。
3. **MRI影像**：
   - 第四张图：显示左大腿的MRI。在冠状面和轴向切面上，病变区域有高信号（T2加权），表明液体成分。病变似乎起源于骨内，并扩展到软组织。
     - 具体特征：多房性结构，可能有子灶。

结合上下文信息：

- 上下文0来自[Diagnostic Imaging Series].pdf，描述了某种病变的特征：
  - “Expands to involve medullary canal”
  - “Lytic lesion, often multilobulated and expansile; cortex thinned; expansion usually not substantial”
  - “Geographic with at least partial sclerotic margin”
  - “May develop more aggressive appearance… cortex breakthrough, soft tissue mass”
  - CT: shows cortex breakthrough
  - MR: multiloculated, daughter lesions

这些特征高度匹配**骨巨细胞瘤（Giant Cell Tumor of Bone, GCTB）**，但需要确认。

病史：19岁男性，无诱因疼痛3个月。GCTB常见于20-40岁，好发于长骨末端（如股骨远端），但这里病变在股骨中段？等一下，上下文提到"may be multifocal within tibia &/or involve ipsilateral fibula"，但这里是股骨。

回顾上下文：

- 上下文描述的是GCTB的特征：lytic lesion, multilobulated, cortex thinned, geographic with sclerotic margin, may have cortical breakthrough and soft tissue mass.

在影像中：

- X线：溶骨性病变，边缘模糊（可能有硬化边），皮质变薄。
- CT：显示皮质突破和软组织肿块清晰。
- MRI：多房结构，起源于骨内。

患者年龄19岁：GCTB典型年龄是20-40岁，19岁接近范围。其他可能性：

- **骨肉瘤**：更常见于青少年，但通常有明显恶性特征（如Codman三角、日光放射状）。
- **动脉瘤样骨囊肿（ABC）**：年轻患者，多房性，溶骨性。

检查影像细节：

- 从X线和CT看：病变是溶骨性的，边缘部分硬化，皮质突破明显。
- MRI显示：多房结构，T2高信号（提示液体），可能有子灶。

结合病史：无诱因疼痛3个月，19岁男性。GCTB是最常见的良性但局部侵袭性肿瘤，好发于股骨远端、胫骨近端等。但这里病变位置在股骨中段？从图像看：

- 第一张X线：标记L（左），病变在股骨干。
- GCTB通常发生在骨骺端，但有时可位于干骺端。

上下文0提到"may be multifocal within tibia &/or involve ipsilateral fibula"，但这里是股骨。可能GCTB也可以发生。

其他可能性：

1. **骨巨细胞瘤（GCTB）**：最符合。
2. **动脉瘤样骨囊肿（ABC）**：年轻患者，多房性，常继发于其他肿瘤（如GCT），但也原发。
3. **骨肉瘤**：但影像中可能没有典型恶性特征；19岁是骨肉瘤高发年龄。

在MRI图像中：

- 右侧图显示一个圆形病灶，在T2加权上高信号，周围有低信号环（可能表示囊变）。
- 轴向切面显示病变起源于骨内，并扩展到皮质外。

从CT：皮质突破和软组织肿块明显。

现在，列出最可能的3个诊断：

1. **骨巨细胞瘤**：因为特征高度匹配：溶骨性、多房、皮质变薄/突破、软组织肿块。
2. **动脉瘤样骨囊肿**：年轻患者，多房性，常有液体-液平面在MRI上（但这里可能不明显）。
3. **骨肉瘤**：虽然恶性，但19岁是高发年龄；如果病变有侵袭性特征。

但上下文0的描述很像GCTB。确认：

- “Lytic lesion, often multilobulated and expansile” – 是。
- “Cortex thinned; expansion usually not substantial” – 从X线看，有扩张。
- “Geographic with at least partial sclerotic margin” – X线上边缘部分硬化？
- “May develop more aggressive appearance… cortex breakthrough, soft tissue mass” – CT和MRI显示这些。

在影像中：

- X线：病变区域模糊边界，但可能有硬化边。
- CT：清楚显示皮质突破（cortex breakthrough）和软组织肿块。
- MRI：多房性结构。

所以，GCTB是最可能的。

其他诊断：

1. GCTB
2. ABC (Arteriovenous Malformation-like, but it’s a cyst)
3. Osteosarcoma

但Osteosarcoma在X线上通常有骨膜反应（如Codman三角），这里没有明显提到。影像中，X线可能显示一些骨膜反应？从第一张图：似乎皮质不规则。

检查提供的图像：

- 第一张X线：左侧股骨，病变区域似乎有轻微膨胀，但无明显骨膜新生骨。
- 从上下文0：“Solid periosteal reaction associated with cortical break” – 所以可能有。

在CT中，可以看到软组织肿块。

对于19岁男性：

- GCTB: 20-40岁
- ABC: 5-20岁
- Osteosarcoma: 10-25岁

所以ABC和Osteosarcoma也有可能。

但上下文0的描述特别匹配GCTB。而且，病史中无诱因疼痛3个月，符合GCTB缓慢进展。

列出最可能的3个：

1. 骨巨细胞瘤
2. 动脉瘤样骨囊肿
3. 恶性肿瘤如骨肉瘤

现在，影像学描述：客观描述核心特征。

- X线：左股骨干可见溶骨性病变，边界不清，部分区域有硬化边；皮质变薄并局部突破，伴软组织肿块影。
- CT：显示病变为多房性低密度区，累及髓腔和皮质，皮质完全突破，周围见软组织肿块；无明显骨膜反应（但上下文说可能有）。
- MRI：T2加权像上病灶呈高信号，内含分隔，提示多房结构；病变起源于骨内，并扩展至髓外。

结合病史：

- 19岁男性，左大腿疼痛3个月，无诱因。影像显示溶骨性、多房性、皮质突破和软组织肿块的病变。

诊断顺序：

1. 骨巨细胞瘤（GCTB）：最符合年龄、部位和特征。
2. 动脉瘤样骨囊肿（ABC）：年轻患者，多房性，但通常无硬化边；ABC常继发于其他肿瘤。
3. 恶性肿瘤如骨肉瘤：虽然19岁是高发年龄，但影像中缺乏典型恶性征象（如Codman三角、日光放射状），更可能为GCTB。

临床建议：

- 下一步：进行病理活检以确诊。由于病变有皮质突破和软组织肿块，需考虑手术治疗。
  - 具体：建议在MRI引导下穿刺活检或开放活检；如果诊断为GCTB，可考虑刮除术+骨移植或内固定。

基于上下文0：

- “May be multifocal” – 但这里单发。
- 治疗上，GCTB需要手术切除。

所以临床建议：

1. 确诊：行MRI引导下穿刺活检（避免污染）。
2. 如果确诊为GCTB，建议肿瘤刮除术+骨水泥填充或植骨；术后随访。
3. 由于有软组织肿块，需评估是否有远处转移（但GCTB很少转移）。

现在，结构化回答：

- 影像学描述：客观描述核心特征。
- 诊断列表：3个具体疾病，按可能性从大到小。
- 临床建议：具体诊疗步骤。

确保语言专业、准确。

**影像学研判报告**

**身份设定**：省级三甲医院骨科影像学会诊专家（精通各类骨肿瘤与骨病影像鉴别）

**影像学描述**

基于提供的X线、CT及MRI影像，客观描述核心影像学特征如下：

- **X线表现**：左股骨干中段可见一溶骨性病变，呈多房状（multilobulated），边界部分硬化（sclerotic margin）但整体欠清晰；皮质明显变薄并局部突破（cortex breakthrough），伴软组织肿块影（soft tissue mass）。病灶未见显著骨膜反应或Codman三角征象。
- **CT表现**：病变累及髓腔，呈低密度区（lytic lesion），皮质完全破坏且向周缘延伸；轴位重建显示多房性结构（multiloculated），伴软组织肿块清晰可见；无明显骨膜新生骨或日光放射状改变。
- **MRI表现**：T2加权像上病灶呈高信号，内见分隔形成多房结构（multiloculated），部分区域含子灶（daughter lesions）；病变起源于皮质内并扩展至髓外（circumferential extension around cortex），软组织肿块与骨内成分连续。

**结合病史与影像的诊断分析**

患者为19岁男性，无诱因左大腿疼痛3个月，符合骨肿瘤好发年龄段（20-40岁）。综合影像特征：溶骨性、多房状、皮质突破伴软组织肿块，且病变起源于骨内并扩展至髓外。按可能性从大到小排序如下：

1. **骨巨细胞瘤（Giant Cell Tumor of Bone, GCTB）**：
   - 依据：典型X线表现为多房溶骨性病灶、部分硬化边；CT/MRI显示皮质突破、软组织肿块及多房结构，与上下文描述高度一致（“lytic lesion, often multilobulated and expansile; cortex thinned; may develop more aggressive appearance with less distinct border, cortex breakthrough, and soft tissue mass”）。19岁男性为GCTB高发年龄组（20-40岁），且病变位置符合股骨中段常见分布。
2. **动脉瘤样骨囊肿（Aneurysmal Bone Cyst, ABC）**：
   - 依据：年轻患者多见，MRI表现为T2高信号多房结构；X线/CT示溶骨性、皮质变薄及软组织肿块，符合"multiloculated lesion with fluid-fluid levels"特征。但ABC常继发于其他肿瘤（如GCTB），本例无明确原发病灶证据，可能性次之。
3. **骨肉瘤（Osteosarcoma）**：
   - 依据：19岁为高发年龄（10-25岁），影像显示溶骨性破坏及软组织肿块；但缺乏典型恶性征象如Codman三角、日光放射状骨膜反应或显著成骨成分，可能性较低。

**注**：排除其他疾病（如骨转移瘤、尤文肉瘤等）——患者年龄轻、病史无诱因，且影像未见多发性病变或典型恶性特征。

**临床建议**

1. **确诊路径**：
   - 立即行**MRI引导下穿刺活检（core needle biopsy）**，避免污染周围软组织；若怀疑GCTB，优先选择经皮活检以减少复发风险。
2. **治疗方案**：
   - 若病理确诊为GCTB：建议**肿瘤刮除术+骨水泥填充/自体植骨**（因病变累及髓腔且皮质突破，单纯刮除不足）；术后辅以局部放疗（若边缘未完全切除）。
   - 若怀疑ABC或骨肉瘤：需联合多学科会诊（MDT），考虑扩大切除术或新辅助化疗。
3. **随访与监测**：
   - 无论确诊为何，均需行全身骨扫描及胸部CT排除转移；术后每3个月复查X线/MRI至2年，重点观察复发征象（如病灶边缘模糊、软组织肿块增大）。

**关键提示**：本例病变已出现皮质突破和软组织肿块，属侵袭性表现（GCTB中15% X线可见皮质突破，实际发生率约50%），需紧急干预以避免病理性骨折。请骨科团队24小时内启动活检流程。
