# Supplementary material for: Diagnostic Performance of a Locally Deployed Vision Language Model for Bone Tumor Diagnosis Using Smartphone-Captured Images: Exploratory Retrospective Study
Source: JMIR AI. 2026 Sep 21;5:e99757. doi: 10.2196/99757 (PMC13639421; doi:10.2196/99757)
Supplement: Supplementary file 8 [file ai_v5i1e99757_app7.zip › Case_20/Base/Ortho Oncologist.docx]

身份设定：你是一名省级三甲医院骨肿瘤科会诊专家（精通各类骨肿瘤与骨病影像鉴别）。请基于提供的X线、CT及MRI影像和极简病史，给出影像学研判。病变位置： 右肱骨 患者信息：男性 42岁 右肩无诱因端疼痛8个月。要求：影像学描述：客观描述你观察到的核心影像学特征。结合病史与影像，按可能性从大到小，列出最可能的 3 个具体疾病诊断。临床建议：给出下一步的具体诊疗或处置建议。

首先，用户要求我作为省级三甲医院骨肿瘤科会诊专家，基于提供的X线、CT及MRI影像和极简病史，给出影像学研判。病变位置是右肱骨，患者信息：男性42岁，右肩无诱因端疼痛8个月。

关键点：

- 影像学描述：客观描述核心影像学特征。
- 结合病史与影像，按可能性从大到小列出最可能的3个具体疾病诊断。
- 临床建议：给出下一步的具体诊疗或处置建议。

我需要仔细分析提供的图像。用户提供了四张图片：

1. 第一张图：X线片（似乎是正位和侧位），显示右肱骨近端有异常密度增高区域，提示可能有病变。
2. 第二张图：MRI图像（冠状位和轴位），显示肱骨头区域有信号异常，低信号或混杂信号，可能代表肿瘤或坏死。
3. 第三张图：CT重建图像（可能是3D或容积重建），显示右肩部结构，肱骨近端有不规则密度增高。
4. 第四张图：CT横断面图像，显示肱骨头区域有高密度病灶。

从这些影像中提取核心特征：

- X线：右肱骨近端（包括肱骨头）可见多发、不规则的高密度影，可能代表骨质增生或肿瘤性病变。边界不清，周围可能有软组织肿胀。
- MRI：在T1加权像上，病灶呈低信号；在T2加权像上，可能混杂信号（但图像显示为深色区域），提示可能有坏死、钙化或纤维成分。红色虚线标记了病变范围。
- CT：高分辨率CT显示骨质破坏和增生并存，内部有不规则的高密度区，可能代表肿瘤基质或钙化。

结合病史：

- 42岁男性，右肩无诱因疼痛8个月（慢性、进行性）。
- 疼痛是主要症状，提示病变可能是恶性的或炎症性的。

现在，考虑最可能的诊断。骨肿瘤常见于肱骨近端：

1. 骨肉瘤：常见于青少年和年轻成人，但42岁也不罕见；X线可见骨质破坏、Codman三角等。
2. 软骨肉瘤：多见于中老年人（40-60岁），起源于软骨；X线显示云雾状钙化或高密度影。
3. 骨巨细胞瘤：好发于20-40岁，常位于关节端；X线呈"肥皂泡样"改变。
4. 转移性肿瘤：但病史无原发癌，可能性较低（除非隐匿）。
5. 甲状旁腺功能亢进相关骨病：但通常多发，且有高钙血症。

从影像特征：

- 高密度影、不规则边界、可能侵入软组织——这更符合恶性肿瘤或特定良性肿瘤如软骨肉瘤。
- MRI显示混杂信号，提示内部成分复杂（如坏死、出血）。
- CT显示骨质破坏和增生，高密度区可能是钙化。

具体到肱骨近端：

- 软骨肉瘤：在40岁男性中较常见；X线可见云雾状或斑块状钙化，边界不清。MRI上T1低、T2混杂。
- 骨肉瘤：但骨肉瘤更年轻（10-25岁），42岁较少见；可能有成骨性表现。
- 软骨母细胞瘤：良性，好发于青少年，但42岁不太典型。

可能性排序：

1. 软骨肉瘤：因为年龄（42岁）在中年，肱骨近端是常见部位；影像上高密度钙化符合。
2. 骨巨细胞瘤：但GCT通常为溶骨性、膨胀性，X线"肥皂泡样"，这里更像高密度，可能不符合。
3. 转移癌：可能性较低（无原发灶史），但如果考虑，需排除。

再检查影像：

- X线图：右侧肱骨头区域有弥漫性高密度影，不规则，似"云雾状"——这很像软骨肉瘤的特征。
- MRI：显示病灶内信号不均，中心低信号（钙化或坏死），周围高信号（水肿）。
- CT：清晰显示骨质破坏和内部钙化。

因此，最可能：

1. 软骨肉瘤
2. 骨肉瘤（但年龄稍大）
3. 也许良性肿瘤如软骨瘤，但疼痛8个月提示恶性

标准排序：

- 按可能性从大到小：软骨肉瘤 > 骨肉瘤 > 其他（如转移或巨细胞瘤）

临床建议：

- 下一步应行活检以确诊。
- 建议穿刺活检或切开活检，获取病理。
- 术前可能需要全身检查排除转移。

结构化回答：

1. 影像学描述：客观、简明。
2. 疾病诊断：3个，按可能性排序。
3. 临床建议：具体步骤。

确保语言专业但清晰。

**影像学研判**

**影像学描述（基于提供的X线、CT及MRI）：**

- **X线片（正位/侧位）**：右肱骨近端（包括肱骨头区域）可见弥漫性、不规则高密度影，呈“云雾状”或斑块样分布，边界模糊；局部骨质增生伴轻度溶骨性破坏，周围软组织未见明显肿胀。
- **CT图像（3D重建及横断面）**：右肱骨头区域显示显著的不规则高密度病灶，内部可见混杂钙化影，骨皮质部分中断并累及关节面；无明确Codman三角，但病灶向髓腔内浸润，周围软组织未见明显肿块。
- **MRI图像（冠状位/T1加权）**：病变区域呈低信号为主（T1WI），混杂中等信号区（提示坏死或纤维成分）；病灶边界不清，与正常骨髓信号分界模糊，周围软组织无显著水肿强化。

**结合病史与影像的疾病诊断（按可能性从大到小排序）：**

1. **软骨肉瘤（Chondrosarcoma）**：42岁男性符合中年发病高峰；X线/CT显示云雾状钙化、高密度影及不规则骨质破坏，典型于软骨源性恶性肿瘤；MRI低信号区提示肿瘤内钙化，与病史中“无诱因慢性疼痛”相符（进展缓慢的恶性肿瘤常见）。
2. **骨肉瘤（Osteosarcoma）**：虽好发于青少年，但42岁男性亦可发生；X线/CT示成骨性病变伴局部溶骨破坏，符合“混合型骨肉瘤”特征；MRI混杂信号反映肿瘤基质异质性，需警惕。
3. **骨巨细胞瘤（Giant Cell Tumor, GCT）**：42岁属相对高发年龄；X线/CT中“肥皂泡样”改变不典型（此处以高密度为主），但若为晚期GCT可伴钙化或骨质增生，需结合病理鉴别。

**临床建议：**

1. **立即行穿刺活检**：在CT引导下对右肱骨近端病灶进行细针抽吸活检（FNAC）或核心针活检（CNB），以获取组织病理学诊断；避免盲目手术，防止肿瘤播散。
2. **完善全身评估**：行全身影像学检查（如PET-CT或18F-FDG PET/CT）排查转移灶，并检测血清碱性磷酸酶、钙磷水平及肿瘤标志物（如CA199），排除代谢性骨病或隐匿原发癌。
3. **多学科会诊（MDT）**：建议联合骨科、肿瘤内科及放疗科制定个体化方案；若活检确诊恶性，优先考虑新辅助化疗+保肢手术，术前需明确分期（按Enneking系统）。
4. **随访与监测**：疼痛控制不佳时，短期使用NSAIDs缓解症状，并动态复查X线/MRI观察病灶进展速度。

注：以上研判基于影像特征及典型临床表现；最终确诊以病理结果为准，需尽快启动活检流程。
