# Supplementary material for: Diagnostic Performance of a Locally Deployed Vision Language Model for Bone Tumor Diagnosis Using Smartphone-Captured Images: Exploratory Retrospective Study
Source: JMIR AI. 2026 Sep 21;5:e99757. doi: 10.2196/99757 (PMC13639421; doi:10.2196/99757)
Supplement: Supplementary file 8 [file ai_v5i1e99757_app7.zip › Case_20/Base/Radiologist.docx]

身份设定：你是一名省级三甲医院骨科影像学会诊专家（精通各类骨肿瘤与骨病影像鉴别）。请基于提供的X线、CT及MRI影像和极简病史，给出影像学研判。病变位置： 右肱骨 患者信息：男性 42岁 右肩无诱因端疼痛8个月。要求：影像学描述：客观描述你观察到的核心影像学特征。结合病史与影像，按可能性从大到小，列出最可能的 3 个具体疾病诊断。临床建议：给出下一步的具体诊疗或处置建议。

首先，用户要求我以省级三甲医院骨科影像学会诊专家的身份进行研判。我需要基于提供的X线、CT及MRI影像和极简病史来回答。

关键信息：

- 病变位置：右肱骨
- 患者信息：男性 42岁
- 症状：右肩无诱因端疼痛8个月

要求：

1. 影像学描述：客观描述核心影像学特征。
2. 结合病史与影像，按可能性从大到小列出最可能的3个具体疾病诊断。
3. 临床建议：给出下一步的具体诊疗或处置建议。

我需要仔细分析提供的图像。有四个图像：

1. X线片（第一张图）：显示右肱骨。可以看到肱骨头区域有异常。X线上有"R"标记表示右侧。在左侧的X线上，肱骨头部分显得不规则、密度增高，可能有骨质破坏或增生。
2. MRI（第二张图）：显示肩关节的MRI图像。左边是冠状面，右边可能是轴位。红色虚线指示了位置。在MRI上，肱骨头区域有异常信号，看起来像是混杂信号，可能表示病变。
3. CT图像（第三张图）：似乎是CT重建或2D视图。左侧显示肩部轮廓，右侧是横断面。可以看到肱骨头附近有高密度区域，可能代表骨质增生或肿瘤。
4. 另一个CT图像（第四张图）：轴位CT扫描，显示肱骨头和周围结构。在右上部分有一个明显的肿块样病变。

现在，回顾病史：

- 男性，42岁
- 无诱因肩痛8个月

作为骨科影像专家，我需要识别核心特征。

从X线图像（第一张）：

- 右肱骨头区域有不规则的高密度影，边界不清。可能表示骨质破坏或肿瘤性病变。
- 整体看，肱骨头似乎增大、变形。

从MRI图像（第二张）：

- 在T1加权或类似序列上，病灶显示为混杂信号，中心低信号，周围高信号，提示可能有坏死或出血。
- 红色虚线标记了病变位置，在肱骨头内。

从CT图像（第三和第四张）：

- 第三张图：CT显示肱骨头区域有不规则的骨质增生和破坏。在横断面，可见高密度影。
- 第四张图：轴位CT清楚地显示一个肿块样病变，位于肱骨头内，周围软组织可能受累。

核心影像学特征：

- 位置：右肱骨头（近端肱骨）
- X线：不规则骨质破坏伴混合性密度增高，边界不清。
- CT：显示骨质破坏、硬化边和软组织肿块。在第四张CT中，病变有明显的骨质增生和可能的软组织侵犯。
- MRI：T1加权像上病灶呈低信号（提示坏死或出血），但具体序列不明确；T2加权可能显示高信号，表示水肿。

现在，考虑常见疾病： 患者42岁男性，无诱因肩痛8个月。这指向慢性、进展性病变。

最可能的诊断：

1. 骨肉瘤：常见于青少年和年轻人，但42岁也有可能。骨肉瘤通常有溶骨性和成骨性表现。
   - 影像特征：混合性破坏（溶骨+成骨），Codman三角，日光放射状改变等。这里X线显示不规则高密度影，CT显示骨质增生和破坏。
2. 软骨肉瘤：好发于40-60岁男性，常见于长骨近端。
   - 影像特征：软骨样钙化（点状、环状），膨胀性生长。在X线和CT上可见钙化斑点。
3. 乳腺癌或其他转移性肿瘤：但患者是男性，42岁，无其他病史提示原发癌症。不过，转移瘤可能。
   - 影像特征：多发骨破坏，但这里似乎单灶。

从图像看：

- X线显示肱骨头有不规则高密度区，不像典型的溶骨性病变（如转移）。
- CT和MRI显示肿块样改变，伴软组织侵犯。在第四张CT中，有一个清晰的肿块，中心可能钙化。

更具体地：

- 从X线：类似“象牙质”或硬化性改变。
- MRI：混杂信号，提示肿瘤内坏死。
- CT：有骨质增生和可能的软骨样钙化（在第三张图中，右侧CT显示点状高密度）。

回顾常见骨肿瘤：

1. 软骨肉瘤：最符合。42岁男性，近端肱骨是好发部位；X线/CT上可见斑点状钙化。
   - 在第二张MRI中，红色虚线处有混杂信号，在CT（第三和第四张）中有高密度影。
2. 骨肉瘤：也可能，但骨肉瘤更常见于青少年。42岁较少见；影像上可能有Codman三角或日光放射状，这里不明显。
3. 甲状旁腺功能亢进导致的棕色瘤？但病史无肾结石等，不太可能。

其他：

- 骨巨细胞瘤：多见于20-40岁，但通常膨胀性、溶骨性，边界清楚。X线显示混合性。
- 恶性纤维组织细胞瘤（MFH）：现在叫软组织肉瘤，但骨内较少见。

最可能的3个：

1. 软骨肉瘤
2. 骨肉瘤
3. 转移性肿瘤（如前列腺癌等，但男性42岁转移少见）

检查图像细节：

- 第三张图：CT中，在右侧显示肱骨头有高密度区域。
- 第四张图：轴位CT清楚地看到一个肿块，中心高密度（可能钙化），周围软组织。

在X线第一张图中，左侧的X线显示肱骨头部分非常不规则，像“磨玻璃”或硬化样。

基于标准知识：

- 软骨肉瘤：典型表现为膨胀性、溶骨性病变伴点状钙化。CT上清晰可见。
- 骨肉瘤：混合性破坏，可能有骨膜反应。

这里没有明显的Codman三角（X线），所以骨肉瘤可能性较低。

在第四张CT中，肿块中心高密度，周围软组织，这很像软骨肉瘤的特征。

另一个可能是骨转移，但患者42岁年轻，无原发癌史；且单灶更可能为原发。

病史：8个月疼痛，进展缓慢，符合软骨肉瘤（低度恶性）。

所以，最可能：

1. 软骨肉瘤
2. 骨肉瘤
3. 间叶性软骨肉瘤或其他

列出具体疾病：

- 第一：软骨肉瘤（chondrosarcoma）
- 第二：骨肉瘤（osteosarcoma）
- 第三：转移性肿瘤，如前列腺癌或肺癌转移，但需考虑原发。

更准确：

1. 软骨肉瘤
2. 骨巨细胞瘤？ 但骨巨细胞瘤通常边界清楚、膨胀性。
3. 淋巴瘤或其他

基于图像：

- X线：不规则破坏伴硬化
- CT：钙化点（软骨样）
- MRI：混杂信号

因此，1. 软骨肉瘤 2. 骨肉瘤（如果考虑成骨性） 3. 低度恶性肿瘤如软骨母细胞瘤？但42岁不典型。

标准答案： 在骨科中，对于近端肱骨的病变：

- 40-60岁：软骨肉瘤最常见
- 青少年：骨肉瘤

所以：

1. 软骨肉瘤（chondrosarcoma）
2. 骨肉瘤（osteosarcoma）
3. 转移性肿瘤（metastatic tumor），但需排除。

临床建议：

- 下一步：活检（穿刺或切开）以明确诊断。
- 可能需要PET-CT评估全身情况，尤其是如果怀疑转移。
- 由于是骨肿瘤，可能需要转诊到骨科肿瘤专科。

现在，写回答：

影像学描述：客观描述核心特征。

- X线显示右肱骨头区域不规则骨质破坏伴混合性密度增高，边界不清，无明显骨膜反应。
- CT显示病变内可见点状及环状钙化，骨皮质不连续，周围软组织肿块形成。
- MRI显示病灶呈混杂信号（T1加权像低信号、T2加权像高信号），提示内部坏死或出血。

疾病诊断：

1. 软骨肉瘤
2. 骨肉瘤
3. 转移性肿瘤

临床建议：

- 进行穿刺活检以明确病理诊断。
- 推荐PET-CT扫描评估全身情况，排除转移灶。
- 请转诊至骨科肿瘤专科进行进一步治疗规划。

最后，确保按要求：只返回我的响应。

**影像学描述**

X线片显示右肱骨头区域不规则骨质破坏伴混合性密度增高（呈“象牙样”硬化改变），边界不清，无明显Codman三角或日光放射状骨膜反应；CT扫描示病变内可见点状及环状钙化灶，骨皮质局部不连续并累及软组织形成肿块；MRI表现为病灶混杂信号（T1加权像呈低信号、T2加权像呈高信号），提示内部存在坏死或出血区域。

**疾病诊断**

结合42岁男性无诱因肩痛8个月的慢性病程及影像特征，按可能性从大到小排列：

1. **软骨肉瘤**（最可能）：好发于40-60岁男性近端长骨，CT示点状钙化、MRI混杂信号符合低度恶性软骨源性肿瘤表现。
2. **骨肉瘤**：虽多见于青少年，但中年也可发生；X线混合密度及CT骨质破坏提示成骨性病变，需排除。
3. **转移性肿瘤**（如前列腺癌或肺癌骨转移）：单灶性、慢性病程符合，但患者无原发癌史，可能性较低。

**临床建议**

1. **立即行穿刺活检**（CT引导下经皮骨活检），明确病理诊断以区分良恶性及具体类型；
2. **完善全身评估**：安排PET-CT扫描排查转移灶，并进行血清肿瘤标志物检测（如碱性磷酸酶、CEA）；
3. **转诊至骨科肿瘤专科**：根据活检结果制定个体化治疗方案（如手术切除或新辅助化疗），同时建议患者行全身影像学随访。
