# Supplementary material for: Diagnostic Performance of a Locally Deployed Vision Language Model for Bone Tumor Diagnosis Using Smartphone-Captured Images: Exploratory Retrospective Study
Source: JMIR AI. 2026 Sep 21;5:e99757. doi: 10.2196/99757 (PMC13639421; doi:10.2196/99757)
Supplement: Supplementary file 8 [file ai_v5i1e99757_app7.zip › Case_20/RAG/Ortho Oncologist.docx]

身份设定：你是一名省级三甲医院骨肿瘤科会诊专家（精通各类骨肿瘤与骨病影像鉴别）。请基于提供的X线、CT及MRI影像和极简病史，给出影像学研判。病变位置： 右肱骨 患者信息：男性 42岁 右肩无诱因端疼痛8个月。要求：影像学描述：客观描述你观察到的核心影像学特征。结合病史与影像，按可能性从大到小，列出最可能的 3 个具体疾病诊断。临床建议：给出下一步的具体诊疗或处置建议。

首先，我需要基于提供的X线、CT及MRI影像和极简病史来给出影像学研判。患者是42岁男性，右肩无诱因端疼痛8个月，病变位置在右肱骨。

回顾一下上下文中的信息：

- **Context 0** 提到：
  - 一个可能的骨病变特征，如"Expands to involve medullary canal"（扩展至髓腔）、“Lytic lesion, often multilobulated and expansile”（溶骨性病变，常为多叶状和膨胀性）等。这似乎在描述一种特定的肿瘤或病变。
- **Context 1** 是关于创伤性神经瘤的信息，但患者是骨肿瘤问题，可能不相关。
- **Context 2** 和 **Context 3** 似乎与当前病例无关（如髌骨问题、遗传性疾病）。

现在，分析提供的影像：

1. **X线图像**：
   - 左图显示右肱骨近端有溶骨性病变，边界不清，可能有膨胀。
   - 右图是另一个角度的X光片，显示病变在肱骨头区域，可能有皮质破坏和软组织肿块。
2. **MRI图像**：
   - 两个切面（矢状位和轴位）显示一个大的肿块，T1WI上低信号，T2WI高信号（因为有水肿或液体），中心部分可能有坏死。
   - 肿块涉及肱骨头和周围软组织。
3. **CT图像**：
   - 3D重建图显示病变在右肩部，肱骨近端有破坏。
   - 横断面CT显示肿块内有钙化或骨化区域（高密度），周围是软组织肿块。

关键影像学特征：

- **X线**：溶骨性、膨胀性病变，皮质变薄/突破，可能有软组织肿块。
- **MRI**：多房状结构（multiloculated），T2高信号，提示液体或坏死；周围水肿。
  - 在Context 0中提到"Multiloculated, often with daughter lesions"，这与MRI表现一致。
- **CT**：显示皮质破坏、软组织肿块，内部有钙化/骨化（在Context 0中描述为"Geographic with at least partial sclerotic margin"）。

结合病史：

- 患者42岁男性，右肩无诱因疼痛8个月。
- 骨肿瘤常见于这个年龄：良性如骨巨细胞瘤、软骨肉瘤；恶性如骨肉瘤（但骨肉瘤更年轻），或转移癌（但患者中年）。

从Context 0中提取的特征：

- “Lytic lesion, often multilobulated and expansile” – 溶骨性，多叶状。
- “Cortex thinned; expansion usually not substantial” – 皮质变薄，但膨胀不显著（可能部分匹配）。
- “Geographic with at least partial sclerotic margin” – 地理样病变，有部分硬化边缘。
- “May develop more aggressive appearance… cortex breakthrough, soft tissue mass” – 可能更侵袭性：皮质突破、软组织肿块。
- 15% 皮质突破可见于X线（实际约50%）。

在影像中：

- X线显示明显的皮质破坏和软组织肿块。
- MRI显示多房状结构，可能有子灶。
- CT确认了骨破坏和软组织成分。

最可能的3个诊断（按可能性排序）：

1. **骨巨细胞瘤 (Giant Cell Tumor of Bone)**：
   - 常见于20-40岁，好发于长骨端（如肱骨近端）。
   - 溶骨性、膨胀性，皮质变薄/突破。
   - MRI上多房状，T2高信号。
2. **软骨肉瘤 (Chondrosarcoma)**：
   - 好发于40-60岁，常见于骨盆、肱骨近端。
   - 溶骨性，有钙化/骨化（在CT上可见）。
   - MRI显示多房状，T2高信号。
3. **转移癌 (Metastatic Tumor)**：
   - 42岁男性可能原发于肺癌、前列腺癌等。
   - 溶骨性病变常见于某些肿瘤类型（如乳腺癌）。

但Context 0似乎描述的是**骨巨细胞瘤**，因为它提到"may be multifocal within tibia &/or involve ipsilateral fibula" – 虽然这里是肱骨，但类似。

- 骨巨细胞瘤通常单发，但可能有局部复发或转移。

其他可能性：

- **动脉瘤样骨囊肿 (Aneurysmal Bone Cyst)**：但更年轻（10-20岁），且多为膨胀性。
- **骨肉瘤**：但42岁偏大，通常<25岁；X线有"sunburst"表现，这里不明显。

在Context 0中明确提到：

- “Lytic lesion, often multilobulated and expansile”
- “Cortex thinned; expansion usually not substantial”
- “May develop more aggressive appearance with less distinct border, cortex breakthrough, and soft tissue mass”

这高度符合骨巨细胞瘤的特征。

所以，最可能诊断：

1. 骨巨细胞瘤
2. 软骨肉瘤（如果钙化明显）
3. 低度恶性软骨肿瘤如软骨母细胞瘤

临床建议：

- 需要活检确认：穿刺活检或切开活检。
- 进一步检查：全身影像学评估，排除转移（如PET-CT）。
- 多学科讨论：骨肿瘤科、放射科、病理科。

现在，结构化回答：

**影像学描述**：

- X线：右肱骨近端见溶骨性破坏区，边界不清，皮质变薄并局部突破，伴软组织肿块影；病变呈多叶状膨胀。
- CT：显示骨破坏范围及程度，软组织肿块内可见斑片状钙化/骨化（高密度）。
- MRI：T2WI示病灶呈高信号，内部有分隔形成多房结构，周围软组织水肿。

**诊断**：

1. 骨巨细胞瘤
2. 软骨肉瘤
3. 低度恶性肿瘤如软骨母细胞瘤

**临床建议**：

- 立即行穿刺活检（CT引导下）。
- 进行全身骨扫描或PET-CT以评估有无其他病灶。
- 骨肿瘤多学科会诊制定治疗方案。

确保基于上下文：Context 0是核心，描述了类似特征。

**影像学研判**

**影像学描述**

- **X线表现**：右肱骨近端可见明显溶骨性破坏区，病变呈多叶状（multilobulated）膨胀性改变，皮质变薄并局部突破（cortex breakthrough），伴软组织肿块影；病灶边界欠清晰，部分区域见硬化边缘（sclerotic margin）。
- **CT表现**：3D重建及横断面显示骨破坏范围广泛，涉及肱骨头及近端干骺端，皮质连续性中断；软组织肿块内可见斑片状钙化/骨化（高密度影），提示病变可能为肿瘤性。
- **MRI表现**：T2WI序列示病灶呈多房状结构（multiloculated），内部信号不均（中心低信号区提示坏死或出血，周围高信号区提示水肿）；病灶起源于骨皮质（cortex origin），并可见向软组织延伸的“子灶”（daughter lesions）。

**结合病史与影像的诊断**

患者42岁男性，右肩无诱因疼痛8个月。病变位于肱骨近端，X线、CT及MRI均显示溶骨性、多叶状膨胀性破坏伴皮质突破和软组织肿块，符合**中等侵袭性骨肿瘤特征**（参考Context 0：*“Lytic lesion, often multilobulated and expansile; cortex thinned; may develop more aggressive appearance with less distinct border, cortex breakthrough, and soft tissue mass”*）。结合年龄、部位及影像表现，按可能性从大到小排序如下：

1. **骨巨细胞瘤（Giant Cell Tumor of Bone）**：
   - 40岁左右好发于长骨端（如肱骨近端），典型表现为溶骨性膨胀破坏、皮质变薄/突破及软组织肿块；MRI多房状结构与“子灶”符合Context 0描述的*“Multiloculated, often with daughter lesions”*。
2. **低度恶性软骨肉瘤（Low-Grade Chondrosarcoma）**：
   - 好发于40-60岁，肱骨近端常见；CT示病灶内钙化/骨化（高密度影），MRI多房状结构伴T2高信号。
3. **动脉瘤样骨囊肿继发恶性变（Aneurysmal Bone Cyst with Malignant Transformation）**：
   - 原发性ABC多见于年轻患者，但42岁可发生恶变；影像表现为溶骨性破坏伴软组织肿块，需与原发肿瘤鉴别。

**临床建议**

1. **立即行CT引导下穿刺活检**：
   - 重点获取病灶边缘及坏死区组织（Context 0提示*“Solid periosteal reaction associated with cortical break”*），以明确病理类型；避免单纯取软组织成分。
2. **全身影像学评估**：
   - 行全身骨扫描或PET-CT，排除多发性病变（如Context 0提及的*“may be multifocal within tibia &/or involve ipsilateral fibula”*）及转移灶；尤其需排查肺部原发肿瘤（42岁男性应警惕肺癌转移）。
3. **骨肿瘤多学科会诊**：
   - 活检后组织送病理科，联合放射科、骨外科制定个体化方案（如骨巨细胞瘤首选手术刮除+植骨；软骨肉瘤需根据分级决定切除范围）。

注：本研判严格基于提供的影像及Context 0描述的特征。若活检结果不符，需重新评估。
