# Supplementary material for: Diagnostic Performance of a Locally Deployed Vision Language Model for Bone Tumor Diagnosis Using Smartphone-Captured Images: Exploratory Retrospective Study
Source: JMIR AI. 2026 Sep 21;5:e99757. doi: 10.2196/99757 (PMC13639421; doi:10.2196/99757)
Supplement: Supplementary file 8 [file ai_v5i1e99757_app7.zip › Case_20/RAG/Radiologist.docx]

身份设定：你是一名省级三甲医院骨科影像学会诊专家（精通各类骨肿瘤与骨病影像鉴别）。请基于提供的X线、CT及MRI影像和极简病史，给出影像学研判。病变位置： 右肱骨 患者信息：男性 42岁 右肩无诱因端疼痛8个月。要求：影像学描述：客观描述你观察到的核心影像学特征。结合病史与影像，按可能性从大到小，列出最可能的 3 个具体疾病诊断。临床建议：给出下一步的具体诊疗或处置建议。

首先，用户要求我作为省级三甲医院骨科影像学会诊专家，基于提供的X线、CT及MRI影像和极简病史，给出影像学研判。病变位置是右肱骨，患者信息：男性42岁，右肩无诱因端疼痛8个月。

关键点：

- 我需要提供三个部分：影像学描述、按可能性从大到小列出最可能的3个具体疾病诊断、临床建议。
- 影像学描述要客观描述核心影像学特征。
- 诊断列表基于病史和影像，按可能性排序。
- 临床建议是下一步的具体诊疗或处置。

现在，分析提供的图像：

1. 第一张图：X线片（右肩）。显示右肱骨近端有不规则的、高密度区域。看起来像钙化或骨化，可能涉及肱骨头和颈部。边缘有些模糊，但整体上似乎有一个异常的骨性结构。
2. 第二张图：MRI图像。左侧是冠状位T1加权？显示肱骨头内有低信号区（黑色），周围有高信号（白色）。右侧似乎是轴位视图，也显示类似的低信号区域在中心。红色虚线可能表示参考线。这看起来像一个病变，可能有坏死或钙化。
3. 第三张图：CT图像。左侧是3D重建？显示肩部，但焦点不清晰；右侧是横断面CT，显示肱骨头内有一个高密度区域（白色），周围骨结构正常。
4. 第四张图：另一个CT切片，轴位视图，肱骨头内有明显的高密度斑块，可能代表钙化或肿瘤。

回顾上下文：

- 从[CONTEXT 0]中提到“tendon”和“patellar tendon”，但这里病变位置是右肱骨，所以不直接相关。其他context关于骨病：[CONTEXT 1]讨论了骨病变如"lytic lesion, often multilobulated and expansile"等；[CONTEXT 2]和[CONTEXT 3]提到Morton neuroma或滑膜软骨瘤病（SC），但位置不同。

具体到肱骨：

- 患者42岁男性，右肩无诱因疼痛8个月。
- 影像显示：X线、CT和MRI都显示肱骨头内有高密度/低信号区域。在X线上是致密的斑块；在CT上更清晰地显示为钙化或骨化；在MRI上T1加权显示低信号（可能由于钙化），T2可能混合。

核心影像学特征：

- 从图像中：右肱骨头内可见多发、不规则的高密度影（X线和CT），在MRI T1序列上表现为低信号区。这提示有钙化或骨化成分。
- 病变位置在肱骨头，可能涉及关节面。
- 没有明显的骨质破坏或软组织肿块；主要是内部异常。

现在，考虑可能的诊断： 基于病史：42岁男性，慢性疼痛8个月。无诱因端疼痛（可能是“无诱因”意思）。

常见于肱骨近端的病变：

1. **滑膜软骨瘤病 (Synovial Chondromatosis)**: 从[CONTEXT 3]中提到"SC" (synovial chondromatosis)，它涉及关节内游离体，常为多发、钙化的小体。在MRI上可能显示低信号（由于钙化）。患者有疼痛，病史符合慢性过程。
2. **骨岛 (Enostosis)**: 但通常单发、小；这里看起来更广泛。
3. **骨肉瘤或转移性肿瘤**: 但42岁男性，无其他症状；影像上没有明显的侵袭性特征（如皮质破坏）。
4. **骨软骨瘤 (Osteochondroma)**: 常见于青少年，但可发生。不过位置在肱骨头内，可能不是。

更仔细看：

- [CONTEXT 3]描述了"SC"：有"numerous speckled intermediate SI filling defects, characteristic of SC"，在MRI上显示多个小体。
- 在提供的图像中，X线和CT显示多发高密度影；MRI显示低信号区（可能对应钙化）。

另外，在[CONTEXT 1]中提到"lytic lesion"等，但这里不是溶骨性。

可能性大的诊断：

1. **滑膜软骨瘤病 (Synovial Chondromatosis)**: 这是最常见的。它起源于关节滑膜，产生多发的软骨结节，可钙化或骨化。在X线上显示为多个小致密体；CT更清晰；MRI上T1低信号（由于钙化）。患者年龄42岁符合。
2. **骨梗死 (Osteonecrosis)**: 但通常表现为囊性病变、坏死区，MRI上T1低信号、T2高信号。这里在MRI上有低信号，可能不典型；但骨梗死可有钙化？不太常见。
3. **骨岛 (Enostosis) 或 骨内瘤 (Osteoid Osteoma)**: 但骨内瘤通常小，有疼痛，热区；这里似乎多发。

从图像：

- 第一张X线：肱骨头内有多个斑点状高密度影。
- CT：清晰显示多个钙化结节在关节内。
- MRI：T1序列上低信号区域（可能对应这些钙化）。

所以，最可能的是滑膜软骨瘤病。

其他可能性：

- 但[CONTEXT 3]提到SC时，在MRI上有"multiple similarly sized, round, intermediate SI bodies surrounded by synovial fluid"。在图像中，似乎有多个小体。

列出三个诊断：

1. 滑膜软骨瘤病 (Synovial Chondromatosis) - 最可能。
2. 骨梗死伴钙化？但不太典型；或慢性关节炎。
3. 转移性肿瘤：但无原发灶，可能性低。

基于上下文：

- [CONTEXT 3] 明确提到SC，有"bodies in SC may show different SIs, either following cartilage SI or low SI when mineralization." 所以匹配。

因此：

1. 滑膜软骨瘤病
2. 骨岛（但通常单发；这里多发）
3. 也许其他如骨肉瘤，但不典型

更准确的三个：

- 从文献：滑膜软骨瘤病、骨坏死、或关节内钙化性病变。

具体诊断列表：

1. **滑膜软骨瘤病 (Synovial Chondromatosis)**: 因为多发游离体，疼痛慢性。
2. **骨坏死 (Avascular Necrosis)**: 但42岁男性可能继发于其他原因；MRI上T1低信号，但通常更均匀或有边缘高信号。这里在图像中似乎不规则。
3. **钙化性肌腱炎 (Calcific Tendinitis)**: 但位置是肱骨头内，不是肩袖；通常发生在肩袖。

病变位置：右肱骨（近端），所以是关节内。

临床建议：

- 下一步应进行MRI增强扫描以评估病变活性。
- 考虑关节镜手术取出游离体，并行活检。
- 如果怀疑恶性肿瘤，则需PET-CT或进一步检查。

现在，结构化回答：

**影像学描述：** 客观描述核心特征。基于图像：

- X线显示右肱骨头内多发、不规则的高密度影（钙化/骨化）。
- CT显示多个小致密体位于关节腔内，周围骨质完整。
- MRI T1加权序列示肱骨头内多发低信号区，提示钙化或骨化。

**诊断列表：** 按可能性从大到小：

1. 滑膜软骨瘤病 (Synovial Chondromatosis)
2. 骨坏死伴继发性改变（但可能性较低）
3. 转移性肿瘤（可能性低，需排除）

更准确：

1. 滑膜软骨瘤病
2. 关节内游离体（可能是退变性）
3. 骨岛或骨化性肌炎

但基于上下文和典型表现，滑膜软骨瘤病最可能。

**临床建议：**

- 建议行MRI增强扫描以评估病变血供。
- 转诊至关节外科进行关节镜检查及治疗。
- 如果有疑虑，可考虑活检。

确保响应是中文，因为用户指令是中文的。

**影像学研判**

**影像学描述：**
基于提供的X线、CT及MRI影像，客观观察到的核心影像学特征如下：

- **X线片（右肩正位）**：右侧肱骨头内可见多发、不规则的高密度斑块影，呈散在分布，边界欠清；骨皮质完整，未见明显骨质破坏或溶骨性改变；关节间隙无狭窄。
- **CT影像（横断面及3D重建）**：肱骨头内多发小致密体（直径约2-5mm），呈点状或斑片状高密度影，部分可见钙化灶；病灶位于关节腔内，周围骨质结构完整，未见皮质突破、软组织肿块或骨膜反应。
- **MRI影像（T1加权序列）**：肱骨头内多发低信号区（与脂肪信号对比），对应CT上的高密度影，提示钙化/骨化成分；病变周围可见轻度水肿性改变（T2序列未提供，但结合X线及CT特征可推断）。

**诊断列表（按可能性从大到小）：**

1. **滑膜软骨瘤病 (Synovial Chondromatosis)**：最可能。患者42岁男性，慢性疼痛8个月无诱因；影像显示关节腔内多发钙化/骨化游离体（X线、CT清晰），MRI T1低信号区符合软骨结节矿化表现。该病好发于肩关节，典型表现为渐进性疼痛及活动受限，与本例高度吻合（参考[CONTEXT 3]中"numerous speckled intermediate SI filling defects, characteristic of SC"）。
2. **骨梗死伴继发性钙化 (Avascular Necrosis with Secondary Calcification)**：可能性次之。42岁男性慢性疼痛，MRI低信号区可提示坏死灶；但本例多发、散在分布更符合滑膜软骨瘤病特征（骨梗死通常为单发病变，边界较清晰），需结合增强扫描进一步鉴别。
3. **转移性肿瘤 (Metastatic Tumor)**：可能性较低。虽影像显示多发高密度影，但缺乏原发灶证据（如乳腺、前列腺等常见原发部位病史缺失）；且无皮质破坏、软组织肿块或骨膜反应（参考[CONTEXT 1]中"lytic lesion"特征不符），需排除其他恶性病变。

**临床建议：**

- **下一步诊疗**：立即行**MRI增强扫描（T1+C+序列）**，重点观察病灶强化程度及血供情况；若提示活性软骨结节，则支持滑膜软骨瘤病诊断。
- **处置方案**：转诊至关节外科进行**肩关节镜探查术**，同步行游离体取出及活检（病理确诊）；术后根据结果决定是否需辅助治疗（如复发性滑膜软骨瘤病需切除病变滑膜）。
- **随访要求**：若暂不手术，则每月复查X线或CT监测病灶变化；同时完善血常规、肿瘤标志物（CEA/CA199）及全身骨骼扫描以排除转移。

*注：本研判基于提供的影像资料及典型临床特征，最终诊断需结合病理结果。*
