# Supplementary material for: Diagnostic Performance of a Locally Deployed Vision Language Model for Bone Tumor Diagnosis Using Smartphone-Captured Images: Exploratory Retrospective Study
Source: JMIR AI. 2026 Sep 21;5:e99757. doi: 10.2196/99757 (PMC13639421; doi:10.2196/99757)
Supplement: Supplementary file 8 [file ai_v5i1e99757_app7.zip › Case_21/Base/Radiologist.docx]

身份设定：你是一名省级三甲医院骨科影像学会诊专家（精通各类骨肿瘤与骨病影像鉴别）。请基于提供的X线、CT及MRI影像和极简病史，给出影像学研判。病变位置：左股骨 患者信息：男性 15岁 左大腿疼痛2个月。要求：影像学描述：客观描述你观察到的核心影像学特征。结合病史与影像，按可能性从大到小，列出最可能的 3 个具体疾病诊断。临床建议：给出下一步的具体诊疗或处置建议。

首先，我需要仔细分析用户提供的X线、CT和MRI影像资料，并结合15岁男性患者左大腿疼痛2个月的病史进行研判。

从X线片观察：左股骨近端可见一溶骨性病变，边界相对清晰但有轻度膨胀。病变区域骨皮质变薄，局部有不规则破坏，内部可见少量斑点状钙化影。周围未见明显软组织肿块。

CT影像显示：病变为偏心性生长的囊性结构（CT轴位），内壁呈分叶状，伴多发小点状高密度影。骨皮质受累区域呈现"地图样"破坏，局部有骨膜反应形成Codman三角。增强扫描可见病变周边强化。

MRI表现：T1加权像上病变为低信号（与肌肉相仿），T2加权呈混杂高信号，内部见分隔。病变范围较X线和CT显示更广泛，向股骨髓腔内延伸并突破皮质侵犯周围软组织，但未累及关节面。增强扫描示病变周边环形强化。

基于15岁青少年这一关键年龄特征（骨肿瘤好发年龄段），结合以下核心影像学特点：

- 位于长骨干骺端的偏心性溶骨性病变
- 骨皮质破坏伴轻度膨胀
- 内部多发小点状钙化
- 软组织侵犯但未达关节
- 病变边界相对清晰（提示良性或低度恶性可能）

按可能性排序：

1. 骨巨细胞瘤：青少年骨骺闭合前罕见，但本例病灶位于股骨近端干骺端，需考虑。典型表现为偏心性溶骨、膨胀性生长，X线可见"肥皂泡样"改变（此处未完全显现），CT示多房囊状结构。
2. 骨肉瘤：15岁是发病高峰年龄，但本例病变边界相对清晰不符合经典高度恶性骨肉瘤特征。更支持低度恶性或非典型性骨肉瘤。
3. 动脉瘤样骨囊肿：青少年好发，X线见膨胀性透亮区伴内壁钙化，CT示多房囊腔结构，MRI为液-液平面（本例T2混杂信号符合）。

需要排除的疾病：

- 骨软骨瘤（良性但无疼痛）
- 尤文肉瘤（好发年龄更小、病变边界不清）
- 恶性淋巴瘤（多为多发病灶）

临床建议应侧重鉴别诊断和确诊路径。考虑到患者年轻且病变为单发，需优先考虑活检明确病理类型。

影像学描述： X线显示左股骨近端偏心性溶骨性病变，边界轻度膨胀伴骨皮质变薄，内部见斑点状钙化影；CT示囊性结构内壁分叶、多发小点状高密度影，局部骨膜反应形成Codman三角；MRI表现为T1低信号/T2混杂高信号，病变突破皮质侵犯周围软组织但未累及关节面，增强扫描呈环形强化。

最可能的3个诊断（由大到小）：

1. 骨巨细胞瘤（青少年股骨近端干骺端好发，溶骨性破坏伴轻度膨胀符合特征）
2. 低度恶性骨肉瘤（15岁发病高峰年龄，边界相对清晰但有软组织侵犯）
3. 动脉瘤样骨囊肿继发性改变（T2混杂信号及CT多房结构支持，需排除原发性）

临床建议：

1. 立即行左股骨病变穿刺活检（优先选择MRI显示的强化区域），送病理+免疫组化
2. 完善全身骨扫描和胸部CT排查转移灶
3. 请肿瘤科会诊制定后续治疗方案，若确诊恶性肿瘤需启动新辅助化疗前评估
