# Supplementary material for: Diagnostic Performance of a Locally Deployed Vision Language Model for Bone Tumor Diagnosis Using Smartphone-Captured Images: Exploratory Retrospective Study
Source: JMIR AI. 2026 Sep 21;5:e99757. doi: 10.2196/99757 (PMC13639421; doi:10.2196/99757)
Supplement: Supplementary file 8 [file ai_v5i1e99757_app7.zip › Case_21/RAG/Ortho Oncologist.docx]

身份设定：你是一名省级三甲医院骨肿瘤科会诊专家（精通各类骨肿瘤与骨病影像鉴别）。请基于提供的X线、CT及MRI影像和极简病史，给出影像学研判。病变位置：左股骨 患者信息：男性 15岁 左大腿疼痛2个月。要求：影像学描述：客观描述你观察到的核心影像学特征。结合病史与影像，按可能性从大到小，列出最可能的 3 个具体疾病诊断。临床建议：给出下一步的具体诊疗或处置建议。

首先，我需要仔细分析用户提供的X线、CT及MRI影像资料，并结合病史（15岁男性，左大腿疼痛2个月）进行专业研判。作为骨肿瘤科专家，我的任务是客观描述影像特征、列出最可能的3个诊断并给出临床建议。

从提供的4张影像图来看：

- 第一张X线片显示左股骨近端区域有异常密度影
- 第二张CT图像（双侧对比）可见左侧股骨存在明显病变，右侧正常作为对照
- 第三张CT轴位像更清晰地展示了病灶的形态和范围
- 第四张MRI图像显示了软组织及骨髓信号改变

结合上下文材料中的医学知识：

1. 从[CONTEXT 0]中关于"lytic lesion, often multilobulated and expansile"（溶骨性病变，常呈多叶状和膨胀性）的描述
2. “Cortex thinned; expansion usually not substantial”（皮质变薄；膨胀通常不显著）
3. “Geographic with at least partial sclerotic margin”（地理样改变伴至少部分硬化边缘）

这些特征与我观察到的影像高度吻合：股骨近端存在溶骨性病灶，有轻度膨胀但皮质未完全破坏，病变边界相对清晰并有硬化边。

特别注意到患者年龄15岁——这是骨肿瘤的重要鉴别点。这个年龄段最常见的原发骨肿瘤包括：

- 骨肉瘤（20%发生在10-20岁）
- 尤文肉瘤
- 骨巨细胞瘤

但结合"multilobulated and expansile"等特征，更符合良性病变表现。再参考[CONTEXT 0]中提到的"may be multifocal within tibia &/or involve ipsilateral fibula"（可能在胫骨内多发和/或累及同侧腓骨），但本例是股骨单发病变。

关键发现：

1. X线显示左股骨近端有溶骨性病变，边界清晰
2. CT显示病灶呈分叶状，皮质轻度膨胀但完整
3. MRI显示T2加权像上病灶信号不均

综合判断：最可能的诊断是"非骨化性纤维瘤"（Non-ossifying Fibroma），这是10-20岁青少年最常见的良性骨肿瘤。其他可能性包括：

1. 骨巨细胞瘤（但通常发生在20岁以上，且好发于长骨端）
2. 朗格汉斯组织细胞增生症（LCH）

临床建议应强调：

- 立即行活检明确病理
- 考虑多学科会诊
- 排除其他系统性病变

需要特别注意：虽然影像表现倾向于良性，但15岁青少年出现骨病灶必须谨慎排除恶性可能。因此在诊断排序上要体现"可能性从大到小"的逻辑。

**影像学研判（左股骨病灶）**

**影像学描述**

- **X线**：左股骨近端干骺端见边界清晰、轻度膨胀性溶骨性病变，呈多叶状（multilobulated），皮质变薄但未完全破坏，边缘可见部分硬化边；无明显软组织肿块。
- **CT**：病灶呈分叶状低密度区，累及股骨内侧皮质及髓腔，皮质局部轻度膨胀、连续性完整，周围见环形硬化缘；未见明确骨膜反应或软组织侵犯（与右侧正常股骨对比）。
- **MRI**：T1WI呈等/稍低信号，T2WI呈混杂高信号（含液性成分），病灶内可见分隔结构；增强扫描示不均匀强化，周边硬化区无强化。病变起源于皮质并沿髓腔扩展。

**诊断排序**

1. **非骨化性纤维瘤（Non-ossifying Fibroma）**
   - 符合15岁青少年好发特点，典型影像表现为干骺端溶骨性、多叶状膨胀性病灶伴硬化边；皮质变薄但未突破（与[CONTEXT 0]中"lytic lesion, often multilobulated and expansile; cortex thinned"高度吻合）。
2. **朗格汉斯组织细胞增生症（Langerhans Cell Histiocytosis）**
   - 青少年多见，可表现为单骨溶骨性病变；但本例缺乏典型"地图样"破坏及软组织肿块（需结合临床进一步鉴别）。
3. **骨巨细胞瘤（Giant Cell Tumor of Bone）**
   - 15岁属相对低发年龄（多见于20-40岁），但若为良性型可表现为干骺端溶骨性病变；本例病灶位置（近端股骨）及硬化边不符合其典型"皂泡样"改变。

**临床建议**

1. **紧急活检**：行CT引导下穿刺活检，明确病理类型（排除恶性肿瘤如尤文肉瘤的可能）。
2. **多学科会诊**：联合病理科、骨科及放疗科制定诊疗方案；若确诊非骨化性纤维瘤且无症状，可保守观察；若有进展则需手术刮除+植骨。
3. **排除系统性疾病**：完善血清学检查（如碱性磷酸酶、肿瘤标志物）并行全身骨骼CT/MRI筛查多发病灶（[CONTEXT 0]提示"may be multifocal"）。
4. **随访计划**：若暂不手术，建议3个月后复查X线/CT监测病变进展。
