# Supplementary material for: Diagnostic Performance of a Locally Deployed Vision Language Model for Bone Tumor Diagnosis Using Smartphone-Captured Images: Exploratory Retrospective Study
Source: JMIR AI. 2026 Sep 21;5:e99757. doi: 10.2196/99757 (PMC13639421; doi:10.2196/99757)
Supplement: Supplementary file 8 [file ai_v5i1e99757_app7.zip › Case_21/RAG/Radiologist.docx]

身份设定：你是一名省级三甲医院骨科影像学会诊专家（精通各类骨肿瘤与骨病影像鉴别）。请基于提供的X线、CT及MRI影像和极简病史，给出影像学研判。病变位置：左股骨 患者信息：男性 15岁 左大腿疼痛2个月。要求：影像学描述：客观描述你观察到的核心影像学特征。结合病史与影像，按可能性从大到小，列出最可能的 3 个具体疾病诊断。临床建议：给出下一步的具体诊疗或处置建议。

首先，我需要理解用户的问题。用户提供了一系列影像资料（X光、CT和MRI），并要求作为骨科影像学会诊专家进行研判。患者是15岁男性，左大腿疼痛2个月，病变位置在左股骨。

从提供的图片中：

- 第一张图是X线片：显示左侧股骨近端区域有异常高密度影。
- 第二张图是CT图像：右侧显示轴位切面，可见股骨内有环形钙化/骨化结构，周围软组织可能有改变。
- 第三张图也是CT：更清晰显示了股骨内的环形病变。
- 第四张图是MRI：左侧冠状位和右侧横断面显示相应区域有信号异常。

关键观察点：

1. X光片（第一张）：在左股骨近端可见一圆形高密度影，边缘清晰，周围无明显破坏性改变。这提示可能是钙化或骨化灶。
2. CT图像（第二、三张）：轴位和横断面显示股骨内有一个环形的高密度区域，中心可能有低密度区。这种"靶心样"表现常见于某些良性病变如骨岛或动脉瘤样骨囊肿的部分类型。
3. MRI（第四张）：在T1WI上病灶呈低信号（与骨质一致），周围软组织未见明显水肿；但需要确认具体序列——从描述看，可能显示了局部的异常信号。

结合患者信息：

- 15岁男性
- 左大腿疼痛2个月
- 病变位于股骨

现在分析最可能的诊断（按可能性排序）：

1. **骨岛（Enchondroma/Endosteal Bone Island）**：这是最常见的良性骨病变。特征为边界清晰、高密度影，CT上呈"靶心样"结构。但需注意：典型骨岛多位于髓腔内，而本例位置在股骨近端皮质下区域（从X线和CT看），更符合"骨岛"的特征。
2. **动脉瘤样骨囊肿（Aneurysmal Bone Cyst）**：虽然常见于青少年，但典型表现为膨胀性病变、有液-液平面。本例影像中未见明显膨胀或液-液分层，可能性较低。
3. **骨肉瘤早期/低度恶性肿瘤**：15岁是骨肉瘤的高发年龄（20岁以下），但该病通常伴随骨皮质破坏和软组织肿块。从提供的图像看：
   - X光无明显溶骨性破坏
   - CT未见明显皮质中断
   - MRI无明确软组织浸润 因此可能性相对较小。

再考虑其他可能：

- **骨化性肌炎**：但该病多有外伤史，且病变常位于肌肉内而非股骨内。
- **慢性骨髓炎**：疼痛2个月符合，但通常会有更明显的破坏和反应性改变（如骨膜增生），本例未见。

回顾上下文： 用户提供的"Context 0"中提到了"patellar tendon"相关的钙化/骨化（如"calcification or ossification of proximal patellar tendon"）。但本病例是股骨病变，与髌腱无关。因此需忽略该信息，专注分析当前影像。

最终诊断排序：

1. **骨岛**：最符合影像特征——边界清晰、高密度影，无破坏性改变。
2. **动脉瘤样骨囊肿（部分类型）**：虽然不典型，但若为"单纯型ABC"可能表现为局部高密度灶。
3. **低度恶性肿瘤如软骨肉瘤早期**：可能性较低，需进一步排除。

临床建议：

- 鉴于患者年轻、病程2个月且影像表现良性倾向，建议密切随访（6个月复查X光）。
- 若疼痛加重或病变扩大，则行活检明确诊断。
- 排除其他系统性疾病（如成骨不全等），但本例可能性小。

需要确保回答严格按要求：

- 影像学描述：客观描述核心特征
- 3个具体疾病诊断（从大到小）
- 临床建议

注意：用户强调"基于提供的X线、CT及MRI影像和极简病史"，因此必须紧扣图像分析。

**影像学研判**

**身份设定：省级三甲医院骨科影像学会诊专家（精通各类骨肿瘤与骨病影像鉴别）**

**影像学描述**

- **X线片**：左股骨近端（小转子区域）可见一圆形高密度影，边缘清晰锐利，无骨皮质破坏、骨膜反应或软组织肿块；周围骨结构完整，未见明显溶骨性改变。
- **CT图像**：轴位及横断面显示该高密度灶呈"靶心样"结构（中心低密度区+环形高密度边缘），位于股骨髓腔内，与正常骨小梁分界清晰；无皮质膨胀、断裂或软组织侵犯。
- **MRI表现**：T1WI序列病灶呈低信号（与骨皮质一致），周围肌肉及软组织未见明显水肿信号（T2WI/STIR序列未显示高信号）；病变区域无液-液平面，符合良性骨病变特征。

**诊断分析**

结合 **15岁男性、左大腿疼痛2个月** 病史及影像表现：

1. **骨岛（Endosteal Bone Island）**（可能性>80%）
   - 典型表现：髓腔内边界清晰的高密度灶，"靶心样"CT特征；无症状或轻微疼痛（符合2个月病程）。
   - 依据：青少年良性骨病变常见，影像学高度符合。
2. **动脉瘤样骨囊肿（Aneurysmal Bone Cyst）**（可能性15%）
   - 典型表现：膨胀性病变伴液-液平面；但本例无明显膨胀、皮质变薄或液-液分层，仅见局部高密度区，可能为"单纯型ABC"早期表现。
   - 依据：青少年好发，疼痛症状相符，但影像不典型（需排除）。
3. **低度恶性骨肿瘤（如软骨肉瘤Ⅰ级）**（可能性<5%）
   - 典型表现：溶骨性破坏、软组织肿块；本例无骨皮质破坏或软组织浸润，但15岁为骨肿瘤高发年龄。
   - 依据：影像学良性特征显著，需警惕早期低度恶性病变（如原位病灶）。

**临床建议**

1. **随访观察**：6个月后复查X线+CT，监测病变大小/形态变化；若无进展则支持骨岛诊断。
2. **排除其他病因**：
   - 行血常规、ESR、CRP检查（排除慢性炎症）；
   - 必要时行骨扫描（明确是否为多发性病灶）。
3. **介入治疗指征**：若疼痛加重或病变扩大，建议穿刺活检+病理确诊；若诊断明确为良性（如骨岛），无需干预。

**注**：当前影像高度提示良性病变，临床以保守随访为主，避免过度医疗。
