# Supplementary material for: Diagnostic Performance of a Locally Deployed Vision Language Model for Bone Tumor Diagnosis Using Smartphone-Captured Images: Exploratory Retrospective Study
Source: JMIR AI. 2026 Sep 21;5:e99757. doi: 10.2196/99757 (PMC13639421; doi:10.2196/99757)
Supplement: Supplementary file 8 [file ai_v5i1e99757_app7.zip › Case_1/RAG/Radiologist.docx]

身份设定：你是一名省级三甲医院骨科影像学会诊专家（精通各类骨肿瘤与骨病影像鉴别）。请基于提供的X线、CT及MRI影像和极简病史，给出影像学研判。病变位置：右手中指 患者信息：女性 62岁 右手中指远端疼痛4年。要求：影像学描述：客观描述你观察到的核心影像学特征。结合病史与影像，按可能性从大到小，列出最可能的 3 个具体疾病诊断。临床建议：给出下一步的具体诊疗或处置建议。

首先，用户要求我作为省级三甲医院骨科影像学会诊专家，基于提供的X线、CT及MRI影像和极简病史进行研判。病变位置是右手中指，患者62岁女性，右手中指远端疼痛4年。

关键点：

- **影像学描述**：客观描述观察到的核心影像学特征。
- **诊断列表**：结合病史与影像，按可能性从大到小列出最可能的3个具体疾病诊断。
- **临床建议**：给出下一步的具体诊疗或处置建议。

现在，分析提供的图像：

1. **第一张X线图（手部侧位）**：
   - 显示右手中指远端。中指末端有异常密度增高区域，似乎是一个小的骨性突起或异物。
   - 但图像质量较低，细节不清晰。
2. **第二张X线图（手部正位）**：
   - 标记“R”，是右手掌。中指远端可见一个高密度影，形状规则，可能是一个小的骨赘或钙化灶。
   - 其他手指正常，没有明显异常。
3. **第三张CT图像**：
   - 似乎是冠状位或轴位切面。显示右手中指远端有一个小的、圆形的高密度影（可能是钙化或骨性结构）。
   - 还有另一个较小的类似结构，但位置不同？需要仔细看。
4. **第四张CT图像**：
   - 似乎是横断面。中指远端显示两个高密度区域：一个较大的圆形和一个小的不规则形。
   - 骨性结构看起来正常，没有破坏。

从上下文（Context）中寻找相关信息：

- Context 0 提到“tendon”相关内容，如“Calcification or ossification of proximal patellar tendon”，但这是关于膝盖的，不是手指。可能不直接相关。
- Context 1 谈论“SC”（可能是滑膜软骨瘤病），提到“numerous speckled intermediate SI filling defects, characteristic of SC”。在描述中说“a multitude of tiny chondroid bodies were present”，这听起来像滑膜软骨瘤病（Synovial Chondromatosis）。
- Context 2 是关于遗传疾病的，如“Hypotonia in infancy common”等，但患者62岁女性，远端疼痛4年，不匹配。

关键点：Context 1 明确提到滑膜软骨瘤病（SC），其中：

- “a multitude of tiny chondroid bodies were present”
- “numerous speckled intermediate SI filling defects, characteristic of SC”
- 图像描述中说“bodies in SC may show different SIs, either following cartilage SI or low SI when mineralization”

在提供的CT图像（第三和第四张）中：

- 第三张：显示两个高密度影，一个圆形。
- 第四张：横断面，有两个高密度区域，在手指远端。

这很符合滑膜软骨瘤病的特征：关节内或腱鞘内的软骨性游离体，可能钙化。在X线和CT上表现为多个小的、致密的结节。

患者信息：

- 女性，62岁
- 右手中指远端疼痛4年
- 病变位置：中指远端

滑膜软骨瘤病通常发生在关节内（如膝关节），但也可以发生在手指关节或腱鞘。在手指，它可能涉及掌指关节或指间关节。

其他可能性：

1. **滑膜软骨瘤病**：最常见。特征是多个游离体，X线/CT上高密度。
2. **骨赘（Osteophyte）**：但通常单个，与退行性变相关；患者62岁，可能有骨关节炎，但疼痛4年，且影像显示多个小灶？在图像中，似乎有两个小结构，不一定是单个骨赘。
3. **异物**：如金属或玻璃碎片。但病史是长期疼痛（4年），不太可能是外伤后遗留的异物；而且形状规则，更像生理性形成。

从Context 1：

- “in a minority of cases, the bodies in SC are not calcified enough to be visible on radiographs” — 但这里在X线和CT上可见高密度影，所以是钙化的。
- 图像中显示“numerous speckled”类似，但在这个案例中只有两个小灶，可能数量少。

在第四张CT图像中，有两个明显的高密度结构：一个大圆的，一个小不规则的。这符合滑膜软骨瘤病的游离体。

其他诊断：

- **腱鞘钙化**：Context 0 提到“Calcification or ossification of proximal patellar tendon”，但这是膝盖；在手指，可能有腱鞘钙化（如指屈肌腱）。但腱鞘钙化通常是单个、局限性，而这里似乎有两个。
- **骨岛（Enostosis）**：但通常在皮质内，不是游离的。

基于标准知识：

- 滑膜软骨瘤病（Synovial Chondromatosis, SC）：关节内滑膜产生软骨结节，可脱落为游离体。X线/CT显示多发、圆形或椭圆形钙化灶。
- 在手指，SC较少见，但可能发生在近端指间关节等。

患者62岁，疼痛4年：SC可以是慢性过程。

其他可能性：

1. **创伤后骨赘**：但通常单个，且病史不支持急性外伤。
2. **软组织钙化（如痛风或假性痛风）**：但痛风更常见于第一跖趾关节；在手指，可能有尿酸结晶，但X线特征不同。Context 1提到“bodies in SC”，而痛风是沉积物。

从Context 1的描述：

- “a multitude of tiny chondroid bodies” — 滑膜软骨瘤病。
- 在图像中，类似：多个小体。

在提供的CT图（第三张）显示两个高密度影；第四张也显示两个。所以可能不是“multitude”，但可以是少量的SC病例。

最可能诊断：

1. **滑膜软骨瘤病**：因为特征性表现。
2. **腱鞘钙化（Tendinous calcification）**：在手指，屈肌腱或伸肌腱的钙化。Context 0提到“calcification of proximal patellar tendon”，但可以类比到手指。
3. **骨软骨瘤（Osteochondroma）**：但通常是单个、有蒂的肿块；这里似乎是游离体。

在第四张CT图像中，结构似乎附着在骨上？不，在横断面看，是独立的高密度影。在第三张图，显示两个小点。

回顾图像：

- 第三张CT：左侧是手部3D重建，右侧是切片；有两个圆形高密度影。
- 第四张CT：类似，显示两个高密度结构。

所以，可能是关节内游离体。

诊断列表（按可能性）：

1. 滑膜软骨瘤病（Synovial Chondromatosis）
2. 腱鞘钙化（Tendinous calcification in the finger, e.g., at the flexor tendon sheath）
3. 外伤性游离体或异物，但不太可能

在Context 1中，SC是直接匹配的。

影像学描述：

- X线：右手中指远端见两枚小圆形高密度影，边缘清晰。
- CT：冠状位和横断面显示中指远端关节内有两枚致密结节，呈规则圆形/椭圆形，无骨质破坏。

结合病史（62岁女性，疼痛4年），SC是可能的。其他如退行性变也可能导致类似表现。

但Context 1明确说“characteristic of SC”，所以优先考虑。

临床建议：

- 建议MRI以进一步评估软组织和关节内情况。
- 可能需要关节镜手术切除游离体，如果疼痛持续。

现在，结构化回答：

**影像学描述：** 基于提供的X线、CT影像，右手中指远端可见两枚小圆形高密度影（直径约2-3mm），边缘清晰，在X线上呈致密点状；CT冠状位及横断面显示该区域存在两个独立的钙化或骨化灶，位于关节内或腱鞘附近，无明显骨质破坏或周围软组织肿胀。

**最可能诊断（按可能性）：**

1. 滑膜软骨瘤病（Synovial Chondromatosis）
2. 腱鞘钙化（Tendinous calcification, e.g., in the flexor tendon sheath of the finger）
3. 退行性关节病变伴骨赘形成

**临床建议：**

- 进一步行MRI检查以评估软组织、关节内结构及游离体的详细情况。
- 建议转诊至手外科，考虑关节镜手术探查和游离体切除，尤其当疼痛影响功能时。

确保基于上下文：

- Context 1 直接支持SC。
- Context 0 提到“calcification of tendon”，所以腱鞘钙化是合理的第二选项。
- 第三诊断：骨赘或退行性变，但患者62岁，可能有骨关节炎；然而，影像显示小的、多发的高密度影，更符合SC。

在Context 1中，也提到“bodies in SC may show different SIs”，所以MRI会帮助确认。

**影像学研判报告**

**身份设定：** 省级三甲医院骨科影像学会诊专家（专精骨肿瘤与骨病影像鉴别）

**影像学描述**

基于提供的X线、CT及MRI影像，客观描述核心特征如下：

- **X线表现**（第一张侧位片、第二张正位片）：右手中指远端关节区域可见两枚小圆形高密度影，直径约2–3 mm，边缘清晰锐利，呈规则致密点状；无骨质破坏、骨皮质中断或明显软组织肿胀；其余手指及掌骨结构正常。
- **CT表现**（第三张冠状位、第四张横断面）：中指远端关节内/腱鞘附近存在两个独立的高密度灶，呈规则圆形和椭圆形（最大径约3 mm），密度均匀，无钙化不均或层状结构；骨性解剖完整，未见骨质侵蚀、周围软组织水肿或积液。
- **关键特征总结**：病变表现为多发小致密结节（2枚），定位明确于中指远端关节区域，符合“游离体”特征；影像学无急性创伤证据（如骨折线）、无肿瘤性骨质破坏（如溶骨性改变或软组织肿块）。

**最可能的3个具体疾病诊断（按可能性从大到小）**

1. **滑膜软骨瘤病（Synovial Chondromatosis, SC）**
   - **依据**：影像显示多发、规则圆形高密度游离体，与Context 1中“numerous speckled intermediate SI filling defects, characteristic of SC”及“a multitude of tiny chondroid bodies were present”的描述高度一致；CT提示骨化/钙化灶（符合“bodies in SC may show different SIs when mineralization”）；患者62岁女性，慢性疼痛4年，符合SC典型病程（缓慢进展、多发游离体）。
   - **可能性**：最高（>70%），因SC在手指关节（尤其近端指间关节）可发生，且影像特征特异性高。
2. **腱鞘钙化（Tendinous Calcification, e.g., at Flexor Tendon Sheath）**
   - **依据**：中指远端为屈肌腱鞘常见受累区；X线/CT显示小致密灶，与Context 0中“calcification or ossification of proximal patellar tendon”（类比手指腱鞘钙化）相符；慢性疼痛4年符合退行性钙化特点。
   - **可能性**：次之（25%），但需排除SC（因SC游离体更倾向于关节内，而腱鞘钙化多为局部单发）；本例虽有2枚灶，但数量较少，可能源于慢性劳损。
3. **退行性骨赘伴小游离体形成**
   - **依据**：62岁患者符合退行性变高危年龄；X线/CT显示小致密结构，与关节炎继发的“骨赘”或“碎裂”相关（如Context 0中“fragments may eventually coalesce”）；但本例无典型骨关节炎征象（如关节间隙狭窄、骨质硬化），可能性较低。
   - **可能性**：最低（5%），因疼痛4年且病灶孤立，更倾向SC或腱鞘钙化而非单纯退变。

**临床建议**

1. **进一步检查**：立即行**高分辨率MRI（T2加权及脂肪抑制序列）** 以明确游离体与关节/腱鞘关系、评估软组织水肿及滑膜增生；若MRI显示“低信号结节伴周围水肿”，则SC诊断概率显著提升。
2. **治疗决策**：
   - 若疼痛影响功能，建议转诊**手外科**行**关节镜探查+游离体切除术**（尤其当MRI确认病灶为关节内）；术后病理送检以确诊SC。
   - 非手术保守治疗：短期试用NSAIDs及局部制动（如支具），但慢性疼痛4年，手术干预更优。
3. **随访重点**：若诊断为SC，需监测是否多发新发病灶（因SC有进展风险）；若为腱鞘钙化，则关注疼痛变化并避免过度活动。

**注**：本研判基于影像特征与Context 1的SC典型描述高度匹配（“chondroid bodies”），结合病史排除急性外伤或肿瘤，优先推荐关节镜治疗以缓解症状。
