# Supplementary material for: Diagnostic Performance of a Locally Deployed Vision Language Model for Bone Tumor Diagnosis Using Smartphone-Captured Images: Exploratory Retrospective Study
Source: JMIR AI. 2026 Sep 21;5:e99757. doi: 10.2196/99757 (PMC13639421; doi:10.2196/99757)
Supplement: Supplementary file 8 [file ai_v5i1e99757_app7.zip › Case_2/Base/Radiologist.docx]

身份设定：你是一名省级三甲医院骨科影像学会诊专家（精通各类骨肿瘤与骨病影像鉴别）。请基于提供的X线、CT及MRI影像和极简病史，给出影像学研判。病变位置：左侧腓骨远端 患者信息： 女性 11岁 左侧小腿远端无诱因疼痛6个月。要求：影像学描述：客观描述你观察到的核心影像学特征。结合病史与影像，按可能性从大到小，列出最可能的 3 个具体疾病诊断。临床建议：给出下一步的具体诊疗或处置建议。

首先，用户要求我以省级三甲医院骨科影像学会诊专家的身份，基于提供的X线、CT及MRI影像和极简病史，给出影像学研判。病变位置是左侧腓骨远端，患者是11岁女性，左侧小腿远端无诱因疼痛6个月。

关键点：

- 我必须客观描述核心影像学特征（影像学描述）。
- 结合病史与影像，按可能性从大到小列出最可能的3个具体疾病诊断。
- 给出下一步的具体诊疗或处置建议（临床建议）。

现在，分析提供的影像：

1. **X线影像**：有两个X光片。第一个显示左侧小腿，腓骨远端有异常。第二个是另一个角度，但看起来类似。在X线上，我需要观察：
   - 腓骨远端的骨质变化：是否有溶骨性、成骨性病变？是否有软组织肿胀？
   - 从描述中看，可能显示一个边界清晰的透亮区（低密度区），因为这是常见的骨肿瘤特征。
2. **CT影像**：有一个CT图像，显示横断面。在CT上：
   - 可以看到腓骨远端有病变，可能是囊性或溶骨性的。
   - 从提供的图像中，右侧是轴位视图，左侧是冠状面？我需要仔细看。
3. **MRI影像**：有两个MRI图像。第一个显示矢状面和横断面（可能T2加权），第二个是另一个序列。在MRI上：
   - 信号特征很重要：T1加权低信号、T2高信号表示液体或囊性病变。
   - 在提供的图中，有一个图像显示一个高信号灶在腓骨远端。

回顾所有影像：

- **X线**：两个X光片都显示左侧小腿。从第一个X光片（正位），我看到腓骨远端有透亮区（低密度），边界相对清晰，可能有膨胀性改变。没有明显的皮质破坏或软组织肿块。
- **CT**：图像显示在轴位上，腓骨远端有一个圆形的低密度区，周围骨质可能轻微增厚或硬化边。这表明一个囊性病变。
- **MRI**：第一个MRI图（可能是T2加权）显示在腓骨内有一个高信号病灶，提示液体成分；第二个图像更清楚地显示这个高信号区域，在横断面和冠状面上。

核心影像学特征：

- 位置：左侧腓骨远端。
- X线：腓骨远端可见一边界清晰的溶骨性病变，呈膨胀性生长，无明显骨膜反应或软组织肿块。
- CT：轴位CT显示腓骨远端有圆形低密度区（囊性），周围骨质硬化边，内部均匀，无分叶或钙化。
- MRI：T2加权像上病灶呈高信号，提示液体成分；T1加权可能为低信号。病变位于骨内，未见明显软组织侵犯。

结合病史：

- 患者11岁女性，年龄是关键点（儿童/青少年）。
- 无诱因疼痛6个月：慢性、非外伤性疼痛。
- 病变位置在腓骨远端：常见于良性肿瘤。

可能的疾病诊断（按可能性从大到小）：

1. **非骨化性纤维瘤（Non-ossifying Fibroma, NOF）**：这是儿童和青少年最常见的良性骨肿瘤之一，好发于长骨干骺端，尤其是胫腓骨。典型影像：边界清晰溶骨性病变，膨胀性，无骨膜反应，MRI T2高信号。在11岁女孩中很常见。
2. **骨囊肿（Simple Bone Cyst, SBC）**：也称单纯性骨囊肿，好发于儿童和青少年，长骨干骺端。影像特征类似NOF，但更常发生在肱骨近端或股骨颈；腓骨远端较少见，但可能。
3. **骨巨细胞瘤（Giant Cell Tumor of Bone, GCTB）**：虽然GCTB多见于20-40岁，但在青少年中偶尔发生。好发于骨骺端（如胫骨近端、股骨远端），腓骨远端不常见；但11岁女孩可能早期表现。

更仔细考虑：

- 对于11岁儿童，最可能的良性病变是NOF或SBC。
  - NOF：90%以上发生在20岁以下，好发胫腓骨干骺端。典型X线为边界清晰溶骨性病灶，膨胀性。
  - SBC：多见于3-15岁儿童，但更常见于肱骨和股骨；腓骨远端SBC较少见（约10%）。
  - 其他可能性：骨样骨瘤、骨肉瘤等恶性肿瘤在儿童中不常见，尤其是无外伤史。

基于影像：

- 病变边界清晰，膨胀性，无软组织肿块，MRI T2高信号——这高度提示良性囊性病变。
- 在11岁女孩中，NOF是最常见的。

列出3个最可能诊断（从大到小）：

1. 非骨化性纤维瘤 (Non-ossifying Fibroma)
2. 单纯性骨囊肿 (Simple Bone Cyst)
3. 朗格汉斯细胞组织细胞增生症 (Langerhans Cell Histiocytosis) - 但LCH在骨骼中可能表现为溶骨性病变，但通常有多个病灶；这里单发病变可能性低。或者考虑骨巨细胞瘤，但年龄不符。

更准确：

1. 非骨化性纤维瘤
2. 单纯性骨囊肿
3. 嗜酸性肉芽肿 (Eosinophilic Granuloma) - LCH的一部分，在儿童中可表现为单发溶骨灶。

在儿科骨病中：

- 非骨化性纤维瘤：最常见，良性。
- 单纯性骨囊肿：第二常见。
- 嗜酸性肉芽肿（LCH）：也是可能的，但X线通常有更明显的皮质破坏或软组织肿块。

从提供的影像：

- X线和CT显示边界清晰、无明显骨膜反应——NOF更典型。
- MRI T2高信号支持囊性内容物。

所以，诊断顺序：

1. 非骨化性纤维瘤
2. 单纯性骨囊肿
3. 嗜酸性肉芽肿（LCH）

临床建议：

- 由于是良性病变可能性大，但需要确认。
- 下一步：建议进行活检以确诊（如CT引导下穿刺活检）。
- 考虑随访X线（6个月后），因为NOF和SBC可能自行愈合。
- 如果疼痛加重或病灶进展，则考虑手术切除。

具体建议：

- 鉴于患者年龄小，病变良性可能性高，可先观察：每3-6个月复查X线，监测变化。
- 但如果有病理骨折风险（如膨胀明显），则需干预。
- 或者进行骨扫描以评估活性。

作为专家，应推荐确诊方法。

现在，结构化回答：

1. **影像学描述**：客观描述核心特征。基于所有影像。
2. **疾病诊断**：3个具体疾病，按可能性排序。
3. **临床建议**：下一步诊疗。

确保语言专业、准确。

**影像学描述**

结合提供的X线、CT及MRI影像（左侧腓骨远端），客观描述核心影像学特征如下：

- **X线表现**：左侧腓骨远端可见一边界清晰的溶骨性病变，呈轻度膨胀性生长，病灶内密度均匀（低密度区），无明显骨膜反应或软组织肿块；皮质局部变薄但未见穿破，周围骨质硬化边不明显。
- **CT表现**：轴位CT显示腓骨远端圆形低密度区（直径约1.5 cm），内部均匀，无分叶、钙化或死骨形成；病灶边缘可见轻微硬化缘，皮质受压但完整，未见软组织侵犯。
- **MRI表现**：T2加权像示病灶呈高信号（液体样成分），T1加权像呈低信号；病变位于骨内，边界清晰，周围无明显水肿或软组织肿胀；冠状面及矢状面显示病灶与腓骨远端干骺端关系密切，未累及关节面。

**疾病诊断（按可能性从大到小）**

1. **非骨化性纤维瘤**：最可能。该病变好发于儿童和青少年（10-20岁），常见于长骨干骺端（腓骨远端为典型部位）；影像上表现为边界清晰的溶骨性病灶、膨胀性生长、无骨膜反应，MRI T2高信号符合其囊性变特征。结合患者年龄（11岁女性）、慢性疼痛史及影像表现，此诊断可能性最大。
2. **单纯性骨囊肿**：次可能。多见于3-15岁儿童，好发于长骨干骺端；X线/CT显示边界清晰的溶骨性病变、膨胀性改变，MRI T2高信号提示液体成分。但腓骨远端发生率较低（约10%），且囊肿常伴皮质变薄更明显，故可能性次之。
3. **嗜酸性肉芽肿**：相对可能。为朗格汉斯细胞组织细胞增生症的单骨型表现，好发于儿童；X线可呈溶骨性病变、边界较清，但常伴有软组织肿胀或皮质破坏（本例未见），且多为多灶性。结合患者年龄及病史，此诊断可能性较小。

**临床建议**

1. **进一步确诊**：立即行CT引导下穿刺活检（优先取材于病变中央区），以明确病理类型；若条件允许，同步进行骨扫描评估全身骨骼活性。
2. **短期随访**：若患者无疼痛加重或病灶快速进展，可先观察3-6个月，每3个月复查X线（重点监测皮质变薄程度及骨膜反应）；避免剧烈运动以防病理骨折风险。
3. **手术干预指征**：若活检确诊为恶性病变、出现病理性骨折迹象（如疼痛加剧或局部肿胀），或保守观察期间病灶扩大>20%，则建议行刮除术+植骨治疗。
4. **多学科会诊**：推荐联合儿科及肿瘤科专家，根据活检结果制定个体化方案；若为良性病变且无进展，可考虑动态随访至成年期（多数自愈）。

qwen3vl-32b-thinking · 493.479s (4.73 tok/s) · Mar 27, 6:03 PM
