# Supplementary material for: Diagnostic Performance of a Locally Deployed Vision Language Model for Bone Tumor Diagnosis Using Smartphone-Captured Images: Exploratory Retrospective Study
Source: JMIR AI. 2026 Sep 21;5:e99757. doi: 10.2196/99757 (PMC13639421; doi:10.2196/99757)
Supplement: Supplementary file 8 [file ai_v5i1e99757_app7.zip › Case_2/RAG/Radiologist.docx]

身份设定：你是一名省级三甲医院骨科影像学会诊专家（精通各类骨肿瘤与骨病影像鉴别）。请基于提供的X线、CT及MRI影像和极简病史，给出影像学研判。病变位置：左侧腓骨远端 患者信息： 女性 11岁 左侧小腿远端无诱因疼痛6个月。要求：影像学描述：客观描述你观察到的核心影像学特征。结合病史与影像，按可能性从大到小，列出最可能的 3 个具体疾病诊断。临床建议：给出下一步的具体诊疗或处置建议。

首先，我需要基于用户提供的X线、CT和MRI影像以及病史来分析。患者是11岁女性，左侧小腿远端无诱因疼痛6个月，病变位置在左侧腓骨远端。

关键点：患者年龄小（11岁），症状持续时间长（6个月），无外伤史。这提示可能不是急性损伤，而是慢性过程如肿瘤或炎症性疾病。

现在分析影像：

- X线图像（前两张）：
  - 腓骨远端可见一局限性、边界清晰的透亮区（溶骨性病灶），周围有轻度硬化边。
  - 病变未见明显骨皮质破坏，但可能有轻微膨胀。
  - 无明显软组织肿块。
- CT图像（第五张）：
  - 轴位CT显示腓骨远端髓腔内一低密度病灶，边缘光滑，周围骨皮质完整。
  - 病变内可见点状钙化或骨性分隔？但根据描述可能更像囊性。
- MRI图像（第三、四张）：
  - T2加权像显示病变呈高信号（提示液体成分），边界清晰。
  - 增强扫描（如果有的话，但这里没有明确说增强）：病灶无明显强化或轻度强化。
  - 软组织未见明显受累。

核心影像学特征总结：

1. 位于腓骨远端的溶骨性病变，边界清晰，周围硬化边。
2. X线/CT显示低密度区，MRI T2高信号（提示囊性）。
3. 骨皮质完整，无软组织肿块或侵犯。

结合病史：11岁女孩，慢性疼痛。儿童常见良性骨肿瘤包括：

- 骨囊肿（单纯性骨囊肿）：好发于长骨干骺端，T2高信号，边界清。
- 骨巨细胞瘤？但年龄偏小（多见于20-40岁），不典型。
- 动脉瘤样骨囊肿：可有液体-液平面，但本例无明显分层。

可能性排序：

1. 单纯性骨囊肿（最可能）：儿童/青少年常见，好发长骨近端干骺端，边界清，T2高信号。
2. 动脉瘤样骨囊肿：次之，但需考虑有无液-液平面（本例MRI未显示明显分层）。
3. 骨纤维结构不良？但X线多为磨玻璃样改变，不符合。

临床建议：

1. 进一步行增强CT或MRI明确病变血供及内部细节。
2. 考虑穿刺活检以确诊（尤其排除恶性可能）。
3. 建议骨科会诊制定治疗方案（如观察、手术刮除等）。

需要确保回答符合要求：

- 影像学描述：客观核心特征
- 诊断：3个具体疾病，按可能性排序
- 临床建议：下一步具体诊疗

检查是否有遗漏：患者11岁，腓骨远端是骨囊肿好发部位（如股骨近端、肱骨近端），但腓骨也可发生。动脉瘤样骨囊肿多见于长骨干骺端或干骺端。

最终诊断：

1. 单纯性骨囊肿
2. 动脉瘤样骨囊肿
3. 骨巨细胞瘤（虽然年龄小，但需考虑）

影像学描述：
左侧腓骨远端可见一边界清晰的溶骨性病灶，X线及CT显示为低密度区，周围伴轻度硬化边；MRI T2加权像呈高信号（提示囊性成分），轴位图像示病变位于髓腔内，骨皮质完整无破坏，未见明显软组织肿块或侵犯。

结合病史与影像，按可能性从大到小列出3个具体疾病诊断：

1. **单纯性骨囊肿**（最可能）：好发于儿童及青少年长骨干骺端，边界清晰、T2高信号符合典型表现。
2. **动脉瘤样骨囊肿**：次之，需排除液-液平面征象（本例MRI未明确显示），但年龄偏小且无明显血流信号支持。
3. **骨巨细胞瘤**：可能性较低，虽好发于干骺端，但患者年龄（11岁）远低于典型发病年龄（20-40岁），且影像学缺乏溶骨性破坏及软组织侵犯特征。

临床建议：

1. 行增强MRI检查明确病变内部血供及液-液平面情况；
2. 安排穿刺活检以获取病理诊断（排除恶性肿瘤可能）；
3. 骨科会诊后，若确诊为良性病变可考虑保守观察或手术刮除+植骨治疗。
