# Supplementary material for: Diagnostic Performance of a Locally Deployed Vision Language Model for Bone Tumor Diagnosis Using Smartphone-Captured Images: Exploratory Retrospective Study
Source: JMIR AI. 2026 Sep 21;5:e99757. doi: 10.2196/99757 (PMC13639421; doi:10.2196/99757)
Supplement: Supplementary file 8 [file ai_v5i1e99757_app7.zip › Case_3/Base/Ortho Oncologist.docx]

身份设定：你是一名省级三甲医院骨肿瘤科会诊专家（精通各类骨肿瘤与骨病影像鉴别）。请基于提供的X线、CT及MRI影像和极简病史，给出影像学研判。病变位置：左胫骨 患者信息：女性 6岁 左小腿无明显诱因疼痛5天。要求：影像学描述：客观描述你观察到的核心影像学特征。结合病史与影像，按可能性从大到小，列出最可能的 3 个具体疾病诊断。临床建议：给出下一步的具体诊疗或处置建议。

首先，我需要仔细分析提供的X线、CT及MRI影像，并结合病史来做出研判。患者是6岁女性，左小腿无明显诱因疼痛5天。

**1. 影像学描述（客观描述核心特征）：**

- **X线片（第1、2张图）：**
  - 左胫骨中段可见一圆形/椭圆形透亮区，边界相对清晰。
  - 病变区域无明显骨膜反应或软组织肿块。
  - 骨皮质局部轻度膨胀，但未见病理性骨折。
- **CT扫描（第4、5张图）：**
  - 轴位CT显示左胫骨中段有低密度区，内部可见点状钙化或骨性分隔。
  - 病变边界清晰，与周围正常骨组织对比明显。
  - 骨皮质变薄但连续，无明显破坏。
- **MRI（第3张图）：**
  - T2加权像显示病变区呈高信号，提示液体成分或富含水分的组织。
  - 病变中央有低信号区，可能为纤维分隔或钙化。
  - 周围软组织无明显水肿。

**2. 结合病史与影像分析：**

- **关键点：**
  - 患者6岁（儿童期），左小腿疼痛5天，无外伤史。
  - 影像学表现为胫骨中段的囊性病变，边界清晰、密度/信号不均，提示良性过程。

**3. 可能疾病诊断（按可能性排序）：**

1. **非骨化性纤维瘤（NOF）**：
   - 最常见于儿童和青少年，好发于长骨干骺端。
   - X线表现为边界清晰的透亮区，常伴骨皮质膨胀；CT/MRI显示囊性改变、内部分隔。
   - 本例符合：6岁女童、胫骨中段病灶、无明显恶性征象。
2. **单纯性骨囊肿（SC）**：
   - 儿童期多见，好发于长骨干骺端/干骺端交界处。
   - X线为边界清晰的透亮区；MRI呈T1低信号、T2高信号（液体成分），本例T2高信号符合。
3. **骨巨细胞瘤（GCT）**：
   - 虽罕见于6岁儿童，但需排除。GCT多见于20-40岁，好发于骨骺端。
   - 本例病灶位置偏干骺端、无明显骨膜反应或软组织侵犯，可能性较低。

（注：其他如骨肉瘤、尤文肉瘤等恶性肿瘤在6岁儿童中罕见且影像表现不符。）

**4. 临床建议：**

- **下一步诊疗：**
  - 建议行穿刺活检以明确病理诊断。
  - 若确诊为良性病变（如NOF），可考虑保守治疗或刮除植骨术；若需手术，应由骨肿瘤专科医生评估。

**最终回答结构：**

1. 影像学描述：客观、简洁地总结核心特征。
2. 3个最可能诊断：按可能性排序，并简要说明依据。
3. 临床建议：具体、可操作的下一步措施。

影像学描述：左胫骨中段可见边界清晰的圆形/椭圆形透亮区，X线显示局部骨皮质轻度膨胀但连续；CT轴位示病变内部有低密度区伴点状钙化或骨性分隔，骨皮质变薄未见破坏；MRI（T2加权）呈高信号，中央可见低信号分隔，周围软组织无水肿。

结合病史与影像，按可能性从大到小的3个具体疾病诊断：

1. 非骨化性纤维瘤（6岁儿童好发于胫骨干骺端，边界清晰透亮区伴骨皮质膨胀，CT/MRI符合囊性改变特征）。
2. 单纯性骨囊肿（儿童期常见，X线及MRI表现为界限清楚的液性病灶，T2高信号支持液体成分）。
3. 骨巨细胞瘤（罕见于6岁患儿，但需排除：虽好发于骨骺端且本例位置偏干骺端，无明显恶性征象如软组织侵犯或骨膜反应）。

临床建议：立即行CT引导下穿刺活检明确病理诊断；若确诊良性病变（如非骨化性纤维瘤），建议由骨肿瘤专科医生评估保守治疗或刮除植骨术方案。

qwen3vl-32b-thinking · 344.847s (2.88 tok/s) · Mar 29, 5:33 PM
