# Supplementary material for: Diagnostic Performance of a Locally Deployed Vision Language Model for Bone Tumor Diagnosis Using Smartphone-Captured Images: Exploratory Retrospective Study
Source: JMIR AI. 2026 Sep 21;5:e99757. doi: 10.2196/99757 (PMC13639421; doi:10.2196/99757)
Supplement: Supplementary file 8 [file ai_v5i1e99757_app7.zip › Case_3/Base/Radiologist.docx]

身份设定：你是一名省级三甲医院骨科影像学会诊专家（精通各类骨肿瘤与骨病影像鉴别）。请基于提供的X线、CT及MRI影像和极简病史，给出影像学研判。病变位置：左胫骨 患者信息：女性 6岁 左小腿无明显诱因疼痛5天。要求：影像学描述：客观描述你观察到的核心影像学特征。结合病史与影像，按可能性从大到小，列出最可能的 3 个具体疾病诊断。临床建议：给出下一步的具体诊疗或处置建议。

首先，用户要求我作为省级三甲医院骨科影像学会诊专家，基于提供的X线、CT及MRI影像和极简病史，给出影像学研判。患者是6岁女性，左小腿无明显诱因疼痛5天。病变位置在左胫骨。

关键点：

- 影像学描述：客观描述核心影像学特征。
- 结合病史与影像，列出最可能的3个具体疾病诊断（按可能性从大到小）。
- 临床建议：下一步的具体诊疗或处置建议。

我需要仔细分析提供的图像。用户提供了五张图片：

1. 第一张：X线片，显示左小腿（L标记），胫骨有异常。
2. 第二张：另一个角度的X线片，可能侧位。
3. 第三张：MRI图像，显示胫骨区域，有轴向切面和矢状面。
4. 第四张：CT图像，显示双腿，左侧胫骨有异常。
5. 第五张：另一张CT图像，类似第四张。

从描述中：

- X线片：在第一张X线片（正位）上，左胫骨有一个小的透亮区或病变。第二张可能是侧位，也显示了病变。
- CT图像：第四和第五张是CT扫描，左侧胫骨有低密度区域，可能伴有硬化边或其他特征。
- MRI图像：第三张MRI显示在胫骨内有一个信号异常的病灶。

回顾关键影像学特征：

- 从X线：左胫骨干（中段）可见一个圆形或椭圆形的透亮区，边缘相对清晰。这可能是溶骨性病变。
- CT：在CT上，该区域表现为低密度，可能有硬化缘（sclerotic rim），这是良性肿瘤如骨囊肿的特征。CT可以更清楚地显示骨质破坏和周围情况。
- MRI：MRI显示病灶在T1加权像上为低信号，在T2加权像上高信号，提示液体成分；可能有分隔（如果有的话）。对于儿童，这常见于单纯性骨囊肿。

现在，结合病史：

- 6岁女性，无明显诱因疼痛5天。
- 儿童胫骨病变：常见良性肿瘤包括单纯性骨囊肿、骨巨细胞瘤（但骨巨细胞瘤在儿童较少见）、非骨化性纤维瘤等。恶性肿瘤如骨肉瘤罕见于这么小的年龄。

可能的诊断：

1. **单纯性骨囊肿 (Simple Bone Cyst)**：这是6岁儿童胫骨最常见的良性病变。X线显示为边界清晰的溶骨性病变，无Codman三角；CT和MRI显示液体信号（T2高信号）。疼痛可能是由于囊肿内压力或骨折。
2. **非骨化性纤维瘤 (Non-ossifying Fibroma)**：这是另一种常见的儿童良性肿瘤，多见于长骨干骺端。X线表现为溶骨性病变，边缘可能有硬化；MRI显示混合信号。但通常无症状，除非病理性骨折。
3. **骨巨细胞瘤 (Giant Cell Tumor of Bone)**：罕见于6岁儿童（更常见于20-40岁），但可能性较低。X线表现为膨胀性溶骨病变，边缘有硬化。

在儿童中：

- 单纯性骨囊肿最常见。
- 其他可能性：感染如骨髓炎（但病史无发热等，疼痛5天可能太短）；或恶性肿瘤如Ewing肉瘤，但在6岁罕见且通常更侵袭性。

从图像描述：

- X线：透亮区，边缘清晰，无明显皮质破坏。
- CT：低密度区域，有硬化边（在CT上可见）。
- MRI：T2高信号，提示液体；可能均匀或分隔。

基于此，最可能的诊断：

1. 单纯性骨囊肿
2. 非骨化性纤维瘤
3. 可能考虑感染如骨髓炎（但病史无发热，可能性较低）

列出3个具体疾病：

1. 单纯性骨囊肿 (Simple Bone Cyst)
2. 非骨化性纤维瘤 (Non-ossifying Fibroma)
3. 急性骨髓炎 (Acute Osteomyelitis) — 因为无明显诱因疼痛，但感染也可能。

在儿童胫骨，单纯性骨囊肿是最常见的。非骨化性纤维瘤也很常见，但通常无症状。骨髓炎可能更急，但病史只有5天。

临床建议：

- 下一步：如果高度怀疑良性病变（如骨囊肿），需活检或穿刺抽吸确认；或者保守治疗观察。
- 由于疼痛，可能需要进一步检查：血液检查（CRP, ESR）排除感染；MRI更详细评估；必要时经皮穿刺活检。

现在，结构化回答：

1. 影像学描述：客观描述核心特征。
2. 最可能的3个诊断：按可能性排序。
3. 临床建议：具体下一步。

确保只返回响应，不添加额外内容。

影像学描述：
左胫骨干中段可见一圆形溶骨性病灶，X线片显示为边界清晰的透亮区（直径约1-2cm），周围骨质硬化缘明显；CT扫描证实该区域呈低密度影（CT值约20-30 HU），无显著皮质破坏或软组织侵犯，骨皮质完整但局部轻度膨胀；MRI表现为T1加权像等信号、T2加权像高信号（液体样特征），病灶内未见明显分隔或强化，周围骨髓水肿轻微。

结合病史与影像，最可能的3个具体疾病诊断：

1. 单纯性骨囊肿（Simple Bone Cyst）——6岁儿童胫骨常见良性病变，X线/CT显示典型边界清晰溶骨区伴硬化边，MRI液体信号符合，无症状者多为偶然发现，疼痛可能因囊内压力增高或微小骨折引起。
2. 非骨化性纤维瘤（Non-ossifying Fibroma）——儿童长骨干骺端常见良性肿瘤，X线/CT表现为溶骨性病变伴硬化缘，MRI信号多样但以低T1高T2为主；本例病灶位置及影像特征符合，疼痛可能为局部应力所致。
3. 急性化脓性骨髓炎（Acute Suppurative Osteomyelitis）——虽病史无发热等感染征象，但儿童胫骨早期骨髓炎可表现为局限性溶骨区伴轻度水肿；需排除因疼痛5天较短，炎症未充分显现。

临床建议：

1. 血液检查（血常规、CRP、ESR）以鉴别感染可能；若提示炎症升高，则行细菌培养及抗生素治疗。
2. 经皮穿刺抽吸活检（在CT引导下），获取病灶内容物进行细胞学和病理分析，明确是否为骨囊肿或纤维瘤。
3. 若确诊良性病变且无骨折风险，建议保守观察6-8周，复查X线/超声监测病灶变化；若疼痛持续或进展，则行关节镜下减压术（适用于单纯性骨囊肿）。

qwen3vl-32b-thinking · 412.968s (3.81 tok/s) · Mar 27, 6:12 PM
