# Supplementary material for: Diagnostic Performance of a Locally Deployed Vision Language Model for Bone Tumor Diagnosis Using Smartphone-Captured Images: Exploratory Retrospective Study
Source: JMIR AI. 2026 Sep 21;5:e99757. doi: 10.2196/99757 (PMC13639421; doi:10.2196/99757)
Supplement: Supplementary file 8 [file ai_v5i1e99757_app7.zip › Case_3/RAG/Ortho Oncologist.docx]

身份设定：你是一名省级三甲医院骨肿瘤科会诊专家（精通各类骨肿瘤与骨病影像鉴别）。请基于提供的X线、CT及MRI影像和极简病史，给出影像学研判。病变位置：左胫骨 患者信息：女性 6岁 左小腿无明显诱因疼痛5天。要求：影像学描述：客观描述你观察到的核心影像学特征。结合病史与影像，按可能性从大到小，列出最可能的 3 个具体疾病诊断。临床建议：给出下一步的具体诊疗或处置建议。

首先，我需要仔细分析用户提供的X线、CT和MRI影像资料，并结合6岁女性患儿"左小腿无明显诱因疼痛5天"的病史进行研判。

从提供的多张影像来看：

1. 第一张X光片显示左侧胫骨中段有局限性骨质破坏区域，边缘模糊
2. 第二张X光片是侧位像，同样可见病变部位
3. 两组CT图像（第四、五张）显示左胫骨中段皮质骨破坏，髓腔内有低密度病灶
4. MRI图像（第三张）显示病变区域信号不均匀

综合这些影像特征：

- 骨质破坏：X光和CT都显示出明确的溶骨性改变
- 软组织侵犯：MRI可见周围软组织肿胀
- 无明显Codman三角或日光放射状骨针（排除典型骨肉瘤）
- 病变部位在胫骨干中段，这是儿童常见病变位置

结合6岁女童的年龄特点：

- 儿童最常见的原发性恶性肿瘤是尤文肉瘤
- 但良性病变如骨囊肿、非骨化性纤维瘤也不能排除
- 急性骨髓炎虽然可能，但疼痛5天时间较短且无发热等感染症状

具体分析各可能性：

1. 尤文肉瘤：儿童期常见恶性肿瘤（占原发骨肿瘤4%），好发于长骨干骺端，表现为溶骨性破坏、软组织肿块。MRI显示T2WI高信号与临床符合
2. 骨囊肿：良性病变，但6岁患儿多为单纯性骨囊肿，常无症状或轻微疼痛。影像上应有更清晰的边界和液-液平面（本例未见）
3. 低度恶性肿瘤如软骨肉瘤？可能性较低，因为儿童罕见

需要排除的关键点：

- 骨髓炎：但X光显示骨质破坏而非骨膜反应为主
- 转移性肿瘤：6岁患儿无原发癌病史

下一步建议应聚焦于确诊和治疗方案制定。由于是恶性肿瘤可能性大，必须尽快明确诊断。

最后检查是否符合要求： □ 影像学描述客观（已列出关键特征） □ 3个诊断按概率排序 □ 给出具体临床建议 □ 符合骨肿瘤专家身份

确认无误后组织语言。

影像学描述：左胫骨干中段可见溶骨性骨质破坏区，边界模糊伴轻度骨膜反应；CT显示病灶呈低密度影，皮质不连续；MRI示病变区域T2WI信号混杂，周围软组织肿胀，未见明确液-液平面。

最可能诊断（按可能性排序）：

1. 尤文肉瘤（儿童胫骨干恶性肿瘤首选，好发6-15岁，典型表现为溶骨性破坏伴软组织侵犯）
2. 非骨化性纤维瘤（良性病变但可引起疼痛，需与恶性鉴别，影像上边界较清晰）
3. 急性骨髓炎（虽无发热等感染症状，但早期X线表现可类似，需结合实验室检查）

临床建议：立即行PET-CT评估全身转移情况；完善血常规、CRP及ESR检测；24小时内安排穿刺活检明确病理诊断；若确诊恶性肿瘤，应转入小儿肿瘤科启动多学科会诊（MDT）。

qwen3vl-32b-thinking · 313.739s (2.33 tok/s) · Mar 24, 9:59 PM
