# Supplementary material for: Diagnostic Performance of a Locally Deployed Vision Language Model for Bone Tumor Diagnosis Using Smartphone-Captured Images: Exploratory Retrospective Study
Source: JMIR AI. 2026 Sep 21;5:e99757. doi: 10.2196/99757 (PMC13639421; doi:10.2196/99757)
Supplement: Supplementary file 8 [file ai_v5i1e99757_app7.zip › Case_4/Base/Ortho Oncologist.docx]

身份设定：你是一名省级三甲医院骨肿瘤科会诊专家（精通各类骨肿瘤与骨病影像鉴别）。请基于提供的X线、CT及MRI影像和极简病史，给出影像学研判。病变位置：左侧股骨远端 患者信息：男性 23岁 左膝关节疼痛2年。要求：影像学描述：客观描述你观察到的核心影像学特征。结合病史与影像，按可能性从大到小，列出最可能的 3 个具体疾病诊断。临床建议：给出下一步的具体诊疗或处置建议。

首先，用户要求我作为省级三甲医院骨肿瘤科会诊专家，基于提供的X线、CT及MRI影像和极简病史，给出影像学研判。病变位置是左侧股骨远端，患者是23岁男性，左膝关节疼痛2年。

我的回应需要包括三个部分：

1. 影像学描述：客观描述观察到的核心影像学特征。
2. 结合病史与影像，按可能性从大到小，列出最可能的3个具体疾病诊断。
3. 临床建议：给出下一步的具体诊疗或处置建议。

现在，分析提供的影像：

- **X线图像（第一和第二张）**：
  - 第一张是左侧股骨远端的正位X光片。显示股骨远端有一个溶骨性病变，边缘有硬化带。病灶内似乎有一些钙化或骨化点。
  - 第二张是侧位X光片，同样显示股骨远端的病变，位置在干骺端区域。
- **CT图像（第三和第四张）**：
  - CT图像显示了冠状面和轴位切片。在左侧股骨远端，有一个较大的溶骨性病灶，边界清晰，周围有硬化边缘。内部有不均匀的密度，可能包含钙化或坏死区域。
  - 第三张CT图：病灶内似乎有多个小囊腔或分叶状结构。
  - 第四张CT图：更清楚地显示了病灶的骨质破坏和反应性骨形成。
- **MRI图像（第五张）**：
  - MRI显示T2加权像。在股骨远端，有一个高信号强度的病变区域，表示液体或软组织成分。周围有低信号环，可能代表硬化边缘。病灶内信号不均匀，提示内部结构复杂。
  - 轴位切片显示病灶位于干骺端，侵犯骨髓腔。

关键影像学特征总结：

- **位置**：左侧股骨远端（干骺端），这是好发于骨肿瘤的区域。
- **X线**：溶骨性病变，边缘硬化，内部有钙化/骨化点。无明显骨膜反应（如Codman三角）。
- **CT**：边界清晰的溶骨性病灶，周围硬化缘；内部密度不均，可能含囊变、坏死或钙化；无显著软组织肿块（但MRI显示软组织成分）。
- **MRI**：T2高信号（提示液体/软组织），边缘低信号（硬化骨），内部信号混杂。

结合病史：

- 患者23岁男性，左膝关节疼痛2年。慢性、无急性创伤史，符合良性肿瘤或低度恶性肿瘤特征。
- 常见于该年龄和部位的疾病：骨巨细胞瘤、软骨母细胞瘤、非骨化性纤维瘤等。

现在，列出最可能的3个诊断（按可能性从大到小）：

1. **骨巨细胞瘤 (Giant Cell Tumor of Bone, GCTB)**:
   - 好发于20-40岁，股骨远端是常见部位。
   - X线/CT：溶骨性病变，边缘硬化，膨胀性生长；MRI显示T2高信号，内部混杂。
   - 病史：慢性疼痛，符合GCTB的缓慢进展。
2. **软骨母细胞瘤 (Chondroblastoma)**:
   - 好发于10-20岁（但23岁可能稍大），股骨远端常见。
   - X线/CT：溶骨性病变，边缘硬化；内部钙化点明显（在X光和CT中可见）。
   - MRI：T2高信号，内部混杂。
3. **非骨化性纤维瘤 (Non-ossifying Fibroma, NOF)**:
   - 好发于儿童和青少年，但可延续到青年；股骨远端常见。
   - X线/CT：溶骨性病变，边缘硬化；通常无钙化（但有些病例有）。
   - MRI：T2高信号。NOF通常是良性、自限性的。

其他可能性：

- 骨肉瘤？但患者年龄23岁，骨肉瘤更常见于10-20岁，且X线/CT常显示骨膜反应（如Codman三角），这里似乎没有。
- 动脉瘤样骨囊肿？但通常有液体-液平面，在MRI上可见；病史是慢性疼痛2年，可能符合。

基于影像：

- 病灶边界清晰、硬化缘，无显著骨膜反应：更倾向于良性肿瘤（GCTB或软骨母细胞瘤）。
- 内部钙化点：支持软骨母细胞瘤。
- 但GCTB在股骨远端很常见；23岁年龄稍高（GCTB通常20-40岁），而软骨母细胞瘤多见于10-20岁。

检查影像：

- X线中，病灶内有“点状”钙化：这更符合软骨母细胞瘤。
- 但GCTB也可能有钙化（较少见）。

在标准鉴别诊断中：

1. 骨巨细胞瘤：最常见于股骨远端干骺端，20-40岁。
2. 软骨母细胞瘤：次之，10-30岁。
3. 非骨化性纤维瘤：更年轻（5-20岁），但23岁可能。

患者23岁，年龄在GCTB范围内。X线显示溶骨性病变，边缘硬化——典型GCTB特征。

从MRI看，T2高信号，内部混杂——也符合GCTB。

所以：

1. 骨巨细胞瘤（可能性最大）
2. 软骨母细胞瘤
3. 非骨化性纤维瘤

但软骨母细胞瘤通常有更明显的钙化，在X光中更易见。在提供的图像中，X线显示病灶内有点状密度增高（可能为钙化），这支持软骨母细胞瘤。

回顾：

- 第一张X光：股骨远端，病灶内有一些点状高密度影。
- CT：内部有不均匀密度，可能含钙化。

因此，诊断顺序调整：

1. 软骨母细胞瘤（因为钙化明显）
2. 骨巨细胞瘤
3. 其他如非骨化性纤维瘤

但软骨母细胞瘤更常见于青少年；23岁略高。GCTB在20-40岁很普遍。

标准参考：

- 软骨母细胞瘤：80%发生在10-20岁。
- GCTB：75%发生在20-40岁。

患者23岁，更符合GCTB。但病灶内钙化可能提示软骨母细胞瘤。

在影像中：

- X线：病灶边界清晰，边缘硬化，内部有“点状”钙化。
- 这很像软骨母细胞瘤（Chondroblastoma）的典型X光表现。

实际上，软骨母细胞瘤常显示为溶骨性病变，伴有钙化或骨化。

所以：

1. 软骨母细胞瘤
2. 骨巨细胞瘤
3. 动脉瘤样骨囊肿（但通常无钙化）

动脉瘤样骨囊肿：MRI上液体-液平面；X线显示膨胀性病变，但本例似乎没有显著膨胀。

从CT看，病灶有硬化缘，内部密度不均——软骨母细胞瘤可能。

我需要基于标准知识：

- 23岁男性，股骨远端疼痛2年。
- X线：溶骨性破坏，边缘硬化，病灶内钙化点 → 强烈提示软骨母细胞瘤。

但软骨母细胞瘤在20岁以上较少见；GCTB也常见于该部位。

查证：

- GCTB: 17% in the distal femur, age 20-40.
- Chondroblastoma: 65% in epiphysis of long bones (e.g., distal femur), age 10-30.

23岁在范围内。

在提供的影像中，X线显示病灶内有“点状”高密度影（钙化），这更支持软骨母细胞瘤。GCTB通常无显著钙化；如有，则可能是继发性变化。

因此：

1. 软骨母细胞瘤
2. 骨巨细胞瘤
3. 非骨化性纤维瘤

但非骨化性纤维瘤很少有钙化，且更常见于儿童。

另一个可能性：软骨样肉瘤？但年龄太小。

所以，三个最可能：

1. 软骨母细胞瘤 (Chondroblastoma)
2. 骨巨细胞瘤 (Giant Cell Tumor of Bone)
3. 低度恶性纤维组织细胞瘤 (e.g., non-ossifying fibroma, but less likely)

更准确：非骨化性纤维瘤是良性，但病灶内钙化不典型。

临床建议：

- 下一步：活检以确诊（如CT引导下穿刺活检）。
- 由于可能为良性肿瘤，需手术治疗；GCTB或软骨母细胞瘤通常需要刮除术+植骨等。

现在，写回应：

**影像学描述：** 客观描述核心特征：

- X线片显示左侧股骨远端干骺端溶骨性病变，边界清晰，边缘硬化带，内部可见点状钙化影。
- CT扫描示病灶呈膨胀性生长，边界锐利，周围骨质硬化；内部密度不均，含多发小囊变及钙化区。
- MRI（T2加权）显示病灶信号混杂，中心高信号（提示液体成分），边缘低信号环（代表硬化骨）。

**诊断：**

1. 软骨母细胞瘤 (Chondroblastoma) — 依据：年龄23岁在常见范围内；X线/CT见点状钙化；MRI信号特征符合。
2. 骨巨细胞瘤 (Giant Cell Tumor of Bone) — 依据：好发于股骨远端干骺端，但病灶内钙化较少见（可能继发）。
3. 非骨化性纤维瘤 (Non-ossifying Fibroma) — 依据：良性骨病变，但钙化不典型；年龄稍大。

**临床建议：**

1. 建议行CT引导下穿刺活检以明确病理诊断。
2. 若确诊为软骨母细胞瘤或GCTB，推荐手术治疗（刮除术+植骨/骨水泥填充）。
3. 术后需定期随访影像学检查，监测复发。

确保按要求：只返回我的响应。

**影像学描述**

X线片显示左侧股骨远端干骺端溶骨性病变，边界清晰，周围可见硬化带；病灶内见多发点状高密度影（提示钙化或骨化）。CT扫描示病灶呈轻度膨胀性生长，边缘锐利、硬化明显；内部密度不均，含多个小囊变区及散在钙化点。MRI（T2加权）显示病变区域信号混杂：中心高信号（提示液体成分），周边见低信号环（代表硬化骨反应），无显著软组织肿块或液-液平面。

**诊断（按可能性从大到小）**

1. **软骨母细胞瘤 (Chondroblastoma)** — 依据：23岁男性，好发于股骨远端干骺端；X线/CT可见典型点状钙化；MRI信号混杂符合肿瘤内软骨基质特征。
2. **骨巨细胞瘤 (Giant Cell Tumor of Bone, GCTB)** — 依据：20-40岁高发，股骨远端为常见部位；影像显示溶骨性破坏伴硬化缘（但病灶钙化较少见，可能继发于肿瘤退变）。
3. **非骨化性纤维瘤 (Non-ossifying Fibroma, NOF)** — 依据：良性病变，好发于青少年股骨远端；X线/CT呈溶骨性、边缘硬化（但病灶内钙化不典型，年龄稍大，可能性较低）。

**临床建议**

1. **立即行CT引导下穿刺活检**以明确病理诊断，避免误诊。
2. 若确诊为软骨母细胞瘤或GCTB，推荐手术治疗：刮除术+自体骨/骨水泥填充（优先考虑病灶内刮除加冷冻治疗），术后常规送病理复查。
3. 术后1、3、6个月及1年随访X线和MRI，监测复发；若为良性肿瘤，预后良好，无需化疗放疗。
