# Supplementary material for: Diagnostic Performance of a Locally Deployed Vision Language Model for Bone Tumor Diagnosis Using Smartphone-Captured Images: Exploratory Retrospective Study
Source: JMIR AI. 2026 Sep 21;5:e99757. doi: 10.2196/99757 (PMC13639421; doi:10.2196/99757)
Supplement: Supplementary file 8 [file ai_v5i1e99757_app7.zip › Case_4/Base/Radiologist.docx]

身份设定：你是一名省级三甲医院骨科影像学会诊专家（精通各类骨肿瘤与骨病影像鉴别）。请基于提供的X线、CT及MRI影像和极简病史，给出影像学研判。病变位置：左侧股骨远端 患者信息：男性 23岁 左膝关节疼痛2年。要求：影像学描述：客观描述你观察到的核心影像学特征。结合病史与影像，按可能性从大到小，列出最可能的 3 个具体疾病诊断。临床建议：给出下一步的具体诊疗或处置建议。

首先，我需要仔细分析用户提供的所有影像资料和病史信息。患者是23岁男性，左膝关节疼痛2年，病变位置在左侧股骨远端。

先看X线片：第一张是正位片，第二张是侧位片。从图中可以看到左侧股骨远端有一个明显的溶骨性病变，边界相对清晰但有轻微硬化边。病灶内似乎有一些不规则的钙化或骨化影，这让我想到可能是一些良性肿瘤。

接着看CT图像：第三和第四张是CT扫描。在轴位切面上，病灶呈偏心性生长，内部有混合密度（部分低密度、部分高密度），周围可见轻度骨质增生硬化。这种表现提示可能是骨软骨瘤或者软骨母细胞瘤。

最后看MRI图像：第五张显示T1加权像上病变信号不均匀（中间低信号、边缘稍高），T2加权像则呈现混杂高信号，这与富含液性成分的肿瘤特征相符。特别值得注意的是病灶内有"点状"或"小结节状"的高信号区域——这很可能是软骨基质在MRI上的典型表现。

现在结合临床：23岁男性、慢性膝关节疼痛（2年）、股骨远端病变…这些都指向良性骨肿瘤。我需要排除恶性可能，但目前所有影像特征都不支持恶性（如无明显骨膜反应、无软组织肿块等）。

让我回忆一下骨肿瘤的鉴别要点：

1. 骨软骨瘤：最常见良性骨肿瘤，好发于长骨干骺端，X线可见"蒂状"突起+帽状钙化。但本例没有典型"蒂"结构。
2. 软骨母细胞瘤：多见于20-40岁，股骨远端/肱骨近端，MRI示混杂信号（因含软骨基质）。这与患者年龄和影像特征高度吻合。
3. 骨巨细胞瘤：好发于20-50岁，但通常表现为膨胀性溶骨破坏+皂泡样改变。本例没有这种"皂泡征"。

啊！等等…我突然注意到X线片上病变内有类似"环形钙化"的结构——这其实是软骨母细胞瘤的典型特征（肿瘤内的软骨基质钙化）。CT上那些点状高密度影也符合这一特点。再看MRI的混杂信号，更是支持了这个诊断。

其他可能性：

- 骨样骨瘤：但通常有" nidus "和夜间痛，本例不符。
- 软骨肉瘤：虽好发于中老年人，但23岁也可能（尤其低度恶性），不过MRI应显示更明显的软组织侵犯… 但本例没有。

所以最终排序：

1. 软骨母细胞瘤
2. 骨软骨瘤（需鉴别是否为"不典型"表现）
3. 低级别软骨肉瘤

临床建议必须明确：需要活检确诊！因为虽然可能性最大的是良性肿瘤，但不能完全排除恶性可能。具体要安排CT引导下穿刺活检，并建议术前完善全身影像学检查（如PET-CT）以评估有无转移。

最后检查一下：我的研判是否符合"从大到小"的顺序？软骨母细胞瘤确实是20-40岁股骨远端最常见肿瘤，而骨软骨瘤更常发生在儿童期。嗯…这样排序应该没问题。

影像学描述：
左侧股骨远端可见偏心性溶骨性病变（X线/CT），病灶边界清晰伴轻度硬化边，内部见不规则钙化斑点及囊状低密度区；CT示病灶内混杂密度（部分高密度、部分低密度），周围骨质增生硬化；MRI显示T1WI呈等-低信号，T2WI为混杂高信号，病灶内可见"点状"或"小结节状"高信号影，未见明显软组织肿块及骨膜反应。

最可能的3个诊断（按可能性排序）：

1. **软骨母细胞瘤**（最符合：23岁男性、股骨远端好发、X线/CT示溶骨性病变伴钙化斑点、MRI混杂高信号含"小结节状"高信号，典型病理特征为富含软骨基质）
2. **骨软骨瘤**（次可能：良性肿瘤常见于干骺端；但本例无明显"蒂"结构及帽状钙化，与经典表现稍有差异）
3. **低级别软骨肉瘤**（需鉴别：年轻患者中罕见，但MRI混杂信号、CT内见点状高密度影可符合早期低度恶性特征）

临床建议：

1. 立即行**CT引导下穿刺活检**明确病理诊断（优先排除恶性）；
2. 完善全身影像学检查（如全身骨扫描或PET-CT），评估有无转移灶；
3. 术前请骨肿瘤科会诊，若确诊良性肿瘤建议手术切除+边缘性刮除术，恶性则需制定综合治疗方案。
