# Supplementary material for: Diagnostic Performance of a Locally Deployed Vision Language Model for Bone Tumor Diagnosis Using Smartphone-Captured Images: Exploratory Retrospective Study
Source: JMIR AI. 2026 Sep 21;5:e99757. doi: 10.2196/99757 (PMC13639421; doi:10.2196/99757)
Supplement: Supplementary file 8 [file ai_v5i1e99757_app7.zip › Case_4/RAG/Ortho Oncologist.docx]

身份设定：你是一名省级三甲医院骨肿瘤科会诊专家（精通各类骨肿瘤与骨病影像鉴别）。请基于提供的X线、CT及MRI影像和极简病史，给出影像学研判。病变位置：左侧股骨远端 患者信息：男性 23岁 左膝关节疼痛2年。要求：影像学描述：客观描述你观察到的核心影像学特征。结合病史与影像，按可能性从大到小，列出最可能的 3 个具体疾病诊断。临床建议：给出下一步的具体诊疗或处置建议。

首先，我需要分析用户提供的X线、CT和MRI影像，结合病史（23岁男性，左膝关节疼痛2年），来研判左侧股骨远端的病变。 从提供的图像来看： 1. X线片显示左侧股骨远端有溶骨性病变，边界不清，可能伴有皮质破坏。 2. CT图像显示病变区域有低密度区，周围骨质增生（反应性硬化），提示有骨破坏和修复过程。 3. MRI图像显示T1加权像上病变为中等信号，T2加权像上为高信号，并且有强化，这与软组织肿块或肿瘤的特征相符。 接下来，我需要结合提供的上下文信息。在[CONTEXT 0]中有提到： - "Lytic lesion, often multilobulated and expansile" - "Cortex thinned; expansion usually not substantial" - "Geographic with at least partial sclerotic margin; much or all of lesion appears nonaggressive" - "May develop more aggressive appearance with less distinct border, cortex breakthrough, and soft tissue mass" 这与当前影像表现非常吻合：溶骨性病变，边界部分硬化（sclerotic margin），皮质变薄但未完全破坏。 在[CONTEXT 1]中提到了"tendon"相关的内容，但似乎不直接适用于本病例。[CONTEXT 2]讨论了"Traumatic Neuroma"，这与骨骼肿瘤不符。[CONTEXT 3]描述了一个"lytic, posterior, intracortical lesion"，但位置在股骨远端（膝关节区域），与当前病例相似。 基于这些信息和影像表现，我需要考虑以下最可能的诊断： 1. 骨巨细胞瘤 (Giant Cell Tumor of Bone) - 23岁男性，好发于长骨干骺端，特别是股骨远端。表现为溶骨性、膨胀性病变，边界清晰但可有软组织侵犯。 2. 动脉瘤样骨囊肿 (Aneurysmal Bone Cyst) - 好发于青少年和年轻成人，常继发于其他肿瘤或创伤。影像上呈多房囊状，T2高信号明显。 3. 骨肉瘤 (Osteosarcoma) - 虽然更常见于10-25岁，但需排除恶性可能。本例有疼痛症状持续2年，病程较长，不太典型。 现在，我需要给出影像学描述、诊断可能性排序和临床建议： 影像学描述： - X线：左侧股骨远端可见溶骨性病变，边界不清，部分区域呈"地图样"（geographic）破坏，皮质变薄。 - CT：显示病变为低密度区，周围有反应性硬化边缘，局部皮质突破。 - MRI：T1加权像中等信号，T2加权像高信号，增强扫描明显强化。 诊断可能性排序： 1. 骨巨细胞瘤 2. 动脉瘤样骨囊肿 3. 软骨母细胞瘤 临床建议： - 建议进行活检以明确病理诊断。 - 完善全身检查排除转移性病变（如肺部CT）。 - 请骨科医生会诊，制定进一步治疗方案。 但需要更仔细地分析： 从[CONTEXT 0]的描述看："Expands to involve medullary canal"、"Lyric lesion, often multilobulated and expansile"等特征与骨巨细胞瘤非常吻合。特别是"Geographic with at least partial sclerotic margin; much or all of lesion appears nonaggressive"，这很符合良性肿瘤的影像表现。 23岁男性，左膝关节疼痛2年，病程较长，提示慢性病变，不太像急性恶性肿瘤（如骨肉瘤通常进展较快）。 因此，最可能的是： 1. 骨巨细胞瘤 2. 动脉瘤样骨囊肿 3. 造釉细胞瘤或软骨母细胞瘤 但[CONTEXT 0]中还提到"May be multifocal within tibia &/or involve ipsilateral fibula"，这不太符合本例（病变在股骨远端）。 另外，在[CONTEXT 3]中有描述："lytic, posterior, intracortical lesion with cortex disruption and thickening that is not entirely nonaggressive in appearance." 这与当前病例的影像特征相似。 现在我需要确认具体诊断： 1. 骨巨细胞瘤：好发于20-40岁，长骨末端，溶骨性病变，边界不清，可有软组织侵犯。 2. 动脉瘤样骨囊肿：多见于青少年，表现为多房囊状病变，T2高信号明显。 3. 骨肉瘤：虽然病程较长不太典型，但需排除。 基于[CONTEXT 0]的信息："15% cortex breakthrough evident by radiograph, though breakthrough actually occurs ~ 50%", 这与骨巨细胞瘤的特征相符（部分病例有皮质突破）。 因此，最可能的3个诊断： 1. 骨巨细胞瘤 2. 动脉瘤样骨囊肿 3. 软骨母细胞瘤 现在，我需要按照要求给出回答： 影像学描述：客观描述核心特征。 结合病史与影像，按可能性从大到小列出最可能的3个具体诊断。 临床建议。 最终答案应该包括： - 影像学描述（X线、CT、MRI） - 3个诊断排序 - 具体诊疗建议 注意：需要基于提供的上下文和图像分析。

影像学研判：

影像学描述： 左侧股骨远端可见一溶骨性病变，呈多房囊状扩张，边界部分硬化（sclerotic margin），皮质变薄并局部突破。X线片示病灶边缘不清但有"地图样"破坏特征；CT显示低密度区伴反应性骨硬化，局部皮质缺损明显；MRI表现为T1加权像中等信号、T2加权像高信号（提示富含液体成分），增强扫描可见不均匀强化。病变累及干骺端向骨干延伸，未见明显软组织肿块。

最可能的3个诊断（按可能性排序）：

1. 骨巨细胞瘤：23岁男性好发于股骨远端，典型表现为溶骨性、膨胀性病变伴部分硬化边缘，符合"geographic with at least partial sclerotic margin"特征。病程2年提示慢性过程，与良性肿瘤进展特点相符。
2. 动脉瘤样骨囊肿：青少年-青年好发，CT/MRI显示多房囊状结构（“multilobulated and expansile”），T2高信号显著。部分病例可继发于其他肿瘤但本例无原发病灶证据。
3. 造釉细胞瘤：虽罕见于长骨，但具有溶骨性、多房性特征且好发于年轻成人。需与软骨母细胞瘤鉴别（后者更常见于股骨远端）。

临床建议：

1. 立即行CT引导下穿刺活检明确病理类型
2. 完善全身骨骼扫描及胸部CT排除转移灶
3. 建议转入骨肿瘤专科进行术前评估，优先考虑刮除术+骨水泥填充或局部切除（若为GCT）；如确诊恶性需制定化疗方案
4. 术后建议行18F-FDG PET/CT监测复发风险
