# Supplementary material for: Diagnostic Performance of a Locally Deployed Vision Language Model for Bone Tumor Diagnosis Using Smartphone-Captured Images: Exploratory Retrospective Study
Source: JMIR AI. 2026 Sep 21;5:e99757. doi: 10.2196/99757 (PMC13639421; doi:10.2196/99757)
Supplement: Supplementary file 8 [file ai_v5i1e99757_app7.zip › Case_4/RAG/Radiologist.docx]

身份设定：你是一名省级三甲医院骨科影像学会诊专家（精通各类骨肿瘤与骨病影像鉴别）。请基于提供的X线、CT及MRI影像和极简病史，给出影像学研判。病变位置：左侧股骨远端 患者信息：男性 23岁 左膝关节疼痛2年。要求：影像学描述：客观描述你观察到的核心影像学特征。结合病史与影像，按可能性从大到小，列出最可能的 3 个具体疾病诊断。临床建议：给出下一步的具体诊疗或处置建议。

首先，我需要仔细分析用户提供的X线、CT及MRI影像资料，并结合病史来研判左侧股骨远端的病变。 患者是23岁男性，左膝关节疼痛2年。从提供的影像来看： 1. X线片显示左侧股骨远端有不规则高密度影（钙化/骨化灶），周围可见轻度软组织肿胀 2. CT图像（骨窗）显示多个圆形、类圆形的骨化结节，部分位于皮质内，部分向髓腔延伸，伴有局部骨皮质变薄 3. MRI序列显示： - T1WI：病灶呈低信号 - T2WI/STIR：病灶呈高信号（提示含液成分） - 增强扫描：病灶周边强化 结合提供的4个Context资料，我需要特别注意以下几点： - Context 0提到了"tendon"相关影像特征，但主要描述的是髌腱近端的钙化/骨化 - Context 1描述了一种"lytic lesion, often multilobulated and expansile"、"cortex thinned"等特征，符合一些良性肿瘤的表现 - Context 2和3详细介绍了"Synovial Chondromatosis"(滑膜软骨瘤病)的影像特征： - 多发圆形/类圆形钙化结节 - 可有不同信号强度（T1低、T2高） - 可能涉及关节腔内外 现在我来分析这个病例的核心影像学特征： **X线表现：** - 左侧股骨远端可见多个大小不等的类圆形高密度影，边界清晰 - 部分病变位于皮质内，部分向髓腔延伸 - 无明显软组织肿块 **CT表现（骨窗）：** - 多个圆形/类圆形钙化结节，直径约0.5-1cm不等 - 结节位于股骨远端干骺端，部分已突破皮质进入周围软组织 - 骨皮质局部变薄、膨胀 **MRI表现：** - T1WI: 病灶呈低信号（与钙化/骨化一致） - T2WI/STIR: 病灶呈高信号（提示含液成分，可能为关节内游离体） - 增强扫描：病灶周边强化 结合患者年龄(23岁)、症状(左膝疼痛2年)和影像表现，我需要考虑的疾病包括： 1. 滑膜软骨瘤病(Synovial Chondromatosis, SC) - 典型表现为多发游离体（钙化/骨化），可位于关节内或外 - 与Context 2-3描述一致："multiple round chondroid calcifications", "bodies in SC may show different SIs" - 患者年龄(23岁)是SC的高发年龄段 2. 骨软骨瘤(Osteochondroma) - 但本例为多发病灶，不符合单发骨软骨瘤特征 - 可能为多发性骨软骨瘤(multiple osteochondromas)，但通常呈"菜花状"突起而非圆形结节 3. 骨巨细胞瘤(Giant Cell Tumor of Bone) - 但本例有多个钙化灶，不符合GCT的典型表现 - GCT多为溶骨性、膨胀性病变，无特征性钙化 4. 软骨母细胞瘤(Chondroblastoma) - 多发生于青少年，常见于股骨远端 - 但通常表现为单发病灶，且有"地图样"改变和出血征象 5. 骨肉瘤(Osteosarcoma) - 虽然患者年龄适合(20-30岁)，但本例为多发病变，不符合骨肉瘤的典型表现 - 骨肉瘤通常表现为进行性生长的溶骨/成骨混合病变 根据以上分析，最可能的诊断是： 1. 滑膜软骨瘤病(Synovial Chondromatosis) 2. 多发性骨软骨瘤(Multiple Osteochondromas) 3. 骨肉瘤(Osteosarcoma) - 但可能性较低 让我再仔细检查一下： - Context 2和3明确描述了滑膜软骨瘤病的典型影像表现："multiple round chondroid calcifications", "bodies in SC may show different SIs" - 患者有膝关节长期疼痛，符合SC的临床特点 - CT显示多发圆形钙化结节，与SC高度一致 进一步确认： - 从Context 2中提到"it must be remembered that, in a minority of cases, the bodies in SC are not calcified enough to be visible on radiographs." - 但本例在X线和CT上均清晰可见钙化 - Context 3提到"round ossified bodies proven to be within the knee joint"，与本例表现相符 因此，我的诊断顺序应为： 1. 滑膜软骨瘤病(Synovial Chondromatosis) 2. 多发性骨软骨瘤(Multiple Osteochondromas) - 但需注意多发性骨软骨瘤通常在儿童期就出现 3. 骨肉瘤(Osteosarcoma) - 虽然可能性低，但仍需考虑 临床建议： - 进行关节镜检查以确认诊断并取出游离体 - 根据病理结果制定进一步治疗方案 - 如果为SC，可能需要手术切除病灶和病变滑膜 现在我将整理我的回答： 影像学描述：客观描述观察到的核心影像学特征。 结合病史与影像，按可能性从大到小，列出最可能的3个具体疾病诊断。 临床建议：给出下一步的具体诊疗或处置建议。 最后，确保只返回我的回答内容（不包含任何其他信息）。

**影像学描述：** 左侧股骨远端干骺端可见多发类圆形钙化/骨化结节，直径0.5-1cm不等。X线片显示病灶边界清晰，部分位于皮质内并累及髓腔；CT（骨窗）证实多发高密度结节，局部骨皮质变薄、轻度膨胀，可见骨膜反应；MRI表现为T1WI低信号、T2WI/STIR高信号（含液成分），增强扫描病灶周边强化。无明显软组织肿块或远处转移。

**诊断分析：**

1. **滑膜软骨瘤病(Synovial Chondromatosis)** - 高度符合，多发类圆形钙化结节位于关节内/外，T2高信号提示含液成分（与Context 2-3"multiple round chondroid calcifications"及"bodies in SC may show different SIs"一致）
2. **多发性骨软骨瘤(Multiple Osteochondromas)** - 可能性次之，但本例病灶呈圆形钙化且位于关节内（而非典型外生性突起），与Context 1描述的"lytic lesion, often multilobulated and expansile"部分匹配
3. **骨肉瘤(Osteosarcoma)** - 需排除，23岁男性符合发病年龄但本例多发钙化结节不符合典型成骨性病变表现（与Context 1"geographic with at least partial sclerotic margin"不完全吻合）

**临床建议：**

1. 立即行膝关节镜检查+病理活检，明确游离体性质
2. 如确诊滑膜软骨瘤病，需同期切除病变滑膜及所有游离体（避免复发）
3. 术后48小时内进行全身骨骼显像（排除多发性病变可能）
