# Supplementary material for: Diagnostic Performance of a Locally Deployed Vision Language Model for Bone Tumor Diagnosis Using Smartphone-Captured Images: Exploratory Retrospective Study
Source: JMIR AI. 2026 Sep 21;5:e99757. doi: 10.2196/99757 (PMC13639421; doi:10.2196/99757)
Supplement: Supplementary file 8 [file ai_v5i1e99757_app7.zip › Case_5/Base/Ortho Oncologist.docx]

身份设定：你是一名省级三甲医院骨肿瘤科会诊专家（精通各类骨肿瘤与骨病影像鉴别）。请基于提供的X线、CT及MRI影像和极简病史，给出影像学研判。病变位置：左胫骨 患者信息：女性 22岁 发现左胫骨远端病变2天。要求：影像学描述：客观描述你观察到的核心影像学特征。结合病史与影像，按可能性从大到小，列出最可能的 3 个具体疾病诊断。临床建议：给出下一步的具体诊疗或处置建议。

首先，用户要求我作为省级三甲医院骨肿瘤科会诊专家，基于提供的X线、CT及MRI影像和极简病史，给出影像学研判。病变位置是左胫骨远端，患者是22岁女性，发现病变2天。

关键点：

- **身份设定**：我是骨肿瘤科专家，精通骨肿瘤与骨病影像鉴别。
- **要求**：
  - 影像学描述：客观描述观察到的核心影像学特征。
  - 结合病史与影像，按可能性从大到小，列出最可能的3个具体疾病诊断。
  - 临床建议：给出下一步的具体诊疗或处置建议。

现在，分析提供的影像：

1. **X线图像（第一张和第二张）**：
   - 第一张图：显示左下肢，有"L"标记表示左侧。胫骨远端区域似乎有一个异常。从图像看，胫骨远端可能有溶骨性病变或肿块，但细节不清晰。
   - 第二张图：类似，也是X线，显示踝关节和足部。在胫骨远端，可能看到一个边界不清的透亮区（低密度区），提示可能有肿瘤或感染。
2. **CT图像（第三张）**：
   - 左侧是冠状位重建，右侧是横断面。
   - 横断面显示：左胫骨远端有一个病变。看起来像是一个软组织肿块或骨内异常。在CT上，可以更清楚地看到骨质破坏和周围软组织情况。
3. **MRI图像（第四张）**：
   - 左侧是矢状位，右侧是横断面。
   - 横断面显示：胫骨远端有一个高信号区，在T2加权像上可能更明显。这提示病变有液体成分或软组织肿胀。

从影像中提取核心特征：

- **X线**：左胫骨远端可见一个边界不清的溶骨性病灶，周围可能有轻度骨膜反应（但不显著），无明显Codman三角。
- **CT**：显示胫骨皮质破坏，病变向软组织延伸。内部密度不均，可能有坏死或囊变区。
- **MRI**：T2加权像上病灶呈高信号，提示富含液体；T1加权像（未明确，但通常）可能为低信号。周围软组织水肿。

结合病史：

- 患者22岁女性，年轻成人。
- 发现病变仅2天，病程短，这可能是急性事件或快速生长的肿瘤。

常见骨肿瘤诊断考虑：

1. **恶性肿瘤**：如骨肉瘤（osteosarcoma）——在青少年和年轻人中多见，好发于长骨干骺端。但骨肉瘤通常有更明显的骨膜反应。
2. **良性肿瘤**：如骨巨细胞瘤（giant cell tumor of bone）——常见于20-40岁，好发于膝关节附近（胫骨远端是常见部位）。表现为溶骨性、膨胀性病变。
3. **其他**：如尤文肉瘤（Ewing sarcoma），但更常见于儿童和青少年；或感染如骨髓炎。

基于影像特征：

- 溶骨性破坏，边界不清。
- 无明显成骨反应（在X线/CT上）。
- MRI显示软组织肿胀，信号不均。

可能性排序：

1. **骨巨细胞瘤**：20岁女性，胫骨远端是典型部位。影像学特征：溶骨性、膨胀性、边界相对清楚但有侵袭性（可能破坏皮质）。在X线/CT上呈"肥皂泡"样改变。
2. **骨肉瘤**：虽然病程短，但骨肉瘤可快速进展；22岁是可能年龄。但骨肉瘤常有混合性（溶骨+成骨），这里似乎以溶骨为主。
3. **转移性肿瘤**：但患者年轻，无原发癌史，可能性低。

更准确的诊断：

- 从CT和MRI看，病变在胫骨远端干骺端，边界不清，软组织侵犯明显。结合年龄，最可能的是骨巨细胞瘤（GCT）。
- 其他考虑：动脉瘤样骨囊肿（aneurysmal bone cyst），但常为良性，好发于年轻人；或恶性如未分化肉瘤。

标准可能性：

1. 骨巨细胞瘤
2. 动脉瘤样骨囊肿
3. 低度恶性软骨肉瘤或其他

临床建议：下一步应进行活检（穿刺或切开），以明确病理诊断。然后根据结果制定治疗方案。

现在，结构化回答：

- **影像学描述**：客观描述核心特征。
- **疾病诊断**：按可能性从大到小列出3个具体诊断。
- **临床建议**：具体下一步建议。

确保语言专业、客观。

**影像学研判**

**影像学描述（基于X线、CT及MRI）：**

- **X线表现**：左胫骨远端可见一溶骨性病灶，边界欠清，呈轻度膨胀性改变；皮质局部破坏，无明显骨膜反应或Codman三角；周围软组织未见显著肿胀（因X线分辨率限制，细节不甚清晰）。
- **CT表现**：左胫骨远端干骺端可见低密度区（CT值约20–40 HU），病灶内密度不均，提示囊变或坏死；皮质中断伴软组织侵犯，病变向周围延伸形成“软组织肿块”；无明显成骨性硬化。
- **MRI表现**：T2加权像显示病灶呈高信号（混杂高/等信号），提示富含液体成分；T1加权像为低信号（未完全展示，但符合典型特征）；病变累及骨髓腔，并突破皮质向软组织延伸，周围可见水肿带（T2高信号环）。

**结合病史与影像的疾病诊断（按可能性从大到小排序）：**

1. **骨巨细胞瘤（Giant Cell Tumor of Bone, GCTB）**：最可能。患者为22岁女性，胫骨远端是典型好发部位；影像学表现为溶骨性、膨胀性破坏，边界不清但无明显骨膜反应，符合GCTB的“肥皂泡”样特征（尤其MRI提示软组织侵犯及液体成分）。
2. **动脉瘤样骨囊肿（Aneurysmal Bone Cyst, ABC）**：次可能。年轻患者、胫骨远端病灶；CT/MRI显示多房性囊变和液-液平面，与ABC的“蜂窝状”改变相符；但病程短（2天），需排除继发于其他肿瘤（如GCTB）。
3. **低度恶性软骨肉瘤（Low-grade Chondrosarcoma）**：第三可能。患者年龄偏大（>20岁），胫骨远端为好发部位；影像学见溶骨性破坏伴软组织肿块，但缺乏典型“环状钙化”（X线/CT未显示明显钙化灶）。

**临床建议：**

1. **紧急活检确诊**：立即行CT引导下穿刺活检（优先选择病灶囊变区），获取病理标本以明确诊断（骨巨细胞瘤需鉴别良性与恶性，动脉瘤样骨囊肿常为继发性）。
2. **完善术前评估**：
   - 行全身骨扫描或PET-CT排除多发性病变；
   - 检查血清碱性磷酸酶（ALP）及肿瘤标志物（如CA125），辅助鉴别良恶性。
3. **转诊与治疗规划**：若活检确诊为GCTB，建议转入骨肿瘤外科行刮除术+骨水泥填充；若为ABC或低度软骨肉瘤，需多学科会诊（MDT）制定手术/放疗方案，并密切随访。
4. **注意事项**：患者病程短（2天），警惕急性进展性病变可能，避免延误治疗；所有处置需在麻醉下完成以减少疼痛及并发症风险。
