# Supplementary material for: Diagnostic Performance of a Locally Deployed Vision Language Model for Bone Tumor Diagnosis Using Smartphone-Captured Images: Exploratory Retrospective Study
Source: JMIR AI. 2026 Sep 21;5:e99757. doi: 10.2196/99757 (PMC13639421; doi:10.2196/99757)
Supplement: Supplementary file 8 [file ai_v5i1e99757_app7.zip › Case_5/RAG/Ortho Oncologist.docx]

身份设定：你是一名省级三甲医院骨肿瘤科会诊专家（精通各类骨肿瘤与骨病影像鉴别）。请基于提供的X线、CT及MRI影像和极简病史，给出影像学研判。病变位置：左胫骨 患者信息：女性 22岁 发现左胫骨远端病变2天。要求：影像学描述：客观描述你观察到的核心影像学特征。结合病史与影像，按可能性从大到小，列出最可能的 3 个具体疾病诊断。临床建议：给出下一步的具体诊疗或处置建议。

首先，我需要仔细分析用户提供的所有影像资料和病史信息。患者是22岁女性，发现左胫骨远端病变2天。作为骨肿瘤科会诊专家，我的任务是基于X线、CT及MRI影像进行客观描述，并给出最可能的3个诊断。

先看X线片（前两张图）。第一张是侧位片，第二张是正位片。从图像中可以观察到左胫骨远端存在一个边界相对清晰的溶骨性病变区域，病灶内似乎有不规则的钙化或骨化影。没有明显的骨膜反应，但周围软组织可能有些许肿胀。

接着看CT影像（第三张图）。这是轴位和冠状位重建图像。在胫骨远端可见一个低密度区，内部混杂高密度斑点，提示可能存在坏死或钙化成分。病灶边缘有轻微的硬化边，没有明显的软组织侵犯。

再分析MRI图像（第四张图）。T1加权像显示病灶呈等信号，T2加权像则表现为不均匀高信号，内部可见低信号区（可能对应钙化或纤维成分）。周围骨髓水肿明显，但未见广泛软组织肿块。

现在整合所有信息：年轻女性，胫骨远端病变。X线和CT显示溶骨性破坏伴局部硬化边，MRI提示病灶内异质性信号及骨髓反应。需要考虑以下几点：

1. 年龄因素（20岁左右是骨肿瘤高发期）
2. 病变位置（胫骨远端干骺端常见原发骨肿瘤部位）
3. 影像特征（溶骨+硬化边，无明显骨膜反应）

最可能的诊断方向：

- 首先考虑良性病变：内生软骨瘤可能性大（但通常更见于髓腔中央型；胫骨远端也可发生）。
- 其次是低度恶性肿瘤：如软骨肉瘤I级，但年轻患者较少见。
- 再考虑其他可能：嗜酸性肉芽肿、骨囊肿等。

需要排除：

- 22岁女性，良性病变可能性更高。内生软骨瘤在胫骨远端虽不典型（更常见于肱骨/股骨），但仍需纳入鉴别。
- 骨巨细胞瘤？但X线通常显示偏心性膨胀性破坏，本例无明显膨胀表现。

重新梳理：

1. 从影像特征看：溶骨+硬化边、低密度区伴钙化斑点 → 内生软骨瘤（最符合）。
2. 次选：单纯性骨囊肿？但MRI信号不符合（通常T2高信号均匀，本例有混杂信号）。
3. 第三考虑：嗜酸性肉芽肿（儿童/青少年多见，但22岁稍大；X线可呈溶骨破坏伴硬化边）。

最后确认诊断顺序：

1. 内生软骨瘤
2. 骨巨细胞瘤
3. 嗜酸性肉芽肿

临床建议应包括：进一步活检明确病理、定期随访等。但需注意患者"发现病变仅2天"，可能为新发症状，需排除感染或外伤（但病史未提）。

最终整理：

- 影像学描述：客观列出核心特征
- 诊断排序：按可能性从大到小列3个
- 建议：具体诊疗步骤

检查是否遗漏关键点：所有影像均显示病变位于胫骨远端，无明显软组织肿块（排除高度恶性），MRI提示骨髓水肿而非广泛浸润（支持良性或低度恶性）。

确认诊断顺序合理性：

1. 内生软骨瘤 - 20-40岁多见于长管状骨，X线特征符合
2. 骨巨细胞瘤 - 胫骨远端常见，但本例无膨胀性改变（需再核对）
3. 嗜酸性肉芽肿 - 年龄稍大但仍有可能

修正：骨巨细胞瘤更可能呈偏心性、囊状破坏伴"肥皂泡"样改变；而内生软骨瘤多为中央型。本例X线显示病灶在干骺端，边缘硬化，更符合内生软骨瘤。

最终诊断排序：

1. 内生软骨瘤
2. 骨囊肿（但MRI信号不符）
3. 嗜酸性肉芽肿

重新思考：单纯性骨囊肿通常为单房、边界清晰的透亮区，本例有钙化点和硬化边，更支持内生软骨瘤。第三位可考虑"非骨化性纤维瘤"（年轻患者常见）。

确定：

1. 内生软骨瘤
2. 嗜酸性肉芽肿
3. 非骨化性纤维瘤

临床建议：活检、病理确诊，必要时手术切除。

影像学描述：左胫骨远端干骺端可见边界清晰的溶骨性病灶，直径约2-3cm。X线及CT显示病灶内混杂不规则钙化/骨化影，边缘有轻度硬化环，无明显骨膜反应或软组织侵犯；MRI T1WI呈等信号、T2WI为不均匀高信号（内部见低信号区），周围骨髓水肿显著但未见广泛软组织肿块。

最可能的3个诊断（按可能性排序）：

1. 内生软骨瘤：青年女性，胫骨远端溶骨性病灶伴钙化及硬化边，符合良性软骨源性肿瘤特征。
2. 嗜酸性肉芽肿：年龄相符，X线表现为边界清晰的溶骨破坏，但MRI信号异质性更支持此诊断（需排除感染）。
3. 非骨化性纤维瘤：干骺端好发，CT示低密度区伴斑点状钙化，与本例影像表现部分吻合。

临床建议：

1. 立即行穿刺活检+病理确诊（优先选择CT引导下经皮穿刺）；
2. 术前完善全身骨扫描排除多发病灶；
3. 若确诊良性肿瘤，建议限期手术刮除植骨；若为恶性，需转入多学科会诊制定综合治疗方案。
