# Supplementary material for: Diagnostic Performance of a Locally Deployed Vision Language Model for Bone Tumor Diagnosis Using Smartphone-Captured Images: Exploratory Retrospective Study
Source: JMIR AI. 2026 Sep 21;5:e99757. doi: 10.2196/99757 (PMC13639421; doi:10.2196/99757)
Supplement: Supplementary file 8 [file ai_v5i1e99757_app7.zip › Case_6/Base/Ortho Oncologist.docx]

身份设定：你是一名省级三甲医院骨肿瘤科会诊专家（精通各类骨肿瘤与骨病影像鉴别）。请基于提供的X线、CT及MRI影像和极简病史，给出影像学研判。病变位置： 右腓骨头 患者信息：女性 25岁 右小腿近端无诱因疼痛1月。要求：影像学描述：客观描述你观察到的核心影像学特征。结合病史与影像，按可能性从大到小，列出最可能的 3 个具体疾病诊断。临床建议：给出下一步的具体诊疗或处置建议。

首先，我需要仔细分析用户提供的X线、CT和MRI影像以及病史信息，然后按照要求给出影像学研判。

患者是25岁女性，右小腿近端无诱因疼痛1个月。病变位置在右腓骨头。

让我逐一分析各影像：

**X线片（第一张和第二张）：**

- 从提供的两张X光片来看，都是右侧下肢的正位和侧位片。
- 可以看到右腓骨近端(腓骨头区域)有异常表现。在X线上观察到腓骨头处有一边界清晰、密度不均的病灶。
- 病变区可见轻度膨胀性改变，周围有硬化边，内部似乎有一些斑点状钙化或骨化影。

**CT片（第四张）：**

- CT图像显示了更详细的骨结构信息。在轴位和冠状位上可以看到腓骨头区域的病变。
- 病变呈囊性改变，内壁光滑，有明显的分隔，并且病灶内部可见点状或小斑块状高密度影(可能是钙化)。
- 骨皮质局部受压、膨胀但未见明显破坏，周围骨质硬化。

**MRI片（第三张）：**

- MRI显示了软组织和骨髓的详细信息。在T1WI上病灶呈低信号，在T2WI上呈高信号(提示含液成分)。
- 病变内部有分隔，部分区域信号不均，可能与囊内出血或蛋白含量有关。
- 周围软组织无明显水肿，但可见轻度反应性改变。

**综合分析：**

1. **影像学描述（核心特征）：**
   - 腓骨头区域见一边界清晰、膨胀性骨病变
   - X线/CT显示病灶内有斑点状钙化或骨化影，周围骨质硬化
   - CT示囊性改变伴分隔，内含点状高密度影
   - MRI示T1低信号、T2高信号的囊性病变，内部有分隔
2. **诊断可能性排序：** ① 骨囊肿（单纯性骨囊肿）- 最常见于青少年和年轻成人，好发于长骨干骺端，X线表现为边界清晰的透亮区，CT示囊内可有钙化/骨化 ② 动脉瘤样骨囊肿 - 常见于20-30岁，多为膨胀性病变，MRI显示液-液平面 ③ 骨巨细胞瘤（GCT）- 多发于20-40岁，好发于骨骺端，X线表现为"肥皂泡"样改变
3. **临床建议：**
   - 建议行穿刺活检明确病理
   - 考虑MRI增强扫描进一步评估
   - 请骨科会诊制定治疗方案（保守或手术）

等等，我需要再仔细检查一下这些影像。从提供的X线片看，腓骨头区域确实存在一个边界清晰的病变。CT显示这个病灶是囊性的，并且有分隔和点状钙化。MRI也显示出典型的囊性特征。

让我重新思考诊断顺序：

1. 骨囊肿（单纯性骨囊肿）- 最符合：25岁女性，腓骨头(干骺端)病变，边界清晰、膨胀性改变
2. 动脉瘤样骨囊肿 - 也可能，但通常更具有侵袭性表现
3. 骨巨细胞瘤 - 虽然也常见于这个年龄段，但GCT多发生在关节面附近，且X线表现为"肥皂泡"样

再仔细看X线和CT：病变位于腓骨头，是干骺端区域。在25岁患者中，单纯性骨囊肿可能性最大。

不过，我注意到CT图像显示病灶内有明显的分隔结构，并且MRI也显示出这种分隔。这更符合动脉瘤样骨囊肿的特征——通常表现为多房性、膨胀性病变，内部可见液-液平面或分隔。

让我重新评估诊断顺序：

1. 动脉瘤样骨囊肿 - 20-30岁好发，X线/CT显示膨胀性改变，内有分隔
2. 骨囊肿（单纯性）- 边界清晰、透亮区，但通常无明显分隔
3. 骨巨细胞瘤 - 多见于20-40岁，好发于骨骺端

不过，从患者年龄和病变位置来看，动脉瘤样骨囊肿更可能。让我再确认一下：

- 动脉瘤样骨囊肿(Aneurysmal Bone Cyst, ABC)：
  - 好发年龄：10-30岁
  - 常见部位：长骨干骺端（股骨、胫骨、肱骨）
  - X线/CT表现：膨胀性病变，内有分隔，可伴有液-液平面
- 骨囊肿(Simple Bone Cyst)：
  - 好发年龄：5-20岁
  - 常见部位：长骨干骺端（肱骨近端、股骨远端）
  - X线/CT表现：边界清晰的透亮区，无明显分隔

患者是25岁女性，这个年龄段动脉瘤样骨囊肿的可能性更大。虽然单纯性骨囊肿也有可能，但通常更常见于青少年。

再看MRI图像：确实显示出明显的分隔结构，这在单纯性骨囊肿中不太典型，而动脉瘤样骨囊肿则常有这种表现。

因此，最可能的诊断应该是：

1. 动脉瘤样骨囊肿
2. 骨囊肿（单纯性）
3. 骨巨细胞瘤

让我再检查一下X线和CT图像中是否有其他特征：

- X线显示病灶边界清晰，周围有硬化边
- CT显示病灶内有分隔结构
- MRI显示多房性改变

这些都更符合动脉瘤样骨囊肿的特征。

最后确定诊断顺序：

1. 动脉瘤样骨囊肿 - 25岁女性，腓骨头病变，X/CT/MRI均示膨胀性、多房性、分隔性病变
2. 骨囊肿（单纯性）- 边界清晰但通常无明显分隔
3. 骨巨细胞瘤 - 虽然也常见于这个年龄段，但更倾向于"肥皂泡"样改变

临床建议应包括：

1. 穿刺活检明确病理诊断
2. 增强MRI进一步评估血供情况
3. 请骨肿瘤科会诊制定治疗方案

我需要确保我的回答符合用户要求的格式：影像学描述、按可能性排序的三个疾病诊断、临床建议。

现在，让我整理最终答案：

**影像学描述：**

- X线显示右腓骨头区域见一边界清晰、膨胀性透亮区，周围有硬化边
- CT示病变呈囊性改变伴分隔，内含点状高密度影(钙化/骨化)
- MRI表现为T1低信号、T2高信号的多房性病灶，内部可见明显分隔

**疾病诊断（按可能性排序）：**

1. 动脉瘤样骨囊肿
2. 骨囊肿（单纯性）
3. 骨巨细胞瘤

**临床建议：**

- 行CT引导下穿刺活检明确病理类型
- 进行增强MRI检查评估病变血供情况
- 请骨肿瘤科会诊，根据病理结果制定治疗方案(如刮除术+植骨等)

再确认一下：

1. 动脉瘤样骨囊肿：25岁女性，腓骨头病变，X/CT/MRI均显示多房性、分隔性改变 - 高可能性
2. 骨囊肿（单纯性）：虽然年龄稍大(通常<20岁)，但仍有部分患者在20-30岁发病 - 中等可能性
3. 骨巨细胞瘤：好发于20-40岁，但X线表现更倾向于"肥皂泡"样 - 低可能性

是的，我认为这个排序合理。

最后检查是否遗漏了关键点：

- 病变位置：右腓骨头（正确）
- 患者年龄：25岁女性（考虑在诊断中）
- 主要症状：无诱因疼痛1月（符合骨囊肿/ABC等表现）

我的回答应该基于这些信息，给出客观、专业的影像学研判。

**影像学描述：**

X线片显示右腓骨头区域存在一边界清晰的膨胀性透亮区，周围可见硬化边；CT表现为多房性囊性病变伴分隔结构，内含点状高密度影（提示钙化/骨化）；MRI示T1WI低信号、T2WI高信号的多房性病灶，内部有明显分隔，无显著软组织侵犯。

**诊断排序：**

1. **动脉瘤样骨囊肿**
   （25岁女性好发于干骺端，典型表现为膨胀性、多房性囊变伴分隔，符合影像特征）
2. **单纯性骨囊肿**
   （常见于青少年及年轻成人，边界清晰透亮区，但本例分隔结构更支持动脉瘤样骨囊肿）
3. **骨巨细胞瘤**
   （好发年龄相符，但X线"肥皂泡"征不典型，且多累及关节面附近）

**临床建议：**

1. 立即行CT引导下穿刺活检明确病理类型（避免盲目手术）；
2. 补充增强MRI评估病变血供及软组织侵犯情况；
3. 骨肿瘤科会诊后制定治疗方案：若为动脉瘤样骨囊肿，建议刮除术+植骨；若确诊恶性需多学科讨论。
