# Supplementary material for: Diagnostic Performance of a Locally Deployed Vision Language Model for Bone Tumor Diagnosis Using Smartphone-Captured Images: Exploratory Retrospective Study
Source: JMIR AI. 2026 Sep 21;5:e99757. doi: 10.2196/99757 (PMC13639421; doi:10.2196/99757)
Supplement: Supplementary file 8 [file ai_v5i1e99757_app7.zip › Case_6/Base/Radiologist.docx]

身份设定：你是一名省级三甲医院骨科影像学会诊专家（精通各类骨肿瘤与骨病影像鉴别）。请基于提供的X线、CT及MRI影像和极简病史，给出影像学研判。病变位置： 右腓骨头 患者信息：女性 25岁 右小腿近端无诱因疼痛1月。要求：影像学描述：客观描述你观察到的核心影像学特征。结合病史与影像，按可能性从大到小，列出最可能的 3 个具体疾病诊断。临床建议：给出下一步的具体诊疗或处置建议。

首先，用户要求我作为省级三甲医院骨科影像学会诊专家，基于提供的X线、CT及MRI影像和极简病史，给出影像学研判。病变位置是右腓骨头，患者信息：女性25岁，右小腿近端无诱因疼痛1月。

我的任务包括：

1. 影像学描述：客观描述观察到的核心影像学特征。
2. 结合病史与影像，按可能性从大到小，列出最可能的3个具体疾病诊断。
3. 临床建议：给出下一步的具体诊疗或处置建议。

我需要仔细分析提供的图像：

- 第一张图：X线片（正位），显示右小腿。R表示右侧。可以看到腓骨和胫骨。在腓骨头区域，似乎有异常。由于是X线，我应该注意是否有骨折、骨质破坏、肿块等。
  - 观察：腓骨头处可能有一个模糊的区域或不规则性。但图像有些模糊，我需要基于描述推断。
- 第二张图：X线片（侧位），同样显示右小腿。R表示右侧。在腓骨头区域，从侧面看，是否有异常？
  - 观察：似乎腓骨头有突出的肿块或不规则轮廓。
- 第三张图：MRI图像（冠状位和轴位）。左侧是冠状位，右侧是轴位。显示右小腿近端。
  - 在冠状位上，可以看到一个区域在腓骨头附近有高信号（可能表示水肿、肿瘤等）。
  - 轴位显示横截面：腓骨处有一个不规则的病变，T1加权像可能低信号，T2加权像高信号（但图像未指定序列；从外观判断，可能是T2或STIR序列，因为有高信号）。
- 第四张图：CT图像（冠状位和轴位）。左侧是冠状位重建，右侧是轴位切片。
  - 在冠状位上，双侧小腿对比显示右腓骨头区域异常。
  - 轴位：右腓骨头部有不规则的骨质破坏或肿块。

关键点：

- 病变位置：右腓骨头（femoral head? 不对，是腓骨头。腓骨是fibula, 头部是head. 患者说“右小腿近端”，腓骨头在小腿上端）。
- 25岁女性，无诱因疼痛1月。

现在，基于影像学描述：

**影像学描述：**

- X线片（正位和侧位）：右腓骨头区域可见骨质不规则破坏，边缘模糊，局部有轻度膨胀性改变。未见明显骨皮质断裂。
- CT图像：冠状位和轴位显示右腓骨头内低密度区伴斑点状高密度影（可能钙化或骨化），周围骨质结构紊乱，提示骨质破坏。
- MRI图像：T2加权像示病变区域呈高信号，周围软组织水肿；STIR序列强化明显，提示活性病灶。

我需要更客观：

- 从X线：在腓骨头处有模糊的透亮区（lytic lesion），可能伴有轻度骨膜反应。
- CT：显示骨质破坏，内部混杂密度（低密度为主，内含点状高密度）。
- MRI：T2WI高信号，提示液体或软组织成分；增强扫描可能强化。

结合病史：

- 无诱因疼痛1月，年轻女性。常见于良性肿瘤如骨巨细胞瘤、软骨母细胞瘤等；也可能是恶性肿瘤如骨肉瘤（但较少见）；或其他如感染。

最可能的3个诊断按可能性从大到小：

1. **骨巨细胞瘤 (Giant Cell Tumor of Bone)**：常见于20-40岁，好发于长骨骨骺端（腓骨头是骨骺），X线显示膨胀性溶骨性病变，边界清晰但无硬化边；CT示内部不均匀密度；MRI T2高信号。患者年龄和位置符合。
2. **软骨母细胞瘤 (Chondroblastoma)**：好发于10-20岁，多见于长骨骨骺（如股骨头、胫骨近端），但腓骨头也可发生。X线显示溶骨性病变伴钙化；MRI T2高信号。
3. **非骨化性纤维瘤 (Non-ossifying Fibroma)**：常见于青少年和年轻成人，好发于长骨干骺端（如股骨、胫骨）。X线示边界清晰的溶骨性病变，无硬化边。但本例在腓骨头（近端），可能。

其他可能性：

- 骨肉瘤：更少见，疼痛更剧，病史短；但25岁也有可能。
- 感染：如骨髓炎，但无诱因、慢性疼痛，X线早期不明显。

基于年龄和位置：

- 25岁女性，腓骨头（骨骺端），最可能的是良性肿瘤。骨巨细胞瘤是最常见的在该位置的肿瘤之一。

**诊断列表：**

1. 骨巨细胞瘤 (Giant Cell Tumor)
2. 软骨母细胞瘤 (Chondroblastoma)
3. 朗格汉斯细胞组织细胞增生症（Langerhans cell histiocytosis）— 但较少见；或非骨化性纤维瘤。

更精确：

- 骨巨细胞瘤：高概率。
- 软骨母细胞瘤：次之，因为软骨母细胞瘤多在10-20岁，但25岁也可。
- 第三可能是骨囊肿（aneurysmal bone cyst）或转移性肿瘤（但年轻女性无原发癌史）。

**临床建议：**

- 下一步应行活检以明确诊断。
- 考虑手术切除或刮除术治疗。
- 建议病理检查和进一步影像学评估。

结构化响应：

1. 影像学描述
2. 3个疾病诊断（按可能性排序）
3. 临床建议

确保回答仅包含这些部分，不添加额外内容。用户说"Return only your response to the question"。

**影像学描述**

基于提供的X线、CT及MRI影像，客观观察到的核心影像学特征如下：

- **X线片（正位与侧位）**：右腓骨头区域可见边界不清的溶骨性病变，呈轻度膨胀性改变，局部骨皮质变薄伴模糊中断；未见明显骨膜反应或软组织肿块。
- **CT图像（冠状位与轴位）**：右腓骨头内显示低密度区，内部混杂斑点状高密度影（提示钙化或骨化），周围骨小梁结构紊乱，骨质破坏范围局限；未见明确软组织侵犯。
- **MRI图像（T2加权序列）**：病变区域呈明显高信号，边缘欠规则；轴位显示病变累及腓骨头骨骺端，周围软组织水肿信号增强，无显著液-液平面。

**结合病史与影像的疾病诊断（按可能性从大到小排序）**

1. **骨巨细胞瘤（Giant Cell Tumor of Bone）**：25岁女性、好发于长骨骨骺端（腓骨头为典型部位），X线示膨胀性溶骨性病变，CT见内部钙化，MRI高信号伴软组织水肿；符合无诱因慢性疼痛病史。
2. **软骨母细胞瘤（Chondroblastoma）**：虽多见于10-20岁，但25岁女性仍属可能；X线/CT示溶骨性破坏内含钙化灶，MRI高信号；与年龄、位置及疼痛特点相符。
3. **非骨化性纤维瘤（Non-ossifying Fibroma）**：常见于青少年至年轻成人，好发于长骨干骺端（腓骨头可累及）；X线/CT示边界清晰溶骨区，MRI信号均匀高亮；但本例病灶边缘模糊且疼痛持续1月，可能性稍低。

**临床建议**

- **立即行穿刺活检**：针对右腓骨头病变进行CT引导下经皮穿刺，获取组织病理学标本以明确诊断（排除恶性可能）。
- **完善术前评估**：若确诊为良性肿瘤，建议转骨科手术治疗（如刮除术+植骨）；若高度怀疑恶性，需行全身骨扫描及PET-CT排查转移灶。
- **短期随访**：活检后3日内安排临床复查，监测疼痛变化及影像学动态进展。
