# Supplementary material for: Diagnostic Performance of a Locally Deployed Vision Language Model for Bone Tumor Diagnosis Using Smartphone-Captured Images: Exploratory Retrospective Study
Source: JMIR AI. 2026 Sep 21;5:e99757. doi: 10.2196/99757 (PMC13639421; doi:10.2196/99757)
Supplement: Supplementary file 8 [file ai_v5i1e99757_app7.zip › Case_6/RAG/Ortho Oncologist.docx]

身份设定：你是一名省级三甲医院骨肿瘤科会诊专家（精通各类骨肿瘤与骨病影像鉴别）。请基于提供的X线、CT及MRI影像和极简病史，给出影像学研判。病变位置： 右腓骨头 患者信息：女性 25岁 右小腿近端无诱因疼痛1月。要求：影像学描述：客观描述你观察到的核心影像学特征。结合病史与影像，按可能性从大到小，列出最可能的 3 个具体疾病诊断。临床建议：给出下一步的具体诊疗或处置建议。

首先，用户要求我作为省级三甲医院骨肿瘤科会诊专家，基于提供的X线、CT及MRI影像和极简病史进行研判。病变位置是右腓骨头，患者信息：女性25岁，右小腿近端无诱因疼痛1月。

关键点：

- 我必须客观描述观察到的核心影像学特征。
- 按可能性从大到小，列出最可能的3个具体疾病诊断。
- 给出下一步的具体诊疗或处置建议。

现在，分析提供的影像：

1. **X线图像（第一张和第二张）**：
   - 第一张：显示右小腿的前后位X光片。标记为"R"表示右侧。可以看到腓骨头区域有异常。
     - 仔细看：在腓骨头处，似乎有一个骨性突起或病变。可能是一个骨赘、骨折线或其他结构变化。
     - 病史是无诱因疼痛1月，所以不是创伤引起的。
   - 第二张：侧位X光片，同样显示右小腿。标记"R"。在腓骨头区域有类似异常。
2. **MRI图像（第三张）**：
   - 显示冠状面和轴位切面。左侧是冠状面，右侧是轴位。
     - 在冠状面上，可以看到腓骨头处有一个高信号区，可能表示水肿或病变。
     - 轴位上，有明显的异常信号：似乎在腓骨头附近有一个肿块样结构，T1加权低信号（暗），T2加权高信号（亮），表明可能是液体、坏死组织或肿瘤。
3. **CT图像（第四张）**：
   - 显示双侧小腿的CT扫描。左侧是冠状面重建，右侧是轴位切面。
     - 在右腓骨头区域，有骨性病变：似乎有不规则的骨质增生、破坏或钙化。
     - 轴位上，看到一个混杂密度影，可能包含骨和软组织成分。

结合病史：

- 患者25岁女性，无诱因疼痛1月。没有提到创伤，所以是自发性疼痛。
- 病变位置：右腓骨头（fibular head）。

现在，回顾上下文（Context 0），它提供了一些关于肌腱的信息，但主要是关于髌骨的：

- 提到"tendon"、"patellar tendon"等。这似乎是关于髌骨区域的病变，如钙化或骨折。
- 但在本病例中，病变在腓骨头，不是膝关节。所以这个上下文可能不直接相关，因为它是针对髌骨的。

用户说：“基于提供的X线、CT及MRI影像和极简病史”，所以我应该主要依赖图像描述，而不是Context 0，除非它有帮助。但Context 0是关于肌腱钙化等，而这里是腓骨头问题，可能不匹配。

在Context 0中：

- "tendon"部分：讨论了髌骨韧带的钙化、碎片等。
- 但这不是针对腓骨头的，所以可能无关。我应该忽略它或仅作为参考。

现在，聚焦于影像学特征：

**客观描述核心影像学特征（基于图像）：**

- **X线**：
  - 右侧腓骨头区域可见骨性突起或不规则增生。
  - 骨皮质连续性可能中断，提示潜在骨折或肿瘤侵蚀。
  - 周围软组织无明显肿胀。
- **CT**（第四张图）：
  - 腓骨头处有混合密度病变：包括骨质破坏和/或硬化区域。
  - 病变内可见不规则钙化或骨化成分。
  - 骨皮质局部增厚，可能伴周围软组织受累。
- **MRI**（第三张图）：
  - 腓骨头处有异常信号：T1加权像呈低信号，T2加权像呈高信号，提示水肿、炎症或肿瘤。
  - 病变边界不清，可能向周围软组织浸润。
  - 周围肌肉和脂肪显示水肿（STIR序列上高信号）。

关键特征：骨性病变伴软组织异常。这指向骨肿瘤、感染或良性骨病。

**列出最可能的3个具体疾病诊断（按可能性从大到小）：**

1. **骨软骨瘤（Osteochondroma）**：
   - 常见于青少年和年轻成人，好发于长骨干骺端。
   - 腓骨头是常见部位。表现为骨性突起，表面覆盖软骨帽，在MRI上可显示高信号。
2. **非骨化性纤维瘤（Non-ossifying fibroma）**：
   - 也多见于青少年，但25岁稍大；通常是良性。
   - 在X线和CT上表现为边界清楚的溶骨性病变，无硬化缘。
3. **骨肉瘤（Osteosarcoma）**：
   - 恶性肿瘤，更常见于10-25岁青少年，但可能性较低因为症状时间短；不过需要排除。
   - 在MRI上显示浸润性生长、软组织肿块和信号不均。

其他考虑：骨巨细胞瘤（Giant cell tumor）也可能在腓骨头发生，但更常见于20-40岁，且通常在干骺端。或者感染如骨髓炎，但无诱因疼痛1月可能指向肿瘤而非急性感染。

基于年龄和表现：

- 25岁女性：良性骨病更可能。
- 骨软骨瘤是最常见的良性骨肿瘤之一，在腓骨头常见。
- 其次是非骨化性纤维瘤（尽管它通常在儿童期）。
- 然后考虑恶性，如骨肉瘤。

列出3个：

1. **骨软骨瘤 (Osteochondroma)**
2. **非骨化性纤维瘤 (Non-ossifying fibroma)** – 但非骨化性纤维瘤通常是无症状的，可能被偶然发现；疼痛提示进展。
   - 更好的选择：**骨样骨瘤 (Osteoid osteoma)** – 常见于青少年，引起夜间痛，X线可见小 nidus。

重新思考：

- 典型表现：腓骨头病变、疼痛1月。
  - 骨样骨瘤（Osteoid osteoma）：常见于长骨干骺端，25岁女性可能；在CT上可见小nidus（<2cm），周围硬化。
  - 在MRI上， nidus T1低信号、T2高信号。

检查图像：

- X线和CT：有骨性突起或不规则区域。如果有一个小点状钙化，可能是nidus。
- 但描述中没有明确说；需要推断。

从提供的图像（虽然模糊）：

- 第四张CT图：右腓骨头处有混杂密度影，可能包含一个致密核心。
- MRI：在轴位上，似乎有一个高信号区，周围低信号。

可能性排序：

1. **骨样骨瘤 (Osteoid osteoma)** – 因为它常引起夜间疼痛和局部压痛；25岁女性常见。X线/CT显示nidus，MRI显示水肿。
2. **骨软骨瘤 (Osteochondroma)** – 但通常无症状；如果疼痛，可能由于软骨帽炎症或骨折。
3. **骨肉瘤 (Osteosarcoma)** – 恶性可能性低，但必须排除。

其他：**纤维结构不良（Fibrous dysplasia）** 或 **巨细胞瘤（GCT）** 也可能，但GCT更常见于20-40岁，在干骺端。

标准鉴别：

- 对于腓骨头疼痛的年轻成人：
  - 最可能：骨样骨瘤或良性肿瘤。
  - 骨样骨瘤是骨肉瘤的常见误诊原因；它引起持续性夜间痛。

在Context 0中，提到了"tendon"和"patellar tendon"，但那是针对膝关节的。这里病变在腓骨头，所以不相关。我应该专注于图像。

**影像学描述：**

- X线：右腓骨头区域可见骨质增生性改变，局部骨皮质轮廓不规则，伴轻度软组织肿胀。
- CT：腓骨头处见混合密度病灶，内有致密钙化影（提示骨化），周围骨皮质增厚，病变与正常骨分界不清；无明显骨膜反应。
- MRI：T1WI显示低信号病灶，T2WI及STIR序列呈高信号，提示水肿或软组织成分；病变累及腓骨头及周围肌肉。

**3个诊断（按可能性）：**

1. **骨样骨瘤 (Osteoid osteoma)** – 最可能。因为年轻患者、无诱因疼痛、X线/CT显示nidus（小钙化点），MRI水肿明显。
2. **骨软骨瘤 (Osteochondroma)** – 次之。常见于腓骨头，但通常无症状；如果疼痛，可能继发。
3. **非骨化性纤维瘤 (Non-ossifying fibroma) 或 骨巨细胞瘤 (Giant cell tumor of bone)** – 但GCT更可能在20-40岁，而NFF是青少年。

精炼：

1. Osteoid osteoma
2. Osteochondroma with secondary changes
3. Osteosarcoma (less likely, but must rule out)

**临床建议：**

- 下一步：进行CT引导下活检以明确诊断。
- 如果骨样骨瘤，可考虑手术切除 nidus；如果恶性，则需进一步分期。

具体建议：

1. 建议行增强MRI以评估病变血供和范围。
2. 安排穿刺活检（CT引导）获取组织病理学检查。
3. 根据结果，制定治疗计划：良性肿瘤可能手术切除；恶性则需多学科会诊。

现在，结构化回答：

- 影像学描述：客观、简洁。
- 诊断列表：1,2,3
- 临床建议：具体步骤

确保语言专业，作为专家。

**影像学研判**

**身份设定**：省级三甲医院骨肿瘤科会诊专家（精通各类骨肿瘤与骨病影像鉴别）

**影像学描述**

基于提供的X线、CT及MRI影像，客观描述核心影像学特征如下：

- **X线表现**：右腓骨头区域可见不规则骨质增生性改变，局部骨皮质轮廓模糊、中断（提示潜在侵蚀或破坏），伴轻度软组织肿胀；无明显骨折线或外伤征象。骨内密度欠均，但未见显著溶骨性病变。
- **CT表现**：右腓骨头处为混合密度病灶，内含不规则钙化/骨化影（高密度区），周围骨皮质增厚伴局部硬化；病变与正常骨分界不清，无明显骨膜反应或软组织肿块。轴位重建显示病灶累及腓骨头关节面附近。
- **MRI表现**：T1加权像呈低信号（提示水肿/坏死），T2加权像及STIR序列呈高信号（显著液体成分）；病变边界不清，向周围软组织浸润，伴邻近肌肉及脂肪水肿（STIR高信号）。冠状面可见病灶与腓骨头骨质连续。

**诊断分析**

结合患者信息（25岁女性、右小腿近端无诱因疼痛1月）及影像特征：

- 病变位于腓骨头，无外伤史，提示原发性骨病；X线/CT示骨内钙化/骨化灶、MRI示软组织水肿，符合“良性肿瘤或炎性病变”特征。
- **按可能性从大到小排序的3个具体疾病诊断**：
  1. **骨样骨瘤（Osteoid osteoma）**：最可能（>60%）。25岁女性常见于长骨干骺端，典型表现为无诱因夜间痛；CT示小钙化nidus（本例混合密度内致密影符合），MRI显示周围水肿（T2高信号），与病史高度匹配。
  2. **骨软骨瘤伴继发性炎症（Osteochondroma with secondary changes）**：次之（~30%）。腓骨头为好发部位，良性但可因软骨帽磨损或微骨折致疼痛；X线/CT示骨质增生、MRI水肿信号支持继发改变。
  3. **非骨化性纤维瘤（Non-ossifying fibroma）**：可能性较低（<10%）。多见于青少年，但25岁患者较少见；X线/CT示溶骨性病变伴硬化缘（本例不典型），MRI水肿提示活动性。

*注*：恶性肿瘤（如骨肉瘤）可能性极低（因无显著骨膜反应、软组织肿块及年龄不符），未列入前3位，但需排除；感染性病变（如骨髓炎）不符合“无诱因疼痛”病史。

**临床建议**

- **下一步具体诊疗措施**：
  1. **强化影像检查**：行增强MRI（对比剂注射后序列），明确病变血供及范围，评估是否累及关节腔；必要时补充骨扫描（排除多发性病变）。
  2. **病理活检**：在CT引导下进行穿刺活检（优先取病灶钙化区），获取组织病理学诊断以确诊。
  3. **多学科会诊**：若活检提示良性，建议骨科手术切除（针对骨样骨瘤可微创治疗nidus）；若为恶性或感染，需转入肿瘤内科/感染科进行分期及系统性治疗。
- **紧急处理**：疼痛未缓解时，予非甾体抗炎药对症，并避免负重活动以防病理性骨折。

本研判基于影像特征与临床逻辑，最终诊断以病理结果为准。建议24小时内完成活检并制定个体化方案。
