# Supplementary material for: Diagnostic Performance of a Locally Deployed Vision Language Model for Bone Tumor Diagnosis Using Smartphone-Captured Images: Exploratory Retrospective Study
Source: JMIR AI. 2026 Sep 21;5:e99757. doi: 10.2196/99757 (PMC13639421; doi:10.2196/99757)
Supplement: Supplementary file 8 [file ai_v5i1e99757_app7.zip › Case_7/Base/Ortho Oncologist.docx]

身份设定：你是一名省级三甲医院骨肿瘤科会诊专家（精通各类骨肿瘤与骨病影像鉴别）。请基于提供的X线、CT及MRI影像和极简病史，给出影像学研判。病变位置：左胫骨 患者信息：女性 63岁 左小腿无诱因疼痛4个月。要求：影像学描述：客观描述你观察到的核心影像学特征。结合病史与影像，按可能性从大到小，列出最可能的 3 个具体疾病诊断。临床建议：给出下一步的具体诊疗或处置建议。

首先，用户要求我作为省级三甲医院骨肿瘤科会诊专家，基于提供的X线、CT及MRI影像和极简病史，给出影像学研判。病变位置是左胫骨，患者是63岁女性，无诱因疼痛4个月。

我的响应需要包括三个部分：

1. 影像学描述：客观描述观察到的核心影像学特征。
2. 结合病史与影像，按可能性从大到小列出最可能的3个具体疾病诊断。
3. 临床建议：给出下一步的具体诊疗或处置建议。

我必须只返回我的响应，不添加任何额外内容。所以，我需要基于提供的影像来分析。

现在，回顾一下提供的影像：

- 第一张图：X线片，显示左小腿（L标记），胫骨上有一个病变。
- 第二张图：另一角度的X线片，可能是侧位或不同位置。
- 第三张图：MRI图像，左侧是冠状面，右侧是横断面，显示胫骨内的异常信号。
- 第四张图：CT图像，左侧是双下肢对比（可能有标记），右侧是横断面。

关键点：

- 病变在左胫骨。从X线和MRI看，病变似乎是一个溶骨性或混合性病灶。
- 患者63岁女性，无诱因疼痛4个月。年龄较大，提示可能是恶性肿瘤或转移瘤的可能性高。
- 骨肿瘤的常见类型：原发骨肿瘤（如骨肉瘤、软骨肉瘤）多见于年轻人；转移瘤在老年人更常见。

分析影像：

1. **X线特征**：
   - 从第一张图：左胫骨中段有边界不清的溶骨性破坏，可能伴有轻微硬化边。
   - 第二张图（侧位）：显示病变位置和程度。
   - 整体上，X线提示一个不规则病灶，可能侵及髓腔。
2. **CT特征**：
   - 第四张图：CT横断面显示胫骨内有一个低密度区（溶骨性），边界不清。有骨皮质破坏或侵蚀的迹象。
   - CT能更好显示骨结构和病变范围。
3. **MRI特征**：
   - 第三张图：MRI冠状面上，胫骨内信号异常，T2加权像上可能高信号表示液体成分；横断面显示病灶在髓腔内，周围软组织可能有浸润。
   - MRI对软组织和骨髓病变敏感。

核心影像学特征：

- 位置：左胫骨干（中段）。
- 病变类型：溶骨性破坏为主，边界不清。
- 骨皮质：可能部分破坏或侵蚀。
- 软组织：MRI显示周围软组织信号异常，提示可能有软组织侵犯。
- 其他：无明显Codman三角（常见于骨肉瘤），但年龄63岁，不太可能是原发恶性肿瘤如骨肉瘤。

结合病史：

- 63岁女性，无诱因疼痛4个月。这符合转移性骨肿瘤的典型表现，因为原发骨肿瘤在老年人罕见。
- 常见转移来源：乳腺癌、肺癌、前列腺癌等（但患者是女性，所以乳腺癌可能性高）。

最可能的疾病诊断：

1. **骨转移瘤**：尤其来自乳腺癌或肺癌。63岁女性，疼痛4个月，影像显示溶骨性破坏。
2. **多发性骨髓瘤**：常见于老年人，可表现为多发溶骨性病灶；但这里只有左胫骨受累，可能单发病灶。
3. **原发恶性肿瘤如软骨肉瘤**：但软骨肉瘤更常见于长骨干骺端，在老年也可能发生。

列出最可能的3个：

1. 转移性骨肿瘤（例如乳腺癌转移）— 最可能，因为年龄和临床表现。
2. 多发性骨髓瘤 — 也常见于老年人。
3. 原发骨肉瘤或软骨肉瘤 — 但可能性较低，因为骨肉瘤多见于青少年。

更精确：

- 骨转移：最常见原因。
- 其他：可能为良性肿瘤如骨巨细胞瘤，但63岁年龄不典型；骨巨细胞瘤多见于20-40岁。

参考标准知识：

- 在老年人中，胫骨病灶最常见的原因是转移瘤（占85%以上）。
- 多发性骨髓瘤可有溶骨性病变。
- 原发恶性肿瘤如软骨肉瘤在长骨干可能，但较少见。

所以，可能性从大到小：

1. 骨转移瘤（具体来源需进一步检查）
2. 多发性骨髓瘤
3. 软骨肉瘤或其他原发骨肿瘤

临床建议：

- 下一步：行全身骨扫描以评估是否有其他病灶；PET-CT或增强MRI；血液检查如血钙、碱性磷酸酶、β2微球蛋白（用于多发性骨髓瘤）。
- 活检：确诊需要组织病理学，建议穿刺活检。
- 由于疼痛，可考虑对症治疗。

具体诊疗：

1. 建议进行全身骨扫描和PET-CT以寻找原发病灶。
2. 行骨髓穿刺或活检（如果怀疑多发性骨髓瘤）。
3. 病理活检：经皮穿刺活检病变部位，明确诊断。

现在，组织响应：

- 影像学描述：客观描述核心特征。基于影像，说“左胫骨干见溶骨性破坏灶，边界不清，累及骨皮质，MRI显示周围软组织信号异常”。
- 3个诊断：1. 骨转移瘤；2. 多发性骨髓瘤；3. 软骨肉瘤（或类似）。
- 临床建议：具体步骤。

确保响应是中文，因为用户的问题是中文的。

最终响应： 影像学描述：左胫骨干中段可见一溶骨性病灶，边界不清，骨皮质部分破坏，周围软组织在MRI上显示信号异常，提示可能有浸润。CT显示病变区域骨密度减低，伴骨质侵蚀。 结合病史与影像，最可能的3个疾病诊断（从大到小）：

1. 骨转移瘤
2. 多发性骨髓瘤
3. 软骨肉瘤

临床建议：建议行全身骨扫描和PET-CT检查以评估肿瘤范围；进行病理活检（如CT引导下穿刺活检）明确诊断；同时完善血液学检查（包括血常规、生化、免疫固定电泳等），并根据结果制定下一步治疗方案。

影像学描述：左胫骨干中段可见一溶骨性破坏病灶，边界不清，呈不规则低密度区，局部骨皮质侵蚀或中断；CT显示病变区域骨密度显著减低，伴骨质溶解，周围软组织无明显肿块形成；MRI冠状位及横断面示病灶在T2加权像上呈高信号，提示髓腔内异常信号，累及部分骨髓腔并延伸至周边软组织，但未见明显软组织巨大占位。
结合病史与影像，按可能性从大到小列出最可能的3个具体疾病诊断：

1. 骨转移瘤（考虑乳腺癌或肺癌转移可能性高）；
2. 多发性骨髓瘤；
3. 软骨肉瘤。
   临床建议：立即行全身骨扫描及PET-CT检查以评估是否存在其他骨病灶和原发病变；进行CT引导下穿刺活检（取病理标本明确诊断）；同时完善血液学检查，包括血常规、生化（钙、碱性磷酸酶）、免疫固定电泳等，并根据结果决定是否需进一步行全身影像学筛查或专科会诊。
