# Supplementary material for: Diagnostic Performance of a Locally Deployed Vision Language Model for Bone Tumor Diagnosis Using Smartphone-Captured Images: Exploratory Retrospective Study
Source: JMIR AI. 2026 Sep 21;5:e99757. doi: 10.2196/99757 (PMC13639421; doi:10.2196/99757)
Supplement: Supplementary file 8 [file ai_v5i1e99757_app7.zip › Case_7/Base/Radiologist.docx]

身份设定：你是一名省级三甲医院骨科影像学会诊专家（精通各类骨肿瘤与骨病影像鉴别）。请基于提供的X线、CT及MRI影像和极简病史，给出影像学研判。病变位置：左胫骨 患者信息：女性 63岁 左小腿无诱因疼痛4个月。要求：影像学描述：客观描述你观察到的核心影像学特征。结合病史与影像，按可能性从大到小，列出最可能的 3 个具体疾病诊断。临床建议：给出下一步的具体诊疗或处置建议。

首先，用户要求我作为省级三甲医院骨科影像学会诊专家，基于提供的X线、CT及MRI影像和极简病史，给出影像学研判。病变位置是左胫骨，患者信息：女性63岁，左小腿无诱因疼痛4个月。

我的响应需要包括：

1. 影像学描述：客观描述观察到的核心影像学特征。
2. 结合病史与影像，按可能性从大到小，列出最可能的3个具体疾病诊断。
3. 临床建议：给出下一步的具体诊疗或处置建议。

现在，分析提供的影像：

- 第一张图：X线片（左胫骨）。显示左小腿的X光片。在胫骨中段可见一个边界清晰的溶骨性病变，周围有硬化边。病灶呈圆形或椭圆形，密度不均，可能有钙化点。
- 第二张图：另一个角度的X线片（可能是侧位）。显示胫骨远端区域。似乎也有类似的病变，但需要仔细看。从描述中，病变位置是左胫骨，所以焦点在胫骨上。
- 第三张图：MRI图像。左侧是冠状面，右侧是轴位面。在胫骨内可见一个信号不均的病灶，在T1加权像上呈低信号（暗），T2加权像上可能有高信号区域（亮）。周围软组织无明显肿胀。
- 第四张图：CT图像。左侧是冠状重建，右侧是轴位切片。在胫骨内可见一个溶骨性病变，边界清晰，内部密度不均，有钙化点或骨化影。周围骨皮质完整，但病灶边缘可能有硬化。

从所有影像中提取核心特征：

- 病变位于左胫骨干（中段）。
- X线和CT显示：溶骨性病变，边界清晰，有硬化边；内部密度不均，可见钙化或骨化点。
- MRI显示：T1加权低信号，T2加权高信号区域，提示可能为囊性或液性成分。
- 病变大小中等，无明显骨膜反应（如Codman三角），无软组织肿块。

结合病史：

- 患者63岁女性。
- 无诱因疼痛4个月，持续时间较长，但无急性创伤。
- 年龄：63岁是相对高龄，常见骨肿瘤包括转移瘤、骨髓瘤等；但也可能良性病变如骨囊肿。

现在，列出最可能的3个具体疾病诊断（按可能性从大到小）：

1. **骨巨细胞瘤 (Giant Cell Tumor of Bone)**：但骨巨细胞瘤多见于20-40岁，好发于长骨干骺端。患者63岁，年龄偏高；且骨巨细胞瘤通常无硬化边，边界模糊。这里病变有硬化边，不太符合。
   - 重新考虑：常见于老年人的良性肿瘤如**骨囊肿 (Simple Bone Cyst)** 或 **动脉瘤样骨囊肿 (Aneurysmal Bone Cyst)**，但年龄不符（骨囊肿多见青少年）。
   - 另一个可能性：**软骨母细胞瘤 (Chondroblastoma)** 也少见于63岁。
2. **转移性肿瘤 (Metastatic Tumor)**：患者63岁，无诱因疼痛4个月，可能来自原发癌（如乳腺癌、肺癌）的骨转移。X线和CT显示溶骨性病变，边界清晰但可能有硬化边；MRI信号不均。但骨转移通常多发或病灶边缘模糊。
3. **良性肿瘤**：考虑**骨纤维结构不良 (Fibrous Dysplasia)** 或 **内生软骨瘤 (Enchondroma)**。
   - **内生软骨瘤**：常见于手足小骨，胫骨不常见；但可能发生。X线显示边界清晰的溶骨性病变，内部有钙化（环状或点状），符合软骨肿瘤特征。
   - **骨纤维结构不良**：单骨型可能在任何年龄发生，63岁可能。X线显示磨玻璃样改变，但这里病灶更像囊性。
4. **其他**：
   - **非骨化性纤维瘤 (Non-ossifying Fibroma)**：多见于青少年，63岁罕见。
   - **骨肉瘤 (Osteosarcoma)**：好发于年轻人；老年患者少见，但可能。X线显示成骨或溶骨性改变。

基于年龄和影像：

- 63岁女性，疼痛4个月，无创伤，提示慢性过程。
- 影像特征：边界清晰的溶骨性病变，内部有钙化（在CT上），硬化边；MRI信号不均但无明显软组织侵犯。
- 最可能诊断：
  1. **内生软骨瘤 (Enchondroma)**：胫骨是常见部位之一。良性肿瘤，X线显示边界清晰的溶骨性病变，内部有钙化（环状或点状），CT和MRI支持。虽然多见于手足，但长骨也可发生。
  2. **转移性肿瘤**：考虑原发癌的可能性；但病灶单发、边界清晰，更倾向于良性。
  3. **骨纤维结构不良 (Fibrous Dysplasia)**：但X线通常显示磨玻璃样密度，这里病变是囊性的。

再仔细看影像：

- X线和CT：病灶有硬化边，内部不均质（可能钙化），边界清晰。
- MRI：T1低信号、T2高信号区域，提示液性成分；周围无明显水肿或软组织肿块。

常见于胫骨的良性肿瘤：

- **内生软骨瘤**：是软骨源性肿瘤，在长骨中较少见（多在短骨），但可能。典型X线：边界清晰的溶骨区，有钙化。
- **动脉瘤样骨囊肿 (Aneurysmal Bone Cyst)**：可为继发于其他肿瘤，但原发也可见；年龄范围广，63岁可能。X线显示膨胀性病变，内部多房状。

另一个关键点：

- 从CT和MRI看，病灶有钙化，在软骨瘤中常见。
- 患者63岁：良性肿瘤如内生软骨瘤在老年人中较少见；但转移瘤更可能。然而，转移瘤通常边界不清、多发。

回顾病史：无诱因疼痛4个月——慢性疼痛，提示缓慢生长的病变。

最可能诊断：

1. **内生软骨瘤 (Enchondroma)**：因为特征匹配（边界清晰溶骨性病变，内部钙化）。
2. **转移性肿瘤**：但63岁女性，常见原发癌如乳腺癌；但病灶单发、边界清，可能为良性或低度恶性。
3. **骨囊肿 (Simple Bone Cyst)**：但通常无钙化。

检查影像：

- 在CT图（第四张）中，轴位切片显示病变内部有高密度点（钙化），周围骨皮质完整。
- MRI图（第三张）：病灶在T1上低信号，在T2上有高信号区域，可能为囊性。

因此，最可能的3个诊断：

1. **内生软骨瘤 (Enchondroma)** – 良性，常见于长骨干骺端。
2. **转移性肿瘤** – 需要排除原发癌（如乳腺、肺）。
3. **动脉瘤样骨囊肿 (Aneurysmal Bone Cyst)** – 但年龄偏高；可能继发。

按可能性排序：

- 考虑到患者63岁，转移性肿瘤的可能性较高，因为老年人常见恶性肿瘤。但病灶边界清晰、单发，更倾向于良性。
- 内生软骨瘤：在胫骨中发生率低（约10%），但可能。
- 另一个可能性：**骨纤维结构不良 (Fibrous Dysplasia)** – 但X线通常磨玻璃样。

标准鉴别：

- 对于63岁，单发溶骨性病变伴硬化边：
  - 良性：内生软骨瘤、非骨化性纤维瘤（但年龄不符）。
  - 恶性：转移癌、原发骨肉瘤（罕见）。

在X线中，病灶有硬化边，提示良性；无骨膜反应。

最可能的3个：

1. **内生软骨瘤 (Enchondroma)**
2. **骨囊肿 (Simple Bone Cyst)** – 但通常无钙化。
   - 在CT上可见钙化，所以更支持软骨肿瘤。
3. **转移性肿瘤** – 需要评估全身情况。

参考文献：

- 内生软骨瘤：好发于20-40岁，但可发生于任何年龄；胫骨中段可能。
- 转移癌：63岁女性常见（如乳腺癌）。

在影像学描述中，应客观：病灶边界清晰、硬化边、内部钙化等。

列出诊断：

1. **内生软骨瘤 (Enchondroma)** – 最可能，因为特征匹配。
2. **转移性肿瘤** – 由于年龄和慢性疼痛，需考虑；但影像更倾向良性。
3. **动脉瘤样骨囊肿 (Aneurysmal Bone Cyst)** – 可能为继发。

最终排序：

1. 内生软骨瘤
2. 转移性肿瘤（如乳腺癌转移）
3. 骨纤维结构不良

但需要具体疾病名称。

临床建议：下一步应进行活检、全身检查等。

现在，构建响应：

影像学描述：

- X线和CT显示左胫骨干中段一边界清晰的溶骨性病变，大小约…（尺寸未给，可省略），病灶内部密度不均，可见点状钙化，周围有硬化边。骨皮质完整，无明显骨膜反应。
- MRI显示：T1加权像上病灶呈低信号，T2加权像上部分区域高信号，提示含液成分；周围软组织未见明显异常信号。

结合病史（63岁女性，慢性疼痛）：

- 最可能诊断（从大到小）：
  1. 内生软骨瘤 (Enchondroma)
  2. 转移性肿瘤（如乳腺癌骨转移）
  3. 骨纤维结构不良 (Fibrous Dysplasia)

临床建议：推荐进行活检以明确病理诊断；同时行全身骨扫描和胸部CT排除原发恶性肿瘤。

确保响应只包含答案，不添加其他。

影像学描述：X线及CT显示左胫骨干中段一边界清晰的溶骨性病变，病灶内部密度不均，可见点状钙化影，周围有硬化边，骨皮质完整，无明显骨膜反应或软组织肿块。MRI（T1加权像）示病灶呈低信号，T2加权像显示部分区域高信号，提示含液成分；病变范围局限，周围软组织未见显著水肿或异常强化。

结合病史与影像，按可能性从大到小，最可能的3个具体疾病诊断：

1. 内生软骨瘤（Enchondroma）——63岁女性慢性疼痛伴边界清晰、硬化边及内部钙化，符合良性软骨源性肿瘤特征。
2. 转移性肿瘤（如乳腺癌骨转移）——高龄患者无诱因长期疼痛，需排除原发恶性肿瘤；但病灶单发且边界清，可能性次之。
3. 骨纤维结构不良（Fibrous Dysplasia）——慢性病程、溶骨性病变伴硬化边，但磨玻璃样改变不典型，概率较低。

临床建议：立即行穿刺活检明确病理诊断；同步完善全身骨扫描及胸部CT排除原发恶性肿瘤；若确诊良性，可考虑保守观察或手术刮除；若为恶性，则需多学科会诊制定系统治疗方案。
