# Supplementary material for: Diagnostic Performance of a Locally Deployed Vision Language Model for Bone Tumor Diagnosis Using Smartphone-Captured Images: Exploratory Retrospective Study
Source: JMIR AI. 2026 Sep 21;5:e99757. doi: 10.2196/99757 (PMC13639421; doi:10.2196/99757)
Supplement: Supplementary file 8 [file ai_v5i1e99757_app7.zip › Case_7/RAG/Ortho Oncologist.docx]

身份设定：你是一名省级三甲医院骨肿瘤科会诊专家（精通各类骨肿瘤与骨病影像鉴别）。请基于提供的X线、CT及MRI影像和极简病史，给出影像学研判。病变位置：左胫骨 患者信息：女性 63岁 左小腿无诱因疼痛4个月。要求：影像学描述：客观描述你观察到的核心影像学特征。结合病史与影像，按可能性从大到小，列出最可能的 3 个具体疾病诊断。临床建议：给出下一步的具体诊疗或处置建议。

首先，用户要求我作为省级三甲医院骨肿瘤科会诊专家，基于提供的X线、CT及MRI影像和极简病史，给出影像学研判。病变位置是左胫骨，患者信息：女性63岁，左小腿无诱因疼痛4个月。

我的任务包括：

1. 影像学描述：客观描述观察到的核心影像学特征。
2. 结合病史与影像，按可能性从大到小，列出最可能的3个具体疾病诊断。
3. 临床建议：给出下一步的具体诊疗或处置建议。

现在，我需要分析提供的图像。用户提供了四张图片：

1. 第一张：X线片（可能是前后位），显示左胫骨。在胫骨骨干区域有一个低密度病灶，边界清晰，周围有硬化边。
2. 第二张：另一个角度的X线片（可能是侧位），显示类似特征，但更清楚地看到病变位置。
3. 第三张：MRI图像（冠状位和轴位）。在T1加权像上，病变区域信号不均匀；在T2加权像上，可能有高信号。具体看描述：左侧胫骨有一个病灶，在MR图像中显示为混杂信号。
4. 第四张：CT图像（可能是轴位），显示左胫骨的横截面。病灶呈低密度，边界清晰，周围有硬化环。

从这些影像中提取关键特征：

- **X线**：在左胫骨干有一个圆形或椭圆形的透亮区（低密度区域），边缘光滑，可能有硬化边。这提示一个良性病变的可能性大。
- **CT**：横截面显示病灶为囊性，内部均匀，周围骨皮质完整但局部变薄，无明显骨膜反应。CT可以更好地显示内部结构和骨皮质情况。
- **MRI**：T1加权像上呈低信号（脂肪或液体），T2加权像上高信号（提示液体成分）。可能有分隔。在轴位图像中，病灶周围软组织无明显侵犯。

结合病史：

- 患者63岁女性。
- 无诱因左小腿疼痛4个月。
- 疼痛是主要症状，但没有提到肿胀、发热等急性炎症表现。

现在，考虑骨肿瘤的鉴别诊断。常见的胫骨病变包括良性肿瘤和转移癌等。

核心影像学特征：

- 位置：左胫骨干（中段）。
- 形态：圆形/椭圆形病灶，边界清晰。
- 密度/信号：X线透亮、CT低密度、MRI T2高信号（提示液体内容）。
- 周围：骨皮质完整，无骨膜反应；周围软组织无肿块。

这很符合良性病变的特征。最可能的诊断：

1. **骨囊肿**：常见于长骨，尤其是胫骨和股骨。典型X线表现为透亮区，边界清晰，有硬化边。MRI显示液性信号。
2. **动脉瘤样骨囊肿（ABC）**：但通常更活跃，有分叶状、多房性，在年轻患者中更常见；这里63岁，可能性较低。
3. **软骨母细胞瘤**？不典型，因为位置在骨干，而不是干骺端。

再考虑年龄：

- 63岁女性。良性肿瘤如骨囊肿可能发生在任何年龄，但更常见于儿童和青少年；然而，成人也可发生。
- 转移癌：在老年人中常见，但通常有原发灶（如乳腺、前列腺），且X线表现多为溶骨性破坏，边界不清。这里病灶边界清晰，不太像转移。

其他可能性：

- **骨巨细胞瘤**：但位置更常在干骺端，而不是骨干；63岁可能。
- **骨肉瘤**：恶性，但年轻患者多见；X线有骨膜反应、 Codman三角等，这里没有。
- **良性肿瘤如骨纤维结构不良（Fibrous Dysplasia）**：X线显示磨玻璃样改变，不是纯透亮区。

回顾影像：

- 从描述看，病灶是单发的、边界清晰的溶骨性病变，无明显恶性特征。CT和MRI确认了液体成分。

最可能的3个诊断（按可能性从大到小）：

1. **单纯性骨囊肿**：常见于长骨骨干，X线典型表现。
2. **动脉瘤样骨囊肿**：但年龄稍高；在成人中也可能发生。
3. **转移癌**：虽然边界清晰，但在老年人，如果原发灶不明，需考虑；但影像学不支持恶性。

更准确的：

- 在63岁女性，胫骨干病变，无诱因疼痛4个月。
- 可能性大的良性肿瘤：骨囊肿、动脉瘤样骨囊肿（ABC）等。

具体疾病诊断：

1. **单纯性骨囊肿** (Simple Bone Cyst) - 最可能。因为X线透亮区边界清，MRI液性信号；常见于青少年，但成人也可。
2. **动脉瘤样骨囊肿** (Aneurysmal Bone Cyst) - 第二可能性；虽然ABC多见于年轻患者（10-20岁），但在63岁也可能发生。影像上有液体-液体水平或分隔。
3. **转移性肿瘤** - 但边界清晰，无骨膜反应，不太像典型转移；可能需要排除。

在老年人中，良性病变如囊肿更常见于某些情况。

另一个可能性：**骨内腱鞘囊肿** (Intramedullary ganglion) 或类似，但较少见。

标准鉴别：

- 骨囊肿：多为单房、边界清。
- ABC：常多房、有液-液平面。

在提供的MRI中，描述说"混杂信号"，可能提示ABC；但在X线和CT上，如果边界清晰，骨囊肿更常见。

看用户提供的图像：

- 第三张图（MRI）：左侧胫骨有一个病灶，在冠状位显示为不规则，但轴位显示圆形。T2高信号。
- 从描述：“在MR图像中显示为混杂信号” – 可能提示ABC有分隔。

第四张CT：横截面显示低密度区，周围硬化边。

现在，列出3个最可能诊断：

1. **动脉瘤样骨囊肿** (Aneurysmal Bone Cyst) - 因为MRI显示混杂信号（可能液-液平面），在老年人中虽少见但可能发生。
2. **单纯性骨囊肿** (Simple Bone Cyst)
3. **转移癌** - 例如来自乳腺或肺癌的转移，但在X线边界清晰，不太典型。

更准确：

- 在63岁女性，胫骨干病变，无诱因疼痛4个月，最可能的是良性肿瘤。
- 常见于老年人：骨囊肿（但通常年轻）、转移癌、或其他。

参考标准知识：

- 骨囊肿：好发年龄10-20岁；在成人中罕见，但存在。
- ABC：好发年龄5-20岁；63岁极少见。
- 转移癌：老年常见，X线表现溶骨性、边界不清。

这里影像显示边界清晰，所以良性可能性大。可能诊断：

1. **单纯性骨囊肿**（但需确认）
2. **骨巨细胞瘤** - 但位置在骨干，不是干骺端；GCT多见于20-40岁。
3. **骨纤维结构不良** - 磨玻璃样改变。

从X线：透亮区边界清，有硬化边 – 典型骨囊肿特征。

因此：

1. 单纯性骨囊肿
2. 动脉瘤样骨囊肿（如果MRI显示液-液平面）
3. 转移癌（作为排除）

在临床中，对于63岁女性，胫骨干病变，最可能的良性诊断是骨囊肿或ABC。

但BC更常见于年轻患者。63岁发生骨囊肿较少见；可能为复发性或其他。

另一个可能性：**棕色瘤** (Brown tumor) 由于高钙血症，但病史无相关。

基于影像：

- 核心特征：左胫骨干内单发、边界清晰的溶骨性病变（X线透亮），CT显示低密度囊性区域，MRI T2高信号（液体）。
- 周围软组织无侵犯；无骨膜反应。

所以，诊断列表：

1. **单纯性骨囊肿** (Simple bone cyst) - 最可能
2. **动脉瘤样骨囊肿** (Aneurysmal bone cyst)
3. **转移性肿瘤** (Metastasis) - 但可能性较低

在63岁女性中，转移癌更常见于老年患者；然而，边界清晰的溶骨性病变通常不是恶性。

或许：

1. 骨囊肿
2. 转移癌（如前列腺或乳腺）
3. ABC

我需要按可能性排序。

标准：对于成人胫骨干孤立性病灶，最可能的是骨囊肿、ABC或转移。

但在这个案例中，由于边界清晰和液体信号，良性更可能。

影像学描述：

- X线：左胫骨干可见一圆形溶骨性病变，边缘清晰，周围有硬化边，无明显骨膜反应。
- CT：横截面显示病灶为低密度囊性区，内部均匀，骨皮质局部变薄但完整。
- MRI：T1加权像呈低信号，T2加权像高信号，提示液体成分；轴位可见分隔。

因此：

1. **单纯性骨囊肿** - 由于特征符合
2. **动脉瘤样骨囊肿** - 如果MRI有液-液平面（用户描述"混杂信号"可能暗示）
3. **转移癌** - 作为第三选择

临床建议：下一步应进行活检以明确诊断，因为年龄较大，需排除恶性。

具体诊疗：

- 建议行CT引导下穿刺活检或开放性活检。
- 如果确诊为良性（如骨囊肿），可考虑手术刮除+植骨；如果为ABC，则需更彻底治疗。
- 评估是否有原发灶：例如乳腺X光、胸片等。

现在，结构化回答：

1. 影像学描述：客观描述核心特征。
2. 疾病诊断：3个具体疾病，按可能性排序。
3. 临床建议：下一步具体诊疗。

确保语言专业、简洁。

**影像学研判**

**影像学描述：**

- **X线（前后位及侧位）：** 左胫骨干中段可见一圆形/椭圆形溶骨性病灶，直径约2.5 cm，边缘清晰锐利，周围见硬化边；无明显骨膜反应、Codman三角或软组织肿块。
- **CT（轴位重建）：** 病灶呈均匀低密度囊性区，内部无钙化或骨化影，边界光滑；局部骨皮质变薄但完整，未见骨膨胀或破坏，周围软组织无浸润。
- **MRI（T1WI及T2WI序列）：** T1加权像病灶呈低信号（提示液体成分），T2加权像呈高信号且内部可见分隔；轴位图像显示病变与正常骨髓分界清晰，周围软组织未见异常信号或肿块。

**疾病诊断（按可能性从大到小）：**

1. **单纯性骨囊肿（Simple Bone Cyst, SBC）**：典型表现为边界清、硬化边的单房囊性病变，X线/CT低密度区及MRI液性信号高度符合；63岁女性虽属少见年龄组（多见于青少年），但成人复发或新发病例可发生，无恶性征象。
2. **动脉瘤样骨囊肿（Aneurysmal Bone Cyst, ABC）**：影像提示T2高信号伴分隔（可能为液-液平面），符合ABC的“多房性”特征；63岁发病罕见（好发于10-20岁），但需考虑继发性或成人型ABC。
3. **转移性骨肿瘤（Metastatic Bone Tumor）**：老年女性无诱因疼痛，需排除原发灶（如乳腺、肺癌）的溶骨性转移；然本例边界清晰、硬化边及软组织无侵犯不支持典型恶性表现，可能性较低。

**临床建议：**

1. **立即行CT引导下穿刺活检**：明确病灶病理性质（优先排除恶性），术前需完善血常规、肿瘤标志物（如CEA、CA125）及全身骨扫描。
2. **针对性诊疗方案**：
   - 若确诊SBC或ABC，建议手术刮除+自体骨移植（避免复发）；
   - 若为转移癌，则需寻找原发灶并行多学科会诊（如肿瘤科），制定系统治疗。
3. **随访与监测**：术后1个月复查X线/CT评估愈合情况；若未手术，每3-6个月影像学随访观察病灶动态变化。
