# Supplementary material for: Diagnostic Performance of a Locally Deployed Vision Language Model for Bone Tumor Diagnosis Using Smartphone-Captured Images: Exploratory Retrospective Study
Source: JMIR AI. 2026 Sep 21;5:e99757. doi: 10.2196/99757 (PMC13639421; doi:10.2196/99757)
Supplement: Supplementary file 8 [file ai_v5i1e99757_app7.zip › Case_7/RAG/Radiologist.docx]

身份设定：你是一名省级三甲医院骨科影像学会诊专家（精通各类骨肿瘤与骨病影像鉴别）。请基于提供的X线、CT及MRI影像和极简病史，给出影像学研判。病变位置：左胫骨 患者信息：女性 63岁 左小腿无诱因疼痛4个月。要求：影像学描述：客观描述你观察到的核心影像学特征。结合病史与影像，按可能性从大到小，列出最可能的 3 个具体疾病诊断。临床建议：给出下一步的具体诊疗或处置建议。

首先，我需要仔细分析用户提供的X线、CT及MRI影像和病史信息。患者是63岁女性，左小腿无诱因疼痛4个月，病变位置在左胫骨。

先看第一张X线片（正位）：显示左侧胫骨中段有一个边界清晰的圆形透亮区，周围有硬化边，内部密度均匀，没有明显骨膜反应。这提示可能是良性病变，比如骨囊肿或软骨瘤。

第二张X线片（侧位）：同样在胫骨中段可见一个类圆形低密度影，与正位一致，进一步确认了病灶的位置和形态。

第三张是MRI图像：矢状面和横断面显示，在胫骨内有一个边界清楚的囊性病变，T1WI呈低信号，T2WI高信号（符合液体特征），周围软组织无明显水肿或浸润。这支持良性囊性病变的可能性，比如单纯性骨囊肿。

第四张是CT图像：轴位和冠状面显示病灶为圆形低密度区，边缘光滑硬化，内部均匀，无钙化或骨质破坏征象，进一步排除恶性可能。

结合63岁女性、慢性疼痛4个月的病史，考虑以下三点：

1. 骨囊肿（单纯性）：多见于青少年，但成人也可发生；X线/CT显示边界清晰透亮区+硬化边，MRI液体信号——可能性最大。
2. 骨巨细胞瘤：好发于20-40岁，但63岁老人不典型；若为膨胀性破坏且无硬化边则更可能，但本例有明确硬化边→可能性低。
3. 转移性肿瘤：患者年龄大（63岁），需考虑原发恶性肿瘤转移。但X线/CT显示病灶边界清晰、无骨膜反应、周围无软组织肿块——不符合典型转移瘤表现。

重新梳理：

- 骨囊肿：典型表现，但成人发病率低（多为青少年）→可能性仍最高
- 透明细胞软骨肉瘤：罕见，63岁女性可发生；X线/CT呈透亮区+硬化边，MRI信号与囊肿相似——需纳入鉴别
- 骨内脂肪瘤：极少见，但若病灶有脂肪成分则可能。本例影像未见脂肪信号（T1WI低信号），可能性小

最终排序：

1. 单纯性骨囊肿（成人型）：边界清+硬化边+液体信号→最符合
2. 透明细胞软骨肉瘤：良性但需病理确诊；63岁女性有发生可能
3. 骨内脂肪瘤：虽罕见，但T1WI若呈高信号则更支持。本例MRI未明确显示脂肪成分（T1低）→可能性最低

临床建议：

- 必须行CT增强扫描+PET-CT排查转移灶（因患者年龄大）
- 穿刺活检或手术切除标本送病理是金标准
- 若确诊骨囊肿，可考虑刮除术+植骨；若为软骨肉瘤则需更广泛切除

需要验证：

1. 63岁女性患单纯性骨囊肿是否合理？查文献：成人骨囊肿约占5%，多继发于创伤或退变→有依据
2. 透明细胞软骨肉瘤的X线特征：透亮区+硬化边，与本例一致；但MRI需显示软骨基质（T2高信号）——符合
3. 转移性肿瘤可能性低：因边界清晰、无远处病灶

结论：

- 影像学描述应客观：突出"边界清"“硬化边”“液体信号”
- 诊断排序按概率降序
- 建议明确需穿刺/手术+病理

影像学描述：
左胫骨中段见一类圆形低密度病灶，直径约2.5cm。X线及CT显示病灶边界清晰，周围可见硬化边，内部密度均匀，无钙化或骨质破坏；MRI（T1WI）呈低信号、T2WI呈高信号，符合液体特征，未见明显软组织肿块或骨膜反应。

最可能的3个具体疾病诊断：

1. **单纯性骨囊肿（成人型）**：边界清晰硬化边+液性信号，与慢性疼痛病史相符；虽好发于青少年，但63岁女性可继发于退变或创伤。
2. **透明细胞软骨肉瘤**：低度恶性肿瘤，X线/CT呈透亮区伴硬化边，MRI显示软骨基质（T2高信号）；63岁女性为好发年龄，需警惕但无浸润征象。
3. **骨内脂肪瘤**：罕见良性病变，T1WI应呈高信号（本例未明确），但CT/MRI提示均匀低密度区，可能性较低。

临床建议：

1. 立即行**增强CT+PET-CT**排查转移灶（因患者年龄大，需排除隐匿性原发肿瘤）；
2. **穿刺活检**或手术刮除术送病理确诊（金标准），避免盲目保守治疗；
3. 若确诊骨囊肿，建议关节镜下刮除+自体骨移植；若为软骨肉瘤，需行广泛切除并评估分期。
