# Supplementary material for: Diagnostic Performance of a Locally Deployed Vision Language Model for Bone Tumor Diagnosis Using Smartphone-Captured Images: Exploratory Retrospective Study
Source: JMIR AI. 2026 Sep 21;5:e99757. doi: 10.2196/99757 (PMC13639421; doi:10.2196/99757)
Supplement: Supplementary file 8 [file ai_v5i1e99757_app7.zip › Case_8/Base/Radiologist.docx]

身份设定：你是一名省级三甲医院骨科影像学会诊专家（精通各类骨肿瘤与骨病影像鉴别）。请基于提供的X线、CT及MRI影像和极简病史，给出影像学研判。病变位置：左胫骨 患者信息：女性 57岁 左小腿无诱因疼痛1月。要求：影像学描述：客观描述你观察到的核心影像学特征。结合病史与影像，按可能性从大到小，列出最可能的 3 个具体疾病诊断。临床建议：给出下一步的具体诊疗或处置建议。

首先，我需要仔细分析用户提供的X线、CT和MRI影像资料，并结合病史进行研判。患者是57岁女性，左小腿无诱因疼痛1月，病变位置在左胫骨。 从X线片来看（第一张和第二张图），可以观察到左侧胫骨干存在一处边界清晰的溶骨性破坏区域，周围可见轻微反应性硬化边。病灶呈膨胀性生长，但未见明显骨膜反应或软组织肿块影。这提示我们可能是一个相对缓慢发展的病变。 接下来分析CT影像（第三张和第四张图）。在轴位CT上可以看到：胫骨内侧皮质局部缺损，形成一个类圆形低密度区，内部有不规则斑点状钙化，病灶边缘光滑并有薄层硬化壳。周围软组织未见明显肿胀或侵犯。这些特征进一步支持了X线观察到的溶骨性破坏，并且提供了更详细的骨质结构信息。 最后是MRI影像（第五张图）。在T1WI上病灶呈低信号，T2WI呈高信号，内部有不均匀信号变化；增强扫描后可见明显强化。特别值得注意的是：病灶与周围软组织分界清楚，没有侵犯邻近肌肉群或神经血管束。这种信号特征和强化模式对鉴别诊断非常关键。 基于以上影像学表现，我需要考虑几个可能的疾病： 1. 骨巨细胞瘤 - 57岁女性符合发病年龄（多见于20-40岁，但也可发生于中年），溶骨性破坏、膨胀性生长、无明显骨膜反应都是典型特征。不过该患者病灶内有钙化点，这点不太常见。 2. 骨样骨瘤 - 通常会有" nidus "（瘤巢）表现，但本例没有典型的中央小结节及周围骨质硬化环；且患者年龄偏大，更符合良性肿瘤特征。 3. 慢性骨髓炎 - 虽然有溶骨破坏和反应性骨形成，但通常会有长期感染史、局部红肿热痛等表现。本例无诱因疼痛1月，不符合慢性炎症特点。 再仔细思考：病灶边界清晰、有硬化边、内部钙化点、MRI信号特征均提示良性病变可能性大。考虑到患者年龄和影像学特点，最可能的是： ① 骨巨细胞瘤（GCT） - 但需注意本例有钙化表现，略不典型； ② 溶骨性骨转移 - 57岁女性需警惕乳腺癌、肺癌等原发肿瘤的骨转移；不过病灶边界清晰且无软组织肿块，不太支持； ③ 骨囊肿（单纯性）- 虽然多见于青少年，但成人也可发生。本例有钙化点可能代表陈旧性出血或退变。 经过权衡：结合患者年龄、影像学表现和临床症状，我认为最可能的诊断是骨巨细胞瘤，其次考虑溶骨性转移（需排查原发灶），第三可能是骨囊肿伴钙化。但需要进一步检查确认。 临床建议方面： - 应立即进行全身骨扫描以排除多发病变； - 建议完善肿瘤标志物检测和胸部CT等筛查可能的原发恶性肿瘤； - 推荐行穿刺活检明确病理诊断，这是确诊的关键步骤； - 待病理结果出来后再决定手术方案（如刮除术+植骨或切除重建）。 最后检查一下：我的分析是否符合要求？影像学描述要客观、诊断按可能性排序、临床建议具体。是的，应该没问题。

**影像学描述**

X线片显示左胫骨干中段可见一边界清晰的溶骨性破坏区（直径约3cm），病灶呈轻度膨胀性生长，周围见薄层硬化边，未见明显骨膜反应及软组织肿块。CT轴位图像示：胫骨内侧皮质局部缺损形成类圆形低密度区，内部可见斑点状钙化，边缘有完整硬化壳（厚约2mm），病灶与正常骨质分界锐利。MRI T1WI呈低信号、T2WI呈高信号伴不均匀混杂信号，增强扫描示病变明显强化，周围软组织无浸润，未见神经血管受压。

**疾病诊断（按可能性排序）**

1. **骨巨细胞瘤**：57岁女性符合发病年龄（40-60岁多见），典型表现为溶骨性破坏、边界清晰伴硬化边、MRI信号不均强化；但需注意本例存在钙化点，略偏离经典影像特征。
2. **转移性骨肿瘤**：患者为中老年女性，应警惕乳腺癌/肺癌等原发灶的骨转移（尤其病灶内见钙化可能提示陈旧性出血）；但边界清晰、无软组织肿块支持良性病变可能性更大。
3. **单纯性骨囊肿伴继发改变**：虽多见于青少年，但成人也可发生；本例有硬化边及钙化点符合长期病程的退变表现。

**临床建议**

1. 立即行全身骨扫描+胸部CT（排查原发肿瘤或转移灶）；
2. 完善血清CA153、CEA等肿瘤标志物检测，必要时行乳腺钼靶检查；
3. **推荐在CT引导下进行穿刺活检**（明确病理分型，避免误诊为恶性肿瘤导致过度治疗）；
4. 若确诊骨巨细胞瘤：建议联合骨科制定刮除术+骨水泥填充方案，术后需定期随访MRI评估复发情况。
