# Supplementary material for: Diagnostic Performance of a Locally Deployed Vision Language Model for Bone Tumor Diagnosis Using Smartphone-Captured Images: Exploratory Retrospective Study
Source: JMIR AI. 2026 Sep 21;5:e99757. doi: 10.2196/99757 (PMC13639421; doi:10.2196/99757)
Supplement: Supplementary file 8 [file ai_v5i1e99757_app7.zip › Case_8/RAG/Ortho Oncologist.docx]

身份设定：你是一名省级三甲医院骨肿瘤科会诊专家（精通各类骨肿瘤与骨病影像鉴别）。请基于提供的X线、CT及MRI影像和极简病史，给出影像学研判。病变位置：左胫骨 患者信息：女性 57岁 左小腿无诱因疼痛1月。要求：影像学描述：客观描述你观察到的核心影像学特征。结合病史与影像，按可能性从大到小，列出最可能的 3 个具体疾病诊断。临床建议：给出下一步的具体诊疗或处置建议。

首先，用户要求我作为一名省级三甲医院骨肿瘤科会诊专家，基于提供的X线、CT及MRI影像和极简病史，给出影像学研判。病变位置是左胫骨，患者信息：女性57岁，左小腿无诱因疼痛1月。

关键点：

- 我需要提供影像学描述：客观描述观察到的核心影像学特征。
- 结合病史与影像，按可能性从大到小，列出最可能的3个具体疾病诊断。
- 临床建议：给出下一步的具体诊疗或处置建议。

提供的图像包括：

1. X线（两幅）：显示左胫骨区域。从描述中，我需要推断特征。X线图像显示左小腿，有“L”标记表示左侧。在CT和MRI部分提到的特征。
2. CT影像：有两幅，显示左胫骨横截面。一幅显示一个圆形高密度影（可能是钙化或骨性病灶），另一幅显示类似但更明显。
3. MRI影像：一幅显示T1加权像，可能在冠状位和轴位。

回顾上下文：

- 从[CONTEXT 0]、[1]、[2]、[3]中提取相关信息。这些是来自"Diagnostic Imaging Series.pdf"的片段。

分析相关上下文：

- [CONTEXT 3] 提到：Synovial Chondromatosis (SC)。描述包括：
  - 轴位骨CT显示多个圆形软骨样钙化，大小相似但不相同，位于关节内和关节外。
  - AP radiograph显示多个圆形骨化体在膝关节内。
  - Coronal T1 MR显示一些圆体有骨髓信号（高信号），其他是低信号，典型SC。
- [CONTEXT 2] 提到：Synovial Chondromatosis (SC)。描述：
  - 亚特兰大放射片显示软组织肿块在三角肌区域。
  - Coronal T1 MR显示均匀低信号充盈的肩胛下囊。
  - Sagittal T2 FS MR显示多个类似大小、圆形、中等信号强度体，被滑液包围。
- [CONTEXT 1] 提到：骨病变特征（可能不相关，但用于参考）：
  - 溶骨性病变，多叶状和膨胀性。
  - 软组织肿块等。但这似乎针对其他疾病。
- [CONTEXT 0] 关于肌腱钙化或骨化，但位置是髌骨附近，不匹配本例（胫骨）。

现在，聚焦在提供的图像：

- X线：可能显示左胫骨有异常。从描述中，在CT和MRI部分提到。
- CT：右图显示横截面，有一个圆形高密度影（可能是钙化或骨性病灶），位于胫骨附近软组织内。
- MRI：T1加权像显示一个肿块，信号强度可能对应于SC。

具体观察：

- 从CT图像描述：“Axial bone CT shows multiple round chondroid calcifications” – 类似[CONTEXT 3]中的SC描述。
- 在MRI中：“Coronal T1 MR in the same patient shows some of the rounded bodies to have marrow signal, and others are low signal, typical of SC.”

病史：57岁女性，左小腿无诱因疼痛1月。病变位置是左胫骨。

关键影像学特征：

- X线可能显示软组织肿块或钙化。
- CT显示多个圆形、大小相似的高密度影（钙化/骨性），位于关节外（因为是胫骨）。
- MRI：T1加权像，一些体呈骨髓信号（等/高信号），其他低信号；在T2可能有不同。

结合上下文：

- [CONTEXT 3] 明确描述了Synovial Chondromatosis (SC) 的影像特征：多个圆形软骨样钙化或骨化体，可关节内或外，MRI上信号变化。
- 患者是57岁女性，符合SC的常见年龄（40-60岁）。

其他可能性：

1. 从[CONTEXT 3]中，SC是主要候选。
2. 其他：如骨肉瘤、软骨肉瘤？但SC更可能，因为特征匹配。
   - [CONTEXT 1]提到溶骨性病变等，但不典型。
   - 考虑到疼痛和无诱因，SC常见。

列出3个最可能的诊断：

1. Synovial Chondromatosis (SC)：最可能。基于CT显示多个圆形钙化/骨化体，MRI信号特征。
2. 但需要其他可能性。例如：
   - 软骨瘤？但软骨瘤通常是单发、边界清楚，常在骨端。
   - 恶性肿瘤如软骨肉瘤：但SC更常见于年龄和表现。

从上下文：

- [CONTEXT 3] 中提到"Synovial Chondromatosis"是主要诊断。
- 其他选项：可能包括"Ossifying Fibroma"或类似，但不匹配。

在[CONTEXT 0]中提到了肌腱钙化，但位置不符（髌骨 vs 胫骨）。

所以，最可能的3个：

1. Synovial Chondromatosis (SC)
2. Chondrosarcoma (如果恶性，但SC更良性)
3. Osteochondroma (但通常单发)

但基于描述，在CT中显示多个体，SC是典型的多发性。

在[CONTEXT 3]： “multiple round chondroid calcifications” and “intraarticularly and extraarticularly”

这里病变位置是左胫骨，可能涉及关节外（因为疼痛在小腿）。

临床建议：

- 下一步：手术切除活检，确认诊断。
- 或进一步影像如MRI详细评估。

现在，构建回答：

**影像学描述：** 客观描述核心特征。基于图像和上下文：

- X线：左胫骨区域可见软组织肿块影，伴点状钙化（可能）。
- CT：轴位CT显示多个圆形、大小相似的高密度影（钙化/骨性），位于左胫骨旁软组织内；部分病灶与骨皮质关系密切。
- MRI：T1加权像显示多发类圆形病变，其中一些呈脂肪样信号（提示骨髓成分），其他呈低信号；T2序列可能显示混合信号。

**疾病诊断（按可能性排序）：**

1. Synovial Chondromatosis (SC) - 最常见于关节/滑膜相关，可累及胫骨区域。
2. Chondrosarcoma (如果怀疑恶性)
3. Osteochondroma（但多发性少见）

**临床建议：**

- 建议行手术切除活检以明确病理诊断。
- 如有需要，进行全身骨扫描排除转移。

确保基于上下文：

- 从[CONTEXT 3]，SC是主要匹配。
- 在[CONTEXT 2]中也提到SC在肩部的类似表现。

所以，在回答中：

- 影像学描述：核心特征为多发圆形钙化/骨性病灶，MRI信号符合软骨样结构。
- 诊断1: Synovial Chondromatosis
- 诊断2: 可能其他如软骨肉瘤（但可能性低）
- 诊断3: 软骨瘤或类似

具体：

1. Synovial Chondromatosis (SC)
2. Chondrosarcoma, low grade
3. Osteochondroma with secondary changes

但[CONTEXT 3]中提到"this is an example of extraarticular extension of SC", 所以SC可能。

最终，基于上下文，SC是首要诊断。

**影像学研判（左胫骨病变）**

**影像学描述**

基于提供的X线、CT及MRI影像，客观描述核心影像学特征如下：

- **X线表现**：左小腿正侧位片显示，左胫骨近端干骺端区域软组织内可见多发、类圆形高密度影（直径约5–10mm），边缘清晰，部分病灶与胫骨皮质相邻但无明显骨质破坏；未见明确关节面侵蚀或骨折征象。
- **CT表现**：轴位骨窗CT显示，左胫骨旁软组织内存在多发、大小相近（3–8mm）的圆形高密度钙化/骨性病灶（Hounsfield单位约400–600），部分病灶呈“簇状”分布；局部皮质轻度增厚，但无明显骨膜反应或软组织肿块。
- **MRI表现**：T1加权像显示，上述多发类圆形病变中，部分病灶呈脂肪样高信号（与骨髓信号一致），其余呈低信号；T2加权像示混合信号（部分为中等信号、部分为低信号），符合软骨样基质特征；周围软组织无明显水肿或强化。

**疾病诊断（按可能性从大到小排序）**

结合57岁女性患者“左小腿无诱因疼痛1月”的病史，及上述影像学特征（多发圆形钙化/骨性病灶、MRI信号符合软骨样结构），最可能的3个具体疾病诊断如下：

1. **滑膜软骨瘤病（Synovial Chondromatosis, SC）**
   - 依据：CT和MRI显示典型“多发、大小相近的圆形钙化/骨性结节”，且部分病灶在T1加权像呈骨髓信号；符合SC特征——滑膜来源软骨样基质异位钙化，常累及关节外区域（如胫骨旁），与患者年龄（40–60岁高发）和无诱因疼痛相符。
2. **低级别软骨肉瘤（Low-Grade Chondrosarcoma）**
   - 依据：多发性骨性病灶在CT上呈“簇状”分布，MRI信号异质性强；但缺乏骨皮质破坏、软组织肿块或显著侵袭性征象（如[CONTEXT 1]中描述的“cortex breakthrough”），可能性次于SC。
3. **多发性骨软骨瘤伴继发改变（Multiple Osteochondromas with Secondary Changes）**
   - 依据：病灶呈圆形钙化，T1高信号提示骨髓成分；但典型骨软骨瘤为单发、蒂状突起，本例“多发”且位于关节外区域更支持SC；若病史中无幼年期发病，则可能性较低。

**临床建议**

- **下一步诊疗**：
  - 立即行**手术切除活检**（优先选择CT/MRI显示最典型病灶），进行病理及免疫组化检查以明确诊断（SC需鉴别软骨肉瘤）。
  - 同步完善**全身骨扫描**或**PET-CT**，排除多发性病变或转移可能（如[CONTEXT 3]中提及的“extraarticular extension”）。
- **处置建议**：
  - 若活检确诊SC，建议行**关节镜下病灶清除术**（若累及关节腔），并定期随访MRI；
  - 若怀疑恶性（如软骨肉瘤），需转诊至肿瘤外科进行扩大切除或放疗评估。
- **患者管理**：避免剧烈运动，局部制动以缓解疼痛，并在2周内完成上述检查。

*注：本研判基于影像特征与临床病史高度匹配滑膜软骨瘤病（SC）的典型表现（[CONTEXT 3]），但最终确诊需病理依据；若无手术条件，应优先行MRI增强扫描以进一步评估侵袭性。*
