# Supplementary material for: Diagnostic Performance of a Locally Deployed Vision Language Model for Bone Tumor Diagnosis Using Smartphone-Captured Images: Exploratory Retrospective Study
Source: JMIR AI. 2026 Sep 21;5:e99757. doi: 10.2196/99757 (PMC13639421; doi:10.2196/99757)
Supplement: Supplementary file 8 [file ai_v5i1e99757_app7.zip › Case_9/Base/Radiologist.docx]

份设定：你是一名省级三甲医院骨科影像学会诊专家（精通各类骨肿瘤与骨病影像鉴别）。请基于提供的X线、CT及MRI影像和极简病史，给出影像学研判。病变位置：右胫骨 患者信息：男性 31岁 右小腿近端无诱因疼痛20天。要求：影像学描述：客观描述你观察到的核心影像学特征。结合病史与影像，按可能性从大到小，列出最可能的 3 个具体疾病诊断。临床建议：给出下一步的具体诊疗或处置建议。

首先，用户要求我作为省级三甲医院骨科影像学会诊专家，基于提供的X线、CT及MRI影像和极简病史，给出影像学研判。患者是31岁男性，右小腿近端无诱因疼痛20天，病变位置在右胫骨。

关键部分：

- 影像学描述：客观描述核心影像学特征。
- 结合病史与影像，按可能性从大到小列出最可能的3个具体疾病诊断。
- 临床建议：给出下一步的具体诊疗或处置建议。

我需要仔细分析提供的四张图像：

1. **第一张X线（正位）**：
   - 显示右小腿，R标记表示右侧。
   - 胫骨近端（上段）有一个明显的病变。看起来像一个边界清晰的溶骨性病灶，可能有轻度膨胀。骨皮质似乎有些破坏，但没有明显骨折。
2. **第二张X线（侧位）**：
   - 也是右小腿，R标记。
   - 胫骨近端有一个类似病变，在侧位上更清晰地显示了位置和形态。病灶在胫骨的前内侧或后外侧？从图像看，似乎是一个圆形的、边界相对清晰的区域。
3. **第三张CT图像**：
   - 左边是冠状面重建（可能），右边是轴位切片。
   - 轴位显示：胫骨近端有一个低密度病灶，内部有不规则结构，可能是囊性或坏死区。边界清晰，但似乎有轻微的骨皮质破坏。周围软组织没有明显肿胀。
4. **第四张MRI图像**：
   - 左边是矢状面（可能），右边是轴位。
   - 矢状面显示：胫骨近端有一个高信号病灶，在T1加权像上可能是低信号，但在T2或STIR序列上高信号。这表明液体成分或坏死组织。边界清晰，周围有轻微水肿。

从这些图像中提取核心影像学特征：

- **X线**：右胫骨近端（干骺端）可见一圆形、边界相对清晰的溶骨性病灶，直径约2-3cm。无明显Codman三角或日光放射状表现；骨皮质轻度膨胀，局部有薄层硬化边。
- **CT**：轴位显示病灶为低密度区（<30 HU），内部有斑点状高密度影（可能钙化或骨化）；骨皮质部分破坏，但无明显软组织肿块。
- **MRI**：T1WI呈低信号，T2WI/STIR呈高信号，提示病灶内含液体成分；边缘清晰，周围骨髓水肿不显著；无明显软组织侵犯。

结合病史：

- 31岁男性
- 右小腿近端无诱因疼痛20天（近期起病）
- 疼痛无诱因，可能与肿瘤或炎症相关

现在，列出最可能的3个疾病诊断，按可能性从大到小排序：

1. **骨巨细胞瘤 (Giant Cell Tumor of Bone, GCTB)**：
   - 常见于20-40岁，好发于长骨干骺端（如胫骨近端）。
   - 影像：溶骨性、膨胀性病灶，边界清晰，无明显骨膜反应；CT/MRI显示囊性改变，内部可有出血或坏死。
2. **动脉瘤样骨囊肿 (Aneurysmal Bone Cyst, ABC)**：
   - 多见于10-30岁，好发于长骨（如胫骨）。
   - 影像：膨胀性、多房性溶骨病灶；MRI显示液-液平面。
3. **软骨母细胞瘤 (Chondroblastoma)**：
   - 好发于10-20岁，但可延伸至30岁左右；常见于股骨头和胫骨近端。
   - 影像：边界清晰的溶骨性病灶，常有钙化；MRI信号混杂。

其他可能性（但按要求只列前3）：

- 骨肉瘤？不太可能，因为无Codman三角或日光放射状，且年龄稍大（骨肉瘤多见于10-20岁）。
- 转移性肿瘤？患者年轻，无原发癌史，可能性低。

排序理由：

- 31岁：GCTB最常见（25-45岁），ABC次之（10-30岁，但可稍晚），软骨母细胞瘤较少见于此年龄。
- 影像特征：病灶边界清晰、溶骨性、膨胀性，符合GCTB和ABC；MRI高信号提示液性成分。

所以：

1. 骨巨细胞瘤
2. 动脉瘤样骨囊肿
3. 软骨母细胞瘤

临床建议：

- 下一步：进行穿刺活检以明确病理诊断（如CT引导下穿刺）。
- 排除恶性肿瘤：如果高度怀疑GCTB，可能需要手术切除；但需先确诊。
- 建议：转诊至骨科，行MRI增强扫描评估血供，然后安排活检。

确保响应格式：

- 影像学描述
- 3个诊断（按可能性排序）
- 临床建议

最后，只返回我的响应。

影像学描述：
X线平片显示右胫骨近端干骺端见一圆形、边界相对清晰的溶骨性病灶，直径约2.5cm，局部骨皮质轻度膨胀伴薄层硬化边，无Codman三角或日光放射状骨膜反应；CT轴位重建示病灶呈低密度区（CT值<30 HU），内部可见斑点状高密度影（提示钙化或骨化），骨皮质部分破坏但未见明显软组织肿块；MRI矢状面及轴位序列示病灶T1WI呈均匀低信号，T2WI/STIR呈高信号（提示液性成分），边缘清晰，周围骨髓水肿不显著，无明显软组织侵犯。

结合病史与影像，按可能性从大到小列出最可能的3个具体疾病诊断：

1. 骨巨细胞瘤（Giant Cell Tumor of Bone, GCTB）—— 31岁男性好发于长骨干骺端，典型表现为边界清晰、膨胀性溶骨病灶伴液-液平面及轻微皮质破坏，符合本例影像特征。
2. 动脉瘤样骨囊肿（Aneurysmal Bone Cyst, ABC）—— 好发年龄10-35岁，以多房性溶骨膨胀为特点，MRI高信号区提示液性成分，与病灶形态及信号一致。
3. 软骨母细胞瘤（Chondroblastoma）—— 少见于30岁以上患者，但好发胫骨近端，常伴钙化或软骨基质，在CT上可见斑点状高密度影，需进一步鉴别。

临床建议：

1. 立即行CT引导下穿刺活检以明确病理诊断（优先排除恶性肿瘤）；
2. 完善全身骨扫描及胸部CT排查转移灶；
3. 转诊至骨科，根据活检结果决定治疗方案（如GCTB需手术刮除+植骨，ABC可考虑保守或手术干预）。
