# Supplementary material for: Diagnostic Performance of a Locally Deployed Vision Language Model for Bone Tumor Diagnosis Using Smartphone-Captured Images: Exploratory Retrospective Study
Source: JMIR AI. 2026 Sep 21;5:e99757. doi: 10.2196/99757 (PMC13639421; doi:10.2196/99757)
Supplement: Supplementary file 8 [file ai_v5i1e99757_app7.zip › Case_9/RAG/Ortho Oncologist.docx]

身份设定：你是一名省级三甲医院骨肿瘤科会诊专家（精通各类骨肿瘤与骨病影像鉴别）。请基于提供的X线、CT及MRI影像和极简病史，给出影像学研判。病变位置：右胫骨 患者信息：男性 31岁 右小腿近端无诱因疼痛20天。要求：影像学描述：客观描述你观察到的核心影像学特征。结合病史与影像，按可能性从大到小，列出最可能的 3 个具体疾病诊断。临床建议：给出下一步的具体诊疗或处置建议。

首先，我需要分析用户提供的X线、CT及MRI影像，并结合病史进行研判。患者是31岁男性，右小腿近端无诱因疼痛20天，病变位置在右胫骨。

从提供的影像来看：

- X线片显示：右胫骨近端（上段）有溶骨性病变，边缘可见部分硬化边，皮质变薄但未见明显突破。病灶呈多房性（multiloculated），大小约3-4cm。
- CT图像显示：病灶位于胫骨干骺端，内部有分隔结构，皮质轻度膨胀、变薄，无明确骨膜反应。CT值提示为软组织密度，但部分区域可见钙化/骨化点（可能对应MRI中的高信号）。
- MRI图像（T1WI和T2WI）：病灶在T1呈低信号，在T2呈混杂中高信号（提示含液体成分），内部有分叶状结构。周围软组织无明显侵犯，但可见轻度水肿。

结合提供的上下文信息：

- CONTEXT 0提到"lytic lesion, often multilobulated and expansile; cortex thinned… geographic with at least partial sclerotic margin"等特征，这与骨巨细胞瘤（GCT）的典型表现高度吻合。此外还指出"may develop more aggressive appearance"但本例未见皮质突破或软组织肿块。
- 该病灶位于胫骨干骺端（31岁男性），符合GCT好发于20-40岁、长骨骨骺区的特征。

其他可能诊断：

1. 骨巨细胞瘤：最符合，因多房性溶骨、边缘硬化、皮质变薄但无突破。
2. 动脉瘤样骨囊肿（ABC）：可表现为多房性膨胀性病变，但通常为液-液平面（MRI上更明显），本例未见此特征。且ABC好发于青少年，患者31岁稍大。
3. 骨肉瘤：可能性较低，因无恶性征象如骨膜反应、软组织肿块等；但需排除低级别成骨性肿瘤。

临床建议：

- 优先考虑GCT，需进行MRI增强扫描明确分隔及血供情况
- 推荐活检（经皮或开放）以确诊
- 若确认为GCT，可考虑刮除术+骨水泥填充

需要特别注意：CONTEXT 3中的"Synovial Chondromatosis"不符合本例特征（无关节内游离体），而CONTEXT 2的"Traumatic Neuroma"与骨病变无关。因此核心诊断应聚焦于GCT。

**影像学描述**

1. **X线表现**：右胫骨干骺端可见多房性溶骨性病灶，呈分叶状膨胀生长（大小约3-4cm），皮质轻度变薄但未见明显破坏；病灶边缘可见部分硬化边，无明确骨膜反应。
2. **CT表现**：病灶位于胫骨干骺端，内部呈多房性结构伴细小分隔，密度不均（含软组织及钙化成分）；皮质轻度膨胀、变薄，未见皮质突破或软组织肿块。
3. **MRI表现**：T1WI呈低信号，T2WI呈混杂中高信号（提示液性成分），病灶内可见分叶状结构；周围软组织无明显侵犯，但骨髓水肿信号（STIR序列）轻度增高。

**诊断可能性排序**

1. **骨巨细胞瘤（GCT）**
   - 核心依据：31岁男性、胫骨干骺端多房性溶骨性病变伴边缘硬化边、皮质变薄但无突破；符合"geographic with partial sclerotic margin"及"multilobulated and expansile"特征（参考CONTEXT 0）。
   - 排除点：未见明显软组织肿块或骨膜反应，不符合恶性转化表现。
2. **动脉瘤样骨囊肿（ABC）**
   - 核心依据：多房性膨胀性病灶、T2高信号液性成分；31岁患者虽非典型发病年龄（好发于青少年），但需考虑继发性ABC可能。
   - 排除点：未见典型的"液-液平面"，CT无显著脂肪密度或出血征象。
3. **低级别骨肉瘤**
   - 核心依据：溶骨性病灶伴轻度皮质膨胀、T2高信号；需警惕恶性可能（但非典型）。
   - 排除点：缺乏骨膜反应、软组织肿块及显著新生骨形成，不符合"aggressive appearance"。

**临床建议**

1. **立即完善增强MRI**：明确病灶分隔强化程度及血供情况（GCT通常呈中度不均匀强化），排除恶性转化征象。
2. **病理活检**：推荐经皮穿刺活检（避开血管丰富区），必要时开放活检以确诊，避免误诊为良性病变而延误治疗。
3. **多学科会诊**：若确诊GCT，建议骨肿瘤科联合放疗科制定方案（如刮除术+骨水泥填充）；若考虑低级别骨肉瘤，则需行广泛切除术。
4. **随访监测**：无论病理结果如何，建议2周内复查X线评估病灶动态变化（GCT进展较快）。

注：根据CONTEXT 0描述，GCT在胫骨病变中占35%-40%，且"15% cortex breakthrough evident by radiograph, though breakthrough actually occurs ~50%"——本例未见突破征象但需高度警惕潜在侵袭性。
