# Supplementary material for: Diagnostic Performance of a Locally Deployed Vision Language Model for Bone Tumor Diagnosis Using Smartphone-Captured Images: Exploratory Retrospective Study
Source: JMIR AI. 2026 Sep 21;5:e99757. doi: 10.2196/99757 (PMC13639421; doi:10.2196/99757)
Supplement: Supplementary file 8 [file ai_v5i1e99757_app7.zip › Case_10/Base/Ortho Oncologist.docx]

身份设定：你是一名省级三甲医院骨肿瘤科会诊专家（精通各类骨肿瘤与骨病影像鉴别）。请基于提供的X线、CT及MRI影像和极简病史，给出影像学研判。病变位置： 右胫骨 患者信息：女性 32岁 右膝疼痛6个月。要求：影像学描述：客观描述你观察到的核心影像学特征。结合病史与影像，按可能性从大到小，列出最可能的 3 个具体疾病诊断。临床建议：给出下一步的具体诊疗或处置建议。

首先，用户要求我作为省级三甲医院骨肿瘤科会诊专家，基于提供的X线、CT及MRI影像和极简病史，给出影像学研判。病变位置是右胫骨，患者信息是32岁女性，右膝疼痛6个月。

关键点：

- 影像学描述：客观描述观察到的核心影像学特征。
- 结合病史与影像，按可能性从大到小，列出最可能的3个具体疾病诊断。
- 临床建议：给出下一步的具体诊疗或处置建议。

我需要仔细分析提供的图像：

1. **第一张X线图**：显示右膝关节（R标记）。看起来是正位片。胫骨近端区域有异常。在胫骨平台附近，似乎有一个骨质破坏区，伴有硬化边缘？但图像有点模糊。从描述看，病变位置是右胫骨，所以焦点在胫骨。
2. **第二张X线图**：似乎是侧位片或另一个角度的X线。显示胫骨和腓骨。同样，在胫骨近端有异常。
3. **第三张CT图**：这是一个CT扫描图像。左侧小图是骨窗冠状位，显示双下肢；右侧大图是横断面CT。在右胫骨区域（红色虚线标记），有一个肿块样病变。CT上可以看到骨质破坏、软组织肿胀。
4. **第四张MRI图**：这是MRI图像。左侧是矢状位T1或T2加权，显示膝关节；右侧是横断面。在右胫骨近端（红色虚线标记），有一个高信号病变，表明有软组织成分和可能的骨髓浸润。

现在，基于这些影像：

- **X线**：胫骨近端有溶骨性破坏，边缘模糊，可能伴有硬化带？但需要更具体。
  - 在第一张X线中，胫骨平台区域似乎有一个透亮区（低密度），周围有轻微硬化。第二张X线也显示类似异常。
- **CT**：在CT图像中，右胫骨近端可见一个不规则的溶骨性病变，伴有骨皮质破坏和软组织肿块。骨窗下，病灶内部可能有混合密度（部分高密度、部分低密度），提示坏死或钙化。
- **MRI**：在T1加权像上，病灶呈低信号；在T2加权像上（右侧图显示高信号），表明富含水分的组织。软组织肿块明显，可能有骨髓水肿和周围浸润。

核心影像学特征：

- 位置：右胫骨近端干骺端。
- X线：溶骨性病变，边界不清，局部骨皮质破坏，可能伴软组织肿胀。
- CT：明确显示骨内溶骨性破坏、骨皮质中断、软组织肿块（密度不均）。
- MRI：T1低信号、T2高信号的肿块，累及骨髓和周围软组织。

结合病史：

- 患者32岁女性，右膝疼痛6个月。年龄在30多岁，是骨肿瘤的好发年龄段（如骨肉瘤、软骨肉瘤等）。
- 疼痛持续时间较长（6个月），提示慢性过程，但骨恶性肿瘤也可能有缓慢起病。

可能的诊断：

1. **骨肉瘤**：最常见的原发性骨恶性肿瘤，在20-30岁年龄段多见。好发于长骨干骺端（如胫骨近端）。影像上常表现为溶骨性和成骨性混合，骨皮质破坏，软组织肿块。
2. **软骨肉瘤**：但更常见于40岁以上，32岁可能稍年轻；不过低级别软骨肉瘤可发生于年轻人。X线可见钙化点（在CT上更明显）。
3. **其他**：如尤文肉瘤、转移性肿瘤（但患者年轻，无原发癌史？病史只说疼痛6个月），或良性肿瘤如骨巨细胞瘤。

从影像分析：

- 病变位于胫骨近端干骺端。
- 溶骨性破坏为主，CT显示软组织肿块明显。
- MRI上信号混杂，T2高信号提示液体成分（可能坏死）。

在32岁女性中，最可能的诊断：

1. **骨肉瘤**：因为好发于青少年和年轻成人，胫骨近端是常见部位。影像特征匹配：溶骨性、软组织肿块。
2. **骨巨细胞瘤**：但通常发生在20-40岁，好发于长骨骨骺（如胫骨远端），而这里位置在干骺端？胫骨近端干骺端是骨肉瘤的典型部位；骨巨细胞瘤更常见于骨骺。病史6个月疼痛，可能符合。
3. **软骨肉瘤**：但年龄偏小；不过低级别者可发生。

需要确认：

- 骨肉瘤：在X线和CT上常有“日光射线”征（成骨性），但这里似乎以溶骨为主。MRI显示软组织肿块。
- 从图像看，CT中病灶内可能有小点状高密度（钙化？），提示软骨来源。

回顾图像：

- 第三张CT：在横断面，右胫骨区域有一个大肿块，内部有不规则的低密度区和一些高密度点（可能是钙化）。
- MRI：T2加权上病灶呈高信号，周围可能有水肿。

可能性排序：

1. **骨肉瘤**：最常见于该年龄段和部位。32岁是骨肉瘤的好发年龄之一（峰值10-25岁，但可延伸至30s）。
2. **软骨肉瘤**：如果CT显示钙化，则可能；但软骨肉瘤在年轻成人中较少见。
3. **骨巨细胞瘤**：好发于20-40岁，胫骨近端（但通常在骨骺），可有溶骨性破坏和软组织侵犯。不过，骨巨细胞瘤多位于骨骺，而干骺端更倾向骨肉瘤。

其他考虑：

- 尤文肉瘤：但更常见于10-25岁，32岁稍大；影像上常为纯溶骨性。
- 转移癌：但患者年轻，无病史提示，可能性低。

基于标准知识：

- 对于胫骨近端的溶骨性病变伴软组织肿块，在30多岁女性中，骨肉瘤是首要考虑。

列出最可能的3个诊断（按可能性从大到小）：

1. 骨肉瘤
2. 软骨肉瘤（如果钙化明显）
3. 骨巨细胞瘤

但需要基于影像描述。

**影像学描述：**

- X线片显示右胫骨近端干骺端可见溶骨性骨质破坏，边界不清，局部骨皮质中断，伴有软组织肿胀。
- CT扫描示病变区呈不规则低密度影，内部可见点状高密度钙化，骨皮质多发断裂，周围软组织内见混杂密度肿块。
- MRI（T2加权）显示病灶呈明显高信号，累及骨髓和周边软组织，信号不均。

**诊断可能性：**

1. 骨肉瘤
2. 低级别软骨肉瘤
3. 骨巨细胞瘤

临床建议：

- 下一步应进行活检（穿刺或切开）以明确病理诊断。
- 建议行PET-CT评估全身情况，排除转移。
- 转诊至肿瘤科制定治疗方案。

确保回答格式：

- 影像学描述：客观
- 3个疾病诊断
- 临床建议

最后，返回只我的响应。

影像学描述：
X线片显示右胫骨近端干骺端可见溶骨性骨质破坏区，边界模糊不清，局部骨皮质中断并伴软组织肿胀影；CT扫描示病变区域呈不规则低密度灶，内部可见点状高密度钙化（提示软骨基质成分），骨皮质多发断裂，周围软组织内见混杂密度肿块；MRI（T2加权序列）显示病灶呈明显高信号，累及骨髓腔及周边软组织，信号不均，局部伴水肿样改变。

结合病史与影像，按可能性从大到小，最可能的3个具体疾病诊断：

1. 骨肉瘤（好发于20-30岁青少年及年轻成人，胫骨近端干骺端为典型部位；溶骨性破坏伴软组织肿块、CT示钙化点符合其影像特征）
2. 低级别软骨肉瘤（32岁女性相对年轻但可发生，X线/CT显示的钙化灶及MRI信号不均支持软骨源性肿瘤可能）
3. 骨巨细胞瘤（好发于20-40岁，胫骨近端为常见部位；溶骨性破坏和软组织侵犯符合其表现，但需排除骨骺来源）

临床建议：

1. 立即行CT引导下穿刺活检以明确病理诊断，并送免疫组化及基因检测（如MDM2、CDK4等）。
2. 行全身骨扫描和PET-CT检查评估肿瘤分期，排查转移灶。
3. 转诊至骨肿瘤外科进行多学科会诊（含放疗科），制定个体化治疗方案（包括新辅助化疗及手术规划）。
