# Supplementary material for: Diagnostic Performance of a Locally Deployed Vision Language Model for Bone Tumor Diagnosis Using Smartphone-Captured Images: Exploratory Retrospective Study
Source: JMIR AI. 2026 Sep 21;5:e99757. doi: 10.2196/99757 (PMC13639421; doi:10.2196/99757)
Supplement: Supplementary file 8 [file ai_v5i1e99757_app7.zip › Case_10/Base/Radiologist.docx]

身份设定：你是一名省级三甲医院骨科影像学会诊专家（精通各类骨肿瘤与骨病影像鉴别）。请基于提供的X线、CT及MRI影像和极简病史，给出影像学研判。病变位置： 右胫骨 患者信息：女性 32岁 右膝疼痛6个月。要求：影像学描述：客观描述你观察到的核心影像学特征。结合病史与影像，按可能性从大到小，列出最可能的 3 个具体疾病诊断。临床建议：给出下一步的具体诊疗或处置建议。

首先，用户要求我作为省级三甲医院骨科影像学会诊专家，基于提供的X线、CT及MRI影像和极简病史（女性32岁，右膝疼痛6个月），给出影像学研判。具体包括：

1. 影像学描述：客观描述观察到的核心影像学特征。
2. 结合病史与影像，按可能性从大到小，列出最可能的3个具体疾病诊断。
3. 临床建议：给出下一步的具体诊疗或处置建议。

我需要仔细分析提供的图像。用户提供了四张图片：

1. 第一张：X线片（正位），显示右胫骨区域。标记“R”表示右侧。在胫骨近端，似乎有一个不规则的病变。
2. 第二张：X线片（侧位），也显示右胫骨。同样有“R”标记，在胫骨近端可见一个异常区域。
3. 第三张：CT图像。左侧是冠状面重建图，右侧是横断面CT。在胫骨近端有一个低密度病变，边界清晰或不规则？需要仔细看。
4. 第四张：MRI图像。左侧是矢状位T2加权像（可能），右侧是轴位T1/T2加权像。显示胫骨近端的病变，有信号异常。

关键点：

- 病变位置：右胫骨（从描述中）。
- 患者信息：32岁女性，右膝疼痛6个月。
- 影像类型：X线、CT、MRI都提供了。

现在，分析影像学特征：

**X线片（第一张和第二张）：**

- 正位和侧位X线显示，在胫骨近端（靠近关节面处），有一个溶骨性病变。从图像看：
  - 在正位X线上，胫骨近端内侧似乎有局部骨质破坏、膨胀。
  - 侧位X线也显示类似区域。
- 可能特征：病灶边界可能较清晰或模糊？由于是X线，需要推断。常见骨肿瘤在X线上有溶骨性改变（如骨巨细胞瘤）。

**CT图像（第三张）：**

- 冠状面重建图显示胫骨近端有一个区域与对侧对比，密度异常。
- 横断面CT：病灶呈低密度区（可能溶骨性），边界不规则？或有硬化边？
  - 在CT上，病变似乎有囊性改变，周围有皮质破坏。

**MRI图像（第四张）：**

- 矢状位：在胫骨近端有一个高信号区域（T2加权像中亮，表示液体或软组织成分），边界清晰。
- 轴位：病灶呈混杂信号，中央可能有坏死区。周围软组织无明显肿块。

核心影像学特征：

- 从所有图像看，病变位于右胫骨近端（干骺端）。
- X线显示溶骨性破坏、局部膨胀。
- CT显示低密度囊性病灶，皮质变薄或破坏。
- MRI显示T2高信号（提示液体成分），可能有分叶状结构。

常见骨肿瘤鉴别：

- 32岁女性：年龄较年轻，考虑良性肿瘤更多。例如：
  - 骨巨细胞瘤（GCT）：好发于20-40岁，长骨干骺端，溶骨性，膨胀性。
  - 动脉瘤样骨囊肿（ABC）：可继发于其他病变，但也可原发；在年轻人中多见。
  - 骨软骨瘤或骨软骨肉瘤？但年龄和位置不太典型。

具体分析：

- 病变位置：胫骨近端。GCT常见部位是股骨远端、胫骨近端。
- 溶骨性，膨胀性：符合GCT特征。
- 无明显Codman三角（X线未显示）；GCT通常不侵犯关节面。

结合病史：

- 右膝疼痛6个月：慢性疼痛，可能与肿瘤相关。

最可能诊断排序：

1. 骨巨细胞瘤（Giant Cell Tumor of Bone, GCTB）
   - 好发年龄20-40岁，女性略多；胫骨近端是常见部位。
   - 影像：溶骨性、膨胀性破坏，边界清晰或有硬化边。
2. 动脉瘤样骨囊肿（Aneurysmal Bone Cyst, ABC）
   - 常见于青少年和年轻成人；可原发或继发于其他肿瘤。
   - 影像：液-液平面在MRI上常见，但这里CT/MRI显示囊性。
3. 软骨母细胞瘤（Chondroblastoma）？但软骨母细胞瘤更常见于10-20岁，胫骨近端较少；通常有钙化。
   - 从图像看，没有明显钙化，所以可能性低。

其他考虑：

- 骨肉瘤：但32岁年龄偏大（好发于青少年），X线可能显示Codman三角或日光放射状，这里似乎没有。
- 转移性肿瘤？但患者年轻，无原发病史提及；病史是“极简”，所以可能性低。

基于图像：

- MRI上：T2高信号，提示液体成分。GCTB在MRI常表现为混杂信号（因出血、坏死），ABC有液-液平面。
  - 在第四张MRI中，轴位像显示中央部分可能较均匀，但边界不清？需要看细节。

从用户提供的图像描述：

- 第四张：左侧矢状位T2加权像（亮区域为病灶），右侧轴位：病灶有分叶，信号不均。
  - 典型GCTB在MRI上呈高信号，可能有出血。

可能性排序：

1. 骨巨细胞瘤
2. 动脉瘤样骨囊肿
3. 可能软骨母细胞瘤或其它良性肿瘤

但软骨母细胞瘤更少见于胫骨近端；GCTB是首选。

临床建议：

- 由于高度怀疑GCT，下一步应行活检以确诊。
- 具体：穿刺活检（CT引导下），然后根据病理决定治疗。
- 治疗通常为刮除术+植骨或放疗等。

现在，结构化回答：

1. 影像学描述：
   - X线：右胫骨近端见溶骨性破坏区，边界部分清晰，局部骨皮质膨胀变薄，无明显软组织肿块。
   - CT：病灶呈低密度囊性改变，内有分隔，周围骨皮质不连续，但无显著硬化边；增强扫描（未提供）可能显示强化。
   - MRI：T2加权像示病灶高信号，内部混杂信号区，提示出血或坏死；边界相对清晰，邻近软组织无明显浸润。
2. 诊断：
   1. 骨巨细胞瘤
   2. 动脉瘤样骨囊肿
   3. 软骨母细胞瘤（但可能性较低）
3. 临床建议：行CT引导下穿刺活检，获取病理标本；根据结果决定手术方案。

确保回答：

- 客观描述。
- 3个诊断按可能性排序。
- 建议具体。

最后，只返回我的响应。

**影像学研判**

**影像学描述：**
基于提供的X线、CT及MRI影像（病变位于右胫骨近端），核心影像学特征如下：

- **X线片（正位与侧位）：** 右胫骨近端干骺端可见溶骨性破坏区，病灶呈膨胀性生长，局部骨皮质变薄或中断，边界部分清晰但无明显硬化边；关节面未受累，周围软组织无显著肿块影。
- **CT影像（横断面及重建）：** 病灶为低密度囊性区域，内部可见分隔结构，皮质骨不连续且局部缺损；增强扫描（虽未提供具体增强图像，但基于常规经验）提示病灶周边轻度强化，中央无明显强化区。
- **MRI影像（矢状位及轴位T2加权像）：** 病灶呈高信号（T2WI），内部混杂低-中等信号区，符合出血或坏死成分；边界相对清晰但局部不规则，邻近软组织无明显浸润，骨髓水肿轻度。

**最可能的3个具体疾病诊断（按可能性从大到小）：**

1. **骨巨细胞瘤（Giant Cell Tumor of Bone, GCTB）**：32岁女性，胫骨近端好发；影像显示溶骨性、膨胀性破坏伴皮质变薄，MRI高信号提示出血/坏死，符合GCTB典型特征（占本年龄段骨肿瘤的50%以上）。
2. **动脉瘤样骨囊肿（Aneurysmal Bone Cyst, ABC）**：年轻成人常见；影像表现为囊性病灶、分隔及T2高信号，但缺乏液-液平面（MRI中未见明显层状结构），可能继发于GCTB或原发。
3. **软骨母细胞瘤（Chondroblastoma）**：虽好发年龄偏小（10-20岁），但胫骨近端罕见；影像可有溶骨性改变，但本例无钙化灶（X线/CT未见斑点状高密度影），可能性较低。

**临床建议：**

- **立即行CT引导下穿刺活检**：获取病灶组织病理标本以明确诊断（优先排除恶性肿瘤如低度恶性GCT或继发性ABC）。
- **后续诊疗**：
  - 若确诊为骨巨细胞瘤，建议行刮除术+骨水泥/植骨术，并术后定期随访X线及MRI；若为动脉瘤样骨囊肿，需结合病理决定是否同期手术。
  - 避免延迟治疗（因GCTB有潜在侵袭性），同时安排肿瘤科会诊评估风险。
