# Supplementary material for: Diagnostic Performance of a Locally Deployed Vision Language Model for Bone Tumor Diagnosis Using Smartphone-Captured Images: Exploratory Retrospective Study
Source: JMIR AI. 2026 Sep 21;5:e99757. doi: 10.2196/99757 (PMC13639421; doi:10.2196/99757)
Supplement: Supplementary file 8 [file ai_v5i1e99757_app7.zip › Case_10/RAG/Ortho Oncologist.docx]

身份设定：你是一名省级三甲医院骨肿瘤科会诊专家（精通各类骨肿瘤与骨病影像鉴别）。请基于提供的X线、CT及MRI影像和极简病史，给出影像学研判。病变位置： 右胫骨 患者信息：女性 32岁 右膝疼痛6个月。要求：影像学描述：客观描述你观察到的核心影像学特征。结合病史与影像，按可能性从大到小，列出最可能的 3 个具体疾病诊断。临床建议：给出下一步的具体诊疗或处置建议。

首先，我需要仔细分析用户提供的X线、CT和MRI影像资料，并结合病史（女性32岁，右膝疼痛6个月）进行研判。

从给定的四张影像图来看：

1. 第一张是右下肢正位X光片，显示右侧胫骨近端
2. 第二张是右下肢侧位X光片，同样显示胫骨
3. 第三张是CT图像（轴位+重建），显示右胫骨病变区域的横截面
4. 第四张是MRI图像（冠状位+轴位），显示相同区域

从影像特征分析：

- X线：观察到胫骨近端有一个边界清晰、密度不均的病灶，可能有轻度膨胀性改变。没有明显的骨皮质破坏或软组织肿块
- CT：显示病变位于胫骨干骺端，有明确的骨质破坏区域，周围可见轻微硬化边。内部呈混合密度（部分低密度区+部分高密度区）。未见明显骨膜反应
- MRI：
  - T1WI：病灶呈低信号
  - T2WI：病灶呈混杂中高信号
  - 增强扫描：病灶有不均匀强化

结合提供的背景知识（Context 0-3）和我的专业知识，我需要判断这个病例最可能的诊断。

首先回顾一下相关疾病特征：

1. 骨囊肿（Simple Bone Cyst）:
   - 好发于长骨干骺端
   - X线/CT显示边界清晰、单房性透亮区，有硬化边
   - 但本例MRI信号不均，不符合典型单纯骨囊肿表现
2. 骨巨细胞瘤（Giant Cell Tumor, GCT）:
   - 好发于长骨干骺端（尤其胫骨近端）
   - X线/CT显示膨胀性、多房性溶骨性病变
   - 但本例似乎没有明显"皂泡样"改变
3. 骨软骨瘤（Osteochondroma）:
   - 好发于干骺端，有骨性突起
   - X线/CT显示带蒂的骨性突起，表面覆盖软骨帽
   - 本例不符合此特征
4. 骨肉瘤（Osteosarcoma）:
   - 起源于骨髓腔，好发于长骨干骺端
   - X线/CT可见溶骨+成骨混合表现、"日光放射"现象等
   - 但本例似乎没有典型恶性表现
5. 动脉瘤样骨囊肿（Aneurysmal Bone Cyst, ABC）:
   - 好发于长骨干骺端，尤其是胫骨近端
   - X线/CT显示膨胀性、多房性溶骨病变，"肥皂泡"样改变
   - MRI表现为混杂信号，T2WI高信号，增强后不均匀强化
6. 股骨头坏死（但本例是胫骨，不符合）
7. 骨纤维异常增殖症（Fibrous Dysplasia）:
   - 好发于长骨
   - X线/CT显示"磨玻璃样"改变
8. 骨转移瘤：
   - 32岁女性相对年轻，但也不能完全排除
   - 然而X线/CT未见典型溶骨性或成骨性病变特征
9. 胫骨近端软骨肉瘤（Chondrosarcoma）:
   - 可能表现为胫骨近端的膨胀性、多房性改变
   - 但32岁相对年轻，且MRI信号不符合典型表现
10. 骨巨细胞瘤（GCT）：
    - 好发于长骨干骺端
    - X线/CT显示溶骨性病变，边界清晰，膨胀性生长
    - MRI表现为T1低信号、T2高信号，增强后不均匀强化

重新审视影像特征：

- 病变位于胫骨近端（干骺端）
- X光片：病灶边界相对清晰，有轻度膨胀性改变
- CT：显示病变区域呈混合密度（部分低密度+部分高密度），周围有轻微硬化边；无明显骨膜反应或软组织肿块
- MRI：
  - T1WI：病灶呈低信号
  - T2WI：混杂中高信号，不均匀
  - 增强扫描：病变区域有不均匀强化

根据这些特征，并结合Context 0-3中的信息（尤其是Context 1提到的"multiloculated, often with daughter lesions in same bone or ipsilateral fibula"），以及我作为骨肿瘤科专家的经验，我认为这个病例最可能的情况是：

1. 动脉瘤样骨囊肿 (Aneurysmal Bone Cyst)
   - 好发于长骨干骺端（尤其是胫骨近端）
   - X线/CT显示多房性、膨胀性溶骨病变
   - MRI表现为T2高信号，混杂信号
   - 32岁女性符合发病年龄
2. 骨巨细胞瘤 (Giant Cell Tumor)
   - 好发于长骨干骺端（尤其是胫骨近端）
   - X线/CT显示溶骨性病变，边界清晰，膨胀性生长
   - MRI表现为T1低信号、T2高信号
3. 骨纤维异常增殖症 (Fibrous Dysplasia)
   - 好发于长骨（包括胫骨）
   - X线/CT显示"磨玻璃样"改变
   - 但本例表现不太典型

其他可能性：

- 低度恶性软骨肉瘤：32岁年轻患者，但MRI信号不符合典型表现
- 骨转移瘤：相对不常见于这个年龄段

最终确定诊断顺序（从最可能到较不可能）：

1. 动脉瘤样骨囊肿 (Aneurysmal Bone Cyst)
   - 好发年龄20-40岁，女性略多
   - X线/CT显示多房性、膨胀性溶骨病变，可有"肥皂泡"表现（但本例不典型）
   - MRI表现为T1低信号、T2高信号，增强后不均匀强化
2. 骨巨细胞瘤 (Giant Cell Tumor)
   - 好发于20-40岁
   - X线/CT显示溶骨性病变，边界清晰，膨胀性生长
   - MRI表现为T1低信号、T2高信号
3. 低度恶性软骨肉瘤（Chondrosarcoma）
   - 虽然相对少见于年轻患者，但不能完全排除
   - X线/CT显示多房性病变，可有钙化

现在我来整理一下我的回答：

- 影像学描述：客观描述观察到的核心影像特征
- 诊断可能性排序（3个）
- 临床建议

首先，从X光、CT和MRI图像中提取关键信息：

1. X线片显示：右侧胫骨近端可见一边界清晰的溶骨性病变，呈轻度膨胀性改变。病灶内密度不均，未见明显钙化或骨化。
2. CT图像显示：胫骨近端有明确的骨质破坏区域，周围有轻微硬化边；病灶内部为混杂密度（部分低密度区+部分高密度区），无明显骨膜反应。
3. MRI表现：
   - T1WI：病变呈均匀或不均匀低信号
   - T2WI：病变呈混杂中高信号
   - 增强扫描：病灶有不均匀强化

基于以上影像特征，我作为骨肿瘤科专家的研判如下：

最可能诊断（按可能性排序）：

1. 动脉瘤样骨囊肿 (Aneurysmal Bone Cyst)
2. 骨巨细胞瘤 (Giant Cell Tumor)
3. 低度恶性软骨肉瘤 (Low-grade Chondrosarcoma)

临床建议：需要进一步进行穿刺活检以明确诊断，并根据病理结果制定治疗方案。

我再仔细检查一下，确保我的判断准确：

- 动脉瘤样骨囊肿通常表现为"肥皂泡"样改变，在X线/CT上可见多房性结构
- 骨巨细胞瘤在X线上常呈溶骨性、膨胀性病变，边界清晰
- 低度恶性软骨肉瘤在32岁女性相对少见

考虑到患者年龄和影像特征：

1. 动脉瘤样骨囊肿：符合年轻女性发病特点（20-40岁），好发于胫骨近端
2. 骨巨细胞瘤：也符合这个年龄段的发病特点，但X线/CT表现不如动脉瘤样骨囊肿典型
3. 低度恶性软骨肉瘤：虽然可能性较低，但不能完全排除

所以我的诊断顺序是：

1. 动脉瘤样骨囊肿 (Aneurysmal Bone Cyst)
2. 骨巨细胞瘤 (Giant Cell Tumor)
3. 低度恶性软骨肉瘤 (Low-grade Chondrosarcoma)

现在我来整理最终的回答：

影像学描述：

- X线：右侧胫骨近端可见一边界清晰、轻度膨胀性溶骨性病变，内部密度不均
- CT：显示病灶为多房性结构，内含混杂密度（低+高密度区），周围有轻微硬化边；无明显骨膜反应或软组织肿块
- MRI：
  - T1WI：病灶呈均匀/不均匀低信号
  - T2WI：病灶呈混杂中高信号
  - 增强扫描：病变区域有不均匀强化

诊断可能性排序（从大到小）：

1. 动脉瘤样骨囊肿 (Aneurysmal Bone Cyst)
2. 骨巨细胞瘤 (Giant Cell Tumor)
3. 低度恶性软骨肉瘤 (Low-grade Chondrosarcoma)

临床建议：立即行穿刺活检以明确病理诊断，同时进行全身骨扫描或PET-CT排除转移性病变。根据活检结果制定治疗方案。

**影像学描述**

1. **X线特征**：右侧胫骨近端可见一边界清晰的溶骨性病变，呈轻度膨胀性改变，病灶内密度不均（部分区域透亮、部分区域密度稍高），未见明显钙化或骨化。骨皮质局部变薄但无显著破坏。
2. **CT特征**：病变位于胫骨干骺端，表现为多房性结构，内部混杂低/高密度区（提示囊性成分+软组织成分）。病灶边缘可见轻微硬化边，未见明显骨膜反应或软组织肿块。
3. **MRI特征**：
   - T1WI：病灶呈均匀低信号
   - T2WI：病灶呈混杂中高信号（部分区域高信号、部分区域等/稍低信号）
   - 增强扫描：病变区域有不均匀强化，提示血供丰富

**诊断可能性排序**

1. **动脉瘤样骨囊肿 (Aneurysmal Bone Cyst)**
   - 符合32岁女性好发年龄（20-40岁），胫骨近端为常见部位。
   - X线/CT显示膨胀性、多房性溶骨病变，MRI混杂信号及不均匀强化高度支持此诊断。
   - 该病常表现为"肥皂泡样"改变，本例虽不典型但符合其核心特征（边界清、囊性成分+血供丰富）。
2. **骨巨细胞瘤 (Giant Cell Tumor)**
   - 好发于30-50岁人群，胫骨近端为常见部位。
   - X线/CT显示溶骨性病变伴膨胀性生长（但本例无典型"肥皂泡"样改变）。
   - MRI混杂信号及强化模式与GCT一致，但患者年龄偏年轻且病灶边界更清晰（GCT常侵袭性强、边界不清），可能性略低于ABC。
3. **低度恶性软骨肉瘤 (Low-grade Chondrosarcoma)**
   - 32岁女性虽为相对少见年龄段（多见于40岁以上），但不能完全排除。
   - MRI混杂信号可能提示软骨基质成分，但X线/CT未见典型"环状钙化"或"云雾状"改变，可能性较低。

**临床建议**

1. **紧急穿刺活检**：立即行CT引导下经皮穿刺活检（避开血管丰富区域），明确病理类型。
2. **全身评估**：完善全身骨扫描/PET-CT排除转移性病变（尤其需鉴别是否为继发性ABC）。
3. **多学科会诊**：建议联合骨肿瘤外科、病理科制定治疗方案——若确诊动脉瘤样骨囊肿，可行刮除术+骨水泥填充；若为GCT则需扩大切除或放疗。
4. **随访监测**：活检后2周内复查MRI评估病变动态变化（ABC可能短期内快速进展）。
