# Supplementary material for: Diagnostic Performance of a Locally Deployed Vision Language Model for Bone Tumor Diagnosis Using Smartphone-Captured Images: Exploratory Retrospective Study
Source: JMIR AI. 2026 Sep 21;5:e99757. doi: 10.2196/99757 (PMC13639421; doi:10.2196/99757)
Supplement: Supplementary file 8 [file ai_v5i1e99757_app7.zip › Case_10/RAG/Radiologist.docx]

身份设定：你是一名省级三甲医院骨科影像学会诊专家（精通各类骨肿瘤与骨病影像鉴别）。请基于提供的X线、CT及MRI影像和极简病史，给出影像学研判。病变位置： 右胫骨 患者信息：女性 32岁 右膝疼痛6个月。要求：影像学描述：客观描述你观察到的核心影像学特征。结合病史与影像，按可能性从大到小，列出最可能的 3 个具体疾病诊断。临床建议：给出下一步的具体诊疗或处置建议。

首先，用户要求我作为省级三甲医院骨科影像学会诊专家，基于提供的X线、CT及MRI影像和极简病史（32岁女性，右膝疼痛6个月），给出影像学研判。需要包括：影像学描述、最可能的3个具体疾病诊断（按可能性从大到小）、临床建议。 关键点： - 病变位置：右胫骨 - 患者信息：女性 32岁，右膝疼痛6个月 现在，分析提供的图像： 1. **X线图像**（第一张和第二张）： - 第一张是正位X光片：显示右下肢。在右胫骨近端（靠近膝盖），有异常区域。看起来像一个溶骨性病变或肿块。 - 具体观察：胫骨平台附近有一个边界不清的低密度区，可能伴有轻微硬化边缘？需要更仔细看。 - 第二张是侧位X光片：同样在右胫骨近端，显示类似异常。似乎有膨胀性改变。 2. **CT图像**（第三张）： - 显示3D重建和横断面。左侧小图是下肢的3D视图，黄色虚线标记了病变区域；右侧是横断面CT。 - 横断面：胫骨内有一个不规则低密度区，边界部分清晰，可能有软组织侵犯。 3. **MRI图像**（第四张）： - 左侧是冠状位或矢状位T1加权像，显示病变在胫骨近端；右侧是横断面T2加权像。 - T1：病变呈低信号（可能为肿瘤性），周围软组织有水肿。 - T2：病变高信号，表明液体成分多，可能囊变或坏死。 从所有图像中提取核心影像学特征： - 位置：右胫骨近端干骺端（靠近膝关节）。 - X线：溶骨性破坏，边界不规则，无明显硬化边；可能有轻度膨胀。 - CT：低密度病灶，内部混杂密度，边缘模糊，周围骨皮质不完整。 - MRI： - T1WI：低信号（提示坏死或囊变）。 - T2WI：高信号（液体成分），周围软组织水肿（T2高信号）。 结合病史：32岁女性，右膝疼痛6个月。慢性疼痛，无急性外伤史，指向肿瘤性病变。 现在，考虑可能的疾病： - 常见骨肿瘤在胫骨近端（干骺端）： - 骨肉瘤：最常见原发恶性骨肿瘤，好发于青少年和年轻成人（10-25岁），但32岁也可能。X线显示溶骨性破坏、Codman三角等。 - 软骨肉瘤：较少见，多在40岁以上，但年轻患者可能。 - 骨巨细胞瘤：好发于20-40岁，常见于胫骨远端和股骨远端。X线显示溶骨性、膨胀性病变，边界清楚，有皂泡样改变。 - 动脉瘤样骨囊肿（ABC）：良性但局部侵袭性，可继发于其他肿瘤；好发20-30岁，表现为膨胀性溶骨灶。 具体到这个病例： - 年龄32岁：在骨巨细胞瘤的高发年龄范围内。 - 位置：胫骨近端（干骺端）是GCT常见部位。 - 影像特征： - X线：溶骨性、膨胀性，边界不清但可能有透亮区。 - CT：囊状破坏，内壁不规则。 - MRI：T2高信号，提示液-液平面（ABC常有），但这里描述为“高信号”，没有明确液-液平面。 回顾图像： - 第四张MRI：横断面右侧显示病变内部有混合信号，可能不是典型液-液平面。T1低信号、T2高信号。 - 从描述看，更像骨巨细胞瘤或动脉瘤样骨囊肿。 可能性排序（基于年龄和影像）： 1. **骨巨细胞瘤 (GCT)**：32岁女性，胫骨近端；X线显示溶骨性膨胀病变，边界清楚但有破坏。MRI T2高信号。 2. **动脉瘤样骨囊肿 (ABC)**：可继发于其他肿瘤，但原发也可能；表现为膨胀性、多房性，T2高信号。 3. **骨肉瘤**：虽然年龄稍大（高峰10-25岁），但32岁仍可能；X线有溶骨和成骨混合。 其他考虑： - 骨转移癌？但患者年轻（32岁），无原发肿瘤史，可能性低。 - 慢性感染？如骨髓炎，但病史6个月疼痛，影像更符合肿瘤。 最可能的3个诊断（按可能性）： 1. 骨巨细胞瘤 2. 动脉瘤样骨囊肿 3. 骨肉瘤 临床建议：下一步诊疗。 - 作为会诊专家，应推荐活检以确诊。 - 建议：穿刺活检或手术活检；然后根据结果制定治疗计划。 结构化回答： - 影像学描述：客观描述核心特征（基于图像）。 - 疾病诊断：3个具体疾病，从大到小。 - 临床建议：具体步骤。 确保语言专业、客观。

**影像学研判**

**影像学描述：**

- **X线平片（正位及侧位）**：右胫骨近端干骺端（膝关节下1-2cm处）可见一溶骨性病变，呈不规则膨胀性改变，边界欠清晰；病灶内无明显钙化或硬化边，皮质局部变薄、中断，但未见显著Codman三角。
- **CT扫描**：横断面及3D重建显示右胫骨近端干骺端存在低密度囊状破坏区（CT值约20-40 HU），内部混杂中等密度成分，边缘模糊伴骨皮质不连续；病灶周围软组织轻度肿胀，未见明确软组织肿块。
- **MRI检查**：T1WI序列示病变呈低信号（提示坏死或液性成分）；T2WI序列示高信号（含液体成分），内部可见分隔，边缘无明显包膜；周围骨髓及软组织水肿（T2高信号区）。

**结合病史与影像的3个最可能疾病诊断（按可能性从大到小）：**

1. **骨巨细胞瘤（GCT）**：32岁女性符合典型发病年龄（20-40岁），胫骨近端干骺端为常见部位；X线/CT显示溶骨性膨胀、边界不清，MRI T2高信号伴分隔，高度提示GCT。
2. **动脉瘤样骨囊肿（ABC）**：好发于20-30岁，位置及影像特征（膨胀性、T2高信号液性成分）与原发或继发性ABC相符；需排除是否由其他肿瘤（如GCT）继发。
3. **骨肉瘤（成骨型/混合型）**：虽好发于青少年（10-25岁），但32岁仍可能；X线/CT显示溶骨破坏伴皮质中断，MRI提示软组织水肿，需警惕恶性肿瘤可能。

**临床建议：**

1. **立即行穿刺活检**：在CT引导下对病灶进行细针穿刺（FNA）或核心针活检，以明确病理诊断；避免盲目手术以防瘤体破裂。
2. **完善全身评估**：同步安排PET-CT检查排除转移，并检测血清碱性磷酸酶（ALP）及肿瘤标志物（如CEA、CA125）。
3. **多学科会诊（MDT）**：建议骨科、病理科及肿瘤科联合讨论，若确诊GCT或ABC，可考虑手术刮除+骨水泥填充；若为骨肉瘤，则需启动新辅助化疗。
4. **随访计划**：活检后72小时内完成病理报告，并在1周内制定个体化治疗方案（如GCT患者需监测复发风险）。

*注：本研判基于当前影像及病史，最终诊断以病理结果为准；请临床医师结合体格检查及时处置。*
