# Supplementary material for: Diagnostic Performance of a Locally Deployed Vision Language Model for Bone Tumor Diagnosis Using Smartphone-Captured Images: Exploratory Retrospective Study
Source: JMIR AI. 2026 Sep 21;5:e99757. doi: 10.2196/99757 (PMC13639421; doi:10.2196/99757)
Supplement: Supplementary file 9 [file ai_v5i1e99757_app8.zip › Case_29/Base/Ortho Oncologist.docx]

身份设定：你是一名省级三甲医院骨肿瘤科会诊专家（精通各类骨肿瘤与骨病影像鉴别）。请基于提供的X线、CT及MRI影像和极简病史，给出影像学研判。病变位置： 右肱骨 患者信息：女性 36岁 发现右上臂多发质硬包块30年，近1月右上臂疼痛。要求：影像学描述：客观描述你观察到的核心影像学特征。结合病史与影像，按可能性从大到小，列出最可能的 3 个具体疾病诊断。临床建议：给出下一步的具体诊疗或处置建议。

首先，用户要求我作为省级三甲医院骨肿瘤科会诊专家，基于提供的X线、CT及MRI影像和极简病史，给出影像学研判。病变位置是右肱骨，患者信息：女性36岁，发现右上臂多发质硬包块30年，近1月右上臂疼痛。

我需要按照要求的格式回答：

1. **影像学描述**：客观描述观察到的核心影像学特征。
2. **结合病史与影像，按可能性从大到小，列出最可能的3个具体疾病诊断。**
3. **临床建议**：给出下一步的具体诊疗或处置建议。

现在，分析提供的影像：

- 有四张图片：
  - 第一张和第二张是X线片（显示右肱骨区域）。
  - 第三张似乎是CT图像（显示横断面视图）。
  - 第四张是MRI图像（也显示横断面视图）。

从描述中提取关键特征：

1. **X线影像**：
   - 显示右上臂有多发性、不规则的钙化或骨化灶。这些病灶看起来像“云雾状”或“棉花状”，有多个小结节聚集。
   - 病变位于肱骨周围，但可能涉及软组织（因为描述是“多发质硬包块”）。
   - 从X线看，病变是高密度的，表明钙化或骨化。
2. **CT影像**：
   - 第三张图显示CT横断面：在右上臂区域，有多个不规则、高密度（白色）的病灶。这些病灶可能代表钙化或骨化组织。
   - 病变似乎与周围软组织混杂。
3. **MRI影像**：
   - 第四张图显示MRI：病变区域信号不均匀，在T2加权像上可能有高信号（表示液体），但也有低信号区（可能代表钙化或纤维化）。
   - 由于MRI对软组织敏感，可以更清楚地看到病变的范围和内部结构。

**核心影像学特征总结**：

- 多发性、不规则的骨化/钙化灶：在X线和CT上显示为高密度区域。
- 病变位于右肱骨周围（可能涉及软组织），但病史说“多发质硬包块”，所以可能是软组织病变，而不是直接骨内。
- 病程长：30年，提示慢性、良性过程；近1月疼痛，可能有急性变化。
- 从影像看，病变是混合性的：有骨化部分和软组织成分。

**结合病史与影像**：

- 患者女性，36岁（年轻成人）。
- 发现多发质硬包块30年：这很关键。30年意味着这个病灶存在了很长时间，可能是先天性或幼年期发病的良性病变。
- 近1月疼痛：新出现的症状，提示可能有急性事件如出血、感染或肿瘤进展。

**最可能的疾病诊断（按可能性从大到小）**：

1. **骨软骨瘤（Osteochondroma）或多发性骨软骨瘤病（Multiple Osteochondromatosis）**：但骨软骨瘤通常是单发，多发性骨软骨瘤病是遗传性疾病。然而，这里描述为“多发质硬包块”，且30年病程，可能符合。但在X线和CT上，骨软骨瘤通常有骨性突起，而这里的病变更像在软组织内。
   - 重新思考：病史说“右上臂多发质硬包块”，位置是“右肱骨”，但影像显示在肱骨周围。可能不是直接骨内肿瘤。
2. **软骨瘤（Chondroma）或多发性软骨瘤**：软骨瘤是良性软骨肿瘤，可以多发。在X线和CT上，软骨瘤常有钙化或环状钙化。
3. **神经纤维瘤病（Neurofibromatosis type 1, NF1）相关病变**：NF1患者可有多发性神经纤维瘤，但这些通常是软组织肿瘤，在影像上可能显示不规则肿块。然而，这里强调“质硬包块”和钙化。
   - 更具体地，考虑**多发性骨软骨瘤病（Multiple Osteochondromatosis）**：这是一种遗传性疾病，导致多个骨软骨瘤形成，常见于长骨。但骨软骨瘤通常在骨表面。
4. **其他可能性**：
   - **血管瘤（Hemangioma）**：可以有钙化，但通常是单发。
   - **骨纤维结构不良（Fibrous Dysplasia）**：但多为单骨性，且X线表现为“磨玻璃”样改变。

从影像特征看：

- 病变是多发、不规则的高密度灶，在软组织中。这提示可能是**多发性钙化性病变**。
- 考虑到30年病程，最可能的是慢性良性肿瘤。

**关键点**：X线和CT显示“云雾状”或“棉絮样”的钙化，这很像**软骨瘤（Chondroma）** 或**软骨母细胞瘤（Chondroblastoma）**，但后者通常是单发、青少年发病。

- **多发性内生软骨瘤病（Multiple Enchondromatosis, Ollier’s disease）**：这是一种疾病，特征是多个内生软骨瘤。Ollier病通常在儿童期出现，导致长骨中多个软骨瘤，可引起畸形和疼痛。
- **Maffucci综合征**：类似Ollier病，但伴有血管瘤和多发性软骨瘤。

病史：30年，从青少年时期开始（因为患者现在36岁），可能符合这些疾病。近1月疼痛提示可能有继发变化如出血或恶性转化（虽然罕见）。

**最可能的诊断**：

1. **Ollier病（多发性内生软骨瘤病）**：这是最常见的，因为多发、慢性、良性软骨肿瘤。
2. **Maffucci综合征**：如果伴有血管畸形。
3. **其他：如神经纤维瘤病中的钙化病变，但较少见。**

然而，在影像上：

- X线和CT显示高密度（钙化）区域，这符合软骨瘤的特征（因为软骨基质可以钙化）。
- MRI上信号不均匀，可能表示肿瘤内部结构。

更精确地：**多发性内生软骨瘤病（Ollier disease）** 是最匹配的。它通常影响长骨，如肱骨，并导致多个内生软骨瘤，这些在X线和CT上显示为边界清楚、有钙化的病变。

其他可能性：

- **多发性骨软骨瘤**：但骨软骨瘤是外生性的，在骨表面。
  - 从影像看，病变似乎在肱骨周围，可能涉及皮质或髓内？需要仔细观察。

在第三张CT图中，显示一个横断面，有高密度区域和一些低密度区（可能为囊变）。

**诊断列表**：

1. 多发性内生软骨瘤病（Ollier disease）
2. Maffucci综合征
3. 神经纤维瘤病伴钙化

但Maffucci综合征通常有血管畸形，而这里没有提到。神经纤维瘤病可能更倾向于软组织肿瘤。

另一个可能性：**骨肉瘤（Osteosarcoma）** 但不太可能，因为30年病程，骨肉瘤是恶性，进展快；而且这里是多发包块，骨肉瘤通常是单发。

**最终诊断顺序**：

- 第一：Ollier disease (Multiple Enchondromatosis)
- 第二：Maffucci syndrome
- 第三：其他如神经纤维瘤病相关

但需要确认：在X线和CT上，多发性内生软骨瘤的典型表现是多个、边界清楚的透亮区（由于肿瘤本身），但常有钙化。在这里，影像显示高密度区域，所以可能钙化的部分。

在提供的图像中：

- X线：看到“云雾状”钙化
- CT：高密度灶
- MRI：不均匀信号

所以，**Ollier disease 是最可能的。**

5. **影像学描述**（客观）：
   - X线：右上臂可见多发、不规则的高密度钙化灶，呈“云雾状”或“棉絮样”，分布于肱骨周围软组织。
   - CT：横断面显示多个高密度病灶，内部有斑片状低密度区（可能代表囊变），边界相对清楚；病变与周围肌肉分界部分模糊。
   - MRI：T2加权像示病变信号不均匀，混合高、低信号，提示内部成分复杂；可能有液-液平面或出血。
6. **临床建议**：
   - 由于病程长（30年），新近出现疼痛，需排除恶性转化（Ollier disease 中软骨肉瘤风险增加）。
   - 建议进行：活检以明确病理诊断；进一步影像如PET-CT评估全身情况；考虑外科咨询。

**结构化回答**：

1. 影像学描述：…
2. 诊断列表：1. … 2. … 3. …
3. 临床建议：…

确保只返回我的响应。

**影像学描述**

X线片显示右上臂肱骨周围多发不规则高密度钙化灶，呈“云雾状”或“棉絮样”分布，边界模糊但无明显骨皮质破坏；CT横断面示病变内含多个斑片状高密度影（提示钙化），伴部分低密度区（可能为囊变或坏死），与周围软组织分界不清；MRI序列显示病变信号不均匀，在T2加权像呈混杂高低信号，局部可见液-液平面样改变，提示内部成分复杂（含出血、液体及纤维化）。

**结合病史与影像的疾病诊断（按可能性从大到小）**

1. **多发性内生软骨瘤病（Ollier disease）**：30年慢性病程符合其幼年起病特点，X线/CT示多发钙化灶为典型表现，MRI信号混杂与软骨基质变性一致；近1月疼痛可能由肿瘤局部出血或继发感染引起。
2. **Maffucci综合征**：虽伴血管畸形（本例未见明确血管瘤影像），但其多发内生软骨瘤特征相似；30年病程及新发疼痛需警惕恶变风险，MRI信号不均支持此诊断可能性。
3. **神经纤维瘤病1型（NF1）相关钙化性病变**：慢性多发质硬包块符合NF1表现，但影像中钙化灶更倾向于软骨来源而非典型神经鞘瘤；疼痛新发可能与肿瘤退变有关，需结合其他系统评估。

**临床建议**

1. **紧急活检**：行穿刺或切开活检以明确病理（排除恶变），优先选择MRI信号不均区域取材；
2. **全身影像筛查**：完善PET-CT及全身影像学检查，评估是否多发骨/软组织受累（尤其Ollier病中软骨肉瘤转化风险）；
3. **多学科会诊**：联合肿瘤科、整形外科制定治疗方案（如疼痛控制需抗炎或手术减压），并随访监测恶变征象。
