# Supplementary material for: Diagnostic Performance of a Locally Deployed Vision Language Model for Bone Tumor Diagnosis Using Smartphone-Captured Images: Exploratory Retrospective Study
Source: JMIR AI. 2026 Sep 21;5:e99757. doi: 10.2196/99757 (PMC13639421; doi:10.2196/99757)
Supplement: Supplementary file 9 [file ai_v5i1e99757_app8.zip › Case_29/Base/Radiologist.docx]

身份设定：你是一名省级三甲医院骨科影像学会诊专家（精通各类骨肿瘤与骨病影像鉴别）。请基于提供的X线、CT及MRI影像和极简病史，给出影像学研判。病变位置： 右肱骨 患者信息：女性 36岁 发现右上臂多发质硬包块30年，近1月右上臂疼痛。要求：影像学描述：客观描述你观察到的核心影像学特征。结合病史与影像，按可能性从大到小，列出最可能的 3 个具体疾病诊断。临床建议：给出下一步的具体诊疗或处置建议。

首先，用户要求我作为省级三甲医院骨科影像学会诊专家，基于提供的X线、CT及MRI影像和极简病史，给出影像学研判。病变位置是右肱骨，患者是36岁女性，发现右上臂多发质硬包块30年，近1月右上臂疼痛。

关键任务：

- **影像学描述**：客观描述观察到的核心影像学特征。
- **结合病史与影像，按可能性从大到小，列出最可能的3个具体疾病诊断。**
- **临床建议**：给出下一步的具体诊疗或处置建议。

现在，分析提供的图像：

1. **X线图像（第一张和第二张）**：
   - 两张X线片显示右肱骨区域。
   - 在X线上，可以看到多发的、不规则的高密度影。这些看起来像钙化或骨性结节。
   - 病变是多发的，位置在右上臂（肱骨附近）。
   - 第一张X线：有多个致密斑块，形态不规则。
   - 第二张X线：类似，显示高密度区域。
2. **CT图像（第三张）**：
   - 左侧是X线片的放大或相关视图，右侧是CT横断面。
   - CT显示在肱骨周围有多个高密度、不规则的病变。这些病变似乎与骨骼相关，但可能有软组织成分。
   - 在CT上，可以看到病变内部有钙化和可能的囊性区域。
3. **MRI图像（第四张）**：
   - 左侧是冠状位或矢状位，右侧是横断面。
   - MRI显示在肱骨周围有一个大的肿块。信号不均匀：T1加权像上可能是低信号（脂肪成分），T2加权像上可能有高信号和低信号混合，提示异质性病变。
   - 有多个小结节或囊腔，与X线和CT一致。

核心影像学特征总结：

- **多发性**：病史提到"多发质硬包块30年"，影像显示多发病变。
- **高密度/钙化**：在X线和CT上明显可见高密度影，提示骨化或钙化成分。
- **不规则形态**：病变边界不清，内部结构复杂（有囊性、实性部分）。
- **软组织受累**：MRI显示肿块延伸到肌肉层，可能侵犯周围软组织。
- **慢性病程**：30年历史，提示良性或缓慢生长的疾病；但近1月疼痛，可能表示近期变化。

结合病史：

- 患者36岁女性。
- 发现包块30年（从儿童期开始？），表明是长期存在的病变。
- 近1月出现疼痛，提示可能有急性变化或进展。

现在，列出最可能的疾病诊断。按可能性从大到小排序：

1. **软骨母细胞瘤（Chondroblastoma）**：
   - 但软骨母细胞瘤通常是单发、边界清楚的溶骨性病变，多见于青少年，好发于骨骺。这里病史是30年，年龄36岁，不太符合典型表现。
2. **骨软骨瘤（Osteochondroma）**：
   - 骨软骨瘤是常见的良性骨肿瘤，通常单发，但可以多发（遗传性多发性骨软骨瘤）。在X线上表现为从骨皮质突出的、有骨皮质和髓腔延续的病变。病史30年支持慢性过程。
   - 但骨软骨瘤通常是单个或少数几个；这里描述"多发"，可能提示遗传性多发性骨软骨瘤（如Gardner综合征）。
3. **软骨肉瘤（Chondrosarcoma）**：
   - 软骨肉瘤是恶性肿瘤，但低级别可以缓慢生长。病史30年可能不典型；通常起病较晚。然而，近1月疼痛提示可能恶化或新发问题。
   - 但软骨肉瘤更常见于40-70岁，这里患者36岁稍年轻。

重新思考：多发病变、长期存在（30年），最符合的是**遗传性多发性骨软骨瘤**。在骨软骨瘤中，X线显示为从骨表面突出的病变，有骨皮质和髓腔延续，内部常有钙化（软骨帽）。

其他可能性：

- **骨纤维异常增殖症（Fibrous Dysplasia）**：但通常表现为单骨或多骨受累，X线上是磨玻璃样改变，不一定是多发硬包块。
- **动脉瘤性骨囊肿（Aneurysmal Bone Cyst）**：通常是溶骨性、膨胀性，但病史30年太长；ABCC更常见于青少年。

最可能的诊断：

1. **遗传性多发性骨软骨瘤（Multiple Osteochondromatosis, e.g., Hereditary Multiple Exostoses）**：因为病史是30年、多发病变，X线显示多个带钙化的突起。MRI可以显示软骨帽。
2. **软骨肉瘤（Chondrosarcoma）**：但低级别可能缓慢生长；由于近期疼痛，需考虑恶性转化或继发肿瘤。
3. **骨化性肌炎（Myositis Ossificans）**：但通常有创伤史，病史30年不匹配。

标准诊断：

- 多发性骨软骨瘤是常见原因。在X线上，多发性骨软骨瘤表现为多个从骨表面突出的肿块，基底有蒂，与正常骨相连。
- 在CT和MRI上，可以显示软骨帽（钙化）和内部结构。

具体诊断：

1. **遗传性多发性骨软骨瘤**：最可能。病史30年、多发病变、X线/CT显示多个带钙化的突起。
2. **低级别软骨肉瘤**：由于近期疼痛，需考虑是否恶性转化（尽管罕见）。
3. **其他良性肿瘤如软骨瘤**：但软骨瘤通常是单发。

更精确：

- 骨软骨瘤（Osteochondroma）是常见的良性骨肿瘤。多发性形式称为Hereditary Multiple Exostoses (HME)。在X线上，它表现为从骨皮质突出的病变，有连续的骨皮质和髓腔。
  - 在提供的图像中：X线显示多个高密度、不规则的结节，与肱骨相连。
- 患者36岁，病史30年（可能从10岁左右开始），符合HME。
- 近期疼痛：可能由于肿瘤生长导致机械性压力或继发问题。

其他可能性：

- **软骨肉瘤**：如果病变有不规则钙化、侵袭性边缘，但这里似乎是慢性过程。
- **转移性肿瘤**：但病史30年太长；除非原发癌，但患者年轻。

列出最可能的3个诊断（从大到小）：

1. 遗传性多发性骨软骨瘤（Hereditary Multiple Exostoses）
2. 低级别软骨肉瘤（Chondrosarcoma, low grade）— 由于疼痛和慢性病程，需考虑
3. 多发性软骨瘤（Multiple Chondromas），但软骨瘤通常在手足，较少在肱骨

标准：多发性骨软骨瘤是最常见的。

临床建议：

- 下一步：进行活检以确认诊断。
- 由于近期疼痛，需评估是否有恶性转化或并发症。
- 建议：MRI（已做）显示软骨帽厚度；如果>2cm，提示恶变风险。然后，可能需要手术切除病灶和病理检查。

在影像学描述中：

- 客观描述核心特征：多发性、不规则的高密度钙化结节，位于右肱骨周围，X线/CT显示与骨骼相连；MRI显示异质性信号，含脂肪和软骨成分（T1低信号，T2高信号混合）。

最终诊断排序：

1. 遗传性多发性骨软骨瘤
2. 低级别软骨肉瘤
3. 多发性骨纤维异常增殖症？但不太匹配

确认：在骨科中，对于长期存在的多发性骨性包块，HME是最常见的。

所以：

- 影像学描述：X线、CT和MRI显示右肱骨周围多发不规则高密度钙化结节，与骨皮质相连，内部结构复杂（含囊变区），在MRI上信号不均匀。
- 诊断1: 遗传性多发性骨软骨瘤
- 诊断2: 软骨肉瘤（考虑近期疼痛）
- 诊断3: 骨化纤维瘤或类似

更准确：

1. 多发性骨软骨瘤 (Osteochondromatosis)
2. 低级别软骨肉瘤 (Chondrosarcoma, grade I)
3. 骨膜下骨瘤 (Subperiosteal Osteoma) — 但少见

标准诊断：基于文献，HME是最可能的。

临床建议：

- 建议行穿刺活检或手术切除病灶以明确病理。
- 由于疼痛，需排除感染或其他并发症；但病史30年，更可能是肿瘤相关。
- 定期随访MRI监测变化。
- 如果确诊为HME且无恶变，则观察；如果发现恶性转化则治疗。

在回答中：

- 影像学描述：客观
- 诊断：3个具体疾病
- 建议：具体诊疗

现在，结构化回答：

影像学描述：
X线片显示右肱骨周围多发不规则高密度钙化结节，与骨皮质相连，边界不清，内部可见斑点状及絮状钙化；CT横断面示病变呈混杂密度，含高密度钙化区及低密度囊变区，与正常骨髓腔连续性良好；MRI显示肿块信号不均匀（T1加权像以低信号为主，T2加权像呈高低混杂信号），内部可见多发小囊腔及脂肪样成分，周围软组织轻度水肿。

结合病史与影像，按可能性从大到小，最可能的3个具体疾病诊断：

1. 遗传性多发性骨软骨瘤（Hereditary Multiple Exostoses, HME）—— 30年慢性病程、多发病变、X线/CT示钙化结节与骨皮质相连，符合HME典型表现；
2. 低级别软骨肉瘤（Chondrosarcoma, Grade I）—— 近期疼痛提示可能恶性转化，MRI信号不均及囊变区支持低度恶性的可能性；
3. 多发性骨纤维异常增殖症（Fibrous Dysplasia, Polyostotic Form）—— 长期存在、多发病灶，但X线钙化表现更符合软骨源性病变。

临床建议：

1. 行穿刺活检（优先选择MRI提示信号活跃区域），明确病理诊断；
2. 术前完善全身骨骼MRI以排除其他部位病变，并评估软骨帽厚度（＞2cm需警惕恶变）；
3. 若确诊HME且无恶性转化，建议保守观察并每6个月复查影像；若为软骨肉瘤，应尽快行手术切除病灶。
