# Supplementary material for: Diagnostic Performance of a Locally Deployed Vision Language Model for Bone Tumor Diagnosis Using Smartphone-Captured Images: Exploratory Retrospective Study
Source: JMIR AI. 2026 Sep 21;5:e99757. doi: 10.2196/99757 (PMC13639421; doi:10.2196/99757)
Supplement: Supplementary file 9 [file ai_v5i1e99757_app8.zip › Case_29/RAG/Ortho Oncologist.docx]

身份设定：你是一名省级三甲医院骨肿瘤科会诊专家（精通各类骨肿瘤与骨病影像鉴别）。请基于提供的X线、CT及MRI影像和极简病史，给出影像学研判。病变位置： 右肱骨 患者信息：女性 36岁 发现右上臂多发质硬包块30年，近1月右上臂疼痛。要求：影像学描述：客观描述你观察到的核心影像学特征。结合病史与影像，按可能性从大到小，列出最可能的 3 个具体疾病诊断。临床建议：给出下一步的具体诊疗或处置建议。

首先，我需要仔细分析用户提供的X线、CT和MRI影像以及病史信息，然后基于我的专业知识进行研判。

患者情况：

- 女性，36岁
- 病变位置：右肱骨
- 发现右上臂多发质硬包块30年（长期存在）
- 近1个月出现疼痛

从提供的影像来看：

X线片显示：

- 右肱骨区域有多个高密度钙化或骨化灶，呈不规则团块状分布
- 位置在肱骨周围软组织中，与骨骼有一定关系但不直接位于骨内
- 高密度影呈现多发、聚集的特征

CT影像（第三张图）：

- 显示了更清晰的高密度钙化/骨化灶
- 软组织中的团块状结构有明确的边界
- 可以看到内部有不规则的致密区域和低密度区，提示可能包含软组织成分
- 与骨骼的关系：似乎从肱骨周围延伸出来

MRI影像（第四张图）：

- 显示了病变在软组织中的分布情况
- 病变内部信号混杂，既有高信号区域也有低信号区域
- 边界相对清晰但不规则
- 部分区域显示为囊性改变或坏死区

结合提供的上下文信息：

从[CONTEXT 1]中看到关于"Synovial Chondromatosis"(滑膜软骨瘤病)的描述：

- “multiple round chondroid calcifications of similar, but not identical, sizes located both intraarticularly and extraarticularly”
- “round ossified bodies proven to be within the knee joint. In addition, there is a lobular mass that appears more conglomerate located medial to the joint.”
- “The rounded bodies to have marrow signal”

从[CONTEXT 2]中看到关于"Chondromyxoid Fibroma"(软骨粘液样纤维瘤)的描述：

- “Lytic lesion, often multilobulated and expansile”
- “Cortex thinned; expansion usually not substantial”
- “Geographic with at least partial sclerotic margin”

从[CONTEXT 3]中看到关于"Ossification of Patellar Tendon"(髌腱钙化)的描述：

- “Calcification or ossification of proximal patellar tendon”
- “No donor site at inferior pole of patella”

现在，我来分析这个病例：

1. **影像学特征**:
   - 多发性、不规则的高密度影（X线和CT）
   - 位于右肱骨周围软组织
   - 长期存在（30年），近期出现疼痛
   - MRI显示内部信号混杂，有囊变/坏死区
2. **鉴别诊断**:
   - 滑膜软骨瘤病(Synovial Chondromatosis)：多发性、钙化或骨化的游离体，长期存在，符合患者的30年病史。虽然滑膜软骨瘤病通常发生在关节内，但也可以有额外关节的延伸。
   - 多发性骨软骨瘤(Multiple Osteochondroma)：但这些病变位于软组织而非骨骼表面
   - 软骨肉瘤(Chondrosarcoma)：可能性较低，因为患者年龄较轻（36岁）且病程较长，但需要排除恶性可能

基于我的专业知识和提供的影像：

1. **最可能的诊断**：
   - 滑膜软骨瘤病(Synovial Chondromatosis)：这是最常见的考虑。它通常表现为关节内多发钙化或骨化的游离体，可以有额外关节延伸。患者30年长期存在的包块符合这种疾病的慢性过程。
2. **第二可能的诊断**：
   - 多发性软骨瘤(Multiple Chondromas)：特别是如果病变起源于骨骼周围组织
3. **第三可能的诊断**：
   - 软骨肉瘤(Chondrosarcoma)：虽然可能性较低，但因为患者近期出现疼痛，需要排除恶性肿瘤的可能性。不过，考虑到病程长、无明显侵袭性表现，这种可能性相对较小。

现在我来详细分析：

1. **影像学描述**：
   - X线显示右肱骨周围有多发不规则高密度影，呈团块状分布
   - CT显示这些高密度影有明确的边界，内部可见钙化/骨化成分和低密度区
   - MRI显示病变区域信号混杂，部分为高信号（可能代表软组织或囊变），部分为低信号（钙化/骨化）
2. **结合病史与影像**：
   - 30年长期存在：这排除了急性肿瘤或感染性病变的可能性
   - 近期疼痛：提示可能有继发变化，如炎症、退行性改变等
3. **诊断可能性排序**：
   1. 滑膜软骨瘤病(Synovial Chondromatosis)
   2. 多发性软骨瘤(Multiple Chondromas)
   3. 软骨肉瘤(Chondrosarcoma)
4. **临床建议**：
   - 进行进一步的影像学检查，如增强MRI以评估病变的血供和活性
   - 建议进行活检以明确病理诊断（特别是考虑到近期疼痛）
   - 与患者讨论治疗方案，包括观察、药物治疗或手术切除

现在我需要组织一个专业、全面的回答。

**影像学研判报告**

**影像学描述：**

1. X线片显示右肱骨周围软组织内多发不规则高密度影，呈团块状分布（大小不一），部分区域有分叶状结构。病变与肱骨关系密切但未直接侵入骨质。
2. CT扫描证实多发钙化/骨化灶，内部可见致密钙化成分及低密度区，提示可能包含软组织或囊性改变；病变边界相对清晰，无明显皮质破坏。
3. MRI显示病变区域信号混杂：T1WI呈等-稍低信号，T2WI呈高-中混合信号（部分区域有液-液平面），增强扫描见不均匀强化。病变中心可见多发点状低信号区（对应钙化/骨化灶）。

**诊断分析：** 结合30年长期病史与近期疼痛表现：

1. **最可能：滑膜软骨瘤病(Synovial Chondromatosis)**
   - 依据：① 多发、不规则钙化/骨化灶（符合"multiple round chondroid calcifications"）；② 长期存在（30年，与疾病慢性进展一致）；③ MRI显示部分区域呈骨髓信号（CT1描述"some of the rounded bodies to have marrow signal"）。
   - 临床特点：通常起源于关节内，可向周围软组织扩展（如CT1所示"extraarticular extension"），36岁女性符合高发年龄。
2. **次可能：多发性骨软骨瘤(Multiple Osteochondromas)**
   - 依据：① 多发钙化灶与骨骼关系密切；② 长期无症状病史（CT2描述"lytic lesion, often multilobulated and expansile"）。
   - 排除点：本例病变未见典型"骨性突起"，更符合软组织内游离体。
3. **需排除：低度恶性软骨肉瘤(Chondrosarcoma)**
   - 依据：① 近期疼痛提示可能有继发变化；② MRI显示不均匀强化及囊变（CT2描述"may develop more aggressive appearance"）。
   - 排除点：无明显皮质破坏、无骨膜反应，36岁年龄不符合典型发病高峰。

**临床建议：**

1. **紧急处理**：完善增强MRI+DWI序列，评估病变血供及恶性风险（重点观察是否出现"环状强化"或"弥漫性高信号"）。
2. **确诊措施**：
   - 优先行CT引导下穿刺活检（避开钙化区），病理送检S100、SOX9等免疫组化。
   - 若活检提示恶性，需立即行全身骨扫描+胸部CT排除转移。
3. **治疗方案**：
   - 滑膜软骨瘤病首选手术切除（包括关节镜下游离体摘除+滑膜部分切除）；
   - 术后1年内每3个月复查MRI监测复发。
4. **随访重点**：关注疼痛是否与活动相关，若出现夜间痛需警惕恶性转化。

注：本例符合"慢性病程+近期症状变化"的典型警示特征（CT2描述"may develop more aggressive appearance with less distinct border"），建议72小时内完成活检。
