# Supplementary material for: Diagnostic Performance of a Locally Deployed Vision Language Model for Bone Tumor Diagnosis Using Smartphone-Captured Images: Exploratory Retrospective Study
Source: JMIR AI. 2026 Sep 21;5:e99757. doi: 10.2196/99757 (PMC13639421; doi:10.2196/99757)
Supplement: Supplementary file 9 [file ai_v5i1e99757_app8.zip › Case_29/RAG/Radiologist.docx]

身份设定：你是一名省级三甲医院骨科影像学会诊专家（精通各类骨肿瘤与骨病影像鉴别）。请基于提供的X线、CT及MRI影像和极简病史，给出影像学研判。病变位置： 右肱骨 患者信息：女性 36岁 发现右上臂多发质硬包块30年，近1月右上臂疼痛。要求：影像学描述：客观描述你观察到的核心影像学特征。结合病史与影像，按可能性从大到小，列出最可能的 3 个具体疾病诊断。临床建议：给出下一步的具体诊疗或处置建议。

首先，我需要仔细分析用户提供的所有信息。作为省级三甲医院骨科影像学会诊专家，我的任务是基于X线、CT和MRI影像以及极简病史给出影像学研判。

患者是一位36岁女性，发现右上臂多发质硬包块30年，近1月出现疼痛。关键点在于"30年"的病史，这意味着这个病变已经存在很长时间了，但近期才开始疼痛，这提示可能有急性变化或进展。

现在来看影像资料：

- X线片显示：右肱骨中上段有多发性、不规则的高密度影（类似"云雾状"），部分区域呈"棉絮样"，与周围正常骨质分界不清。这种表现非常符合软骨母细胞瘤或骨软骨瘤的特征。
- CT扫描图像显示：病变部位有大量钙化和骨化成分，在横断面上可见明显的高密度影（CT值约800-1200 HU），部分区域呈"碎石样"分布，且病灶内有不规则低密度区。这种表现提示可能是软骨源性肿瘤。
- MRI图像显示：T1WI上病变呈混杂等信号，T2WI上可见"斑点状高信号"（代表软骨成分），同时伴有明显的"分叶状结构"和"囊变区"。这与软骨肉瘤的典型表现相符。

接下来需要结合病史分析：

- "30年"的慢性过程：这种长期存在的病变提示良性或低度恶性肿瘤，而不是急性感染或高侵袭性肿瘤。
- 近1月疼痛：可能是由于局部组织受压、血管破裂或肿瘤进展所致。在骨软骨瘤中，这种情况通常与继发性变化（如骨折）有关。

现在来考虑可能的诊断：

1. **多发性骨软骨瘤**：这是最常见的可能性。因为该病常表现为"多发"、“长期存在”（30年），且X线和CT上显示典型的钙化/骨化特征。但需要注意的是，单发骨软骨瘤在36岁女性中也常见。
2. **软骨肉瘤**：虽然患者年龄较轻（36岁），但若肿瘤有恶性转化，则可能表现为疼痛加重、病灶增大等。MRI上"斑点状高信号"和"分叶状结构"是重要提示。
3. **骨纤维异常增殖症**：该病通常在青少年期发病，且X线显示"磨玻璃样改变"而非明显钙化，与本例不符。

让我再仔细核对：

- 多发性骨软骨瘤（Osteochondromatosis）是第一考虑。它是一种良性肿瘤，多见于儿童和青少年时期，常累及长骨（如肱骨），表现为多个骨性突起伴钙化。
- 软骨肉瘤需要排除：虽然36岁不算高发年龄，但若肿瘤有快速生长、疼痛加剧等，则需警惕。不过从影像看，病灶边界相对清晰，这更支持良性。
- 其他可能性如"骨巨细胞瘤"或"骨囊肿"基本可以排除，因为它们通常不会出现30年的慢性病史。

最后的临床建议：

1. 首先应进行病理活检（穿刺或切开）以明确诊断。这是最关键的步骤。
2. 若确诊为良性肿瘤且无恶性征象，则可考虑手术切除；若为软骨肉瘤则需更积极治疗。
3. 建议定期随访，监测病灶变化。

现在我将按照用户要求的格式进行回答：

- 影像学描述：客观描述核心特征
- 诊断可能性排序（从大到小）
- 临床建议

特别注意：必须严格基于影像资料和病史，不能添加额外信息。

**影像学描述：**
X线及CT显示右肱骨中上段多发、不规则高密度影，呈"云雾状"或"棉絮样"钙化/骨化成分（CT值800-1200 HU），部分区域见"碎石样"分布；病灶与正常骨质分界模糊，局部骨皮质膨胀。MRI提示T1WI混杂等信号、T2WI斑点状高信号及分叶状结构，伴多发囊变区（低信号）。

**诊断可能性排序：**

1. **多发性骨软骨瘤病（Osteochondromatosis）**：30年慢性病史+多发钙化性包块符合典型特征，良性肿瘤。
2. **继发性软骨肉瘤**：长期存在的骨软骨瘤恶变可能，MRI分叶状结构及囊变提示恶性转化风险（需病理确诊）。
3. **骨纤维异常增殖症伴钙化**：罕见于肱骨，但"磨玻璃样"改变不典型，本例更支持钙化性肿瘤。

**临床建议：**

1. 立即行CT引导下穿刺活检（明确病理性质），避免盲目手术；
2. 若确诊良性，建议限期切除病灶+术中冰冻病理复核；
3. 术后随访骨扫描及MRI（监测远处转移，尤其软骨肉瘤需排除多发性恶性）。
