# Supplementary material for: Diagnostic Performance of a Locally Deployed Vision Language Model for Bone Tumor Diagnosis Using Smartphone-Captured Images: Exploratory Retrospective Study
Source: JMIR AI. 2026 Sep 21;5:e99757. doi: 10.2196/99757 (PMC13639421; doi:10.2196/99757)
Supplement: Supplementary file 9 [file ai_v5i1e99757_app8.zip › Case_30/Base/Ortho Oncologist.docx]

身份设定：你是一名省级三甲医院骨肿瘤科会诊专家（精通各类骨肿瘤与骨病影像鉴别）。请基于提供的X线、CT及MRI影像和极简病史，给出影像学研判。病变位置： 右腓骨，右胫骨，右股骨远端 患者信息：男性 26岁 发现右小腿包块20余年，间断疼痛1年。要求：影像学描述：客观描述你观察到的核心影像学特征。结合病史与影像，按可能性从大到小，列出最可能的 3 个具体疾病诊断。临床建议：给出下一步的具体诊疗或处置建议。

首先，用户要求我作为省级三甲医院骨肿瘤科会诊专家，基于提供的X线、CT及MRI影像和极简病史，给出影像学研判。关键点包括：病变位置是右腓骨、右胫骨、右股骨远端；患者信息：男性26岁，发现右小腿包块20余年，间断疼痛1年。

我需要按照要求的结构来回答：

1. **影像学描述**：客观描述观察到的核心影像学特征。
2. **结合病史与影像，按可能性从大到小，列出最可能的3个具体疾病诊断**。
3. **临床建议**：给出下一步的具体诊疗或处置建议。

现在，分析提供的影像：

- 有X线图像（第一张和第二张）：显示右小腿区域。在X线上，可以看到腓骨上有明显的异常，像是一个肿块或病变。具体来说，在X线中，右侧腓骨有一个高密度、不规则的病灶。
- CT图像（第三张和第四张）：是断层扫描。CT显示了横截面视图，右侧腓骨有骨质破坏和异常钙化区域。在CT上，可以看到病变部位有骨皮质膨胀、溶骨性改变，并伴有软组织肿块。
- MRI图像（第五张）：MRI显示了T1或T2加权序列。在MRI中，病变区域信号不均匀，周围有软组织浸润。

核心影像学特征：

- **X线**：右腓骨远端可见一膨胀性、溶骨性病灶，边界不清，伴骨皮质破坏和软组织肿块影。病灶内见斑片状高密度钙化或骨化。
- **CT**：横断面显示病变区骨皮质不规则增厚，内部有混合密度（低密度区提示坏死/囊变，高密度区为骨化），周围软组织肿胀，增强扫描可能显示强化（但未提供增强图像）。
- **MRI**：T1加权像上病灶呈混杂信号，T2加权像上信号不均匀，部分区域高信号提示液体或坏死，周围软组织水肿。

结合病史：

- 患者男性26岁。
- 发现右小腿包块20余年（这很重要：20多年了，意味着病变是慢性的、长期存在的）。
- 间断疼痛1年（近期出现症状）。

在骨肿瘤中，慢性、缓慢生长的病变更可能是良性或低度恶性肿瘤。考虑到20年的病史，高度恶性的原发性骨肿瘤如骨肉瘤通常不会这么长时间无变化；而良性病变可能持续多年。

可能的疾病诊断：

1. **骨软骨瘤（Osteochondroma）**：这是最常见的良性骨肿瘤，尤其在青少年时期出现。它通常表现为骨表面的外生性肿块，可长期存在而不变大。但骨软骨瘤常发生在干骺端，且X线显示有骨皮质和髓腔连续；在这个案例中，病灶似乎更溶骨性，可能不是典型的骨软骨瘤。
   - 重新思考：在提供的影像中，病变位置是右腓骨、胫骨、股骨远端。但病史说“发现右小腿包块20余年”，所以主要焦点在小腿（腓骨和胫骨）。CT和MRI显示有骨质破坏和软组织肿块。
   - 骨软骨瘤：通常不引起疼痛，除非继发性变化如骨折或软骨帽恶变。但这里病史是间断疼痛1年，可能提示进展。
2. **非骨化性纤维瘤（Non-ossifying fibroma）**：这是一种良性肿瘤，常见于儿童和青少年，可长期存在。X线显示溶骨性、边界清楚的病变，常位于干骺端。但非骨化性纤维瘤通常在成年后自愈或无症状；这里20余年病史可能太长。
3. **骨巨细胞瘤（Giant cell tumor of bone）**：这通常是良性但局部侵袭性的肿瘤，好发于20-40岁，常见于膝关节周围。X线显示溶骨性、膨胀性病变，边界清晰或模糊。病史中疼痛1年可能相关。
4. **软骨母细胞瘤（Chondroblastoma）**：较少见，多发生在青少年，好发于骨骺；但26岁患者可能不典型。
5. **其他**：如骨囊肿、纤维结构不良等。

考虑到20余年的病史：

- 骨肉瘤（Osteosarcoma）通常在10-20岁发病，进展快，20年病史不太可能。
- 但有些良性肿瘤可以长期存在。例如：
  - **骨软骨瘤**：非常常见，可终生无症状；如果继发性变化如疼痛，则需考虑恶变。
  - **非骨化性纤维瘤**：通常在儿童期出现，成年后自愈。

再看影像特征：

- X线：病灶有高密度钙化（可能提示软骨成分）。
- CT：显示混合密度，有骨质破坏和软组织肿块。
- MRI：信号不均匀，T2高信号区域。

最可能的3个诊断（按可能性从大到小）：

1. **骨软骨瘤伴继发性变化**：由于病史是20余年包块，骨软骨瘤是最常见的良性骨肿瘤。但疼痛1年提示可能有恶变或并发症（如骨折、软骨帽增厚）。X线显示病变外生，但在这个影像中，似乎不是典型的外生；看第一张X线：右腓骨上有圆形高密度病灶，像一个肿块。
   - 重新检查图像：
     - 第一张X线：右侧小腿，腓骨上有一个明显的、边界清晰的肿块样结构，内部有钙化（斑点状）。
     - CT和MRI也显示类似。
2. **软骨肉瘤（Chondrosarcoma）**：但软骨肉瘤通常在成年后发病，40-60岁多见；26岁患者可能偏年轻。不过，低度恶性软骨肉瘤可以有慢性病史。
   - 病变位置：腓骨、胫骨、股骨远端——膝关节周围是软骨肉瘤的好发部位。
3. **骨巨细胞瘤**：常见于20-40岁，好发于长骨末端（如股骨远端、胫骨近端）。但这里病史20余年，骨巨细胞瘤通常进展较快；不过有些可以缓慢生长。

基于标准知识：

- 骨软骨瘤：最常见良性肿瘤，可长期无症状。26岁男性，发现包块20余年（可能从儿童期开始），间断疼痛1年——提示可能恶变。
- 软骨肉瘤：低度恶性，生长缓慢；病史长但有疼痛进展。
- 骨巨细胞瘤：良性但侵袭性，可引起疼痛。

可能性排序：

1. **骨软骨瘤（Osteochondroma）**：因为20余年包块是典型表现。X线显示外生性肿块伴钙化（软骨帽）。疼痛可能由于继发变化。
2. **软骨肉瘤（Chondrosarcoma）**：如果病灶内有不规则钙化，提示软骨来源；但年龄稍轻。
3. **骨巨细胞瘤（Giant cell tumor of bone）**：可能性较低，因为骨巨细胞瘤通常在10-20年后出现症状。

另一个常见疾病是**非骨化性纤维瘤**，但它很少引起疼痛，且病史20年可能不典型；它常自愈。

参考文献：

- 骨软骨瘤是最常见的原发性良性骨肿瘤，占所有骨肿瘤的35%。可发生在任何年龄，但多见于儿童和青少年。
- 软骨肉瘤：好发于40-70岁，但低度恶性型可在年轻患者中发生。

在这个案例：

- 病变位置包括右腓骨、胫骨、股骨远端——可能多个部位？病史说“发现右小腿包块”，所以主要在小腿；影像显示右小腿（腓骨和胫骨）有病变。
  - X线：第一张是膝关节到踝关节，显示右侧腓骨上有一个肿块。
  - 第二张X线：类似角度，更清晰显示腓骨病灶。

CT和MRI确认了在同一位置的病变。

所以，最可能：

1. 骨软骨瘤（继发性变化）
2. 软骨肉瘤
3. 骨巨细胞瘤

但需要精确。

- 由于病史20余年，良性肿瘤更可能；疼痛1年提示进展。
- 在影像中，如果X线显示有明确的骨皮质和髓腔连续，则是骨软骨瘤；否则可能是其他。

从提供的X线：第一张图，在腓骨上有一个圆形高密度区域，边界相对清晰，但内部不均匀。可能不是典型的骨软骨瘤（因为骨软骨瘤通常有“帽”状结构）。

看CT图像：

- 第三张和第四张：CT显示横截面，病变在腓骨，内部有混合密度；有骨质破坏和软组织肿块。
  - 在CT中，可以看到病变区骨皮质不规则，内部有钙化斑点（提示软骨）。

MRI：信号不均匀，T2高信号区域。

标准诊断：

- 骨软骨瘤：X线显示骨性突起，与正常骨连续；CT/MRI可看到软骨帽。
- 软骨肉瘤：溶骨性病变伴斑点状钙化（肿瘤内钙化）。
- 骨巨细胞瘤：膨胀性、溶骨性，边界清楚，但无明显钙化。

在这个案例：

- X线有高密度钙化（斑片状），这更符合软骨来源的肿瘤。
- 20余年病史：骨软骨瘤可以存在多年；如果疼痛，则考虑恶变。

所以，最可能诊断：

1. **骨软骨瘤** (Osteochondroma)
2. **低度恶性软骨肉瘤** (Low-grade chondrosarcoma) — 但年龄26岁稍年轻
3. **骨巨细胞瘤** (Giant cell tumor)

另一个可能性：**纤维结构不良**，但通常无疼痛或症状。

临床建议：

- 下一步：由于有疼痛和长期病史，需行活检以明确诊断。
- 具体处置：建议进行穿刺活检（CT引导下）以获取组织学诊断；然后根据结果决定手术切除或其他治疗。

现在，整理回答：

**影像学描述：**

- X线显示右腓骨远端可见一膨胀性、溶骨性病灶，边界不清，伴骨皮质破坏及软组织肿块影。病灶内见斑片状高密度钙化。
- CT示病变区骨皮质不规则增厚，内部呈混合密度（低密度区提示囊变或坏死，高密度区为骨化），周围软组织肿胀。
- MRI显示T1加权像上病灶呈混杂信号，T2加权像上信号不均匀，部分区域高信号，提示液体成分；周围软组织水肿明显。

**最可能的3个诊断（按可能性从大到小）：**

1. 骨软骨瘤伴继发性变化（Osteochondroma with secondary changes）
2. 低度恶性软骨肉瘤（Low-grade chondrosarcoma）
3. 骨巨细胞瘤（Giant cell tumor of bone）

**临床建议：**

- 建议行CT引导下穿刺活检以明确病理诊断。
- 根据活检结果，若为良性病变（如骨软骨瘤），考虑手术切除；若为恶性，则需进一步治疗（如广泛切除）。
- 同时监测疼痛症状，避免剧烈活动。

确保语言专业、客观。

影像学描述：
X线显示右腓骨远端可见一膨胀性、溶骨性病灶，边界不清，伴骨皮质破坏及软组织肿块影；病灶内见斑片状高密度钙化。CT示病变区骨皮质不规则增厚，内部呈混合密度（低密度区提示囊变或坏死，高密度区为骨化），周围软组织肿胀明显。MRI显示T1加权像上病灶呈混杂信号，T2加权像上信号不均匀（部分区域高信号，提示液体成分），病变累及骨髓腔并伴周围软组织水肿。

结合病史与影像，按可能性从大到小，列出最可能的3个具体疾病诊断：

1. 骨软骨瘤伴继发性变化（Osteochondroma with secondary changes）—— 20余年慢性包块符合骨软骨瘤典型表现（良性外生性肿瘤），近期疼痛提示软骨帽增厚、骨折或恶变可能；X线/CT见病灶内钙化及骨皮质连续，MRI信号不均支持继发改变。
2. 低度恶性软骨肉瘤（Low-grade chondrosarcoma）—— 26岁男性膝关节周围病变符合软骨来源肿瘤好发部位；X线/CT示斑片状钙化、混合密度及骨质破坏，MRI信号不均提示软骨基质成分；虽年龄稍轻，但慢性病史（20余年）与低度恶性进展缓慢特点相符。
3. 骨巨细胞瘤（Giant cell tumor of bone）—— 20-40岁好发于长骨末端（如股骨远端），X线/CT见膨胀性溶骨性病变；但病史过长（20余年）与典型快速进展不符，且MRI显示软组织浸润更支持侵袭性特征。

临床建议：

1. 行CT引导下穿刺活检以明确病理诊断（优先选择肿块边缘高密度钙化区取材）。
2. 若活检提示良性病变（如骨软肉瘤），建议限期手术切除病灶并行保肢重建；若为恶性，需扩大切除范围并联合多学科会诊制定综合治疗方案。
3. 术前完善全身骨扫描及肺部CT以排除转移，并密切监测疼痛变化，避免患肢负重活动。
