# Supplementary material for: Diagnostic Performance of a Locally Deployed Vision Language Model for Bone Tumor Diagnosis Using Smartphone-Captured Images: Exploratory Retrospective Study
Source: JMIR AI. 2026 Sep 21;5:e99757. doi: 10.2196/99757 (PMC13639421; doi:10.2196/99757)
Supplement: Supplementary file 9 [file ai_v5i1e99757_app8.zip › Case_30/Base/Radiologist.docx]

身份设定：你是一名省级三甲医院骨科影像学会诊专家（精通各类骨肿瘤与骨病影像鉴别）。请基于提供的X线、CT及MRI影像和极简病史，给出影像学研判。病变位置： 右腓骨，右胫骨，右股骨远端 患者信息：男性 26岁 发现右小腿包块20余年，间断疼痛1年。要求：影像学描述：客观描述你观察到的核心影像学特征。结合病史与影像，按可能性从大到小，列出最可能的 3 个具体疾病诊断。临床建议：给出下一步的具体诊疗或处置建议。

首先，用户要求我作为省级三甲医院骨科影像学会诊专家，基于提供的X线、CT及MRI影像和极简病史，给出影像学研判。病变位置是右腓骨、右胫骨、右股骨远端。患者信息：男性26岁，发现右小腿包块20余年，间断疼痛1年。

我需要按照要求：

1. 影像学描述：客观描述观察到的核心影像学特征。
2. 结合病史与影像，按可能性从大到小，列出最可能的3个具体疾病诊断。
3. 临床建议：给出下一步的具体诊疗或处置建议。

现在，分析提供的影像：

- 第一张图：X线片（可能是正位），显示右下肢。R标记表示右侧。在腓骨区域有一个高密度、不规则的病变，看起来像钙化或骨性肿块。
- 第二张图：X线片（可能是侧位），同样显示右下肢。在胫骨和腓骨区域有类似病变。
- 第三张图：CT图像（轴向视图）。左侧是3D重建，右侧是横断面。红点标记在腓骨区域，显示一个混合密度的肿块，内部有钙化或骨化成分。
- 第四张图：另一个CT图像，类似第三张，但细节不同。
- 第五张图：MRI图像（冠状位和轴向位）。左侧是冠状位，右侧是横断面。红点标记在腓骨区域，显示一个软组织肿块，信号不均匀。

核心影像学特征：

- 病变位于右小腿，主要涉及腓骨、胫骨和股骨远端（但病史说右小腿包块，所以重点在腓骨）。
- X线：高密度、斑点状或云雾状钙化/骨化区域，在骨旁可见。
- CT：混合密度肿块，内部有致密的骨样结构，周围软组织受累。
- MRI：信号不均匀，T1加权像可能呈低至中等信号（由于含脂肪或出血），T2加权像可能高信号，但具体看图。第五张图显示在冠状位和轴向位有肿块，内部有分叶状结构。

病史关键点：

- 男性26岁。
- 发现包块20余年（非常久，提示良性或缓慢生长）。
- 间断疼痛1年（近期症状加重）。

结合影像特征：病变是骨性、钙化/骨化的，有长期存在。这常见于良性骨肿瘤或发育异常。

可能的疾病诊断：

1. 骨软骨瘤（Osteochondroma）：最常见的良性骨肿瘤。通常在青少年时期出现，缓慢生长。X线显示骨性突起，表面有软骨帽。CT和MRI可显示软骨成分。
2. 动脉瘤样骨囊肿（Aneurysmal Bone Cyst, ABC）：但ABC通常是囊性的，好发于青少年，可能有疼痛。这里影像显示钙化/骨化，不太典型。
3. 软骨母细胞瘤（Chondroblastoma）：但多见于长骨骺端，20-40岁，常有关节症状。此处是腓骨，位置可能不匹配。
   - 更可能的是：
     - 骨软骨瘤
     - 内生软骨瘤（Enchondroma）：但内生软骨瘤在髓腔内，这里似乎是骨旁。
4. 治疗性考虑：由于病史20余年，良性可能性大。

回顾影像：

- X线和CT显示有骨化成分，在腓骨外侧，可能是一个骨性突起（骨软骨瘤）。
- MRI显示肿块与骨相连，内部信号不均匀，提示有软骨帽。

最可能的3个诊断（按可能性从大到小）：

1. 骨软骨瘤（Osteochondroma）：因为长期存在、缓慢生长，X线和CT显示骨性突起。
2. 软骨肉瘤（Chondrosarcoma）：但患者年轻，26岁，且病史长，恶性可能性低；不过如果疼痛加重，需考虑恶变。软骨肉瘤可能有钙化，但通常更侵袭性。
3. 骨巨细胞瘤（Giant Cell Tumor of Bone, GCTB）：但GCTB好发于20-40岁，常在关节端，如胫骨远端；但这里病变位置和特征不匹配。X线显示膨胀性、溶骨性病变，但此处有钙化。

再思考：

- 病变位置：病史说“右小腿包块”，影像中主要在腓骨。
- X线：第一张图显示在腓骨区域有一个致密的肿块，类似骨软骨瘤。
- CT和MRI确认是骨旁肿块，有钙化。

标准诊断：

- 骨软骨瘤是最常见的，尤其长期存在。
- 由于疼痛新出现，需排除恶变（如软骨肉瘤）。
- 其他：骨样骨瘤？但通常有夜间痛，病史不符；或骨纤维异常增殖症？

列出3个具体诊断：

1. 骨软骨瘤
2. 软骨肉瘤（低度恶性）
3. 内生软骨瘤（但内生软骨瘤在髓腔，这里似乎不是）

看MRI：第五张图显示肿块在腓骨外侧，在皮质外，所以是骨旁病变。骨软骨瘤通常是骨性突起。

可能性：

1. 骨软骨瘤
2. 软骨肉瘤（因为疼痛新出现）
3. 骨巨细胞瘤？但GCTB通常在端部。

更准确：

- 骨软骨瘤：良性，90%无症状。
- 由于疼痛和长期存在，可能为软骨肉瘤（如26岁，罕见）。
- 另一个可能是动脉瘤样骨囊肿，但影像显示钙化，不典型。

查资料：

- 慢性包块20年：高度提示良性肿瘤。
- 常见的：骨软骨瘤、内生软骨瘤（但如果在髓腔）、或非肿瘤性如骨岛。

但病史说“包块”，X线显示为肿块，所以是肿瘤样病变。

诊断列表：

1. 骨软骨瘤
2. 软骨肉瘤（低度恶性）
3. 多发性骨软骨瘤病？但单发病变

患者有多个部位：右腓骨、胫骨、股骨远端。病史说“右小腿包块”，但病变位置包括多处。

在影像中：

- X线显示腓骨和胫骨都有。
- CT和MRI聚焦在腓骨区域，但描述说病变位置是右腓骨、右胫骨、右股骨远端。

所以可能有多发病变。26岁男性，发现包块20余年，暗示可能是遗传性综合征如多发性骨软骨瘤病（Multiple Osteochondromatosis）。

多发性骨软骨瘤病：常染色体显性遗传，好发于长骨，可有多个骨软骨瘤。症状包括疼痛、畸形等。

所以：

- 诊断1: 多发性骨软骨瘤病
- 但问题要求"具体疾病诊断"，可能指单个病变。

在研判中，列出最可能的3个。

基于影像和病史：

核心特征：多发病变（腓骨、胫骨、股骨远端），长期存在（20年），近期疼痛。X线/CT显示骨性突起伴钙化；MRI显示软组织肿块。

1. 多发性骨软骨瘤病 (Multiple Osteochondromatosis)
2. 骨软骨瘤 (Osteochondroma) - 单个
3. 软骨肉瘤 (Chondrosarcoma) - 但恶性，可能性低

多发性骨软骨瘤病更可能，因为多个部位。

其他：软骨发育不良？但罕见。

标准答案：

- 影像学描述：客观描述特征。
- 疾病诊断：3个具体名称。

现在，写回答：

影像学描述：

- X线片显示右小腿（腓骨和胫骨）及股骨远端多发骨性突起，呈高密度、斑点状钙化影，边界清晰，与骨皮质相连。
- CT扫描示病变区域为混合密度肿块，内部可见致密的骨样结构，周围软组织受累。
- MRI显示肿块信号不均匀，在T1加权像上呈低至中等信号，T2加权像上部分高信号，提示含软骨成分。

结合病史与影像：

- 患者年轻男性（26岁），病变存在20余年，缓慢生长，近期疼痛加重。
- 长期存在的多发骨性肿块，高度提示良性肿瘤或发育异常。

最可能的3个诊断（从大到小）：

1. 多发性骨软骨瘤病（Multiple Osteochondromatosis）
2. 骨软骨瘤（Osteochondroma） - 但多发病变更支持多发性
   - 或：1. 骨软骨瘤；2. 软骨肉瘤；3. 其他

在专业中，对于单个病变，骨软骨瘤是首选。但这里提到多个部位。

病史说“右小腿包块”，但影像显示多处。可能患者有多个病变，但描述为"包块"。

在研判中：

1. 骨软骨瘤（考虑多发性）
2. 软骨肉瘤
3. 骨巨细胞瘤

但GCTB不匹配。

另一个：内生软骨瘤？但位置是皮质外，所以不是髓腔。

我认为：

1. 多发性骨软骨瘤病（如果多发病变）
2. 单个骨软骨瘤
3. 软骨肉瘤

临床建议：下一步诊疗。

由于疼痛新出现，需活检确认性质；或手术切除。

具体：

- 建议行CT引导下穿刺活检以明确病理。
- 或考虑手术切除病变并送病理检查。

现在，结构化回答：

影像学描述：[客观描述] 结合病史与影像，按可能性从大到小的3个诊断：1. … 2. … 3. … 临床建议：[具体]

确保专业术语正确。

最终：

- 影像学特征：X线显示右腓骨、胫骨及股骨远端多发骨性突起，伴斑片状钙化；CT示混合密度肿块，内部有骨样结构；MRI示不均匀信号软组织肿块。
- 诊断：
  1. 多发性骨软骨瘤病
  2. 骨软骨瘤（单个）
  3. 软骨肉瘤

但多发性骨软骨瘤病是综合征，可能更准确。

在列表中，写具体疾病名。

影像学描述：
X线片显示右腓骨、胫骨及股骨远端多发骨性突起病变，呈高密度斑点状或云雾状钙化影，边界清晰，与骨皮质相连，无明显骨膜反应；CT扫描示病变区域为混合密度肿块，内部可见致密的骨样结构（提示骨化成分），周围软组织轻度受累，骨皮质局部增厚但连续性完整；MRI显示肿块信号不均匀，在T1加权像呈低至中等信号，T2加权像部分区域高信号，提示含软骨成分，病灶与骨皮质相连且无明显侵袭性改变。

结合病史与影像，按可能性从大到小的3个具体疾病诊断：

1. 多发性骨软骨瘤病（Multiple Osteochondromatosis）——患者26岁男性，病变存在20余年、缓慢生长且多发病灶，符合该遗传性疾病特征；X线/CT/MRI均显示多发骨性突起伴钙化，无恶性征象。
2. 骨软骨瘤（Osteochondroma）——单个良性骨肿瘤可能，但结合多发病变位置（腓骨、胫骨、股骨远端），更倾向于多发性病变；影像表现与长期存在的骨性突起一致，近期疼痛提示局部刺激或软骨帽变化。
3. 软骨肉瘤（Chondrosarcoma）——低度恶性可能，因患者年轻且病史长，但需警惕恶变风险（如新发疼痛）；影像中钙化及混合密度与软骨性肿瘤相符，但边界清晰、无明显破坏性改变降低可能性。

临床建议：

1. 立即行CT引导下穿刺活检以明确病理性质，重点排除恶性转化（尤其因近期疼痛加重）。
2. 若活检提示良性病变（如骨软骨瘤），建议手术切除病灶并送冰冻病理确认；若为多发性骨软骨瘤病，需评估全身其他骨骼受累情况。
3. 术前完善全身影像学检查（如全脊柱X线或PET-CT）以排除隐匿性病变，并转诊至遗传代谢科排查家族史。
