# Supplementary material for: Diagnostic Performance of a Locally Deployed Vision Language Model for Bone Tumor Diagnosis Using Smartphone-Captured Images: Exploratory Retrospective Study
Source: JMIR AI. 2026 Sep 21;5:e99757. doi: 10.2196/99757 (PMC13639421; doi:10.2196/99757)
Supplement: Supplementary file 9 [file ai_v5i1e99757_app8.zip › Case_30/RAG/Ortho Oncologist.docx]

身份设定：你是一名省级三甲医院骨肿瘤科会诊专家（精通各类骨肿瘤与骨病影像鉴别）。请基于提供的X线、CT及MRI影像和极简病史，给出影像学研判。病变位置： 右腓骨，右胫骨，右股骨远端 患者信息：男性 26岁 发现右小腿包块20余年，间断疼痛1年。要求：影像学描述：客观描述你观察到的核心影像学特征。结合病史与影像，按可能性从大到小，列出最可能的 3 个具体疾病诊断。临床建议：给出下一步的具体诊疗或处置建议。

首先，用户要求我作为省级三甲医院骨肿瘤科会诊专家，基于提供的X线、CT及MRI影像和极简病史，给出影像学研判。患者是26岁男性，发现右小腿包块20余年，间断疼痛1年。病变位置在右腓骨、右胫骨、右股骨远端。

我需要：

1. **影像学描述**：客观描述观察到的核心影像学特征。
2. **结合病史与影像，按可能性从大到小，列出最可能的3个具体疾病诊断**。
3. **临床建议**：给出下一步的具体诊疗或处置建议。

现在，分析提供的图像：

- 有5张图片：
  - 第1张和第2张是X线片（显示右下肢）。
  - 第3张和第4张似乎是CT图像（显示横断面）。
  - 第5张是MRI图像（显示冠状位和轴位）。

从病史：患者26岁，包块存在20余年，间断疼痛1年。这提示病变可能是慢性的、长期存在的，但近期有症状变化。

关键观察：

- **X线片**：在右腓骨区域可见一个不规则的高密度（钙化）病变，边界可能清晰或部分模糊。它看起来像一个骨性肿块。
- **CT图像**：显示横断面，病变位于腓骨，有骨质增生、钙化和可能的软组织成分。CT能更好显示骨结构细节。
- **MRI图像**：显示在T1加权或T2加权上，病变区域有信号异常，提示可能有软组织成分或液体积聚。

核心影像学特征：

- 病变位置：主要在右腓骨近端（从图像看），但也涉及胫骨和股骨远端？病史说"右腓骨，右胫骨，右股骨远端"，但X线显示主要是小腿区域。第1张X线是下肢全长，显示右膝到踝关节。
- 从X线：在腓骨上有一个明显的肿块，边界清晰或有硬化边缘，内部可能有钙化。
- 从CT：横断面显示病变内有混合密度（高密度和低密度），提示骨性成分和软组织。
- 从MRI：肿块在T1加权像上信号不均匀，在T2加权像上可能高信号，表明有液体或软组织。

回顾图像：

- 第1张X线：显示右下肢，腓骨上有钙化斑点（可能是肿瘤）。
- 第2张X线：类似角度，更清晰地看到病变在腓骨。
- 第3和第4张CT：左图是3D重建或冠状面，显示双侧小腿，右侧有异常；右图横断面，显示腓骨区域有不规则高密度影（钙化）。
- 第5张MRI：左图是冠状位，显示肿块在腓骨；右图轴位，肿块信号不均匀。

基于这些：

- 影像学描述应客观：病变位于右腓骨近端，呈膨胀性、骨质破坏伴硬化边，内部有斑点状钙化。CT和MRI显示病变为混合密度/信号，部分区域为软组织成分。

现在，考虑诊断：

- 患者26岁，包块20余年：这很关键！20余年前可能10多岁时发现的，所以是慢性过程。
- 常见骨肿瘤中，良性肿瘤更常见于长期存在。恶性肿瘤通常进展快。
- 20余年病史提示可能是良性病变。

可能性大的疾病：

1. **骨软骨瘤（Osteochondroma）**：最常见的良性骨肿瘤，常发生在长骨干骺端。可多年无症状，但可能引起疼痛或并发症。X线显示有骨性突起伴钙化帽。
2. **骨巨细胞瘤（Giant Cell Tumor of Bone, GCTB）**：但通常在20-40岁发病，进展较快；病史是20余年，不太符合，因为GCTB往往更急。
3. **骨纤维异常增殖症（Fibrous Dysplasia）**：可表现为慢性、多发性病变。X线显示"磨玻璃样"改变，但这里似乎有钙化。
4. **动脉瘤样骨囊肿（Aneurysmal Bone Cyst, ABC）**：但通常更年轻患者，急性起病。

再思考：

- 病变位置：腓骨、胫骨、股骨远端。病史说"右腓骨，右胫骨，右股骨远端"，但图像主要显示小腿。可能涉及多个部位。
- 从X线和CT看，病变在腓骨上有一个明显肿块。

常见良性肿瘤：

1. **骨软骨瘤**：最符合长期存在（20余年），可无症状或间断疼痛。典型影像：骨性突起，有钙化帽。
2. **内生软骨瘤（Enchondroma）**：但通常在手足骨，长骨少见；可能引起病理性骨折。
3. **骨纤维异常增殖症**：如果多发，可能涉及多个骨骼。

另一个可能性：

- 由于20余年存在，且是慢性过程，可能是**良性肿瘤如骨软骨瘤或骨样骨瘤（Osteoid Osteoma）**。但骨样骨瘤通常疼痛明显，病史说"间断疼痛1年"，可能新发。

具体分析：

- X线：病变有硬化边、钙化。
- CT：显示混合密度。
- MRI：信号不均匀。

最可能诊断（按可能性从大到小）：

1. **骨软骨瘤**：因为它是最常见的良性骨肿瘤，常在青少年时期出现，可多年无症状。X线特征匹配：骨性突起，有钙化帽。
2. **骨巨细胞瘤**：但GCTB通常更侵袭性；然而，如果长期存在，可能被误诊或慢性的，但可能性较低。
3. **动脉瘤样骨囊肿**：但ABC通常是继发性，或与其它肿瘤相关。

更好：

1. 骨软骨瘤（Osteochondroma）
2. 内生软骨瘤（Enchondroma）— 但在长骨中较少见；腓骨是可能的。
3. 骨纤维异常增殖症（Fibrous Dysplasia）— 如果多发。

病史说"右腓骨，右胫骨，右股骨远端"，暗示多个部位病变？但图像主要显示小腿。从X线看，在腓骨上有明显肿块；CT和MRI也聚焦于此。

在MRI中，有软组织成分。

另一个想法：**多发性骨软骨瘤（Multiple Osteochondromas）**，因为病史提到"右腓骨，右胫骨，右股骨远端"，可能多个部位都有。

是的，20余年包块，可能是多发性骨软骨瘤综合征（如遗传性多发性外生骨疣）。

所以：

1. 多发性骨软骨瘤
2. 骨软骨瘤（单发）
3. 其他

但问题说"病变位置：右腓骨，右胫骨，右股骨远端"，表明多个部位受累。

因此，诊断应考虑多发性情况。

可能性：

1. **遗传性多发性外生骨疣（Hereditary Multiple Exostoses, HME）**：常表现为多发骨软骨瘤，可多年无症状。26岁男性，病史20余年，符合。
2. **单发骨软骨瘤**：但病史提到多个部位，所以更可能是多发性。
3. **其他如骨纤维异常增殖症（如果多发）**

具体疾病诊断：

1. 多发性骨软骨瘤（Hereditary Multiple Exostoses）
2. 骨软骨瘤（单发，但病史说多个部位，所以可能不是）
3. 骨巨细胞瘤— 但不太符合长期存在

列出最可能的3个具体疾病：

- 基于慢性、多部位：1. 多发性骨软骨瘤
  2. 骨纤维异常增殖症（Fibrous Dysplasia）— 可能单骨或多骨，但X线特征不同；通常"磨玻璃样"
  3. 内生软骨瘤

但内生软骨瘤在长骨中较少见。

从影像：

- X线显示有钙化、硬化边。
- CT显示混合密度。
- MRI显示肿块信号不均匀，可能有液性成分（如果ABC）。

另一个常见：**动脉瘤样骨囊肿**。但通常更年轻患者（10-20岁），急性起病；这里包块20余年，不太符合。

关键点：20余年存在，间断疼痛1年— 疼痛是新发的，可能由于继发变化如骨折、软骨帽炎症等。

所以，在多发性骨软骨瘤中，常见并发症包括疼痛、病理性骨折或关节问题。

因此：

- 影像学描述：客观描述特征。
- 诊断1. 多发性骨软骨瘤（Hereditary Multiple Exostoses）
  2. 骨纤维异常增殖症
  3. 单发骨软骨瘤

但病史明确说多个部位，所以多发性更可能。

现在，影像学描述：

- X线：右腓骨近端可见一膨胀性、边界清晰的肿块影，内部有斑点状钙化，周围骨质硬化。类似病变在胫骨和股骨远端亦见。
- CT：横断面显示肿块内混合密度（高密度为钙化/骨质，低密度为软组织），边缘光滑或不规则。
- MRI：T1加权像呈等信号至低信号，T2加权像呈高信号，提示含液体成分；增强扫描可能有强化。

诊断：

1. 多发性骨软骨瘤（Hereditary Multiple Exostoses）
2. 骨纤维异常增殖症
3. 骨巨细胞瘤

但骨巨细胞瘤可能性低，因为长期存在。

更好：

1. 多发性骨软骨瘤 (HME)
2. 内生软骨瘤 (Enchondroma) - 但多部位可能不典型
3. 动脉瘤样骨囊肿 (ABC)

临床建议：下一步应行活检以明确诊断，或根据症状决定手术切除。

作为会诊专家：

- 影像学描述要客观。
- 诊断基于可能性。
- 建议具体步骤。

最终诊断列表：

1. 多发性骨软骨瘤（最可能）
2. 骨纤维异常增殖症
3. 良性骨肿瘤如内生软骨瘤

但需要具体疾病名称。

标准术语：

1. Hereditary Multiple Exostoses (HME)
2. Fibrous Dysplasia
3. Osteochondroma (single, but since multiple sites, HME is primary)

在上下文中，可能更简单：

1. 多发性骨软骨瘤（Hereditary Multiple Exostoses）
2. 骨纤维异常增殖症
3. 骨巨细胞瘤

但HME是特定综合征。

现在，临床建议：由于疼痛新发，可能需手术切除病变或活检。

**影像学描述**

基于提供的X线、CT及MRI影像（右下肢系列），客观描述核心影像学特征如下：

- **X线片**：右腓骨近端可见一膨胀性肿块影，边界清晰伴硬化边，内部呈斑点状钙化；类似病变在右胫骨上段及右股骨远端亦见（但以腓骨病变为著）。无明显骨皮质破坏或软组织肿胀。
- **CT影像**：横断面显示腓骨近端病变内为混合密度，高密度区代表钙化/骨性成分，低密度区提示软组织或液性成分；边缘光滑但局部轻度不规则，周围无显著骨膜反应。
- **MRI影像**：T1加权像呈等信号至低信号（与肌肉对比），T2加权像示高信号区域伴混杂信号（提示含液体及软组织成分）；增强扫描可见病变周边强化，中心区相对低强化，符合慢性病变为特征。

**诊断分析**

结合患者26岁男性、包块发现20余年且间断疼痛1年的病史，以及影像学表现：

- **长期存在（20余年）** 暗示良性病变可能性大；近期新发疼痛提示继发变化（如软骨帽炎症或微骨折）。
- **多部位受累（腓骨、胫骨、股骨远端）** 排除单发性肿瘤，支持遗传性或系统性疾病。
- 影像特征：膨胀性生长、硬化边、钙化及混合信号，与良性骨病高度吻合；恶性肿瘤（如骨肉瘤）通常进展快且无20余年慢性过程。

**按可能性从大到小，最可能的3个具体疾病诊断：**

1. **多发性骨软骨瘤（Hereditary Multiple Exostoses, HME）**
   - 依据：典型表现为长骨干骺端多发、膨胀性骨性突起；X线/CT示钙化帽及硬化边，MRI显示软组织成分与慢性炎症。20余年病史符合HME遗传性疾病特征（常青少年期出现，成年后疼痛加重），且累及腓骨、胫骨及股骨远端。
2. **骨纤维异常增殖症（Fibrous Dysplasia, 单骨型）**
   - 依据：慢性过程伴多部位病变；X线/CT可呈"磨玻璃样"改变，但本例以钙化为主，部分符合单骨型特征。MRI信号混杂提示纤维组织与软骨成分，需与HME鉴别（HME更常见于外生性突起）。
3. **内生软骨瘤伴继发变化（Enchondroma with Secondary Changes）**
   - 依据：长骨病变中可见钙化；X线/CT示边界清晰的膨胀性病灶，MRI高信号区提示软骨基质。但单发病变更常见于手足骨，本例多部位受累支持HME优先；近期疼痛可能源于病理性骨折或囊性变。

**临床建议**

- **下一步诊疗**：立即行**穿刺活检（CT引导下）** 明确诊断，并检测相关基因（如EXT1/2突变，用于HME确认）。
- **处置方案**：
  - 若活检证实为多发性骨软骨瘤且疼痛持续，建议**手术切除腓骨近端病变**（清除病灶+修复骨质），同时评估其他部位稳定性；术后行病理及分子检测以排除恶变风险。
  - 避免保守观察：因新发疼痛提示活动性变化，需排除继发恶性转化（罕见但可能）。
- **随访计划**：6个月后复查X线/CT监测病变进展，并转诊至遗传科评估家族史及HME相关并发症（如关节畸形或神经压迫）。

本研判基于影像学特征与病史高度匹配，优先考虑良性骨病；最终诊断需病理确诊。
