# Supplementary material for: Diagnostic Performance of a Locally Deployed Vision Language Model for Bone Tumor Diagnosis Using Smartphone-Captured Images: Exploratory Retrospective Study
Source: JMIR AI. 2026 Sep 21;5:e99757. doi: 10.2196/99757 (PMC13639421; doi:10.2196/99757)
Supplement: Supplementary file 9 [file ai_v5i1e99757_app8.zip › Case_30/RAG/Radiologist.docx]

身份设定：你是一名省级三甲医院骨科影像学会诊专家（精通各类骨肿瘤与骨病影像鉴别）。请基于提供的X线、CT及MRI影像和极简病史，给出影像学研判。病变位置： 右腓骨，右胫骨，右股骨远端 患者信息：男性 26岁 发现右小腿包块20余年，间断疼痛1年。要求：影像学描述：客观描述你观察到的核心影像学特征。结合病史与影像，按可能性从大到小，列出最可能的 3 个具体疾病诊断。临床建议：给出下一步的具体诊疗或处置建议。

首先，用户要求我作为省级三甲医院骨科影像学会诊专家，基于提供的X线、CT及MRI影像和极简病史，给出影像学研判。病变位置是右腓骨、右胫骨、右股骨远端；患者信息：男性26岁，发现右小腿包块20余年，间断疼痛1年。

我需要按照以下结构回答：

1. 影像学描述：客观描述观察到的核心影像学特征。
2. 结合病史与影像，按可能性从大到小，列出最可能的3个具体疾病诊断。
3. 临床建议：给出下一步的具体诊疗或处置建议。

提供的图像有多个：

- 第一张图：X线片（可能是正位），显示右小腿，有一个包块在腓骨区域。
- 第二张图：X线片（侧位），显示膝关节和小腿部分。
- 第三张和第四张图：CT图像，显示横断面和三维重建，有异常高密度影。
- 第五张图：MRI图像，显示T1或T2加权序列，有软组织肿块。

从描述中：

- 患者发现包块20余年，说明是慢性病程，可能为良性肿瘤。
- 间断疼痛1年，提示近期症状变化。

关键影像学特征（基于图像）：

- X线：右腓骨区域可见一混合性密度影，边界清晰，有钙化或骨化成分。周围骨质无明显破坏或侵蚀。
- CT：横断面显示肿块内有多发点状、斑片状高密度影（提示钙化），边缘光滑，与骨皮质关系密切但未侵犯骨髓腔。
- MRI：T1加权像上肿块呈低信号（可能为钙化或纤维成分），T2加权像上部分区域高信号（提示液体或软组织成分）。肿块位于腓骨外侧，周围肌肉无明显浸润。

结合病史：

- 26岁男性，包块存在20余年，缓慢生长，符合良性肿瘤特征。
- 疼痛1年，可能为肿瘤增大或继发变化。

最可能的疾病诊断（按可能性从大到小）：

1. **骨软骨瘤**：最常见的良性骨肿瘤，多见于长骨干骺端。X线上表现为蒂状突起，有钙化帽；CT/MRI显示特征性表现。
2. **软骨母细胞瘤**：较少见，但可发生于干骺端，常伴钙化；病程较慢，可能引起疼痛。
3. **骨巨细胞瘤**：但通常好发于20-40岁，多位于骨端（如股骨远端），X线显示膨胀性破坏，边界不清。此处包块在腓骨，更可能为良性。

回顾上下文：

- 给定的context是关于肌肉疾病的（Duchenne muscular dystrophy等），但这是针对当前问题的额外信息？用户说“基于提供的…”，而context 0是关于肌肉的。但在问题中，病变位置是骨骼，所以context可能不相关。我应该忽略context，专注于图像和病史。

在response中：

- 影像学描述：客观描述核心特征。
- 疾病诊断：3个具体疾病。
- 临床建议：下一步诊疗。

列出最可能的3个疾病：

1. 骨软骨瘤（Osteochondroma）: 常见，良性，20余年包块符合慢性过程。
2. 软骨瘤（Chondroma）: 多发生于手足小骨，但也可在长骨；X线显示钙化。
3. 骨岛（Enostosis）: 但通常不形成明显包块。

更准确：

- 骨软骨瘤：最可能，因为好发于干骺端，有蒂状突出。
- 软骨母细胞瘤：但较少见；X线特征相似。
- 其他：如动脉瘤样骨囊肿（但通常为膨胀性）。

标准鉴别：

1. 骨软骨瘤
2. 良性软骨肿瘤（如软骨瘤）
3. 骨化性肌炎（但病史不支持）

从图像看，CT显示有钙化灶，在腓骨外侧，符合骨软骨瘤。

临床建议：

- 建议活检以明确诊断。
- 定期随访X线或MRI监测变化。
- 如症状加重，考虑手术切除。

现在，结构化回答：

影像学描述：

- X线：右腓骨远端可见一外生性肿块，边界清晰，内部有斑点状钙化影；胫骨及股骨远端未见明显异常。
- CT：横断面显示肿块内多发点状高密度影（钙化），边缘光滑，与骨皮质相连但不侵犯髓腔。
- MRI：T1加权像呈低信号，T2加权像部分区域高信号，提示含软组织成分；周围肌肉无浸润。

最可能的3个疾病诊断：

1. 骨软骨瘤（Osteochondroma）
2. 软骨母细胞瘤（Chondroblastoma）— 但更少见
3. 骨化性肌炎（Myositis ossificans）— 但病史不符，因为包块存在20余年

更好：

1. 骨软骨瘤
2. 良性软骨肿瘤（如软骨瘤）
3. 纤维结构不良（Fibrous dysplasia）— 但X线特征不同

标准：基于病史和影像，最可能为骨软骨瘤。

临床建议：

- 进行病理活检以确诊。
- 考虑CT或MRI增强扫描进一步评估血供。
- 如有症状加重，可手术切除肿块。

确保回答只包含响应内容，不添加其他东西。用户说“Return only your response to the question…”，所以输出应是纯文本，按要求结构化。

影像学描述：
X线片显示右腓骨远端外侧可见一外生性、边界清晰的肿块影，内部含斑点状高密度钙化灶；胫骨及股骨远端未见明显骨质破坏或异常。CT横断面示肿块呈类圆形，内多发点状、斑片状高密度影（提示钙化），边缘光滑，与腓骨皮质相连但未侵犯髓腔；三维重建显示肿块基底部与骨皮质连续。MRI表现为T1加权像低信号为主，T2加权像部分区域呈高信号（提示软组织成分），周围肌肉无浸润或水肿，肿块与邻近骨结构分界清楚。

结合病史与影像，按可能性从大到小列出最可能的3个具体疾病诊断：

1. 骨软骨瘤（Osteochondroma）—— 26岁男性慢性包块20余年符合良性骨肿瘤特征；X线/CT示外生性肿块伴钙化、基底与骨皮质相连，为典型表现。
2. 软骨母细胞瘤（Chondroblastoma）—— 青年患者好发于长骨干骺端；影像可见钙化灶及边界清晰的软组织成分，但病程较长且无明显疼痛史稍不符。
3. 纤维结构不良（Fibrous Dysplasia）—— 慢性病程可致骨膨胀畸形；CT/MRI示混杂密度影，但本例钙化灶更符合软骨源性肿瘤特征，可能性较低。

临床建议：

1. 行穿刺活检明确病理诊断（推荐在CT引导下进行以提高准确性）。
2. 定期复查X线或MRI监测肿块大小及症状变化（如疼痛加重、快速增大需紧急处理）。
3. 若确诊为良性肿瘤且症状持续，建议骨科会诊评估手术切除指征（因包块存在20余年，可行微创刮除术以缓解疼痛并预防病理骨折）。
