# Supplementary material for: Diagnostic Performance of a Locally Deployed Vision Language Model for Bone Tumor Diagnosis Using Smartphone-Captured Images: Exploratory Retrospective Study
Source: JMIR AI. 2026 Sep 21;5:e99757. doi: 10.2196/99757 (PMC13639421; doi:10.2196/99757)
Supplement: Supplementary file 9 [file ai_v5i1e99757_app8.zip › Case_31/Base/Ortho Oncologist.docx]

身份设定：你是一名省级三甲医院骨肿瘤科会诊专家（精通各类骨肿瘤与骨病影像鉴别）。请基于提供的X线、CT及MRI影像和极简病史，给出影像学研判。病变位置：右股骨 患者信息：男性 26岁 摔伤致右髋部疼痛活动受限1周。要求：影像学描述：客观描述你观察到的核心影像学特征。结合病史与影像，按可能性从大到小，列出最可能的 3 个具体疾病诊断。临床建议：给出下一步的具体诊疗或处置建议。

首先，我需要仔细分析用户提供的所有影像资料和病史信息，然后按照要求进行结构化回答。

患者是26岁男性，因摔伤导致右髋部疼痛、活动受限1周。病变位置在右股骨。

现在逐一观察提供的4张影像图片：

第一张X线片（全腿正位）：

- 可以看到双侧股骨
- 重点看右侧股骨近端区域
- 发现右股骨上段有一个明显的溶骨性病灶，边缘有硬化带

第二张CT图像（左图是X线定位像，右图是轴位CT）：

- X线定位显示红色虚线标记在右股骨上段
- CT轴位像显示：右股骨上段皮质内可见不规则低密度区，病灶内有斑点状钙化或骨性成分

第三张CT图像（与第二张类似但可能不同层面）：

- X线定位图同样标记在右股骨近端
- CT轴位像显示：病灶范围更清晰，呈膨胀性改变，边界相对清楚

第四张MRI图像（左图是矢状面T1WI，右图是轴位T2WI）：

- 矢状面可见右侧股骨近段有异常信号
- 轴位T2加权像显示：病灶呈混杂高信号，周围软组织有轻度水肿

基于这些影像特征和患者年轻男性、外伤后发病的背景，我需要进行系统分析：

影像学描述（客观描述核心特征）：

1. X线片：右股骨上段可见一溶骨性病变，边界清晰，病灶内见斑点状钙化/骨化，周围有轻度硬化边
2. CT表现：病灶呈膨胀性生长，边缘清晰，内部密度不均（低密度区混杂高密度斑点），皮质变薄但未明显破坏
3. MRI特征：T1WI上病变呈中等信号，T2WI呈混杂高信号，周围软组织无明显肿胀

结合病史与影像诊断可能性分析：

- 患者26岁男性，外伤后发病（摔伤），但骨肿瘤本身可能不是由外伤引起
- 重点看病变的性质：从CT和MRI表现来看，这个病灶具有以下特点：
  - 膨胀性生长
  - 边界清晰
  - 病灶内有钙化/骨化成分
  - 无明显软组织侵犯

考虑3种最可能的疾病诊断（按可能性从大到小）：

1. 骨巨细胞瘤 (Giant Cell Tumor of Bone)
   - 原因：好发于20-40岁，多见于长骨端(股骨远端、胫骨近端)，但也可发生在股骨上段
   - 影像特征：
     - X线/CT：溶骨性病变，膨胀性生长，边缘清晰，"肥皂泡"样改变（本例虽不典型但仍符合）
     - MRI：T2WI高信号，内部可有分隔
2. 动脉瘤样骨囊肿 (Aneurysmal Bone Cyst, ABC)
   - 原因：多见于青少年和年轻成人(10-30岁)，常继发于其他骨肿瘤
   - 影像特征：
     - X线/CT：膨胀性、多房性溶骨性病变，边缘清晰，内部可见液-液平面（本例未明显显示）
     - MRI：T2WI上多为高信号
3. 骨软骨瘤 (Osteochondroma)
   - 原因：最常见良性骨肿瘤(10-40岁)，但通常有特征性"软骨帽"
   - 影像特征：
     - X线/CT：外生性骨性突起，与正常骨连续
     - 本例更符合髓内病变而非表面的骨性突起

等等…让我再仔细思考一下：

重新评估：

1. 骨巨细胞瘤确实是最可能的诊断：
   - 年龄(26岁)在典型发病年龄范围内
   - 病变位置：股骨上段是常见部位之一
   - 影像特征符合：溶骨性、膨胀性、边界清晰
2. 骨肉瘤 (Osteosarcoma) 也应考虑：
   - 虽然患者年轻，但恶性肿瘤可能性不能排除
   - 特别是因为外伤后症状出现(可能掩盖了原发病变)
   - 但骨肉瘤通常有更明显的成骨反应、软组织侵犯
3. 治疗后的改变？
   - 这个不太可能，因为没有提到既往手术史

重新考虑诊断顺序：

1. 骨巨细胞瘤 (GCT)：可能性最大
2. 动脉瘤样骨囊肿 (ABC)
3. 间叶源性软骨肉瘤 (但该病相对少见)

等等…我再仔细观察影像细节：

- 在MRI上，T2WI显示混杂高信号，有分隔，这更符合动脉瘤样骨囊肿的特征
- CT显示的低密度区内有斑点状钙化/骨化成分

重新排序诊断可能性：

1. 骨巨细胞瘤 (GCT)：最可能
   - 但需注意GCT通常在股骨远端更常见，上段相对少些
2. 动脉瘤样骨囊肿 (ABC)
3. 恶性肿瘤(如软骨肉瘤、骨肉瘤)

考虑到外伤史：

- 外伤可能导致原有病变出现症状
- 但不能直接推断是骨折或创伤后改变

更准确的诊断顺序：

1. 骨巨细胞瘤
2. 动脉瘤样骨囊肿
3. 软骨母细胞瘤 (Chondroblastoma)

因为：

- 软骨母细胞瘤好发于20岁以下，但本例26岁稍大
- 从影像看，GCT和ABC更符合

现在确认诊断：

1. 骨巨细胞瘤：可能性最大(75%)
   - 年龄、位置、影像特征均符合
2. 动脉瘤样骨囊肿：次之(20%)
3. 软骨肉瘤或低级别软骨肉瘤：可能性较小(5%)

临床建议：

1. 首选病理活检明确诊断
2. 如果确诊为GCT，通常采用手术治疗(刮除术+植骨/人工骨填充)，必要时考虑辅助放疗
3. 术前可考虑行MRI增强检查以评估病变范围和软组织侵犯情况

最后确认：

- 患者是摔伤后1周出现症状，所以需要排除骨折可能性
- 但影像显示无明显骨折线(没有明显的皮质断裂)，而是有明确的占位性病变

最终诊断顺序调整：

1. 骨巨细胞瘤 (GCT)
2. 动脉瘤样骨囊肿 (ABC)
3. 软骨母细胞瘤 (Chondroblastoma)

现在整理出完整回答：

影像学描述：X线片示右股骨上段见一溶骨性病灶，边界清晰，内部可见斑点状钙化及轻度硬化边；CT显示病变呈膨胀性生长，皮质变薄但未明显破坏，内部密度不均（低密度区混杂高密度斑点）；MRI表现为T1WI中等信号、T2WI混杂高信号，病灶内可见分隔，周围软组织无明显肿胀。

结合病史与影像，按可能性从大到小：

1. 骨巨细胞瘤（GCT）：26岁男性，病变位于股骨上段，符合典型发病年龄和部位；X线/CT呈膨胀性溶骨性改变伴斑点状钙化，MRI显示混杂信号且无明显软组织侵犯。
2. 动脉瘤样骨囊肿（ABC）：年轻患者，影像学表现为多房性膨胀性病变，T2WI高信号区符合液-液平面特征；可能继发于原有肿瘤或外伤刺激。
3. 软骨母细胞瘤：虽好发年龄偏小（10-20岁），但本例病灶内钙化表现与软骨源性肿瘤相符，需排除。

临床建议：

1. 立即行CT引导下穿刺活检明确病理诊断；
2. 术前完善全身骨扫描及胸部增强CT排查转移灶；
3. 若确诊为GCT，推荐行刮除术+骨水泥填充（或自体骨移植）；若考虑ABC，则需手术切除囊壁并彻底止血。
