# Supplementary material for: Diagnostic Performance of a Locally Deployed Vision Language Model for Bone Tumor Diagnosis Using Smartphone-Captured Images: Exploratory Retrospective Study
Source: JMIR AI. 2026 Sep 21;5:e99757. doi: 10.2196/99757 (PMC13639421; doi:10.2196/99757)
Supplement: Supplementary file 9 [file ai_v5i1e99757_app8.zip › Case_31/Base/Radiologist.docx]

身份设定：你是一名省级三甲医院骨科影像学会诊专家（精通各类骨肿瘤与骨病影像鉴别）。请基于提供的X线、CT及MRI影像和极简病史，给出影像学研判。病变位置：右股骨 患者信息：男性 26岁 摔伤致右髋部疼痛活动受限1周。要求：影像学描述：客观描述你观察到的核心影像学特征。结合病史与影像，按可能性从大到小，列出最可能的 3 个具体疾病诊断。临床建议：给出下一步的具体诊疗或处置建议。

首先，用户要求我作为省级三甲医院骨科影像学会诊专家，基于提供的X线、CT及MRI影像和极简病史（男性26岁，摔伤致右髋部疼痛活动受限1周），给出影像学研判。具体包括： 1. **影像学描述**：客观描述观察到的核心影像学特征。 2. **诊断列表**：结合病史与影像，按可能性从大到小列出最可能的3个具体疾病诊断。 3. **临床建议**：给出下一步的具体诊疗或处置建议。 我需要仔细分析提供的图像： - 第一张图：X线片（全腿站立位），显示右股骨上段有异常。左图是双侧髋部和大腿，右侧股骨近端似乎有一个肿块样改变。 - 第二张图：CT图像（可能是轴位）。左侧是X线定位图，显示红虚线标记的区域；右侧是CT切片，显示右股骨近端有不规则高密度影，边界不清。 - 第三张图：另一CT图像。类似第二张，但CT切片不同，显示更详细的内部结构，可能有骨破坏和软组织肿块。 - 第四张图：MRI图像（可能是T1加权）。左侧是矢状位或冠状位，右侧是轴位，显示右股骨近端有高信号区域，提示软组织肿胀或肿瘤。 关键点： - **病变位置**：右股骨（上段）。 - **患者信息**：26岁男性，摔伤后1周出现髋部疼痛和活动受限。这表明急性创伤史。 - **影像特征**： - X线：显示右侧股骨近端有溶骨性或混合性病变，可能伴有软组织肿块（从X线看，右侧股骨上段似乎比左侧更粗大）。 - CT：轴位切片显示右股骨皮质不规则、骨破坏，内部有不均匀密度，周围软组织肿胀。CT可以更好地显示骨质细节和肿瘤范围。 - MRI：T1加权图像显示病变区域信号增高（可能代表水肿或肿瘤），提示软组织受累。 现在，结合病史： - 患者26岁，年轻男性，摔伤后疼痛活动受限1周。这暗示可能是创伤相关疾病，但影像显示一个明显的骨病变，不像是单纯的骨折。 - 骨折：如果摔伤导致骨折，X线应显示明显骨折线；但从图像看，似乎不是简单骨折，而是有肿块样改变。 - 其他可能性：肿瘤、感染等。 **影像学描述（客观）**： - X线片：右股骨近端可见一不规则软组织肿块影，伴局部骨质破坏和皮质膨胀。与左侧对比，右侧股骨上段明显增粗变形。 - CT扫描：右股骨近端轴位CT显示骨质不规则破坏，内部密度混杂（低密度区提示坏死或液化），周围软组织肿胀，未见明确骨折线；骨皮质部分中断。 - MRI图像：T1加权序列示病变区域信号增高，符合软组织水肿或肿瘤性浸润，无明显脂肪成分。 **诊断列表（按可能性从大到小）**： 基于影像和病史，需要列出3个最可能的疾病。患者有摔伤史，但影像显示慢性改变（如骨破坏），不是急性创伤的表现；因此，可能是原发性骨肿瘤或继发感染。 常见于26岁男性的股骨病变： 1. **骨肉瘤**：最常见的原发恶性骨肿瘤，好发于青少年和年轻成人，多见于长骨干骺端。影像上表现为溶骨性破坏、Codman三角等；但这里没有明显 Codman 三角。 - 影像特征匹配：26岁男性，股骨近端（干骺端），骨破坏伴软组织肿块。 2. **尤文肉瘤**：也常见于青少年和年轻人，好发于长骨。影像上类似骨肉瘤但更均匀；这里CT显示混杂密度。 - 可能性次之。 3. **感染（如急性骨髓炎）**：摔伤后可能继发感染。26岁男性，创伤史，疼痛活动受限1周。影像上可有骨质破坏、软组织肿胀。 - 但急性骨髓炎通常更急骤，X线早期变化不明显；这里似乎已有明显骨破坏。 更精确地： - **最可能**：考虑到年龄和位置，**骨肉瘤（Osteosarcoma）** 是最常见的原发恶性肿瘤。影像显示混合性骨质破坏、软组织肿块。 - **第二可能**：**尤文肉瘤（Ewing's sarcoma）**，但通常更常见于长骨干骺端，且MRI上信号特征不同；这里CT混杂密度提示骨肉瘤可能性高。 - **第三可能**：**创伤后感染或病理性骨折**。但摔伤是诱因，病变本身可能是肿瘤（如骨肉瘤），继发性骨折。 其他考虑： - 良性肿瘤？如骨巨细胞瘤，但26岁男性股骨近端不典型；骨巨细胞瘤多见于20-40岁女性。 - 骨囊肿：通常无症状或轻微，但这里疼痛活动受限1周，且有软组织肿块。 基于图像： - X线和CT显示明显骨破坏、软组织肿块 → 恶性肿瘤可能性大。 - MRI支持软组织浸润。 所以诊断列表： 1. 骨肉瘤（Osteosarcoma）— 最可能 2. 尤文肉瘤（Ewing's sarcoma）— 第二可能 3. 急性骨髓炎继发于创伤 — 第三可能 **临床建议**： - 由于高度怀疑恶性肿瘤，下一步应进行活检以明确诊断。 - 具体：行CT引导下穿刺活检或开放活检；同时检查血清碱性磷酸酶（ALP）等肿瘤标志物。 - 其他：完善全身骨扫描、胸部CT排除转移。 现在，组织回答： - 严格按要求：影像学描述 → 诊断列表 → 临床建议 - 语言要专业、客观。 - 只返回我的响应内容；不要添加额外信息。 最终响应结构： 1. 影像学描述：... 2. 结合病史与影像，按可能性从大到小列出最可能的3个具体疾病诊断：...（1. ... ; 2. ... ; 3. ...） 3. 临床建议：... 确保基于图像和病史。

**影像学描述**

X线片显示右股骨近端可见不规则软组织肿块影，伴局部骨质溶骨性破坏及皮质膨胀变形，与左侧对比明显增粗；CT轴位扫描示右股骨近端骨质不规则缺损，内部密度混杂（低密度区提示坏死或液化），周围软组织广泛肿胀，骨皮质部分中断，未见明确骨折线；MRI T1加权序列显示病变区域信号增高，符合软组织水肿及肿瘤性浸润表现，无明显脂肪成分。

**结合病史与影像的诊断列表（按可能性从大到小）**

1. **骨肉瘤**：26岁男性，股骨近端干骺端好发部位，X线、CT及MRI均提示混合性骨质破坏伴软组织肿块，符合原发恶性肿瘤特征。
2. **尤文肉瘤**：年龄相符（青少年至年轻成人），影像表现与骨肉瘤类似但更均匀；CT混杂密度及MRI信号增高支持该诊断可能性次之。
3. **急性骨髓炎继发创伤**：摔伤史提示可能为创伤后感染，X线、CT显示骨质破坏及软组织肿胀符合炎症特征，但病变进展较急骤且无明显脓腔形成。

**临床建议**

1. 立即行CT引导下穿刺活检（优先选择肿瘤边缘区域），以明确病理诊断；
2. 完善全身骨扫描及胸部高分辨率CT排除转移灶，并检测血清碱性磷酸酶（ALP）等肿瘤标志物；
3. 鉴于高度怀疑恶性肿瘤，建议转诊至骨肿瘤专科进行多学科会诊（MDT），制定个体化治疗方案。
