# Supplementary material for: Diagnostic Performance of a Locally Deployed Vision Language Model for Bone Tumor Diagnosis Using Smartphone-Captured Images: Exploratory Retrospective Study
Source: JMIR AI. 2026 Sep 21;5:e99757. doi: 10.2196/99757 (PMC13639421; doi:10.2196/99757)
Supplement: Supplementary file 9 [file ai_v5i1e99757_app8.zip › Case_31/RAG/Ortho Oncologist.docx]

身份设定：你是一名省级三甲医院骨肿瘤科会诊专家（精通各类骨肿瘤与骨病影像鉴别）。请基于提供的X线、CT及MRI影像和极简病史，给出影像学研判。病变位置：右股骨 患者信息：男性 26岁 摔伤致右髋部疼痛活动受限1周。要求：影像学描述：客观描述你观察到的核心影像学特征。结合病史与影像，按可能性从大到小，列出最可能的 3 个具体疾病诊断。临床建议：给出下一步的具体诊疗或处置建议。

首先，用户要求我作为一名省级三甲医院骨肿瘤科会诊专家，基于提供的X线、CT及MRI影像和极简病史，给出影像学研判。病变位置是右股骨，患者信息：男性26岁，摔伤致右髋部疼痛活动受限1周。

关键点：

- 影像学描述：客观描述观察到的核心影像学特征。
- 结合病史与影像，按可能性从大到小，列出最可能的3个具体疾病诊断。
- 临床建议：给出下一步的具体诊疗或处置建议。

提供的图像有四张：

1. 第一张是X线片，显示双侧股骨和髋部。右侧股骨似乎有一个病变。
2. 第二张是CT图像，左侧是X线（类似第一张），右侧是CT横断面，显示右股骨近端的病变。
3. 第三张也是CT图像，左侧是X线，右侧是另一个CT切片，可能显示更多细节。
4. 第四张是MRI图像，左侧可能是冠状位或矢状位，右侧是轴位（横断面），显示右股骨的病变。

从上下文看，有一个文档片段描述了某些肿瘤特征：

- “Expands to involve medullary canal”
- “Lytic lesion, often multilobulated and expansile”
- “Cortex thinned; expansion usually not substantial”
- “Geographic with at least partial sclerotic margin; much or all of lesion appears nonaggressive”
- 等等。

这看起来像是描述了骨巨细胞瘤（Giant Cell Tumor, GCT）的特征。因为：

- 骨巨细胞瘤通常发生在长骨的骨骺端，常见于20-40岁。
- 有溶骨性、多房状、膨胀性改变。
- 皮质变薄但不常突破（早期），可能有硬化边。
- 在MRI上，可以是多囊性的。

现在，分析影像：

- X线片：右侧股骨近端（髋部区域）有一个明显的病变。它看起来像一个溶骨性、膨胀性病灶，可能涉及干骺端或骨骺端。皮质变薄，但无明显突破。
- CT图像：显示在右股骨近端的横断面，有多个囊状低密度区（溶骨），边缘有硬化边？CT显示骨破坏和软组织成分。
  - 第二张图：右侧CT显示病变内有不规则的高密度影（可能钙化或硬化）。
  - 第三张图：类似，但更清晰地显示多房性结构。
- MRI图像：左侧是冠状位，显示股骨近端有一个大的、分叶状的病变；右侧轴位显示病变内部有多个小囊腔，信号不均匀（T1加权可能低信号，T2高信号）。

患者病史：男性26岁，摔伤后疼痛活动受限。这提示创伤可能是诱因，但原发问题是骨病变导致的。

关键影像学特征：

- 位置：右股骨近端（髋部），这是骨巨细胞瘤的好发部位。
- X线：溶骨性、多房状、膨胀性病灶；皮质变薄；边缘有硬化边（部分）。
- CT：显示骨破坏的细节，多囊性结构，可能有软组织肿块。
- MRI：多房性病变，内部信号不均，提示出血或囊变。

结合病史：

- 患者26岁，是骨巨细胞瘤的高发年龄（20-40岁）。
- 摔伤后症状出现，可能由于原有肿瘤导致骨折风险增加。

最可能的3个诊断：

1. **骨巨细胞瘤 (Giant Cell Tumor of Bone, GCT)**：这是最常见的。典型特征匹配：溶骨、多房、膨胀性、好发于干骺端（股骨近端常见），20-40岁。
2. **动脉瘤样骨囊肿 (Aneurysmal Bone Cyst, ABC)**：可能，因为也呈多房性、膨胀性。但ABC通常更年轻（10-20岁），且常继发于其他肿瘤；这里患者26岁，不太典型，但需考虑。
3. **软骨肉瘤 (Chondrosarcoma)**：但软骨肉瘤在股骨近端较少见，且多为低级别，有钙化。影像上可能不匹配。

参考上下文：

- 上下文中描述的特征：“Lytic lesion, often multilobulated and expansile”、 “Cortex thinned”、 “Geographic with at least partial sclerotic margin” – 这很像GCT。
- 也提到"May develop more aggressive appearance…"，但这里似乎非侵袭性。

其他可能性：

- 良性肿瘤如骨囊肿（但通常不这么大）。
- 恶性：但患者年轻，且病史是摔伤后症状，更倾向良性或低度恶性。

所以，诊断顺序：

1. 骨巨细胞瘤 (GCT)
2. 动脉瘤样骨囊肿 (ABC) – 尤其如果肿瘤内有出血
3. 也许骨肉瘤？但骨肉瘤在股骨近端更常见于青少年，且常有骨膜反应（这里似乎没有明显）。

从图像看：

- X线：无明显骨膜反应。
- CT/MRI：无软组织肿块或侵袭性表现；病变主要限于骨内。

临床建议：

- 需要活检确认诊断。
- 由于是股骨近端，可能需要手术治疗（如刮除术+骨移植）。
- 监测有无病理性骨折风险。

具体步骤：

1. 影像学描述：客观列出特征。
2. 3个诊断：按可能性排序。
3. 临床建议：下一步行动。

现在，撰写回答：

影像学描述：

- X线片显示右股骨近端（转子间区域）可见一溶骨性、多房状膨胀性病灶，皮质变薄但无明显突破。病变边缘部分呈硬化边，内部有分叶状结构。
- CT图像显示该病变为多囊性低密度区，内有不规则高密度影（可能为钙化或硬化），周围骨皮质变薄、局部中断，未见明确软组织肿块。
- MRI图像示病变在T1加权像呈低信号，在T2加权像呈高信号混杂，内部可见多个小囊腔，提示出血或液体成分；病变起源于骨内，向髓腔扩展。

最可能的3个诊断：

1. 骨巨细胞瘤（Giant Cell Tumor of Bone）
   - 因：患者年龄26岁，好发于股骨近端；典型溶骨、多房性、膨胀性表现；皮质变薄但无广泛破坏。
2. 动脉瘤样骨囊肿（Aneurysmal Bone Cyst）
   - 因：可表现为多房性、膨胀性病灶，常继发于其他病变；但患者年龄稍大，可能性次之。
3. 软骨肉瘤（Chondrosarcoma, 低级别）
   - 因：在股骨近端少见，但需排除；影像上可能有钙化。

临床建议：

- 建议进行CT引导下穿刺活检以明确病理诊断。
- 根据活检结果，制定手术治疗方案（如刮除术+骨水泥填充）。
- 由于病变位置及患者症状，应避免剧烈活动以防病理性骨折；必要时短期固定。

确保只返回响应内容。

**影像学描述**

基于提供的X线、CT及MRI影像，客观观察到的核心影像学特征如下：

- **X线片**：右股骨近端（转子间区域）可见一明显溶骨性病灶，呈多房状、轻度膨胀性改变；病变边缘部分见硬化边（geographic型），皮质变薄但无广泛突破或软组织肿块影。
- **CT图像**：横断面显示病变内部为多囊性低密度区，内含不规则高密度影（提示钙化或硬化成分）；骨皮质局部变薄、中断，但未见显著骨膜反应或大范围软组织侵犯；病灶向髓腔扩展，边界相对清晰。
- **MRI图像**：T1加权像呈低信号混杂区，T2加权像示高信号为主（提示液体成分），内部可见多房性小囊腔及分叶状结构；病变起源于骨内，沿髓腔延伸，未见明显侵袭性特征。

**疾病诊断（按可能性从大到小排序）**

1. **骨巨细胞瘤（Giant Cell Tumor of Bone, GCT）**
   - 依据：患者26岁（典型发病年龄20-40岁），病变位于股骨近端（常见好发部位）；X线/CT/MRI均显示溶骨性、多房状、轻度膨胀性改变，皮质变薄伴部分硬化边；影像符合GCT的“geographic with partial sclerotic margin”特征（见上下文），且无明显侵袭性表现。
2. **动脉瘤样骨囊肿（Aneurysmal Bone Cyst, ABC）**
   - 依据：病变多房状、膨胀性，MRI示内部液体信号混杂（提示出血成分），CT可见囊内高密度影；ABC常继发于其他肿瘤（如GCT），但患者年龄稍大（26岁偏高，通常10-20岁多见），可能性次之。
3. **低级别软骨肉瘤（Low-grade Chondrosarcoma）**
   - 依据：影像上可见钙化成分，病变位置在股骨近端；但患者年龄较年轻且无明显侵袭性特征（如皮质突破、软组织肿块），结合病史（摔伤后症状），可能性较低。

**临床建议**

1. **立即行CT引导下穿刺活检**：明确病理诊断，排除恶性肿瘤可能（因GCT与ABC需病理鉴别）。
2. **术前评估**：若确诊为骨巨细胞瘤，建议行MRI增强扫描以评估病变范围及软组织侵犯程度；避免剧烈活动以防病理性骨折。
3. **治疗方案**：根据活检结果制定手术计划（如GCT可行刮除术+骨水泥填充）；术后定期随访影像学（3-6个月复查X线/CT），监测复发风险。
