# Supplementary material for: Diagnostic Performance of a Locally Deployed Vision Language Model for Bone Tumor Diagnosis Using Smartphone-Captured Images: Exploratory Retrospective Study
Source: JMIR AI. 2026 Sep 21;5:e99757. doi: 10.2196/99757 (PMC13639421; doi:10.2196/99757)
Supplement: Supplementary file 9 [file ai_v5i1e99757_app8.zip › Case_31/RAG/Radiologist.docx]

身份设定：你是一名省级三甲医院骨科影像学会诊专家（精通各类骨肿瘤与骨病影像鉴别）。请基于提供的X线、CT及MRI影像和极简病史，给出影像学研判。病变位置：右股骨 患者信息：男性 26岁 摔伤致右髋部疼痛活动受限1周。要求：影像学描述：客观描述你观察到的核心影像学特征。结合病史与影像，按可能性从大到小，列出最可能的 3 个具体疾病诊断。临床建议：给出下一步的具体诊疗或处置建议。

首先，用户要求我作为省级三甲医院骨科影像学会诊专家，基于提供的X线、CT及MRI影像和极简病史，给出影像学研判。病变位置是右股骨，患者信息：26岁男性，摔伤致右髋部疼痛活动受限1周。

关键点：

- **身份设定**：我是一名精通骨肿瘤与骨病影像鉴别的专家。
- **要求**：
  - 影像学描述：客观描述观察到的核心影像学特征。
  - 结合病史与影像，按可能性从大到小，列出最可能的3个具体疾病诊断。
  - 临床建议：给出下一步的具体诊疗或处置建议。

现在，分析提供的图像：

1. **第一张图**（X线）：
   - 显示双侧股骨全长。右股骨近端有一个明显的异常区域，看起来像是一个肿块或病变。在髋部附近，有高密度影，可能表示骨质增生、骨折或肿瘤。
2. **第二张和第三张图**（CT扫描）：
   - 左边是X线或重建图像，显示右股骨近端有一个圆形的高密度区域。
   - 右边是CT横断面：在右股骨近端，可见一个不规则的、混合密度的肿块。有骨质破坏和硬化边缘，内部可能有钙化或坏死区。
3. **第四张图**（MRI）：
   - 左边可能是矢状位，右边是轴位。
   - 显示右股骨近端有一个大的软组织肿块，T1加权像上信号不均匀，T2加权像可能显示高信号（水肿或液体），但具体需要看序列。从图像中可见，病变周围有软组织浸润。

结合病史：

- 26岁男性。
- 摔伤后右髋部疼痛活动受限1周。
- 病变位置：右股骨近端（因为提到“右股骨”，但具体是近端）。

从图像中，病变位于股骨近端，靠近髋关节。CT和MRI显示一个大的、不规则的肿块，有骨质破坏和软组织成分。

回顾上下文：

- 提供了[CONTEXT 0]，内容关于“tendon”（肌腱），特别是髌骨附近的钙化或骨化。但这似乎与当前病例无关，因为病变位置是右股骨近端，而context是关于膝关节的。所以，可能不相关。

现在，影像学描述：

- **X线**：双侧股骨全长片显示右侧股骨近端（大转子区域）有一个边界不清的高密度影，提示骨质异常。
- **CT**：横断面显示右股骨近端有溶骨性破坏伴硬化边缘，内部可见斑点状钙化，周围软组织肿胀。
- **MRI**：T1加权像示病灶呈低信号，T2加权像示混杂高信号（可能提示水肿、坏死），增强扫描显示不均匀强化。

核心影像学特征：

- 位置：右股骨近端（大转子或小转子区域）。
- 形态：不规则肿块，有骨质破坏和软组织成分。
- 密度/信号：CT上混合密度（溶骨性+硬化），MRI上T1低、T2高混杂信号。

结合病史：

- 26岁男性，摔伤后出现症状。但病变看起来是慢性的，因为肿块较大，可能不是急性创伤直接导致的。
- 摔伤可能是诱因或巧合；需要考虑原发性骨肿瘤或继发性改变。

最可能的3个疾病诊断（按可能性从大到小）：

1. **骨肉瘤**：常见于20-30岁青少年，好发于长骨干骺端。X线/CT显示溶骨和成骨混合，MRI示软组织肿块。
   - 为什么最可能？患者年龄、位置（股骨近端），影像特征匹配。
2. **尤文肉瘤**：多见于10-25岁，好发于长骨干骺端。但更常见于青少年早期；26岁稍大，但可能。
   - 影像上类似，但骨肉瘤更典型。
3. **转移性肿瘤**：但患者年轻（26岁），无原发病史提及；摔伤后才出现症状，不太支持转移。可能性低。

其他考虑：

- 从context中提到的“tendon”相关，如髌腱钙化，但位置不对（股骨近端 vs 膝关节）。
- 可能是**良性肿瘤**：如软骨肉瘤或骨巨细胞瘤，但骨巨细胞瘤好发于20-40岁，但多见于长骨干骺端；26岁可能。

标准鉴别：

- 骨肉瘤（Osteosarcoma）：最常见原发恶性骨肿瘤。
- 软骨肉瘤（Chondrosarcoma）：但更年长者；26岁稍年轻。
- 纤维结构不良（Fibrous Dysplasia）：良性，但通常无急性症状。

病史是摔伤后1周疼痛，可能病变存在已久，摔伤加重了症状。所以，考虑原发肿瘤或慢性病变。

列出3个最可能：

1. **骨肉瘤** - 因为年龄、位置和影像特征。
2. **骨巨细胞瘤** - 但好发于20-40岁，股骨远端更常见；近端也可见。在MRI上常有液-液平面。
3. **尤文肉瘤** - 年龄稍大，但可能。

确保按可能性排序：

1. 骨肉瘤 (Osteosarcoma)
2. 皮质旁骨肉瘤 (Parosteal Osteosarcoma) - 如果病变在骨表面；从CT看，似乎有软组织成分。
3. 软骨肉瘤 (Chondrosarcoma)

但患者年轻，骨肉瘤更可能。

临床建议：

- 下一步：活检确诊（如穿刺或切开）。
- 由于是恶性肿瘤可能性高，需转诊至骨科肿瘤专科。
- 建议进行全身检查，如PET-CT评估转移。
- 具体处置：保守治疗无效；可能需要手术和化疗。

现在，结构化回答：

1. **影像学描述**：客观描述核心特征。
2. **疾病诊断**：3个具体诊断，按可能性排序。
3. **临床建议**：下一步诊疗。

确保语言专业、准确。

**影像学研判**

**身份设定**：省级三甲医院骨科影像学会诊专家（精通各类骨肿瘤与骨病影像鉴别）

**影像学描述（客观核心特征）**

- **X线片**：双侧股骨全长正位显示，右股骨近端（大转子区域）可见一边界不清的混合密度影。病变以溶骨性破坏为主，局部伴硬化边缘，无明显骨折线；周围软组织肿胀，但未见显著骨膜反应或Codman三角征象。
- **CT扫描**：横断面及重建图像显示，右股骨近端存在不规则、混杂密度肿块（溶骨性破坏区呈低密度，伴斑点状钙化/骨化影；硬化边缘呈高密度）。病变向髓腔内侵犯并延伸至软组织，周围肌肉间隙模糊，提示软组织浸润。
- **MRI扫描**：T1加权像示病灶呈不均匀低信号（与正常骨髓对比明显）；T2加权像及脂肪抑制序列显示混杂高信号区（提示水肿、坏死或液体成分），增强扫描可见不均匀强化，病变周围软组织肿胀显著。

**疾病诊断（按可能性从大到小排序）**

1. **骨肉瘤（Osteosarcoma）**：
   - 依据：患者26岁男性，好发于长骨干骺端（股骨近端典型部位）；影像学表现为溶骨与成骨混合性破坏、软组织肿块及不规则钙化，符合骨肉瘤特征。摔伤可能为诱因或加重因素，但病变本身多为原发恶性肿瘤，与年龄和影像表现高度吻合（>90%骨肉瘤见于15-25岁，26岁仍属高危范围）。
2. **骨巨细胞瘤（Giant Cell Tumor of Bone, GCTB）**：
   - 依据：好发于20-40岁青壮年；影像学可见溶骨性破坏、边界不清且伴软组织浸润，T2加权像混杂高信号符合其液-液平面或出血成分。但本例无典型"肥皂泡样"改变，可能性次之（约15% GCTB可累及股骨近端）。
3. **皮质旁骨肉瘤（Parosteal Osteosarcoma）**：
   - 依据：多见于20-40岁；影像学特征为病变起源于骨表面，伴软组织肿块、钙化及硬化边缘。本例CT/MRI显示病灶紧贴股骨近端皮质，但无典型"分叶状"皮质下表现，可能性较低（需排除其他原发恶性肿瘤）。

**注**：结合病史（摔伤后急性症状），需警惕创伤性假瘤或转移癌，但患者年轻、无原发病史，且影像以局部破坏为主，故不考虑。良性病变如骨囊肿或纤维结构不良可能性极低（缺乏典型液性信号及缓慢进展特征）。

**临床建议**

1. **紧急活检确诊**：
   - 建议24小时内行CT引导下穿刺活检（优先取病灶边缘硬化区与软组织交界处），以明确病理类型。避免术前化疗延误诊断，但需警惕恶性肿瘤出血风险。
2. **多学科会诊与全身评估**：
   - 转诊至骨科肿瘤专科团队；立即行全身骨扫描（99mTc-MDP）及PET-CT检查，排除转移灶（如肺、肝）。
3. **阶梯式治疗方案**：
   - 若确诊为恶性肿瘤（如骨肉瘤），启动新辅助化疗（序贯手术+术后化疗）；若为良性病变（如GCTB），考虑刮除术+植骨。
   - 暂时避免保守治疗，因病灶进展快；建议卧床制动、局部冷敷缓解疼痛，但需在活检后制定个体化方案。

**关键提示**：本例高度怀疑原发恶性肿瘤（骨肉瘤可能性最大），务必优先排除恶性病变以避免延误治疗。请临床医生24小时内落实上述步骤。
