# Supplementary material for: Diagnostic Performance of a Locally Deployed Vision Language Model for Bone Tumor Diagnosis Using Smartphone-Captured Images: Exploratory Retrospective Study
Source: JMIR AI. 2026 Sep 21;5:e99757. doi: 10.2196/99757 (PMC13639421; doi:10.2196/99757)
Supplement: Supplementary file 9 [file ai_v5i1e99757_app8.zip › Case_32/Base/Ortho Oncologist.docx]

身份设定：你是一名省级三甲医院骨肿瘤科会诊专家（精通各类骨肿瘤与骨病影像鉴别）。请基于提供的X线、CT及MRI影像和极简病史，给出影像学研判。病变位置：右股骨 患者信息：男性 48岁右髋部疼痛2年加重2个月。要求：影像学描述：客观描述你观察到的核心影像学特征。结合病史与影像，按可能性从大到小，列出最可能的 3 个具体疾病诊断。临床建议：给出下一步的具体诊疗或处置建议。

首先，我需要基于提供的X线、CT和MRI影像以及病史来研判右股骨病变。患者是48岁男性，右髋部疼痛2年，加重2个月。

**步骤1: 观察影像学特征**

- **X线图像（第一张和第二张）：**
  - 第一张：显示右股骨近端（包括大转子区域）。可见股骨头、颈及干骺端。在股骨上段外侧，似乎有一个不规则的病变区域，边界模糊。
  - 第二张：是另一角度的X线片，可能为斜位或侧位。同样显示右股骨近端，病变区域更明显，有溶骨性改变和可能的皮质破坏。
- **CT图像（第三张）：**
  - 左侧：冠状位重建图，显示双侧髋关节。右侧股骨上段有一个不规则低密度区，周围有骨质增生。
  - 右侧：轴位切片，清晰显示病变区域的内部结构：有囊性变、钙化点或骨化影，皮质部分破坏，周围软组织肿胀。
- **MRI图像（第四张）：**
  - 左侧：冠状位T2加权像。右侧股骨近端可见高信号区，表明液体成分或肿瘤活跃区域。
  - 右侧：轴位T1/T2序列。病变在T1上呈低信号，在T2上呈高信号，提示含液性病灶；周围有软组织肿胀。

**核心影像学特征总结（客观描述）：**

- **X线：** 右股骨近端干骺端可见溶骨性破坏区，边界不清，局部皮质变薄、中断，无明显Codman三角。
- **CT：** 病变内见囊性坏死及散在钙化/骨化灶；骨质不规则破坏，周围软组织肿块形成。
- **MRI：** T1加权像呈低信号，T2加权像呈高信号（提示富含液体），病变向髓腔内延伸并突破皮质至软组织。

**步骤2: 结合病史分析**

- 病史：48岁男性，右髋部疼痛2年（慢性）、加重2个月（近期进展）。
- 无明确外伤史、感染史等。考虑肿瘤性病变可能性大。

**步骤3: 列出最可能的3个诊断（按可能性排序）**

1. **骨肉瘤：**
   - 常见于青少年，但48岁也可发生（继发性或去分化型）。X线可见溶骨+成骨混合表现；CT/MRI显示软组织肿块、皮质破坏。本例有溶骨性破坏和软组织侵犯，符合。
2. **转移性骨肿瘤：**
   - 48岁男性需警惕原发癌（如前列腺癌、肺癌）转移至股骨。X线/CT可见多灶性破坏；MRI显示高信号肿块。但本例无其他部位病灶提示，可能性稍低。
3. **巨细胞瘤：**
   - 多见于20-40岁女性，48岁男性较少见。典型表现：偏心性溶骨、膨胀性生长、"肥皂泡"样改变；X线/CT可见囊状破坏区伴骨膜反应不明显。本例有类似特征（如囊变），但年龄稍大。

**步骤4: 临床建议**

- **确诊：** 行穿刺活检（CT引导下）明确病理。
- **进一步检查：**
  - 全身骨扫描（排除多发转移）
  - 胸部CT（排查原发肿瘤，如肺癌）
  - 血清碱性磷酸酶、肿瘤标志物检测
- **治疗准备：**
  - 若确诊为恶性肿瘤，需多学科会诊（MDT）制定方案。
  - 暂时避免剧烈活动以防病理性骨折。

**验证思路：**

- 重点检查是否遗漏关键特征：
  - X线中"无Codman三角"→ 排除典型骨肉瘤？但本例有软组织肿块，仍需考虑（去分化骨肉瘤）。
  - CT显示钙化/骨化灶→ 巨细胞瘤通常无此表现；转移瘤多为单一溶骨性破坏。
- 最可能诊断：结合年龄、部位及影像特点，**骨肉瘤**可能性最大（尤其48岁男性需警惕继发性骨肉瘤）。

最终答案应严格按格式要求：

1. 影像学描述
2. 3个诊断（从大到小）
3. 临床建议

确保语言专业、简洁。

**影像学描述**

- **X线：** 右股骨近端干骺端见不规则溶骨性破坏区，边界模糊，局部皮质变薄并中断；无明显Codman三角，未见显著成骨反应。
- **CT：** 病变呈偏心性囊状低密度影（内含散在钙化/骨化点），皮质不规则破坏伴软组织肿块形成；髓腔内可见不均匀强化，周围骨膜增生不明显。
- **MRI：** T1加权像呈低信号，T2加权像呈高信号（提示液性成分）；病变向髓腔内延伸并突破皮质至软组织，周围见轻度水肿带，未见明显坏死区。

**诊断（按可能性从大到小排序）**

1. **骨肉瘤**（去分化型或继发性骨肉瘤）：48岁男性中年发病，溶骨+成骨混合破坏、软组织肿块及髓腔侵犯符合恶性肿瘤特征；CT示钙化/骨化灶提示高分化成分。
2. **转移性骨肿瘤**（如前列腺癌、肺癌等原发灶转移）：慢性疼痛加重史，X线/CT显示单发溶骨性破坏伴软组织浸润；需排除远处病灶。
3. **巨细胞瘤**：偏心性囊状膨胀性破坏符合典型表现，但年龄稍大（多见于20-40岁），且MRI未见"肥皂泡"样分隔结构，可能性较低。

**临床建议**

1. **立即行CT引导下穿刺活检**明确病理类型（优先排除恶性肿瘤）。
2. **完善全身检查**：
   - 全身骨扫描+胸部增强CT（排查转移灶及原发肿瘤）；
   - 血清碱性磷酸酶、肿瘤标志物（如PSA、CEA）检测。
3. **多学科会诊**：若确诊恶性，联合骨科、肿瘤内科制定综合治疗方案（术前新辅助化疗+保肢手术）。
4. **避免负重活动**：预防病理性骨折风险，必要时行临时内固定。
