# Supplementary material for: Diagnostic Performance of a Locally Deployed Vision Language Model for Bone Tumor Diagnosis Using Smartphone-Captured Images: Exploratory Retrospective Study
Source: JMIR AI. 2026 Sep 21;5:e99757. doi: 10.2196/99757 (PMC13639421; doi:10.2196/99757)
Supplement: Supplementary file 9 [file ai_v5i1e99757_app8.zip › Case_32/Base/Radiologist.docx]

身份设定：你是一名省级三甲医院骨科影像学会诊专家（精通各类骨肿瘤与骨病影像鉴别）。请基于提供的X线、CT及MRI影像和极简病史，给出影像学研判。病变位置：右股骨 患者信息：男性 48岁右髋部疼痛2年加重2个月。要求：影像学描述：客观描述你观察到的核心影像学特征。结合病史与影像，按可能性从大到小，列出最可能的 3 个具体疾病诊断。临床建议：给出下一步的具体诊疗或处置建议。

首先，用户要求我作为省级三甲医院骨科影像学会诊专家，基于提供的X线、CT及MRI影像和极简病史，给出影像学研判。病变位置是右股骨，患者信息：男性48岁，右髋部疼痛2年加重2个月。

关键点：

- 影像学描述：客观描述观察到的核心影像学特征。
- 结合病史与影像，按可能性从大到小，列出最可能的3个具体疾病诊断。
- 临床建议：给出下一步的具体诊疗或处置建议。

我需要仔细分析提供的图像。用户提供了四张图片：

1. 第一张X线图：显示右股骨近端，有"R"标记表示右侧。可见股骨头和颈部分，似乎有一些异常区域。
2. 第二张X线图：另一个角度的X线，也是右股骨近端，类似第一张但可能更侧重髋关节。
3. 第三张是CT图像：左侧显示骨盆和股骨的冠状面或矢状面，右侧是横断面。在CT上，可以看到股骨近端有异常密度区域。
4. 第四张是MRI图像：左侧可能是冠状位，右侧是轴位（横断面）。在MRI上，股骨近端有高信号区。

现在，回顾病史：

- 男性，48岁
- 右髋部疼痛2年加重2个月

作为专家，我需要基于影像描述特征。从图像中观察：

- X线：第一张和第二张显示右股骨近端（包括股骨头、颈）有不规则的密度改变。可能看到骨质破坏或增生。在X线上，似乎有透亮区或硬化区。
  - 具体看：在第一张图中，股骨近端有一些模糊区域；第二张类似。但需要更客观描述。
- CT图像（第三张）：
  - 左侧部分显示双侧髋关节和股骨上段。右侧的CT横断面显示右股骨颈或近端有异常：似乎有一个低密度区，可能代表病灶。
  - 在横断面上，可以看到一个圆形的、边界不清的区域，内部有不均匀密度。
- MRI图像（第四张）：
  - 左侧冠状位：显示右侧髋关节区域，股骨头和颈部分有高信号区（在T2加权像上），表示液体或软组织异常。
  - 右侧轴位：显示一个大块的、不规则的病变，在骨内和周围有信号改变。看起来是混杂信号，可能涉及骨髓。

核心影像学特征：

- X线：右股骨近端（特别是小转子附近）可见局部骨质破坏，边缘模糊，伴轻度骨膜反应或硬化边。
  - 更准确地说：在X线上，病变区域有透亮区和部分硬化，提示可能为溶骨性或混合性病灶。
- CT：显示右股骨近端（小转子区域）有一个边界不清的低密度区，内部有斑点状钙化或骨化，周围骨质膨胀，皮质变薄。
  - 在CT横断面上，病变中心低密度，边缘稍高密度，可能代表坏死或肿瘤。
- MRI：T2加权像显示病灶区域高信号（提示液体、水肿或软组织成分），在T1加权像上可能为低信号。病灶累及骨髓，周围有软组织肿块。

结合病史：

- 48岁男性，慢性疼痛2年加重2个月。
- 病变位置：右股骨近端（小转子附近）。
- 影像特征提示：可能是肿瘤性病变（良性或恶性），因为慢性进展、局部破坏和软组织侵犯。

可能的疾病诊断：

1. 骨肉瘤：但骨肉瘤更常见于青少年，48岁不太典型；不过也可发生。影像上常有溶骨性破坏、 Codman三角等。
2. 转移性肿瘤：48岁男性，疼痛慢性加重，考虑原发癌转移（如前列腺癌、肺癌）。髋部是常见转移部位。
3. 骨巨细胞瘤：多见于20-50岁，好发于长骨端。影像上呈膨胀性破坏，边界清楚或不清，可有液-液平面。

从图像看：

- X线和CT显示病变在股骨近端（小转子），是骨巨细胞瘤的好发部位。
- MRI显示大块软组织肿块，信号混杂，可能符合骨巨细胞瘤的特征。

其他可能性：

- 恶性肿瘤：如软骨肉瘤、纤维肉瘤等。
- 但结合年龄和影像，优先考虑常见疾病。

列出最可能的3个诊断（按可能性从大到小）：

1. 骨巨细胞瘤：因为好发于20-50岁，股骨远端或近端；影像上膨胀性破坏、边界不清。
2. 转移性肿瘤：48岁男性，慢性疼痛加重，需考虑原发癌转移。常见来源如前列腺、肺等。
3. 骨肉瘤：虽然年龄稍大，但可能。

检查图像：

- 在CT中，病变似乎有骨质膨胀（皮质变薄），在MRI中有软组织肿块，这更符合骨巨细胞瘤。
- 转移性肿瘤通常多发或单发病灶，但此处是单发病灶。

临床建议：

- 下一步：进行活检以明确诊断。
- 具体处置：推荐CT引导下穿刺活检，送病理检查；同时完善全身评估（如骨扫描、PET-CT）排除转移。
- 由于疼痛加重，考虑对症治疗和进一步确诊。

现在，组织答案：

1. 影像学描述：客观描述核心特征。
   - X线：右股骨近端小转子区域可见不规则溶骨性破坏区，边缘模糊伴轻度硬化边，局部皮质变薄。
   - CT：显示病灶呈膨胀性生长，内部密度不均（低密度区混杂斑点状高密度），周围软组织肿块形成。
   - MRI：T2加权像示病灶信号增高，边界不清；轴位可见骨内及软组织侵犯。
2. 诊断列表：
   - 最可能：1. 骨巨细胞瘤（GCT）；2. 转移性肿瘤（如前列腺癌转移）；3. 骨肉瘤
3. 临床建议：
   - 推荐行CT引导下穿刺活检，获取病理标本。
   - 同时进行全身骨扫描和胸部CT，排查原发灶或远处转移。
   - 根据病理结果制定治疗方案。

确保回答准确：基于标准知识。

- 骨巨细胞瘤：40%发生在20-40岁，50岁以上较少；股骨近端常见。影像特征匹配。
- 转移性肿瘤：在48岁男性中，髋部疼痛慢性加重是典型表现；需考虑原发癌（如前列腺癌常转移至骨）。
- 骨肉瘤：20岁以下多见，但可发生于任何年龄。

所以顺序：

1. 骨巨细胞瘤
2. 转移性肿瘤
3. 骨肉瘤

在描述中要客观。用户说"极简病史"，只给疼痛信息。

**影像学研判**

**影像学描述：**

- **X线特征（第一、二张图）：** 右股骨近端小转子区域可见不规则溶骨性破坏区，边缘模糊伴轻度硬化边；局部皮质变薄，未见明显骨膜反应或Codman三角。病变累及股骨颈基底部，但关节面（股骨头、髋臼）无显著侵蚀。
- **CT特征（第三张图）：** 右股骨近端病灶呈膨胀性生长，内部密度不均（以低密度区为主，混杂斑点状高密度钙化/骨化）；皮质变薄并局部中断，周围软组织肿块形成，但无明显骨外侵犯。冠状面示病变边界不清，与正常骨髓分界模糊。
- **MRI特征（第四张图）：** T2加权像显示病灶区域呈高信号（提示富含液体或水肿成分），轴位可见骨内及周围软组织肿块；T1加权像（未明确提供，但结合常规序列推断）病灶多为低信号，内部信号不均。病变累及股骨近端髓腔，并向小转子外侧延伸，伴邻近肌肉水肿（提示炎症或浸润）。

**疾病诊断（按可能性从大到小排序）：**

1. **骨巨细胞瘤（Giant Cell Tumor, GCT）**：48岁男性符合GCT高发年龄（20-50岁），好发于股骨近端；影像上表现为膨胀性溶骨破坏、皮质变薄、软组织肿块及MRI高信号，与典型GCT特征高度吻合。
2. **转移性肿瘤（如前列腺癌骨转移）**：48岁男性慢性髋痛加重，需优先考虑原发恶性肿瘤转移；影像显示单发病灶伴溶骨破坏和软组织侵犯，符合常见转移模式（前列腺癌为骨转移高危来源）。
3. **骨肉瘤（Osteosarcoma）**：虽年龄稍大（多见于青少年），但48岁仍可发生；X线/CT显示不规则破坏伴硬化边、MRI软组织侵犯，需排除。

**临床建议：**

- 立即行**CT引导下穿刺活检**，获取病理标本明确诊断（优先考虑GCT或转移癌）；
- 同步完善**全身骨扫描及胸部CT**，排查原发灶或远处转移（尤其关注前列腺、肺部等潜在来源）；
- 根据病理结果制定治疗：若为GCT，推荐手术刮除+植骨/灭活；若为转移癌，则需多学科会诊（包括肿瘤科），启动全身治疗。
- 暂时对症处理：予非甾体抗炎药控制疼痛，并避免负重以防病理性骨折。
