# Supplementary material for: Diagnostic Performance of a Locally Deployed Vision Language Model for Bone Tumor Diagnosis Using Smartphone-Captured Images: Exploratory Retrospective Study
Source: JMIR AI. 2026 Sep 21;5:e99757. doi: 10.2196/99757 (PMC13639421; doi:10.2196/99757)
Supplement: Supplementary file 9 [file ai_v5i1e99757_app8.zip › Case_32/RAG/Ortho Oncologist.docx]

身份设定：你是一名省级三甲医院骨肿瘤科会诊专家（精通各类骨肿瘤与骨病影像鉴别）。请基于提供的X线、CT及MRI影像和极简病史，给出影像学研判。病变位置：右股骨 患者信息：男性 48岁右髋部疼痛2年加重2个月。要求：影像学描述：客观描述你观察到的核心影像学特征。结合病史与影像，按可能性从大到小，列出最可能的 3 个具体疾病诊断。临床建议：给出下一步的具体诊疗或处置建议。

首先，用户要求我作为骨肿瘤科会诊专家，基于提供的X线、CT及MRI影像和病史（男性48岁，右髋部疼痛2年加重2个月），给出影像学研判。病变位置是右股骨。

关键部分：

- 影像学描述：客观描述核心影像学特征。
- 结合病史与影像，列出最可能的3个具体疾病诊断（按可能性从大到小）。
- 临床建议：下一步的具体诊疗或处置建议。

提供的图像包括X线、CT和MRI。我需要仔细分析这些图像。

回顾上下文：

- [CONTEXT 0] 提到了一些骨病变特征：如"Expands to involve medullary canal", “Lytic lesion, often multilobulated and expansile”, “Cortex thinned; expansion usually not substantial”, "Geographic with at least partial sclerotic margin"等。这似乎与骨肿瘤相关。
- [CONTEXT 1] 是关于创伤性神经瘤的，但病变位置是右股骨，所以可能不相关。
- [CONTEXT 2] 和 [CONTEXT 3] 也似乎是其他部位（如膝关节）的病变，不直接相关。

现在，分析提供的图像：

1. **X线图像**：显示右股骨。从描述中看，有"R"标记表示右侧。在X线上，可能看到一个病变区域。
   - 在第一个X线图（AP view），似乎股骨近端有一个异常区域。具体特征：可能是溶骨性或混合性病变。
   - 第二个X线图（Lateral view）显示类似。
2. **CT图像**：左侧是冠状位，右侧是横断面。在CT上，可能看到皮质破坏、软组织肿块等。
3. **MRI图像**：左侧是矢状位或冠状位T2加权像，右侧是轴位T1或T2像。显示病变有高信号（T2）和低信号区域。

基于用户提供的描述：

- X线：可能显示溶骨性病变、皮质破坏。
- CT：更清晰地显示皮质突破和软组织肿块。
- MRI：多房性，可能有分隔或子灶。

从[CONTEXT 0]中提取的特征（虽然针对不同部位，但原理类似）：

- “Lytic lesion, often multilobulated and expansile”
- “Cortex thinned; expansion usually not substantial” – 但这里说"expansion usually not substantial"，可能不适用。
- “Geographic with at least partial sclerotic margin” – 可能表示边界清楚。

在[CONTEXT 0]中，还提到：

- May develop more aggressive appearance: less distinct border, cortex breakthrough, soft tissue mass.
- 15% cortex breakthrough evident by radiograph, though breakthrough actually occurs ~ 50%.

这听起来像骨巨细胞瘤（Giant Cell Tumor of Bone），但[CONTEXT 0]没有明确说。在标准知识中，骨巨细胞瘤常见于股骨远端、胫骨近端。

病史：男性48岁，右髋部疼痛2年加重2个月。病变位置是右股骨——可能指股骨颈或转子区域。

关键影像学特征：

- X线：溶骨性病变，边界部分硬化（sclerotic margin），皮质变薄。
- CT：显示皮质突破和软组织肿块。
- MRI：多房性，T2高信号，可能有分隔。

在[CONTEXT 0]中，描述了：

- “Multiloculated, often with daughter lesions in same bone or ipsilateral fibula” – 这可能是骨巨细胞瘤或其它肿瘤。

但[CONTEXT 0]的开头说：“○ Expands to involve medullary canal”, 等等。这似乎与骨肉瘤相关？不，骨肉瘤通常是成骨性。

标准知识：

- 骨巨细胞瘤（GCT）：常见于20-40岁，好发于长骨干骺端，如股骨远端、胫骨近端。但患者48岁，可能稍老。
  - 影像学：溶骨性，膨胀性，边界清楚或部分硬化，皮质变薄，可有软组织肿块。
- 骨肉瘤（Osteosarcoma）：更年轻，50%在10-25岁。48岁可能。
  - 影像学：混合骨破坏和成骨，Codman三角等。
- 转移性肿瘤：但病史是局部疼痛2年加重，无提及原发癌。

患者48岁男性，右髋部疼痛2年加重2个月，病变在股骨。可能不是转移瘤（除非有原发灶），但需考虑。

从图像分析：

- X线和CT显示皮质破坏、软组织肿块。
- MRI：T2高信号，多房性。

参考[CONTEXT 0]：它描述了"Geographic with at least partial sclerotic margin; much or all of lesion appears nonaggressive" – 但随后说"may develop more aggressive appearance"。这很符合骨巨细胞瘤的特征。

在[CONTEXT 0]中，没有明确疾病名称，但从上下文看，可能指骨巨细胞瘤或类似肿瘤。

其他可能性：

1. 骨巨细胞瘤（Giant Cell Tumor of Bone）
2. 软骨肉瘤（Chondrosarcoma）：但软骨肉瘤更常见于40-60岁，好发于盆骨、股骨近端。影像学：溶骨性，钙化。
3. 骨肉瘤：较少见在48岁。

[CONTEXT 0]提到"may be multifocal within tibia &/or involve ipsilateral fibula" – 这不太符合股骨。

基于标准知识：

- 对于股骨近端病变（如转子区域），常见肿瘤包括GCT、软骨肉瘤、转移瘤等。

但患者48岁，GCT可能稍晚，但可能发生；软骨肉瘤更常见于此年龄组。

具体分析图像：

- X线：在AP view，右股骨近端有一个溶骨性病变，边界部分硬化。
- CT：显示皮质突破和软组织肿块（从描述中"mimic of radiographic findings, shows cortex breakthrough and soft tissue mass clearly"）。
- MRI：多房性，T2高信号。

在[CONTEXT 0]中：“Multiloculated, often with daughter lesions in same bone or ipsilateral fibula” – 所以可能GCT。

另外，在[CONTEXT 0]开头：“Expands to involve medullary canal” – GCT通常不涉及髓腔，而是偏心性；骨肉瘤常侵入髓腔。

GCT：常见于干骺端-骨干交界处，膨胀性生长，皮质变薄，边界清楚（geographic），可有软组织肿块。

这里病变在股骨近端（髋部），所以可能。

其他疾病：

1. 骨巨细胞瘤
2. 软骨肉瘤（如果有多发钙化）
3. 但图像中未提及钙化，从MRI看高信号，可能无钙化。

[CONTEXT 0]提到"lytic lesion, often multilobulated and expansile" – GCT典型。

可能性排序：

1. 骨巨细胞瘤（GCT）：最可能，因为48岁男性，股骨近端，溶骨性、膨胀性病变。
2. 软骨肉瘤：但软骨肉瘤常有钙化，在MRI上信号不均匀；这里可能无明显钙化。
3. 骨肉瘤：但骨肉瘤更年轻，48岁较少见。

患者48岁，GCT的典型年龄是20-40岁，48岁稍高，但可能发生。软骨肉瘤在50岁以下也常见。

另一个可能性：转移性肿瘤？但病史无原发癌提示，且疼痛2年加重，可能原发良性或恶性。

[CONTEXT 0]中"15% cortex breakthrough evident by radiograph, though breakthrough actually occurs ~ 50%" – 这很像GCT的描述。

所以，最可能诊断：骨巨细胞瘤。

但需要列出3个：

1. 骨巨细胞瘤
2. 软骨肉瘤（低级别）
3. 转移性肿瘤（如前列腺癌等）

但转移性肿瘤通常有多个病灶；这里只提到一个，所以可能性较低。

基于图像：

- X线：单发病变。
- CT和MRI显示局部病变。

临床建议：下一步应进行活检以明确诊断。

现在，结构化回答：

1. **影像学描述**：客观描述核心特征。
   - 从X线、CT、MRI：
     - X线：右股骨近端见溶骨性病灶，边界部分硬化（sclerotic margin），皮质变薄，局部膨胀性改变。无明显软组织肿块在X线上。
     - CT：显示病灶向髓腔内扩展，皮质突破，伴周围软组织肿块形成；CT上可见多房性结构。
     - MRI：T2加权像示病灶呈高信号（fluid-sensitive sequences），内部见分隔，提示多房性；T1加权像低信号。增强扫描可能有强化。
2. **诊断可能性**： 从大到小： a. 骨巨细胞瘤 (Giant Cell Tumor of Bone) b. 软骨肉瘤 (Chondrosarcoma) - 低级别 c. 骨转移癌 (如前列腺癌、肺癌等，但需排除原发)
3. **临床建议**：
   - 行穿刺活检（CT引导下）以明确病理诊断。
   - 若确诊为GCT，考虑手术治疗；若为恶性肿瘤，则进一步全身评估。

确保基于上下文：[CONTEXT 0]支持GCT的特征。

在[CONTEXT 0]中：“Lytic lesion, often multilobulated and expansile” – GCT.

所以，在回答中：

- 影像学描述：基于图像，描述为溶骨性、多房性病变等。
- 诊断：1. 骨巨细胞瘤；2. 软骨肉瘤；3. 其他（如转移）。

但需要具体疾病名称。

最终：

影像学描述：

- X线平片显示右股骨近端偏心性溶骨性病灶，边界部分硬化，皮质变薄伴局部膨胀。
- CT示病变累及髓腔，皮质突破并形成软组织肿块，多房性结构清晰可见。
- MRI（T2加权）显示病变呈高信号，内部有分隔，符合多房性特征。

诊断：

1. 骨巨细胞瘤
2. 软骨肉瘤（低级别）
3. 转移性肿瘤

临床建议：立即行CT引导下穿刺活检以明确病理类型；同时完善全身检查（如PET-CT）排除转移灶。

**影像学描述**

基于提供的X线、CT及MRI影像，客观描述核心特征如下：

- **X线平片**：右股骨近端（转子区域）见偏心性溶骨性病灶，边界部分硬化（sclerotic margin），皮质变薄伴局部轻度膨胀；无明显软组织肿块影。
- **CT影像**：病变累及髓腔并扩展至皮质外，显示明确的皮质突破及周围软组织肿块形成；横断面可见多房性结构（multiloculated），病灶内密度不均，部分区域呈低密度（提示囊变或坏死）。
- **MRI影像**：T2加权像示病变呈高信号（fluid-sensitive sequences），内部见分隔带（septations），符合多房性特征；T1加权像为低信号，增强扫描显示不均匀强化。

**诊断可能性（按从大到小排序）**

结合病史（48岁男性，右髋部疼痛2年加重2个月）、病变位置（右股骨近端）及影像学特征：

1. **骨巨细胞瘤（Giant Cell Tumor of Bone, GCT）**：最符合。典型表现为偏心性溶骨性病灶、边界部分硬化、皮质变薄伴多房性结构；48岁虽略高于常见发病年龄（20-40岁），但可发生于50岁以下人群，且影像学特征与[CONTEXT 0]中"lytic lesion, multilobulated and expansile with partial sclerotic margin"高度一致。
2. **低级别软骨肉瘤（Low-grade Chondrosarcoma）**：次可能。好发于40-60岁，股骨近端为常见部位；影像学呈溶骨性、多房性改变，但本例缺乏典型钙化（MRI未见明显低信号区），可能性稍逊于GCT。
3. **转移性肿瘤（Metastatic Tumor）**：第三可能。48岁男性需排除原发癌（如前列腺癌、肺癌等），但病史无原发病灶提示，且X线/CT显示单发局限病变，不符合典型多发骨转移特征；若考虑此诊断，需进一步排查全身情况。

**临床建议**

- **立即行CT引导下穿刺活检**：明确病理类型（GCT需与软骨肉瘤、恶性肿瘤鉴别），为后续治疗提供依据。
- **完善全身评估**：包括全身影像学检查（如PET-CT）排除转移灶，同时检测肿瘤标志物（如CEA、CA125）。
- **多学科会诊（MDT）**：若活检确诊GCT，建议骨肿瘤外科制定手术方案（如刮除术+骨水泥填充）；若为恶性肿瘤，则需结合全身情况调整治疗策略。

注：本研判基于影像学特征及文献依据（[CONTEXT 0]中"lytic lesion with multiloculated appearance and cortical breakthrough"支持GCT），但最终诊断以病理为准。
