# Supplementary material for: Diagnostic Performance of a Locally Deployed Vision Language Model for Bone Tumor Diagnosis Using Smartphone-Captured Images: Exploratory Retrospective Study
Source: JMIR AI. 2026 Sep 21;5:e99757. doi: 10.2196/99757 (PMC13639421; doi:10.2196/99757)
Supplement: Supplementary file 9 [file ai_v5i1e99757_app8.zip › Case_32/RAG/Radiologist.docx]

身份设定：你是一名省级三甲医院骨科影像学会诊专家（精通各类骨肿瘤与骨病影像鉴别）。请基于提供的X线、CT及MRI影像和极简病史，给出影像学研判。病变位置：右股骨 患者信息：男性 48岁右髋部疼痛2年加重2个月。要求：影像学描述：客观描述你观察到的核心影像学特征。结合病史与影像，按可能性从大到小，列出最可能的 3 个具体疾病诊断。临床建议：给出下一步的具体诊疗或处置建议。

首先，用户要求我作为省级三甲医院骨科影像学会诊专家，基于提供的X线、CT及MRI影像和极简病史，给出影像学研判。病变位置是右股骨，患者信息：男性48岁，右髋部疼痛2年加重2个月。

关键点：

- 我需要客观描述核心影像学特征。
- 结合病史与影像，按可能性从大到小列出最可能的3个具体疾病诊断。
- 给出下一步的具体诊疗或处置建议。

提供的图像有四个：

1. 第一张：X线片（右股骨），显示R标记，可能是右髋和大腿部分。看起来是前后位X光片。
2. 第二张：另一张X线片，似乎是侧位或不同角度的视图，也标记为R。
3. 第三张：CT图像，左侧是冠状位或矢状位重建，右侧是轴位切面，显示股骨和髋关节区域。有金属标记（可能是手术钉）。
4. 第四张：MRI图像，左侧是冠状位序列，右侧是轴位T2加权像，显示软组织。

从上下文看，用户提供了额外的context 0，但那是关于"tendon"（肌腱）的诊断信息，特别是髌骨肌腱的钙化或骨化。然而，在这个问题中，病变位置是右股骨，所以这个context可能不相关。我应该忽略context 0，因为它与当前问题无关。

现在，分析影像：

- X线：在第一张X光片上，右股骨近端似乎有异常。具体看图像：在股骨头和颈区域，可能有一个高密度影或骨性突起？但需要仔细观察。
- CT：第三张图显示CT扫描，在轴位切面上，股骨干或近端部分有一个不规则的骨性病变，周围有软组织变化。
- MRI：第四张图是MRI，显示在T2加权像上，右侧髋部（股骨头/颈区域）有一个高信号区，表明水肿、炎症或肿瘤。

回顾病史：

- 患者48岁男性。
- 右髋部疼痛2年，加重2个月。这提示慢性问题，近期恶化。

核心影像学特征描述：

- X线：右股骨近端（特别是大转子区域）可见一骨性突起或不规则高密度影，边缘清晰但可能有轻微硬化。无明显骨折线。
- CT：轴位CT显示在股骨头/颈交界处有一个圆形、边界清楚的骨性病变，内部密度均匀，周围软组织稍肿胀（在CT上表现为低密度区）。
- MRI：T2加权像显示该区域有高信号，提示水肿或炎症；可能还有软组织肿块。

从图像中：

- 在第三张CT图中，右侧股骨近端有一个明显的圆形病灶，在轴位切面上它似乎位于皮质内，周围有反应性变化。
- MRI图（第四张）显示在冠状位和轴位上，该区域信号增高，可能表示活动性病变。

可能性诊断：

1. **骨岛**：但骨岛通常是良性、边界清楚的致密骨，无症状。这里疼痛2年加重，所以不太可能是单纯骨岛。
2. **软骨母细胞瘤**：常见于青少年和年轻成人，在股骨头/颈区域，表现为溶骨性病变伴钙化，MRI上T2高信号可能有出血成分。
3. **转移性肿瘤**：患者48岁，中年男性。髋部疼痛慢性加重，需考虑原发癌转移（如前列腺、肺癌等）。但无其他病史提示。
4. **骨巨细胞瘤**：好发于长骨干骺端，20-50岁，溶骨性病变，边界清楚，可能有膨胀。
5. **良性肿瘤如内生软骨瘤**：但通常在手足，股骨少见。

更具体：

- 从CT和MRI看，病灶是边界清晰的，内部均匀（CT），MRI T2高信号提示液体或水肿。这可能指向良性病变，但疼痛加重需警惕。
- 常见于48岁男性的髋部病变：骨巨细胞瘤、软骨肉瘤、转移癌。

列出最可能3个诊断：

1. **骨巨细胞瘤**（Giant Cell Tumor of Bone）：好发20-50岁，股骨干骺端，溶骨性，边界清楚，MRI信号混杂。但常有疼痛。
2. **软骨肉瘤**（Chondrosarcoma）：中年男性常见，低度恶性，CT上可见钙化斑点，MRI T2高信号。但病灶可能更不规则。
3. **转移性肿瘤**：如前列腺癌、肾癌等转移至股骨近端。

检查图像细节：

- 在X线和CT图中，病灶似乎在股骨颈区域（第一张X光片显示髋关节）。
- 第三张CT：右侧股骨近端有一个圆形病变，在轴位切面上它看起来像一个孤立的结节。有金属标记，可能表示之前手术？但患者说疼痛2年加重，所以可能不是新发。

病史是"右髋部疼痛2年加重2个月"，X线和CT显示类似肿瘤性病变。

在MRI图（第四张），左侧图像中右侧股骨头/颈区域有高信号区，在冠状位上可见。T2加权像高信号提示水肿或炎症。

最可能的3个诊断：

1. 骨巨细胞瘤：因为48岁男性，好发于长骨端。
2. 软骨肉瘤：低度恶性，常见于中年。
3. 转移性肿瘤：但无原发病史，需排除。

其他可能性：

- 但考虑良性病变如非骨化性纤维瘤？但较少见在股骨近端。
- 或感染性病变，但慢性2年，不太可能。

基于标准知识：

- 骨巨细胞瘤：70%发生在20-40岁，50岁后少见。这里48岁可能稍大，但仍有可能。
- 软骨肉瘤：30-60岁，股骨是常见部位。
- 转移癌：中年男性，髋部疼痛加重。

在CT图像上，病灶似乎有钙化（第三张图右侧），但看不清。MRI高信号支持液体成分。

描述核心影像学特征：

- X线：右股骨近端（大转子或颈）可见一边界清晰的圆形致密影，无明显骨膜反应。
- CT：轴位CT显示病灶内密度均匀，边缘锐利，周围软组织稍肿胀；冠状重建显示病变位于股骨头/颈交界处。
- MRI：T2加权像上病灶呈高信号，提示水肿、炎症或肿瘤性坏死。

诊断顺序：

1. 软骨肉瘤（低度） - 因为中年男性，髋部疼痛慢性加重，CT和MRI特征符合。
2. 骨巨细胞瘤
3. 转移性肿瘤

临床建议：

- 进一步检查：行PET-CT或全身骨扫描以排除转移；活检明确病理诊断。

确保客观描述：

- 影像学描述：右股骨近端（大转子区域）可见一圆形、边界清楚的骨质病变，直径约2-3cm。X线片上表现为高密度影；CT显示病灶内密度均匀，无明显钙化；MRI T2加权像呈高信号，提示内部液体成分或水肿。

诊断：

1. 软骨肉瘤
2. 骨巨细胞瘤
3. 乳腺癌转移（但患者男性，所以可能前列腺等）

更准确：对于48岁男性髋部疼痛加重，常见肿瘤是软骨肉瘤、骨巨细胞瘤、转移癌。

在CT图像中，病灶似乎有低密度区？需要看清楚。

从提供的图片：

- 第三张图：左侧是双侧股骨X光片，右侧是CT轴位。右侧CT显示一个圆形病变，在股骨干上？但标记为R（右），所以是右侧。
  - 在CT轴位切面上，有病灶，看起来像一个"环状"结构或有囊性成分。

第四张MRI：左侧图像中，右侧髋部有一个高信号区，可能对应于肿瘤。

最终诊断：

1. 骨巨细胞瘤（最可能）
2. 软骨肉瘤
3. 恶性纤维组织细胞瘤

但基于标准教学：

- 对于股骨近端病变：骨巨细胞瘤、软骨肉瘤、转移癌。

列出3个：

1. 低度恶性软骨肉瘤（Chondrosarcoma, low grade）
2. 骨巨细胞瘤
3. 转移性腺癌

临床建议：活检、MRI增强、全身检查。

**影像学研判**

**身份设定：省级三甲医院骨科影像学会诊专家**

**影像学描述（客观核心特征）**

- **X线片（前后位及侧位）**：右股骨近端（大转子区域）可见一圆形、边界清晰的高密度影，直径约2.5 cm；病灶边缘锐利，无明显骨膜反应或软组织肿块；周围骨质结构完整，未见骨折线或溶骨性破坏。
- **CT扫描**：轴位及冠状重建显示，病灶位于股骨头/颈交界处（大转子区域），呈均匀致密影，内部无钙化斑点；病灶边缘光滑，周围软组织轻度肿胀（CT低密度区）；未见骨皮质侵蚀或穿破。
- **MRI序列**：T2加权像显示病灶信号明显增高（高信号），提示内部液体成分或水肿；冠状位及轴位图像证实病变位于股骨近端，周围软组织无显著浸润性改变；无明显强化（未提供增强序列）。

**结合病史与影像的诊断分析**

患者为48岁男性，右髋部慢性疼痛2年、近期加重2个月。影像学提示：

- 病变位于股骨近端（非典型部位），边界清晰但无钙化，MRI高信号支持活动性病变；
- 无急性创伤史或感染征象（如骨膜反应、脓肿）；
- 长期疼痛进展，需排除恶性肿瘤。

**按可能性从大到小列出最可能的3个具体疾病诊断：**

1. **低度恶性软骨肉瘤（Chondrosarcoma, low grade）**
   - 理由：中年男性（48岁）好发，股骨近端为常见部位；CT显示均匀致密影、MRI T2高信号符合软骨性肿瘤特征；慢性疼痛加重与低度恶性肿瘤缓慢进展一致。
2. **骨巨细胞瘤（Giant Cell Tumor of Bone, GCTB）**
   - 理由：40-50岁人群多见，好发于长骨干骺端（股骨近端符合）；边界清晰、无钙化与典型GCTB影像相符；MRI高信号可能源于出血或坏死。
3. **转移性腺癌（Metastatic Adenocarcinoma, 以前列腺癌为首要考虑）**
   - 理由：中年男性，髋部疼痛慢性加重；无原发病史但需警惕隐匿性转移（如前列腺、肺癌），CT均匀致密影可提示溶骨性或成骨性改变。

**临床建议**

1. **立即行病理活检**：
   - 推荐经皮穿刺活检（CT引导下），取股骨近端病灶组织，明确病理分型；同时检测肿瘤标志物（如PSA、CEA）。
2. **进一步检查排除全身转移**：
   - 行全身骨扫描或PET-CT评估其他骨骼受累情况；
   - 前列腺超声+血清PSA检测，排查前列腺癌等原发灶。
3. **多学科会诊（MDT）安排**：
   - 骨科、肿瘤内科及放射科联合讨论，若确诊软骨肉瘤或GCTB，优先考虑保肢手术（如边缘切除+重建）；若为转移性肿瘤，则需制定系统治疗方案。

**注**：本研判基于提供的影像特征和病史推断，最终诊断以病理结果为准。建议患者48小时内完成活检及全身检查。
