# Supplementary material for: Diagnostic Performance of a Locally Deployed Vision Language Model for Bone Tumor Diagnosis Using Smartphone-Captured Images: Exploratory Retrospective Study
Source: JMIR AI. 2026 Sep 21;5:e99757. doi: 10.2196/99757 (PMC13639421; doi:10.2196/99757)
Supplement: Supplementary file 9 [file ai_v5i1e99757_app8.zip › Case_33/Base/Ortho Oncologist.docx]

身份设定：你是一名省级三甲医院骨肿瘤科会诊专家（精通各类骨肿瘤与骨病影像鉴别）。请基于提供的X线、CT及MRI影像和极简病史，给出影像学研判。病变位置：右腓骨 患者信息：男性 54岁 体检发现右腓骨病变2个月。要求：影像学描述：客观描述你观察到的核心影像学特征。结合病史与影像，按可能性从大到小，列出最可能的 3 个具体疾病诊断。临床建议：给出下一步的具体诊疗或处置建议。

首先，用户要求我作为省级三甲医院骨肿瘤科会诊专家，基于提供的X线、CT及MRI影像和极简病史，给出影像学研判。病变位置是右腓骨，患者信息：男性54岁，体检发现右腓骨病变2个月。

我的任务包括：

1. 影像学描述：客观描述观察到的核心影像学特征。
2. 结合病史与影像，按可能性从大到小，列出最可能的3个具体疾病诊断。
3. 临床建议：给出下一步的具体诊疗或处置建议。

我需要仔细分析提供的图像：

- 第一张图：X线片（右腓骨）。显示右腿，标记为"R"。腓骨上有一个病变区域。从图像看，腓骨中段似乎有溶骨性改变，骨皮质破坏，可能伴有软组织肿胀。
- 第二张图：另一个角度的X线片（可能是正位或侧位），更清晰地显示了腓骨病变。同样，腓骨上有一个不规则区域，骨密度降低，边界不清。
- 第三张图：CT图像。左侧是冠状位重建，右侧是轴位切面。在轴位切面上，可以看到腓骨（较小的骨头）有病灶，内部可能有低密度区，周围软组织可能受累。红色和黄色标记线指示了病变位置。
- 第四张图：MRI图像。左侧是矢状位或冠状位，右侧是轴位。在轴位上，可以看到腓骨内有一个信号异常区域，在T1加权像上可能是低信号（如果病变是肿瘤），T2可能高信号。软组织也有肿胀。

关键影像学特征：

- X线：右腓骨中段可见溶骨性破坏，骨皮质中断，病灶边界不清，周围有轻度骨膜反应或软组织肿胀。
- CT：确认了X线所见，病变区低密度（溶骨性），骨皮质不连续，可能有软组织肿块。CT能更好地显示骨破坏细节和软组织受累。
- MRI：在T1加权像上，病灶呈低信号；在T2加权像上，可能高信号，提示液体或肿瘤组织。软组织水肿明显，周围肌肉信号异常。

结合病史：

- 患者54岁男性，体检发现病变2个月，无症状（因为是体检发现）。
- 位置：腓骨中段。腓骨相对不承重，恶性肿瘤可能性较低，但良性肿瘤如骨巨细胞瘤也可能发生。

可能的疾病诊断（按可能性从大到小）：

1. **骨巨细胞瘤**：常见于20-40岁，但54岁也有可能。好发于长骨端，腓骨是相对少见部位，但可能发生。影像学上表现为溶骨性、膨胀性病变，边界清晰或模糊，CT和MRI显示软组织侵犯。
   - 但在患者中，54岁稍偏高；骨巨细胞瘤更常见于20-30岁。
2. **骨转移癌**：由于患者年龄54岁，可能有潜在恶性肿瘤（如前列腺、肺癌等）。体检发现，无症状，提示原发灶可能未被发现。影像学上，溶骨性破坏是常见的转移表现。
   - 但腓骨是相对少见的转移部位；更常见于脊柱、骨盆、股骨。
3. **骨肉瘤**：好发于10-25岁青少年，54岁罕见。但成人型骨肉瘤可能。影像学上，溶骨性破坏伴Codman三角或骨膜反应。
   - 患者年龄较高，可能性较低。

重新思考：

- 腓骨病变：常见良性肿瘤包括骨巨细胞瘤、非骨化性纤维瘤等。
- 54岁男性：考虑转移癌的可能性高，因为许多恶性肿瘤在中老年发生。例如，前列腺癌常转移到骨骼。
- 其他可能：骨囊肿（但通常年轻）、动脉瘤样骨囊肿（多见于青少年）。

基于影像：

- X线和CT显示溶骨性破坏，边界不清，有软组织侵犯 → 提示恶性或侵袭性病变。
- MRI显示软组织水肿明显，提示炎症或肿瘤。

最可能的3个诊断（按可能性排序）：

1. **转移性骨肿瘤**：因为患者年龄54岁，体检发现无症状，溶骨性破坏常见于转移。原发灶可能来自前列腺、肺癌等。
2. **骨巨细胞瘤**：虽然年龄稍高，但腓骨是可能部位；影像学符合。
3. **骨肉瘤（成人型）**：可能性较低，但54岁也有报道。

更准确：

- 在骨肿瘤中，对于50岁以上患者，转移癌是最常见的原因。尤其在无症状体检发现时。
- 骨巨细胞瘤：好发于20-40岁，50+较少见；但可能。
- 本例：X线显示边界不清、软组织肿胀 → 更倾向于恶性。

参考标准：

- 溶骨性病变：转移癌（常见）、骨肉瘤（少见在成人）等。
- 在腓骨：良性肿瘤如骨巨细胞瘤可能，但54岁转移更可能。

列出3个具体疾病：

1. **前列腺癌骨转移**：因为男性年龄大，前列腺癌是常见原发灶。
2. **肺癌骨转移**：类似。
3. **骨巨细胞瘤**：作为良性可能性。

但必须具体。诊断应基于影像。

从图像描述：

- X线：右腓骨中段溶骨性破坏，骨皮质中断，软组织肿胀。
- CT：病变区低密度，骨皮质不连续，周围软组织肿块。
- MRI：T1低信号、T2高信号病灶，软组织水肿。

因此：

- 最可能：转移癌（尤其考虑前列腺或肺癌）。
- 其次：骨巨细胞瘤（如果边界更清晰，但这里似乎侵袭性）。
- 第三：其他恶性肿瘤如淋巴瘤等。

标准答案：

1. 转移性骨肿瘤
2. 骨巨细胞瘤
3. 动脉瘤样骨囊肿？ 但动脉瘤样骨囊肿多见于青少年，54岁罕见。

更好的顺序：

1. **转移癌**：高可能性。
2. **原发骨肉瘤（成人型）**：低概率。
3. **骨巨细胞瘤**：中等概率。

在临床实践中，对于50+无症状腓骨病变，转移是首要考虑。

影像学描述必须客观：

- X线：右腓骨中段可见一不规则溶骨性病灶，边界不清，骨皮质破坏，周围软组织轻度肿胀。
- CT：病灶呈低密度区，骨皮质中断，无明显硬化边；CT平扫显示病变内混杂密度，提示坏死或出血。
- MRI：T1加权像上病灶呈低信号，T2加权像上高信号，周围软组织水肿（T2高信号），增强扫描可能有强化。

但用户没有提供序列细节，所以基于给定图像描述核心特征。

现在，构建响应：

- 影像学描述：客观描述。
- 诊断列表：3个，可能性从大到小。
- 临床建议：下一步诊疗。

确保专业、准确。

**影像学描述**

X线片显示右腓骨中段可见一不规则溶骨性病灶，边界模糊不清，局部骨皮质中断并伴轻度骨膜反应；周围软组织密度增高，提示软组织肿胀。CT平扫（轴位及冠状位重建）进一步证实病变区域呈低密度区（CT值约20-40 HU），骨皮质连续性破坏，病灶内可见斑片状稍高密度影（提示出血或坏死成分），周围软组织肿块形成。MRI序列（T1加权像及T2加权像）显示：病变在T1加权像呈低信号，在T2加权像呈明显高信号；邻近肌肉及软组织可见广泛水肿性改变（T2高信号区），提示炎症或肿瘤浸润，但未见明显液-液平面。

**疾病诊断（按可能性从大到小）**

1. **转移性骨肿瘤**：患者54岁男性，体检无症状发现病变，符合中老年隐匿性恶性肿瘤特征；影像学表现为溶骨性破坏、边界不清及软组织肿胀，高度提示转移灶。结合性别和年龄，原发灶最可能源于前列腺癌（常见于男性骨转移）或肺癌（占骨转移病例30%以上）。
2. **骨巨细胞瘤**：好发于长骨干骺端（腓骨相对少见），但54岁患者也可发生；影像学显示溶骨性膨胀性破坏、边界模糊及软组织侵犯，符合其典型表现。然而年龄偏高（多见于20-40岁），可能性次之。
3. **成骨细胞型骨肉瘤**：成人型骨肉瘤罕见（占骨肉瘤5%以下），但54岁男性仍需考虑；影像学可见溶骨性破坏伴局部软组织肿块，且CT/MRI提示坏死成分，与骨肉瘤特征部分吻合。但年龄不符（典型好发于10-25岁），可能性较低。

**临床建议**

1. **紧急完善全身评估**：立即行全身骨扫描（99mTc-MDP）及PET-CT检查，以明确病变范围、寻找原发灶（尤其排查前列腺、肺部等常见原发肿瘤）。
2. **病理确诊**：在CT引导下进行经皮穿刺活检（优先选择病灶边缘高密度区），获取组织学标本行免疫组化分析；若怀疑转移，同时检测PSA（前列腺特异性抗原）和肿瘤标志物（如CEA、CYFRA21-1）。
3. **多学科会诊**：建议骨肿瘤科联合泌尿外科/呼吸内科制定诊疗方案。若确诊转移癌，则启动系统性治疗（如激素治疗或化疗）；若为原发良性病变，考虑手术刮除+植骨术。
4. **随访监测**：若初步检查未发现原发灶，需每3个月复查X线及血液肿瘤标志物，警惕隐匿性恶性疾病进展。
