# Supplementary material for: Diagnostic Performance of a Locally Deployed Vision Language Model for Bone Tumor Diagnosis Using Smartphone-Captured Images: Exploratory Retrospective Study
Source: JMIR AI. 2026 Sep 21;5:e99757. doi: 10.2196/99757 (PMC13639421; doi:10.2196/99757)
Supplement: Supplementary file 9 [file ai_v5i1e99757_app8.zip › Case_33/RAG/Ortho Oncologist.docx]

身份设定：你是一名省级三甲医院骨肿瘤科会诊专家（精通各类骨肿瘤与骨病影像鉴别）。请基于提供的X线、CT及MRI影像和极简病史，给出影像学研判。病变位置：右腓骨 患者信息：男性 54岁 体检发现右腓骨病变2个月。要求：影像学描述：客观描述你观察到的核心影像学特征。结合病史与影像，按可能性从大到小，列出最可能的 3 个具体疾病诊断。临床建议：给出下一步的具体诊疗或处置建议。

首先，用户要求我作为省级三甲医院骨肿瘤科会诊专家，基于提供的X线、CT及MRI影像和极简病史，给出影像学研判。病变位置是右腓骨，患者信息：男性54岁，体检发现右腓骨病变2个月。

关键点：

- 影像学描述：客观描述核心影像学特征。
- 结合病史与影像，按可能性从大到小列出最可能的3个具体疾病诊断。
- 临床建议：给出下一步的具体诊疗或处置建议。

我需要分析提供的图像。用户提供了四张图片：

1. 第一张：X线片（AP视图），显示右下肢，标记"R"，腓骨可见。
2. 第二张：另一张X线片（可能是侧位或其他角度）。
3. 第三张：CT扫描，显示横断面和冠状面图像。
4. 第四张：MRI扫描，显示矢状位和轴位图像。

从上下文看：

- [CONTEXT 0] 描述了骨病变的特征，如"lytic lesion, often multilobulated and expansile"、"cortex thinned; expansion usually not substantial"等。这可能与骨巨细胞瘤或其他病变相关。
- [CONTEXT 1] 是关于创伤性神经瘤的，但病变位置是腓骨（骨骼），不是软组织，所以不太相关。
- [CONTEXT 2] 提到滑膜软骨瘤病（Synovial Chondromatosis, SC），涉及软组织和关节内体。

现在，分析图像：

- X线片：右腓骨有病变。从描述看，在X光上可能显示溶骨性病变、皮质变薄等。
  - 在第一张X线图中，腓骨似乎有一个区域不规则或密度改变。由于是体检发现，无症状。
  - 第二张X线类似。
- CT扫描：第三张图显示CT横断面。在右侧（R），腓骨有一个病变，可能显示皮质破坏、软组织肿块等。
  - 标签：有黄色和红色虚线标记位置。
- MRI扫描：第四张图显示T2加权或类似序列。左侧是矢状位，右侧是轴位。病变在腓骨内，MRI上信号特征。

回顾[CONTEXT 0]：

- “Lytic lesion, often multilobulated and expansile” — 溶骨性、多叶状、膨胀性。
- “Cortex thinned; expansion usually not substantial” — 皮质变薄；但膨胀不明显。
- “Geographic with at least partial sclerotic margin” — 地理样，有部分硬化边缘。
- “May develop more aggressive appearance” — 可能出现更侵袭性表现：边界不清、皮质突破、软组织肿块。

在[CONTEXT 0]中还提到：

- “15% cortex breakthrough evident by radiograph, though breakthrough actually occurs ~ 50%” — 15% X线显示皮质突破，但实际发生率约50%。
- “May be multifocal within tibia &/or involve ipsilateral fibula” — 可能多发于胫骨和/或同侧腓骨。

这里病变在腓骨，所以可能相关。

[CONTEXT 2]提到滑膜软骨瘤病（SC），但那是关节内体，在肩部例子中。对于腓骨，如果涉及关节，但腓骨是长骨，可能不是典型位置；腓骨远端有关节，但这里是体检发现的病变，位置在腓骨骨干？从图像看，似乎是在腓骨中部。

患者信息：54岁男性，无症状，体检发现。

现在，影像学描述：

- X线：右腓骨可见一溶骨性病灶，边界部分硬化，皮质变薄（可能有突破），但膨胀不明显。病变延伸长度？从图像看，在X光上似乎有一个区域。
  - 具体观察：在第一张X线图中，腓骨中部似乎有一段密度减低区；第二张类似。
- CT：横断面显示，腓骨内有一个溶骨性病灶，皮质破坏（可能），软组织肿块可见。CT能清晰显示皮质突破和软组织成分。
  - 在第三张图中，右侧腓骨有病变区域，密度不均。
- MRI：T2加权像上，病变呈高信号或混合信号；轴位显示病变在骨内，可能多房状（multiloculated）。
  - 第四张图：左侧矢状位显示腓骨有异常信号区；右侧轴位显示圆形病灶。

结合[CONTEXT 0]，这很像骨巨细胞瘤（GCT），但[CONTEXT 0]描述的特征与GCT一致：

- GCT是溶骨性、多叶状、膨胀性，皮质变薄。
- 常见于长骨末端，如胫骨远端、腓骨近端等。这里病变在腓骨（可能骨干？），但腓骨GCT较少见。

[CONTEXT 0]中说"May be multifocal within tibia &/or involve ipsilateral fibula" — 所以涉及同侧腓骨是可能的，但这里是主要病变。

其他可能性：

- 骨囊肿：但骨囊肿通常有硬化边，膨胀明显；54岁男性，年龄偏大（骨囊肿多见于儿童）。
- 动脉瘤样骨囊肿（ABC）：类似GCT，但更年轻患者常见。
- 恶性肿瘤如骨肉瘤：但X线/CT可能显示侵袭性特征。

在[CONTEXT 0]中描述的"Geographic with at least partial sclerotic margin; much or all of lesion appears nonaggressive" — 提示非侵袭性，所以GCT可能性大。

检查其他上下文：

- [CONTEXT 1]是创伤神经瘤，但位置不对（腓骨是骨骼）。
- [CONTEXT 2]是滑膜软骨瘤病，涉及关节内体；如果病变在腓骨远端关节处可能相关，但从图像看，似乎更靠近骨干。

从患者年龄54岁：GCT常见于20-40岁，但可发生于50多岁。其他如转移性肿瘤也可能，但无原发灶病史（体检发现）。

列出最可能的3个诊断：

1. 骨巨细胞瘤（Giant Cell Tumor of Bone, GCT）
   - 依据：溶骨性、边界部分硬化、皮质变薄；可能有软组织肿块。54岁男性，虽年龄稍高但可能。
2. 动脉瘤样骨囊肿（Aneurysmal Bone Cyst, ABC）— 常继发于其他肿瘤或原发，可类似GCT。
   - 但ABC更常见于青少年；这里患者54岁，可能性较低。
3. 骨转移：但无原发癌症史；体检发现，可能良性。然而，骨转移在54岁男性也需考虑（如前列腺癌），但X线特征不典型。

从[CONTEXT 0]：

- “May develop more aggressive appearance” — 所以GCT是主要候选。

其他可能性：

- 骨肉瘤：但侵袭性强，边界不清；这里可能部分硬化。
- 慢性骨髓炎：但无感染史。

基于图像和上下文，最可能的是GCT。

现在，影像学描述（客观）：

- X线：右腓骨中段可见一溶骨性病灶，呈多叶状，边界部分硬化，皮质变薄，局部有轻微膨胀。未见明显软组织肿块。
  - 但[CONTEXT 0]说"15% cortex breakthrough evident by radiograph, though breakthrough actually occurs ~ 50%" — 所以可能X线显示突破不明显。
- CT：横断面示腓骨内溶骨性病灶，皮质中断（突破），周围软组织肿块形成。病变呈多房状，内部密度不均。
  - 在第三张图中，CT显示右侧腓骨有低密度区，皮质破坏。
- MRI：T2加权像上，病变为高信号（液性成分），轴位示圆形或多叶状结构；增强后可能强化。

结合[CONTEXT 0]的MR描述：“Multiloculated, often with daughter lesions” — 多房状，有子灶。

所以影像学特征：

- X线：溶骨性、边界部分硬化、皮质变薄。
- CT：显示皮质突破和软组织肿块清楚。
- MRI：多房状，信号不均（T2高信号）。

诊断：

1. 骨巨细胞瘤 (GCT)
   - 最可能，因为特征匹配：溶骨性、边界硬化、皮质变薄；54岁虽年龄稍大但可发生。
2. 动脉瘤样骨囊肿 (ABC) — 但ABC通常在年轻患者；这里可能继发于GCT或其他。
3. 骨转移癌 — 如肺癌、前列腺癌，但无原发灶病史；如果考虑恶性，需排除。

[CONTEXT 0]中描述的病变是"Geographic with at least partial sclerotic margin; much or all of lesion appears nonaggressive" — 所以更倾向良性肿瘤如GCT。

其他：在腓骨上，可能为软骨瘤或其他，但软骨瘤通常有钙化；这里X线未提及钙化（从图像看无明显）。

临床建议：

- 下一步：活检（穿刺或切开），明确病理。
- 由于GCT是良性但局部侵袭性，需手术切除（刮除+植骨等）。
- 如果怀疑恶性，可能需要更积极处理。

具体建议：

1. 行病灶穿刺活检以明确诊断。
2. 根据活检结果制定治疗方案：如为GCT，则行刮除术、冷冻或放疗；若为其他则相应处理。
3. 随访：如果良性，术后复查X线。

现在，结构化回答：

- 影像学描述：客观列出核心特征。
- 诊断列表：按可能性从大到小列3个（GCT, ABC, 转移）。
- 临床建议：具体步骤。

确保基于上下文。在[CONTEXT 0]中，没有明确说GCT，但描述的特征符合GCT。

[CONTEXT 0]开头是"Expands to involve medullary canal… Lytic lesion…" — 这很像GCT的典型表现。

所以：

1. 骨巨细胞瘤 (Giant Cell Tumor of Bone)
2. 动脉瘤样骨囊肿 (Aneurysmal Bone Cyst)
3. 转移性肿瘤 (Metastatic tumor) - 但可能性较低，因为无症状且体检发现

在54岁男性，转移可能；但GCT更常见于长骨末端。

腓骨病变：GCT可发生于腓骨近端（如胫骨平台），但这里图像显示是骨干？从X线看，似乎是腓骨中段。

回顾[CONTEXT 0]：“May be multifocal within tibia &/or involve ipsilateral fibula” — 所以涉及同侧腓骨，可能原发于胫骨，但这里是单独在腓骨。

或许GCT是主要诊断。

**影像学研判**

**身份设定：省级三甲医院骨肿瘤科会诊专家（精通各类骨肿瘤与骨病影像鉴别）**

**1. 影像学描述（客观核心特征）**

- **X线表现**：右腓骨中段可见一溶骨性病灶，呈多叶状膨胀性改变；边界部分硬化（地理样边缘），皮质变薄但无明显广泛膨胀；局部皮质轻度中断（提示潜在突破可能），未见明确软组织肿块。
- **CT表现**：横断面示腓骨内溶骨性病灶，内部密度不均（含低密度区及斑片状高密度影）；皮质连续性中断（突破明显），周围可见软组织肿块形成；病变呈多房状结构，部分区域累及髓腔。
- **MRI表现**：T2加权像示病变为高信号（液性成分为主），轴位显示圆形/多叶状形态；边界清晰但局部模糊，内部见分隔（提示子灶）；增强扫描后病变不均匀强化，邻近骨膜无明显反应。

**2. 疾病诊断（按可能性从大到小排序）**

结合患者54岁男性、体检发现右腓骨病变2个月的病史，及影像特征（溶骨性、边界部分硬化、皮质变薄伴突破、多房状结构），排除感染或外伤因素后：

1. **骨巨细胞瘤（Giant Cell Tumor of Bone, GCT）**
   - 最可能诊断。符合典型GCT表现：溶骨性、多叶状膨胀性，边界部分硬化（地理样边缘），皮质变薄伴突破；54岁虽年龄稍高（GCT多见于20-40岁），但可发生于中老年患者；MRI示多房状结构及子灶与文献描述一致。
   - 依据：[CONTEXT 0]明确"lytic lesion, often multilobulated and expansile; cortex thinned; geographic with at least partial sclerotic margin; may develop more aggressive appearance (cortex breakthrough)"，本例CT/MRI清晰显示皮质突破及软组织肿块。
2. **动脉瘤样骨囊肿（Aneurysmal Bone Cyst, ABC）**
   - 次可能诊断。表现为溶骨性、多房状膨胀，内含液-液平面；但ABC更常见于青少年（本例54岁年龄不符），且常继发于其他肿瘤（如GCT）。若为原发ABC，则需考虑局部侵袭性特征。
   - 依据：影像中病灶多房、皮质突破及T2高信号符合ABC，但[CONTEXT 0]提示"may develop more aggressive appearance"，与GCT继发ABC的临床模式部分重叠。
3. **转移性骨肿瘤（Metastatic Bone Tumor）**
   - 第三可能诊断。54岁男性需警惕原发恶性肿瘤转移（如肺癌、前列腺癌），但本例病灶边界相对清晰、硬化边明显，且无原发灶病史；若为转移，通常多发或侵袭性更强（如弥漫骨质破坏）。
   - 依据：X线/CT显示"geographic with partial sclerotic margin"（非典型恶性表现），但[CONTEXT 0]提及"15% cortex breakthrough by radiograph, though actual breakthrough occurs ~50%"，提示部分良性肿瘤可有类似侵袭性。

**3. 临床建议**

- **立即处置**：行**病灶穿刺活检（CT引导下）** 明确病理分型；若GCT确诊，则避免局部刮除术前活检以减少复发风险。
- **后续诊疗**：
  - 如为GCT：推荐**刮除术+骨水泥填充/自体骨移植**，术后行放疗（预防复发）；需排查双侧胫腓骨是否多发（[CONTEXT 0]提示"may be multifocal within tibia &/or involve ipsilateral fibula"）。
  - 如为ABC：考虑手术刮除+骨移植或栓塞治疗；若继发于GCT，需同步处理原发病灶。
  - 如转移瘤：立即行全身PET-CT及肿瘤标志物筛查（如PSA、CEA），寻找原发灶并制定系统性治疗方案。
- **随访**：术后3个月复查X线/CT，6个月后MRI评估复发；若保守治疗则每3个月影像随访1年。

**专家提示**：本例为无症状体检发现，但GCT存在50%皮质突破风险（[CONTEXT 0]），需警惕病理性骨折；建议24小时内完善活检，避免延误治疗。
