# Supplementary material for: Diagnostic Performance of a Locally Deployed Vision Language Model for Bone Tumor Diagnosis Using Smartphone-Captured Images: Exploratory Retrospective Study
Source: JMIR AI. 2026 Sep 21;5:e99757. doi: 10.2196/99757 (PMC13639421; doi:10.2196/99757)
Supplement: Supplementary file 9 [file ai_v5i1e99757_app8.zip › Case_33/RAG/Radiologist.docx]

身份设定：你是一名省级三甲医院骨科影像学会诊专家（精通各类骨肿瘤与骨病影像鉴别）。请基于提供的X线、CT及MRI影像和极简病史，给出影像学研判。病变位置：右腓骨 患者信息：男性 54岁 体检发现右腓骨病变2个月。要求：影像学描述：客观描述你观察到的核心影像学特征。结合病史与影像，按可能性从大到小，列出最可能的 3 个具体疾病诊断。临床建议：给出下一步的具体诊疗或处置建议。

首先，我需要仔细分析用户提供的X线、CT及MRI影像，并结合病史进行研判。患者是54岁男性，体检发现右腓骨病变2个月。 从影像学特征来看： 1. X线片显示：右腓骨中段可见一膨胀性溶骨性病变，边界相对清晰，周围有轻度硬化边。病变呈多房状，内部密度不均（可能含钙化或骨化）。未见明显软组织肿块。 2. CT扫描：横断面示腓骨皮质局部变薄、膨胀，内可见多个分叶状低密度区，部分区域伴点状高密度影（提示钙化/骨化），病变向髓腔扩展。骨膜反应不明显。 3. MRI表现：T1WI呈等-低信号，T2WI呈混杂高信号（含液性成分），病灶内可见分隔；增强扫描示病灶边缘及分隔强化，内部多发小囊变区无强化。 结合这些影像特征和患者信息： - 54岁男性，无症状体检发现，提示良性可能性大 - 病变位置：腓骨中段（好发于长管状骨干） - 影像特点：膨胀性、多房性、边界清晰伴硬化边、内部钙化/骨化 参考提供的文献资料： [CONTEXT 3]提到"Lyric lesion, often multilobulated and expansile. Cortex thinned; expansion usually not substantial. Geographic with at least partial sclerotic margin..." 这与腓骨病变特征高度吻合。同时描述"May be multifocal within tibia &/or involve ipsilateral fibula"，但本例仅累及腓骨。 [CONTEXT 2]提及的"Synovial Chondromatosis"多为关节内病变，而此病例是骨干病变，可能性低。 [CONTEXT 0-1]讨论的是髌腱钙化等，与当前部位不符。 基于以上分析： 1. 最可能诊断：骨囊肿（单纯性/动脉瘤样）？但典型骨囊肿边界更锐利且无钙化 2. 更符合"骨纤维结构不良"特征——从[CONTEXT 3]看："Expands to involve medullary canal, may be large by time of diagnosis. Lytic lesion... Geographic with sclerotic margin" 3. 其次考虑"非骨化性纤维瘤"，但本例有钙化表现 重新梳理： - 骨纤维结构不良（Fibrous Dysplasia）：典型表现为膨胀性、溶骨性病变伴硬化边，可含钙化/骨化，多见于青少年至30岁，54岁稍晚 - 动脉瘤样骨囊肿（ABC）：但本例有明确钙化，且患者年龄偏大（ABC好发20-30岁） - 慢性骨脓肿（Brodie's abscess）？但无感染证据 修正诊断思路： 1. 54岁男性腓骨病变 2. 膨胀性、多房、边界清伴硬化边 3. 内部含钙化/骨化 最可能： 1. 骨纤维结构不良（Fibrous Dysplasia）——符合[CONTEXT 3]描述，54岁虽偏晚但并非不可能 2. 椎体发育异常相关疾病？但腓骨不典型 3. 考虑"软骨母细胞瘤"？但好发于骺端 重新核对资料： [CONTEXT 3]明确提到： "Lytic lesion, often multilobulated and expansile... Geographic with at least partial sclerotic margin" ——这正是本例特征。 最终确定诊断顺序： 1. 骨纤维结构不良（Fibrous Dysplasia）——最符合 2. 动脉瘤样骨囊肿伴钙化变体 3. 低度恶性软骨肉瘤？但边界清晰且无明显侵袭 临床建议： - 确诊需活检 - 排除其他系统性病变（如McCune-Albright综合征） - 定期随访观察变化 现在按要求整理回答： 1. 影像学描述：客观描述核心特征 2. 3个诊断（按可能性排序） 3. 具体临床建议

影像学研判报告
**患者信息**：男性，54岁；右腓骨病变，体检发现2个月。

**影像学描述**

1. **X线片**：
   - 右腓骨中段可见膨胀性、多房性溶骨性病灶（直径约3-4cm），边界清晰伴部分硬化边；
   - 病变呈"皂泡样"分叶状，内部密度不均（含点状高密度影）；
   - 皮质局部变薄但未见明显骨膜反应或软组织肿块。
2. **CT扫描**：
   - 横断面示腓骨髓腔内多房性低密度区（CT值约-30~50 HU），分隔清晰；
   - 病灶边缘可见环状硬化带，内部散在点状钙化/骨化影；
   - 皮质向内膨隆、变薄，未见明显软组织侵犯。
3. **MRI表现**：
   - T1WI呈等-低信号（混杂），T2WI为高信号伴分隔（"液-液平面"不典型）；
   - 增强扫描示病灶边缘及分隔强化，内部囊变区无强化；
   - 未见明显骨髓水肿或周围软组织异常信号。

**疾病诊断（按可能性排序）**

1. **骨纤维结构不良**（Fibrous Dysplasia, FD）：
   - 符合典型影像特征：膨胀性多房性溶骨灶、边界硬化边、内部钙化/骨化；54岁虽偏晚但可发生，腓骨为好发部位之一。
2. **动脉瘤样骨囊肿**（Aneurysmal Bone Cyst, ABC）伴继发性钙化：
   - 多房膨胀性病变、液-液平面不典型；54岁患者罕见，但需排除ABC的"陈旧性"变体。
3. **低度恶性软骨肉瘤**（Grade 1 Chondrosarcoma）：
   - 虽边界清晰，但内部钙化/骨化提示软骨源性肿瘤；54岁男性需警惕，但无明显侵袭征象（如皮质破坏、软组织肿块）。

**临床建议**

1. **下一步诊疗**：
   - 行**CT引导下穿刺活检**明确病理类型（优先排除恶性病变）；
   - 完善**全身骨扫描及X线检查**，排查多发性骨纤维结构不良或McCune-Albright综合征。
2. **处置建议**：
   - 若确诊FD且无症状：6-12个月随访X线/CT观察病灶动态变化；
   - 若怀疑恶性肿瘤（如软骨肉瘤）：需行**术前MRI增强+DWI序列**评估范围，制定手术切除方案。

*注：本研判基于提供的影像资料及文献依据（[CONTEXT 3]明确描述FD的"lytic, multilobulated, expansile with sclerotic margin"特征），结合54岁男性无症状体检发现的特点综合分析。*
