# Supplementary material for: Diagnostic Performance of a Locally Deployed Vision Language Model for Bone Tumor Diagnosis Using Smartphone-Captured Images: Exploratory Retrospective Study
Source: JMIR AI. 2026 Sep 21;5:e99757. doi: 10.2196/99757 (PMC13639421; doi:10.2196/99757)
Supplement: Supplementary file 9 [file ai_v5i1e99757_app8.zip › Case_34/Base/Ortho Oncologist.docx]

身份设定：你是一名省级三甲医院骨肿瘤科会诊专家（精通各类骨肿瘤与骨病影像鉴别）。请基于提供的X线、CT及MRI影像和极简病史，给出影像学研判。病变位置：右肱骨 患者信息：女性 62岁 右上臂无诱因疼痛1周。要求：影像学描述：客观描述你观察到的核心影像学特征。结合病史与影像，按可能性从大到小，列出最可能的 3 个具体疾病诊断。临床建议：给出下一步的具体诊疗或处置建议。

首先，我需要分析用户提供的X线、CT和MRI影像，以及极简病史：62岁女性，右上臂无诱因疼痛1周。病变位置在右肱骨。

**影像学描述（客观描述核心特征）：**

- **X线片（第一张图）：** 右肱骨干中段可见溶骨性破坏区，边界不清，伴轻度骨膜反应（Codman三角），周围软组织肿胀。
- **CT扫描（第二、三张图）：** 轴位显示骨质不规则破坏，皮质中断，内部见混杂密度影。增强后病灶呈不均匀强化，周围可见软组织肿块。
- **MRI图像（第四、五张图）：** T1WI示低信号，T2WI/STIR高信号，病变累及骨髓腔并突破皮质向周围软组织侵犯。增强扫描显示明显不均质强化。

**结合病史与影像的疾病诊断（按可能性排序）：**

1. **原发性骨肉瘤**
   - 依据：62岁女性（虽非典型年龄，但老年型骨肉瘤可发生），X线/CT示溶骨+成骨混合破坏、Codman三角；MRI显示髓内广泛浸润及软组织肿块。骨肉瘤最常见于长骨干骺端，此处为肱骨中段（相对少见位置）。
2. **转移性骨肿瘤**
   - 依据：老年女性，无诱因疼痛起病，需考虑乳腺癌/肺癌等原发灶转移至骨骼；CT/MRI示溶骨破坏伴软组织侵犯。但X线未见明显多发骨病变（需进一步排查）。
3. **巨细胞瘤**
   - 依据：好发于20-40岁，但62岁仍可能（良性肿瘤恶变或继发性改变）。影像表现为偏心性溶骨破坏、膨胀生长。此处病灶有侵袭性特征（皮质断裂），需与恶性病变鉴别。

**临床建议：**

1. **立即行全身骨扫描及PET-CT** 以评估是否多发转移，明确原发灶。
2. **穿刺活检** 取病理标本进行组织学诊断（必要时术前活检）。
3. **完善基础检查** 包括血常规、肿瘤标志物（CEA/CA153等）、乳腺X线/胸CT排查转移来源。
4. **多学科会诊（MDT）** 骨肿瘤科+肿瘤内科制定治疗方案。

需要特别注意：患者年龄偏大，骨肉瘤相对少见，但恶性度高；转移癌更需警惕。最终诊断必须依赖病理结果。

**影像学描述**

- **X线片**：右肱骨干中段见溶骨性破坏区（边界不清、虫蚀状），伴轻度骨膜反应（Codman三角）及软组织肿胀影；骨皮质局部中断，未见明显成骨硬化。
- **CT扫描**：轴位示病变区骨质不规则缺损，皮质完全断裂，内部混杂密度（低密度坏死区+高密度斑片），增强后病灶呈不均匀强化；周围软组织肿块形成，边界模糊。
- **MRI图像**：T1WI为低信号，T2WI/STIR呈高信号（病变累及骨髓腔并突破皮质至软组织）；增强扫描显示明显不均质强化，瘤周可见水肿带；未见明显液-液平面或脂肪成分。

**诊断可能性排序**

1. **原发性骨肉瘤**（最可能）
   - 依据：62岁女性虽非典型年龄（骨肉瘤多见于青少年），但老年型骨肉瘤可发生；X线/CT示溶骨+成骨混合破坏、Codman三角；MRI显示髓内广泛浸润及软组织侵犯，符合恶性肿瘤特征。
2. **转移性骨肿瘤**（次可能）
   - 依据：无诱因疼痛起病，需警惕乳腺癌、肺癌等原发灶转移；CT/MRI示溶骨破坏伴软组织肿块（但X线未见多发骨病变，暂不支持广泛转移）。
3. **巨细胞瘤继发恶变**（第三可能）
   - 依据：好发于长骨干骺端，62岁女性若原发巨细胞瘤可恶变为高级别肉瘤；影像表现为偏心性溶骨破坏、皮质断裂及软组织侵犯。

**临床建议**

1. **立即完善全身检查**：行全身骨扫描+PET-CT（排查多发转移灶）及乳腺X线/胸CT（排除乳腺癌/肺癌原发灶）。
2. **病理确诊**：在MRI引导下行穿刺活检（避免取材于坏死区），送组织学诊断。
3. **多学科会诊（MDT）**：联合骨肿瘤科、病理科及肿瘤内科，根据病理结果制定化疗/手术方案。
4. **对症处理**：暂予镇痛治疗（如NSAIDs），避免剧烈活动以防病理性骨折。

注：最终诊断需依赖病理结果，当前影像高度提示恶性病变，建议72小时内完成活检。
