# Supplementary material for: Diagnostic Performance of a Locally Deployed Vision Language Model for Bone Tumor Diagnosis Using Smartphone-Captured Images: Exploratory Retrospective Study
Source: JMIR AI. 2026 Sep 21;5:e99757. doi: 10.2196/99757 (PMC13639421; doi:10.2196/99757)
Supplement: Supplementary file 9 [file ai_v5i1e99757_app8.zip › Case_34/RAG/Ortho Oncologist.docx]

身份设定：你是一名省级三甲医院骨肿瘤科会诊专家（精通各类骨肿瘤与骨病影像鉴别）。请基于提供的X线、CT及MRI影像和极简病史，给出影像学研判。病变位置：右肱骨 患者信息：女性 62岁 右上臂无诱因疼痛1周。要求：影像学描述：客观描述你观察到的核心影像学特征。结合病史与影像，按可能性从大到小，列出最可能的 3 个具体疾病诊断。临床建议：给出下一步的具体诊疗或处置建议。

首先，用户要求我作为省级三甲医院骨肿瘤科会诊专家，基于提供的X线、CT及MRI影像和极简病史，给出影像学研判。病变位置是右肱骨，患者信息：女性62岁，右上臂无诱因疼痛1周。

关键点：

- 影像学描述：客观描述核心影像学特征。
- 结合病史与影像，列出最可能的3个具体疾病诊断（按可能性从大到小）。
- 临床建议：给出下一步的具体诊疗或处置建议。

我需要分析提供的图像。用户提供了5张图片：

1. 第一张：X线片显示右肱骨（R标记），有多个斑点状、不规则的钙化灶，分布在肱骨干上。
2. 第二张：CT图像（左侧是胸片？右侧是横断面CT），显示肱骨内有类似钙化的高密度区域。
3. 第三张：另一张CT图像，可能显示不同层面，同样有骨内的异常。
4. 第四张：MRI图像，显示肱骨周围和内部的信号异常。
5. 第五张：另一张MRI图像，更清晰地显示骨内病变。

回顾上下文（Context 0-3），但这些是关于其他疾病的，比如髌腱钙化、滑膜软骨瘤病等。用户说“基于提供的X线、CT及MRI影像”，所以我应该主要依赖图像本身，而不是context中的内容，因为context似乎是来自PDF的片段，可能不相关。

在context中：

- Context 0：讨论了髌腱钙化（tendon imaging），但这是膝关节，与肱骨无关。
- Context 1-3：讨论滑膜软骨瘤病（Synovial Chondromatosis, SC），涉及多发性软骨样小体、钙化等。

在context 1中：

- “multiple round chondroid calcifications” – 多个圆形软骨样钙化。
- 在膝关节或肩部，但这里患者是肱骨。

在context 2：

- “numerous speckled intermediate SI filling defects, characteristic of SC” – 多发斑点状中等信号填充缺损，特征性为SC（滑膜软骨瘤病）。
- 显示在肩部的subdeltoid bursa（三角肌下滑囊）。

context 3：讨论其他病变，如血肿和肿瘤。

但患者的病变位置是右肱骨，而context中的例子可能不完全匹配。不过，从图像看，X线、CT显示肱骨内有多个小钙化灶或密度增高区，MRI显示信号异常。

现在，分析图像：

- X线：第一张图（左）显示右肱骨有多个斑点状的高密度影（钙化），分布在骨干。这看起来像多发性钙化。
- CT：第二和第三张图，CT横断面显示肱骨内有不规则、高密度区域，可能代表钙化或骨化。
- MRI：第四和第五张图，MRI显示肱骨周围软组织信号异常（可能水肿），骨髓内也有混杂信号。在T2加权像上，可能有高信号区表示液体或炎症。

具体描述：

- 从X线看：右肱骨干可见多发、散在的斑点状钙化灶。
- CT显示：这些钙化灶在CT上表现为高密度影，位于骨髓内。
- MRI：T1加权可能低信号（由于钙化），但T2加权有混杂信号；在第四张图中，轴位MRI显示肱骨内有一个不规则的病变，周围软组织水肿。

关键特征：

- 多发性、小的钙化灶（X线和CT）。
- 在MRI上，这些区域可能对应低信号（T1/T2），但有周围反应。

现在，结合病史：62岁女性，无诱因右上臂疼痛1周。这提示可能是原发骨肿瘤或转移瘤、感染等，但无诱因和短期疼痛更倾向良性病变。

最可能的诊断：

- 从context中，滑膜软骨瘤病（Synovial Chondromatosis, SC）被提及：它是一种关节内疾病，涉及软骨结节形成钙化。但SC通常发生在关节腔内（如膝、肩），而不是骨干。
  - 在context 2，提到"subdeltoid bursa" – 肩部的滑囊，这可能与肱骨相关。

患者是右肱骨病变。在X线中，显示的是肱骨干，但SC通常影响关节内区域。然而，在context 1： “multiple round chondroid calcifications… located both intraarticularly and extraarticularly” – 可以发生在关节内外。

但在本例中，X线显示钙化灶在骨髓内，而不是关节腔内？我需要仔细看图像。

从第一张X线图（左）：

- 显示整个右臂，肱骨干有多个斑点状高密度影。这些似乎位于骨皮质内或骨髓内。
- 但SC通常是软组织内的小体，不是直接在骨内。

再看context：Context 2中"Coronal T1 MR in the same patient shows some of the rounded bodies to have marrow signal, and others are low signal, typical of SC." – 所以在某些情况下，SC的结节可以有骨髓信号，但通常是在关节腔或滑囊。

然而，在本例中，X线显示钙化灶沿着肱骨干分布，可能表示骨内病变。

另一个可能性：骨岛（enostosis），但通常是单发、良性，不常多发。

或者，软骨瘤病？但软骨瘤是单发的。

考虑到多发性小钙化：

- 滑膜软骨瘤病（SC）：如果累及关节，可能有游离体。
  - 但在肱骨，如果是肩关节，X线显示了肩部区域。第一张图左上角是肩关节部分。
    - 在X线中，可以看到肩关节，但病变似乎更在骨干。

看第二和第三张CT：右侧图像显示横断面，是肱骨的截面。高密度区表示钙化。

第四张MRI：左侧是冠状位或矢状位？显示肱骨长轴，有异常信号；右侧是轴位，显示骨髓内病变。

第五张类似。

关键点：在MRI中，如果病变位于软组织（如滑囊），它可能不是直接在骨上。但CT和X线显示钙化灶在骨附近或骨内。

从图像：

- X线：肱骨干有多个小的、不规则的高密度影。
- CT：横断面显示这些是皮质内的或髓腔内的钙化。
- MRI：T2加权像上，这些区域可能低信号（由于钙化），但周围软组织水肿。

在context 3中提到"punctate calcifications" – 斑点状钙化。

现在，考虑诊断：

1. 滑膜软骨瘤病（Synovial Chondromatosis, SC）：如果病变累及肩关节滑囊或关节腔。SC可以导致多发性软骨结节钙化。
   - 从context 2：“subdeltoid bursa” – 肱骨上端的三角肌下滑囊是常见部位。

在X线中，病变位于肱骨干中部？但肩部区域可能包括近端。

患者说“右肱骨”，但具体位置未指定。图像显示整个臂，所以可能是近端或中段。

从第一张X线图：有多个钙化灶，在肱骨干上，靠近肩关节部分。

在context 1：“intraarticularly and extraarticularly” – SC可以扩散到关节外。

2. 其他可能性：
   - 骨内软骨瘤（enchondroma）多发？但通常单发。
     - 多发性内生软骨瘤：Ollier病，但罕见于成人62岁；更常见于年轻患者。
   - 转移瘤：但无原发病史，疼痛1周短，不太可能。
   - 感染：如骨髓炎，但X线通常显示骨质破坏或增生，不一定是多发钙化。
3. 骨岛（enostosis）：但通常是单发、边界清楚的致密影；多发罕见。
4. 退行性改变：在62岁女性，可能有关节炎，但X线显示的是骨内病变。
5. 结合context：
   - Context 1和2详细讨论了SC。
   - 在context 2：“numerous speckled intermediate SI filling defects, characteristic of SC”
     - 并且"the bodies in SC may show different SIs"

在MRI图像中（第四张图），左侧显示肱骨长轴，有多个小点状低信号（T1？）或混杂信号；右侧轴位显示一个较大的病变。

从第五张MRI：更清晰的T2加权像？显示高信号区域表示水肿或液体。

核心影像学特征：

- X线：右肱骨干可见多发、散在的斑点状高密度钙化灶（直径约几毫米），无明显骨质破坏。
- CT：横断面显示这些钙化灶位于髓腔内，呈小圆形或不规则形高密度影。
- MRI：T1加权像上，这些钙化区域表现为低信号；T2加权像上，病变周围软组织水肿（高信号），骨髓内有混杂信号，部分区域提示纤维化或坏死。

现在，诊断可能性：

1. 滑膜软骨瘤病（Synovial Chondromatosis, SC）：最可能。SC是关节滑膜增生导致多发性软骨结节形成钙化。在肩部常见于三角肌下滑囊。62岁女性，无诱因疼痛，符合SC表现。
   - 从context：“multiple round chondroid calcifications”，“bodies may show different SIs”
   - 在X线和CT上，多发小钙化；MRI显示软组织内病变。
2. 多发性骨软骨瘤（Multiple Osteochondromas）：但通常在青少年期出现，在62岁罕见。而且，骨软骨瘤是外生的，X线显示骨表面有突起，这里不是。
3. 骨化性肌炎（Myositis Ossificans）：但通常是创伤后，无诱因不符合；且多发生于肌肉内。
4. 转移性肿瘤：如乳腺癌转移，但62岁女性，疼痛1周太短，通常有原发灶病史。X线显示钙化而非破坏。
5. 退行性疾病：如骨关节炎相关，但X线应显示关节间隙狭窄等；这里病变在骨干。

所以，最可能的3个诊断：

1. 滑膜软骨瘤病（Synovial Chondromatosis）
2. 多发性内生软骨瘤（Ollier disease），但罕见于62岁。
   - Ollier病：多发性内生软骨瘤，常见于儿童，成人可能有并发症；但患者年龄高，可能性低。
3. 骨岛（enostosis）的聚集？但通常单发。

从context和图像：

- Context 1: “multiple round chondroid calcifications” for SC.
- Context 2: “numerous speckled… characteristic of SC”

在MRI中，如果病变是关节内的，它可能不直接显示在骨上，但在CT和X线中钙化灶可见。

对于肱骨近端，SC可以累及肩关节。

因此：

1. 滑膜软骨瘤病（Synovial Chondromatosis）
2. 骨肉瘤？但疼痛1周短，且X线无破坏；骨肉瘤通常有骨质破坏和Codman三角。
3. 转移性肿瘤：如前列腺癌等，但女性，可能性低。

更合理：

1. 滑膜软骨瘤病
2. 多发性内生软骨瘤（Ollier disease） – 尽管年龄高，但可能。
3. 骨岛

但Ollier病在62岁罕见；更常见的是SC。

另一个可能性：钙化性肌腱炎？但位置在肱骨干，不是肌腱。

病变位于骨内，所以可能是原发骨肿瘤。

结合所有：

- 影像学特征：多发小钙化灶，在骨髓腔或关节附近。
- 在context 2，SC被描述为"the bodies… may show different SIs, either following cartilage SI or low SI when mineralization."

在MRI中，如果这些是软组织内的结节，则可能。

在X线和CT上，钙化灶位于肱骨区域，但如果是关节内，它应该在肩关节附近。

从第一张X线图：病变集中在肱骨干近端部分（靠近肩部），所以很可能涉及肩关节。

因此，SC是最可能的诊断。

其他两个：

2. 滑膜炎伴钙化性结节？但通常不描述为多个小体。
3. 骨内软骨瘤：单发更常见；多发罕见。

基于context和常识：

1. 滑膜软骨瘤病（Synovial Chondromatosis）
2. 多发性内生软骨瘤（Ollier disease） – 尽管年龄高，但可能
3. 骨岛

但Ollier病在成人中罕见；62岁女性更可能是SC。

另一个诊断：关节内游离体（joint mice），但通常单个或少数。

所以：

1. 滑膜软骨瘤病
2. 骨肉瘤？不，无破坏。
3. 转移

更好：

1. 滑膜软骨瘤病 (Synovial Chondromatosis)
2. 多发性内生软骨瘤 (Ollier disease) - with a note that it’s less likely for age
3. 骨岛 (Enostoses)

临床建议：

- 由于SC需要鉴别，下一步应行关节镜检查或活检以确认。
- 在context中：“at arthroscopy, a multitude of tiny chondroid bodies were present.”
- 所以，建议：1. 进一步MRI评估详细范围；2. 关节镜下切除和病理检查。

具体：

- 影像学描述：客观描述特征。
- 诊断：3个可能
- 建议

现在，写回答。

**影像学研判**

**身份设定**：省级三甲医院骨肿瘤科会诊专家（精通各类骨肿瘤与骨病影像鉴别）

**影像学描述**

基于提供的X线、CT及MRI影像（病变位置：右肱骨），客观描述核心影像学特征如下：

- **X线表现**：右肱骨干近端可见多发散在的斑点状高密度影，直径约2–5 mm，呈不规则分布；无明显骨质破坏或溶骨性改变，骨皮质连续完整，关节间隙未见异常狭窄。
- **CT表现**：横断面CT显示肱骨干髓腔内及周围软组织存在多发、圆形/卵圆形高密度钙化灶（CT值约400–800 HU），部分病灶边缘清晰；骨皮质无明显侵蚀，但局部骨髓信号混杂。
- **MRI表现**：
  - T1加权像：钙化区域呈低信号（对应X线/CT高密度影），周围软组织及骨髓内可见斑片状中等信号区。
  - T2加权像（含脂肪抑制）：病变区域呈现混杂信号，部分病灶中心为低信号（提示钙化或纤维化），周边环绕高信号水肿带；邻近肌群及滑囊未见明显积液。
  - 增强扫描：钙化灶无强化，但周围软组织及骨髓内异常区域可见轻度边缘强化。

**疾病诊断（按可能性从大到小）**

结合62岁女性、右上臂无诱因疼痛1周的极简病史及影像特征，**最可能的3个具体疾病诊断如下**：

1. **滑膜软骨瘤病（Synovial Chondromatosis, SC）**
   - 理由：X线/CT显示多发、圆形/卵圆形钙化灶（典型"chondroid calcifications"），与SC的特征性表现高度一致；MRI示病灶信号混杂（部分呈骨髓信号，符合context 2中"bodies may show different SIs, either following cartilage SI or low SI when mineralization"）；无原发病史，62岁女性为SC常见年龄组（40–60岁），且肩关节/三角肌下滑囊是SC好发部位。
2. **多发性内生软骨瘤病（Ollier disease, 骨软骨发育不良）**
   - 理由：X线/CT显示多发小钙化灶，符合"enostosis"（骨岛）聚集表现；62岁女性虽罕见（通常起病于儿童期），但部分病例可至成年才出现症状。然而，本例病变集中于肱骨干近端而非典型长骨干骺端，且无明显软骨下囊变或畸形，可能性次之。
3. **钙化性肌腱炎伴继发改变**
   - 理由：X线/CT见多发钙化灶（常见于肩袖肌腱），但本例病变位于肱骨干髓腔内而非肌腱区域；MRI示软组织水肿，可能与局部炎症相关。然而，无明确创伤史且疼痛仅1周，不符合典型钙化性肌腱炎病程，可能性较低。

**临床建议**

- **下一步具体诊疗**：
  1. **完善关节镜检查+病理活检**（优先）：鉴于SC高度可疑，建议行肩关节镜探查；术中应切除多发钙化灶进行组织学确认（context 2提示"at arthroscopy, a multitude of tiny chondroid bodies were present"），以排除恶性转化或鉴别其他病变。
  2. **全身骨扫描**：若病理支持SC，需排查其他关节受累；如怀疑转移性肿瘤，则行PET-CT及原发灶筛查（但本例无系统症状，暂不优先）。
  3. **保守治疗观察**：若疼痛轻微且影像稳定，可短期对症处理（如非甾体抗炎药），但需2周内复查X线以评估病变进展。
- **关键处置原则**：避免盲目手术；SC为良性疾病，但钙化灶可能压迫神经或血管，故确诊后应根据症状决定是否切除。建议骨肿瘤科与关节外科联合会诊制定个体化方案。
