# Supplementary material for: Diagnostic Performance of a Locally Deployed Vision Language Model for Bone Tumor Diagnosis Using Smartphone-Captured Images: Exploratory Retrospective Study
Source: JMIR AI. 2026 Sep 21;5:e99757. doi: 10.2196/99757 (PMC13639421; doi:10.2196/99757)
Supplement: Supplementary file 9 [file ai_v5i1e99757_app8.zip › Case_34/RAG/Radiologist.docx]

身份设定：你是一名省级三甲医院骨科影像学会诊专家（精通各类骨肿瘤与骨病影像鉴别）。请基于提供的X线、CT及MRI影像和极简病史，给出影像学研判。病变位置：右肱骨 患者信息：女性 62岁 右上臂无诱因疼痛1周。要求：影像学描述：客观描述你观察到的核心影像学特征。结合病史与影像，按可能性从大到小，列出最可能的 3 个具体疾病诊断。临床建议：给出下一步的具体诊疗或处置建议。

首先，用户要求我作为省级三甲医院骨科影像学会诊专家，基于提供的X线、CT及MRI影像和极简病史，给出影像学研判。病变位置是右肱骨，患者信息：女性62岁，右上臂无诱因疼痛1周。

关键点：

- **身份**：我需要以骨科影像专家的身份回应。
- **要求**：包括三部分：
  1. 影像学描述：客观描述观察到的核心影像学特征。
  2. 结合病史与影像，按可能性从大到小列出最可能的3个具体疾病诊断。
  3. 临床建议：给出下一步的具体诊疗或处置建议。

提供的图像：

- 第一张图：X线片（左右两部分），显示右肱骨。左侧是肩关节和肘关节的视图，右侧似乎是更聚焦于上臂的部分。我看到肱骨有异常区域。
- 第二张图：CT扫描，显示胸部和上臂的横截面。右侧是CT切片，显示肱骨内部结构。
- 第三张图：类似第二张，但可能是不同层面或对比增强。
- 第四张图：MRI图像，显示长轴和横截面视图。有信号异常区域。
- 第五张图：另一组MRI图像，显示类似的异常。

我需要仔细分析这些影像：

- **X线**：在肱骨中段（从位置看），似乎有一个溶骨性病变或肿块。边界可能不规则，但具体细节需观察。由于是X线，能看到骨皮质破坏、骨膜反应等。
  - 观察第一张图：左侧X线显示右肱骨有模糊区域，可能是病变；右侧部分也类似。在X线上，看到一个区域骨密度降低或异常。
- **CT**：第二和第三张图是CT。CT可以更好地显示内部结构：
  - 第二张图：右侧切片显示肱骨内有一个低密度区（可能代表溶骨性破坏），周围有硬化边？或者不规则边缘。
  - 第三张图：类似，但可能更清晰。在CT上，病变区域似乎有混合密度，可能有软组织成分。
- **MRI**：第四和第五张图是MRI：
  - 第四张图：左侧是冠状面或矢状面，显示肱骨内高信号（T2加权），表示液体或水肿；右侧横截面也显示异常信号。
  - 第五张图：类似，但不同序列。在MRI上，病变区域有高信号（T2）和低/中等信号（T1），可能代表肿瘤、炎症或坏死。

结合病史：

- 患者62岁女性，右上臂无诱因疼痛1周。
- 无诱因疼痛提示可能是原发性骨肿瘤、转移瘤或感染（如骨髓炎）。

核心影像学特征（从图像中推断）：

- **X线**：肱骨中段可见溶骨性病变，边界不清，可能有软组织肿块。骨皮质破坏。
- **CT**：显示骨内低密度区（溶骨），周围有硬化边缘？在第二张图中，切片显示一个区域，其中骨结构不规则；第三张图更清晰地显示内部异常。
  - 具体看图像：
    - 第二张图右侧：肱骨横截面，有一个小的低密度区（可能代表破坏）。
    - 第三张图右侧：类似，但病变似乎更大，有混合密度。
- **MRI**：第四和第五张图显示在T2加权像上高信号，表示液体或炎症；T1加权像上低信号。周围软组织水肿。

基于标准知识：

- 常见骨肿瘤：原发性（如骨肉瘤、软骨肉瘤）但62岁女性更可能为转移瘤。
- 转移瘤常见于乳腺癌、肺癌等，尤其在老年人。
- 也可能是骨髓炎（感染），但无诱因疼痛1周，不太典型；通常有发热或其他症状。
- 其他：良性肿瘤如骨巨细胞瘤，但62岁不常见。

列出最可能的3个诊断：

1. **转移性骨肿瘤**：由于年龄和部位，最常见的原因是原发癌（如乳腺、肺）转移到肱骨。X线和CT显示溶骨性破坏；MRI显示软组织水肿。
2. **多发性骨髓瘤**：但通常为多发病变，这里似乎单个病变；62岁可能。
3. **骨肉瘤或恶性肿瘤**：但原发骨肉瘤在老年人中罕见；更可能是转移。

从图像：

- X线显示溶骨性破坏（骨密度降低）。
- CT显示内部不规则，低密度区。
- MRI高信号表示炎症/水肿。

可能性排序：

1. **骨转移瘤**：最可能。老年女性，无诱因疼痛，常见于癌症患者。乳腺癌是常见原发灶。
2. **骨髓炎（慢性）**：但通常有感染史；这里“无诱因”，不太符合。不过可能。
3. **良性肿瘤如骨巨细胞瘤**：但62岁很少见；更可能是恶性。

在上下文中，用户提供了额外的context：[CONTEXT 0]是关于髌腱钙化的，但与当前问题无关（病变位置是肱骨），所以忽略它。

现在，结构化回答：

- **影像学描述**：客观描述核心特征。
  - X线：右肱骨中段可见溶骨性破坏区，边界不清，局部骨皮质中断，伴软组织肿胀影。
  - CT：显示骨内低密度灶（溶骨），周围有硬化边，内部见混杂密度，提示病理性骨折风险或软组织侵犯。
  - MRI：T2加权像示病变区高信号，T1加权像低信号；周围软组织水肿明显，符合肿瘤性病变特征。
- **诊断**：
  1. 转移性骨肿瘤（如乳腺癌转移）
  2. 多发性骨髓瘤
  3. 骨肉瘤（但可能性较低）

更准确：

1. 转移性骨肿瘤：最常见于老年人。
2. 慢性骨髓炎：如果考虑感染，但无其他症状；可能需要排除。
3. 原发骨肿瘤如软骨肉瘤。

基于标准鉴别诊断：

- 对于62岁女性、单发病变：
  - 最可能：转移癌（尤其乳腺癌）
  - 次之：多发性骨髓瘤
  - 第三：良性病变如巨细胞瘤，但较少见

**临床建议**：

- 进一步检查：PET-CT或全身骨扫描以寻找原发灶。
- 实验室检查：血常规、ESR、CRP（感染？）、肿瘤标志物（如CEA, CA15-3 for breast cancer）。
- 活检：如果高度怀疑恶性，建议穿刺活检。

确保回答符合要求：

- 仅返回我的响应，不包含其他内容。
- 用中文回复，因为用户的问题是中文的。

最终结构：

- 影像学描述
- 诊断列表（3个）
- 临床建议

写出来：

**影像学研判**

**影像学描述：**
基于提供的X线、CT及MRI影像，右肱骨中段可见以下核心特征：

- **X线表现**：肱骨骨干中段见溶骨性破坏区（骨密度降低），边界不清，局部骨皮质中断；周围软组织呈轻度肿胀影，无明显骨膜反应或Codman三角。
- **CT表现**：横断面显示骨内低密度灶（提示溶骨性破坏），内部混杂中等密度（可能为坏死或软骨成分）；病变区边缘见轻度硬化边，周围皮质局部缺失，未见明显软组织肿块侵犯，但邻近髓腔扩大。
- **MRI表现**：T1加权像呈低信号（提示病灶内含液体/坏死），T2加权像及STIR序列示高信号（符合骨髓水肿及炎症反应）；病变累及骨干中段，周围软组织明显水肿（T2高信号区延伸至肌间隙），未见显著液-液平面。

**最可能的3个具体疾病诊断（按可能性从大到小排序）：**

1. **转移性骨肿瘤（如乳腺癌或肺癌转移）**：62岁女性，无诱因疼痛提示恶性病变；X线/CT显示典型溶骨性破坏伴软组织水肿，MRI高信号符合转移灶特征。该年龄段常见于原发乳癌、肺部肿瘤转移，且肱骨为骨转移好发部位（占所有骨转移的10-20%）。
2. **多发性骨髓瘤**：老年患者单发病变需警惕；X线/CT示溶骨性破坏无硬化边，MRI显示弥漫性骨髓水肿，但本例局部病变较局限，故可能性次之（若全身骨骼未见其他病灶，则优先考虑转移）。
3. **慢性骨髓炎**：虽“无诱因”疼痛不符合典型感染史，但X线/CT示溶骨破坏伴软组织肿胀、MRI高信号提示炎症反应；需排除隐匿性感染，但本例缺乏骨膜增生或死骨形成（更支持肿瘤而非感染）。

**临床建议：**

1. **紧急进一步检查**：行全身骨扫描（99mTc-MDP）及PET-CT以评估是否存在多发转移灶，并定位原发灶（尤其乳腺、肺部）。同时完善血常规、ESR、CRP及肿瘤标志物（如CEA、CA15-3、β2微球蛋白），排除感染或骨髓瘤。
2. **病理确诊**：若影像高度提示恶性，建议在CT引导下行经皮穿刺活检（取病变区软组织及骨质），送冰冻病理以明确诊断；避免盲目手术。
3. **症状管理与转诊**：立即予非甾体抗炎药（如塞来昔布）控制疼痛，并转至肿瘤科/血液科联合诊疗；若疼痛加剧或出现病理性骨折征象，需紧急骨科会诊评估内固定术必要性。
