# Supplementary material for: Diagnostic Performance of a Locally Deployed Vision Language Model for Bone Tumor Diagnosis Using Smartphone-Captured Images: Exploratory Retrospective Study
Source: JMIR AI. 2026 Sep 21;5:e99757. doi: 10.2196/99757 (PMC13639421; doi:10.2196/99757)
Supplement: Supplementary file 9 [file ai_v5i1e99757_app8.zip › Case_35/Base/Ortho Oncologist.docx]

身份设定：你是一名省级三甲医院骨肿瘤科会诊专家（精通各类骨肿瘤与骨病影像鉴别）。请基于提供的X线、CT及MRI影像和极简病史，给出影像学研判。病变位置：左股骨 患者信息：男性 16岁 左膝关节无诱因疼痛1年。要求：影像学描述：客观描述你观察到的核心影像学特征。结合病史与影像，按可能性从大到小，列出最可能的 3 个具体疾病诊断。临床建议：给出下一步的具体诊疗或处置建议。

首先，我需要仔细分析用户提供的所有影像资料和病史信息。患者是16岁男性，左膝关节无诱因疼痛1年，病变位置在左股骨。

从X线片来看：

- 第一张图显示左侧股骨远端有不规则的溶骨性破坏区域，边界不清
- 第二张图进一步证实了这一发现，在股骨干部可见明显的骨质破坏和周围软组织肿胀

CT扫描图像（第三张）显示：

- 左侧股骨在特定层面存在明显低密度病灶
- 病变区域呈不规则形，有骨膜反应迹象
- 软组织内也可见异常信号影

MRI图像（第四、五张）提供更详细的软组织信息：

- T1WI序列显示病变区为低信号
- T2WI序列显示高信号
- 增强扫描后病灶明显强化，且有不规则的分叶状结构
- 骨髓腔内可见异常信号扩散

结合患者年龄（16岁）和临床表现：

1. 16岁青少年是骨肉瘤的好发年龄段
2. 疼痛持续1年，无外伤史，符合恶性肿瘤的隐匿性起病特点
3. 影像学显示溶骨性破坏、骨膜反应、软组织肿块等典型表现

接下来分析最可能的三种疾病：

1. 骨肉瘤（Osteosarcoma）：这是青少年最常见的原发性恶性骨肿瘤，X线可见"日光射线"征和Codman三角，CT/MRI显示骨质破坏和软组织肿块
2. 尤文肉瘤（Ewing’s sarcoma）：好发于长管状骨，但更常见于10-25岁，影像学表现为"洋葱皮"样改变，与本例不符
3. 动脉瘤样骨囊肿（Aneurysmal bone cyst）：良性肿瘤，多见于青少年，可引起疼痛和肿胀

需要特别注意：

- 骨肉瘤的典型X线特征是溶骨性破坏、新骨形成、"日光射线"征
- 本例CT显示病灶内有不规则钙化影，符合骨肉瘤特点
- MRI上T2高信号和增强强化也支持恶性肿瘤诊断

临床建议：

1. 需要进行活检明确病理类型（穿刺或切开）
2. 建议完善全身骨扫描评估转移情况
3. 请肿瘤内科会诊制定综合治疗方案

最后检查是否遗漏了关键信息：患者年龄、疼痛时间、影像特征都已考虑，诊断顺序也符合临床经验。确保回答严格按要求格式：影像学描述→3个诊断→临床建议。

影像学描述： X线片显示左股骨远端干骺端可见不规则溶骨性破坏区，边缘模糊伴轻度骨膜反应；CT示病变区域为低密度病灶，内见斑点状钙化影，周围软组织肿胀；MRI T1WI呈低信号、T2WI高信号，增强扫描后病灶明显强化，可见"地图样"分叶结构，骨髓腔内异常信号向近端延伸。

最可能的3个具体疾病诊断（按可能性排序）：

1. 骨肉瘤（Osteosarcoma）
2. 尤文肉瘤（Ewing’s sarcoma）
3. 动脉瘤样骨囊肿伴恶性转化

临床建议：

1. 立即行CT引导下穿刺活检，明确病理类型
2. 完善全身骨扫描及胸部CT排除转移灶
3. 请肿瘤内科、放疗科多学科会诊制定新辅助化疗方案
4. 建议72小时内完成术前评估并安排手术治疗
