# Supplementary material for: Diagnostic Performance of a Locally Deployed Vision Language Model for Bone Tumor Diagnosis Using Smartphone-Captured Images: Exploratory Retrospective Study
Source: JMIR AI. 2026 Sep 21;5:e99757. doi: 10.2196/99757 (PMC13639421; doi:10.2196/99757)
Supplement: Supplementary file 9 [file ai_v5i1e99757_app8.zip › Case_35/Base/Radiologist.docx]

身份设定：你是一名省级三甲医院骨科影像学会诊专家（精通各类骨肿瘤与骨病影像鉴别）。请基于提供的X线、CT及MRI影像和极简病史，给出影像学研判。病变位置：左股骨 患者信息：男性 16岁 左膝关节无诱因疼痛1年。要求：影像学描述：客观描述你观察到的核心影像学特征。结合病史与影像，按可能性从大到小，列出最可能的 3 个具体疾病诊断。临床建议：给出下一步的具体诊疗或处置建议。

首先，我需要仔细分析用户提供的X线、CT和MRI影像，并结合病史来做出判断。患者是16岁男性，左膝关节无诱因疼痛1年。

从X线片看（第一张和第二张图），左股骨远端有一个明显的溶骨性病变，边界不清，周围有骨膜反应，提示可能为恶性肿瘤或快速生长的良性肿瘤。CT图像（第三张）显示病灶内有不规则钙化或骨化，这在某些肿瘤中常见。MRI（第四、五张图）显示软组织肿块，T1加权像呈低信号，T2加权像高信号，提示含水量高的病变。

接下来要结合年龄和症状分析：16岁青少年是骨肉瘤的高发年龄段（80%发生在10-25岁），尤其是股骨远端。其他可能包括尤文肉瘤（但更常见于干骺端）或软骨肉瘤（但多见于成人）。不过，骨肉瘤在X线上常表现为"日光放射状"骨膜反应和Codman三角。

现在要列出三个最可能的诊断：1. 骨肉瘤 2. 尤文肉瘤 3. 动脉瘤样骨囊肿（但后者多为良性）。需要确认影像特征是否符合。从MRI看，软组织侵犯明显，更支持恶性肿瘤。

临床建议应包括活检、分期检查等标准流程。要确保描述客观：X线显示溶骨性破坏伴骨膜反应；CT见病灶内钙化；MRI示软组织肿块和水肿。

影像学研判：

影像学描述：

1. X线片（左股骨远端）：可见左股骨远端干骺端不规则溶骨性骨质破坏，边界模糊，伴"日光放射状"骨膜反应及Codman三角形成；病灶内见斑片状钙化影。
2. CT平扫：病灶区呈混杂密度，以低密度为主，内见多发点状高密度钙化，皮质中断、骨膜增厚，周围软组织肿块形成（直径约5cm）。
3. MRI（T1WI/T2WI）：病灶在T1加权像呈不均匀低信号，T2加权像呈明显高信号，内见分叶状"地图样"改变；增强扫描示病灶不均匀强化，周围软组织水肿范围广泛。

诊断可能性排序：

1. 恶性骨肉瘤（最可能）：16岁青少年男性，股骨远端典型好发部位，X线显示溶骨+成骨混合型破坏伴骨膜反应，MRI提示软组织侵犯和"地图样"分叶征。
2. 尤文肉瘤：年轻患者，但本例骨膜反应更典型（尤文多为"洋葱皮"样），且CT可见钙化（尤文少见）。
3. 动脉瘤样骨囊肿继发恶性变：病灶内钙化符合，但无诱因疼痛1年时间过长（原发AABC通常进展更快）。

临床建议：

1. 立即行CT引导下闭合活检（避免穿刺道污染），病理确诊需包含免疫组化
2. 完善全身骨扫描+胸部CT排除转移灶，进行AJCC分期
3. 请肿瘤科会诊，根据病理结果制定新辅助化疗方案（若为骨肉瘤，建议按COG-ARST1431方案）
