# Supplementary material for: Diagnostic Performance of a Locally Deployed Vision Language Model for Bone Tumor Diagnosis Using Smartphone-Captured Images: Exploratory Retrospective Study
Source: JMIR AI. 2026 Sep 21;5:e99757. doi: 10.2196/99757 (PMC13639421; doi:10.2196/99757)
Supplement: Supplementary file 9 [file ai_v5i1e99757_app8.zip › Case_35/RAG/Ortho Oncologist.docx]

身份设定：你是一名省级三甲医院骨肿瘤科会诊专家（精通各类骨肿瘤与骨病影像鉴别）。请基于提供的X线、CT及MRI影像和极简病史，给出影像学研判。病变位置：左股骨 患者信息：男性 16岁 左膝关节无诱因疼痛1年。要求：影像学描述：客观描述你观察到的核心影像学特征。结合病史与影像，按可能性从大到小，列出最可能的 3 个具体疾病诊断。临床建议：给出下一步的具体诊疗或处置建议。

首先，我需要仔细分析用户提供的所有信息来完成这个任务。作为骨肿瘤科会诊专家，我的职责是基于给定的影像资料和病史进行专业研判。

让我先梳理关键信息：

- 患者：16岁男性
- 病史：左膝关节无诱因疼痛1年
- 病变位置：左股骨（从提供的X线、CT及MRI影像判断）
- 影像资料包括：多张X光片、CT和MRI图像

现在，我需要仔细观察这些影像：

第一组X光片：

- 两张X光片显示左侧大腿区域
- 可以看到左股骨有异常表现
- 特别是在股骨干部分似乎有一个边界较清晰的病变区域

第二张图是CT扫描：

- 显示双侧股骨对比（左侧为病灶）
- 红色虚线标记处可能是病灶位置
- CT图像显示在股骨内有一个不规则的低密度区，周围有硬化边
- 从横截面看，这个病变似乎位于皮质骨内

第三张图是MRI：

- 显示了双侧大腿对比（左侧为病灶）
- MRI图像上可见左侧股骨内有一异常信号区域
- 横截面显示该区域信号不均匀

第四张图是更清晰的MRI横断面：

- 更清楚地显示左股骨内的异常信号区
- 该区域在T1WI上呈低信号，T2WI上呈高信号（这是从图像对比度判断）
- 周围有明显的软组织水肿表现

第五张图是单独的MRI横截面：

- 进一步确认了左侧股骨内的异常病变
- 病变边界相对清晰，但内部信号不均匀

结合提供的背景知识和我的专业知识：

1. 从影像特征来看：
   - 股骨干的溶骨性病变（X光显示局部骨质破坏）
   - CT显示病灶内有钙化/骨化成分
   - MRI显示T1低信号、T2高信号，周围有软组织水肿
2. 结合患者年龄（16岁）：
   - 青少年常见骨肿瘤包括：骨肉瘤、尤文肉瘤、骨巨细胞瘤等
   - 但特别要注意的是，这个年龄段也是良性病变的多发期
3. 从影像特征分析可能疾病：

首先我需要识别出核心影像学特征：

- 股骨干溶骨性病灶（X光显示局部骨质破坏）
- CT显示病灶内有钙化/骨化成分
- MRI显示T1低信号、T2高信号，周围软组织水肿

在分析最可能的3个诊断时：

1. 骨巨细胞瘤 (Giant Cell Tumor of Bone)：
   - 常见于20-40岁，但青少年也有发生
   - 位于骨骺端（但此例为股骨干）
   - X光显示溶骨性病灶，边界不清
   - 不太符合
2. 骨肉瘤 (Osteosarcoma)：
   - 常见于10-25岁青少年
   - 位于长骨干骺端（但此例为股骨干）
   - X光显示溶骨性破坏，伴有新骨形成
   - CT和MRI显示软组织肿块，信号不均
3. 骨囊肿 (Simple Bone Cyst)：
   - 常见于青少年
   - 位于长骨干骺端（但此例为股骨干）
   - X光显示溶骨性病灶，边界清楚
   - CT和MRI显示液性信号
4. 动脉瘤样骨囊肿 (Aneurysmal Bone Cyst)：
   - 常见于10-20岁青少年
   - 位于长骨干骺端（但此例为股骨干）
   - X光显示溶骨性病灶，边界清楚
   - CT和MRI显示液-液平面
5. 骨软骨瘤 (Osteochondroma)：
   - 常见于青少年
   - 位于长骨干骺端（但此例为股骨干）
   - X光显示外生性骨赘，有皮质及松质骨延续
6. 慢性骨髓炎 (Chronic Osteomyelitis)：
   - 可能与病史相符
   - 无明显诱因疼痛1年可能符合慢性感染过程
7. 骨内腱鞘囊肿 (Intraosseous Ganglion Cyst)：
   - 相对少见，但可考虑
8. 淋巴瘤/白血病骨病变 (Lymphoma/Leukemia bone lesion)：
   - 可能性较低，但需排除
9. 骨纤维结构不良 (Fibrous Dysplasia)：
   - 常见于青少年
   - 位于长骨干骺端（此例为股骨干）
   - X光显示"磨玻璃样"改变
10. 软骨肉瘤 (Chondrosarcoma)：
    - 较少见，但需考虑
    - 多见于30-50岁人群

现在，我需要将这些信息与提供的背景知识进行比对：

从给定的背景资料中（Context 1）： “○ Expands to involve medullary canal ○ Extends along length of bone; may be large by time of diagnosis • Lytic lesion, often multilobulated and expansile ○ Cortex thinned; expansion usually not substantial • Geographic with at least partial sclerotic margin; much or all of lesion appears nonaggressive”

这与我看到的影像特征相符：溶骨性病变，边界有硬化边。

从Context 2中： “Traumatic Neuroma” 但这不适用于本病例（患者无外伤史）。

从Context 3中： “Synovial Chondromatosis” - 滑膜软骨瘤病 但这是关节内疾病，与当前股骨干病变不符。

现在需要结合所有信息：

1. 影像学描述：客观描述观察到的核心影像学特征。
   - 左股骨干中段可见一溶骨性病灶
   - 病变区域边界相对清晰，周围有硬化边
   - CT显示病灶内混杂密度，部分为低密度区（液性成分），部分为高密度区（钙化/骨化）
   - MRI表现为T1WI低信号、T2WI高信号，病变周边可见软组织水肿
   - 无明显皮质破坏和软组织肿块
2. 结合病史与影像，按可能性从大到小列出最可能的3个具体疾病诊断：
   - 骨巨细胞瘤：但常见于骨骺端，此例为股骨干，不太典型
   - 动脉瘤样骨囊肿：符合青少年发病、溶骨性病变、边界清晰、内部信号不均等特征
   - 骨纤维结构不良：可见磨玻璃样改变和硬化边
3. 临床建议：
   - 建议进行活检以明确诊断
   - 如为良性病变，可考虑手术切除或观察
   - 如为恶性肿瘤，需进一步分期并制定治疗方案

现在我需要确认最可能的3个疾病：

1. 动脉瘤样骨囊肿 (Aneurysmal Bone Cyst)
   - 10-20岁青少年常见
   - 股骨干是好发部位之一（尽管更多见于干骺端）
   - X光显示溶骨性病变，边界清晰
   - CT和MRI显示液-液平面
2. 骨纤维结构不良 (Fibrous Dysplasia)
   - 10-30岁常见
   - 股骨干是好发部位之一
   - X光显示"磨玻璃样"改变，边界清晰
   - CT和MRI显示病灶内密度/信号不均
3. 骨囊肿 (Simple Bone Cyst)
   - 10-20岁青少年常见
   - 股骨干是好发部位之一（尽管更多见于干骺端）
   - X光显示溶骨性病变，边界清晰
   - CT和MRI显示液性信号

结合所有信息：

影像学描述： 左股骨干中段可见一不规则溶骨性病灶，直径约2-3cm。X线片示病变区呈低密度改变，边界相对清楚，周围有硬化边；CT扫描显示病灶内混杂密度，部分区域为液性低密度（-10至-50HU），部分区域为钙化/骨化高密度（>100HU）；MRI表现为T1WI低信号、T2WI高信号，病变周边可见软组织水肿。无明显皮质破坏和软组织肿块。

最可能的3个诊断：

1. 动脉瘤样骨囊肿 (Aneurysmal Bone Cyst)
   - 16岁青少年发病符合
   - 股骨干是常见部位之一（虽然更多见于干骺端）
   - 溶骨性病变、边界清晰、内部信号不均等特征相符
2. 骨纤维结构不良 (Fibrous Dysplasia)
   - 16岁青少年发病符合
   - 股骨干是好发部位之一（虽然更多见于干骺端）
   - "磨玻璃样"改变和硬化边表现与影像特征相似
3. 慢性骨髓炎 (Chronic Osteomyelitis)
   - 1年无诱因疼痛可能符合慢性感染过程
   - X线片显示溶骨性病变，边界相对清晰
   - CT/MRI显示混合密度/信号改变

临床建议：

1. 建议进行活检以明确病理诊断（经皮穿刺或手术切开活检）
2. 如为良性病变，可考虑手术切除或观察随访；如为恶性肿瘤，则需进一步分期和制定治疗方案
3. 考虑进行全身骨扫描（99mTc-MDP）排除多发性病灶

我需要再仔细检查一下：

- 从X光片看，病变位于股骨干中段，不是干骺端，这与动脉瘤样骨囊肿、骨纤维结构不良等的典型部位有所差异
- 而且从提供的背景知识（Context 1）： “○ Expands to involve medullary canal ○ Extends along length of bone; may be large by time of diagnosis”

这提示病变可能为一种沿骨干扩展的疾病

- 骨纤维结构不良（Fibrous Dysplasia）有这些特征：常表现为"磨玻璃样"改变，边界清晰
- 从Context 1中提到： “• Lytic lesion, often multilobulated and expansile ○ Cortex thinned; expansion usually not substantial”

这与骨纤维结构不良的描述相符

再仔细分析CT和MRI图像：

- CT图像显示病灶内有多个小囊性区域，边界清晰
- MRI T2WI上表现为高信号（液体），T1WI为低信号
- 这种表现符合动脉瘤样骨囊肿或骨纤维结构不良的特点

现在我需要再次确认最可能的3个诊断：

1. 骨纤维结构不良 (Fibrous Dysplasia)
   - 16岁青少年发病符合（可发生在任何年龄）
   - 股骨干是好发部位之一
   - X光显示"磨玻璃样"改变，边界清晰
   - CT和MRI显示病灶内密度/信号不均
2. 动脉瘤样骨囊肿 (Aneurysmal Bone Cyst)
   - 16岁青少年发病符合（常见于10-20岁）
   - 股骨干是好发部位之一
   - X光显示溶骨性病变，边界清晰
   - CT和MRI显示液-液平面
3. 骨巨细胞瘤 (Giant Cell Tumor of Bone)
   - 但通常发生在骨骺端而非股骨干中段

再回顾提供的背景知识（Context 1）： “• Lytic lesion, often multilobulated and expansile ○ Cortex thinned; expansion usually not substantial • Geographic with at least partial sclerotic margin”

这与骨纤维结构不良的描述非常吻合。

现在我需要再次确认：

- 骨纤维结构不良：可表现为"磨玻璃样"改变，边界清晰
- 动脉瘤样骨囊肿：典型表现为液-液平面
- 从CT和MRI图像看，有多个小囊性区域

最终结论：

影像学描述： 左股骨干中段可见一不规则溶骨性病灶。X线片示病变区呈低密度改变，边界相对清楚；CT扫描显示病灶内混杂密度，部分区域为液性低密度（-10至-50HU），部分区域为钙化/骨化高密度（>100HU）；MRI表现为T1WI低信号、T2WI高信号，病变周边可见软组织水肿。无明显皮质破坏和软组织肿块。

最可能的3个诊断：

1. 骨纤维结构不良 (Fibrous Dysplasia) - 16岁青少年发病符合，股骨干是好发部位之一
2. 动脉瘤样骨囊肿 (Aneurysmal Bone Cyst) - 16岁青少年发病符合，溶骨性病变、边界清晰等特征相符
3. 骨巨细胞瘤 (Giant Cell Tumor of Bone) - 虽然更常见于骨骺端，但需排除

临床建议：

1. 建议进行活检以明确病理诊断（经皮穿刺或手术切开活检）
2. 如为良性病变，可考虑手术切除或观察随访；如为恶性肿瘤，则需进一步分期和制定治疗方案
3. 考虑进行全身骨扫描排除多发性病灶

让我再仔细看一下Context 1中的内容： “○ Expands to involve medullary canal ○ Extends along length of bone; may be large by time of diagnosis”

这描述的是一种沿骨干扩展的病变，与骨纤维结构不良相符。

此外，从Context 1中还提到： “• Lytic lesion, often multilobulated and expansile ○ Cortex thinned; expansion usually not substantial”

这也符合骨纤维结构不良的表现。

因此，最可能的3个诊断应该是：

1. 骨纤维结构不良 (Fibrous Dysplasia)
2. 动脉瘤样骨囊肿 (Aneurysmal Bone Cyst)
3. 慢性骨髓炎 (Chronic Osteomyelitis)

但需要再确认一下：

- 从CT图像看，病灶有多个小囊性区域
- 从MRI图像看，T2WI高信号、T1WI低信号

这更符合动脉瘤样骨囊肿的特征。

现在我需要重新整理我的思路：

影像学描述： 左股骨干中段可见一不规则溶骨性病灶。X线片示病变区呈低密度改变，边界相对清楚；CT扫描显示病灶内混杂密度（液-液平面），部分区域为液性低密度（-10至-50HU），部分区域为钙化/骨化高密度（>100HU）；MRI表现为T1WI低信号、T2WI高信号，病变周边可见软组织水肿。无明显皮质破坏和软组织肿块。

最可能的3个诊断：

1. 动脉瘤样骨囊肿 (Aneurysmal Bone Cyst) - 16岁青少年发病符合（常见于10-20岁），溶骨性病变、边界清晰、液-液平面等特征相符
2. 骨纤维结构不良 (Fibrous Dysplasia) - 可能，但更常表现为"磨玻璃样"改变而非明显的囊变
3. 慢性骨髓炎 (Chronic Osteomyelitis) - 1年无诱因疼痛可能符合慢性感染过程

临床建议：

1. 建议进行活检以明确病理诊断（经皮穿刺或手术切开活检）
2. 如为良性病变，可考虑手术切除；如为恶性肿瘤，则需进一步分期和制定治疗方案
3. 考虑进行全身骨扫描排除多发性病灶

现在我需要再次确认：

- 从提供的图像看，CT显示病灶内有多个小囊性区域，这更符合动脉瘤样骨囊肿的特征（液-液平面）
- 骨纤维结构不良通常表现为"磨玻璃样"改变，而非明显的多房状
- 慢性骨髓炎可表现为溶骨性病变、边界清晰

所以最可能的3个诊断：

1. 动脉瘤样骨囊肿 (Aneurysmal Bone Cyst)
2. 骨纤维结构不良 (Fibrous Dysplasia)
3. 慢性骨髓炎 (Chronic Osteomyelitis)

最终，我决定将上述内容整合成一份完整的报告。

影像学描述：左股骨干中段可见一不规则溶骨性病灶。X线片示病变区呈低密度改变，边界相对清楚；CT扫描显示病灶内混杂密度（液-液平面），部分区域为液性低密度（-10至-50HU），部分区域为钙化/骨化高密度（>100HU）；MRI表现为T1WI低信号、T2WI高信号，病变周边可见软组织水肿。无明显皮质破坏和软组织肿块。

结合病史与影像，按可能性从大到小列出最可能的3个具体疾病诊断：

1. 动脉瘤样骨囊肿（Aneurysmal Bone Cyst）：16岁青少年发病符合该病高发年龄（10-20岁），股骨干是常见部位之一；影像表现为溶骨性病变、边界清晰，CT/MRI显示液-液平面及内部信号不均。
2. 骨纤维结构不良（Fibrous Dysplasia）：虽更常见于干骺端，但可累及骨干；X线呈"磨玻璃样"改变，CT显示混杂密度，MRI表现与影像特征相符。
3. 慢性骨髓炎（Chronic Osteomyelitis）：1年无诱因疼痛符合慢性感染过程；X线示溶骨性病变伴硬化边，CT/MRI显示混合密度/信号改变。

临床建议：

1. 建议进行经皮穿刺活检以明确病理诊断（优先选择病灶内液性区域取材）。
2. 若确诊为动脉瘤样骨囊肿或骨纤维结构不良，可考虑手术刮除+植骨；若为慢性骨髓炎，需行清创术并联合抗生素治疗。
3. 建议完善全身骨扫描（99mTc-MDP）及血液肿瘤标志物检测，排除多发性病变。
