# Supplementary material for: Diagnostic Performance of a Locally Deployed Vision Language Model for Bone Tumor Diagnosis Using Smartphone-Captured Images: Exploratory Retrospective Study
Source: JMIR AI. 2026 Sep 21;5:e99757. doi: 10.2196/99757 (PMC13639421; doi:10.2196/99757)
Supplement: Supplementary file 9 [file ai_v5i1e99757_app8.zip › Case_35/RAG/Radiologist.docx]

身份设定：你是一名省级三甲医院骨科影像学会诊专家（精通各类骨肿瘤与骨病影像鉴别）。请基于提供的X线、CT及MRI影像和极简病史，给出影像学研判。病变位置：左股骨 患者信息：男性 16岁 左膝关节无诱因疼痛1年。要求：影像学描述：客观描述你观察到的核心影像学特征。结合病史与影像，按可能性从大到小，列出最可能的 3 个具体疾病诊断。临床建议：给出下一步的具体诊疗或处置建议。

首先，用户要求我作为省级三甲医院骨科影像学会诊专家，基于提供的X线、CT及MRI影像和极简病史进行研判。病变位置是左股骨，患者信息：男性16岁，左膝关节无诱因疼痛1年。

我的任务包括：

1. 影像学描述：客观描述观察到的核心影像学特征。
2. 结合病史与影像，按可能性从大到小列出最可能的3个具体疾病诊断。
3. 临床建议：给出下一步的具体诊疗或处置建议。

现在，分析提供的图像：

- **X线图像**（第一张和第二张）：
  - 第一张：显示左股骨下段，似乎有局部密度改变。但图像模糊，细节不清。
  - 第二张：类似，可能显示股骨远端的异常。
  - 从描述看，这些是X光片，显示左股骨。
- **CT图像**（第三张）：
  - 显示双侧股骨的冠状面和轴位。左侧股骨（L标记）似乎有局部病变，可能表现为低密度区或异常结构。
  - 在CT上，可以看到左侧股骨有一个区域与右侧对比明显不同。
- **MRI图像**（第四张和第五张）：
  - 第四张：显示冠状面和轴位，左侧股骨有信号异常。在T1加权像上可能表现为低信号，在T2加权或STIR序列上高信号。
  - 第五张：更清晰的轴位MRI，显示股骨内有一个区域与周围不同，可能是病变。

关键特征：

- 病变位于左股骨（具体位置？从图像看，似乎是近端或中段）。
- 患者16岁男性，疼痛1年无诱因。
- 从影像描述：X线可能显示溶骨性改变；CT显示低密度区、皮质破坏等；MRI显示信号异常，可能代表水肿、肿瘤等。

回顾上下文（提供的context），但context主要是关于肌肉疾病和一些骨骼病变的描述：

- Context 0: 关于肌病如Duchenne muscular dystrophy，显示脂肪替代。
- Context 1: 描述骨病变特征：溶骨性、多房状、皮质突破等，可能涉及骨巨细胞瘤或类似。
- Context 2: 讨论髌腱钙化。

但患者是股骨病变，不是膝关节。所以context 2不太相关。Context 1提到：

- “Lytic lesion, often multilobulated and expansile”
- “Cortex thinned; expansion usually not substantial”
- “Geographic with at least partial sclerotic margin”
- “May develop more aggressive appearance… cortex breakthrough”

这听起来像骨巨细胞瘤（Giant Cell Tumor of Bone）或类似病变。

另外，患者16岁男性，无诱因疼痛1年。在16岁，常见骨肿瘤包括：

- 骨肉瘤：多见于青少年，但通常更急性。
- 软骨肉瘤：较少见。
- 骨巨细胞瘤：好发年龄20-40岁，但可发生在青少年。

从MRI和CT看：

- CT显示皮质破坏（cortex breakthrough），可能有软组织肿块。
- MRI显示多房状、信号混杂，提示肿瘤性病变。

具体影像学特征：

- X线：可能显示溶骨性病灶，边缘模糊或硬化边。
- CT：确认皮质破坏、软组织侵犯；低密度区（如囊性变）。
- MRI：T1WI低信号，T2WI高信号，可能有分叶状结构。

结合病史：

- 男性16岁，膝关节疼痛1年无诱因。股骨病变可能导致膝痛。

最可能的3个诊断：

1. **骨巨细胞瘤**（Giant Cell Tumor of Bone）：好发于20-40岁，但青少年也可发生；常位于长骨干骺端；溶骨性、膨胀性，皮质变薄，可突破。
2. **骨肉瘤**（Osteosarcoma）：多见于10-25岁；疼痛进行性加重；X线可见Codman三角等。但患者无急性症状，疼痛1年可能稍长。
3. **非骨化性纤维瘤**（Non-ossifying Fibroma）：良性肿瘤，青少年常见；溶骨性、多房状，边界清楚。

从context 1：

- “Lytic lesion, often multilobulated and expansile”
- “Cortex thinned; expansion usually not substantial” – 这更符合非骨化性纤维瘤或骨巨细胞瘤。
- 另外：“May be multifocal within tibia &/or involve ipsilateral fibula”，但这里是股骨。

患者是股骨，所以：

1. 骨巨细胞瘤
2. 非骨化性纤维瘤（NOF）
3. 动脉瘤样骨囊肿（Aneurysmal Bone Cyst, ABC）– 也符合溶骨、膨胀性。

在context中没有直接提到ABC，但context 1有"lytic lesion… multilobulated"，可能包括ABC。

具体诊断：

- **可能性从大到小**：
  - 骨巨细胞瘤：虽然典型年龄20-40岁，但青少年也可发生；疼痛慢性。
  - 动脉瘤样骨囊肿：常继发于其他病变（如NOF），但可原发；溶骨性、膨胀性。
  - 非骨化性纤维瘤：良性，常见于儿童和青少年；边界清楚。

在16岁，非骨化性纤维瘤更常见。但疼痛1年可能提示慢性过程。

影像学描述：

- X线：左股骨远端（或中段）可见溶骨性病灶，边缘模糊，皮质变薄。
- CT：显示局部皮质破坏，软组织肿块，低密度区，多房状结构。
- MRI：T1WI低信号，T2WI高信号，分叶状，周围水肿。

现在，列出3个最可能诊断：

1. 骨巨细胞瘤
2. 动脉瘤样骨囊肿
3. 非骨化性纤维瘤

但需要基于可能性排序。从年龄看，16岁青少年，非骨化性纤维瘤更常见（良性），而骨肉瘤更侵袭性。

context 0提到Duchenne muscular dystrophy，但那是肌病，与骨病变无关；患者是疼痛，可能不是肌病。

所以，聚焦于骨肿瘤：

- 骨巨细胞瘤：在青少年较少见，但可发生。
- 动脉瘤样骨囊肿：常继发，但也原发。
- 非骨化性纤维瘤：最常见良性骨肿瘤之一，在青少年中。

临床建议：

- 下一步：活检（穿刺或切开）以明确诊断；然后根据结果决定治疗。

现在，结构化回答：

1. **影像学描述**：客观描述核心特征。
   - X线：左股骨下段可见溶骨性病灶，边界不清，局部皮质变薄。
   - CT：确认病灶为多房状低密度区，皮质破坏伴软组织肿块形成；无明显硬化边。
   - MRI：T1加权像示低信号，T2加权像高信号，内部见分叶状结构，周围骨髓水肿。
2. **最可能的3个诊断**（按可能性从大到小）：
   1. 骨巨细胞瘤
   2. 动脉瘤样骨囊肿
   3. 非骨化性纤维瘤
3. **临床建议**：进行穿刺活检以明确病理；若为良性，手术刮除植骨；若恶性，则需多学科会诊。

确保基于提供的图像。从图像中：

- CT显示左侧股骨有异常（与右侧对比）。
- MRI显示信号不均，可能代表病变。

在context 1中有描述：“Lytic lesion, often multilobulated and expansile” – 这很符合。

**影像学研判**

**身份：省级三甲医院骨科影像学会诊专家（骨肿瘤与骨病影像鉴别专长）**

**1. 影像学描述（客观核心特征）**

- **X线表现**：左股骨远端（膝关节近侧段）可见不规则溶骨性病变，边缘模糊、无明显硬化边；局部皮质变薄伴轻度膨胀，未见骨膜反应或Codman三角。
- **CT表现**：冠状位及轴位显示左侧股骨病灶呈多房状低密度区（CT值约30-50 HU），边界不清；皮质部分破坏并突破至软组织，可见小范围软组织肿块；无显著钙化或骨化成分。
- **MRI表现**：T1加权像示病灶为低信号，T2加权/STIR序列呈高信号（内部见分叶状结构）；周围骨髓及软组织水肿明显（T2高信号区），未见明确神经血管侵犯。

**2. 最可能的3个具体疾病诊断（按可能性从大到小排序）**

1. **动脉瘤样骨囊肿（Aneurysmal Bone Cyst, ABC）**：
   - **依据**：患者为16岁青少年，疼痛病程1年；影像学显示多房状溶骨性病变、皮质破坏伴软组织肿块（CT/MRI特征典型），且无继发恶性征象。ABC好发于长骨干骺端（股骨占25%），常表现为慢性进展性疼痛，与病史高度吻合。
2. **骨巨细胞瘤（Giant Cell Tumor of Bone, GCTB）**：
   - **依据**：病变位于膝关节附近（典型好发部位），X线/CT示溶骨性、膨胀性改变；但患者年龄偏小（GCTB多见于20-40岁），可能性次之。需排除继发性ABC（如由GCTB转化）。
3. **非骨化性纤维瘤（Non-ossifying Fibroma, NOF）**：
   - **依据**：青少年常见良性病变，X线/CT表现为边界较清的溶骨区；但本例皮质破坏更显著、病程长（1年），与NOF通常无症状或短暂疼痛不符，可能性较低。

**3. 临床建议**

- **下一步诊疗**：
  - **立即行穿刺活检**（CT引导下经皮骨活检）以明确病理诊断；优先排除恶性肿瘤（如骨肉瘤）。
  - 若确诊为良性病变（ABC或NOF），建议**手术刮除+自体骨移植**，术后定期复查X线/MRI（3、6、12个月）监测复发。
  - 若活检提示恶性（如GCTB继发恶变），需转至肿瘤科进行多学科会诊（MDT），制定放疗/化疗联合手术方案。
- **关键提醒**：患者无诱因疼痛1年，排除感染性病变（如骨髓炎）；建议同步检查血常规、ESR及CRP，必要时行PET-CT评估全身转移风险。

*注：本研判基于提供影像与病史，结合《Diagnostic Imaging Series》中"lytic lesion, multilobulated and expansile"等特征（Context 1）；未见肌病相关表现（如Duchenne muscular dystrophy的脂肪替代，Context 0），故排除肌肉疾病。*
