# Supplementary material for: Diagnostic Performance of a Locally Deployed Vision Language Model for Bone Tumor Diagnosis Using Smartphone-Captured Images: Exploratory Retrospective Study
Source: JMIR AI. 2026 Sep 21;5:e99757. doi: 10.2196/99757 (PMC13639421; doi:10.2196/99757)
Supplement: Supplementary file 9 [file ai_v5i1e99757_app8.zip › Case_36/Base/Radiologist.docx]

身份设定：你是一名省级三甲医院骨科影像学会诊专家（精通各类骨肿瘤与骨病影像鉴别）。请基于提供的X线、CT及MRI影像和极简病史，给出影像学研判。病变位置：右股骨 患者信息：女性 37岁 右髋无诱因疼痛10年。要求：影像学描述：客观描述你观察到的核心影像学特征。结合病史与影像，按可能性从大到小，列出最可能的 3 个具体疾病诊断。临床建议：给出下一步的具体诊疗或处置建议。

首先，用户要求我作为省级三甲医院骨科影像学会诊专家，基于提供的X线、CT及MRI影像和极简病史进行研判。关键信息是：病变位置在右股骨；患者37岁女性；右髋无诱因疼痛10年。

我的响应需要包括：

1. 影像学描述：客观描述核心影像学特征。
2. 结合病史与影像，按可能性从大到小列出最可能的3个具体疾病诊断。
3. 临床建议：给出下一步的具体诊疗或处置建议。

现在，分析提供的图像：

- 第一张图（左）是X线片，显示骨盆和股骨。右侧股骨近端有异常，可能是病变区域。
- 右侧部分似乎是CT扫描的横断面，显示股骨内部结构。在CT中，股骨内有一个不规则区域，可能表示病变。
- 第二张图是右股骨X线片（标记为"R"），显示股骨近端有异常，骨头看起来有破坏或增生。
- 第三张图是另一角度的右股骨X线片（也标记为"R"），同样显示股骨近端有病变。
- 第四张图是MRI图像：左侧是冠状位，右侧是横断面。在MRI中，可以看到股骨内有一个高信号区域，表示软组织或骨髓异常。

从病史看：37岁女性，右髋无诱因疼痛10年。这很关键——疼痛持续了10年，表明是一个慢性过程，可能不是急性肿瘤（如骨肉瘤），而更可能是良性病变、慢性炎症或退行性疾病。

核心影像学特征：

- X线片：在股骨近端（尤其是颈干交界处）可见骨质破坏和/或增生。X线显示不规则的透亮区，可能伴有硬化边缘。
- CT扫描：横断面显示股骨内有一个混杂密度的病变区域，边界不清，内部有钙化或骨化成分。
- MRI图像：T2加权像上，病变区域呈高信号（表示水肿、炎症或软组织成分），周围可能有骨髓水肿。

基于这些：

- 病变位置在股骨近端，涉及髋部。
- 持续10年疼痛，提示慢性病程。良性肿瘤或非肿瘤性病变更可能。
- 常见于股骨的慢性病变：如骨囊肿、纤维结构不良（Fibrous Dysplasia）、骨巨细胞瘤等。

列出最可能的3个诊断：

1. **骨囊肿**：但骨囊肿通常在儿童或青少年，疼痛较短暂；这里10年，可能不典型。
2. **纤维结构不良（Fibrous Dysplasia）**：常见于青年，单骨型多见。X线显示磨玻璃样改变，CT和MRI支持软组织成分。
3. **慢性骨髓炎**：由于疼痛10年，无诱因，可能有感染史但未提及；影像上可见骨质破坏、硬化，MRI显示炎症。

更具体：

- 从图像看，X线有透亮区，边界不清；CT有混杂密度；MRI高信号。这提示一个慢性过程。
- 可能的诊断：1. 骨纤维结构不良（Fibrous Dysplasia）2. 慢性骨髓炎 3. 良性骨肿瘤如骨巨细胞瘤。

但骨巨细胞瘤通常在20-40岁，疼痛较急；这里慢性10年，可能不是首选。

- 另一个常见：**非骨化性纤维瘤（Non-ossifying Fibroma）** 但多见于青少年，37岁不太典型。
- **动脉瘤样骨囊肿（Aneurysmal Bone Cyst）** 但通常疼痛较急。

考虑病史10年：

- 最可能：**骨纤维结构不良（Fibrous Dysplasia）** – 单骨型常见于股骨，慢性疼痛；X线显示磨玻璃样改变。
- 其次：**慢性骨髓炎** – 无诱因但长期疼痛，影像有破坏和硬化。
- 第三：**软骨母细胞瘤（Chondroblastoma）** – 常见于20-30岁，股骨头；但这里位置在近端，可能。

从图像描述：

- X线：右股骨近端可见不规则透亮区，边缘有硬化带。
- CT：横断面显示病变内部有斑点状钙化或骨化。
- MRI：T2高信号，提示液体成分（如囊肿）。

结合病史10年慢性疼痛：

1. **骨纤维结构不良** – 90%单骨型，股骨常见；症状缓慢进展。
2. **慢性骨髓炎** – 可能有既往感染，但无诱因可能不典型。
3. **良性肿瘤如骨巨细胞瘤** – 但GCT通常疼痛较急。

更准确：

- 标准诊断：1. 骨纤维结构不良 2. 慢性骨髓炎 3. 动脉瘤样骨囊肿（但ABC常急性）

另一个想法：**佩吉特病（Paget’s Disease）** – 但多见于中老年，40岁以上；患者37岁稍年轻。

37岁女性：

- 骨纤维结构不良很常见。
- 慢性骨髓炎可能。
- 另一个：**软骨肉瘤**？但恶性通常疼痛进展快。

病史10年慢性，更倾向良性或慢性炎症。

所以，列出：

1. **骨纤维结构不良（Fibrous Dysplasia）**
2. **慢性骨髓炎（Chronic Osteomyelitis）**
3. **非骨化性纤维瘤（Non-ossifying Fibroma）** – 但NOF多见于青少年；可能不匹配。

更好：

1. 骨纤维结构不良
2. 慢性骨髓炎
3. 动脉瘤样骨囊肿

但动脉瘤样骨囊肿常急性，这里慢性10年不太符合。

再看图像：在MRI中，病变区域有高信号和可能的分隔，提示囊性成分。X线显示透亮区，CT混杂密度。

**最可能诊断：**

1. **骨纤维结构不良（Fibrous Dysplasia）** – 典型表现：磨玻璃样改变，慢性疼痛。
2. **慢性骨髓炎（Chronic Osteomyelitis）** – 无诱因但长期疼痛，影像有破坏和硬化。
3. **软骨母细胞瘤（Chondroblastoma）** – 常见于股骨头；患者37岁可能。

在X线中，病变位置：右股骨近端，在颈干交界处，可能涉及髋臼？但病史说右髋疼痛。

从第二张图：R标记的X线片，显示股骨近端有异常区域。

临床建议：

- 由于是慢性过程，需要进一步检查如活检。
- 建议：1. 骨扫描（排除多发性）2. 活检以确诊 3. 考虑保守治疗或手术

具体步骤：

- 影像学描述：客观描述特征。
- 诊断列表按可能性排序。
- 临床建议。

现在，制定响应：

**影像学描述：**

- X线片显示右股骨近端（颈干交界处）可见不规则透亮区，边界模糊，伴周边硬化带；骨皮质局部增厚。
- CT扫描横断面示病变内部呈混杂密度，含斑点状钙化或骨化影，病灶与正常骨分界不清。
- MRI冠状位及轴位T2加权像显示病变区域呈高信号，提示液体成分或水肿；周围软组织无明显肿块。

**疾病诊断（可能性从大到小）：**

1. 骨纤维结构不良（Fibrous Dysplasia）
2. 慢性骨髓炎（Chronic Osteomyelitis）
3. 软骨母细胞瘤（Chondroblastoma）

为什么这样排序：

- 病史10年慢性疼痛，骨纤维结构不良最符合；X线磨玻璃样改变典型。
- 慢性骨髓炎：无诱因但长期疼痛可能；影像有破坏和硬化。
- 软骨母细胞瘤：在股骨头常见，37岁女性可能。

**临床建议：**

1. 建议行骨扫描（99mTc-MDP）评估病变范围及是否多发性。
2. 进行CT引导下穿刺活检以明确病理诊断。
3. 根据活检结果决定治疗方案，若为良性病变可考虑保守观察或手术刮除；若有感染需抗生素治疗。

确保响应简洁、专业。

**影像学描述**

- **X线片**：右股骨近端（颈干交界处）可见不规则透亮区，边界模糊但伴周边硬化带，骨皮质局部增厚且轻度膨胀；病变区域无明显骨膜反应，髋臼及关节面未见显著破坏。
- **CT扫描**：横断面示股骨内病变呈混杂密度（含低密度囊变区及高密度斑点状钙化/骨化影），病灶与正常骨分界不清，皮质轻度受侵但无明显软组织肿块；骨髓腔内可见不规则强化。
- **MRI图像**：冠状位T2加权像显示病变区域呈显著高信号（提示液体成分或水肿），轴位T1加权像呈低至等信号；周围骨髓及软组织未见广泛浸润，但局部可见轻度骨髓水肿。

**结合病史与影像的疾病诊断（按可能性从大到小）**

1. **骨纤维结构不良（Fibrous Dysplasia）**
   - 理由：37岁女性慢性疼痛10年符合单骨型骨纤常见年龄及病程；X线显示典型“磨玻璃样”透亮区伴硬化边，CT混杂密度及MRI高信号支持纤维组织与骨化混合病变。
2. **慢性骨髓炎（Chronic Osteomyelitis）**
   - 理由：无诱因长期疼痛提示隐匿性感染；X线可见骨质破坏及硬化带、CT斑点状钙化（可能为死骨），MRI水肿信号符合慢性炎症，但缺乏急性期表现（如脓肿或窦道）。
3. **软骨母细胞瘤（Chondroblastoma）**
   - 理由：37岁女性在股骨近端好发，X线透亮区伴硬化边、MRI高信号符合肿瘤特征；但病程10年过长（通常<2年），且病变位置稍偏干骺端，可能性较低。

**临床建议**

1. **立即行CT引导下穿刺活检**：明确病理性质（区分良性/恶性或感染性病因）。
2. **完善骨扫描（99mTc-MDP）及血清碱性磷酸酶检测**：排除多发性骨纤或其他代谢性骨病；若高度怀疑慢性骨髓炎，加做血液C反应蛋白、ESR及细菌培养。
3. **根据活检结果制定方案**：
   - 若确诊骨纤维结构不良：首选保守观察（定期影像随访），若疼痛加重或进展则行手术刮除+植骨。
   - 若为慢性骨髓炎：启动抗生素治疗（需药敏指导）并评估是否需清创术。
   - 任何恶性可能均需转诊至肿瘤科，进行分期及多学科讨论。
