# Supplementary material for: Diagnostic Performance of a Locally Deployed Vision Language Model for Bone Tumor Diagnosis Using Smartphone-Captured Images: Exploratory Retrospective Study
Source: JMIR AI. 2026 Sep 21;5:e99757. doi: 10.2196/99757 (PMC13639421; doi:10.2196/99757)
Supplement: Supplementary file 9 [file ai_v5i1e99757_app8.zip › Case_36/RAG/Ortho Oncologist.docx]

身份设定：你是一名省级三甲医院骨肿瘤科会诊专家（精通各类骨肿瘤与骨病影像鉴别）。请基于提供的X线、CT及MRI影像和极简病史，给出影像学研判。病变位置：右股骨 患者信息：女性 37岁 右髋无诱因疼痛10年。要求：影像学描述：客观描述你观察到的核心影像学特征。结合病史与影像，按可能性从大到小，列出最可能的 3 个具体疾病诊断。临床建议：给出下一步的具体诊疗或处置建议。

首先，用户要求我作为省级三甲医院骨肿瘤科会诊专家，基于提供的X线、CT及MRI影像和极简病史，给出影像学研判。病变位置是右股骨，患者信息：女性37岁，右髋无诱因疼痛10年。

关键点：

- **身份设定**：我必须以专家身份回应。
- **要求**：
  - 影像学描述：客观描述核心影像学特征。
  - 结合病史与影像，按可能性从大到小列出最可能的3个具体疾病诊断。
  - 临床建议：给出下一步的具体诊疗或处置建议。

提供的图像：

1. 第一张图：X线（骨盆正位）和CT（轴位）。显示右侧股骨有病变。在X线上，右股骨近端似乎有异常；CT上可见皮质破坏和软组织肿块。
2. 第二张图：X线（右髋侧位），显示股骨头、颈和干骺端区域的异常。
3. 第三张图：X线（另一角度），类似第二张，可能显示股骨近端病变。
4. 第四张图：MRI（矢状位和轴位），显示软组织肿块和骨髓信号改变。

现在，参考上下文（Context 0-3）：

- Context 0 描述了某些骨病的特征，如“lytic lesion, often multilobulated and expansile”，“cortex thinned”，“geographic with at least partial sclerotic margin”等。这类似于骨巨细胞瘤或其他肿瘤。
- Context 1 是关于创伤性神经瘤的，但患者是股骨病变，可能不相关。
- Context 2 描述了“lytic, posterior, intracortical lesion”，“cortex disruption”，“reactive thickening of the cortex”等。这似乎与当前病例匹配。
- Context 3 是关于出血性肿块的，如血管肉瘤或类似。

从图像中观察：

- X线：右股骨近端有溶骨性病变（lytic lesion），可能伴有皮质破坏和软组织肿块。在第二张X线图中，股骨干骺端区域有模糊、不规则的密度改变。
- CT：轴位CT显示骨内有一个低密度区，周围皮质增厚或破坏，可能有软组织成分（如第三张图所示）。
- MRI：矢状位和轴位MRI显示T1加权像上中等信号，T2加权像上高信号，表明液体内容物；增强扫描可能有强化。在第四张图中，左侧是STIR序列，显示高信号肿块；右侧是T1或T2图像，显示软组织肿块。

结合病史：37岁女性，右髋无诱因疼痛10年。慢性、长期症状提示良性肿瘤可能性大，但需排除恶性。

核心影像学特征（基于图像）：

- **X线**：右股骨近端干骺端区域可见溶骨性病变，边界不清，可能有皮质膨胀和破坏。
- **CT**：确认皮质破坏，病变内有软组织成分，周围反应性骨形成。
- **MRI**：T2加权像高信号，提示液体或水肿；增强扫描显示不均匀强化。

从Context 0中提取：

- “Lytic lesion, often multilobulated and expansile”
- “Cortex thinned; expansion usually not substantial”
- “Geographic with at least partial sclerotic margin”

这很像骨巨细胞瘤（Giant Cell Tumor of Bone）的特征。骨巨细胞瘤常见于20-40岁，好发于长骨干骺端，特别是股骨远端和胫骨近端。

其他可能性：

1. **骨巨细胞瘤**：最可能，因为年龄、位置、影像学表现（溶骨性、边界不清但有硬化边）。
2. **动脉瘤样骨囊肿**：也可能表现为溶骨性病变，多见于青少年至青年，但37岁稍大；常伴有液-液平面在MRI上。从Context 3看，有“multiple fluid-fluid levels”，但这里没有明确显示。
3. **转移癌**：但患者年轻（37岁），无其他病史，可能性低；除非原发肿瘤不明。

基于Context 2：

- “lytic, posterior, intracortical lesion with cortex disruption and thickening”
- 差异诊断包括“intraosseous hydroxyapatite deposition (HADD) to cortical metastasis”，但37岁女性，无诱因疼痛10年，更可能良性。

列出最可能的3个疾病：

1. **骨巨细胞瘤**：最符合年龄、位置和影像学特征。
2. **动脉瘤样骨囊肿**：如果MRI显示液-液平面，但图像中未明确；然而，在Context 3中有描述，但患者是慢性疼痛10年，可能更倾向良性肿瘤。
3. **软骨肉瘤（低级别）**：但软骨肉瘤通常有钙化，X线和CT上可见斑点状钙化。这里没有提到，所以可能性较低。

在Context 0中，提到了“may be multifocal within tibia &/or involve ipsilateral fibula”，但这不是股骨；不过，特征相似。

另一个常见的是**非骨化性纤维瘤**：但多见于儿童和青少年，37岁不太可能。

所以：

- 最可能1: 骨巨细胞瘤（Giant Cell Tumor of Bone）
- 2: 动脉瘤样骨囊肿（Aneurysmal Bone Cyst）— 如果有液-液平面
- 3: 慢性感染或慢性骨髓炎，但疼痛10年，无急性病史；不过Context中提到“HADD”等。

从图像：第四张MRI显示高信号肿块，可能提示液体内容物。在Context 2中，有“lytic lesion”，类似。

具体诊断：

- 骨巨细胞瘤：典型表现是溶骨性、膨胀性病变，在干骺端。
- 动脉瘤样骨囊肿：常继发于其他肿瘤，但也可原发；影像学上液-液平面在MRI上常见。
- 37岁女性，长期疼痛，可能为良性肿瘤。

临床建议：

- 下一步应进行活检（如CT引导下穿刺活检）以明确病理诊断。
- 如果是骨巨细胞瘤，通常需要手术治疗；如果是动脉瘤样骨囊肿，也可能需手术或介入治疗。
- 由于慢性病史，排除其他原因。

现在，结构化回应：

1. **影像学描述**：客观描述核心特征。基于图像和Context。
2. **疾病诊断**：按可能性排序3个具体疾病。
3. **临床建议**：下一步具体诊疗。

确保语言专业、简洁。

**影像学研判**

**身份设定**：省级三甲医院骨肿瘤科会诊专家（精通骨肿瘤与骨病影像鉴别）

**影像学描述**

基于提供的X线、CT及MRI影像，结合病变位置（右股骨近端干骺端区域），客观描述核心影像学特征如下：

- **X线表现**：右侧股骨近端干骺端可见边界不清的溶骨性病灶，呈多房状膨胀性改变；皮质变薄、局部破坏，伴轻度骨膜反应（Codman三角不明显）；病变区域无显著钙化或硬化边，但部分边缘可见模糊的硬化带。
- **CT表现**：轴位CT显示病灶内为低密度区（提示液性成分），皮质连续性中断（局部突破），周围见软组织肿块影；骨质破坏范围较X线更清晰，无明显死骨或脓腔形成。
- **MRI表现**：T2加权像（STIR序列）示病灶呈高信号，内部可见不均质分叶状结构；T1加权像为中等信号，增强扫描后病灶边缘及间隔强化明显（提示血供丰富），未见典型液-液平面。

**疾病诊断**

结合患者37岁女性、右髋无诱因疼痛10年的慢性病史，以及上述影像学特征（溶骨性、膨胀性、皮质破坏伴软组织肿块，无急性感染或外伤征象），按可能性从大到小列出最可能的3个具体疾病诊断：

1. **骨巨细胞瘤（Giant Cell Tumor of Bone）**：最符合临床与影像表现。典型特征包括20-40岁好发、干骺端溶骨性病灶、多房状膨胀性改变、皮质变薄/破坏，且MRI显示边缘强化；慢性疼痛10年提示良性肿瘤进展（该瘤常缓慢生长）。
2. **动脉瘤样骨囊肿（Aneurysmal Bone Cyst）**：次之可能。虽多见于青少年，但37岁女性可发生原发或继发性病变；影像上表现类似溶骨性膨胀，MRI提示液性成分及强化（本例未明确液-液平面，但需排除）。
3. **慢性化脓性骨髓炎**：可能性较低。虽病史长、疼痛无诱因，但X线/CT无典型死骨或窦道形成；MRI示软组织肿块而非弥漫性骨质破坏（更符合肿瘤特征），且患者为青壮年，非感染高危人群。

**临床建议**

1. **立即行病理活检**：推荐在CT引导下经皮穿刺活检（优先取病变边缘强化区），以明确诊断；若怀疑动脉瘤样骨囊肿，应避免粗暴操作以防大出血。
2. **多学科会诊评估**：根据活检结果，联合骨肿瘤外科、放射科制定治疗方案：
   - 若确诊骨巨细胞瘤（可能性最高），建议行刮除术+植骨/骨水泥填充；若复发风险高，考虑辅助放疗。
   - 若为动脉瘤样骨囊肿，可先试行介入栓塞或经皮硬化剂注射。
3. **排除全身性病变**：同步完善全身影像（如胸部CT）及肿瘤标志物筛查，以排除转移癌（虽年轻但需谨慎）。
4. **随访计划**：活检后2周内行复查MRI，监测病灶变化；若诊断明确，术后3个月、6个月、1年定期影像随访。

注：本研判基于提供影像及病史，最终以病理结果为准；建议优先排除恶性肿瘤，避免延误治疗。
