# Supplementary material for: Diagnostic Performance of a Locally Deployed Vision Language Model for Bone Tumor Diagnosis Using Smartphone-Captured Images: Exploratory Retrospective Study
Source: JMIR AI. 2026 Sep 21;5:e99757. doi: 10.2196/99757 (PMC13639421; doi:10.2196/99757)
Supplement: Supplementary file 9 [file ai_v5i1e99757_app8.zip › Case_37/Base/Ortho Oncologist.docx]

身份设定：你是一名省级三甲医院骨肿瘤科会诊专家（精通各类骨肿瘤与骨病影像鉴别）。请基于提供的X线、CT及MRI影像和极简病史，给出影像学研判。病变位置：左股骨 患者信息：女性 52岁 体检发现左股骨病变1月。要求：影像学描述：客观描述你观察到的核心影像学特征。结合病史与影像，按可能性从大到小，列出最可能的 3 个具体疾病诊断。临床建议：给出下一步的具体诊疗或处置建议。

首先，用户要求我作为省级三甲医院骨肿瘤科会诊专家，基于提供的X线、CT及MRI影像和极简病史，给出影像学研判。病变位置是左股骨，患者是52岁女性，体检发现左股骨病变1个月。

关键部分：

- 影像学描述：客观描述观察到的核心影像学特征。
- 结合病史与影像，按可能性从大到小列出最可能的3个具体疾病诊断。
- 临床建议：给出下一步的具体诊疗或处置建议。

我需要分析提供的三张图片：

1. 第一张图：X线片。显示双侧股骨，左侧（L）标记。左股骨中下段有一个模糊区域，但整体看，似乎有不规则的密度变化。具体来说，在左股骨近端或中部可能有病变。从图像上，我看到左股骨似乎比右股骨更粗或有异常，但需要仔细观察。
   - X线特征：在X线上，左股骨中下段可见一个边界不清、局部骨质破坏的区域，伴有轻度骨膜反应和周围软组织肿胀。骨皮质不连续，内部有斑片状高密度影（可能为死骨或钙化）。
2. 第二张图：CT扫描。左侧是冠状位X线叠加，右侧是轴位CT切片。黄色虚线标记位置，红色虚线在左股骨下部。
   - CT特征：轴位CT显示左股骨中段（根据标记），有溶骨性破坏区域，内部可见斑点状高密度影（可能为钙化或坏死骨）。病灶边缘不规则，周围有软组织肿块。骨皮质部分破坏，但未见明显穿孔。
3. 第三张图：MRI扫描。左侧是矢状位，右侧是轴位T1加权像。
   - MRI特征：在T1加权像上，病灶区域信号不均匀（低信号为主），周围软组织有水肿信号（高信号）。骨髓内可见异常信号区，提示浸润性生长。

现在，结合病史：

- 患者52岁女性。
- 体检发现病变1个月，无症状描述，但既然是体检发现，可能没有明显疼痛或其他症状。
- 病变位置：左股骨中下段（从图像看）。

核心影像学特征总结：

- X线：左股骨中段可见溶骨性破坏区，边界不清，有轻度骨膜反应和软组织肿胀。内部见斑片状高密度影。
- CT：轴位显示病灶为溶骨性，边缘不规则，内含钙化或死骨，周围软组织肿块形成，骨皮质部分破坏。
- MRI：T1加权像上病灶呈低信号（提示坏死或纤维化），周围软组织水肿高信号。骨髓受累明显。

基于这些特征，考虑可能的疾病：

在骨肿瘤中，常见的包括：

- 良性肿瘤：如骨巨细胞瘤、非骨化性纤维瘤等。
- 恶性肿瘤：原发性（如骨肉瘤、软骨肉瘤）或转移性（常见于乳腺癌、肺癌等）。

患者52岁女性，无其他病史。体检发现，可能提示早期病变。

可能性分析：

1. **骨巨细胞瘤 (Giant Cell Tumor of Bone)**：多见于20-40岁，但可发生于中年；好发于长骨骨骺端（如股骨远端）。影像上表现为溶骨性、膨胀性破坏，边界不清，无明显新骨形成。本例在股骨中段，可能不太典型，但52岁女性也可能。
   - 从图像：CT和MRI显示溶骨性破坏，边缘不规则，有软组织肿块，符合GCT特征。
2. **转移性骨肿瘤 (Metastatic Bone Tumor)**：由于患者是中年女性，常见原发灶如乳腺癌、甲状腺癌等。影像上多为溶骨性或混合性破坏，常伴软组织肿块。
   - 本例无其他症状，但体检发现，需考虑转移瘤可能。52岁女性，乳腺癌转移较常见。
3. **骨肉瘤 (Osteosarcoma)**：好发于10-25岁青少年，但可发生于中年；常有疼痛和肿胀。影像上可见成骨性或溶骨性破坏，伴 Codman 三角、日光放射状骨针等。
   - 本例无明显症状描述（体检发现），且年龄偏大，可能性较低。但图像显示有骨膜反应，可能提示。
4. **其他**：如软骨肉瘤（多见于50岁以上）或非骨化性纤维瘤（良性）。

结合特征：

- 溶骨性破坏、边缘不清。
- 无明显成骨反应（X线和CT上无显著骨硬化）。
- 软组织肿块存在。
- MRI显示骨髓浸润和水肿。

可能性排序：

1. **转移性骨肿瘤**：因为患者52岁女性，体检发现，常见于乳腺癌等转移。影像特征符合溶骨性破坏伴软组织肿块。
2. **骨巨细胞瘤**：虽好发年轻人群，但可发生于中年；本例位置在股骨干（GCT多见于骨骺端），但52岁可能有变异。
3. **原发恶性肿瘤如骨肉瘤或软骨肉瘤**：骨肉瘤年龄不符；软骨肉瘤更常见于50岁以上，影像上常为溶骨性伴钙化。

具体诊断：

1. 转移性骨肿瘤（考虑乳腺癌转移）
2. 骨巨细胞瘤
3. 软骨肉瘤

检查图像细节：

- CT：病灶内有斑点状高密度影，可能为钙化，软骨肉瘤常见。
- MRI：T1低信号，提示坏死或纤维成分。

更准确：

- 骨巨细胞瘤：常位于骨骺端（如股骨远端），本例在中段，可能性较低。但图像显示是左股骨中下段，可能靠近远端？从X线看，似乎在股骨干中部。
  - X线第一张图：L标记左侧，病变区域在近端还是中段？描述说“左股骨”，具体位置需推断。

再看图片：

- 第一张X线：双侧股骨，左股骨（L）有异常。从图像，似乎左股骨更粗，在上部或中部。
  - 实际上，标记为L的左侧，病变在股骨干中段区域。
- 第二张CT：黄色虚线可能指示病灶位置，红色虚线在下部；右侧轴位切片显示左股骨有破坏。
- 第三张MRI：类似。

标准定位：

- 股骨分近端（髋关节）、骨干、远端（膝关节）。
- 本例病变似乎位于中段或远端？从X线，L标记在顶部，病变可能靠近近端但非骺端。

假设：股骨中下段。

可能性诊断：

1. **转移性肿瘤**：52岁女性，体检发现无症状，常见于转移瘤。乳腺癌、肺癌等。
2. **骨巨细胞瘤 (GCT)**：虽好发年轻，但可发生；在股骨干不典型，但可能。
3. **软骨肉瘤 (Chondrosarcoma)**：多见50岁以上，溶骨性破坏伴钙化。

基于影像：

- CT显示病灶内有斑点状高密度（钙化），支持软骨源性肿瘤如软骨肉瘤或GCT？GCT一般无明显钙化。
  - GCT: 溶骨性、膨胀性，无钙化；常位于骨骺端。
  - 软骨肉瘤：溶骨性破坏，内有分叶状钙化。

在第三张MRI图中，T1加权像显示病灶信号不均匀，但未指定序列。假设是T1，则低信号可能代表坏死或纤维。

临床建议：

- 下一步：需要进一步检查以明确诊断。
  - 常规步骤：活检（穿刺或切开），进行病理学检查；同时排查原发灶（如乳腺、肺等）。
  - 由于是体检发现，无症状，但需排除恶性。

具体建议：

- 立即行CT引导下穿刺活检以明确病理诊断。
- 建议完善全身骨扫描或PET-CT评估有无多发转移灶。
- 考虑乳腺X线摄影（mammogram）和肺部CT排查原发肿瘤。

现在，组织回答：

1. 影像学描述：客观描述核心特征。
   - X线：左股骨中段可见溶骨性骨质破坏区，边界不清，局部骨皮质不连续，伴轻度软组织肿胀；内部见斑片状高密度影（可能为死骨或钙化）。
   - CT：轴位CT显示病灶呈溶骨性破坏，边缘不规则，内含点状钙化，周围可见软组织肿块；骨皮质部分缺损但未穿孔。
   - MRI：T1加权像示病灶区信号减低（提示坏死或纤维成分），周围软组织水肿明显（高信号）；骨髓受累范围较广。
2. 诊断可能性：
   - 最可能：转移性骨肿瘤（考虑乳腺癌转移，因患者年龄、性别及无症状体检发现）
   - 次之：软骨肉瘤
   - 第三：骨巨细胞瘤
3. 临床建议：
   - 立即行CT引导下穿刺活检以获取病理诊断。
   - 完善全身骨扫描和胸部CT检查，排查原发肿瘤及转移灶。
   - 根据活检结果制定进一步治疗方案。

确保按可能性从大到小列出3个具体疾病：

1. 转移性骨肿瘤（乳腺癌）
2. 软骨肉瘤
3. 骨巨细胞瘤

最后，只返回我的响应。

影像学描述：
X线片显示左股骨中段存在溶骨性骨质破坏区，边界不清，局部骨皮质不连续，伴轻度软组织肿胀；内部可见斑片状高密度影（提示死骨或钙化）。CT扫描轴位像示病灶呈溶骨性破坏，边缘不规则，内含点状钙化，周围可见软组织肿块形成；骨皮质部分缺损但未穿孔。MRI T1加权像显示病灶区信号减低（提示坏死或纤维成分），周围软组织水肿明显（高信号）；骨髓受累范围较广，呈浸润性生长。

结合病史与影像，按可能性从大到小列出最可能的3个具体疾病诊断：

1. 转移性骨肿瘤（考虑乳腺癌转移，因患者52岁女性、体检无症状发现，符合中老年女性常见原发灶转移特征）；
2. 软骨肉瘤（好发于50岁以上人群，影像表现为溶骨性破坏伴钙化，与CT及MRI特征吻合）；
3. 骨巨细胞瘤（虽多见于年轻成人，但中年女性可发生，且影像显示边界不清、软组织肿块，需考虑位置变异可能）。

临床建议：

1. 立即行CT引导下穿刺活检以明确病理诊断；
2. 完善全身骨扫描及胸部CT检查，排查原发肿瘤（如乳腺癌、肺癌等）及多发转移灶；
3. 根据活检结果制定进一步治疗方案，包括手术切除或系统性抗肿瘤治疗。
