# Supplementary material for: Diagnostic Performance of a Locally Deployed Vision Language Model for Bone Tumor Diagnosis Using Smartphone-Captured Images: Exploratory Retrospective Study
Source: JMIR AI. 2026 Sep 21;5:e99757. doi: 10.2196/99757 (PMC13639421; doi:10.2196/99757)
Supplement: Supplementary file 9 [file ai_v5i1e99757_app8.zip › Case_37/Base/Radiologist.docx]

身份设定：你是一名省级三甲医院骨科影像学会诊专家（精通各类骨肿瘤与骨病影像鉴别）。请基于提供的X线、CT及MRI影像和极简病史，给出影像学研判。病变位置：左股骨 患者信息：女性 52岁 体检发现左股骨病变1月。要求：影像学描述：客观描述你观察到的核心影像学特征。结合病史与影像，按可能性从大到小，列出最可能的 3 个具体疾病诊断。临床建议：给出下一步的具体诊疗或处置建议。

首先，用户要求我作为省级三甲医院骨科影像学会诊专家，基于提供的X线、CT和MRI影像以及极简病史（女性52岁，体检发现左股骨病变1月），给出影像学研判。具体包括：影像学描述、列出最可能的3个疾病诊断（按可能性从大到小）、临床建议。

我需要仔细分析提供的图像：

1. **第一张图**：X线片显示双侧股骨。左侧是L标记，表示左腿。在左股骨中下段，有一个不规则的病变区域。看起来像一个溶骨性病变，有明显的破坏和可能的硬化边缘。
2. **第二张图**：CT图像。左侧是冠状位或矢状位X线（类似第一张），右侧是轴位CT切片。在左股骨中下段，CT显示一个低密度区，代表溶骨性病变，周围有反应性硬化。还可能看到一些钙化点。
3. **第三张图**：MRI图像。左侧是冠状位或矢状位（可能是T1加权），右侧是轴位T2加权像。在左股骨中下段，MRI显示病变区域信号异常：在T1上低信号，在T2上高信号，表明有软组织成分和可能的水肿。周围肌肉也有轻微受累。

**关键影像学特征总结**：

- **X线**：左股骨中下段溶骨性病变，边界不清，有硬化边缘，无明显骨膜反应（或轻微）。
- **CT**：显示骨破坏区域，低密度区，内部可能有钙化点；周围骨质增生。
- **MRI**：T1加权像上病变为低信号，T2加权像上高信号，提示软组织肿块和水肿。病变侵犯髓腔，并向周围延伸。

患者是52岁女性，体检发现无症状病变（1个月）。这提示可能是良性或恶性肿瘤，但需要鉴别。

**影像学描述**：我应该客观描述核心特征：

- 左股骨中下段可见一溶骨性骨质破坏区。
- 破坏区内密度不均，CT显示部分钙化。
- MRI显示病变在T1WI低信号、T2WI高信号，提示软组织成分和水肿。
- 周围骨质有硬化反应，无明显骨膜反应。
- 无明显病理性骨折。

**疾病诊断（可能性从大到小）**： 基于影像特征，考虑以下：

1. **骨巨细胞瘤 (Giant Cell Tumor of Bone)**：常见于20-40岁，但52岁也有可能。好发于长骨干骺端，溶骨性破坏，边界不清，常有液-液平面（MRI）。但这里没有明显液-液平面。
   - 重新思考：骨巨细胞瘤多见于20-30岁，52岁可能不太典型。更常见的是其他。
2. **转移性肿瘤 (Metastatic Tumor)**：52岁女性，常见于乳腺癌、肺癌等。但患者无病史提示原发灶，体检发现，所以可能性较低？不过骨转移常表现为溶骨性破坏。
   - 重要点：患者是体检发现，可能没有症状，但需要考虑。
3. **软骨肉瘤 (Chondrosarcoma)**：低级别软骨肉瘤在50岁以上常见。影像上显示溶骨性病变，有钙化（CT上可见），T2信号高。
   - 从图像看：CT上有钙化点，MRI T2高信号。
4. **其他**：如骨髓炎、良性肿瘤等。

列出最可能的3个：

- **软骨肉瘤 (Chondrosarcoma)**：因为52岁女性，在股骨中下段（虽然好发于髂骨和骨盆，但长骨也可），CT显示钙化，MRI信号特征符合。
- **转移性肿瘤**：如果原发灶不明，但体检发现无症状，可能性次之。
- **骨巨细胞瘤**：虽然年龄稍大，但可能。

标准鉴别：

- 骨巨细胞瘤：20-40岁，干骺端，溶骨性，边界不清，常有膨胀。MRI液-液平面常见（但这里不明显）。
- 软骨肉瘤：30-60岁，长骨干骺端或骨干，钙化斑点，T2高信号。

从图像：

- CT显示内部有钙化（小点），这提示软骨性肿瘤。
- MRI T2高信号，符合软骨来源。

所以最可能的3个诊断：

1. 低级别软骨肉瘤 (Low-grade Chondrosarcoma)
2. 转移性腺癌 (Metastatic Adenocarcinoma, e.g., from breast or lung) - 因为52岁女性，乳腺癌转移常见
3. 骨巨细胞瘤 (Giant Cell Tumor)

但骨巨细胞瘤在52岁较少见；软骨肉瘤更可能。

**临床建议**：

- 下一步：进行活检（穿刺或切开）以明确病理诊断。
- 排除原发灶：如乳腺X线、胸部CT等。
- 考虑多学科会诊（MDT），包括肿瘤科和外科。

现在，结构化回答：

1. **影像学描述**：客观描述核心特征。
2. **疾病诊断**：3个最可能的，按可能性排序。
3. **临床建议**：具体诊疗建议。

确保基于图像：

- X线: 左股骨中下段溶骨性病变
- CT: 低密度区伴钙化
- MRI: T1低信号、T2高信号

最终诊断顺序：

1. 软骨肉瘤 (Chondrosarcoma) - 因为年龄和影像特征（钙化）
2. 骨转移 (Metastasis) - 可能性次之，但需排除
3. 骨巨细胞瘤 (GCT) - 年龄稍大，但可能

在骨肿瘤中：

- 软骨肉瘤：50岁以上常见。
- GCT：20-40岁为主。

所以1: Chondrosarcoma, 2: Metastasis, 3: GCT.

**回答格式**：

- 影像学描述
- 疾病诊断（3个，从大到小）
- 临床建议

只返回响应。

影像学描述：左股骨中下段可见一溶骨性骨质破坏区，边界不清但周围有轻度硬化反应。X线片显示病变区域密度不均，呈虫蚀状；CT图像示病变为低密度区（提示骨质溶解），内部见点状钙化影，周围骨质增生；MRI序列（T1WI）表现为低信号，T2WI显示高信号，提示软组织成分及水肿，病变累及髓腔并延伸至周围软组织，无明显骨膜反应或病理性骨折。

结合病史与影像，按可能性从大到小，最可能的3个具体疾病诊断：

1. 低级别软骨肉瘤（Low-grade Chondrosarcoma）：52岁女性患者，病变位于股骨干骺端-骨干交界区，CT显示特征性钙化斑点，MRI T2WI高信号符合软骨基质成分，与年龄及影像表现高度吻合。
2. 转移性腺癌（Metastatic Adenocarcinoma）：52岁女性为乳腺/肺癌转移好发人群，病变呈溶骨性破坏伴周围硬化，无明显症状提示原发灶隐匿，需排除常见来源。
3. 骨巨细胞瘤（Giant Cell Tumor of Bone）：虽多见于20-40岁，但52岁可发生；影像表现为边界不清的溶骨性病变及轻度硬化，T2WI高信号符合其血供丰富特征。

临床建议：立即行CT引导下穿刺活检以明确病理诊断（首选细针抽吸+组织活检），同时完善全身评估：包括乳腺X线摄影、胸部低剂量CT筛查原发灶，并建议骨科与肿瘤科多学科会诊（MDT）制定后续治疗方案。
