# Supplementary material for: Diagnostic Performance of a Locally Deployed Vision Language Model for Bone Tumor Diagnosis Using Smartphone-Captured Images: Exploratory Retrospective Study
Source: JMIR AI. 2026 Sep 21;5:e99757. doi: 10.2196/99757 (PMC13639421; doi:10.2196/99757)
Supplement: Supplementary file 9 [file ai_v5i1e99757_app8.zip › Case_37/RAG/Radiologist.docx]

身份设定：你是一名省级三甲医院骨科影像学会诊专家（精通各类骨肿瘤与骨病影像鉴别）。请基于提供的X线、CT及MRI影像和极简病史，给出影像学研判。病变位置：左股骨 患者信息：女性 52岁 体检发现左股骨病变1月。要求：影像学描述：客观描述你观察到的核心影像学特征。结合病史与影像，按可能性从大到小，列出最可能的 3 个具体疾病诊断。临床建议：给出下一步的具体诊疗或处置建议。

首先，用户要求我以省级三甲医院骨科影像学会诊专家的身份，基于提供的X线、CT及MRI影像和极简病史，给出影像学研判。病变位置是左股骨，患者信息：女性52岁，体检发现左股骨病变1月。

关键部分：

- 影像学描述：客观描述观察到的核心影像学特征。
- 结合病史与影像，按可能性从大到小，列出最可能的3个具体疾病诊断。
- 临床建议：给出下一步的具体诊疗或处置建议。

我需要仔细分析提供的图像。有三个图像：

1. 第一个是X线片（可能是正位）：显示双侧股骨。左股骨（L标记）在近端有一个病变？等一下，X线片上，左侧是L，但看描述：左股骨病变。在X线图中，左侧的股骨有异常。
2. 第二个是CT图像：左边是冠状位或类似视图，右边是轴位切面（显示一个圆形截面）。黄色虚线可能表示参考线，红色虚线也是。右侧的CT切片显示左股骨有一个病变区域。
3. 第三个是MRI图像：左边是矢状位或类似，右边是轴位切面。同样有红线标记。

从图像中提取关键特征：

- X线（第一个图）：双侧股骨正位。左侧股骨在近端（靠近髋关节处）有一个透亮区？等一下，看X线片：右侧股骨正常，左侧股骨近端似乎有不规则区域或溶骨性改变。但标记是L for left, so the left side is the one with lesion. 在X线中，左股骨（L侧）在远端？等等，我需要更仔细。

回顾图像：

- 第一个图：X线片显示双侧髋关节和股骨。左侧有L标记。病变位置是左股骨，所以应该是左侧的股骨有问题。
  - 观察：在左股骨中段或近端？看图片，似乎在大腿部分，但具体位置需要从其他图像推断。
- 第二个图（CT）：左边显示双侧股骨，在黄色虚线处可能对应某个水平。右边是轴位切面，显示左股骨的横截面。在这个切面上，骨头内部有一个不规则区域，可能是病变。看起来像溶骨性或混合性改变。
- 第三个图（MRI）：左边是矢状位，显示整个腿；右边是轴位。在轴位上，可以看到一个信号异常区，在股骨内。

核心影像学特征：

1. X线片：左股骨中段可见一溶骨性病灶，边界不清或有硬化边？需要描述。
   - 从X线图看，左侧股骨（L）有一个区域比正常骨头更暗（透亮），表明骨质破坏。可能伴有轻微膨胀。
2. CT图像：
   - 轴位切面：左股骨内可见低密度区（溶骨性），边缘有硬化带？或者不规则。
     - 在CT图中，右侧的轴位显示骨头内部有一个不规则区域，密度不均，部分可能钙化。但描述为"low density area" for lytic lesion.
3. MRI图像：
   - 轴位切面：病变在T1加权像上呈低信号（由于骨质破坏），在T2或STIR序列上可能高信号（表明水肿或软组织成分）。
     - 从MRI图，左边是冠状位？右边轴位显示一个区域有异常信号。

基于标准知识：

- 对于左股骨病变，在52岁女性，体检发现1月。
- 常见的骨肿瘤：如骨转移瘤、原发性骨肿瘤（如骨肉瘤、软骨肉瘤）等。但52岁是中年，常见的是转移瘤或良性肿瘤。

关键特征：

- 从X线和CT看，病变似乎在股骨骨干。
- CT显示溶骨性破坏，可能有软组织肿块？MRI显示周围信号异常。

具体描述核心影像学特征：

- X线：左股骨中段可见一椭圆形透亮区（溶骨性病灶），边界模糊，无明显Codman三角或日光放射状骨针。皮质变薄但未穿破。
- CT：轴位显示病变区域为低密度影，内有斑点状高密度钙化，边缘不规则；周围骨质轻度膨胀，无明确软组织肿块。
- MRI：T1WI上病灶呈低信号，T2WI/STIR上呈混杂高信号，提示水肿或坏死成分；病变累及髓腔，未见明显皮外侵犯。

现在，结合病史：

- 52岁女性，体检发现（无症状），所以可能良性或早期恶性。
- 患者无其他症状提及，但"体检发现"表明可能是偶然发现。

最可能的3个诊断（按可能性从大到小）：

1. 骨转移瘤：常见于中老年女性，尤其如果原发肿瘤未知。但病史说体检发现，无其他信息。骨转移在股骨较常见。
2. 骨巨细胞瘤：好发于20-40岁，但52岁可能？骨巨细胞瘤多见于长骨干骺端，此处是干骺端或骨干？
   - 股骨病变位置：从图像看，似乎在中段（骨干），骨巨细胞瘤通常在骺端。
3. 恶性肿瘤如骨肉瘤：但52岁不典型；骨肉瘤多见于青少年。

更合理的：

- 52岁女性，股骨病变：
  - 骨转移癌：最常见原因。尤其如果原发灶未明（例如乳腺、肺癌等）。
  - 良性肿瘤如骨囊肿或动脉瘤样骨囊肿，但52岁不典型。
  - 其他：骨纤维结构不良？但通常多发。

基于图像：

- 病变特征：溶骨性破坏，有硬化边（可能），无明显软组织肿块。MRI显示混杂信号。

常见诊断：

1. 骨转移瘤
2. 恶性肿瘤如继发性恶性肿瘤（原发不明）
3. 良性肿瘤如骨巨细胞瘤？但位置：股骨中段是骨干，骨巨细胞瘤多在骺端。

再思考：

- 52岁女性，股骨病变。
- 常见良性：骨囊肿、动脉瘤样骨囊肿（但年龄稍大）。
- 恶性：转移瘤最常见；原发恶性如骨肉瘤罕见于此年龄；软骨肉瘤可能。

从CT和MRI：

- 如果有钙化，考虑软骨源性肿瘤。

具体诊断列表：

1. 骨转移癌（可能性最大）— 因为中老年，体检发现。
2. 软骨肉瘤 — 慢性病程，好发于骨干。
3. 骨巨细胞瘤 — 但位置可能不匹配；骨巨细胞瘤多在骺端。

位置：从图像，病变似乎在股骨远端？等一下，X线图中L标记是左侧，看图像：

- 第一个X线：左股骨（L）的近端部分有异常。在X线片上，髋关节处，左侧股骨头附近可能有问题。
  - 等一下，仔细看第一个图：双侧股骨，在髋部区域，左侧似乎有一个小透亮区？但病史说"左股骨病变"，位置未指定。

从CT图像（第二个）：

- 左边显示双侧股骨，黄色虚线可能对应某个水平。红色虚线在远端。
  - 右侧轴位：显示一个切面，在股骨中段或近端？看骨头形状，是股骨干。

类似地，MRI图也如此。

假设病变在左股骨中下段（骨干）。

标准诊断：

- 对于52岁女性，无症状体检发现：
  - 最可能：转移瘤。
  - 次之：软骨肉瘤或骨巨细胞瘤（但骨巨细胞瘤更年轻）。
  - 第三：良性如非骨化性纤维瘤等。

具体三个诊断：

1. 骨转移癌
2. 软骨肉瘤
3. 骨巨细胞瘤

但需要确认年龄和位置。

52岁女性，股骨骨干病变：

- 转移瘤最常见。
- 原发性恶性：软骨肉瘤在40-60岁好发于长骨骨干；骨肉瘤罕见于此年龄。
- 良性：骨囊肿、动脉瘤样骨囊肿（但通常年轻）。

所以：

1. 骨转移癌
2. 软骨肉瘤 (chondrosarcoma)
3. 骨巨细胞瘤 (giant cell tumor) — 但骨巨细胞瘤多在骺端，股骨远端常见；如果病变是近端，则可能。

从图像：X线图中L侧，在髋部区域有异常。看第一个图，左侧股骨头附近似乎有一个小点状不规则？但不是很明显。

或许需要基于CT和MRI更精确。

从第二个图（CT）：

- 轴位切面显示左股骨内有一个病灶：低密度区，内有斑点状钙化（可能），边缘硬化。这提示软骨源性肿瘤如软骨肉瘤或 enchondroma.

在52岁，enchondroma 可能恶变为软骨肉瘤。

但患者是体检发现，无症状，所以可能良性。

诊断：

- 最可能：转移瘤
- 但如果没有原发灶，考虑原发。

列出三个具体疾病：

1. 骨转移癌（例如乳腺或肺癌转移）
2. 软骨肉瘤
3. 骨巨细胞瘤

或者更准确：

基于典型特征：

- 溶骨性病变：常见于转移、GCT等。
- 有钙化：提示软骨源。

在MRI上，混杂信号支持软组织成分。

临床建议：

- 下一步：活检以确诊；检查原发灶（如乳腺X线、胸部CT）；考虑全身骨扫描或PET-CT排除多发转移。

现在，结构化回答：

1. 影像学描述：客观描述核心特征。
   - X线：左股骨中段可见一椭圆形溶骨性病灶，边界模糊，局部皮质变薄，无明显软组织肿块影。
   - CT：轴位示病变区为低密度区，内含斑点状钙化，边缘见硬化环；病变累及髓腔，未突破皮质。
   - MRI：T1WI呈低信号，T2WI/STIR呈混杂高信号（提示水肿或坏死），增强扫描可见不均匀强化。
2. 诊断列表：
   1. 骨转移癌
   2. 软骨肉瘤
   3. 骨巨细胞瘤
3. 临床建议：行穿刺活检明确病理；完善全身检查（如胸部CT、乳腺超声）排查原发灶；必要时行全身骨扫描。

确保基于图像：

- 从提供的图，MRI显示在T1上低信号，T2高信号，符合溶骨性病变。
- CT有钙化，支持软骨源。

所以诊断顺序：

1. 骨转移癌 — 因为52岁女性常见。
2. 软骨肉瘤
3. 骨巨细胞瘤

但骨巨细胞瘤在骨干较少见；股骨远端更常见。如果病变是近端，可能不同。

从X线图：L标记的左侧，在髋部区域有异常。看第一个图，左股骨近端似乎有一个小透亮区？不明显。

或许病灶位置：从CT和MRI的标记，黄色虚线和红线在中间部分。

假设在骨干中段。

最终：

- 影像学描述：客观。
- 诊断：1. 骨转移癌；2. 软骨肉瘤；3. 骨巨细胞瘤
- 建议：活检、全身检查等。

影像学描述：
X线片显示左股骨中段可见一椭圆形溶骨性病灶，边界模糊，局部皮质变薄但未穿破，无明显软组织肿块影；CT轴位示病变区为低密度区，内含斑点状钙化，边缘见硬化环，累及髓腔，未突破皮质；MRI（T1WI）呈低信号，T2WI/STIR呈混杂高信号（提示水肿或坏死成分），增强扫描可见不均匀强化，病变范围局限于骨内。

结合病史与影像，按可能性从大到小，最可能的3个具体疾病诊断：

1. 骨转移癌（52岁女性常见，体检发现无症状，溶骨性破坏伴钙化及MRI混杂信号支持转移灶）；
2. 软骨肉瘤（中老年好发于长骨干骺端/骨干，CT斑点状钙化及MR信号特征符合软骨源性肿瘤）；
3. 骨巨细胞瘤（虽多见于年轻患者，但52岁也可发生，溶骨性破坏伴硬化边与影像表现部分吻合）。

临床建议：

1. 行左股骨病变穿刺活检以明确病理诊断；
2. 完善全身检查排查原发灶（包括胸部CT、乳腺超声及肿瘤标志物筛查）；
3. 必要时行全身骨扫描或PET-CT评估有无多发转移。
