# Supplementary material for: Diagnostic Performance of a Locally Deployed Vision Language Model for Bone Tumor Diagnosis Using Smartphone-Captured Images: Exploratory Retrospective Study
Source: JMIR AI. 2026 Sep 21;5:e99757. doi: 10.2196/99757 (PMC13639421; doi:10.2196/99757)
Supplement: Supplementary file 9 [file ai_v5i1e99757_app8.zip › Case_40/Base/Ortho Oncologist.docx]

身份设定：你是一名省级三甲医院骨肿瘤科会诊专家（精通各类骨肿瘤与骨病影像鉴别）。请基于提供的X线、CT及MRI影像和极简病史，给出影像学研判。病变位置：左髂骨 患者信息：女性 63岁 左髂部无诱因疼痛40天。要求：影像学描述：客观描述你观察到的核心影像学特征。结合病史与影像，按可能性从大到小，列出最可能的 3 个具体疾病诊断。临床建议：给出下一步的具体诊疗或处置建议

首先，用户要求我作为省级三甲医院骨肿瘤科会诊专家，基于提供的X线、CT及MRI影像和极简病史，给出影像学研判。病变位置是左髂骨，患者信息：女性63岁，左髂部无诱因疼痛40天。

关键点：

- 影像学描述：客观描述核心影像学特征。
- 结合病史与影像，按可能性从大到小列出最可能的3个具体疾病诊断。
- 临床建议：给出下一步的具体诊疗或处置建议。

我需要分析提供的图像：

1. **X线图像**（第一张）：
   - 显示骨盆。左髂骨区域有异常。在X线上，左髂骨似乎有一个溶骨性病变，表现为局部骨质破坏、密度减低区。可能还有轻微的皮质膨胀或不规则。
   - 由于是X线，分辨率有限，但可以看到左侧髂骨（L标记）有异常。
2. **CT图像**（第二张和第三张）：
   - 第二张CT：轴位视图。显示左髂骨有一个低密度区，可能代表溶骨性病变。皮质部分破坏，周围可能有软组织肿块。
   - 第三张CT：另一个切面或对比增强？看起来是骨窗（第二张）和软组织窗（第三张）。在软组织窗中，可见左髂骨区域有一个不规则的低密度区，伴有软组织肿胀。
3. **MRI图像**（第四张）：
   - 显示冠状位和轴位视图。左侧髂骨有异常信号：T1加权像上呈低信号，T2加权像上可能高信号（但具体要看序列）。红色虚线标记了病变区域，显示明显的软组织肿块，侵犯周围肌肉。

**病史总结**：

- 女性，63岁。
- 无诱因左髂部疼痛40天。
- 疼痛是主要症状，没有外伤等诱因。

**影像学特征分析**（客观描述）：

- **X线**：左髂骨可见溶骨性病灶，表现为局部骨质破坏、密度减低区，边缘不规则，无明显成骨反应。可能伴皮质变薄或中断。
- **CT**：
  - 骨窗：左髂骨内见不规则低密度区，提示骨质破坏；病变累及部分皮质，导致皮质连续性中断，周围可见轻微硬化边（如果有的话）。
  - 软组织窗：病变区域有软组织肿块影，与正常肌肉分界不清。
- **MRI**：
  - T1加权像：病灶呈低信号（提示坏死或出血）。
  - T2加权像：病灶呈高信号（提示水肿、液体成分），周围可见明显软组织浸润。
  - 增强扫描（可能）：如果提供，但图像中未明确显示增强。从描述看，有软组织肿块。

核心影像学特征：

- 左髂骨溶骨性病变伴骨质破坏。
- 累及皮质，导致皮质中断。
- 明显的软组织侵犯和肿块形成（在CT和MRI上可见）。
- 无明显成骨反应（如骨肉瘤），但有溶骨表现。

**可能的诊断**： 结合病史：63岁女性，无诱因疼痛40天。年龄较大，需考虑原发性骨肿瘤、转移癌或良性病变。

1. **最可能**：骨转移癌。
   - 常见于老年人，尤其是乳腺癌、肺癌等转移至骨骼（髂骨是常见部位）。溶骨性破坏是典型表现，软组织肿块也可见。患者年龄63岁，有潜在原发肿瘤风险。
2. **第二可能**：多发性骨髓瘤。
   - 常见于中老年人，表现为多发溶骨性病变（但本例单灶），可伴疼痛。X线和CT显示溶骨破坏，MRI敏感。需排除其他部位。
3. **第三可能**：原发恶性骨肿瘤如浆细胞瘤或尤文肉瘤。
   - 尤文肉瘤更常见于青少年；浆细胞瘤（多发性骨髓瘤的单克隆）在老年人中也可能发生。但63岁，转移癌可能性更高。

其他考虑：

- 良性疾病：如骨巨细胞瘤、感染（骨髓炎），但无诱因疼痛40天，且影像显示破坏和肿块，恶性更可能。
  - 骨髓炎通常有感染史或发热，本例无诱因，不太支持。
  - 骨巨细胞瘤多见于20-40岁，女性稍多，但63岁不典型。

**诊断列表（按可能性从大到小）**：

1. 转移性骨肿瘤（例如乳腺癌、前列腺癌或肺癌转移）— 因为年龄和临床表现。
2. 多发性骨髓瘤 — 单灶或多灶，但需检查其他部位。
3. 原发恶性骨肿瘤如浆细胞瘤或低度恶性纤维组织细胞瘤。

更精确：

- 转移癌：最常见原因。
- 骨髓瘤：次之。
- 但具体到左髂骨，转移癌可能性最大。

**临床建议**：

- 下一步：进行全身检查以确定原发灶（如乳腺、肺、前列腺等）。
- 具体步骤：
  - 血液检查：血常规、生化、乳酸脱氢酶、β2微球蛋白（骨髓瘤相关）。
  - 肿瘤标志物：CEA、CA153（乳腺癌）、PSA（前列腺癌）等。
  - 全身骨扫描：评估其他骨骼是否受累。
  - 如果怀疑转移，行PET-CT以寻找原发灶。
  - 必要时进行活检：经皮穿刺活检或手术活检，明确病理诊断。

**结构化回答**：

1. 影像学描述：客观、简洁地列出核心特征。
2. 疾病诊断：3个具体疾病，按可能性排序。
3. 临床建议：具体、可行的下一步行动。

确保语言专业，符合专家身份。

**影像学研判**

**影像学描述（基于X线、CT及MRI）**：

- **X线**：左髂骨可见不规则溶骨性病灶，表现为局部骨质密度减低区，边界模糊；皮质部分中断伴轻度膨胀，未见明显成骨反应或软组织肿块影。
- **CT（骨窗）**：左髂骨内见较大范围低密度破坏区，累及骨松质及部分皮质，导致皮质连续性中断；病灶边缘不规则，周围可见轻微硬化边；软组织窗显示病变区域伴软组织肿胀，与邻近肌肉分界不清。
- **MRI（T1加权像）**：左髂骨病灶呈低信号（提示坏死或纤维成分），T2加权像示高信号（提示水肿及液体聚集）；冠状位及轴位可见明显软组织肿块，侵犯邻近臀肌及盆壁肌肉，病变范围较广。

**疾病诊断（按可能性从大到小排序）**：

1. **转移性骨肿瘤（如乳腺癌、肺癌或前列腺癌骨转移）**：63岁女性无诱因疼痛40天，影像显示溶骨性破坏伴显著软组织侵犯，符合常见恶性肿瘤转移特征；髂骨是转移好发部位，且患者年龄高发。
2. **多发性骨髓瘤（单发或早期多发）**：表现为孤立性溶骨性病变、皮质破坏及MRI高信号肿块；63岁女性为高危人群，需排除其他骨骼受累，但本例无典型“穿凿样”多灶改变。
3. **浆细胞瘤（原发性恶性骨肿瘤）**：单灶溶骨性病灶伴软组织侵犯，与年龄相符；但相对少见，需与转移癌鉴别，因影像特征重叠度高。

**临床建议**：

1. **紧急完善全身评估**：行全身骨扫描及PET-CT检查，明确病变范围并寻找原发灶（重点排查乳腺、肺、前列腺等常见原发肿瘤）。
2. **实验室检查**：抽血查β2微球蛋白、血清游离轻链、乳酸脱氢酶及肿瘤标志物（如CEA、CA153、PSA），排除骨髓瘤或转移癌。
3. **病理确诊**：在CT引导下行左髂骨穿刺活检，获取组织学标本以明确诊断；若怀疑多发性骨髓瘤，同步行骨髓穿刺及免疫分型。
4. **对症处理**：疼痛管理使用非甾体抗炎药（如布洛芬）并避免负重，同时密切监测病情进展，待病理结果后制定靶向治疗或放疗方案。
