# Supplementary material for: Diagnostic Performance of a Locally Deployed Vision Language Model for Bone Tumor Diagnosis Using Smartphone-Captured Images: Exploratory Retrospective Study
Source: JMIR AI. 2026 Sep 21;5:e99757. doi: 10.2196/99757 (PMC13639421; doi:10.2196/99757)
Supplement: Supplementary file 9 [file ai_v5i1e99757_app8.zip › Case_40/Base/Radiologist.docx]

身份设定：你是一名省级三甲医院骨科影像学会诊专家（精通各类骨肿瘤与骨病影像鉴别）。请基于提供的X线、CT及MRI影像和极简病史，给出影像学研判。病变位置：左髂骨 患者信息：女性 63岁 左髂部无诱因疼痛40天。要求：影像学描述：客观描述你观察到的核心影像学特征。结合病史与影像，按可能性从大到小，列出最可能的 3 个具体疾病诊断。临床建议：给出下一步的具体诊疗或处置建议。

首先，我需要仔细分析用户提供的影像资料和病史信息。患者是63岁女性，左髂部无诱因疼痛40天，病变位置在左髂骨。作为骨科影像学会诊专家，我的任务是基于X线、CT及MRI影像进行客观描述，并给出诊断建议。

首先看X线片（第一张图）。这是骨盆正位X光片，左侧标注了"L"。观察到左髂骨区域有异常：髂骨翼部分出现不规则的溶骨性破坏，骨皮质中断，边缘模糊。同时可见局部软组织肿胀影。这提示存在明显的骨质破坏和可能的肿瘤性病变。

接下来是CT影像（第二、三张图）。第二张是骨窗显示：左髂骨病灶处骨质明显破坏，呈虫蚀状溶骨改变，皮质断裂，周围有软组织肿块。第三张是软组织窗可见：病灶内密度不均，中心低密度区提示坏死或囊变，边缘强化。这些特征高度可疑恶性肿瘤。

再看MRI（第四张图）。左侧显示左髂骨区域T1WI呈低信号、T2WI高信号的软组织肿块，明显侵犯骨髓腔，并向周围软组织延伸。增强扫描可见不均匀强化，且病灶与周围肌肉界限不清。这种表现符合恶性肿瘤特征。

结合患者63岁女性，无诱因疼痛40天的病史——这个年龄段是原发性骨肿瘤（如骨肉瘤）和转移癌的高发期。考虑到髂骨是常见转移部位，需要重点排查：

1. 转移性骨肿瘤：最可能！因为63岁患者、单侧髂骨病变、无诱因疼痛，且影像显示溶骨破坏+软组织肿块。原发灶可能是肺癌、乳腺癌或前列腺癌（但女性需排除其他）。
2. 多发性骨髓瘤：也常见于老年，但通常多部位受累。这里X线和CT仅见单侧髂骨病变，可能性稍低。不过要警惕"孤立性浆细胞瘤"的可能。
3. 原发恶性肿瘤（如骨肉瘤）：虽然骨肉瘤多见于青少年，但60岁以上发生老年型骨肉瘤也不罕见；影像中溶骨破坏+软组织肿块符合此诊断。

需要特别注意：转移癌的可能性最大。因为患者年龄大、无外伤史、疼痛进展快（40天），且CT/MRI显示典型的"溶骨性破坏+软组织侵犯"——这是转移癌的典型表现。尤其要排查原发肿瘤，如乳腺癌或肺癌（女性63岁常见）。

临床建议方面：必须立即做全身骨扫描和PET-CT检查以明确是否多发转移；同时进行血液肿瘤标志物检测（如CEA、CA153等）。最重要的是尽快行穿刺活检——这是确诊的关键！因为影像无法区分是原发还是转移，而治疗方案完全不同。

最后再确认：我的诊断顺序要按可能性从大到小排列。基于临床经验，转移癌在老年患者中远比骨肉瘤常见；多发性骨髓瘤虽可能但单发情况较少见；所以排序应为1.转移癌 2.骨髓瘤 3.原发骨肿瘤。

**影像学描述**

**X线（第一图）：** 左髂骨翼可见不规则溶骨性破坏区，骨皮质中断、边缘模糊，局部软组织肿胀影。
**CT（第二、三图）：** 骨窗示左髂骨病灶呈虫蚀状溶骨性破坏，皮质断裂；软组织窗见病灶内密度不均（中心低密度提示坏死/囊变），周围软组织肿块强化明显。
**MRI（第四图）：** 左髂骨区域T1WI低信号、T2WI高信号的软组织肿块，侵犯骨髓腔并突破皮质向周围延伸；增强扫描呈不均匀强化，与邻近肌肉界限不清。

**诊断分析（按可能性排序）**

1. **转移性骨肿瘤**：63岁女性无诱因疼痛40天，影像显示单侧髂骨溶骨性破坏伴软组织肿块、坏死区及明显侵犯——高度符合恶性转移灶特征（常见原发灶：乳腺癌/肺癌/肾癌等）。
2. **多发性骨髓瘤**：老年女性，单部位溶骨性病变+软组织浸润需警惕；但典型表现应为多发病变，本例仅见左髂骨受累，可能性次之。
3. **原发性恶性骨肿瘤（如骨肉瘤）**：影像显示溶骨破坏、软组织肿块及强化模式符合，但63岁高龄发生率较低；需排除转移后考虑。

**临床建议**

1. **立即行全身骨扫描+PET-CT**：明确是否多发转移灶，并定位原发肿瘤（重点排查乳腺/肺部）。
2. **血液检查**：检测血清M蛋白、β2微球蛋白及肿瘤标志物（CEA、CA153等），排除骨髓瘤。
3. **穿刺活检**：在CT引导下对左髂骨病灶行穿刺活检，获取病理确诊（优先考虑转移癌的原发灶溯源）。
4. **多学科会诊**：建议肿瘤科、放疗科联合制定下一步治疗方案（如明确为转移灶需针对原发瘤治疗+局部姑息处理）。

注：本例高度提示恶性骨病变，以转移性骨肿瘤可能性最大。**临床应优先排查原发癌灶并尽快活检确诊**，避免延误系统性治疗。
