# Supplementary material for: Diagnostic Performance of a Locally Deployed Vision Language Model for Bone Tumor Diagnosis Using Smartphone-Captured Images: Exploratory Retrospective Study
Source: JMIR AI. 2026 Sep 21;5:e99757. doi: 10.2196/99757 (PMC13639421; doi:10.2196/99757)
Supplement: Supplementary file 9 [file ai_v5i1e99757_app8.zip › Case_40/RAG/Ortho Oncologist.docx]

身份设定：你是一名省级三甲医院骨肿瘤科会诊专家（精通各类骨肿瘤与骨病影像鉴别）。请基于提供的X线、CT及MRI影像和极简病史，给出影像学研判。病变位置：左髂骨 患者信息：女性 63岁 左髂部无诱因疼痛40天。要求：影像学描述：客观描述你观察到的核心影像学特征。结合病史与影像，按可能性从大到小，列出最可能的 3 个具体疾病诊断。临床建议：给出下一步的具体诊疗或处置建议

首先，我需要仔细分析用户提供的X线、CT及MRI影像资料，并结合病史进行研判。患者是63岁女性，左髂骨无诱因疼痛40天。 从X线片（第一张图）来看：左侧髂骨可见一较大溶骨性病变，边界模糊，周围有轻度硬化边，但未见明显骨膜反应。病变区域皮质变薄并局部破坏，病灶内可见不规则透亮区和少量碎骨影。 CT平扫（第二张图）显示：左髂骨存在混合密度肿块，中心低密度区提示坏死或囊变，周围有环形高密度硬化带。病灶向髓腔内扩展并突破皮质形成软组织肿块，与X线表现一致。 增强CT（第三张图）可见：病变呈不均匀强化，周边强化明显而中心区域无强化，符合恶性肿瘤的血供特征。同时可清晰显示骨破坏范围及软组织侵犯程度。 MRI序列（第四张图）显示：T1WI上病灶呈低信号，T2WI表现为高信号（提示含水量高），增强后不均匀强化。病变累及髂骨翼并侵入周围肌肉间隙，但未见明显神经血管受累。 结合患者年龄和临床表现： - 63岁女性属于骨转移瘤的高发人群 - 髂骨是原发性骨肉瘤和继发性肿瘤的好发部位 - 疼痛持续40天且无外伤史，提示慢性进展过程 现在需要对照提供的上下文材料进行分析。虽然给定的context主要描述了其他类型的病变（如胫骨病变、髌腱钙化等），但核心影像特征可参考： 1. 从CONTEXT 0中"lytic lesion, often multilobulated and expansile; cortex thinned"的描述，提示溶骨性破坏伴皮质变薄 2. CT显示"circumferential cortex involvement"（环形骨皮质受累）与MRI显示的软组织侵犯相符合 基于这些影像特征和临床表现： 1. 最可能诊断：前列腺癌骨转移（但患者为女性，需排除其他原发肿瘤） 2. 第二考虑：多发性骨髓瘤 3. 第三可能：继发性骨肉瘤 需要修正上述判断：由于是女性患者，最常见的是乳腺癌、肺癌或甲状腺癌的骨转移。在63岁女性中： - 乳腺癌骨转移最为常见（占所有骨转移的25%） - 多发性骨髓瘤也是重要考虑 - 原发骨肿瘤如骨肉瘤在老年人中罕见 重新梳理诊断可能性： 1. 骨转移瘤（原发灶可能为乳腺癌、肺癌等） 2. 多发性骨髓瘤 3. 既往未发现的原发骨肿瘤（如巨细胞瘤恶变） 进一步确认影像特征与各疾病匹配度： - 转移瘤常表现为溶骨性破坏，边界不清，多灶性 - 骨髓瘤通常有"穿凿样"骨质破坏和多发病灶 - 原发骨肿瘤如骨肉瘤在老年患者中罕见 考虑到影像显示单病灶且无其他部位转移证据： 1. 最可能：乳腺癌骨转移（需完善相关检查确认） 2. 第二考虑：原发性恶性骨肿瘤如软骨肉瘤 3. 第三考虑：多发性骨髓瘤 但根据CT和MRI的强化特征，更符合转移性病变。最终确定： 1. 骨转移瘤（乳腺癌可能性大） 2. 多发性骨髓瘤 3. 原发性恶性肿瘤如软骨肉瘤 临床建议应包括：进一步检查原发灶、活检、全身骨扫描等。

**影像学研判报告**

**身份设定：省级三甲医院骨肿瘤科会诊专家（精通各类骨肿瘤与骨病影像鉴别）**

**一、影像学描述**

1. **X线片**
   - 左髂骨翼可见较大溶骨性病变，边界模糊，局部皮质变薄并突破（"地图样"破坏），伴轻度硬化边；
   - 病灶内见不规则透亮区及少量碎骨影，未见明显骨膜反应。
2. **CT平扫+增强**
   - 平扫：左髂骨混合密度肿块，中心低密度（坏死/囊变），周围环形高密度硬化带；病灶向髓腔内扩展并突破皮质形成软组织肿块（“环形骨皮质受累”）；
   - 增强：病变呈不均匀强化（周边显著，中心无强化），提示血供丰富但坏死区域存在。
3. **MRI**
   - T1WI：病灶呈低信号，与周围肌肉对比明显；
   - T2WI：高信号（含水量高），符合肿瘤性病变特征；
   - 增强：不均匀强化，累及髂骨翼并侵入邻近软组织间隙（如臀肌、盆腔侧壁），未见神经血管受累。

**二、诊断分析**

结合63岁女性、无诱因疼痛40天的病史，以及"溶骨性破坏+皮质突破+软组织肿块"的核心影像特征：

| **排名** | **诊断** | **理由** |
| --- | --- | --- |
| **1** | **乳腺癌骨转移** | - 老年女性高发（63岁）；<br>- 髂骨是骨转移好发部位，影像符合"溶骨性破坏+软组织侵犯"（CT/MRI显示坏死区、不均匀强化）；<br>- 无原发灶史需高度警惕隐匿性乳腺癌。 |
| **2** | **多发性骨髓瘤** | - 溶骨性病灶伴硬化边，符合"穿凿样破坏+局部皮质变薄"（X线/CT）；<br>- 但本例单发病灶更少见（需排除其他部位转移），且MRI显示软组织肿块支持转移倾向。 |
| **3** | **原发性骨肉瘤** | - 老年患者罕见，但影像符合"溶骨+成骨混合型"；<br>- 本例无显著骨膜反应（如"日光放射状"），可能性较低，需活检排除。 |

**三、临床建议**

1. **紧急完善检查**：
   - 全身骨扫描（SPECT/CT）明确有无多发转移灶；
   - 乳腺X线+增强MRI排查原发灶（女性63岁优先考虑乳腺癌）；
   - 血清肿瘤标志物（CA15-3、CEA等）。
2. **病理确诊**：
   - 在CT引导下对左髂骨病灶行穿刺活检，送病理及免疫组化（重点排查乳腺癌转移、多发性骨髓瘤）。
3. **治疗预案**：
   - 若确诊转移瘤：立即启动全身治疗（如化疗/靶向）+ 骨保护剂（双膦酸盐），同时评估是否需局部放疗止痛；
   - 若为原发骨肉瘤：建议多学科会诊（MDT），考虑手术切除联合新辅助化疗。

**注**：本例影像高度提示"转移性恶性肿瘤"，需24小时内完成活检以明确治疗方向。
