# Supplementary material for: Diagnostic Performance of a Locally Deployed Vision Language Model for Bone Tumor Diagnosis Using Smartphone-Captured Images: Exploratory Retrospective Study
Source: JMIR AI. 2026 Sep 21;5:e99757. doi: 10.2196/99757 (PMC13639421; doi:10.2196/99757)
Supplement: Supplementary file 9 [file ai_v5i1e99757_app8.zip › Case_40/RAG/Radiologist.docx]

身份设定：你是一名省级三甲医院骨科影像学会诊专家（精通各类骨肿瘤与骨病影像鉴别）。请基于提供的X线、CT及MRI影像和极简病史，给出影像学研判。病变位置：左髂骨 患者信息：女性 63岁 左髂部无诱因疼痛40天。要求：影像学描述：客观描述你观察到的核心影像学特征。结合病史与影像，按可能性从大到小，列出最可能的 3 个具体疾病诊断。临床建议：给出下一步的具体诊疗或处置建议。

首先，用户要求我作为省级三甲医院骨科影像学会诊专家来分析提供的X线、CT和MRI影像以及极简病史。病变位置是左髂骨，患者是一位63岁女性，无诱因左髂部疼痛40天。 我的任务包括： 1. **影像学描述**：客观描述观察到的核心影像学特征。 2. **结合病史与影像，按可能性从大到小列出最可能的3个具体疾病诊断。** 3. **临床建议**：给出下一步的具体诊疗或处置建议。 现在，我需要仔细分析提供的影像： - **X线图像（第一张）**：显示骨盆正位片。左髂骨区域有异常。在X线上，左髂骨似乎有一个溶骨性病变，表现为局部骨质破坏、密度减低区。可能伴有骨皮质中断或膨胀。右侧看起来正常作为对比。 - **CT图像（第二张和第三张）**： - 第二张CT：轴位骨窗。显示左髂骨有明显的骨质破坏区域，边缘不规则，可能有软组织肿块。骨皮质部分缺失。 - 第三张CT：可能是软组织窗或增强扫描？图像中左髂骨区域有低密度区，代表骨质破坏，并且周围似乎有软组织影。 - **MRI图像（第四张）**： - 左侧是冠状位T1加权像？显示左髂骨有一个高信号病变，在T1上可能为低信号或等信号（取决于序列）。右侧是轴位图像，显示病变区域在T2上呈高信号，提示液体成分或水肿。有明显的软组织肿块延伸到周围。 关键影像学特征： - **X线**：左髂骨溶骨性破坏，皮质中断。 - **CT**：骨窗显示骨质溶解、不规则边缘；软组织窗/增强可能显示软组织浸润。 - **MRI**：T1加权像上病变信号不均匀（可能低或等），T2高信号，提示水肿和软组织肿块。 结合病史： - 患者63岁女性，无诱因疼痛40天。年龄较大，骨肿瘤可能性存在；疼痛时间较短，但无外伤。 - 骨肿瘤常见于中老年人：原发性骨肿瘤（如骨肉瘤、软骨肉瘤）较少见，转移癌更常见。 最可能的3个诊断： 1. **骨转移癌**：63岁女性，左髂骨疼痛。最常见的骨肿瘤是转移癌，尤其是来自乳腺癌、肺癌等。影像上溶骨性破坏符合。 2. **多发性骨髓瘤**：好发于中老年人，常表现为多发溶骨性病变，但这里可能单发；MRI显示软组织肿块支持。 3. **原发骨肿瘤如浆细胞瘤（属于骨髓瘤的一部分）或转移癌更优先。** 具体诊断： - 1. 转移性骨肿瘤（例如：乳腺癌、前列腺癌等转移）。因为63岁女性，常见原发灶是乳腺癌。 - 2. 多发性骨髓瘤：但需确认是否有其他部位病变；这里可能单发，但骨髓瘤可单发。 - 3. 原发骨肿瘤如软骨肉瘤或骨巨细胞瘤。但63岁女性，原发骨肿瘤较少见。 从影像： - 溶骨性破坏、软组织肿块：转移癌和骨髓瘤都可能。 - 骨髓瘤常呈多发、穿凿样；转移癌可单发或多发。 可能性排序： 1. **转移性骨肿瘤**（最常见于老年人，无诱因疼痛）。 2. **多发性骨髓瘤**（但需检查是否有其他部位病变）。 3. **原发骨肉瘤或软骨肉瘤**（但年龄偏大，原发骨肉瘤更年轻；63岁可能有浆细胞瘤）。 临床建议： - 下一步：进行全身骨扫描以评估是否多发性病变。 - 活检：穿刺活检明确病理诊断。 - 原发灶排查：如乳腺X线、肺CT等，因为转移癌常见原发于这些部位。 现在，结构化回答： 1. **影像学描述**： - X线：左髂骨可见溶骨性骨质破坏区，边缘模糊，局部皮质中断。 - CT（骨窗）：左髂骨病灶呈不规则低密度区，骨皮质部分缺失，周围见软组织肿块。 - CT（软组织窗/增强）：病变区域强化不均匀，提示软组织浸润。 - MRI：T1加权像示病变信号减低，T2加权像高信号，伴明显软组织肿块形成。 2. **最可能的3个诊断**： 1. 转移性骨肿瘤（如乳腺癌转移）— 最常见 2. 多发性骨髓瘤 — 次之 3. 原发性浆细胞瘤或溶骨性骨肉瘤 — 较少见 3. **临床建议**： - 立即进行全身骨扫描以评估病变范围。 - 安排CT引导下穿刺活检获取病理标本。 - 排查原发灶：乳腺X线、肺部CT等。 确保回答客观、专业，符合专家身份。只返回我的响应部分。

**影像学描述**

- **X线**：左髂骨可见不规则溶骨性骨质破坏区（密度减低），边界模糊，局部皮质中断，未见明显成骨反应；右侧髂骨及骶髂关节对称正常。
- **CT（骨窗）**：左髂骨病灶呈大片状低密度区，骨皮质部分缺失、不连续，边缘毛糙伴骨嵴形成；软组织窗显示病变周围有中等密度软组织肿块影。
- **MRI**：T1加权像示病灶信号明显减低（与正常骨髓对比），T2加权像呈高信号混杂区，提示水肿及液化成分；冠状位可见病变向髂翼外侧延伸形成软组织肿块，并累及邻近肌肉。

**结合病史与影像的诊断排序**

1. **转移性骨肿瘤（最可能）**：63岁女性无诱因疼痛40天，符合常见恶性肿瘤转移特征；X线/CT显示溶骨性破坏伴软组织肿块，MRI高信号提示活跃病变。原发灶多见于乳腺癌、肺癌或前列腺癌（本例女性需优先排查乳腺来源）。
2. **多发性骨髓瘤**：中老年患者典型表现，影像以溶骨性破坏为主；但本例单发病变可能性稍低，需结合血液学检查排除。
3. **原发性浆细胞瘤（孤立性骨髓瘤）**：63岁女性、溶骨性病灶伴软组织浸润符合特征；但较转移癌少见，需活检确诊。

**临床建议**

1. **立即完善全身骨扫描**：明确是否多发病变以区分转移 vs 单发肿瘤（如骨髓瘤）。
2. **CT引导下穿刺活检**：取病变区软组织及骨质标本行病理学检查，必要时送免疫组化。
3. **排查原发灶**：同步进行乳腺X线/钼靶、胸部增强CT及血清蛋白电泳（筛查肿瘤标志物如CA15-3）。
4. **疼痛管理**：短期予双膦酸盐类药物控制骨痛，避免负重活动。
