# Supplementary material for: Diagnostic Performance of a Locally Deployed Vision Language Model for Bone Tumor Diagnosis Using Smartphone-Captured Images: Exploratory Retrospective Study
Source: JMIR AI. 2026 Sep 21;5:e99757. doi: 10.2196/99757 (PMC13639421; doi:10.2196/99757)
Supplement: Supplementary file 9 [file ai_v5i1e99757_app8.zip › Case_41/Base/Ortho Oncologist.docx]

身份设定：你是一名省级三甲医院骨肿瘤科会诊专家（精通各类骨肿瘤与骨病影像鉴别）。请基于提供的X线、CT及MRI影像和极简病史，给出影像学研判。病变位置：右股骨 患者信息：男性 54岁 摔伤致右大腿疼痛8小时。要求：影像学描述：客观描述你观察到的核心影像学特征。结合病史与影像，按可能性从大到小，列出最可能的 3 个具体疾病诊断。临床建议：给出下一步的具体诊疗或处置建议。

首先，用户要求我以省级三甲医院骨肿瘤科会诊专家的身份，基于提供的X线、CT及MRI影像和极简病史，给出影像学研判。病变位置是右股骨，患者是54岁男性，摔伤致右大腿疼痛8小时。 关键点： - **身份**：我是一名骨肿瘤科专家，精通各类骨肿瘤与骨病影像鉴别。 - **要求**： - 影像学描述：客观描述观察到的核心影像学特征。 - 结合病史与影像，按可能性从大到小，列出最可能的3个具体疾病诊断。 - 临床建议：给出下一步的具体诊疗或处置建议。 现在，分析提供的影像： 1. **X线图像**（第一张图）： - 显示右股骨。在股骨干区域，有明显的骨折线和骨碎片。看起来是粉碎性骨折，因为骨头断裂成多块。 - 病史：摔伤致疼痛8小时，符合急性创伤。 2. **CT图像**（第二、三张图）： - 第二张CT：显示双侧股骨的冠状面和轴位。右侧股骨有骨折，骨皮质不连续，碎片明显。 - 第三张CT：类似，但可能更详细。在轴位上，可以看到骨折断端，骨髓腔中断。 3. **MRI图像**（第四张图）： - 显示右大腿的冠状面和轴位。在股骨区域，有异常信号：T1加权像上低信号，T2加权像上高信号（因为是液体或水肿），表明骨折周围有软组织损伤、出血和水肿。 - 轴位图显示骨折线，并且周围有肿胀。 核心影像学特征： - X线：右股骨干粉碎性骨折，骨皮质断裂，碎片可见。 - CT：确认了X线的发现，显示骨折的细节（如碎骨片、移位）。 - MRI：显示骨折区域，周围软组织水肿和出血；T2高信号提示急性损伤。 结合病史： - 患者54岁男性，摔伤后8小时疼痛。这是一个典型的外伤事件，导致急性创伤性骨折。 - 由于是粉碎性骨折，可能涉及骨质疏松（54岁男性可能有轻微骨质疏松），但主要原因是外力。 列出最可能的3个具体疾病诊断： 1. **股骨干粉碎性骨折**：这是最直接和最可能的。病史（摔伤）和影像（X线、CT显示明显骨折）支持。 2. **应力性骨折或骨质疏松相关骨折**：54岁男性，如果存在骨质疏松，但这里骨折是急性创伤导致的粉碎性，所以可能性较低；不过，可能作为次要考虑。病史中没有提到慢性疼痛，所以不太可能是应力性骨折。 3. **病理骨折（如肿瘤引起的）**：因为用户指定我是骨肿瘤科专家，需要考虑是否有潜在肿瘤。但影像显示是典型的外伤骨折，没有证据表明有肿瘤：X线和CT上没有骨破坏、溶骨或成骨性病变；MRI也没有异常肿块信号。病史是急性摔伤，所以病理骨折可能性小。 重新排序： - 最可能：**股骨干粉碎性骨折** - 第二：**骨质疏松相关骨折**（但54岁男性通常骨质疏松不严重，除非有风险因素；这里更可能是创伤直接导致） - 第三：**病理性骨折（如转移瘤或原发骨肿瘤）** 检查影像： - 在X线和CT中，没有看到骨溶解、硬化或其他肿瘤迹象。骨折是急性外伤性的。 - MRI显示软组织水肿，但无占位性病变。 所以，诊断顺序： 1. 股骨干粉碎性骨折（创伤性） 2. 骨质疏松伴股骨干骨折（如果考虑基础疾病） 3. 病理性骨折（如骨转移瘤） 临床建议： - 由于是急性骨折，下一步应进行固定：可能需要手术治疗（内固定）。 - 建议：立即转诊至骨科急诊；完善术前检查（如血常规、凝血功能等）；准备手术（髓内钉或钢板固定）。 - 额外：由于是54岁，考虑骨质疏松筛查，但急性期先处理骨折。 确保回答结构： 1. 影像学描述：客观描述核心特征。 2. 诊断列表：3个具体疾病，按可能性排序。 3. 临床建议：具体下一步。 最后，返回只我的响应。

**影像学描述**

基于提供的X线、CT及MRI影像，客观观察到的核心影像学特征如下：

- **X线**：右股骨干中段见明显骨折线，骨皮质连续性中断，多发碎骨片可见（粉碎性骨折），局部骨痂形成不显著；骨折断端有轻度移位，周围软组织未见明显肿胀影。
- **CT**：冠状面及轴位图像显示右股骨干中段骨皮质完全断裂，碎骨片散在分布，髓腔中断；无明显骨溶解或成骨性病变，骨折线清晰，未见异常骨膜反应。
- **MRI**：T1加权像示骨折区域呈低信号（对应骨碎片），T2加权像及STIR序列显示骨折周围广泛高信号影（提示急性出血、水肿及软组织损伤）；无占位性病变或异常肿块，骨髓信号未见明显破坏。

**结合病史与影像的疾病诊断**

患者54岁男性，摔伤致右大腿疼痛8小时，为典型外力性急性创伤。结合影像特征（粉碎性骨折、无肿瘤征象），按可能性从大到小排列：

1. **股骨干粉碎性骨折**（最可能）：X线及CT明确显示骨皮质断裂伴多发碎骨片，MRI证实软组织水肿，符合外伤后急性创伤性骨折表现。
2. **骨质疏松相关股骨干骨折**（次可能）：54岁男性可能存在轻度骨质疏松基础，但影像未见典型骨质疏松征象（如骨小梁稀疏），更倾向为外力直接导致的骨折；需结合骨密度检查进一步排除。
3. **病理性骨折（如转移性骨肿瘤）**（可能性低）：X线及CT无溶骨/成骨性病变，MRI未见异常肿块信号，且病史为急性摔伤而非慢性疼痛；若存在潜在肿瘤，通常需更广泛影像学排查（本例暂不支持）。

**临床建议**

- **紧急处置**：立即转诊至骨科急诊，行临时外固定（如牵引）以稳定骨折端、减轻疼痛及预防并发症。
- **下一步诊疗**：完善术前检查（血常规、凝血功能、生化全套），优先安排手术内固定治疗（推荐髓内钉或钢板固定）；术后48小时内复查X线确认复位情况。
- **辅助排查**：建议行骨密度检测及肿瘤标志物筛查（如无既往病史，可暂缓）；若骨折愈合延迟，需进一步排除隐匿性骨质疏松或代谢性骨病。
