# Supplementary material for: Diagnostic Performance of a Locally Deployed Vision Language Model for Bone Tumor Diagnosis Using Smartphone-Captured Images: Exploratory Retrospective Study
Source: JMIR AI. 2026 Sep 21;5:e99757. doi: 10.2196/99757 (PMC13639421; doi:10.2196/99757)
Supplement: Supplementary file 9 [file ai_v5i1e99757_app8.zip › Case_41/Base/Radiologist.docx]

身份设定：你是一名省级三甲医院骨科影像学会诊专家（精通各类骨肿瘤与骨病影像鉴别）。请基于提供的X线、CT及MRI影像和极简病史，给出影像学研判。病变位置：右股骨 患者信息：男性 54岁 摔伤致右大腿疼痛8小时。要求：影像学描述：客观描述你观察到的核心影像学特征。结合病史与影像，按可能性从大到小，列出最可能的 3 个具体疾病诊断。临床建议：给出下一步的具体诊疗或处置建议。

首先，用户要求我作为省级三甲医院骨科影像学会诊专家，基于提供的X线、CT及MRI影像和极简病史，给出影像学研判。病变位置是右股骨，患者信息：男性54岁，摔伤致右大腿疼痛8小时。

我的响应需要包括三个部分：

1. 影像学描述：客观描述观察到的核心影像学特征。
2. 结合病史与影像，按可能性从大到小，列出最可能的3个具体疾病诊断。
3. 临床建议：给出下一步的具体诊疗或处置建议。

现在，分析提供的影像：

- 第一张图是X线片（似乎是右股骨）：显示“R”标记在左侧，表明右侧。图像中可以看到股骨有骨折迹象；骨头不连续，有错位和碎裂的区域。
- 第二张图是CT扫描：左边是X线视图，右边是轴向CT切面。CT显示股骨皮质中断、碎片化，周围软组织可能有肿胀。
- 第三张图也是CT扫描：类似第二张，但可能是不同层面或更详细的CT图像，同样显示骨折和骨碎片。
- 第四张图是MRI：左边是矢状位，右边是轴向T2加权像。MRI显示股骨内信号异常（可能表示水肿、出血），周围软组织有高信号（表明水肿或血肿）。

关键影像学特征：

- X线：右股骨干骨折，可见明显断裂和错位。
- CT：确认骨折位置和程度；皮质骨中断，多碎片，骨髓腔内可见异常密度。
- MRI：T2加权像显示骨髓水肿（高信号），周围软组织肿胀，可能有血肿。

病史：54岁男性，摔伤后8小时右大腿疼痛。这提示急性创伤性事件。

现在，影像学描述： 我需要客观描述核心特征。基于所有图像：

- X线：右股骨干中段可见骨折线，骨皮质断裂，断端有轻度错位。
- CT：轴向CT显示多发骨折碎片，骨皮质不连续，骨髓腔内高密度影（可能为出血）。
- MRI：T2加权像示骨折区域骨髓信号增高（水肿/出血），周围软组织肿胀伴高信号。

结合病史与影像：

- 患者是摔伤后急性疼痛，8小时时间点符合急性创伤。
- 影像显示典型的股骨干骨折特征。没有迹象表明是肿瘤或慢性疾病；这是急性外伤。
- 可能的诊断：最可能的是股骨干骨折（急性）。

列出3个具体疾病诊断按可能性从大到小：

1. 最可能：**右股骨干骨折（急性创伤性）** – 因为病史和影像都明确显示骨折。
2. 第二可能：**病理性骨折** – 但病史是摔伤，且无肿瘤迹象；不过54岁男性，需考虑骨质疏松或潜在疾病。然而，影像没有提示原发性病变（如溶骨性破坏），所以可能性较低。
3. 第三可能：**应力性骨折** – 但这通常不是急性创伤导致的；摔伤更可能是直接暴力骨折。

在骨科中：

- 急性股骨干骨折是常见外伤。
- 病理性骨折：如果患者有骨质疏松、肿瘤等，但这里病史没有提示（仅摔伤），影像显示新鲜骨折无旧病变。
- 其他：如软组织损伤或感染，但MRI显示骨折相关。

更准确的列表：

1. 右股骨干急性骨折
2. 伴骨质疏松的病理性骨折（可能性低）
3. 骨髓炎？但无慢性炎症迹象；时间太短。

基于标准知识：

- 在创伤后8小时，影像学显示骨折，最可能诊断是股骨干骨折。
- 其他可能性：如果骨折线不规则，需考虑肿瘤，但这里是急性伤，所以不太可能。

列出3个具体疾病：

1. 右股骨干骨折（新鲜）
2. 由于骨质疏松导致的病理性骨折
3. 骨肉瘤或其他原发性骨肿瘤？但影像无特征；更可能不是。

在鉴别诊断中：

- 第一：创伤性骨折
- 第二：病理性骨折（如果考虑潜在疾病）
- 第三：其他，如骨坏死

但这里，所有证据指向急性外伤骨折。

临床建议：

- 下一步：确认诊断后，需固定和手术；例如，切开复位内固定（ORIF）。
- 具体：由于是股骨干骨折，通常需要手术治疗。建议住院、术前准备、进行手术干预。
- 也需排除其他损伤：如血管神经检查。

现在，结构化响应：

1. 影像学描述：客观描述特征。
2. 疾病诊断：3个，按可能性排序。
3. 临床建议：具体步骤。

确保语言专业、准确。

影像学描述：
X线片显示右股骨干中段可见明显骨折线，骨皮质连续性中断，断端轻度错位及成角畸形；CT轴向扫描证实多发骨折碎片，骨皮质不完整，髓腔内见高密度影（提示急性出血），周围软组织肿胀；MRI T2加权像示骨折区域骨髓信号弥漫增高（呈高信号，符合水肿/出血表现），邻近肌肉及软组织亦见广泛高信号区（提示血肿及炎症反应）。

结合病史与影像，按可能性从大到小的3个具体疾病诊断：

1. 右股骨干急性创伤性骨折（最可能）—— 病史为摔伤后8小时疼痛，X线、CT及MRI均清晰显示新鲜骨折特征（骨皮质中断、碎片化、髓腔出血及软组织水肿），符合急性外伤机制。
2. 骨质疏松相关病理性骨折（可能性中等）—— 患者54岁男性，存在骨质疏松风险；影像虽无明显溶骨性病变，但骨折形态不规则且轻微暴力即可致损，需排除潜在骨质代谢异常。
3. 低级别骨肉瘤继发病理性骨折（可能性较低）—— 影像未见典型肿瘤特征（如软组织肿块、Codman三角或骨膜反应），但需警惕罕见原发性骨肿瘤在创伤后诱发骨折，尤其患者年龄处于发病高峰。

临床建议：

1. 立即行患肢制动（使用夹板固定）并抬高，避免二次损伤；
2. 住院完善术前检查（包括血常规、凝血功能及下肢血管神经评估），优先安排急诊手术治疗——推荐切开复位内固定（ORIF）或髓内钉固定术以恢复骨连续性；
3. 同步行骨密度检测及肿瘤标志物筛查，排除病理性骨折可能；术后24小时内复查X线确认复位质量，并启动抗感染、镇痛及康复计划。
