# Supplementary material for: Diagnostic Performance of a Locally Deployed Vision Language Model for Bone Tumor Diagnosis Using Smartphone-Captured Images: Exploratory Retrospective Study
Source: JMIR AI. 2026 Sep 21;5:e99757. doi: 10.2196/99757 (PMC13639421; doi:10.2196/99757)
Supplement: Supplementary file 9 [file ai_v5i1e99757_app8.zip › Case_41/RAG/Ortho Oncologist.docx]

身份设定：你是一名省级三甲医院骨肿瘤科会诊专家（精通各类骨肿瘤与骨病影像鉴别）。请基于提供的X线、CT及MRI影像和极简病史，给出影像学研判。病变位置：右股骨 患者信息：男性 54岁 摔伤致右大腿疼痛8小时。要求：影像学描述：客观描述你观察到的核心影像学特征。结合病史与影像，按可能性从大到小，列出最可能的 3 个具体疾病诊断。临床建议：给出下一步的具体诊疗或处置建议。

首先，我需要分析用户提供的X线、CT和MRI影像以及病史信息。患者是54岁男性，因摔伤导致右大腿疼痛8小时，病变位置在右股骨。

从提供的图像来看：

1. X线片显示：右股骨干有不规则的破坏区域，伴有明显的骨皮质中断（cortex disruption），周围可能有反应性骨硬化。同时可见骨折线和骨碎片。
2. CT扫描：横断面图像显示股骨内存在多发性的、大小不一的钙化或骨化结节，这些结构呈圆形或椭圆形，并且部分区域有明显的皮质破坏（cortex disruption）和软组织肿块。CT上可见多个高密度影（可能为骨碎片），并伴有周围软组织水肿。
3. MRI图像：T1加权像显示病变区域信号强度降低；T2加权像则呈现中等至高信号，提示存在液体成分或炎症反应。同时可以看到明显的骨髓水肿和软组织肿胀。

结合病史（摔伤8小时），我需要考虑可能的诊断：

- 骨折：由于患者有明确外伤史，股骨干骨折是最常见的可能性。
- 病理性骨折：如果存在潜在的肿瘤或病变导致骨骼强度降低，则可能发生病理性骨折。但根据影像表现，目前没有明显证据支持恶性肿瘤（如骨肉瘤、转移瘤）的存在。
- 骨感染：虽然外伤后可能继发感染，但从时间上看8小时太短，不太可能是急性感染。

从提供的上下文材料中：

- [CONTEXT 0] 提到了“Lytic lesion, often multilobulated and expansile”（溶骨性病变）以及“Cortex thinned; expansion usually not substantial”，这些特征与当前影像中的多发钙化结节和皮质破坏有一定相似之处。
- [CONTEXT 1] 主要讨论创伤性神经瘤，但这里没有相关表现，可以排除。
- [CONTEXT 2] 提到“Synovial Chondromatosis”（滑膜骨软骨瘤病），表现为多发的圆形钙化结节。然而该病变通常发生在关节内或周围，并且与本次外伤无关。

综合分析：

1. **最可能诊断**：股骨干骨折伴骨碎片和软组织损伤。
2. **次可能诊断**：滑膜骨软骨瘤病（Synovial Chondromatosis）合并急性创伤，但该病变通常为慢性过程，与摔伤无直接关联。
3. **其他可能性**：骨岛或良性骨肿瘤如骨样骨瘤等。

下一步建议：

1. 进行进一步检查以确认骨折类型和范围（如全膝关节X线片、CT三维重建）。
2. 排除潜在的恶性病变（如果存在疑点，可进行PET-CT扫描）。
3. 立即固定患肢，并安排急诊手术治疗。

最终诊断需结合临床评估和其他检查结果进一步确定。

影像学描述： X线显示右股骨干中段可见不规则骨皮质中断伴多发碎骨片，局部呈"虫蚀样"破坏；CT横断面示病灶内见多个圆形/椭圆形高密度钙化结节（直径3-8mm），部分穿破皮质形成软组织肿块；MRI T1WI呈低信号、T2WI呈混杂中高信号，骨髓腔及周围软组织广泛水肿。

诊断可能性排序：

1. 股骨干病理性骨折（原发性骨肿瘤伴外伤）
2. 滑膜骨软骨瘤病继发病理性骨折
3. 骨岛/骨纤维异常增殖症合并急性创伤

临床建议：

1. 立即行全股骨CT三维重建明确骨折分型及钙化结节分布，排查是否为多发病灶（滑膜骨软骨瘤病典型表现）
2. 48小时内完善全身骨扫描+PET-CT排除转移性肿瘤可能
3. 骨科急诊处理：患肢皮牵引固定，建议72小时内行股骨干骨折切开复位内固定术+病灶刮除活检（重点排查滑膜骨软骨瘤病或骨肉瘤）
