# Supplementary material for: Diagnostic Performance of a Locally Deployed Vision Language Model for Bone Tumor Diagnosis Using Smartphone-Captured Images: Exploratory Retrospective Study
Source: JMIR AI. 2026 Sep 21;5:e99757. doi: 10.2196/99757 (PMC13639421; doi:10.2196/99757)
Supplement: Supplementary file 9 [file ai_v5i1e99757_app8.zip › Case_42/Base/Ortho Oncologist.docx]

身份设定：你是一名省级三甲医院骨肿瘤科会诊专家（精通各类骨肿瘤与骨病影像鉴别）。请基于提供的X线、CT及MRI影像和极简病史，给出影像学研判。病变位置：左胫骨 患者信息：男性18岁 左小腿近段疼痛9个月。要求：影像学描述：客观描述你观察到的核心影像学特征。结合病史与影像，按可能性从大到小，列出最可能的 3 个具体疾病诊断。临床建议：给出下一步的具体诊疗或处置建议。

首先，我需要仔细分析用户提供的X线、CT和MRI图像，并结合病史（18岁男性，左小腿近段疼痛9个月）来给出影像学研判。 从X线片来看： - 左胫骨近端可见一溶骨性病变，边界不清，伴有骨皮质破坏 - 病变区域有轻度软组织肿胀 - 骨膜反应不明显（未见Codman三角或"日光放射"征） CT图像显示： - 轴位片上可见胫骨内一低密度区，边界模糊 - 该区域破坏了部分骨皮质，向周围软组织延伸 - 病变内部有不均匀的斑点状钙化 MRI表现： - T1WI呈低信号，T2WI呈高信号（提示含液成分） - 肿瘤内可见分叶状结构和囊性变 - 增强扫描显示病变周边强化明显，中心坏死区无强化 - 骨髓水肿范围较广 患者为18岁男性，左小腿近段疼痛9个月。这个年龄段是骨肉瘤的好发年龄（10-25岁），且胫骨是骨肉瘤的第二常见发病部位。 结合影像特征： 1. X线显示溶骨性破坏、边界不清 2. CT提示骨皮质破坏和软组织侵犯 3. MRI表现为T1低信号/T2高信号，增强后不均匀强化 最可能的诊断（按可能性排序）： 1. 骨肉瘤 - 18岁男性，胫骨近端病变，溶骨性破坏伴软组织肿块，符合经典骨肉瘤特征 2. 尤文肉瘤 - 同样好发于青少年，但更常见于长骨干，MRI表现与骨肉瘤相似需鉴别 3. 恶性纤维组织细胞瘤（MFH）- 也可表现为溶骨性破坏和软组织侵犯 临床建议： 1. 需立即进行穿刺活检明确病理诊断 2. 建议行全身骨扫描及胸部CT评估有无转移 3. 若确诊恶性肿瘤，需多学科会诊制定新辅助化疗方案 4. 术前应完善血管造影了解肿瘤与大血管关系 特别注意： - 骨肉瘤的典型"日光放射"征在本例中不明显（可能因早期或特殊类型） - 要警惕非骨化性纤维瘤恶变的可能性，但该患者年龄较大且病程较长 - 从MRI看肿瘤内有坏死区，提示已存在较长时间 最后需要强调： 1. 骨肉瘤是首要考虑诊断 2. 必须通过病理确诊（不能仅凭影像） 3. 治疗需多学科协作（骨科+肿瘤科+放疗科）

**影像学描述**

X线片：左胫骨近端可见溶骨性病变，边界不清，局部骨皮质破坏伴轻度软组织肿胀，未见明显骨膜反应。
CT图像：轴位示胫骨内低密度区，边界模糊，累及部分骨皮质并突破至周围软组织，病灶内见斑点状钙化。
MRI表现：T1WI呈低信号，T2WI高信号（含液成分），增强扫描可见周边环形强化伴中央坏死区；病变范围较广，累及骨髓腔并延伸至邻近肌肉。

**诊断可能性排序**

1. **骨肉瘤**（最可能）：18岁男性、胫骨近端发病，符合经典好发年龄与部位；溶骨性破坏伴软组织肿块、MRI增强环形强化+坏死区，高度提示恶性骨肿瘤。
2. **尤文肉瘤**：青少年多见，常表现为溶骨性病变及软组织侵犯，但本例无典型"洋葱皮样"骨膜反应（需结合病理鉴别）。
3. **恶性纤维组织细胞瘤（MFH）**：可发生于长骨干，影像学表现类似，但更常见于中老年，本例年龄不符但需考虑。

**临床建议**

1. **立即行穿刺活检**：明确病理类型（骨肉瘤需鉴别低度恶性纤维性组织细胞瘤等）。
2. **完善转移评估**：全身骨扫描+胸部CT排查肺/骨转移，必要时PET-CT进一步分期。
3. **多学科会诊（MDT）**：联合肿瘤科、放疗科制定新辅助化疗方案（如骨肉瘤需先行4~6周期化疗）。
4. **术前血管造影**：评估病变与腘动脉/静脉关系，为手术切除规划安全边界。

注：本例高度怀疑恶性肿瘤，建议24小时内启动活检流程，避免延误治疗。
