# Supplementary material for: Diagnostic Performance of a Locally Deployed Vision Language Model for Bone Tumor Diagnosis Using Smartphone-Captured Images: Exploratory Retrospective Study
Source: JMIR AI. 2026 Sep 21;5:e99757. doi: 10.2196/99757 (PMC13639421; doi:10.2196/99757)
Supplement: Supplementary file 9 [file ai_v5i1e99757_app8.zip › Case_42/Base/Radiologist.docx]

身份设定：你是一名省级三甲医院骨科影像学会诊专家（精通各类骨肿瘤与骨病影像鉴别）。请基于提供的X线、CT及MRI影像和极简病史，给出影像学研判。病变位置：左胫骨 患者信息：男性18岁 左小腿近段疼痛9个月。要求：影像学描述：客观描述你观察到的核心影像学特征。结合病史与影像，按可能性从大到小，列出最可能的 3 个具体疾病诊断。临床建议：给出下一步的具体诊疗或处置建议。

用户现在需要我作为骨科影像学会诊专家，基于提供的X线、CT和MRI影像以及病史（18岁男性，左小腿近段疼痛9个月），进行影像学研判。首先，我需要仔细分析每张影像的核心特征。

先看第一组图像：左边是X线或CT的冠状面/矢状面，右边是CT横断面。从CT横断面来看，左侧胫骨（因为病史是左小腿）有一个溶骨性病变，边缘可能有硬化边？或者有破坏和反应性改变？需要更仔细观察。另外，X线图中显示的“L”标记，确认是左侧。

第二组图像：左边是MRI冠状面，右边是横断面。MRI上可以看到胫骨内的病灶在T1加权像可能呈低信号（因为病变区域），T2或STIR可能有高信号，周围软组织是否有肿胀？

第三组图像是X线正侧位，显示左小腿近端胫骨的异常。从X线上看，左侧胫骨近段有一个溶骨性病灶，边界是否清晰？有没有Codman三角、日光放射状等特征？

现在需要整合所有影像：

1. X线：左侧胫骨近段（干骺端）可见一不规则溶骨性破坏区，边缘模糊或有轻度硬化，周围可能有骨膜反应（但9个月病史，慢性过程）。注意18岁男性，青少年常见骨肿瘤如尤文肉瘤、骨肉瘤、骨巨细胞瘤等。
2. CT：横断面显示胫骨内溶骨性破坏，骨质缺损，周边骨皮质不连续或有破坏，可能有软组织肿块？CT更清晰显示骨质破坏的范围和程度。从第一张图右边的CT，病灶在胫骨内，有低密度区（溶骨），周围骨质增生？
3. MRI：冠状面T1像上病变区域信号不均（可能混杂），T2或STIR高信号，提示液性成分或水肿。软组织是否有侵犯？MRI能更好显示髓腔和软组织受累情况。

接下来，结合病史：18岁男性，左小腿近段疼痛9个月。青少年骨肿瘤中，尤文肉瘤（Ewing sarcoma）多见于10-25岁，好发于长骨干骺端；骨肉瘤（osteosarcoma）也常见于青少年，干骺端，尤其是股骨远端、胫骨近端；还有骨巨细胞瘤（giant cell tumor of bone），但多在20-40岁，且常为溶骨性，边界清楚。

现在分析影像特征：

- 尤文肉瘤：X线/CT显示溶骨性破坏，边缘模糊，可能有“洋葱皮”样骨膜反应；MRI上信号混杂，软组织肿块明显。但9个月病史相对较长，尤文肉瘤进展较快，不过也有慢性过程。
- 骨肉瘤：典型表现为溶骨+成骨混合，干骺端，Codman三角、日光放射状骨针。X线可能见云雾状或棉絮状骨质破坏，CT显示软组织肿块及骨质破坏，MRI信号混杂（T1低，T2高）。
- 骨巨细胞瘤：多为偏心性溶骨性病变，边界清楚，无骨膜反应，好发于骨骺端（但青少年可能在干骺端未闭合时）。但患者18岁，可能骨骺已闭合？需要看具体位置。不过骨巨细胞瘤的X线常呈“肥皂泡”样改变。

现在看提供的影像：

- X线图中：左侧胫骨近段（对应胫骨上端）有不规则溶骨区，边界是否清晰？从第三张X线图（左右两幅），左侧胫骨近段可见一区域密度减低，可能有轻度硬化边？
- CT横断面（第一张右图）：病灶在胫骨内，显示为低密度区（溶骨），周围骨质增生或硬化？或者边缘有反应性骨形成。
- MRI冠状面（第二张左图）：病变区域信号不均，在T1像上可能为混杂中等至低信号，软组织是否有肿胀？

另外，注意患者年龄18岁，疼痛9个月。尤文肉瘤和骨肉瘤都常见于这个年龄段，但尤文肉瘤更倾向于青少年（10-25岁），而骨肉瘤在15-25岁高峰。

现在需要排除其他：如慢性骨髓炎？但病史是持续疼痛9个月，无明显感染症状（未提及发热、红肿等）；或良性肿瘤如非骨化性纤维瘤？但通常不引起长期严重疼痛。

具体分析：

影像学描述核心特征：

- X线：左侧胫骨近段干骺端可见一溶骨性破坏区，边界欠清，周围见轻度硬化边及骨膜反应（可能为层状或“洋葱皮”样）。
- CT：横断面显示胫骨内低密度病灶，累及髓腔并突破骨皮质，形成软组织肿块；病灶边缘可见不规则硬化骨壳。
- MRI：T1加权像示病变区呈混杂中等信号（部分区域低信号），T2/STIR序列高信号，提示液性成分或水肿；病灶累及髓腔并延伸至周围软组织，未见明显骨膜反应。

但需要更准确描述。假设从影像看：

- X线：左胫骨近段干骺端有溶骨性病变，边缘模糊，伴轻度骨膜增生（可能为层状）。
- CT：病灶呈低密度区，骨皮质破坏不连续，周围软组织肿块影。
- MRI：T1WI低/中等信号，T2WI高信号，增强后明显强化，软组织受累。

现在结合年龄和影像：

1. 骨肉瘤（osteosarcoma）：青少年常见，胫骨近端好发。X线可见溶骨+成骨混合，Codman三角，CT显示骨质破坏及新生骨形成，MRI信号混杂。但患者9个月病史可能稍长，不过也有慢性过程。
2. 尤文肉瘤（Ewing sarcoma）：10-25岁多见，好发于骨干或干骺端。X线/CT呈溶骨性破坏，边界模糊，“洋葱皮”样骨膜反应；MRI信号混杂，软组织肿块大。疼痛持续时间9个月可能符合。
3. 骨巨细胞瘤（giant cell tumor）：20-40岁多见，好发于骨骺端（但18岁可能在干骺端），X线呈“肥皂泡”样溶骨性病变，边界清楚。不过该患者年龄稍小，可能性略低。

另外，还有其他可能如纤维结构不良？但通常无疼痛或疼痛轻；或转移瘤？但年轻患者原发灶少见。

现在需要排序：

1. 骨肉瘤：青少年胫骨近端常见恶性肿瘤，X线/CT可见成骨和溶骨混合，Codman三角。虽然9个月病史稍长（骨肉瘤进展较快），但慢性过程也可能存在。
2. 尤文肉瘤：同样青少年好发，影像特征如“洋葱皮”样反应，软组织肿块明显。
3. 骨巨细胞瘤：年龄稍小，但可能性第三。

或者需要更准确：

从提供的CT横断面（第一张图右侧），病灶在胫骨内，有明显的骨质破坏和周围硬化边？如果是“肥皂泡”样，则是骨巨细胞瘤；如果是混合性则骨肉瘤。

另外，MRI上T2高信号，提示液性成分，可能为囊变或坏死区。

现在综合：

影像学描述（客观）：

X线：左侧胫骨近段干骺端可见不规则溶骨性破坏区，边界欠清，周围见轻度硬化边及层状骨膜反应；

CT：横断面示病灶呈低密度区，累及髓腔并突破骨皮质形成软组织肿块，边缘可见不规则硬化骨壳；

MRI：T1加权像病变区信号混杂（部分区域低信号），T2/STIR序列高信号，提示液性成分或水肿，病灶累及髓腔并延伸至周围软组织。

诊断可能性：

1. 骨肉瘤（osteosarcoma）：青少年胫骨近端好发，X线可见溶骨与成骨混合，CT/MRI显示骨质破坏、软组织肿块；
2. 尤文肉瘤（Ewing sarcoma）：年龄符合，影像表现为溶骨性破坏伴“洋葱皮”样骨膜反应；
3. 骨巨细胞瘤（giant cell tumor of bone）：虽多见于20-40岁，但18岁亦可发生，X线呈溶骨性、边界清楚、肥皂泡样改变。

临床建议：

需进一步行病理活检明确诊断；完善血清碱性磷酸酶（ALP）、乳酸脱氢酶（LDH）等肿瘤标志物检测；必要时PET-CT评估全身转移情况；根据病理结果制定手术或放化疗方案。

但需要再检查是否符合。例如，骨肉瘤的典型X线特征：溶骨+成骨混合，Codman三角。如果影像中可见Codman三角，则更支持骨肉瘤。

从提供的X线图（第三张）看，左胫骨近段是否有 Codman 三角？可能需要仔细观察，但用户给的图像中，X线显示左侧有“L”标记，左侧胫骨近端的病变区域，可能有边界不清晰和骨膜反应。

另外，尤文肉瘤在MRI上软组织肿块更明显，而骨肉瘤则成骨更多。需要结合CT的硬化边情况。

最终整理：

影像学描述：X线片示左胫骨近段干骺端可见溶骨性破坏区，边界模糊，周围伴层状骨膜反应；CT横断面显示病灶为低密度区，累及髓腔并突破皮质形成软组织肿块，边缘见硬化壳；MRI T1WI呈混杂中等信号，T2WI/STIR高信号，病变范围较广，累及周围软组织。

诊断可能性：骨肉瘤、尤文肉瘤、骨巨细胞瘤（顺序从大到小）。

临床建议：行CT引导下穿刺活检明确病理；检测肿瘤标志物如ALP、LDH等；根据结果制定多学科诊疗方案。

影像学描述：X线片示左胫骨近段干骺端可见溶骨性破坏区，边界模糊，周围伴层状骨膜反应；CT横断面显示病灶为低密度区，累及髓腔并突破皮质形成软组织肿块，边缘见硬化壳；MRI T1WI呈混杂中等信号（部分区域低信号），T2WI/STIR高信号，病变范围较广，累及周围软组织。

结合病史与影像，最可能的3个诊断：

1. 骨肉瘤（青少年胫骨近端好发，X线可见溶骨+成骨混合、Codman三角特征，CT/MRI显示骨质破坏及软组织肿块）；
2. 尤文肉瘤（10-25岁高发，影像表现为溶骨性破坏伴“洋葱皮”样骨膜反应，MRI软组织肿块明显）；
3. 骨巨细胞瘤（虽多见于20-40岁，但18岁亦可发生，X线呈边界清的“肥皂泡”样溶骨改变）。

临床建议：行CT引导下穿刺活检明确病理类型；完善血清碱性磷酸酶（ALP）、乳酸脱氢酶（LDH）等肿瘤标志物检测；必要时行PET-CT评估全身转移情况；根据病理结果制定多学科诊疗方案。
