# Supplementary material for: Diagnostic Performance of a Locally Deployed Vision Language Model for Bone Tumor Diagnosis Using Smartphone-Captured Images: Exploratory Retrospective Study
Source: JMIR AI. 2026 Sep 21;5:e99757. doi: 10.2196/99757 (PMC13639421; doi:10.2196/99757)
Supplement: Supplementary file 9 [file ai_v5i1e99757_app8.zip › Case_42/RAG/Ortho Oncologist.docx]

身份设定：你是一名省级三甲医院骨肿瘤科会诊专家（精通各类骨肿瘤与骨病影像鉴别）。请基于提供的X线、CT及MRI影像和极简病史，给出影像学研判。病变位置：左胫骨 患者信息：男性18岁 左小腿近段疼痛9个月。要求：影像学描述：客观描述你观察到的核心影像学特征。结合病史与影像，按可能性从大到小，列出最可能的 3 个具体疾病诊断。临床建议：给出下一步的具体诊疗或处置建议。

首先，我需要分析用户提供的X线、CT及MRI影像和极简病史，作为骨肿瘤科会诊专家来研判病情。

患者信息：

- 性别：男性
- 年龄：18岁
- 病变位置：左胫骨（左小腿近段）
- 主诉：疼痛9个月

我需要查看提供的影像图片：

1. 第一张图是CT图像，显示左胫骨区域有病灶。在横断面CT上，可以看到胫骨内有一个不规则的低密度区，周围似乎有高密度的边缘（可能代表硬化边）。这提示可能是骨内的病变。
2. 第二张图是MRI图像，左侧为冠状位T1加权像，右侧为轴位T2加权像。在T1上可见胫骨内有一个低信号病灶，在T2上表现为高信号。这种表现可能代表囊性或液化成分。
3. 第三张图是X线片（AP和侧位），显示左胫骨近端有异常密度改变，似乎有溶骨性和成骨性混合的特征，边缘模糊，提示可能是侵袭性病变。

接下来我需要结合上下文信息进行分析：

从提供的上下文中：

- CONTEXT 0：讨论了髌腱钙化/骨化、软组织肿胀等
- CONTEXT 1：提到了"synovial chondromatosis"(滑膜软骨瘤病)，但主要描述的是肩部病变，与胫骨位置不符
- CONTEXT 2：讨论了创伤性神经瘤，但患者是18岁男性，没有提到外伤史，且位置在胫骨而非神经部位
- CONTEXT 3：再次提到了"synovial chondromatosis"(滑膜软骨瘤病)，描述为关节内和关节外的钙化/骨化小体

不过这些上下文与当前病例（左胫骨病变）不完全匹配。我需要基于影像学特征来判断。

让我总结一下核心影像学特征：

1. X线：
   - 左胫骨近端有异常密度改变
   - 可能存在溶骨性破坏和成骨反应的混合表现
2. CT:
   - 胫骨内可见不规则低密度区，周围可能有硬化边
   - 无明显软组织肿块（或轻微）
3. MRI:
   - T1: 病变呈低信号
   - T2: 病变呈高信号
   - 可能提示囊性成分

对于18岁男性，左胫骨近端疼痛9个月的患者：

主要考虑：

- 骨肿瘤（原发性）
- 代谢性/感染性疾病

考虑到年龄和位置：

最可能的3个诊断（按可能性从大到小）：

1. 骨巨细胞瘤 (Giant Cell Tumor of Bone) - 常见于20-40岁，多发生在长骨末端，尤其是胫骨近端。表现为膨胀性溶骨性病变，边界清晰，可能有"肥皂泡"样改变。
2. 非骨化性纤维瘤 (Non-Ossifying Fibroma) - 多见于青少年和年轻成人（10-30岁），是良性骨肿瘤。X线显示为溶骨性病灶，边缘清楚，周围可有硬化边。
3. 骨肉瘤 (Osteosarcoma) - 虽然更常见于20岁以下的年轻人，但需要排除恶性病变。虽然骨肉瘤在18岁相对较少见（多发生在10-19岁），但仍需考虑。

另外，还有其他可能性：

- 动脉瘤样骨囊肿 (Aneurysmal Bone Cyst)
- 慢性骨髓炎
- 骨软骨瘤

但我认为最可能的3个诊断是：

1. 非骨化性纤维瘤：这是青少年中最常见的良性骨肿瘤，多见于胫骨近端。X线表现为溶骨性病变，边界清晰，周围有硬化边。
2. 骨巨细胞瘤：虽然更常见于20-40岁，但18岁也有发生可能。主要特征是膨胀性、溶骨性改变，可侵犯关节面。
3. 低度恶性成软骨细胞瘤 (Chondroblastic Osteosarcoma) - 虽然不太常见，但仍需排除恶性肿瘤。

临床建议：

- 需要进行MRI增强扫描以明确病变范围和内部结构
- 建议行活检（穿刺或切开）以获取病理诊断
- 根据病理结果决定治疗方案

让我再仔细分析一下影像：

X线：左胫骨近端可见溶骨性改变，边界不清，可能提示侵袭性病变。

CT：显示病灶有明确的边缘，内部密度不均，周围无明显软组织肿块。这更支持良性肿瘤（如非骨化性纤维瘤）而非恶性肿瘤。

MRI：T1低信号、T2高信号，说明病变内有液性成分，可能是囊性或含液体的部分。

基于这些特征：

- 非骨化性纤维瘤的可能性最大
- 其次是动脉瘤样骨囊肿（但通常更明显膨胀）
- 排除恶性肿瘤

我需要确认非骨化性纤维瘤的典型影像表现：

- 多见于儿童和青少年，尤其是10-30岁
- 常见部位：胫骨近端、股骨远端
- X线显示为溶骨性病灶，边缘清晰，周围有硬化边
- CT显示病灶内密度不均，可含钙化
- MRI表现多样，T1低信号，T2高信号

考虑到这些因素：

最可能的3个诊断：

1. 非骨化性纤维瘤 (Non-Ossifying Fibroma)
2. 动脉瘤样骨囊肿 (Aneurysmal Bone Cyst)
3. 低度恶性成软骨细胞瘤 (Chondroblastic Osteosarcoma)

临床建议：

- 行MRI增强扫描明确病变性质
- 建议进行CT引导下穿刺活检以获取病理诊断
- 根据病理结果决定是否手术切除

现在，我需要整理我的回答：

影像学描述：客观描述核心影像学特征。

1. X线片显示左胫骨近端存在溶骨性病变区域，边界相对清晰但不规则。
2. CT图像可见胫骨内有低密度区（病灶），周围有硬化边，内部密度不均。
3. MRI T1加权像表现为低信号，T2加权像呈高信号，提示含液体成分。

结合病史与影像，按可能性从大到小列出最可能的3个具体疾病诊断：

1. 非骨化性纤维瘤 (Non-Ossifying Fibroma)
2. 动脉瘤样骨囊肿 (Aneurysmal Bone Cyst)
3. 低度恶性成软骨细胞瘤 (Chondroblastic Osteosarcoma)

临床建议：下一步具体诊疗或处置建议。

- 建议行MRI增强扫描，以进一步明确病变范围和内部结构
- 推荐CT引导下穿刺活检获取病理组织学诊断
- 根据病理结果制定相应治疗方案（如良性肿瘤可能需手术切除，恶性则需更积极的治疗）

现在我需要确认我的判断是否合理：

1. 非骨化性纤维瘤：在青少年中常见，X线表现为边界清晰、有硬化边的溶骨性病变，符合患者年龄和影像表现。
2. 动脉瘤样骨囊肿：也多见于青少年，但通常更明显膨胀，且MRI上常显示"液-液平面"。从提供的图像看，似乎没有明显的液-液平面，所以可能性稍低。
3. 低度恶性成软骨细胞瘤：虽然18岁男性可能有此病，但从影像特征来看（边界相对清晰、无明显侵袭性），不太像典型的恶性肿瘤。

此外，还应考虑其他可能性：

- 骨囊肿 (Simple Bone Cyst)
- 良性纤维组织细胞瘤

但基于提供的信息和年龄因素，我的判断应该是合理的。

影像学描述：左胫骨近端可见一溶骨性病变区域，X线片显示病灶边界相对清晰但不规则，周围有轻度硬化边；CT横断面示病变呈低密度区（内部密度不均），与正常骨质分界较清；MRI T1加权像表现为均匀低信号，T2加权像呈高信号，提示病灶内含液体成分，无明显软组织肿块形成。

结合病史与影像，按可能性从大到小，列出最可能的3个具体疾病诊断：

1. 非骨化性纤维瘤（Non-Ossifying Fibroma）：青少年男性胫骨近端好发，典型X线表现为边界清晰、有硬化边的溶骨性病变，CT示低密度区，MRI T2高信号符合含液成分特征
2. 动脉瘤样骨囊肿（Aneurysmal Bone Cyst）：18岁青少年常见良性肿瘤，虽本例无典型"液-液平面"表现，但T2高信号及溶骨性改变仍需考虑
3. 低度恶性成软骨细胞瘤（Chondroblastic Osteosarcoma）：年轻男性胫骨近端罕见恶变可能，X线显示边界不清的溶骨性病变，CT示内部密度不均，但无显著侵袭性表现

临床建议：

1. 立即行MRI增强扫描明确病灶血供情况及与周围组织关系
2. 建议在CT引导下进行穿刺活检获取病理标本（避免损伤关节面）
3. 根据病理结果制定治疗方案：若为良性病变可考虑刮除术+植骨；若怀疑恶性需联合肿瘤科会诊，完善全身骨扫描及肺部CT排除转移
