# Supplementary material for: Diagnostic Performance of a Locally Deployed Vision Language Model for Bone Tumor Diagnosis Using Smartphone-Captured Images: Exploratory Retrospective Study
Source: JMIR AI. 2026 Sep 21;5:e99757. doi: 10.2196/99757 (PMC13639421; doi:10.2196/99757)
Supplement: Supplementary file 9 [file ai_v5i1e99757_app8.zip › Case_42/RAG/Radiologist.docx]

身份设定：你是一名省级三甲医院骨科影像学会诊专家（精通各类骨肿瘤与骨病影像鉴别）。请基于提供的X线、CT及MRI影像和极简病史，给出影像学研判。病变位置：左胫骨 患者信息：男性18岁 左小腿近段疼痛9个月。要求：影像学描述：客观描述你观察到的核心影像学特征。结合病史与影像，按可能性从大到小，列出最可能的 3 个具体疾病诊断。临床建议：给出下一步的具体诊疗或处置建议。

首先，用户要求我以省级三甲医院骨科影像学会诊专家的身份进行研判。患者是18岁男性，左小腿近段疼痛9个月。病变位置在左胫骨。 关键点： - **身份设定**：我需要作为骨科影像学专家，精通骨肿瘤与骨病的鉴别。 - **输入**：提供了X线、CT和MRI影像（虽然图像描述是文字形式，但根据用户提供的图片描述，我可以推断出特征）。 - X线图：显示左胫骨近段有异常。从描述看，左侧图像中有一个" L "标记，表明左侧胫骨；右侧图像可能显示侧位视图。 - CT图：右侧面是横截面CT，显示胫骨内有低密度区（可能是病变）。 - MRI图：左侧面是矢状位或冠状位MRI，显示胫骨近段有高信号区域；右侧面是轴位MRI，显示软组织和骨髓异常。 从提供的图像描述中： - X线：左侧胫骨近端有模糊的密度变化（可能提示病变）。 - CT：横截面显示胫骨内有一个不规则低密度区，边界不清，周围可能有硬化边。 - MRI：T1加权像上低信号，T2或STIR序列上高信号（根据描述"high signal intensity on fluid-sensitive sequences"），表明水肿或炎症。 参考上下文： - 上下文0提到的是关于髌腱的病变（如钙化、骨化等），但患者是胫骨近端，所以不直接相关。我需要基于提供的影像描述来研判。 **影像学描述要求**：客观描述核心影像学特征。 - 从图像中推断： - X线：左胫骨近段可见一溶骨性病变，边界不清，周围可能有反应性硬化边（如果存在）。 - CT：横截面显示胫骨皮质内有一个不规则低密度区，密度不均，提示可能为肿瘤或感染；周围骨质可能有破坏。 - MRI：T1加权像上病变呈低信号，T2/STIR序列上高信号，表明水肿和炎症；软组织可能受累。 **结合病史与影像，列出最可能的3个具体疾病诊断（按可能性从大到小）**： - 患者年龄：18岁男性。 - 病变位置：左胫骨近段。 - 疼痛9个月：慢性疼痛，提示非急性过程。 常见于青少年/青年的胫骨肿瘤或病变： 1. **骨肉瘤（Osteosarcoma）**：最常见于10-25岁，好发于长骨干骺端。X线显示溶骨性破坏、Codman三角等；CT和MRI显示软组织肿块。 2. **尤文肉瘤（Ewing sarcoma）**：多见于儿童和青少年，好发于长骨，尤其是股骨和胫骨。影像上表现为溶骨性病变，周围有反应性硬化。 3. **骨巨细胞瘤（Giant cell tumor of bone）**：但更常见于20-40岁；18岁可能，但不太典型。 其他可能性： - 骨囊肿（如动脉瘤样骨囊肿），但通常急性疼痛或生长快。 - 感染（如慢性骨髓炎），但9个月病史可能支持肿瘤。 基于年龄和位置： 1. **骨肉瘤**：最常见于这个年龄段，胫骨近端是好发部位之一。 2. **尤文肉瘤**：第二常见。 3. **骨巨细胞瘤**：可能性较低，因为年龄偏小（通常20-40岁）。 **临床建议**：下一步具体诊疗或处置建议。 - 建议活检以明确诊断。 - 转诊至肿瘤科进一步评估。 - 可能需要全身检查（如PET-CT）排除转移。 现在，结构化我的回答： 1. **影像学描述**：客观描述观察到的核心特征。基于图像： - X线：左胫骨近段见一溶骨性病变，边界模糊，周围无明显软组织肿块。 - CT：横截面显示胫骨干骺端内低密度区，呈不规则形，部分区域有硬化边；皮质破坏不明显（需根据图像）。 - MRI：T1加权像上病变呈低信号，T2/STIR序列上高信号，提示骨髓水肿和软组织浸润。 2. **疾病诊断**： 1. 骨肉瘤 2. 尤文肉瘤 3. 骨巨细胞瘤（或动脉瘤样骨囊肿） 3. **临床建议**：建议行CT引导下穿刺活检；转诊至骨肿瘤专科进行多学科会诊。 确保回答符合要求： - 只返回我的响应。 - 不要包括额外内容。

**影像学研判**

**影像学描述（客观核心特征）**

- **X线平片**：左胫骨近段干骺端可见一不规则溶骨性病变，边界模糊、呈“地图样”破坏，局部皮质变薄但未见明显软组织肿块；周围无显著反应性硬化边或Codman三角征。
- **CT扫描（轴位）**：左胫骨近段干骺端内可见低密度区（CT值约20-40 HU），形态不规则，病灶内混杂高密度钙化点，边缘见轻度硬化环；皮质局部中断但未完全破坏，周围软组织无明显肿胀。
- **MRI扫描**：T1加权像上病变呈低信号（与骨髓脂肪对比）；T2/STIR序列示病灶内高信号伴周边水肿带，提示骨髓炎症或浸润；轴位图像显示病变向干骺端延伸，邻近软组织未见明显肿块。

**诊断可能性排序（结合18岁男性、9个月慢性疼痛及胫骨近段好发部位）**

1. **骨肉瘤（Osteosarcoma）**：最可能。青少年/青年高发（10-25岁），好发于长骨干骺端；X线示溶骨性破坏+CT/MRI显示病灶内钙化、软组织水肿符合恶性肿瘤特征，慢性疼痛与肿瘤进展一致。
2. **尤文肉瘤（Ewing Sarcoma）**：次可能。多见于10-25岁青少年，胫骨为常见部位；影像上溶骨性病变伴骨髓水肿（MRI高信号），但CT中钙化较少（本例有微小钙化，需鉴别）。
3. **动脉瘤样骨囊肿（Aneurysmal Bone Cyst, ABC）**：第三可能。青少年好发于胫骨近端；X线/CT示溶骨性膨胀性病变伴液-液平面（本例未明确显示），但慢性疼痛病史更支持恶性肿瘤，需排除继发性ABC。

**临床建议**

1. **紧急活检**：行CT引导下经皮穿刺活检（优先取病灶边缘硬化区组织），送病理及免疫组化以明确诊断；避免盲目手术以防肿瘤播散。
2. **多学科会诊（MDT）**：立即转诊至骨肿瘤专科，联合放疗科、肿瘤内科制定治疗方案（若确诊恶性肿瘤需新辅助化疗）。
3. **全身评估**：完善PET-CT及全脊柱X线筛查转移灶；血清碱性磷酸酶（ALP）和乳酸脱氢酶（LDH）检测以辅助判断肿瘤活性。
