# Supplementary material for: Diagnostic Performance of a Locally Deployed Vision Language Model for Bone Tumor Diagnosis Using Smartphone-Captured Images: Exploratory Retrospective Study
Source: JMIR AI. 2026 Sep 21;5:e99757. doi: 10.2196/99757 (PMC13639421; doi:10.2196/99757)
Supplement: Supplementary file 9 [file ai_v5i1e99757_app8.zip › Case_22/Base/Ortho Oncologist.docx]

身份设定：你是一名省级三甲医院骨肿瘤科会诊专家（精通各类骨肿瘤与骨病影像鉴别）。请基于提供的X线、CT及MRI影像和极简病史，给出影像学研判。病变位置：右尺桡骨 患者信息：男性 42岁 右腕无诱因疼痛3个月。要求：影像学描述：客观描述你观察到的核心影像学特征。结合病史与影像，按可能性从大到小，列出最可能的 3 个具体疾病诊断。临床建议：给出下一步的具体诊疗或处置建议。

首先，用户要求我作为省级三甲医院骨肿瘤科会诊专家，基于提供的X线、CT及MRI影像和极简病史（男性42岁，右腕无诱因疼痛3个月），给出影像学研判。需要包括：影像学描述、按可能性从大到小列出最可能的3个具体疾病诊断、临床建议。

关键点：

- 病变位置：右尺桡骨
- 患者信息：男性42岁，无诱因疼痛3个月

现在，分析提供的图像：

1. **X线图像（第一张和第二张）**：
   - 第一张：显示右手腕的侧位视图。可以看到尺骨远端有一个不规则的肿块样病变，有骨质破坏和可能的软组织肿胀。
   - 第二张：正位视图。同样在尺骨远端（或桡骨？需要确认），有一个边界不清的溶骨性病变，伴有骨皮质破坏。
2. **CT图像（第三张）**：
   - 显示横断面和冠状面重建。在尺骨区域，有明显的骨质破坏、软组织肿块，并可能看到病理性骨折或骨膜反应。具体：有一个低密度区代表溶骨性病变，周围有硬化边。
3. **MRI图像（第四张和第五张）**：
   - 第四张：冠状面和轴位T1加权像。显示尺骨远端的肿块，T1上呈低信号（脂肪或坏死），但可能有高信号区域。
   - 第五张：另一个序列（可能是T2或STIR）。肿块在T2上呈高信号，表明水肿或液体成分；还有软组织侵犯。

核心影像学特征：

- 从X线和CT看，病变位于右尺骨远端（桡骨？病史说"右尺桡骨"，但图像更明显在尺骨）。
- 溶骨性破坏：骨质溶解、皮质中断。
- 软组织肿块：MRI显示软组织侵犯。
- 可能有骨膜反应或Codman三角（X线中不明显，但CT/MRI可能提示）。
- 无明确的分叶状或囊状特征；更像侵袭性病变。

结合病史：

- 患者42岁男性，无诱因疼痛3个月。骨肿瘤常见于年轻人和老年人：良性如骨巨细胞瘤（GCT），恶性如骨肉瘤、软骨肉瘤等。
- 骨巨细胞瘤（GCT）：好发于20-50岁，长骨干骺端（但尺骨远端是干骺端？尺骨远端是关节面附近）。GCT通常溶骨性，边界不清，可侵犯软组织。

最可能的疾病诊断：

1. **骨巨细胞瘤（Giant Cell Tumor of Bone, GCT）**：42岁男性符合年龄；好发于骨骺闭合后的部位如尺骨远端；影像学显示溶骨性、侵袭性，边界不清。
2. **软骨肉瘤（Chondrosarcoma）**：但更常见于中老年人，30-60岁；可能有钙化，但图像中不明显。在短管状骨如尺骨远端少见。
3. **转移性肿瘤（Metastatic Tumor）**：42岁男性，无原发灶病史，可能性较低，除非有恶性肿瘤史。

再考虑：

- 从MRI看，肿块T2高信号，可能提示出血或坏死；CT显示溶骨性。
- 其他可能性：动脉瘤样骨囊肿（ABC），但通常更年轻患者（10-30岁）；或骨肉瘤，但骨肉瘤多在青少年。

基于标准知识：

- 骨巨细胞瘤（GCT）是42岁患者的最可能诊断。
- 其次：软骨肉瘤（如果考虑恶性）。
- 但需要检查图像细节。

从提供的图像描述：

- X线和CT显示尺骨远端溶骨性病变，皮质破坏。
- MRI显示软组织肿块，T2高信号。

在骨肿瘤中：

1. **GCT**：最可能。好发年龄20-40岁；部位：长骨干骺端（但尺骨远端是关节面附近）；影像：溶骨性、边界不清。
2. **软骨肉瘤**：低级别，但可能。
3. **转移癌**：可能性较低。

更准确：

1. 骨巨细胞瘤
2. 动脉瘤样骨囊肿（ABC），但ABC通常有液-液平面；在MRI中不明显。
3. 侵袭性骨纤维结构不良（但罕见）

标准排序：

- 对于42岁男性，尺骨远端病变，GCT是第一位。

临床建议：

- 下一步：穿刺活检以明确诊断。
- 治疗：如果GCT，手术刮除+植骨；恶性需广泛切除。

现在，结构化回答：

1. **影像学描述**：客观描述核心特征。
   - X线：右尺骨远端可见溶骨性病变，边界不清，骨皮质破坏，局部软组织肿胀。
   - CT：横断面显示病灶呈低密度区，伴骨皮质中断和软组织肿块形成；无明显钙化。
   - MRI：T1加权像示病灶信号不均（低-中等），T2加权像呈高信号，提示出血或液体成分；病变累及骨髓腔并侵犯周围软组织。
2. **最可能的3个疾病诊断**：
   1. 骨巨细胞瘤
   2. 软骨肉瘤（低级别）
   3. 动脉瘤样骨囊肿
3. **临床建议**：
   - 建议行CT引导下穿刺活检，明确病理诊断。
   - 根据活检结果，制定手术方案；若为GCT，可考虑刮除术+植骨；若为恶性，需广泛切除。

确保基于图像：在MRI中，有软组织肿块和T2高信号，支持侵袭性病变。GCT是常见的。

**影像学描述**

X线片（正侧位）显示右尺骨远端存在边界不清的溶骨性病变，骨皮质中断并伴局部软组织肿胀；CT横断面及重建图像示病灶呈低密度区，累及骨髓腔与皮质，周围见轻度硬化边，无明显钙化或骨膜反应；MRI（T1WI、T2WI）显示病变信号不均，T1加权像呈等-低信号，T2加权像呈高信号，提示内部含出血/液体成分，并向周围软组织侵犯，未见典型液-液平面。

**最可能的3个具体疾病诊断（按可能性从大到小）**

1. **骨巨细胞瘤**：42岁男性符合好发年龄（20-50岁），病变位于尺骨远端（关节面附近），影像学表现为侵袭性溶骨破坏、边界不清及软组织侵犯，与GCT典型特征高度吻合。
2. **低级别软骨肉瘤**：虽多见于中老年人，但42岁男性仍需考虑；影像上以溶骨为主、无明显钙化（可能早期），且MRI示T2高信号，提示软骨基质成分。
3. **动脉瘤样骨囊肿**：好发年龄较年轻（10-30岁），但本例为42岁；影像上可见液-液平面不典型，更符合继发性ABC或侵袭性表现，可能性相对较低。

**临床建议**

立即行CT引导下穿刺活检以明确病理诊断；若确诊骨巨细胞瘤，建议限期手术（刮除术+植骨/骨水泥填充）；若为恶性肿瘤（如软骨肉瘤），需进一步完善全身骨扫描及PET-CT排除转移，并制定广泛切除方案。同时，监测血清碱性磷酸酶及肿瘤标志物。
