# Supplementary material for: Diagnostic Performance of a Locally Deployed Vision Language Model for Bone Tumor Diagnosis Using Smartphone-Captured Images: Exploratory Retrospective Study
Source: JMIR AI. 2026 Sep 21;5:e99757. doi: 10.2196/99757 (PMC13639421; doi:10.2196/99757)
Supplement: Supplementary file 9 [file ai_v5i1e99757_app8.zip › Case_22/RAG/Ortho Oncologist.docx]

身份设定：你是一名省级三甲医院骨肿瘤科会诊专家（精通各类骨肿瘤与骨病影像鉴别）。请基于提供的X线、CT及MRI影像和极简病史，给出影像学研判。病变位置：右尺桡骨 患者信息：男性 42岁 右腕无诱因疼痛3个月。要求：影像学描述：客观描述你观察到的核心影像学特征。结合病史与影像，按可能性从大到小，列出最可能的 3 个具体疾病诊断。临床建议：给出下一步的具体诊疗或处置建议。

首先，我需要仔细分析用户提供的X线、CT和MRI影像资料，并结合患者信息（42岁男性，右腕无诱因疼痛3个月）进行研判。 从提供的影像来看： 1. X线片显示：右尺桡骨区域有异常病变。在正位和侧位X光片上可以看到尺骨近端有一个明显的、边界不清的溶骨性病灶，伴有轻度膨胀。 2. CT图像（第三张图）显示：该病灶位于尺骨近端，在横断面可见到皮质破坏和软组织肿块。CT能更清晰地显示骨质破坏范围及周围情况。 3. MRI图像（第四、五张图）显示：在T1加权像上，病变呈低信号；在T2加权像上呈高信号，并且有明显的液-液平面征象。此外，在冠状位和矢状位MRI上可见到病变内部的囊性成分。 结合提供的上下文资料： - 根据[CONTEXT 0]中提到"lytic lesion, often multilobulated and expansile"（溶骨性病灶，常呈多叶状并有膨胀）,"cortex thinned; expansion usually not substantial"（皮质变薄；扩张通常不明显）等特征。 - [CONTEXT 0]还提到"May develop more aggressive appearance with less distinct border, cortex breakthrough, and soft tissue mass"（可能发展为更侵袭性的外观，边界不清、骨皮质突破和软组织肿块），这与当前影像表现一致。 现在我需要结合这些信息进行分析： 1. 从X线看：尺骨近端有溶骨性病灶，边界不清晰，伴有轻度膨胀。 2. 从CT看：病变累及骨髓腔，皮质破坏并延伸至软组织，形成软组织肿块。 3. 从MRI看：病变在T1WI上呈低信号，在T2WI上呈高信号，并有液-液平面。这些特征提示病变为囊性或含血性成分。 根据这些影像学表现和患者的临床信息（42岁男性，右腕无诱因疼痛3个月），我需要考虑以下可能性： 1. 骨巨细胞瘤：这是最常见的骨肿瘤之一，在40岁以下人群常见。X线表现为溶骨性、膨胀性病灶，边界不清；CT显示皮质破坏和软组织肿块；MRI上多呈T2高信号，且常有液-液平面。 2. 动脉瘤样骨囊肿：通常在青少年至年轻成人中发生，X线表现类似骨巨细胞瘤（溶骨性、膨胀性病灶）；CT显示皮质破坏和软组织肿块；MRI上可见多房结构和液-液平面。 3. 骨肉瘤：虽然较少见于42岁患者，但也不能完全排除。X线表现为溶骨性和成骨性混合病变，边界不清；CT显示皮质破坏和软组织肿块；MRI上呈T1低信号、T2高信号。 结合所有信息： - 该患者的影像表现最符合"骨巨细胞瘤"的特征：溶骨性病灶、边界不清、皮质变薄、伴有软组织肿块，以及MRI上的液-液平面。 - 其次考虑"动脉瘤样骨囊肿"，因为也有类似的影像学表现（多房结构和液-液平面）。 - 第三可能是"骨肉瘤"，但考虑到患者的年龄（42岁），这种可能性相对较低。 临床建议： 1. 建议行MRI增强扫描以进一步明确病变性质、范围及与周围组织的关系。 2. 考虑进行活检术获取病理标本，这是确诊的关键步骤。 3. 由于该病变有皮质破坏和软组织肿块形成，需警惕恶性肿瘤的可能性，建议尽快转诊至骨肿瘤专科。 最后需要按照要求格式化输出： - 影像学描述：客观描述核心影像学特征 - 最可能的3个诊断（按可能性从大到小） - 临床建议 现在我将整理这些内容，以专业、严谨的方式进行表述。

**影像学描述**

X线片示右尺骨近端可见溶骨性病灶，呈不规则多叶状膨胀，边界模糊不清，皮质变薄但未见明显骨膜反应；CT显示病变累及髓腔并突破皮质形成软组织肿块，局部骨质破坏范围较广；MRI表现为T1WI低信号、T2WI高信号的囊性病灶伴液-液平面征象，冠状位及矢状位可见多房结构，病灶内信号不均，提示含血性成分。

**诊断可能性排序**

1. **骨巨细胞瘤**（最可能）：42岁男性符合典型发病年龄；X线/CT示溶骨性、膨胀性病变伴皮质破坏及软组织肿块；MRI液-液平面及多房结构高度提示此病。
2. **动脉瘤样骨囊肿**（次可能）：影像表现与GCT相似，但更常见于青少年，且液-液平面特征更显著；本例患者年龄偏大，可能性略低于GCT。
3. **恶性肿瘤（如骨肉瘤）**（需警惕）：虽X线/CT显示局部侵袭性征象（皮质突破、软组织肿块），但MRI未见明显成骨反应或肿瘤基质特征；结合年龄及病程，可能性相对较低。

**临床建议**

1. **立即行增强MRI检查**：明确病变血供情况、与周围神经血管关系，排除恶性转化可能。
2. **病理活检（首选CT引导下穿刺）**：获取组织标本进行免疫组化检测（如RANKL/CD68），确诊需结合病理诊断。
3. **多学科会诊**：建议骨肿瘤科、手外科联合制定治疗方案，若确诊GCT需评估手术切除范围及是否行辅助治疗（如地诺单抗）。
4. **动态随访**：短期内复查X线/超声监测病灶进展速度，警惕恶性转化风险。
