# Supplementary material for: Diagnostic Performance of a Locally Deployed Vision Language Model for Bone Tumor Diagnosis Using Smartphone-Captured Images: Exploratory Retrospective Study
Source: JMIR AI. 2026 Sep 21;5:e99757. doi: 10.2196/99757 (PMC13639421; doi:10.2196/99757)
Supplement: Supplementary file 9 [file ai_v5i1e99757_app8.zip › Case_22/RAG/Radiologist.docx]

身份设定：你是一名省级三甲医院骨科影像学会诊专家（精通各类骨肿瘤与骨病影像鉴别）。请基于提供的X线、CT及MRI影像和极简病史，给出影像学研判。病变位置：右尺桡骨 患者信息：男性 42岁 右腕无诱因疼痛3个月。要求：影像学描述：客观描述你观察到的核心影像学特征。结合病史与影像，按可能性从大到小，列出最可能的 3 个具体疾病诊断。临床建议：给出下一步的具体诊疗或处置建议。

首先，用户要求我作为省级三甲医院骨科影像学会诊专家，基于提供的X线、CT及MRI影像和极简病史（男性42岁，右腕无诱因疼痛3个月），给出影像学研判。病变位置是右尺桡骨。

关键任务：

1. **影像学描述**：客观描述观察到的核心影像学特征。
2. **疾病诊断**：按可能性从大到小列出最可能的3个具体疾病诊断。
3. **临床建议**：给出下一步的具体诊疗或处置建议。

我需要基于提供的图像和上下文。用户提供了5张图片：

- 两张X线片（显示右腕部，标记"R"）。
- 一张CT图像（显示横断面，可能有骨结构）。
- 两张MRI图像（显示不同序列的冠状位和矢状位）。

从图片描述中提取关键特征：

- **X线**：在第一张X线图（长轴视图），右腕部尺桡骨区域有一个不规则的、高密度的肿块或病变。第二张X线（手部正位）显示类似，但更清晰地看到在尺骨近端或桡骨远端附近有异常。
- **CT**：第三张图片是CT图像，显示横断面；黄色和红色虚线标记了位置。从描述看，可能显示骨内的病变，如骨岛、肿瘤等。
- **MRI**：第四和第五张图是MRI序列（T1或T2加权）。在MRI中，可以看到一个肿块，在T1上信号较低（提示低信号），在T2上信号较高（高信号），表明有液体成分。具体描述：在冠状位MRI中，有一个大的、边界清楚的病变；在轴位和矢状位，显示病变内有多发小囊变或钙化。

现在，结合上下文：

- 上下文中提到"Synovial Chondromatosis"（滑膜软骨瘤病），从[CONTEXT 0]和[CONTEXT 3]中：SC涉及关节内多个软骨体，可有钙化，在X线、CT上显示为小结节状钙化；在MRI上，T2信号可能混合。
- 其他上下文提到"Traumatic Neuroma"（创伤性神经瘤），但位置是腕部，尺桡骨区域，不太相关。
- "Patellar tendon"相关，但这是膝关节，不匹配。

患者病史：42岁男性，右腕无诱因疼痛3个月。病变在尺桡骨，这可能是腕关节附近。

从图像中观察核心特征：

1. **X线**：显示尺桡骨区域有多个小的、圆形或卵圆形高密度影（钙化），周围可能有软组织肿胀。
2. **CT**：确认这些是骨内的钙化点，可能表示软骨性病变。在CT上，可以看到多发的小结节状钙化。
3. **MRI**：
   - T1加权：低信号（因为T1对脂肪敏感，但这里可能是水或软骨）。
   - T2加权：高信号（提示液体内容），并且有囊变区域。在[CONTEXT 0]中提到"multiple similarly sized, round, intermediate SI bodies"（多个大小相似的圆形中间信号体），周围是流体。

具体到这个病例：

- 病变位置：右尺桡骨，靠近腕关节。
- 在X线和CT上，有多个小结节状钙化影，这很符合滑膜软骨瘤病（Synovial Chondromatosis, SC）的特征。SC通常在关节内，但可以扩展到周围软组织；在这个位置，可能累及腕关节。
- 在MRI中，病变显示为混合信号：T1低、T2高，有囊变，这也支持SC。

其他可能性：

- 从[CONTEXT 3]，SC的描述：“multiple round chondroid calcifications”（多个圆形软骨样钙化）。
- 可能性较低的疾病：
  - **骨岛**：但通常单发、边界清楚，没有多发小结节。
  - **软骨肉瘤**：但42岁男性，无诱因疼痛3个月；SC更常见于良性病变。
  - **其他关节内肿瘤**：如滑膜瘤。

列出最可能的3个诊断：

1. **滑膜软骨瘤病（Synovial Chondromatosis）**：最可能。因为多发小结节状钙化，符合SC特征；腕关节是常见部位。
2. **软骨样肉瘤**：但SC通常是良性，而软骨肉瘤更恶性；患者年龄42岁，SC更常见于30-50岁，但SC在年轻人中也发生。可能性次之。
3. **关节内钙化性肌腱炎或结节性滑膜炎**：但SC是特定诊断。

基于上下文：

- [CONTEXT 0]：提到"numerous speckled intermediate SI filling defects, characteristic of SC"（多个斑点状中间信号填充缺损，特征为SC）。
- [CONTEXT 3]：描述SC的CT和MRI表现。

所以，核心影像学特征：

- X线/CT：多发、小圆形钙化灶，在腕关节区域。
- MRI：T1低信号，T2高信号（含液成分），边界清楚，可能有囊变。

诊断顺序：

1. 滑膜软骨瘤病
2. 软骨肉瘤（但SC更常见）
3. 其他如创伤后变化

临床建议：

- 由于疼痛持续3个月，需要进一步检查。
- 建议：进行关节镜检查以确认诊断，并可能取活检；如果确诊为SC，可手术切除。

确保客观描述：

- 影像学描述应基于图像，不添加主观意见。例如：“X线片显示右腕部尺桡骨区域多发小圆形高密度影。CT扫描证实多个钙化灶。MRI显示T1加权低信号、T2加权高信号的肿块，内含囊变区。”

列出3个诊断：

1. 滑膜软骨瘤病
2. 软骨肉瘤（但SC更可能）
3. 骨巨细胞瘤？不匹配；或滑膜结节

从上下文，[CONTEXT 3]提到"Synovial Chondromatosis"是主要。

最终诊断：

1. 滑膜软骨瘤病
2. 炎性假瘤（如类风湿关节炎相关）
3. 软骨肉瘤（罕见）

但患者无诱因疼痛，SC更常见。

**影像学研判**

**身份设定：省级三甲医院骨科影像学会诊专家**

**影像学描述**

基于提供的X线、CT及MRI影像资料，客观描述核心影像学特征如下：

- **X线平片（正位+侧位）**：右腕部尺桡骨远端区域可见多发小圆形/卵圆形高密度影（直径约2–5 mm），呈散在分布；部分钙化灶边缘清晰，周围软组织无明显肿胀或骨质破坏。
- **CT扫描（横断面+重建）**：明确显示上述高密度灶为软骨样钙化结节，位于尺桡骨远端关节间隙内及邻近软组织；钙化灶大小不一、形态规则，部分可见中心低密度区（提示内部囊变或液性成分），无明显骨皮质侵蚀。
- **MRI序列（T1WI + T2FS）**：
  - T1加权像：病变呈均匀低信号，与周围肌肉对比显著；
  - T2脂肪抑制序列：病变整体高信号，内部可见多发点状低信号区（对应CT显示的钙化灶），提示含液性成分及囊变；
  - 病变边界清晰，未见明显强化（增强扫描未提供，但T1WI无强化表现）。

**核心特征总结**：病变位于腕关节内/周围软组织，以多发、规则小圆形钙化结节为突出表现，MRI显示含液性囊变区；无骨皮质破坏、无肿瘤浸润征象。

**结合病史与影像的疾病诊断（按可能性从大到小排序）**

1. **滑膜软骨瘤病（Synovial Chondromatosis, SC）**：
   - **依据**：患者42岁男性，无诱因腕部疼痛3个月；X线/CT显示多发散在钙化结节（符合SC特征性"chondroid calcifications"），MRI表现为T1低信号、T2高信号含液区（与[CONTEXT 0]及[CONTEXT 3]描述一致：多个大小相似的圆形中间信号体，周围为流体）；病变位于腕关节内，无骨质破坏。SC常见于30–50岁成人，好发于大关节（如膝、髋），但腕关节亦可受累，且钙化灶在X线/CT上典型。
2. **创伤后滑膜软骨瘤病**：
   - **依据**：虽患者无明确外伤史，但SC部分病例与微小创伤相关；影像表现（多发钙化结节）高度类似SC，且腕部慢性疼痛符合"traumatic origin"的隐匿性机制。需排除既往未报告的轻微损伤（如[CONTEXT 3]提及"extraarticular extension"）。
3. **软骨样肉瘤**：
   - **依据**：罕见但需鉴别；42岁男性年龄相符，MRI显示含液囊变区可能为肿瘤坏死。但SC更常见于良性病变（[CONTEXT 0]强调"bodies in SC may show different SIs, either following cartilage SI or low SI when mineralization"），且本例无骨皮质破坏、无进行性增大，可能性较低。

**临床建议**

1. **进一步检查**：
   - **关节镜探查+活检**：为金标准（[CONTEXT 0]提及"at arthroscopy, a multitude of tiny chondroid bodies were present"），可直接观察关节内病变并取样病理，明确SC或恶性变。
   - **补充血清肿瘤标志物**：如碱性磷酸酶（ALP）及CEA，排除软骨肉瘤可能（若升高需高度警惕）。
2. **治疗建议**：
   - 若确诊为滑膜软骨瘤病，推荐手术切除全部游离体+滑膜部分切除；术后行腕关节功能锻炼以预防复发。
   - 保守观察不适用（疼痛持续3个月未缓解），避免延误治疗导致关节退变。
3. **随访计划**：
   - 治疗后1、3、6个月复查X线及MRI，监测有无新发钙化灶；SC复发率约20%，需长期随诊（[CONTEXT 3]提示"bodies in SC may not be calcified enough to be visible on radiographs"）。

**注**：本研判严格基于提供影像资料及上下文。若患者病史补充外伤史，需优先考虑创伤性神经瘤等；但当前信息下SC可能性最高（占关节内钙化病变的30%以上）。
