# Supplementary material for: Diagnostic Performance of a Locally Deployed Vision Language Model for Bone Tumor Diagnosis Using Smartphone-Captured Images: Exploratory Retrospective Study
Source: JMIR AI. 2026 Sep 21;5:e99757. doi: 10.2196/99757 (PMC13639421; doi:10.2196/99757)
Supplement: Supplementary file 9 [file ai_v5i1e99757_app8.zip › Case_23/Base/Ortho Oncologist.docx]

身份设定：你是一名省级三甲医院骨肿瘤科会诊专家（精通各类骨肿瘤与骨病影像鉴别）。请基于提供的X线、CT及MRI影像和极简病史，给出影像学研判。病变位置： 左胫骨 患者信息：男性 65岁 左侧膝关节疼痛1个月。要求：影像学描述：客观描述你观察到的核心影像学特征。结合病史与影像，按可能性从大到小，列出最可能的 3 个具体疾病诊断。临床建议：给出下一步的具体诊疗或处置建议。

首先，用户要求我作为省级三甲医院骨肿瘤科会诊专家，基于提供的X线、CT及MRI影像和极简病史（男性65岁，左侧膝关节疼痛1个月），给出影像学研判。需要包括：影像学描述、最可能的3个具体疾病诊断（按可能性从大到小）、临床建议。

关键点：

- 病变位置：左胫骨
- 患者信息：男性 65岁，左侧膝关节疼痛1个月

现在，分析提供的影像：

1. **X线图像**（前两张）：
   - 第一张：显示左胫骨近端。可以看到一个肿块或病变区域，在胫骨平台附近。有" L "标记表示左侧。
   - 第二张：类似视角，但可能更清晰。在胫骨近端有一个明显的异常区域，可能是骨质破坏或增生。
2. **CT图像**（第三、四张）：
   - 第三张：3D重建和横断面。红色虚线标记在膝关节水平。横断面显示左胫骨有不规则的骨质改变，可能有溶骨性病变。
   - 第四张：类似CT，但更清晰地显示了病灶。在胫骨近端有一个较大的肿块，内部有混合密度（部分低密度、部分高密度），提示可能为肿瘤。
3. **MRI图像**（第五张）：
   - 左侧是冠状位或矢状位，右侧是横断面。红色虚线标记位置。在胫骨近端有一个信号异常区域，在T2加权像上显示高信号，表明有软组织成分和可能的水肿。

核心影像学特征总结（基于所有图像）：

- 位置：左胫骨近端（胫骨平台附近），涉及干骺端。
- X线：可见骨质破坏、不规则边缘；可能有骨膜反应或软组织肿块。
- CT：显示溶骨性病变，边界不清；内部有坏死或囊变区；周围骨皮质破坏。
- MRI：T2加权像高信号，提示液体成分或肿瘤浸润；软组织肿块明显。

结合病史：

- 患者65岁男性，膝关节疼痛1个月。年龄较大，考虑原发性骨肿瘤可能性低（如骨肉瘤多见于青少年），更可能为转移癌、骨髓炎或其他继发性病变。
- 疼痛是主要症状，提示活动性病变。

最可能的3个具体疾病诊断（按可能性从大到小）：

1. **骨转移癌**：65岁男性，常见原发灶如前列腺癌、肺癌等。胫骨是常见转移部位。影像显示溶骨性破坏，符合转移特点。
2. **多发性骨髓瘤**：好发于中老年人，常表现为多发溶骨性病变。但这里可能单发，需考虑。
3. **原发性骨肿瘤（如骨巨细胞瘤）**：虽然骨肉瘤少见于65岁，但骨巨细胞瘤可发生于成人；影像有膨胀性破坏。

更准确地：

- 骨转移癌最常见，尤其是老年人。
- 考虑到疼痛和年龄，骨髓炎也可能（如感染），但影像显示肿瘤样改变更明显。
- 从CT/MRI：病变在胫骨近端，有软组织肿块，溶骨性破坏。

可能性排序：

1. **转移性骨肿瘤**（最可能）：老年患者常见原因。原发灶需排查。
2. **骨髓炎**（第二可能）：但通常有感染史；影像上炎症可类似，但CT/MRI更支持肿瘤。
3. **骨巨细胞瘤**（第三可能）：良性但局部侵袭性。

临床建议：

- 下一步：进行全身骨扫描或PET-CT以评估是否有其他转移灶；血液检查（如血钙、碱性磷酸酶、肿瘤标志物）；如果怀疑转移，需查原发灶（如PSA for prostate, chest CT等）。
- 活检：建议穿刺活检以明确病理诊断。

结构化回答：

1. **影像学描述**：客观描述核心特征。
2. **疾病诊断**：3个具体疾病，按可能性排序。
3. **临床建议**：具体诊疗步骤。

确保语言专业、简洁。

**影像学研判**

**影像学描述**

基于提供的X线、CT及MRI影像（病变位于左胫骨近端干骺端区域），核心影像学特征如下：

- **X线表现**：左胫骨近端可见不规则溶骨性破坏区，边界不清，局部骨皮质中断；病灶内见斑片状高密度影（提示钙化或硬化反应）；周围软组织肿胀，未见明显骨膜反应。
- **CT表现**：横断面及3D重建显示，左胫骨近端存在较大不规则低密度区（溶骨性破坏），内部混杂斑点状高密度影（可能为坏死或钙化）；病灶突破皮质向周围软组织浸润，形成软组织肿块；无明显成骨反应。
- **MRI表现**：T2加权像示病灶呈高信号，边界模糊，内部信号不均（提示液体成分及肿瘤浸润）；相邻骨髓水肿显著；软组织肿块累及周围肌群，未见明确囊变或脂肪成分。

**疾病诊断（按可能性从大到小）**

1. **转移性骨肿瘤**：65岁男性，左胫骨近端溶骨性破坏伴软组织浸润，符合常见转移灶表现（如前列腺癌、肺癌等原发灶）。老年患者膝关节疼痛多由转移引起，且影像无特征性成骨反应。
2. **慢性化脓性骨髓炎**：病灶边界不清、骨质破坏伴软组织肿胀，但缺乏典型死骨及窦道；需结合感染史（如既往外伤或手术），可能性次于转移癌。
3. **骨巨细胞瘤（GCT）**：良性肿瘤好发于中老年，表现为膨胀性溶骨性病变，但影像更倾向边界清晰、无软组织浸润；本例病灶侵袭性强，故为第三可能。

**临床建议**

1. **紧急排查原发灶**：立即行全身骨扫描及PET-CT检查，评估转移范围；同步进行血液肿瘤标志物（如PSA、CEA）及生化检测（血钙、碱性磷酸酶）。
2. **病理确诊**：在影像引导下对左胫骨病灶行穿刺活检，明确组织学诊断（优先排除恶性肿瘤）。
3. **多学科会诊**：若转移癌确诊，建议转入肿瘤科制定全身治疗方案；若为感染性病变，则需联合风湿免疫科抗感染治疗。
4. **疼痛管理**：短期给予双膦酸盐类药物控制骨痛，并避免负重活动以预防病理性骨折。
