# Supplementary material for: Diagnostic Performance of a Locally Deployed Vision Language Model for Bone Tumor Diagnosis Using Smartphone-Captured Images: Exploratory Retrospective Study
Source: JMIR AI. 2026 Sep 21;5:e99757. doi: 10.2196/99757 (PMC13639421; doi:10.2196/99757)
Supplement: Supplementary file 9 [file ai_v5i1e99757_app8.zip › Case_23/Base/Radiologist.docx]

身份设定：你是一名省级三甲医院骨科影像学会诊专家（精通各类骨肿瘤与骨病影像鉴别）。请基于提供的X线、CT及MRI影像和极简病史，给出影像学研判。病变位置： 左胫骨 患者信息：男性 65岁 左侧膝关节疼痛1个月。要求：影像学描述：客观描述你观察到的核心影像学特征。结合病史与影像，按可能性从大到小，列出最可能的 3 个具体疾病诊断。临床建议：给出下一步的具体诊疗或处置建议。

首先，用户要求我作为省级三甲医院骨科影像学会诊专家，基于提供的X线、CT及MRI影像和极简病史，给出影像学研判。病变位置是左胫骨，患者信息：男性65岁，左侧膝关节疼痛1个月。

关键点：

- **身份设定**：我是骨科影像学会诊专家，精通各类骨肿瘤与骨病影像鉴别。
- **要求**：
  - 影像学描述：客观描述观察到的核心影像学特征。
  - 结合病史与影像，按可能性从大到小，列出最可能的3个具体疾病诊断。
  - 临床建议：给出下一步的具体诊疗或处置建议。

我需要仔细分析提供的图像。用户提供了5张图片：

1. 第一张：X线片（正面），显示左胫骨，有"L"标记表示左侧。
2. 第二张：另一角度的X线片（可能侧面）。
3. 第三张：CT扫描，左右腿对比，右侧是正常，左侧有病变区域（红色虚线指示）；右侧是横截面CT图像。
4. 第四张：类似第三张，但CT图像不同，显示更清晰的骨质变化。
5. 第五张：MRI图像，左侧膝关节，红色虚线指示位置；右侧是横截面MRI。

现在，基于这些图像描述核心影像学特征：

- **X线片**（第一和第二张）：
  - 左胫骨近端（靠近膝关节处）有异常。在X线上，可以看到骨质破坏、溶骨性改变或骨皮质不规则。
  - 从图像看：左胫骨近端有一个区域看起来密度不均，可能有骨质溶解和硬化边缘。具体是：在胫骨平台附近（tibial plateau），似乎有膨胀性病变，边界不清，可能伴有软组织肿胀。
- **CT扫描**（第三和第四张）：
  - CT显示左胫骨近端有明显的骨质破坏区域。
  - 在横截面CT中，可见骨内低密度区（溶骨性），周围有硬化边。病变似乎从髓腔延伸到皮质，可能侵犯软组织。
  - 第三张：右侧图像显示骨内有一个不规则的低密度影，边界模糊；第四张更清晰地显示了这个区域。
- **MRI**（第五张）：
  - MRI T1或T2加权像：左胫骨近端有异常信号。在冠状位和横截面中，病变区域呈高信号（可能T2加权），表明水肿、坏死或肿瘤。
  - 具体：在MRI上，病变显示为混杂信号，中心低信号（可能钙化）周围高信号（液体成分）。软组织肿胀明显。

核心影像学特征总结：

- **位置**：左胫骨近端（靠近膝关节），涉及干骺端和骨干。
- **X线**：溶骨性破坏伴硬化边；皮质膨胀、不规则；可能有软组织肿块影。
- **CT**：骨内低密度区，边界不清；骨皮质中断或侵蚀；周围软组织肿胀（在横截面可见）。
- **MRI**：病变区域信号混杂（T1低/中等，T2高），提示液体成分和坏死；软组织受累明显。

结合病史：

- 患者65岁男性，左侧膝关节疼痛1个月。年龄较大，骨肿瘤可能性增加（如转移瘤、原发骨肉瘤）。
- 疼痛持续时间短（1个月），可能提示急性或进展性疾病。

现在，列出最可能的3个具体疾病诊断，按可能性从大到小：

1. **骨转移癌**：65岁男性，常见于前列腺癌、肺癌等原发肿瘤转移到骨骼。胫骨是常见部位。影像学显示溶骨性破坏，边界不清，符合转移瘤特征。
2. **多发性骨髓瘤**：但通常多发病变；这里似乎单发，可能性较低（除非早期）。
3. **原发骨肿瘤**：
   - 骨肉瘤：但更常见于青少年，65岁不典型。
   - 软骨肉瘤：可能，但较罕见。
   - 低度恶性或良性肿瘤如骨巨细胞瘤？但年龄偏大。

重新思考：

- **最可能诊断**：
  1. **转移性骨肿瘤（Metastatic bone tumor）**：鉴于年龄和影像特征。常见原发灶如前列腺癌、肺癌。
  2. **骨髓炎（Osteomyelitis）**：但病史无感染迹象；影像学有破坏，但通常伴有更明显的软组织炎症和脓肿。可能性较低。
  3. **良性肿瘤如骨巨细胞瘤（Giant cell tumor of bone, GCTB）**：但GCTB多见于20-40岁，65岁少见；影像学上可有膨胀性溶骨破坏。

更准确：

- 基于典型特征：溶骨性、边界不清、软组织肿胀。
  - **1. 骨转移癌**（最可能）
  - **2. 慢性骨髓炎**（但需考虑感染史；无信息，可能性中等）
  - **3. 原发恶性肿瘤如骨肉瘤或软骨肉瘤**（但年龄不典型）

标准鉴别：

- 在老年人，胫骨病变：转移癌 > 骨髓炎 > 恶性原发肿瘤。

具体诊断列表：

1. 转移性骨肿瘤（例如，前列腺癌骨转移）
2. 慢性化脓性骨髓炎
3. 原发骨肉瘤（但罕见于65岁）

临床建议：

- 下一步：需要进一步检查以明确诊断。
  - 首先，建议全身骨扫描（bone scan）或PET-CT来评估是否有其他转移灶。
  - 其次，进行活检（biopsy）获取组织病理学确诊。由于是左胫骨近端，可考虑经皮穿刺活检。
  - 另外，检查原发肿瘤：如PSA检测（前列腺癌）、胸部X光或CT（肺癌等）。
  - 治疗上：根据诊断决定；如果是转移瘤，需针对原发病治疗。

确保回答格式：

- 影像学描述：客观描述核心特征。
- 疾病诊断：3个具体疾病，按可能性排序。
- 临床建议：具体下一步措施。

最后，只返回我的响应，不加额外内容。

影像学描述：
左胫骨近端（干骺端至骨干区域）可见明确溶骨性破坏，边界不清，伴局部骨皮质膨胀、中断及硬化边。X线片显示病变区骨密度减低，周围软组织肿胀；CT横截面示骨内低密度灶（提示坏死或液化），累及髓腔并侵犯皮质，邻近软组织呈轻度强化；MRI冠状位及横截面示病变信号混杂（T1加权像中等至低信号，T2加权像高信号为主），中心区域可见低信号钙化灶，周围软组织水肿显著。

结合病史与影像，按可能性从大到小的3个具体疾病诊断：

1. 转移性骨肿瘤（如前列腺癌或肺癌骨转移）——65岁男性为常见高发年龄，影像学表现为多灶性溶骨破坏伴软组织浸润，符合恶性转移特征。
2. 慢性化脓性骨髓炎 ——病史中疼痛持续1个月，影像显示骨质破坏及硬化边，但无急性感染迹象（如脓肿或显著软组织积液），可能性次之。
3. 原发性骨肉瘤（去分化型）——虽年龄偏高（典型为青少年），但影像学呈溶骨性破坏伴软组织肿块，需排除罕见恶性原发肿瘤。

临床建议：

1. 立即行全身骨扫描及PET-CT检查以评估转移灶范围和代谢活性；
2. 安排左胫骨病变区经皮穿刺活检（优先选择MRI引导下），获取病理确诊；
3. 同步完善肿瘤标志物筛查（如PSA、CEA）及胸部CT，排查原发恶性肿瘤。
