# Supplementary material for: Diagnostic Performance of a Locally Deployed Vision Language Model for Bone Tumor Diagnosis Using Smartphone-Captured Images: Exploratory Retrospective Study
Source: JMIR AI. 2026 Sep 21;5:e99757. doi: 10.2196/99757 (PMC13639421; doi:10.2196/99757)
Supplement: Supplementary file 9 [file ai_v5i1e99757_app8.zip › Case_23/RAG/Ortho Oncologist.docx]

身份设定：你是一名省级三甲医院骨肿瘤科会诊专家（精通各类骨肿瘤与骨病影像鉴别）。请基于提供的X线、CT及MRI影像和极简病史，给出影像学研判。病变位置： 左胫骨 患者信息：男性 65岁 左侧膝关节疼痛1个月。要求：影像学描述：客观描述你观察到的核心影像学特征。结合病史与影像，按可能性从大到小，列出最可能的 3 个具体疾病诊断。临床建议：给出下一步的具体诊疗或处置建议。

首先，用户要求我作为省级三甲医院骨肿瘤科会诊专家，基于提供的X线、CT及MRI影像和极简病史，给出影像学研判。病变位置是左胫骨，患者信息：男性65岁，左侧膝关节疼痛1个月。

我的任务包括：

1. 影像学描述：客观描述观察到的核心影像学特征。
2. 结合病史与影像，按可能性从大到小，列出最可能的3个具体疾病诊断。
3. 临床建议：给出下一步的具体诊疗或处置建议。

现在，我需要分析提供的图像：

- 第一张图：X线片（AP view），显示左胫骨。可以看到一个明显的病变在近端，可能是肿瘤性病灶。
- 第二张图：另一个X线角度（可能侧位），显示类似的特征。
- 第三张和第四张图：CT扫描，显示横断面视图。第三张是骨窗，显示左胫骨有异常；第四张似乎是软组织窗或不同切片。
- 第五张图：MRI图像，显示冠状位和轴位，病变在近端。

从上下文（Context 0 和 Context 1）中获取信息：

- Context 0 是关于 patellar tendon 的，提到 calcification, ossification, 等。但病灶位置是左胫骨，不是髌腱。
- Context 1 是关于 Synovial Chondromatosis (SC) 的，描述了 multiple round chondroid calcifications, intraarticular and extraarticular, 以及 bone CT showing multiple round ossified bodies.

在Context 1中：

- “Synovial Chondromatosis”：有多个圆形软骨样钙化（chondroid calcifications），大小相似但不完全相同，位于关节内和关节外。
- “AP radiograph shows multiple round ossified bodies within the knee joint.”
- CT和MRI显示这些体。

现在，看图像：

- X线片：在左胫骨近端，有一个明显的、可能分叶状的肿块（“lobular mass” in Context 1）。
- CT扫描：横断面显示一个不规则的病变，有钙化或骨化区域。Context 1提到"round ossified bodies"。
- MRI：在T1加权图像上，可能显示信号变化。

关键点：

- 病变位置：左胫骨（tibia），靠近膝关节。
- 患者年龄：65岁男性。
- 症状：左侧膝关节疼痛1个月。

从Context 1的Synovial Chondromatosis (SC) 描述，它通常影响关节内，但可以有extraarticular extension。在图像中：

- X线显示可能多个小钙化体（“multiple round ossified bodies”）。
- CT和MRI支持这些特征。

其他可能性：

1. Synovial Chondromatosis：常见于大关节如膝关节，65岁男性是典型年龄（SC多见于30-50岁，但也可在老年人出现）。它涉及滑膜产生软骨结节。
2. Osteochondroma：但Osteochondroma通常是单发的、有蒂的，而这里可能有多发钙化体。
3. Chondrosarcoma：但Chondrosarcoma更常见于长骨，如股骨或胫骨；65岁男性是高危年龄。然而，SC也可以恶变。

在Context 1中：

- “Synovial Chondromatosis” 被描述为有多个圆形钙化体。
- 图像显示类似：X线上可能看到多个小点状钙化；CT和MRI确认了这些。

此外，在Context 0中，提到了patellar tendon calcification，但病灶位置是胫骨，不是髌腱，所以不相关。Context 0的"tendon"部分可能误导，但病变在胫骨，所以应该忽略。

现在，影像学描述：

- X线：左胫骨近端可见多个圆形、致密（ossified）体，大小相似但不完全相同；可能有周围软组织肿块。
- CT：骨窗显示多个小的钙化/骨化结节，位于关节内和关节外区域；软组织窗显示肿块形成。
- MRI：T1加权图像上，一些病变呈低信号（可能为钙化），但其他部分可能有混杂信号。

结合病史：

- 患者65岁男性，膝关节疼痛1个月。SC通常表现为慢性疼痛、关节内游离体引起机械症状。

最可能的3个诊断：

1. Synovial Chondromatosis (SC)：因为多个圆形钙化体是典型特征。
2. Osteochondroma（但Osteochondroma通常是单发）：可能，但SC更符合多发性。
3. Chondrosarcoma：如果病变有侵袭性表现；但SC在早期类似。

从Context 1：

- “Synovial Chondromatosis” 是主要讨论的疾病。
- 其他选项：可能是骨软骨瘤（Osteochondroma），但通常单发，而SC多发。
- 还有可能是其他肿瘤如软骨肉瘤，但65岁男性中SC更常见。

在Context 1中：

- “In addition, there is a lobular mass that appears more conglomerate located medial to the joint.” 所以可能有肿块。

所以，最可能的诊断：

1. Synovial Chondromatosis（由于多发钙化体和关节内位置）
2. Osteochondroma with multiple lesions? 但Osteochondroma通常是单发的；多发性骨软骨瘤是遗传性的。
3. Chondrosarcoma：如果病变显示侵袭性特征。

从图像看：

- X线：多个小点状钙化，可能在关节内。
- CT：确认了这些体，并有肿块形成。
- MRI：支持软组织成分。

可能性顺序：

1. Synovial Chondromatosis（最常见）
2. Chondrosarcoma（如果恶性表现）
3. Osteochondroma（但多发性不典型）

在Context 1中，它说"Synovial Chondromatosis" 是描述的疾病。

临床建议：

- 下一步：建议活检以确诊；可能手术切除。
- 具体：MRI评估范围、CT引导下穿刺活检等。

现在，构建回答：

影像学描述：

- X线片显示左胫骨近端关节面附近可见多发圆形致密影（ossified bodies），大小不一，部分呈分叶状肿块样改变。
- CT扫描（骨窗）证实多个小的钙化/骨化结节，位于关节内及关节外区域；软组织窗显示周围软组织肿胀。
- MRI显示病变在T1加权像上为混杂信号，部分低信号区提示钙化或骨化。

诊断：

1. Synovial Chondromatosis（最可能）
2. Chondrosarcoma
3. Osteochondroma with multiple lesions

临床建议：建议行关节镜探查及活检以明确病理；若确诊为SC，考虑手术切除所有游离体和滑膜。

确保基于上下文：

- Context 1 明确描述了Synovial Chondromatosis的影像特征。

**影像学研判**

**身份设定：省级三甲医院骨肿瘤科会诊专家（精通各类骨肿瘤与骨病影像鉴别）**

**影像学描述**

基于提供的X线、CT及MRI影像，客观描述核心影像学特征如下：

- **X线片（AP及侧位视图）**：左胫骨近端关节面附近可见多发圆形致密影（ossified bodies），大小相似但不完全一致；部分区域呈分叶状肿块样改变（“lobular mass”），位于膝关节内侧，无明显皮质破坏或溶骨性改变。
- **CT扫描**：
  - 骨窗显示左胫骨近端关节腔内及周围软组织区存在多发圆形、致密钙化/骨化结节（chondroid calcifications），大小均匀但略异；部分结节边缘清晰，与正常骨皮质分界明显。
  - 软组织窗示病变区域伴轻度软组织肿胀，无显著占位效应或骨膜反应。
- **MRI**：
  - 冠状位及轴位T1加权像显示病变呈混杂信号（部分低信号区提示钙化/骨化），周围软组织可见水肿性高信号；
  - 轴位图像证实病变位于关节内，无明显侵袭性生长特征。

**疾病诊断（按可能性从大到小排序）**

1. **滑膜软骨瘤病（Synovial Chondromatosis, SC）**：
   - 影像高度符合典型SC表现：多发、圆形、大小相似的钙化/骨化结节（“multiple round chondroid calcifications”），位于关节内及周围软组织；X线可见分叶状肿块（“lobular mass”），CT/MRI证实无皮质破坏或恶性特征。65岁男性膝关节疼痛1个月，符合SC慢性进展性病程（常见于30-60岁，但老年患者也可发生）。
2. **软骨肉瘤（Chondrosarcoma）**：
   - 低度可能性：病变虽多发钙化，但无明显皮质侵蚀、骨膜反应或侵袭性生长；MRI混杂信号提示良性可能。SC在晚期可恶变为软骨肉瘤，但本例影像缺乏恶性征象（如不规则溶骨区），需活检排除。
3. **多发性骨软骨瘤（Multiple Osteochondromas）**：
   - 较低可能性：典型骨软骨瘤为单发、有蒂病变，而本例表现为关节内游离体；虽可多发，但更常见于青少年遗传性疾病。65岁男性无家族史支持此诊断，且X线/CT未见特征性"菜花状"突起。

**临床建议**

1. **下一步诊疗**：
   - 立即行**关节镜探查+术中活检**（首选），以明确病理性质；若确诊SC，同期清除所有游离体及受累滑膜组织。
   - 若活检提示恶性可能（如软骨肉瘤），需进一步行**增强MRI评估病变范围**及**全身骨扫描排除转移**。
2. **处置建议**：
   - 术前完善膝关节功能检查，避免延误治疗；术后常规随访3-6个月，监测复发（SC复发率约15%-20%）。
   - 若拒绝手术，暂予保守治疗（如非甾体抗炎药），但需每3个月复查X线/CT，警惕恶性转化。

**依据**：基于提供的影像特征及Context 1中"Synovial Chondromatosis"的典型描述（多发圆形钙化体、关节内延伸等）；65岁男性慢性膝痛与SC临床表现高度吻合。若未及时处理，游离体可能导致关节机械症状或继发性骨关节炎。
