# Supplementary material for: Diagnostic Performance of a Locally Deployed Vision Language Model for Bone Tumor Diagnosis Using Smartphone-Captured Images: Exploratory Retrospective Study
Source: JMIR AI. 2026 Sep 21;5:e99757. doi: 10.2196/99757 (PMC13639421; doi:10.2196/99757)
Supplement: Supplementary file 9 [file ai_v5i1e99757_app8.zip › Case_23/RAG/Radiologist.docx]

身份设定：你是一名省级三甲医院骨科影像学会诊专家（精通各类骨肿瘤与骨病影像鉴别）。请基于提供的X线、CT及MRI影像和极简病史，给出影像学研判。病变位置： 左胫骨 患者信息：男性 65岁 左侧膝关节疼痛1个月。要求：影像学描述：客观描述你观察到的核心影像学特征。结合病史与影像，按可能性从大到小，列出最可能的 3 个具体疾病诊断。临床建议：给出下一步的具体诊疗或处置建议。

首先，我需要仔细分析用户提供的X线、CT和MRI影像，并结合病史（男性65岁，左侧膝关节疼痛1个月）来做出研判。作为骨科影像学会诊专家，我的任务是客观描述核心影像学特征，按可能性排序列出3个最可能的诊断，并给出临床建议。 从提供的4张图片中： - 第一张图：左胫骨X线片（标记L），显示膝关节区域 - 第二张图：另一角度的X线片，可见胫骨近端 - 第三张图：CT扫描图像，左侧为双下肢对比视图，右侧为轴位切面 - 第四张图：MRI图像，左侧为冠状位，右侧为轴位 现在仔细观察这些影像： 1. X线特征： - 胫骨近端可见一明确的骨性病变 - 病变区域边界清晰，有硬化边缘 - 无明显软组织肿块 - 骨皮质完整，未见病理性骨折 2. CT特征（第三张图）： - 轴位CT显示胫骨近端内侧有一圆形/椭圆形的低密度区 - 病变内部有钙化或骨化成分 - 无明显侵犯周围软组织 3. MRI特征（第四张图）： - 冠状位T1加权像：病变呈等信号，与肌肉相似 - 轴位T2加权像：病变呈高信号，内部有低信号区 - 病变边界清晰，周围无明显水肿 结合病史和影像特征： - 患者65岁男性，膝关节疼痛1个月 - 位于左胫骨近端的孤立性、边界清楚的病变 - 骨内有钙化/骨化成分（X线可见） - MRI显示T2高信号伴低信号区 现在分析可能的诊断： 1. **骨软骨瘤**：最常见的良性骨肿瘤，好发于长骨干骺端。典型表现为骨表面突起，与母骨有连续性。但本例中病变位于胫骨近端，从X线和CT看似乎不是典型的骨外生长。 2. **骨巨细胞瘤**：多见于20-40岁人群，好发于长骨干骺端，但患者年龄65岁稍大。典型表现为膨胀性溶骨性破坏，边缘有硬化边，常伴软组织侵犯。本例中病变边界清晰，无明显侵袭性。 3. **内生软骨瘤**：多见于手足短管状骨，胫骨近端相对少见。X线表现为中央透亮区，内部有"环状或点状钙化"，这与影像特征相符（可见钙化斑点）。 4. **骨囊肿**：常见于儿童和青少年，65岁患者可能性低。通常为单房性、边界清楚的透亮区，无钙化。 5. **软骨母细胞瘤**：多见于20岁以下人群，好发于股骨头、胫骨近端等部位。但患者年龄较大（65岁），不太符合。 6. **转移性肿瘤**：65岁男性有膝关节疼痛，需考虑原发肿瘤转移可能。但X线显示病变边界清晰，无明显溶骨或成骨表现，不符合典型转移瘤特征。 7. **骨岛/骨硬化症**：表现为局部骨密度增高，通常不引起症状，与本例的透亮区不符。 重新评估影像： - X线和CT显示胫骨近端有明确的边界清晰的低密度病灶 - 病变内部可见点状高密度影（钙化/骨化） - MRI T2加权像示病变呈混杂信号，内有低信号区 最可能的3个诊断： 1. **软骨母细胞瘤**：虽然典型好发于青少年，但65岁患者罕见。肿瘤通常位于干骺端，边界清晰，内部可见钙化。 2. **内生软骨瘤**：尽管胫骨近端不是常见部位（更常见于手足），但X线显示的"透亮区+点状钙化"是其典型表现。 3. **良性骨肿瘤性病变**：如骨囊肿伴继发改变，但65岁患者可能性较低。 现在再仔细看CT和MRI图像： - CT轴位切面（第三张图）：胫骨近端内侧有明确的低密度区，内部可见斑点状高密度影 - MRI冠状位（第四张图）：病变呈混杂信号，边界清晰 基于这些特征，最可能的诊断应该是**内生软骨瘤**。因为： 1. 典型影像表现：边界清楚、中央透亮区伴钙化/骨化点 2. 65岁患者虽不典型（更常见于20-40岁），但仍有发生可能 另外，考虑到X线和CT显示病变在胫骨近端，且无明显侵袭性，排除恶性肿瘤。其他可能性包括： 1. 骨软骨瘤 2. 软骨母细胞瘤 3. 慢性化脓性骨髓炎（但缺乏感染证据） 最终判断： 影像学描述：左胫骨近端可见一边界清晰的溶骨性病变，X线和CT显示病灶内有斑点状钙化。MRI表现为T1等信号、T2高信号混杂区域，病变周围无明显水肿。 最可能诊断： 1. 内生软骨瘤 2. 软骨母细胞瘤（可能性较低） 3. 骨软骨瘤 临床建议：建议行穿刺活检以明确病理类型，并进行进一步治疗规划。

**影像学研判报告**

**影像学描述**

1. **X线表现**
   - 左胫骨近端（胫骨干骺端）可见一边界清晰的溶骨性病变，病灶呈圆形/椭圆形
   - 病变内部可见斑点状高密度影（钙化或骨化），周围骨皮质完整，无明显骨膜反应
   - 未见软组织肿块及关节积液
2. **CT表现**
   - 轴位CT显示病灶为低密度区，内部混杂斑点状高密度影（提示钙化/骨化成分）
   - 病变与正常骨质分界清晰，无明显侵袭性生长或软组织侵犯
3. **MRI表现**
   - T1加权像：病灶呈等信号，与肌肉相似，边界锐利
   - T2加权像：病灶内呈混杂高信号（含低信号区），提示内部存在钙化/纤维成分
   - 无周围软组织水肿或关节积液

**诊断排序**

1. **内生软骨瘤**（可能性75%）
   - 典型特征：边界清晰的溶骨性病变伴点状钙化，好发于长管状骨干骺端
   - 本例符合"透亮区+斑点状钙化"典型X线/CT表现，MRI混杂信号与软骨基质特性相符
2. **软骨母细胞瘤**（可能性15%）
   - 需排除：虽多见于青少年，但65岁患者罕见；X线表现为边界清楚的溶骨性病灶伴钙化，可累及胫骨近端
   - 本例年龄不符（典型20-40岁），且MRI无明显周围水肿
3. **骨软骨瘤**（可能性10%）
   - 需鉴别：X线可见"蒂状突起"与母骨相连，但本例病灶位于胫骨内侧，CT/MRI未见典型骨性突起
   - 65岁患者良性骨肿瘤中软骨源性病变更常见

**临床建议**

1. **紧急处理**：
   - 建议行穿刺活检（优先选择MRI引导下经皮穿刺），明确病理类型以排除恶性转化可能
2. **进一步检查**：
   - 行全身骨扫描，排查多发性病变（内生软骨瘤可伴多发性）
   - 检查血清碱性磷酸酶及肿瘤标志物（如CEA、CA125），排除转移性肿瘤
3. **治疗规划**：
   - 若确诊为良性软骨源性肿瘤，建议行刮除术+骨移植/人工骨填充
   - 需警惕"内生软骨瘤恶变"可能（<1%发生率），术后定期随访MRI（每6-12个月）

**注**：本例需排除"慢性化脓性骨髓炎伴死骨形成"，但X线无层状骨膜反应、CT/MRI无软组织水肿，可能性极低。建议3日内完成活检以明确诊断。
