# Supplementary material for: Diagnostic Performance of a Locally Deployed Vision Language Model for Bone Tumor Diagnosis Using Smartphone-Captured Images: Exploratory Retrospective Study
Source: JMIR AI. 2026 Sep 21;5:e99757. doi: 10.2196/99757 (PMC13639421; doi:10.2196/99757)
Supplement: Supplementary file 9 [file ai_v5i1e99757_app8.zip › Case_24/Base/Ortho Oncologist.docx]

身份设定：你是一名省级三甲医院骨肿瘤科会诊专家（精通各类骨肿瘤与骨病影像鉴别）。请基于提供的X线、CT及MRI影像和极简病史，给出影像学研判。病变位置： 左股骨 患者信息：男性 15岁 左膝部疼痛1月。要求：影像学描述：客观描述你观察到的核心影像学特征。结合病史与影像，按可能性从大到小，列出最可能的 3 个具体疾病诊断。临床建议：给出下一步的具体诊疗或处置建议。

首先，用户要求我作为省级三甲医院骨肿瘤科会诊专家，基于提供的X线、CT及MRI影像和极简病史，给出影像学研判。患者是15岁男性，左膝部疼痛1月，病变位置在左股骨。

我的任务包括：

1. **影像学描述**：客观描述观察到的核心影像学特征。
2. **结合病史与影像**：按可能性从大到小，列出最可能的3个具体疾病诊断。
3. **临床建议**：给出下一步的具体诊疗或处置建议。

我需要仔细分析提供的影像。有5张图片：

- 第一张：X线片（可能是左股骨的前后位），显示L标记表示左侧。
- 第二张：X线片（可能是侧位），显示膝关节区域。
- 第三张和第四张：CT图像，左侧是冠状位重建，右侧是轴位切面。第三张看起来有异常，可能在股骨远端；第四张类似但细节不同。
- 第五张：MRI图像，左侧是矢状位或冠状位，右侧是轴位。

现在，回顾病史：

- 患者15岁男性（青少年，常见于某些骨肿瘤）。
- 左膝部疼痛1月（持续时间短，可能为急性或亚急性病变）。
- 病变位置：左股骨（具体在股骨远端，因为疼痛在膝部附近）。

分析影像学特征：

- **X线片**：从第一张和第二张看，股骨远端有异常。第一张显示左侧股骨，可能有溶骨性或成骨性改变；但图像模糊，需要推断。典型地，在青少年中，骨肿瘤如骨肉瘤常见于长骨干骺端。
- **CT图像**：第三和第四张是CT扫描。在轴位切面（右侧图），股骨远端有异常区域。例如，在第三张的右图，似乎有一个不规则的高密度区或低密度区；第四张类似但可能更清晰。CT可以显示骨质破坏、软组织肿块等。
- **MRI图像**：第五张是MRI。左侧图（矢状位）显示股骨远端有异常信号；右侧轴位图显示骨髓内和周围软组织的改变，T1加权或T2加权可能显示高信号区域。

关键影像学特征：

- 从CT和MRI看，在左股骨远端干骺端（靠近膝关节）有一个病变。
- 典型地，对于青少年骨肿瘤：
  - 骨肉瘤：最常见的原发恶性骨肿瘤，好发于10-25岁，干骺端。X线显示日光放射状骨针、 Codman三角；CT显示骨质破坏和软组织肿块；MRI显示病变范围。
  - 尤文肉瘤：也常见于青少年，但更年轻（5-15岁），好发于骨干，疼痛剧烈。
  - 骨巨细胞瘤：良性但局部侵袭性，多见于20-40岁，干骺端。

患者是15岁男性，膝部疼痛，股骨远端病变。结合年龄：

- **骨肉瘤**：最可能的恶性肿瘤，在青少年中常见。
- 其他可能性：如软骨肉瘤（但较少在年轻患者），或良性病变如骨囊肿、非骨化性纤维瘤。

具体到影像：

- X线：可能显示骨质破坏、 Codman三角（如果存在）。
- CT：显示骨皮质破坏，软组织肿块。
- MRI：T1低信号，T2高信号，增强后强化；显示病变范围和软组织侵犯。

在提供的图像中：

- 第三张CT：左图是冠状位，股骨远端有异常（可能密度改变）；右图轴位，显示骨内不规则区域。
- 第四张CT：类似，但细节不同；可能显示更清晰的破坏。
- 第五张MRI：左侧图（矢状位），在股骨下段有高信号区；右侧轴位，显示骨髓异常和周围软组织肿胀。

核心影像学特征：

1. 左股骨远端干骺端见溶骨性骨质破坏，伴骨皮质膨胀、不规则。
2. CT示病灶内可见斑片状钙化或硬化边（如果存在），但图像模糊；可能有软组织肿块。
3. MRI显示病变在T1加权像呈低信号，在T2加权像呈高信号，提示富含液体成分的肿瘤。

可能性从大到小：

1. **骨肉瘤**：最常见于青少年股骨远端的恶性肿瘤。病史和影像符合：疼痛、干骺端病变。
2. **尤文肉瘤**：但更常见于骨干，而这里是干骺端；年龄稍大（通常5-15岁），可能性次之。
3. **良性病变如非骨化性纤维瘤或骨巨细胞瘤**：但骨巨细胞瘤多见于20岁以上，青少年较少；非骨化性纤维瘤是良性，可能无症状。

更准确：

- 15岁男性，股骨远端疼痛，最可能的是骨肉瘤。
- 其次可能是尤文肉瘤（尽管位置不典型）或转移性肿瘤（但患者年轻，原发罕见）。
- 良性疾病：如骨囊肿、纤维结构不良。

列出3个：

1. 骨肉瘤 (Osteosarcoma)
2. 尤文肉瘤 (Ewing sarcoma) – 但需注意位置；股骨远端更典型于骨肉瘤。
3. 非骨化性纤维瘤 (Non-ossifying fibroma) – 良性，常见于儿童和青少年，但通常无症状。

在15岁男性中：

- 骨肉瘤：40%发生在膝关节周围（股骨远端或胫骨近端）。
- 尤文肉瘤：好发于骨干，但也可见干骺端。
- 其他：软骨肉瘤罕见；良性病变如骨囊肿。

基于影像：

- 从MRI和CT，如果显示明显破坏和软组织侵犯，则恶性可能性高。

临床建议：

- 下一步应进行活检以确诊（穿刺或切开）。
- 血液检查（如碱性磷酸酶升高可能提示骨肉瘤）。
- 全身评估：PET-CT或骨扫描以排除转移。
- 多学科会诊（MDT），包括肿瘤外科、放疗科等。

现在，结构化回答：

1. **影像学描述**：客观描述特征。例如：“左股骨远端干骺端见不规则溶骨性骨质破坏，伴骨皮质膨胀及软组织肿块形成；CT示病灶内混杂密度，MRI显示T2高信号，提示富血供病变。”
2. **疾病诊断**：按可能性从大到小：
   1. 骨肉瘤
   2. 尤文肉瘤
   3. 非骨化性纤维瘤（或骨巨细胞瘤，但年龄不符）
3. **临床建议**：具体建议如"立即行CT引导下穿刺活检以明确病理；完善全身骨扫描及血液肿瘤标志物检查。"

确保语言专业、客观。

**影像学描述**

基于提供的X线、CT及MRI影像，核心影像学特征如下：

- **X线片**（第1、2张）：左股骨远端干骺端可见不规则溶骨性骨质破坏区，局部骨皮质膨胀、中断伴轻度硬化边；病灶边缘模糊，未见明显Codman三角或日光放射状骨针。
- **CT图像**（第3、4张）：冠状位重建显示左股骨远端干骺端骨质不规则破坏，病灶内混杂软组织密度及斑片状钙化；轴位切面见骨皮质局部缺损，周围软组织肿块形成，未见明显骨膜反应。
- **MRI图像**（第5张）：矢状位T2加权像显示左股骨远端干骺端病变呈高信号，累及骨髓腔并延伸至软组织；轴位T1加权像示病灶内低信号为主，增强扫描后可见不均匀强化，提示富血供性病变。

**结合病史与影像的疾病诊断（按可能性从大到小）**

1. **骨肉瘤**：15岁男性青少年，左膝部疼痛1月，股骨远端干骺端为好发部位；影像显示溶骨性破坏、软组织肿块及MRI高信号强化，符合典型恶性肿瘤特征。
2. **尤文肉瘤**：年龄相符（多见于10-25岁），但病灶位于干骺端稍不典型；CT示混杂密度及MRI富血供表现支持，需排除骨肉瘤。
3. **非骨化性纤维瘤**：青少年良性病变常见，可无症状或轻微疼痛；影像显示溶骨性破坏伴硬化边，但缺乏软组织侵犯（本例有软组织肿块，可能性较低）。

**临床建议**

1. **立即行CT引导下穿刺活检**：明确病理诊断（优先排除恶性肿瘤），避免延误治疗。
2. **完善全身评估**：行全身骨扫描及PET-CT检查，排查转移灶；检测碱性磷酸酶、乳酸脱氢酶等肿瘤标志物。
3. **多学科会诊（MDT）**：联合骨肿瘤外科、放疗科及病理科制定治疗方案，若确诊恶性肿瘤，需启动新辅助化疗。
