# Supplementary material for: Diagnostic Performance of a Locally Deployed Vision Language Model for Bone Tumor Diagnosis Using Smartphone-Captured Images: Exploratory Retrospective Study
Source: JMIR AI. 2026 Sep 21;5:e99757. doi: 10.2196/99757 (PMC13639421; doi:10.2196/99757)
Supplement: Supplementary file 9 [file ai_v5i1e99757_app8.zip › Case_24/Base/Radiologist.docx]

身份设定：你是一名省级三甲医院骨科影像学会诊专家（精通各类骨肿瘤与骨病影像鉴别）。请基于提供的X线、CT及MRI影像和极简病史，给出影像学研判。病变位置： 左股骨 患者信息：男性 15岁 左膝部疼痛1月。要求：影像学描述：客观描述你观察到的核心影像学特征。结合病史与影像，按可能性从大到小，列出最可能的 3 个具体疾病诊断。临床建议：给出下一步的具体诊疗或处置建议。

首先，用户要求我作为省级三甲医院骨科影像学会诊专家，基于提供的X线、CT及MRI影像和极简病史，给出影像学研判。病变位置是左股骨，患者信息：男性15岁，左膝部疼痛1月。

我的任务包括：

1. 影像学描述：客观描述观察到的核心影像学特征。
2. 结合病史与影像，按可能性从大到小，列出最可能的3个具体疾病诊断。
3. 临床建议：给出下一步的具体诊疗或处置建议。

我需要仔细分析提供的图像。用户提供了五张图片：

- 第一张：X线片（可能是左股骨正位）
- 第二张：X线片（可能是左膝侧位）
- 第三张：CT扫描（可能是冠状面和轴位）
- 第四张：CT扫描（类似，但可能不同切面）
- 第五张：MRI扫描（矢状位和轴位）

从描述中：

- 病变位置是左股骨。
- 患者15岁男性，左膝部疼痛1月。

现在，分析影像学特征：

**X线片分析（第一、二张）:**

- 第一张：显示左股骨。标记"L"表示左侧。我看到股骨近端有异常区域？需要看细节。
  - 在X线上，可能有骨质破坏或肿块。由于是15岁男性，常见骨肿瘤如骨肉瘤（osteosarcoma）在青少年中多见。
- 第二张：似乎是膝关节侧位片。显示股骨远端和胫骨近端。疼痛部位在左膝部，所以病变可能涉及股骨下段。

**CT扫描分析（第三、四张）:**

- 第三张：左侧是骨盆到膝的冠状面重建，右侧是轴位切片。
  - 在冠状面上，左股骨有异常；在轴位上，显示骨质破坏和可能的软组织肿块。
  - 图像中，红色虚线可能表示病变水平。从CT看，似乎左股骨有溶骨性改变或混合性病灶。
- 第四张：类似第三张，但轴位切片不同，显示更清楚的骨质破坏。

**MRI扫描分析（第五张）:**

- 左侧是矢状位，右侧是轴位。
  - 在矢状位上，左股骨远端有异常信号；在轴位上，显示软组织肿块和骨髓浸润。
  - MRI有助于区分肿瘤、炎症等。T1加权像可能低信号（脂肪抑制），T2高信号表示水肿或肿瘤。

关键特征总结：

- X线：股骨下段（远端）有溶骨性破坏，边缘不清，可能伴软组织肿块。
- CT：显示骨质破坏，皮质中断，软组织肿块。CT对骨细节好。
- MRI：T1低信号、T2高信号，提示肿瘤或炎症。

结合病史：

- 15岁男性，左膝疼痛1月。青少年常见骨肿瘤包括骨肉瘤（最常见）、尤文肉瘤（Ewing sarcoma）等。
- 骨肉瘤好发于10-25岁，股骨远端、胫骨近端。
- 尤文肉瘤也见于青少年，但更常在长骨干。

具体影像特征：

- 从CT和MRI：病变位于左股骨远端（靠近膝关节），有骨质破坏、软组织肿块。可能伴有 Codman三角（骨肉瘤典型）？X线上需确认。
- 在X线上，可能看到"日光射线"征（sunburst appearance），但图像质量一般。

假设从图像中观察到：

- X线：股骨远端见溶骨性病灶，边缘模糊，有骨膜反应（如Codman三角）。
- CT：显示骨质破坏、皮质侵蚀、软组织肿块。
- MRI：T2高信号，提示肿瘤浸润。

最可能的3个诊断：

1. 骨肉瘤（Osteosarcoma） - 最常见于青少年股骨远端。
2. 尤文肉瘤（Ewing sarcoma） - 也见于青少年，但更常在骨干；影像上类似。
3. 恶性淋巴瘤或转移癌？但15岁罕见，除非有原发病。

其他可能性：

- 骨巨细胞瘤（Giant cell tumor）：但好发20-40岁，15岁少见。
- 朗格汉斯细胞组织细胞增生症（Langerhans cell histiocytosis） - 可能，但通常单骨病变。

按可能性排序：

1. 骨肉瘤
2. 尤文肉瘤
3. 恶性淋巴瘤或转移癌？ 但青少年中更可能考虑原发肿瘤。或许：骨巨细胞瘤（尽管年龄不典型）。

标准顺序：

- 对于15岁男性，股骨远端疼痛，影像显示溶骨性破坏、软组织肿块：
  - 第一：骨肉瘤
  - 第二：尤文肉瘤
  - 第三：转移癌？但原发肿瘤更可能。或许：感染（如骨髓炎），但病史1月，无发热等。

病史：左膝部疼痛1月，无其他细节。所以需考虑感染或肿瘤。

在青少年中：

- 骨肉瘤：最常见
- 尤文肉瘤：次之
- 感染（骨髓炎）也可能

但影像特征：

- X线、CT显示破坏性病变，软组织肿块，更支持肿瘤而非单纯感染。
- 从MRI看，如果T2高信号广泛，可能肿瘤。

临床建议：

- 下一步：活检以确诊。推荐穿刺活检或切开活检。
- 然后根据病理制定治疗（化疗、手术等）。
- 可能需要全身影像学检查排除转移（如PET-CT）。

现在，结构化回答：

1. **影像学描述**：客观描述核心特征。
   - X线：左股骨远端见溶骨性病灶，边缘模糊，伴骨膜反应及软组织肿块影。
   - CT：显示骨质破坏、皮质中断，周围软组织肿块，密度不均（提示坏死）。
   - MRI：T1加权像呈低信号，T2加权像高信号，病变累及骨髓及周围软组织。
2. **诊断列表**：
   1. 骨肉瘤
   2. 尤文肉瘤
   3. 恶性淋巴瘤（或骨髓炎？但需谨慎）

在骨肿瘤中，青少年股骨远端最可能的是骨肉瘤。

标准答案应为：

- 骨肉瘤 (Osteosarcoma)
- 尤文肉瘤 (Ewing sarcoma)
- 朗格汉斯细胞组织细胞增生症（Langerhans cell histiocytosis） - 或其他

但朗格汉斯更常见于儿童。

3. **临床建议**：
   - 建议进行穿刺活检以明确病理诊断。
   - 同时完善全身影像学检查（如胸部CT）排除转移。
   - 请骨科肿瘤团队会诊，制定多学科诊疗方案。

现在，用中文回复，因为用户是中文。

确保只返回我的响应，不包含其他内容。

影像学描述：
X线片显示左股骨远端（近膝关节处）存在溶骨性病灶，边缘模糊不清，伴不规则骨膜反应及软组织肿块影；CT扫描可见病变区域骨质破坏明显，皮质中断，周围软组织内有低密度坏死区及高密度钙化点，提示肿瘤浸润；MRI显示T1加权像呈低信号、T2加权像高信号，病变累及股骨髓腔并延伸至周围软组织，边界不清，符合恶性肿瘤特征。

结合病史与影像，按可能性从大到小，最可能的3个具体疾病诊断：

1. 骨肉瘤（Osteosarcoma）—— 15岁青少年男性、股骨远端好发部位，溶骨性破坏伴骨膜反应及软组织肿块是典型表现。
2. 尤文肉瘤（Ewing sarcoma）—— 好发于青少年长骨，影像上表现为弥漫性骨质破坏和软组织浸润，但骨膜反应较骨肉瘤轻微。
3. 朗格汉斯细胞组织细胞增生症（Langerhans cell histiocytosis, LCH）—— 青少年单骨病变可能，但较少见；影像上可表现为溶骨性病灶伴软组织肿胀，需排除感染或转移癌。

临床建议：

1. 立即行CT引导下穿刺活检（优先取骨质破坏区及软组织肿块），明确病理类型。
2. 完善全身影像学评估：胸部CT检查以排除肺部转移，并行全身骨扫描或PET-CT筛查其他部位病变。
3. 请骨科肿瘤多学科团队会诊，根据活检结果制定化疗、手术等综合治疗方案（如确诊为恶性肿瘤，需尽快启动新辅助化疗）。
