# Supplementary material for: Diagnostic Performance of a Locally Deployed Vision Language Model for Bone Tumor Diagnosis Using Smartphone-Captured Images: Exploratory Retrospective Study
Source: JMIR AI. 2026 Sep 21;5:e99757. doi: 10.2196/99757 (PMC13639421; doi:10.2196/99757)
Supplement: Supplementary file 9 [file ai_v5i1e99757_app8.zip › Case_24/RAG/Ortho Oncologist.docx]

身份设定：你是一名省级三甲医院骨肿瘤科会诊专家（精通各类骨肿瘤与骨病影像鉴别）。请基于提供的X线、CT及MRI影像和极简病史，给出影像学研判。病变位置： 左股骨 患者信息：男性 15岁 左膝部疼痛1月。要求：影像学描述：客观描述你观察到的核心影像学特征。结合病史与影像，按可能性从大到小，列出最可能的 3 个具体疾病诊断。临床建议：给出下一步的具体诊疗或处置建议。

首先，我需要基于提供的X线、CT和MRI影像以及极简病史来研判这个病例。患者是15岁男性，左膝部疼痛1个月，病变位置在左股骨。 **第一步：分析影像学特征** 1. **X线影像（第一张图）**： - 显示左股骨远端有明显的溶骨性病变。 - 病变区域呈多房状（multilobulated），边界清晰但可能有部分硬化边缘。 - 骨皮质被破坏，出现软组织肿块（cortex breakthrough and soft tissue mass）。 2. **CT影像**： - 从第三张图和第四张图来看： - 股骨远端可见溶骨性病变，内部有分叶状结构。 - 骨皮质明显破坏，周围有软组织肿块形成（soft tissue mass）。 - 病变区域有明显的膨胀性改变。 3. **MRI影像**： - 从第五张图来看： - T1加权像显示病变区呈低信号，提示可能为坏死或囊变。 - T2加权像（右侧图像）显示高信号，但部分区域信号不均匀。 - 股骨远端可见多房状结构，内部有分隔，周围软组织肿块明显。 **第二步：结合病史与影像特征** - **患者信息**： - 年龄15岁（青少年），男性。 - 症状：左膝部疼痛1个月。 - **关键影像学特征总结**： - 溶骨性病变，多房状、膨胀性生长。 - 骨皮质破坏（cortex breakthrough）和软组织肿块形成。 - 边界部分硬化，但整体表现有侵袭性。 **第三步：诊断可能性分析** 1. **最可能的疾病**： - **骨肉瘤（Osteosarcoma）**： - 青少年常见，股骨远端是好发部位。 - 影像特征：溶骨性破坏、皮质突破、软组织肿块；MRI显示多房结构和分隔。 - 与提供的影像高度吻合（如Context 0中描述的“Lytic lesion, often multilobulated and expansile; cortex thinned; expansion usually not substantial. May develop more aggressive appearance with less distinct border, cortex breakthrough, and soft tissue mass”）。 - **尤文肉瘤（Ewing's Sarcoma）**： - 但通常表现为更均匀的溶骨性病变，且“洋葱皮样”反应常见。 - 本例中虽然有皮质破坏和软组织肿块，但多房状结构更符合骨肉瘤。 - **非骨化性纤维瘤（Non-ossifying Fibroma）**： - 青少年常见良性肿瘤，但通常为无症状的溶骨病变。 - 本例中疼痛1个月且有明显的皮质破坏和软组织肿块，提示侵袭性更高。 2. **其他可能性**： - **骨巨细胞瘤（Giant Cell Tumor of Bone）**： - 但好发于20-40岁，股骨远端次之。 - 本例患者15岁，年龄不太符合。 - **恶性淋巴瘤**： - 可能出现溶骨性破坏，但通常无明显皮质突破。 3. **可能性排序**（结合病史与影像）： - 骨肉瘤（Osteosarcoma） - 尤文肉瘤（Ewing's Sarcoma） - 非骨化性纤维瘤伴恶变 4. **最可能的诊断**：基于上下文，Context 0明确提到“Lytic lesion, often multilobulated and expansile... May develop more aggressive appearance with less distinct border, cortex breakthrough, and soft tissue mass”，这与本例高度一致。因此骨肉瘤可能性最大。 5. **其他考虑因素**： - Context 1中提到的创伤性神经瘤（Traumatic Neuroma）不适用，因为病变在骨骼而非软组织。 - Context 2和3涉及其他疾病（如HADD、SC），但不符合本例特征。 **第四步：临床建议** - **下一步诊疗**： - 立即进行活检以明确诊断（首选穿刺或切开活检）。 - 如确诊为骨肉瘤，需行全身骨扫描和PET-CT排除转移灶。 - 联合多学科团队（MDT）制定治疗方案：化疗+手术。 **最终输出结构**： 1. **影像学描述**： - 客观描述核心特征（X线、CT、MRI）。 2. **诊断排序**（3个具体疾病）： - 从大到小排列，最可能的是骨肉瘤。 3. **临床建议**： - 具体下一步处置。

**影像学研判**

**影像学描述**

- **X线**：左股骨远端可见溶骨性病变，呈多房状（multilobulated）膨胀性生长；骨皮质明显破坏并突破，伴软组织肿块形成；病变边缘部分硬化，但整体边界欠清晰。
- **CT**：病灶内见分叶状低密度区，骨皮质连续性中断（cortex breakthrough），周围可见环形高密度反应性骨增生；软组织肿块与骨破坏区域相连，呈浸润性生长。
- **MRI**：T1WI示病变区为不均匀低信号，T2WI显示混杂高信号伴多房分隔；增强扫描见病灶内强化不均（提示坏死或囊变），软组织肿块与骨破坏区域相连，周围肌肉间隙受累。

**诊断可能性排序**

1. **骨肉瘤（Osteosarcoma）**：
   - 15岁青少年男性，股骨远端好发；影像表现为溶骨性多房病变、皮质突破及软组织肿块（符合Context 0中“Lytic lesion, often multilobulated and expansile… cortex breakthrough and soft tissue mass”），且病灶呈侵袭性生长（15% X线可见皮质破坏，实际约50%）。
2. **尤文肉瘤（Ewing’s Sarcoma）**：
   - 青少年好发，但本例无典型“洋葱皮样”骨膜反应；若病灶边界更模糊且软组织肿块显著，需考虑。
3. **非骨化性纤维瘤伴恶变（Non-ossifying Fibroma with Malignant Transformation）**：
   - 良性病变可呈多房状溶骨破坏，但本例疼痛1个月、皮质突破及软组织肿块提示恶性进展可能。

**临床建议**

1. **紧急活检**：行CT引导下穿刺活检（优先取软组织肿块与骨破坏交界区），明确病理诊断。
2. **全身评估**：
   - 行全身骨扫描及PET-CT排查转移灶（尤其肺部）。
3. **多学科会诊**：
   - 骨肿瘤科、放疗科、病理科联合制定方案；若确诊为骨肉瘤，需启动新辅助化疗+手术切除。

依据：本例青少年男性股骨远端溶骨性病变伴皮质破坏及软组织肿块（Context 0明确描述此类特征），高度提示恶性肿瘤，优先排除骨肉瘤。
