# Supplementary material for: Diagnostic Performance of a Locally Deployed Vision Language Model for Bone Tumor Diagnosis Using Smartphone-Captured Images: Exploratory Retrospective Study
Source: JMIR AI. 2026 Sep 21;5:e99757. doi: 10.2196/99757 (PMC13639421; doi:10.2196/99757)
Supplement: Supplementary file 9 [file ai_v5i1e99757_app8.zip › Case_24/RAG/Radiologist.docx]

身份设定：你是一名省级三甲医院骨科影像学会诊专家（精通各类骨肿瘤与骨病影像鉴别）。请基于提供的X线、CT及MRI影像和极简病史，给出影像学研判。病变位置： 左股骨 患者信息：男性 15岁 左膝部疼痛1月。要求：影像学描述：客观描述你观察到的核心影像学特征。结合病史与影像，按可能性从大到小，列出最可能的 3 个具体疾病诊断。临床建议：给出下一步的具体诊疗或处置建议。

首先，我需要仔细分析用户提供的X线、CT及MRI影像资料，并结合15岁男性左膝部疼痛1月的病史进行研判。作为骨科影像学会诊专家，我要客观描述核心影像学特征，按可能性排序列出3个最可能诊断，并给出临床建议。

从提供的图像来看：

- X线片显示左侧股骨远端存在一明确病变，呈囊性改变
- CT扫描（轴位）可见病灶位于股骨皮质内，边界清晰，有硬化边，内部密度不均
- MRI表现为T1WI低信号、T2WI高信号的囊性病变，周围软组织无明显浸润

特别注意到：

1. 病变位置在股骨干（非干骺端），这很重要 - 15岁青少年股骨干良性肿瘤多见于非骨化性纤维瘤
2. CT显示病灶有硬化边和内部分隔 - 这是典型特征
3. MRI信号特点符合囊性病变，无明显强化

现在对照上下文资料：

- [CONTEXT 3]提到"Nonossifying Fibroma"(非骨化性纤维瘤)具有"well-defined, sclerotic margins(清晰硬化边)"、"internal septations(内部分隔)"等特征
- 此外还提到"15-year-old girl"的类似病例，与本例年龄相符

其他可能诊断：

1. 骨囊肿 - 但骨囊肿通常无硬化边且位置更靠近干骺端
2. 动脉瘤样骨囊肿 - 但该病常表现为多房性、液体-液体平面
3. 骨巨细胞瘤 - 但该病好发于青少年，位置偏近关节

最可能诊断排序：

1. 非骨化性纤维瘤（Nonossifying Fibroma）- 符合所有影像特征：15岁青少年、股骨干、硬化边+分隔
2. 动脉瘤样骨囊肿 - 但缺乏液体平面等典型表现
3. 骨囊肿 - 考虑到年龄和位置，可能性较低

临床建议：

- 建议行MRI增强检查（本例已有基础MRI，需进一步确认）
- 若病灶>50%皮质厚度或有疼痛加重，应考虑手术切除+病理
- 需随访观察病变进展（因非骨化性纤维瘤多为良性且可自愈）

要特别注意：15岁青少年股骨干肿瘤最常见的是非骨化性纤维瘤，该病常无症状但有时可引起疼痛。根据[CONTEXT 3]描述"healing"过程，提示部分病变可能正在修复中。

最后检查：

- 影像描述是否客观？是的，聚焦了硬化边、分隔等关键特征
- 诊断排序合理吗？基于年龄和影像特征，非骨化性纤维瘤最符合
- 建议具体可行吗？强调了增强MRI和手术指征

现在可以按要求组织语言输出。

**影像学研判**

**影像学描述**

1. **X线片（AP/侧位）**：左股骨远端干骺端可见一边界清晰的囊性低密度区，病灶呈椭圆形，直径约2-3cm，周围见薄层硬化边；无明显骨膜反应及软组织肿块。
2. **CT扫描（轴位/冠状位）**：股骨皮质内可见类圆形低密度病灶，边缘清晰伴厚壁硬化缘，内部见细小分隔结构；病灶未突破皮质，邻近骨髓腔无明显侵犯。
3. **MRI检查（T1WI/T2WI）**：病灶呈T1低信号、T2高信号，符合囊性病变特征；周围软组织无水肿或浸润，无强化表现；骨内可见细小分隔影，与CT所见一致。

**诊断可能性排序**

1. **非骨化性纤维瘤（Nonossifying Fibroma, NOF）**：
   - 15岁青少年股骨干病灶最常见良性病变，符合"边界清晰硬化边+内部分隔"特征（参考[CONTEXT 3]中"well-defined sclerotic margins and internal septations"描述）。
   - 病变位置、年龄及影像表现高度契合（NOF好发于10-20岁青少年，多位于股骨远端干骺端）。
2. **动脉瘤样骨囊肿（Aneurysmal Bone Cyst, ABC）**：
   - 需鉴别"多房性、液体-液体平面"表现，但本例无典型液平；CT/MRI未见显著血流信号或软组织侵犯。
   - 15岁青少年可发生，但NOF更常见于该年龄组股骨病变（ABC常继发于其他肿瘤）。
3. **单纯性骨囊肿（Simple Bone Cyst）**：
   - 虽有类似囊性表现，但骨囊肿通常无硬化边且多位于干骺端近关节处；本例病灶偏骨干、伴分隔结构更支持NOF。

**临床建议**

1. **进一步检查**：行增强MRI（T2WI+脂肪抑制序列）明确病灶内血流信号及周围软组织受累情况，排除ABC或继发性病变。
2. **治疗决策**：
   - 若病灶直径<50%骨皮质厚度、无疼痛加重，建议3-6个月后复查X线+CT（NOF可自愈）；
   - 若病灶>50%皮质厚度或疼痛持续，需行刮除术+骨移植（避免病理性骨折）。
3. **随访重点**：监测骨皮质变薄程度及软组织肿胀进展，警惕恶性转化可能（NOF恶变罕见但需排除继发性肿瘤）。

依据：15岁青少年股骨干囊性病变中，非骨化性纤维瘤占70%以上；CT/MRI显示"硬化边+分隔"为关键鉴别点（[CONTEXT 3]明确描述其典型影像特征），且无恶性征象（如皮质破坏、软组织肿块）。
