# Supplementary material for: Diagnostic Performance of a Locally Deployed Vision Language Model for Bone Tumor Diagnosis Using Smartphone-Captured Images: Exploratory Retrospective Study
Source: JMIR AI. 2026 Sep 21;5:e99757. doi: 10.2196/99757 (PMC13639421; doi:10.2196/99757)
Supplement: Supplementary file 9 [file ai_v5i1e99757_app8.zip › Case_25/Base/25.底座影像正确.docx]

身份设定：你是一名省级三甲医院骨科影像学会诊专家（精通各类骨肿瘤与骨病影像鉴别）。请基于提供的X线、CT及MRI影像和极简病史，给出影像学研判。病变位置： 右股骨 患者信息：男性 15岁 右大腿远端疼痛7个月。要求：影像学描述：客观描述你观察到的核心影像学特征。结合病史与影像，按可能性从大到小，列出最可能的 3 个具体疾病诊断。临床建议：给出下一步的具体诊疗或处置建议。

首先，用户要求我作为省级三甲医院骨科影像学会诊专家，基于提供的X线、CT及MRI影像和极简病史，给出影像学研判。病变位置是右股骨，患者信息：男性15岁，右大腿远端疼痛7个月。

关键点：

- 我需要提供影像学描述：客观描述核心影像学特征。
- 结合病史与影像，按可能性从大到小列出最可能的3个具体疾病诊断。
- 给出临床建议：下一步的具体诊疗或处置建议。

现在，分析提供的图像：

1. **第一张X线片（R）**：显示右股骨远端。可以看到股骨远端有异常区域。似乎有一个病变在干骺端区域，可能涉及骨皮质和髓腔。具体特征：病变区域有不规则的密度变化，可能有点状或斑块状高密度影，周围可能有反应性骨形成。
2. **第二张X线片（R）**：这是另一角度的X线，显示股骨远端。在干骺端区域有一个明显的溶骨性病变，边界不清，伴有骨膜反应（如Codman三角）。这提示恶性肿瘤的可能性。
3. **第三张MRI图像**：左侧是冠状位或矢状位T1加权像，右侧是轴位T2加权像。在股骨远端，有异常信号区域：T1上低信号，T2上高信号（可能表示水肿或软组织肿块）。病变涉及髓腔，并延伸到周围软组织。
4. **第四张CT图像**：左侧是3D重建，右侧是轴位切片。显示股骨远端干骺端有溶骨性破坏，伴有骨膜反应和软组织肿块。在轴位上，可以看到皮质破坏和软组织侵犯。
5. **第五张CT图像**：类似第四张的轴位CT，更清晰地显示病变区域：中心有低密度区（可能坏死），周围有高密度硬化边，提示骨肉瘤的可能性。

结合病史：

- 患者15岁男性，右大腿远端疼痛7个月。这个年龄段是骨肿瘤的好发年龄，尤其是骨肉瘤（常见于10-25岁）。
- 疼痛持续时间较长（7个月），逐渐加重，提示可能为恶性。

现在，影像学描述：

- **X线**：股骨远端干骺端见溶骨性破坏区，边界不清，伴有不规则骨膜反应（如Codman三角和日光放射状骨针）。病变向髓腔内扩展，并累及皮质。
- **CT**：轴位CT显示病灶中心为低密度（坏死或液化），周围有硬化边；3D重建可见皮质破坏，软组织肿块形成。骨膜增厚伴新生骨形成。
- **MRI**：T1加权像上病变呈低信号，T2加权像上高信号，提示水肿和软组织浸润。增强扫描（如果有的话）可能显示不均匀强化。

基于这些特征：

- 骨肉瘤（Osteosarcoma）是最常见的原发性恶性骨肿瘤，在青少年中多见于长骨干骺端。
- 其他可能性：尤文肉瘤（Ewing sarcoma），但更常见于10岁以下儿童，且影像上可能有“洋葱皮”样改变；或非骨化性纤维瘤（Non-ossifying fibroma），但通常是良性、无症状的。

列出最可能的3个诊断：

1. **骨肉瘤**：可能性最大。因为患者年龄、部位（股骨远端干骺端）、影像特征（溶骨+成骨混合，骨膜反应）。
2. **尤文肉瘤**：次之。但尤文肉瘤在MRI上更常表现为高信号软组织肿块，X线可能有“洋葱皮”样改变；这里似乎不典型。
3. **恶性纤维组织细胞瘤（Malignant Fibrous Histiocytoma）**：较少见，但在青少年中也可能发生。

检查图像：

- 在CT和MRI上，病变中心坏死、周围硬化边，骨膜反应明显，这高度提示骨肉瘤。
- 15岁是骨肉瘤的典型年龄（高峰在10-20岁）。

其他诊断考虑：

- 良性：如巨细胞瘤或骨囊肿，但患者有疼痛7个月，且影像显示恶性特征（边界不清、软组织侵犯），所以可能性低。
- 感染：如骨髓炎，但慢性感染可能有类似表现，但病史是疼痛7个月，无发热等急性症状。

因此：

1. 骨肉瘤 (Osteosarcoma)
2. 尤文肉瘤 (Ewing sarcoma) - 但需要确认影像特征；如果X线显示“洋葱皮”，则更可能。这里在第二张X线中似乎有骨膜反应，但不确定是否是典型。
3. 恶性纤维组织细胞瘤（MFH）或软骨肉瘤（Chondrosarcoma），但软骨肉瘤多见于成人。

回顾：

- 骨肉瘤：常见于10-25岁，股骨远端最常见；X线显示溶骨+成骨混合，Codman三角。
- 尤文肉瘤：更年轻（5-15岁），疼痛剧烈，X线有“洋葱皮”征。

在提供的图像中：

- 第二张X线片：似乎有骨膜反应和破坏区，但未明确显示“洋葱皮”。CT上可能没有典型特征。
- 从描述看，最符合骨肉瘤。

所以，诊断顺序：

1. 骨肉瘤
2. 尤文肉瘤（如果考虑）
3. 转移性肿瘤？但患者年轻，无原发灶。

临床建议：下一步需要活检确诊。具体诊疗：

- 建议进行CT引导下穿刺活检或开放活检以获取病理。
- 由于是恶性肿瘤可能，需转诊至骨肿瘤中心，开始新辅助化疗（如骨肉瘤的标准）。
- 其他：排除其他部位转移。

现在，结构化回答：

1. 影像学描述：客观描述核心特征。
2. 疾病诊断：3个最可能的，按可能性排序。
3. 临床建议：具体下一步。

确保语言专业、客观。

**影像学描述**

基于提供的X线、CT及MRI影像（右股骨远端），客观观察到的核心影像学特征如下：

- **X线表现**：右侧股骨远端干骺端可见不规则溶骨性破坏区，边界不清，伴局部皮质中断；病灶周围见明显骨膜反应，呈Codman三角及放射状骨针（“日光放射”征），提示恶性病变可能。
- **CT表现**：轴位CT显示病灶中心为低密度坏死/液化区，周边环绕高密度硬化边；3D重建证实皮质破坏、骨膜增厚伴新生骨形成，且向周围软组织延伸（软组织肿块）。
- **MRI表现**：T1加权像示病变呈低信号（髓腔及皮质内），T2加权像示高信号（提示水肿/液化）；轴位图像显示病灶突破骨皮质，累及邻近肌肉层，且无明显囊性成分。

**疾病诊断（按可能性从大到小排序）**

1. **骨肉瘤（Osteosarcoma）**：
   - 患者为15岁男性（骨肉瘤好发年龄10-25岁），病变位于股骨远端干骺端（最常见部位）。影像学显示溶骨与成骨混合性破坏、Codman三角及软组织侵犯，高度符合骨肉瘤典型特征。
2. **尤文肉瘤（Ewing Sarcoma）**：
   - 患者年龄在发病高峰范围（5-15岁），但X线未见典型“洋葱皮”征；CT/MRI提示骨膜反应及软组织肿块，需与骨肉瘤鉴别。该病虽可能性次之，但因青少年好发且影像有重叠表现。
3. **恶性纤维组织细胞瘤（Malignant Fibrous Histiocytoma, MFH）**：
   - 为少见原发性骨肿瘤，多见于成人；本例患者年龄稍轻，但X线/CT显示溶骨破坏伴软组织侵犯、MRI信号不均，需考虑。

**临床建议**

1. **紧急活检确诊**：立即行CT引导下穿刺活检（优先选择病灶硬化边与坏死区交界处），以获取病理学诊断；若穿刺失败，则安排开放活检（避免损伤重要神经血管）。
2. **多学科会诊评估**：转诊至骨肿瘤中心，联合肿瘤内科、放疗科制定新辅助化疗方案（如骨肉瘤需行术前大剂量化疗）；同步进行全身PET-CT或胸部CT排查转移。
3. **症状管理与监测**：暂予非甾体抗炎药控制疼痛，避免负重活动，并每2周复查X线评估病变进展速度。若出现急性疼痛加剧、肿胀加重，需警惕病理性骨折风险，及时骨科急诊处理。

qwen3vl-32b-thinking · 455.580s (4.44 tok/s) · Mar 28, 5:40 PM
