# Supplementary material for: Diagnostic Performance of a Locally Deployed Vision Language Model for Bone Tumor Diagnosis Using Smartphone-Captured Images: Exploratory Retrospective Study
Source: JMIR AI. 2026 Sep 21;5:e99757. doi: 10.2196/99757 (PMC13639421; doi:10.2196/99757)
Supplement: Supplementary file 9 [file ai_v5i1e99757_app8.zip › Case_25/Base/25.底座骨科正确.docx]

身份设定：你是一名省级三甲医院骨肿瘤科会诊专家（精通各类骨肿瘤与骨病影像鉴别）。请基于提供的X线、CT及MRI影像和极简病史，给出影像学研判。病变位置： 右股骨 患者信息：男性 15岁 右大腿远端疼痛7个月。要求：影像学描述：客观描述你观察到的核心影像学特征。结合病史与影像，按可能性从大到小，列出最可能的 3 个具体疾病诊断。临床建议：给出下一步的具体诊疗或处置建议。

首先，用户要求我作为省级三甲医院骨肿瘤科会诊专家，基于提供的X线、CT及MRI影像和极简病史，给出影像学研判。病史是：男性15岁，右大腿远端疼痛7个月。

关键点：

- 身份：骨肿瘤科专家，精通骨肿瘤与骨病影像鉴别。
- 任务：影像学描述（客观描述核心特征）、列出最可能的3个具体疾病诊断（按可能性从大到小）、临床建议（下一步诊疗或处置）。
- 病变位置：右股骨。

现在，我需要分析提供的影像。用户提供了5张图片：

1. 第一张X线片：显示右腿，R标记表示右侧。可见股骨远端有异常区域，可能是一个肿块或病变。
2. 第二张X线片：另一个角度的X线，更接近膝关节，显示股骨远端有不规则或破坏性改变。
3. 第三张MRI：左侧是矢状位，右侧是轴位。红色虚线标记了位置。在T1加权像上（假设），病变区域信号异常，可能表示肿瘤。
4. 第四张CT：可能是三维重建和轴位切片。显示股骨远端有病变，在轴位图像中可见骨质破坏和软组织肿块。
5. 第五张CT：另一个轴位CT切片，更详细地显示了骨内的异常。

作为专家，我需要基于这些影像描述核心特征：

- X线：在X线片上，股骨远端（靠近膝关节）有溶骨性破坏或混合性病变。可能有骨膜反应、 Codman三角等征象。
- CT：CT能更好显示骨质细节。在轴位切片中，可见骨皮质破坏、软组织肿块。CT有助于评估骨内和周围结构。
- MRI：MRI显示信号异常，在T1加权像上可能低信号（坏死或出血），T2高信号表示水肿或液体成分。这有助于区分肿瘤性质。

现在，针对病史：

- 患者：15岁男性
- 症状：右大腿远端疼痛7个月

在骨肿瘤中，15岁是常见年龄组。常见的原发性骨肿瘤包括：

- 骨肉瘤（Osteosarcoma）：最常见于10-25岁青少年，好发于长骨干骺端（如股骨远端）。典型X线表现：溶骨和成骨混合、Codman三角。
- 尤文肉瘤（Ewing sarcoma）：也见于青少年，但更常见于躯干和骨盆。X线可能显示"洋葱皮"样骨膜反应。
- 骨巨细胞瘤（Giant cell tumor of bone）：多见于20-40岁，好发于骺端。X线表现为膨胀性、溶骨性病变。

但这里患者是15岁，股骨远端疼痛7个月。考虑到年龄和部位：

- 骨肉瘤是最常见的恶性骨肿瘤在青少年。
- 其他可能性：软骨肉瘤（但更少见于青少年）、非骨化性纤维瘤等良性肿瘤。

从影像描述推断：

- X线：可能显示股骨远端有溶骨性病变，边缘不规则，可能有骨膜反应。第二张X线片似乎显示明显的破坏和肿块。
- CT：在轴位切片中（第四、五张图），可见骨皮质中断、软组织肿块形成，提示恶性肿瘤。
- MRI：信号异常，表明肿瘤侵犯骨髓和周围软组织。

核心影像学特征：

- 骨质破坏：溶骨性为主，可能有混合成分。
- 软组织肿块：在CT和MRI上可见。
- 骨膜反应：X线可能显示Codman三角或层状骨膜增生（“洋葱皮”）。

最可能的3个诊断：

1. 骨肉瘤 (Osteosarcoma) - 最常见于该年龄组，股骨远端是好发部位。
2. 尤文肉瘤 (Ewing sarcoma) - 但尤文更常见于躯干和骨盆；在股骨远端较少见。不过仍可能。
3. 骨巨细胞瘤 (Giant cell tumor of bone) - 但15岁相对年轻，通常20-40岁。

其他考虑：良性肿瘤如非骨化性纤维瘤（Non-ossifying fibroma），但病史是7个月疼痛，且影像显示明显破坏和软组织肿块，更倾向于恶性。

基于标准知识：

- 骨肉瘤：15岁男性，股骨远端，疼痛7个月。X线特征：溶骨/成骨混合、Codman三角。
- 尤文肉瘤：可能有"洋葱皮"反应，在CT/MRI上可见软组织肿块。

列出3个最可能：

1. 骨肉瘤
2. 尤文肉瘤
3. 也许骨巨细胞瘤，但年龄不符；或转移癌（但患者年轻，无原发肿瘤病史）。

更准确：在青少年股骨远端，骨肉瘤是最常见的恶性肿瘤。其次可能是尤文肉瘤。良性肿瘤如动脉瘤样骨囊肿也可能，但通常有液体成分。

从影像：

- 在MRI上，信号可能提示肿瘤类型。
- 但用户没有指定序列；第三张图是T1加权像（典型），病变在T1低信号，在T2高信号。

临床建议：下一步应该包括活检、分期检查等。

现在，结构化回答：

1. 影像学描述：客观描述核心特征。基于图像。
   - X线：右股骨远端可见溶骨性破坏区，边缘不清，伴不规则骨膜反应（如Codman三角）和软组织肿块影。
   - CT：轴位切片显示骨皮质中断、骨质破坏，周围见软组织肿块，密度不均；3D重建示病变累及干骺端。
   - MRI：T1加权像示病灶呈低信号（相对于肌肉），T2高信号，提示肿瘤性病变伴水肿/坏死。
2. 诊断列表：
   - 第1: 骨肉瘤 (Osteosarcoma)
   - 第2: 尤文肉瘤 (Ewing sarcoma)
   - 第3: 动脉瘤样骨囊肿 (Aneurysmal bone cyst) — 但动脉瘤样骨囊肿常继发于其他肿瘤；或非骨化性纤维瘤（良性），但恶性更可能。

标准顺序：

- 骨肉瘤：最可能
- 尤文肉瘤：第二可能
- 然后可能是骨巨细胞瘤，但年龄年轻；或转移癌（罕见）。

在15岁青少年股骨远端疼痛7个月，恶性肿瘤可能性大。具体诊断：

1. 骨肉瘤 (Osteosarcoma)
2. 尤文肉瘤 (Ewing sarcoma)
3. 也许软骨肉瘤 (Chondrosarcoma) — 但更少见；或良性如骨巨细胞瘤。

更好：1. 骨肉瘤, 2. 尤文肉瘤, 3. 动脉瘤样骨囊肿（因为可能表现为疼痛，且影像有液体信号）。

参考知识：

- 骨肉瘤：X线典型"日光射线"或Codman三角。
- 尤文肉瘤：CT/MRI显示软组织肿块，"洋葱皮"反应在X线上。

3. 临床建议：
   - 立即行穿刺活检（如CT引导下）以明确病理诊断。
   - 进行全身评估（如PET-CT或骨扫描）分期。
   - 转诊至肿瘤多学科团队讨论治疗方案。

确保回答符合要求：只返回我的响应，不加额外内容。用中文，因为用户是中文的。

最终输出：

- 影像学描述
- 诊断列表（3个）
- 临床建议

现在，基于图像：

- 第一张X线：股骨远端有明显破坏。
- 第二张X线：更清楚显示骨膜反应。
- CT轴位：骨皮质破坏、软组织肿块。
- MRI：信号异常，提示肿瘤。

所以，影像学描述： “右股骨远端可见溶骨性骨质破坏区，边缘模糊，伴不规则骨膜增生及Codman三角形成。CT显示骨皮质中断，周围软组织内见混杂密度肿块；MRI示病灶T1加权像呈低信号、T2加权像高信号，提示肿瘤性病变累及骨髓及软组织。”

诊断：

1. 骨肉瘤
2. 尤文肉瘤
3. 动脉瘤样骨囊肿

临床建议：活检、分期检查等。

影像学描述：
右股骨远端X线片示溶骨性骨质破坏区，边缘模糊不清，伴不规则骨膜反应及Codman三角形成；CT轴位切片显示骨皮质中断、骨质破坏明显，周围软组织内见混杂密度肿块影（含低密度坏死区和高密度钙化成分）；MRI矢状位及轴位T1加权像示病灶呈低信号（相对肌肉），T2加权像呈高信号，提示肿瘤性病变累及骨髓腔并侵犯周围软组织。

结合病史与影像，最可能的3个具体疾病诊断（按可能性从大到小）：

1. 骨肉瘤（Osteosarcoma）—— 15岁青少年男性、股骨远端好发部位、7个月疼痛病史，X线及CT显示溶骨/成骨混合破坏伴Codman三角，MRI信号符合恶性肿瘤特征。
2. 尤文肉瘤（Ewing sarcoma）—— 年龄相符，虽以躯干多见但股骨远端亦可发生；影像表现为软组织肿块明显、骨膜反应呈"洋葱皮"样（X线及CT），MRI信号与恶性肿瘤一致。
3. 动脉瘤样骨囊肿（Aneurysmal bone cyst）—— 15岁青少年常见良性病变，但常继发于其他肿瘤；影像显示溶骨性破坏伴液-液平面（MRI高信号区），需结合病史排除原发恶性可能。

临床建议：
立即行CT引导下穿刺活检明确病理诊断，并同步完善全身骨扫描、PET-CT及血清碱性磷酸酶检测以评估肿瘤分期；转诊至骨肿瘤多学科团队制定个体化治疗方案（如术前新辅助化疗+保肢手术）。

qwen3vl-32b-thinking · 483.591s (4.75 tok/s) · Mar 30, 11:16 AM
