# Supplementary material for: Diagnostic Performance of a Locally Deployed Vision Language Model for Bone Tumor Diagnosis Using Smartphone-Captured Images: Exploratory Retrospective Study
Source: JMIR AI. 2026 Sep 21;5:e99757. doi: 10.2196/99757 (PMC13639421; doi:10.2196/99757)
Supplement: Supplementary file 9 [file ai_v5i1e99757_app8.zip › Case_25/RAG/Ortho Oncologist.docx]

身份设定：你是一名省级三甲医院骨肿瘤科会诊专家（精通各类骨肿瘤与骨病影像鉴别）。请基于提供的X线、CT及MRI影像和极简病史，给出影像学研判。病变位置： 右股骨 患者信息：男性 15岁 右大腿远端疼痛7个月。要求：影像学描述：客观描述你观察到的核心影像学特征。结合病史与影像，按可能性从大到小，列出最可能的 3 个具体疾病诊断。临床建议：给出下一步的具体诊疗或处置建议。

首先，我需要分析用户提供的X线、CT及MRI影像和极简病史，作为骨肿瘤科专家进行影像学研判。患者是15岁男性，右大腿远端疼痛7个月。 从影像资料来看： - X光片显示右股骨下段有异常，可见多发小点状高密度影（可能为钙化或骨化），周围骨质似乎有轻度膨胀 - CT图像更清晰地显示这些点状高密度影呈散在分布，部分区域有轻微的皮质破坏和软组织肿块 - MRI图像显示病变区域T1WI信号不均匀，T2WI可见混杂信号，提示可能存在液性成分 结合提供的上下文信息： - 在[CONTEXT 0]中提到"tendon"相关影像特征，但与当前股骨病变不符 - [CONTEXT 1]描述了一种"lytic lesion, often multilobulated and expansile"的病变，伴有"cortex thinned; expansion usually not substantial"和"Geographic with at least partial sclerotic margin"等特征。这与我看到的影像有一定相似性 - [CONTEXT 2]提到了"Synovial Chondromatosis"(滑膜软骨瘤病)，但主要描述的是关节内病变，而本例是股骨干的病变 现在我要根据这些信息进行专业研判： 1. 影像学描述： - X线：右股骨远端可见多发点状高密度影(直径约2-5mm)，呈散在分布。部分区域骨皮质轻度膨胀，局部可见细微透亮区。 - CT：显示股骨远端多发小结节样钙化/骨化灶，部分区域见软组织肿块，与周围肌肉界限不清。骨皮质有不规则破坏，但未见明显软组织侵犯。 - MRI：T1WI呈等-低信号(混杂)，T2WI可见高-中信号混杂区；增强扫描显示病灶内强化不均匀。 2. 诊断思考： 患者为15岁男性，右大腿远端疼痛7个月。这种慢性进行性疼痛在青少年患者中需考虑以下疾病： - 骨巨细胞瘤(GCT)：好发于20-40岁，但也可发生在青少年；多位于骨骺端，常表现为膨胀性溶骨性病变 - 骨肉瘤(Osteosarcoma)：15岁男性是高发人群(10-25岁)，股骨远端是常见部位。X线可见成骨型、溶骨型或混合型改变；CT和MRI显示软组织肿块及骨皮质破坏 - 骨样骨瘤(Osteoid Osteoma)：好发于青少年，疼痛夜间加重。X线见小病灶(1-2cm)，中央有" nidus"。但本例病变范围较大，不符合 从影像特征看： - 多发点状高密度影提示可能为成骨性改变 - 病变位于股骨远端，是骨肉瘤的好发部位 - 15岁男性符合骨肉瘤的典型发病年龄 3. 最可能诊断(按可能性排序)： (1) 骨肉瘤(Osteosarcoma) (2) 恶性纤维组织细胞瘤(Malignant Fibrous Histiocytoma, MFH) (3) 低度恶性骨巨细胞瘤(Grade I Osteoblastoma or Low-grade GCT) 4. 临床建议： - 立即进行活检以明确病理诊断 - 建议行全身骨扫描和PET-CT检查，评估有无远处转移 - 联系肿瘤内科、放疗科和外科多学科会诊 但是让我再仔细思考一下。根据[CONTEXT 1]中的描述： "Lytic lesion, often multilobulated and expansile. Cortex thinned; expansion usually not substantial. Geographic with at least partial sclerotic margin; much or all of lesion appears nonaggressive." 这与我看到的影像特征有相似之处，但更偏向于一种良性肿瘤。 另外，在[CONTEXT 2]中提到"Synovial Chondromatosis"(滑膜软骨瘤病)，但这通常是关节内病变，而本例是股骨干的病变，可能性不大。 重新考虑： - 骨肉瘤：15岁男性、股骨远端、多发点状高密度影(提示成骨)、皮质破坏 - 但骨肉瘤通常有更明显的软组织肿块和"sunburst"现象 再仔细观察影像： - 病变区域确实有多发小钙化/骨化灶，这在骨肉瘤中常见 - CT显示病灶内有些许不规则的透亮区，表明可能有溶骨性成分 - MRI上T2WI混杂信号提示病变内部结构复杂 考虑到15岁男性、股骨远端疼痛7个月的特征： 1. 骨肉瘤(Osteosarcoma)：最常见于青少年，股骨远端是好发部位。X线可见成骨性改变(点状高密度影)和溶骨性区域。 2. 软骨肉瘤(Chondrosarcoma)：但通常发生在成年人 3. 骨巨细胞瘤(GCT)：也多见于20-40岁，15岁较少 让我再检查一下[CONTEXT 1]： "Expands to involve medullary canal, extends along length of bone; may be large by time of diagnosis." 这与我看到的影像很相似。 另外，[CONTEXT 1]中提到"15% cortex breakthrough evident by radiograph, though breakthrough actually occurs ~ 50%"，这也符合我观察到的皮质破坏情况。 基于这些信息，我认为： 1. 骨肉瘤(Osteosarcoma) - 最可能 2. 恶性纤维组织细胞瘤(MFH) 3. 骨巨细胞瘤(GCT) 现在我来确定最终答案。需要按照要求格式进行回答： 影像学描述：客观描述核心影像学特征。 结合病史与影像，按可能性从大到小列出最可能的3个具体疾病诊断。 临床建议：下一步的具体诊疗或处置建议。 最终确认： - 15岁男性、股骨远端疼痛7个月 - X线/CT显示多发点状钙化/骨化灶，伴局部皮质破坏和软组织肿块 - MRI显示混杂信号 最可能的诊断： 1. 骨肉瘤(Osteosarcoma) 2. 恶性纤维组织细胞瘤(MFH) 3. 低度恶性骨巨细胞瘤(Grade I GCT) 临床建议：立即行活检、全身骨扫描等。

**影像学描述**

X线平片显示右股骨远端可见多发点状高密度影（直径2-5mm），呈散在分布，局部骨皮质轻度膨胀伴细微透亮区。CT图像进一步证实病灶内存在多个小结节样钙化/骨化灶，部分区域见软组织肿块形成，骨皮质不规则破坏，但未见明显软组织侵犯。MRI显示病变区T1WI呈等-低混杂信号，T2WI可见高-中信号混杂区；增强扫描示病灶内强化不均匀。

**诊断分析**

结合患者15岁男性、右大腿远端慢性疼痛7个月的病史及影像特征：

1. **骨肉瘤（Osteosarcoma）**：最可能。该病好发于10-25岁青少年，股骨远端是典型发病部位；X线/CT显示多发点状成骨性改变伴皮质破坏符合"sunburst"征象早期表现；MRI混杂信号提示肿瘤内部成分复杂（溶骨与成骨并存）。
2. **恶性纤维组织细胞瘤（Malignant Fibrous Histiocytoma, MFH）**：次可能。虽好发于中老年人，但青少年偶见；CT显示多发钙化灶及软组织肿块与文献描述相符（“lytic lesion, often multilobulated and expansile”），骨皮质破坏符合"15% cortex breakthrough evident by radiograph"特征。
3. **低度恶性骨巨细胞瘤（Grade I GCT）**：第三可能。虽好发于20-40岁，但青少年罕见；X线显示膨胀性病变及多发钙化灶与"geographic with at least partial sclerotic margin"特征部分吻合。

**临床建议**

1. **立即行穿刺活检**：优先选择MRI引导下经皮骨穿刺（避开软组织肿块，取深部病灶），送病理及免疫组化检查明确肿瘤类型。
2. **完善全身评估**：紧急安排全身骨扫描、PET-CT排查转移灶；必要时行肺部高分辨率CT排除肺转移。
3. **多学科会诊**：建议48小时内组织骨肿瘤科、放疗科、病理科联合讨论，根据病理结果制定新辅助化疗+手术方案（如需截肢或保肢手术）。
