# Supplementary material for: Diagnostic Performance of a Locally Deployed Vision Language Model for Bone Tumor Diagnosis Using Smartphone-Captured Images: Exploratory Retrospective Study
Source: JMIR AI. 2026 Sep 21;5:e99757. doi: 10.2196/99757 (PMC13639421; doi:10.2196/99757)
Supplement: Supplementary file 9 [file ai_v5i1e99757_app8.zip › Case_25/RAG/Radiologist.docx]

身份设定：你是一名省级三甲医院骨科影像学会诊专家（精通各类骨肿瘤与骨病影像鉴别）。请基于提供的X线、CT及MRI影像和极简病史，给出影像学研判。病变位置： 右股骨 患者信息：男性 15岁 右大腿远端疼痛7个月。要求：影像学描述：客观描述你观察到的核心影像学特征。结合病史与影像，按可能性从大到小，列出最可能的 3 个具体疾病诊断。临床建议：给出下一步的具体诊疗或处置建议。

首先，我需要分析用户提供的X线、CT及MRI影像资料，并结合病史（15岁男性，右大腿远端疼痛7个月），按照要求进行影像学研判。

从给定的影像资料来看：

- 第一张图：右侧股骨的X光片显示在股骨远端有异常改变
- 第二张图：另一角度的X光片，更清晰地展示了股骨远端的病变区域
- 第三张图：MRI图像（矢状位和轴位），显示出病灶的信号特点
- 第四张图：CT图像（冠状位和横断面），显示了骨质改变

现在我来分析这些影像特征：

1. X线表现：
   - 在股骨远端可见一个边界相对清晰、膨胀性生长的病变区域
   - 病变区有明显的斑点状钙化或骨化影（在X光片上表现为高密度点状结构）
   - 骨皮质局部增厚，但无明显破坏
2. CT表现：
   - 横断面显示病灶内有多发小的钙化/骨化结节
   - 病变区域呈囊性改变，内部有分隔
   - 皮质骨轻微膨胀，但未见明显骨皮质破坏
3. MRI表现：
   - T1加权像：病变区信号不均匀，以低信号为主（对应钙化/骨化部分），间杂中等信号区域
   - T2加权像：病灶内可见多发小的低信号结节（对应钙化/骨化部分）
   - 病变周围软组织无明显异常

结合提供的上下文信息，我需要参考这些资料：

- 从CONTEXT 0中，我们了解到关于肌腱病变的信息，但似乎与本病例不太相关
- CONTEXT 1提到了"Synovial Chondromatosis"(滑膜骨软骨瘤病)，描述了多发的圆形钙化/骨化结节，这与我观察到的影像特征非常相似：多个小的圆点状高密度影（在X线和CT上可见）
- CONTEXT 2提到了一些骨病变的特点，但似乎不是最匹配的
- CONTEXT 3也提及了"Synovial Chondromatosis"，描述了"numerous speckled intermediate SI filling defects, characteristic of SC"（多个斑点状中等信号填充缺损，这是滑膜骨软骨瘤病的特征）

基于这些信息和影像表现：

1. 影像学描述：
   - 右股骨远端可见一膨胀性病变
   - 病变内多发小圆形钙化/骨化结节（X线、CT上清晰显示）
   - MRI示：T1加权像呈低-中等信号，T2加权像以高信号为主，但见多发点状低信号区（对应钙化/骨化部分）
   - 病变边界相对清楚，无明显皮质破坏
2. 最可能的3个诊断（按可能性从大到小）：
   1. 滑膜骨软骨瘤病(Synovial Chondromatosis, SC)
      - 这与我的观察高度一致：多发、圆形、钙化/骨化的结节，无明显恶性特征
      - 年龄（15岁男性）也符合SC的发病年龄特点（通常在20-40岁之间，但也可发生在青少年）
   2. 骨软骨瘤(Osteochondroma)
      - 虽然骨软骨瘤通常是单发、有蒂病变，但在某些情况下也可能表现为多灶性
      - X线和CT显示的钙化/骨化结节与骨软骨瘤的软骨帽钙化相似
   3. 骨巨细胞瘤(Giant Cell Tumor of Bone)
      - 虽然GCT通常不表现出典型的多发钙化，但某些类型可能有轻微钙化
      - GCT常见于20-40岁人群，但在青少年也可发生

但是，我需要再仔细分析。在 CONTEXT 1 和 CONTEXT 3 中明确提到了"Synovial Chondromatosis"的特征：多个圆形、点状的钙化/骨化结节（X线和CT上可见），这与患者的影像表现非常一致。

此外，在CONTEXT 3中提到：“numerous speckled intermediate SI filling defects, characteristic of SC”，即"多个斑点状中间信号填充缺损，是SC的特征性表现"。在MRI图像中我们也可以看到类似的表现：多发低信号结节（对应钙化/骨化部分）。

再来看 CONTEXT 1 中描述：“multiple round chondroid calcifications of similar, but not identical, sizes located both intraarticularly and extraarticularly”，即多个大小相似但不完全相同的软骨样钙化灶，在关节内和关节外都有分布。而本病例的病变位于股骨远端，这可能与膝关节相关。

因此，最有可能的诊断应该是滑膜骨软骨瘤病(Synovial Chondromatosis)。

再看其他可能性：

- 骨软骨瘤：通常表现为单发、有蒂的外生性骨肿瘤，在X线和CT上显示为从骨表面突出的病变。本病例中，病变似乎更广泛，且有多发小结节。
- 骨巨细胞瘤：多发生在关节附近（如股骨远端），但典型的GCT在X线上表现为膨胀性、偏心性的溶骨性病变，边界清晰，无明显钙化。

所以，我重新排序：

1. 滑膜骨软骨瘤病(Synovial Chondromatosis)
2. 骨巨细胞瘤(Giant Cell Tumor of Bone)
3. 骨软骨瘤(Osteochondroma)

但需要再仔细分析。从CT图像看，病变似乎主要位于股骨远端的干骺端区域，并且有明显的钙化/骨化结节。

在 CONTEXT 2 中描述了"Lyric lesion, often multilobulated and expansile. Cortex thinned; expansion usually not substantial."（溶骨性病灶，常为多叶状和膨胀性的。皮质变薄；通常无明显扩张。）这与我们观察到的影像特征不完全一致。

基于所有信息，我认为最可能的诊断是滑膜骨软骨瘤病(Synovial Chondromatosis)。

现在整理我的答案：

1. 影像学描述：客观描述核心影像学特征
2. 3个具体疾病诊断（按可能性从大到小）
3. 临床建议

让我来组织回答：

**影像学研判**

**影像学描述：**

- X线表现：右股骨远端可见一膨胀性病变，边界相对清晰，病灶内呈多发斑点状高密度影（钙化/骨化结节），皮质轻度增厚但无明显破坏。
- CT表现：横断面显示病灶内部有多个小圆形、均匀的钙化/骨化结节，呈"珍珠样"分布；病变区域呈囊性改变伴分隔，周围骨皮质轻微膨胀但完整。
- MRI表现：T1加权像示病灶内多发低信号（对应钙化/骨化部分），间杂中等信号区域；T2加权像显示高信号背景中有多发点状低信号结节。病变边界清晰，周围软组织无明显异常。

**最可能的3个具体疾病诊断：**

1. **滑膜骨软骨瘤病(Synovial Chondromatosis, SC)**
   - 依据：多发、圆形钙化/骨化结节（X线及CT上清晰可见），与CONTEXT 1和3中描述"multiple round chondroid calcifications of similar sizes"特征高度吻合；MRI示"numerous speckled intermediate SI filling defects"（多发斑点状中间信号填充缺损）符合SC典型表现。
   - 病史支持：15岁男性，右大腿远端疼痛7个月，符合SC好发于青少年-青壮年、病程迁延的临床特点。
2. **骨巨细胞瘤(Giant Cell Tumor of Bone, GCT)**
   - 依据：股骨远端为GCT常见部位（占40%），X线显示膨胀性溶骨性病变；但本例钙化结节表现不典型，需进一步鉴别。
   - 排除点：GCT通常无多发钙化，且更易见"肥皂泡样"改变。
3. **骨软骨瘤(Osteochondroma)**
   - 依据：X线可见骨性突起伴钙化软骨帽（与病灶内斑点状高密度影相符）。
   - 排除点：多为单发、有蒂病变，本例呈弥漫性多结节表现不符典型特征。

**临床建议：**

1. **立即行关节镜检查+活检**：明确诊断需获取病理标本（滑膜骨软骨瘤病需排除继发性恶性变）。
2. **完善肿瘤标志物检测**：如碱性磷酸酶、CA-125等，排除其他原发骨肿瘤可能。
3. **多学科会诊**：建议联合骨科、肿瘤内科及放疗科制定治疗方案（SC首选手术切除+关节镜清理；若病理证实为恶性变需化疗）。
4. **随访监测**：术后3个月复查X线/CT，观察有无新发结节或局部复发。
