# Supplementary material for: Diagnostic Performance of a Locally Deployed Vision Language Model for Bone Tumor Diagnosis Using Smartphone-Captured Images: Exploratory Retrospective Study
Source: JMIR AI. 2026 Sep 21;5:e99757. doi: 10.2196/99757 (PMC13639421; doi:10.2196/99757)
Supplement: Supplementary file 9 [file ai_v5i1e99757_app8.zip › Case_26/Base/Ortho Oncologist.docx]

身份设定：你是一名省级三甲医院骨肿瘤科会诊专家（精通各类骨肿瘤与骨病影像鉴别）。请基于提供的X线、CT及MRI影像和极简病史，给出影像学研判。病变位置： 右股骨 患者信息：女性 12岁 右膝疼痛1月。要求：影像学描述：客观描述你观察到的核心影像学特征。结合病史与影像，按可能性从大到小，列出最可能的 3 个具体疾病诊断。临床建议：给出下一步的具体诊疗或处置建议。

首先，我需要仔细分析用户提供的所有影像资料和病史信息。患者是12岁女性，主诉右膝疼痛1个月，病变位置在右股骨。

从X线片开始看：第一张图显示右股骨远端有明显的骨质破坏区域，边界不清，周围可见骨膜反应（“Codman三角”），提示侵袭性病变。第二张X线片是侧位像，同样见股骨远端后方的骨质溶骨性改变伴软组织肿块。

接下来是MRI：第三张图显示T1加权序列中病灶呈低信号，T2高信号（右图），且可见明显的软组织侵犯。特别注意到病灶内有不规则分叶状结构，这很可能是肿瘤的特征。

CT扫描部分（第四、五张图）更清晰地展示了骨质破坏的程度：股骨远端干骺端可见大面积溶骨性破坏，同时伴有骨皮质中断和软组织肿块。CT重建图像显示病变向周围侵袭，并有不规则的钙化或骨化点。

结合患者年龄（12岁女性）和临床表现（单侧膝关节疼痛），我需要回忆常见的骨肿瘤类型：

- 骨肉瘤：好发于10-25岁青少年，干骺端最常见。典型X线表现为"日光放射状"骨膜反应、Codman三角，MRI可见软组织肿块。
- 尤文肉瘤：更年轻（5-15岁），但骨质破坏常呈"洋葱皮"样改变，本例不明显。
- 骨巨细胞瘤：多见于20-40岁，此处年龄不符。

再仔细看影像特征：

1. X线可见典型的Codman三角和放射状骨膜反应（第一张图），这是骨肉瘤的典型征象
2. CT显示溶骨性破坏伴骨皮质中断（第四、五张图）
3. MRI显示软组织肿块（第三张图）且T2高信号

排除其他可能性：

- 骨转移癌：12岁儿童罕见，除非有原发肿瘤病史
- 慢性骨髓炎：通常有感染史，X线可见死骨，但本例无此表现

因此最可能的诊断排序应为：

1. 骨肉瘤（高概率）- 年龄、部位、影像特征完全符合
2. 尤文肉瘤（中等概率）- 但缺乏典型"洋葱皮"征象
3. 动脉瘤样骨囊肿（低概率）- 常为膨胀性病变，本例表现为侵袭性破坏

临床建议要具体：

- 紧急转诊至肿瘤科
- 必须做活检确认病理
- 治疗前需完善全身检查（PET-CT等）
- 由于是恶性肿瘤，需要多学科会诊

最后再检查一遍：所有影像都指向侵袭性骨肿瘤，特别是X线的Codman三角和MRI软组织肿块，12岁女孩膝关节疼痛，90%以上可能是骨肉瘤。必须强调活检的重要性。

**影像学描述**

**核心影像特征（基于多模态影像）：**

- **X线片：** 右股骨远端干骺端见不规则溶骨性破坏区，边界不清；局部骨皮质中断伴"Codman三角"征象（第一、二张图），提示侵袭性病变。
- **MRI：** T1WI示病灶呈低信号，T2WI高信号（第三张图）；股骨远端可见软组织肿块，向周围肌肉间隙侵犯，内部见不规则分叶状结构，伴骨髓水肿。
- **CT：** 股骨远端干骺端溶骨性破坏伴骨皮质中断（第四、五张图），病灶内见斑片状钙化/骨化影；软组织肿块与周围肌间隙界限不清，提示侵袭性生长。

**诊断分析（按可能性排序）**

1. **骨肉瘤**（高度可能）：12岁青少年女性，好发于股骨远端干骺端；X线" Codman三角"、CT溶骨破坏伴钙化、MRI软组织肿块及骨髓侵犯均符合典型表现。
2. **尤文肉瘤**（中度可能）：年龄相符，但缺乏"洋葱皮样"骨膜反应（本例以Codman三角为主），需结合病理鉴别。
3. **动脉瘤样骨囊肿继发恶性变**（低度可能）：原发病灶为膨胀性病变，但本例表现为侵袭性溶骨破坏且无典型液-液平面，可能性较低。

**临床建议**

1. **紧急活检**：立即行穿刺活检或切开活检明确病理分型（避免误诊），需注意术前评估血供及软组织侵犯范围。
2. **多学科会诊**：建议肿瘤科、骨科联合制定治疗方案，完善全身PET-CT排除转移灶。
3. **后续处置**：若确诊恶性肿瘤，应启动新辅助化疗+手术（保肢或截肢）的综合治疗；需警惕病理性骨折风险，术前可予外固定支架保护。

注：本例为典型青少年骨肿瘤表现，影像特征高度指向骨肉瘤，但最终诊断必须依赖病理结果。
