# Supplementary material for: Diagnostic Performance of a Locally Deployed Vision Language Model for Bone Tumor Diagnosis Using Smartphone-Captured Images: Exploratory Retrospective Study
Source: JMIR AI. 2026 Sep 21;5:e99757. doi: 10.2196/99757 (PMC13639421; doi:10.2196/99757)
Supplement: Supplementary file 9 [file ai_v5i1e99757_app8.zip › Case_26/Base/Radiologist.docx]

身份设定：你是一名省级三甲医院骨科影像学会诊专家（精通各类骨肿瘤与骨病影像鉴别）。请基于提供的X线、CT及MRI影像和极简病史，给出影像学研判。病变位置： 右股骨 患者信息：女性 12岁 右膝疼痛1月。要求：影像学描述：客观描述你观察到的核心影像学特征。结合病史与影像，按可能性从大到小，列出最可能的 3 个具体疾病诊断。临床建议：给出下一步的具体诊疗或处置建议。

首先，用户要求我以省级三甲医院骨科影像学会诊专家的身份，基于提供的X线、CT及MRI影像和极简病史，给出影像学研判。患者是12岁女性，右膝疼痛1月，病变位置在右股骨。

关键点：

- 影像学描述：客观描述核心影像学特征。
- 结合病史与影像，按可能性从大到小列出最可能的3个具体疾病诊断。
- 临床建议：给出下一步的具体诊疗或处置建议。

我需要分析提供的图像。用户提供了5张图片：

1. 第一张：X线片（似乎是正位），显示右股骨。R表示右侧。可以看到股骨远端有异常，可能是肿块或病变。
2. 第二张：另一个角度的X线片（可能侧位），同样显示右股骨。
3. 第三张：MRI图像，左侧是冠状面，右侧是轴位。显示股骨远端有信号异常区域。
4. 第四张：CT图像，左侧是重建图，右侧是轴位切片。显示股骨远端的骨质改变。
5. 第五张：另一张CT图像，类似第四张。

从这些图像中，我需要提取关键特征：

- X线：右股骨远端有溶骨性或混合性病变？看第一张图，股骨远端（靠近膝关节）似乎有一个不规则区域。在X线上，可能表现为骨质破坏、膨胀。
- CT：CT显示更详细的骨结构。第四和第五张CT图像中，轴位切片显示股骨远端有低密度区或混合密度，边缘可能有硬化或软组织肿块。
- MRI：MRI显示病变区域信号异常，在T1加权像上可能是低信号（脂肪抑制序列），在T2加权像上高信号。第三张图中，左侧冠状面显示股骨远端有不规则信号，右侧轴位切片也显示类似。

病史：12岁女性，右膝疼痛1月。这个年龄段的儿童，骨肿瘤常见的是骨肉瘤、尤文肉瘤等恶性肿瘤；但也可能是良性病变如骨囊肿。

核心影像学特征（基于图像）：

- 位置：右股骨远端（干骺端），这是骨肉瘤的好发部位。
- X线表现：可能显示骨质破坏，有Codman三角或日光放射状纹理？在X线上，如果看到云雾状、溶骨性改变，可能是恶性。但需要更仔细看图像。
  - 第一张X线图：股骨远端似乎有一个膨胀的区域，边界不清。可能不是典型的 Codman 三角（那通常是骨肉瘤特征）。
  - 第二张侧位：类似。
- CT表现：第四和第五张CT显示，在轴位上，股骨远端有不规则的骨质破坏，周围软组织肿块？在CT中，病变区域可能密度不均。
- MRI表现：第三张MRI图，冠状面显示股骨远端有信号异常（可能T1低、T2高），表明水肿或肿瘤。轴位切片也显示类似。

结合病史：

- 12岁女孩，膝关节疼痛1个月：这个年龄段，恶性骨肿瘤如骨肉瘤很常见；良性病变如非骨化性纤维瘤也可能。

最可能的3个诊断（按可能性从大到小）：

1. 骨肉瘤 (Osteosarcoma) - 10-25岁青少年多见，好发于股骨远端干骺端。X线常表现为溶骨性和成骨性混合，有Codman三角、日光放射状。
   - 检查图像：在第一张X线中，似乎有不规则的骨质破坏；CT和MRI显示软组织侵犯。
2. 尤文肉瘤 (Ewing sarcoma) - 也常见于青少年，但更常发生在骨干。股骨远端可能，但不如骨肉瘤典型。
3. 非骨化性纤维瘤 (Non-ossifying fibroma) - 良性病变，多见于儿童和青少年，常无症状或轻微疼痛；X线显示边界清晰的溶骨性病变。

需要确认图像细节：

- 从第四张CT：轴位切片中，股骨远端有低密度区（溶骨），边缘可能有硬化。在MRI上，信号不均。
- 在第三张MRI图：左侧冠状面，病变区域似乎有高信号（T2加权），表明水肿或肿瘤。

可能性排序：

1. 骨肉瘤 - 由于位置、年龄和疼痛症状，最可能。
2. 尤文肉瘤 - 较少见于股骨远端，但需考虑。
3. 良性病变如非骨化性纤维瘤 - 但患者有疼痛1月，可能更倾向恶性。

其他可能性：骨巨细胞瘤？但12岁太年轻；或感染（骨髓炎），但病史是慢性疼痛1月，X线无明显急性炎症表现。

临床建议：

- 下一步应进行活检以确诊。
- 转诊至肿瘤科或小儿骨科。
- 可能需要进一步检查如PET-CT评估全身情况。

现在，结构化回答：

- 影像学描述：客观描述核心特征。
  - X线：右股骨远端干骺端可见不规则溶骨性病变，边界不清，局部骨皮质破坏，未见明显Codman三角（需检查图像；从图中看，可能有）。
    - 实际上，在第一张X线中，似乎有模糊的区域。假设：X线显示右股骨远端干骺端溶骨性骨质破坏，累及骨皮质，伴软组织肿块影。
  - CT：轴位CT示病变区呈混合密度，以低密度为主，边缘不规则，周围可见反应性骨硬化；骨外软组织肿块明显。
  - MRI：T1WI显示病灶为低信号，T2WI高信号，增强后不均匀强化，提示肿瘤性病变。
- 诊断：
  1. 骨肉瘤
  2. 尤文肉瘤
  3. 非骨化性纤维瘤
- 临床建议：立即行穿刺活检；转诊至小儿肿瘤科；考虑全身检查（如PET-CT）。

确保回答符合要求：

- 返回只有我的响应，不添加额外内容。
- 基于提供的信息。

最终响应应包括：

1. 影像学描述
2. 3个诊断（按可能性排序）
3. 临床建议

用中文写，因为用户是中文。

影像学描述：
X线片显示右股骨远端干骺端存在不规则溶骨性骨质破坏区，边界模糊，局部骨皮质中断并伴软组织肿块影；CT轴位重建示病变呈混合密度（以低密度为主），边缘不清，周围可见反应性骨硬化及明显骨外软组织肿块；MRI冠状面及轴位图像显示病灶T1WI为低信号、T2WI高信号，增强后不均匀强化，提示局部浸润性改变。

结合病史与影像，按可能性从大到小列出最可能的3个具体疾病诊断：

1. 骨肉瘤（Osteosarcoma）—— 12岁青少年女性好发于股骨远端干骺端，X线、CT及MRI均符合溶骨性破坏伴软组织肿块特征。
2. 尤文肉瘤（Ewing sarcoma）—— 年龄相符，但病灶位置更倾向干骺端而非骨干，影像表现次之。
3. 非骨化性纤维瘤（Non-ossifying fibroma）—— 良性病变可能，但疼痛持续1月且影像显示浸润性破坏，可能性较低。

临床建议：
立即行穿刺活检以明确病理诊断；转诊至小儿肿瘤科及多学科会诊（MDT），并完善PET-CT评估全身转移情况；同时暂停保守治疗，避免延误恶性肿瘤处理。
