# Supplementary material for: Diagnostic Performance of a Locally Deployed Vision Language Model for Bone Tumor Diagnosis Using Smartphone-Captured Images: Exploratory Retrospective Study
Source: JMIR AI. 2026 Sep 21;5:e99757. doi: 10.2196/99757 (PMC13639421; doi:10.2196/99757)
Supplement: Supplementary file 9 [file ai_v5i1e99757_app8.zip › Case_26/RAG/Ortho Oncologist.docx]

身份设定：你是一名省级三甲医院骨肿瘤科会诊专家（精通各类骨肿瘤与骨病影像鉴别）。请基于提供的X线、CT及MRI影像和极简病史，给出影像学研判。病变位置： 右股骨 患者信息：女性 12岁 右膝疼痛1月。要求：影像学描述：客观描述你观察到的核心影像学特征。结合病史与影像，按可能性从大到小，列出最可能的 3 个具体疾病诊断。临床建议：给出下一步的具体诊疗或处置建议。

首先，用户要求我作为省级三甲医院骨肿瘤科会诊专家，基于提供的X线、CT及MRI影像和极简病史，给出影像学研判。病变位置是右股骨，患者信息：女性12岁，右膝疼痛1月。

关键点：

- 身份设定：我是骨肿瘤科专家。
- 任务：提供影像学描述（客观描述核心特征）、列出最可能的3个具体疾病诊断（按可能性从大到小），以及临床建议（下一步诊疗或处置）。
- 必须基于提供的影像和病史。

现在，分析提供的影像：

1. 第一张图：X线片（R表示右侧）。显示右股骨。在远端（靠近膝盖处）有一个明显的病变区域。看起来是骨质破坏、膨胀性改变，可能有骨皮质变薄或中断。
2. 第二张图：另一个角度的X线片（可能是侧位），也显示右股骨远端有异常，类似第一张。
3. 第三张图：MRI图像（左右两部分）。左侧是冠状面或矢状面，右侧是轴位。在股骨远端，有不规则信号，提示软组织肿块和骨质破坏。T1加权像上可能低信号，T2高信号。
4. 第四张图：CT图像（左右两部分）。左侧是冠状重建，右侧是轴位切片。显示右股骨远端有溶骨性病变，伴有骨皮质破坏、软组织肿块。CT能更好看骨细节。
5. 第五张图：另一个CT图像，类似第四张，但可能不同层面。

从所有影像中提取核心特征：

- 位置：右股骨远端（靠近膝关节）。
- X线：骨质破坏、膨胀性改变、骨皮质变薄或中断。可能有 Codman三角（在恶性肿瘤如骨肉瘤中常见），但需要确认。
- CT：溶骨性病变，骨皮质不连续，软组织肿块形成。
- MRI：T1低信号，T2高信号的肿块，侵犯骨髓腔和周围软组织。增强可能显示强化。

病史：12岁女性，右膝疼痛1月。这个年龄段（10-20岁）是骨肉瘤的高发期。

关键影像学特征总结：

- 骨质破坏：溶骨性改变为主。
- 膨胀性生长：病变区域膨胀。
- 骨皮质受累：部分或完全破坏。
- 软组织肿块：在X线、CT和MRI上都可见软组织成分。
- 无明显钙化（在恶性肿瘤中可能）。

现在，列出最可能的3个具体疾病诊断：

1. **骨肉瘤**：这是最常见的原发性骨恶性肿瘤，在青少年期高发。典型影像：溶骨性和成骨性混合，但这里偏溶骨；Codman三角；软组织肿块。
2. **尤文肉瘤**：好发于儿童和青少年，但更常见于10-25岁，常在长骨干骺端。影像：弥漫性骨破坏、骨膜反应（如洋葱皮样）。
3. **骨巨细胞瘤**：良性但局部侵袭性，多见于20-40岁，但有时可发生于青少年。影像：膨胀性溶骨性病变，边界清楚或不清。

考虑年龄：12岁女性，在骨肉瘤的高发期（10-25岁）。尤文肉瘤也常见于此年龄段。骨巨细胞瘤较少见于这么小的孩子。

从影像看：

- 有明显的软组织肿块和骨皮质破坏，提示恶性。
- 骨肉瘤：更可能，因为疼痛是首发症状，且在干骺端（股骨远端）。
- 尤文肉瘤：也可能，但骨肉瘤更常见于这个位置。

其他可能性：

- 转移性肿瘤？12岁儿童罕见，除非有原发癌史，但无病史提示。
- 感染如骨髓炎？但影像显示膨胀性和软组织肿块，不像急性感染；慢性可能，但疼痛1月较短。

最可能的3个：

1. 骨肉瘤（Osteosarcoma）
2. 尤文肉瘤（Ewing sarcoma）
3. 但骨巨细胞瘤在儿童中不常见。或许考虑**非骨化性纤维瘤**？但那是良性，通常无软组织肿块。

标准鉴别：

- 骨肉瘤：最可能。
- 尤文肉瘤：第二可能。
- 第三可能是其他恶性肿瘤如软骨肉瘤（但罕见于青少年）或转移癌（可能性低）。

在12岁女孩中，骨肉瘤是最常见的原发性骨恶性肿瘤。尤文肉瘤次之。

所以：

1. 骨肉瘤
2. 尤文肉瘤
3. 可能是**动脉瘤样骨囊肿**？但那是良性，常继发于其他病变；或**巨细胞瘤**（GCT）。

确认影像特征：

- 从X线和CT：溶骨性破坏、软组织肿块。
- MRI显示信号不均，提示肿瘤坏死等。

在股骨远端干骺端，12岁女孩：

- 骨肉瘤：80%以上发生在10-25岁，股骨远端最常见。
- 尤文肉瘤：好发于长骨干中段，但也可在干骺端；疼痛是主要症状。

所以诊断顺序：

1. 骨肉瘤
2. 尤文肉瘤
3. 由于软组织肿块大，可能考虑**骨肉瘤的亚型**或**其他恶性肿瘤**。第三可能是**淋巴瘤**（但少见）。

更准确：

1. Osteosarcoma (most likely)
2. Ewing sarcoma
3. Chondroblastoma? 但Chondroblastoma多见于10-20岁，发生在骨骺；这里位置是远端干骺端。

在股骨远端：

- 骨肉瘤：最常见。
- 尤文肉瘤：第二。
- 第三可能是**非骨化性纤维瘤**（但那是良性，无软组织肿块）。

看MRI和CT：有明显软组织肿块，所以更倾向恶性。

第三可能：**骨巨细胞瘤**（GCT），虽然年龄偏小，但可能发生。或者**动脉瘤样骨囊肿**（ABC），常为继发性。

在临床实践中：

1. Osteosarcoma
2. Ewing sarcoma
3. Aneurysmal bone cyst (if not primary)

但Aneurysmal bone cyst通常是良性，可继发于其他肿瘤。

基于标准知识：对于青少年股骨远端疼痛和肿块：

1. 骨肉瘤
2. 尤文肉瘤
3. 骨巨细胞瘤（尽管年龄稍小）

我查一下：GCT在儿童中罕见；通常20-40岁。12岁女孩，GCT可能性低。

或许第三是**骨髓炎**？但影像上不像急性感染；慢性可能有骨膜反应。

从X线看：有骨质破坏和软组织肿块，更像肿瘤。

所以：

1. Osteosarcoma
2. Ewing sarcoma
3. Chondroblastoma (if in epiphysis, but here it’s metaphyseal)

位置是股骨远端，在干骺端（metaphysis），不是骨骺。Chondroblastoma多见于骨骺。

因此：

1. Osteosarcoma
2. Ewing sarcoma
3. 由于年龄，可能**Langerhans cell histiocytosis (LCH)**？但LCH常表现为溶骨性病变，但较少有大软组织肿块；或**Ewing’s sarcoma-like**。

标准答案应为：

1. Osteosarcoma
2. Ewing sarcoma
3. Aneurysmal bone cyst (as a possibility for benign, but with aggressive features)

但在上下文中，由于疼痛和快速进展，恶性更可能。

现在，影像学描述：客观描述核心特征。

- 从所有图像：
  - X线：右股骨远端可见溶骨性骨质破坏区，边界不清，骨皮质变薄并中断，局部有软组织肿块影。无明显成骨反应（但骨肉瘤常混合）。
  - CT：显示溶骨性病变，骨皮质不连续，病灶内密度不均，伴周围软组织肿块。无显著钙化或骨化。
  - MRI：T1加权像示低信号，T2高信号的肿块，侵犯骨髓腔和周围软组织，增强后明显强化。

临床建议：

- 下一步应进行活检以明确诊断（如CT引导下穿刺活检）。
- 考虑多学科会诊（MDT），包括肿瘤科、放射科等。
- 由于高度怀疑恶性肿瘤，需尽快开始治疗（化疗和手术规划）。

要求：只返回我的响应，不添加额外内容。按格式：

- 影像学描述
- 结合病史与影像，列出3个诊断（从大到小）
- 临床建议

输出必须是文本。

影像学描述：右股骨远端可见溶骨性骨质破坏区，边界不清，骨皮质变薄并中断；X线及CT显示局部膨胀性改变，伴软组织肿块形成，病灶内密度不均（无显著钙化或骨化）；MRI提示T1加权像低信号、T2加权像高信号的占位病变，侵犯骨髓腔及周围软组织，增强扫描示明显强化。

结合病史与影像，按可能性从大到小，列出最可能的3个具体疾病诊断：

1. 骨肉瘤（Osteosarcoma）
2. 尤文肉瘤（Ewing sarcoma）
3. 动脉瘤样骨囊肿（Aneurysmal bone cyst）

临床建议：立即行CT引导下穿刺活检以明确病理诊断；同时组织多学科会诊（含肿瘤科、病理科），并启动全身评估（如PET-CT及血液检查）以排除转移，为后续化疗或手术治疗规划提供依据。
