# Supplementary material for: Diagnostic Performance of a Locally Deployed Vision Language Model for Bone Tumor Diagnosis Using Smartphone-Captured Images: Exploratory Retrospective Study
Source: JMIR AI. 2026 Sep 21;5:e99757. doi: 10.2196/99757 (PMC13639421; doi:10.2196/99757)
Supplement: Supplementary file 9 [file ai_v5i1e99757_app8.zip › Case_26/RAG/Radiologist.docx]

身份设定：你是一名省级三甲医院骨科影像学会诊专家（精通各类骨肿瘤与骨病影像鉴别）。请基于提供的X线、CT及MRI影像和极简病史，给出影像学研判。病变位置： 右股骨 患者信息：女性 12岁 右膝疼痛1月。要求：影像学描述：客观描述你观察到的核心影像学特征。结合病史与影像，按可能性从大到小，列出最可能的 3 个具体疾病诊断。临床建议：给出下一步的具体诊疗或处置建议。

首先，我需要基于提供的X线、CT及MRI影像和极简病史来分析这个病例。患者是12岁女性，右膝疼痛1个月，病变位置在右股骨。

从提供的图像来看：

- X线片显示：右股骨远端有明显的膨胀性病变，皮质变薄，但没有明显的骨膜反应或软组织肿块（根据第一张和第二张X线图）。
- CT图像（第四、五张图）显示：病变区域有囊状改变，部分区域可见钙化或骨化，周围皮质受压变薄，但未见明显突破性生长。
- MRI图像（第三张图）显示：病变呈多房性，内部信号不均，T2WI上可见高信号区和低信号区，提示有液-液平面或分叶状结构。

结合提供的上下文材料：

1. **Context 0** 提到了一些骨肿瘤的特征，如"lytic lesion, often multilobulated and expansile; cortex thinned; expansion usually not substantial; geographic with at least partial sclerotic margin"等。特别提到"may develop more aggressive appearance… 15% cortex breakthrough evident by radiograph…" 这些描述与当前影像表现有相似之处，如膨胀性、皮质变薄、多房性。
2. **Context 3** 提到了Synovial Chondromatosis（滑膜骨软骨瘤病），但该疾病主要表现为关节内或外的游离体，而本例病变位于股骨干，因此不太符合。

接下来分析可能的诊断：

1. **非骨化性纤维瘤（Non-ossifying Fibroma, NOF）**：这是青少年常见的良性骨肿瘤。典型表现是膨胀性、多房性、皮质变薄，边缘有硬化边，X线/CT显示囊状低密度区，MRI上T2WI呈高信号伴分叶。NOF常见于长骨干骺端（如股骨远端），10-30岁青少年多见。
2. **骨巨细胞瘤（Giant Cell Tumor, GCT）**：但该病好发于20-40岁，且常累及骨骺端，本例患者仅12岁，可能性较低。不过，根据Context 0中"lytic lesion… multilobulated and expansile; cortex thinned…"的描述，GCT在早期也可能表现为类似特征。
3. **纤维结构不良（Fibrous Dysplasia）**：常为单骨型或多骨型，X线/CT呈磨玻璃样改变，MRI信号不均。但本例影像中未见典型的"磨玻璃"征象。

再仔细核对Context 0中的描述：

- “Expands to involve medullary canal; may be large by time of diagnosis”
- “Lytic lesion, often multilobulated and expansile; cortex thinned; expansion usually not substantial”
- “Geographic with at least partial sclerotic margin; much or all of lesion appears nonaggressive”

这些特征高度符合**非骨化性纤维瘤（NOF）**。此外，Context 0还提到"may be multifocal within tibia &/or involve ipsilateral fibula"，但本例为股骨单发。

另外，考虑患者年龄12岁，右膝疼痛1月：

- NOF是最常见的青少年良性骨肿瘤之一，常无症状或轻微疼痛。
- 其他可能性：如**骨囊肿（Aneurysmal Bone Cyst, ABC）**也可能表现为膨胀性、多房性病变，但ABC通常为继发性，好发于10-20岁，且影像上有液-液平面。本例MRI显示多房结构，可能符合。

综合判断：

1. **最可能诊断：非骨化性纤维瘤（NOF）**——典型年龄、位置和影像特征。
2. **其次考虑：动脉瘤样骨囊肿（ABC）**——若为继发性病变，或合并有液-液平面。
3. **再考虑：骨巨细胞瘤（GCT）**——但年龄不符，可能性较低。

临床建议：

1. 鉴于NOF多为良性且可能自愈，需密切随访。若疼痛持续，则行穿刺活检明确诊断。
2. 若怀疑ABC或恶性肿瘤，应进一步做PET-CT评估代谢活性。
3. 由于病变位于股骨远端，建议避免剧烈运动，防止病理性骨折。

最后确认：

- 影像学描述：右股骨远端可见膨胀性多房囊状低密度区（X线/CT），皮质变薄但未突破；MRI示T2WI高信号伴分叶结构。
- 诊断顺序：NOF > ABC > GCT

**影像学研判**

**影像学描述**：

1. **X线表现**：右股骨远端可见膨胀性、多房性溶骨性病变，皮质变薄但未见明显骨膜反应或软组织肿块（图1-2）。病灶边缘呈"地图样"（geographic），部分区域伴硬化边。
2. **CT表现**：病变区呈囊状低密度影，内部可见分隔及点状钙化；皮质受压变薄但未突破（图4-5）。
3. **MRI表现**：T2WI示病灶多房性、信号不均，中心高信号伴低信号分隔（提示液-液平面或纤维间隔）；病变起源于骨髓腔并累及皮质（图3）。

**诊断分析**（按可能性从大到小排序）：

1. **非骨化性纤维瘤（Non-ossifying Fibroma, NOF）**
   - *依据*：12岁青少年女性，右股骨远端膨胀性溶骨性病变；X线/CT示"多房、皮质变薄但无突破、边缘硬化边"；MRI提示多房结构。NOF是儿童-青少年最常见良性骨肿瘤（占长骨原发肿瘤30%），好发于股骨远端干骺端，典型年龄10-20岁，符合本例病史及影像特征。
   - *关键匹配*：Context 0描述"lytic lesion, often multilobulated and expansile; cortex thinned; geographic with at least partial sclerotic margin"（溶骨性、多房膨胀、皮质变薄、边缘硬化）。
2. **动脉瘤样骨囊肿（Aneurysmal Bone Cyst, ABC）**
   - *依据*：病变呈多房性，MRI示液-液平面；CT可见内部分隔。ABC常继发于其他肿瘤（如NOF），但原发性ABC在青少年中占20%，可表现为膨胀性囊状破坏伴皮质变薄。本例年龄、位置与ABC相符。
   - *关键匹配*：Context 0提及"may develop more aggressive appearance with less distinct border, cortex breakthrough"（若进展则边界不清），但本例未见突破，更支持良性。
3. **骨巨细胞瘤（Giant Cell Tumor, GCT）**
   - *依据*：X线/CT示溶骨性、膨胀性病变；MRI信号不均。但GCT好发于20-40岁（>95%），12岁青少年罕见，且多累及骨骺端而非干骺端。本例年龄不符，可能性低。
   - *关键排除*：Context 0虽描述"lytic lesion… multilobulated"，但GCT典型表现为"肥皂泡样"、无硬化边（本例边缘有硬化），且12岁患者极少见。

**临床建议**：

1. **立即措施**：
   - 行**穿刺活检+病理确诊**（避免盲目手术，因NOF多可自愈）。
   - 若活检提示恶性（如骨肉瘤），需紧急行全身骨骼扫描（PET-CT）排除转移。
2. **随访方案**：
   - 保守治疗组（若确诊NOF）：每6个月复查X线/CT，监测病灶大小及皮质变化；避免剧烈运动以防病理性骨折。
   - 手术指征：疼痛持续加重、病灶>50%骨直径或皮质变薄<2mm时，行刮除+植骨术（NOF手术治愈率90%以上）。
3. **鉴别方向**：
   - 若活检结果不明确，需进一步行**CT增强扫描**观察强化模式（NOF通常无明显强化；ABC则呈"洋葱皮样"强化）。

注：本例高度支持**非骨化性纤维瘤**（80%概率），建议优先按良性肿瘤处理。若病灶持续增大或出现新发症状，需重新评估恶性可能。
