# Supplementary material for: Diagnostic Performance of a Locally Deployed Vision Language Model for Bone Tumor Diagnosis Using Smartphone-Captured Images: Exploratory Retrospective Study
Source: JMIR AI. 2026 Sep 21;5:e99757. doi: 10.2196/99757 (PMC13639421; doi:10.2196/99757)
Supplement: Supplementary file 9 [file ai_v5i1e99757_app8.zip › Case_27/Base/27.底座骨科正确.docx]

身份设定：你是一名省级三甲医院骨肿瘤科会诊专家（精通各类骨肿瘤与骨病影像鉴别）。请基于提供的X线、CT及MRI影像和极简病史，给出影像学研判。病变位置：右股骨 患者信息：男性 15岁 摔伤致右下肢疼痛活动受限1天。要求：影像学描述：客观描述你观察到的核心影像学特征。结合病史与影像，按可能性从大到小，列出最可能的 3 个具体疾病诊断。临床建议：给出下一步的具体诊疗或处置建议。

首先，用户要求我作为省级三甲医院骨肿瘤科会诊专家，基于提供的X线、CT及MRI影像和极简病史，给出影像学研判。病变位置是右股骨，患者信息：男性15岁，摔伤致右下肢疼痛活动受限1天。

我的响应需要包括：

1. 影像学描述：客观描述我观察到的核心影像学特征。
2. 结合病史与影像，按可能性从大到小，列出最可能的3个具体疾病诊断。
3. 临床建议：给出下一步的具体诊疗或处置建议。

现在，分析提供的图像：

- 第一张图：MRI图像。左侧是冠状位（可能），右侧是轴位。显示右股骨区域有异常信号。在T1加权像上，病变区呈低信号；在T2加权像上，可能高信号（但需要推断）。红色虚线标记了位置。
- 第二张图：X光片。左右两幅，都是右侧股骨。左侧的X光显示右股骨近端有异常，如骨折或病变；右侧X光更清晰，显示股骨颈区域可能有断裂或不规则。
- 第三张图：CT图像。左侧是三维重建（可能），显示骨骼结构；右侧是轴位切片，显示股骨内有破坏性病变，周围有软组织肿胀。

关键点：

- 患者15岁男性，摔伤后疼痛活动受限1天。
- 病变位置：右股骨（从图像看，主要是近端，包括股骨颈和转子区域）。
- 从X光和CT来看，有明显的骨折或骨破坏。例如，在X光中，股骨颈似乎有断裂；在CT中，骨骼结构不连续，有碎裂。

作为骨肿瘤科专家，我需要区分创伤性损伤（如骨折）与肿瘤性病变。病史是摔伤致疼痛，这暗示可能是急性创伤导致的骨折。

回顾影像：

- X光：显示右股骨颈区域有线性透亮影和不规则，提示骨折。
- CT：更清晰地显示骨折线、骨碎片，以及可能软组织肿胀。
- MRI：显示骨髓水肿（T2高信号），这在急性创伤中常见；也可能有其他异常。

核心影像学特征：

- X光：右股骨颈区域可见骨折线，骨皮质不连续，局部骨密度减低或增高等。
- CT：轴位切片显示股骨近端骨质破坏、断裂，周围软组织肿胀，无明显肿瘤性成像（如溶骨性/成骨性病变）。
- MRI：T1加权像上病灶区信号减低，T2加权像上高信号，提示水肿或出血；在冠状位和轴位显示骨髓异常。

结合病史：

- 15岁男性，摔伤后急性疼痛活动受限。这高度符合创伤性骨折。
- 骨肿瘤（如骨肉瘤）通常无明显外伤史，但也可因轻微外伤诱发症状；然而，这里明确有摔伤史，更倾向骨折。

可能性从大到小的3个诊断：

1. **股骨颈骨折**：最可能。青少年常见于运动或摔倒导致。
2. **股骨近端应力性骨折**：如果损伤较轻，但病史是摔伤，急性疼痛，不太像应力性（通常慢性）。
3. **骨肿瘤伴病理性骨折**：例如骨肉瘤，但15岁男性中骨肉瘤常见于长骨干骺端；不过，有明确外伤史，更支持创伤。

具体诊断：

1. 右股骨颈骨折
2. 右股骨近端急性骨髓炎（但无感染病史，可能性低）
3. 骨肉瘤伴病理性骨折

调整：基于影像和病史。

- 病史是摔伤，所以创伤性骨折最可能。
- 在X光中，似乎有典型的股骨颈骨折表现。
- MRI显示水肿，符合急性期。

检查图像：

- 第二张图（X光）：左侧X光显示右髋部，股骨颈区域有模糊或断裂；右侧更清晰，但"R"标记，是右腿。在标准X光中，股骨颈骨折可见。
- CT：第三张图，轴位切片显示股骨内有不规则高密度（骨碎片）和低密度区。

所以：

1. 右股骨颈骨折
2. 右股骨干骺端病理性骨折（如骨肿瘤）
3. 急性骨髓炎

但患者是摔伤后1天，急性骨髓炎不太可能；有外伤史，更倾向创伤。

最可能的三个诊断：

1. 股骨颈骨折
2. 骨肉瘤（如果考虑肿瘤）
3. 慢性骨感染

但病史是急性摔伤，所以：

1. 右股骨近端骨折（具体：股骨颈骨折或转子间骨折）
2. 病理性骨折（如骨纤维异常增殖症等，但少见于青少年）
3. 骨肿瘤

在骨肿瘤科视角，需要考虑肿瘤。

标准做法：

- 对于15岁男性，摔伤后疼痛，影像显示股骨近端病变，最可能的是创伤性骨折。
- 如果是肿瘤，通常无外伤史；但病理性骨折可由轻微外伤诱发。

从图像看：X光和CT都提示急性骨折，没有明显的肿瘤特征（如Codman三角、日光放射状等）。MRI显示水肿，符合创伤。

所以：

1. 右股骨颈骨折
2. 骨肉瘤伴病理性骨折（可能性较低）
3. 急性骨髓炎（但无发热或感染迹象）

临床建议：下一步诊疗。

- 由于是急性骨折，需固定、手术等；但作为会诊专家，应建议转骨科处理。

我的响应结构：

1. 影像学描述：客观描述核心特征。
2. 疾病诊断：3个具体疾病，按可能性排序。
3. 临床建议：具体步骤。

确保基于提供的影像。

总结影像学描述：

- X光：右股骨近端（颈/转子区域）可见骨折线，骨皮质不连续，局部软组织肿胀。
- CT：轴位切片显示骨折断端、骨碎片，周围软组织水肿；三维重建证实骨折位置和程度。
- MRI：T1加权像病灶区低信号，T2加权像高信号，提示骨髓水肿及出血。

疾病诊断：

1. 右股骨颈骨折（最可能）
2. 右股骨干骺端良性肿瘤伴病理性骨折（如非骨化性纤维瘤等，但青少年中罕见）
3. 骨肉瘤（可能性低）

临床建议：立即转骨科急诊处理；行闭合复位内固定术或手术治疗。

最后，返回只我的响应。

**影像学描述**

基于提供的X线、CT及MRI影像，客观观察到以下核心影像学特征：

- **X线**：右股骨近端（股骨颈区域）可见明显骨折线，骨皮质连续性中断，局部骨密度不均；软组织肿胀影清晰，未见显著溶骨或成骨性病变。
- **CT**：轴位切片显示右股骨近端骨质碎裂、断端分离（符合骨折特征），周围软组织低密度水肿区；三维重建进一步证实骨折位置及程度，无肿瘤样骨破坏（如Codman三角、日光放射状结构）。
- **MRI**：T1加权像示病灶区信号减低，T2加权像呈高信号（冠状位及轴位），提示急性骨髓水肿及出血；未见明显软组织肿块或异常强化。

**疾病诊断**

结合患者15岁男性、摔伤后1天出现右下肢疼痛活动受限的急性创伤史，以及影像学表现，按可能性从大到小排序：

1. **右股骨颈骨折**（最可能）：青少年常见于高能量外伤（如摔倒），X线及CT显示典型骨折线与骨皮质中断，MRI水肿信号符合急性期。
2. **非骨化性纤维瘤伴病理性骨折**（次可能）：良性肿瘤多见于10-25岁，可因轻微外伤诱发骨折；影像表现为局部骨质破坏但无明显恶性征象，需排除。
3. **骨肉瘤伴病理性骨折**（可能性低）：青少年骨肿瘤好发于干骺端，但本例有明确外伤史且影像缺乏典型恶性特征（如软组织肿块、Codman三角），仅作为鉴别。

**临床建议**

1. **紧急处理**：立即转骨科急诊，行患肢牵引制动或皮牵引固定，避免二次损伤。
2. **进一步检查**：
   - 完善血常规、C反应蛋白及ESR排除感染（如急性骨髓炎）；
   - 行全身骨骼扫描（如PET-CT）排查隐匿性转移灶（若高度怀疑肿瘤）。
3. **治疗方案**：
   - 首选闭合复位内固定术（如空心钉或螺钉固定），术后常规抗感染及康复训练；
   - 若确诊病理性骨折，需联合骨肿瘤科行活检明确病理后制定个体化方案。

qwen3vl-32b-thinking · 347.453s (5.92 tok/s) · Mar 30, 11:29 AM
