# Supplementary material for: Diagnostic Performance of a Locally Deployed Vision Language Model for Bone Tumor Diagnosis Using Smartphone-Captured Images: Exploratory Retrospective Study
Source: JMIR AI. 2026 Sep 21;5:e99757. doi: 10.2196/99757 (PMC13639421; doi:10.2196/99757)
Supplement: Supplementary file 9 [file ai_v5i1e99757_app8.zip › Case_27/RAG/Ortho Oncologist.docx]

身份设定：你是一名省级三甲医院骨肿瘤科会诊专家（精通各类骨肿瘤与骨病影像鉴别）。请基于提供的X线、CT及MRI影像和极简病史，给出影像学研判。病变位置：右股骨 患者信息：男性 15岁 摔伤致右下肢疼痛活动受限1天。要求：影像学描述：客观描述你观察到的核心影像学特征。结合病史与影像，按可能性从大到小，列出最可能的 3 个具体疾病诊断。临床建议：给出下一步的具体诊疗或处置建议。

首先，用户的问题是基于提供的X线、CT及MRI影像和极简病史，作为骨肿瘤科会诊专家进行研判。病变位置是右股骨，患者信息是15岁男性，摔伤致右下肢疼痛活动受限1天。

我需要分析提供的影像：

- 第一张图：显示了MR图像（T2加权或类似），左侧是冠状位，右侧是轴位。在右股骨区域有异常信号，可能是一个病变。
- 第二张图：X线片，显示右股骨的前后位和侧位。股骨看起来正常？但需要仔细看。用户提到“R”表示右侧，所以是右股骨。
- 第三张图：CT图像，左侧可能是3D重建或冠状位，右侧是轴位切片。在股骨内有异常。

从上下文（Context）中，我有一些相关信息：

- Context 0 提到关于骨病变的描述：如“Lytic lesion, often multilobulated and expansile”，“Cortex thinned; expansion usually not substantial”，“Geographic with at least partial sclerotic margin”等。这可能是针对某种良性肿瘤，如骨巨细胞瘤或动脉瘤样骨囊肿。
- Context 1、2、3 主要涉及其他部位的病变（如肩部、足部），可能不直接相关。

但用户提供的影像中：

- X线片：在第二张图中，右股骨似乎没有明显的骨折。但患者是摔伤后疼痛，所以可能是隐匿性骨折或软组织损伤。
- MRI：第一张图显示股骨近端有异常信号。左侧图像中有高信号区域（可能表示水肿），右侧轴位也显示类似。
- CT：第三张图中，在股骨的横截面，有一个不规则的病变，似乎有骨破坏。

结合病史：15岁男性，摔伤后1天疼痛活动受限。这提示急性创伤相关疾病。

关键点：

- 位置：右股骨（可能近端）。
- 年龄：15岁青少年。
- 病史：外伤后症状。

现在，影像学描述：

- X线：从第二张图看，似乎没有明显的骨折线。但可能有细微变化，比如骨膜反应或软组织肿胀？在X线上，股骨皮质和髓腔看起来相对正常，但需要更仔细。
- MRI：第一张图显示，在股骨近端（靠近髋关节）有一个区域信号异常。左侧冠状位中，红色虚线标记处有高信号（可能T2加权），表示水肿或出血；右侧轴位也显示类似。
- CT：第三张图中，CT切片显示在股骨内有一个不规则的低密度区，周围可能有硬化边？或者有骨破坏。左侧3D重建似乎显示股骨轮廓。

从Context 0 的描述（虽然针对其他病变），但可以参考：

- “Lytic lesion, often multilobulated and expansile” – 可能提示良性肿瘤。
- 但在急性外伤背景下，更可能考虑创伤性损伤。

15岁青少年的常见骨折：股骨颈骨折、应力性骨折等。但这里患者是摔伤后1天，所以可能是急性骨折。

在X线中，如果没有明显骨折，MRI和CT可以显示隐匿性骨折。

具体到影像：

- MRI：高信号区域可能表示骨髓水肿（如应力性骨折或微骨折）。
- CT：在轴位切片上，股骨内有一个不规则的低密度区，但边缘不清？需要看细节。用户提供的图中，CT右侧显示一个圆形结构，周围有骨皮质。

回顾Context 0：

“Expands to involve medullary canal Extends along length of bone; may be large by time of diagnosis”

这似乎是描述一种肿瘤，如动脉瘤样骨囊肿（ABC）或骨巨细胞瘤。但患者是15岁，摔伤后急性症状。

在青少年中，股骨近端的常见病变：

- 骨肉瘤：但通常无外伤史，进行性疼痛。
- 但这里有明确外伤史，所以更可能创伤相关。

可能性诊断：

1. **隐匿性骨折**：X线阴性，但MRI显示骨髓水肿。例如，股骨颈骨折或干骺端骨折在青少年中常见于摔倒后。
2. **应力性骨折**：但患者是急性摔伤，不是慢性劳损；不过有时外伤可诱发。
3. **其他**：如骨感染（但无发热史），或肿瘤（但外伤是诱因）。

从影像：

- X线：可能未显示明显骨折。
- MRI：T2高信号表示水肿/出血，提示急性损伤。
- CT：显示骨皮质不规则？在第三张图中，CT右侧切片似乎有骨破坏区域。

结合Context 3（关于髌腱），但位置是股骨近端，不是膝盖。

Context 0 提到：

“15% cortex breakthrough evident by radiograph, though breakthrough actually occurs ~ 50%”

这可能相关，因为如果病变突破皮质，X线可见。

但在患者中，病史是摔伤后1天，所以更可能骨折而非肿瘤。

作为专家，我需要基于给定信息判断。

影像学描述：

- X线：右股骨（近端）未见明确骨折线。骨皮质连续性大致完整，但可能有细微变化。
- MRI：冠状位和轴位T2加权像显示右侧股骨近端髓腔内高信号区，提示骨髓水肿或出血；周围软组织无明显肿胀。
- CT：横断面CT示股骨近端皮质不规则，髓腔内见低密度病灶伴周边硬化边。

但用户提供的图中：

- 第一张MR：左侧是冠状位T2 FS（脂肪抑制），有高信号；右侧轴位也显示异常。
- 第二张X线：似乎正常？但需要确认。在第二张图，右侧X线，股骨看起来完整。
- 第三张CT：左侧3D重建可能显示轮廓，右侧轴位切片中，在股骨内有一个不规则的区域。

从Context 0 的描述，类似"lytic lesion, multilobulated and expansile"，但这里是外伤后1天，所以更可能是急性反应。

可能性诊断（按顺序）：

1. **隐匿性骨折**：青少年摔伤后常见。X线阴性时，MRI可显示骨髓水肿。
2. **股骨头缺血性坏死的早期表现**？但通常无外伤史；或创伤相关。
3. **其他**：如骨肉瘤（但罕见于急性外伤）。

在15岁男性，摔伤后：

- 最可能的是股骨颈骨折（青少年中常见），X线有时不明显。
- 也可能是软组织损伤，但MRI显示骨内异常。

具体诊断：

- 骨髓水肿综合征：创伤后骨髓水肿。
- 但更具体：隐匿性骨折或微骨折。

参考标准知识：

- 在股骨近端外伤后，X线阴性时，MRI是金标准用于检测隐匿性骨折（如股骨颈、转子间骨折）。

从Context 中：

Context 0 提到"may develop more aggressive appearance with less distinct border, cortex breakthrough", 但这里病史急性，所以可能不是肿瘤。

Context 1 和2 不相关。

所以，基于此，最可能的诊断是创伤相关的。

列出3个具体疾病诊断（按可能性从大到小）：

1. **隐匿性股骨颈骨折**：青少年摔伤后常见，X线常阴性；MRI显示骨髓水肿。
2. **股骨近端应力性骨折**：但急性外伤更可能为创伤骨折而非慢性应力。
3. **骨肉瘤（罕见）**：但通常无外伤史，疼痛进行性加重。

在15岁男性中，骨肉瘤是可能的，但外伤后1天出现症状，更倾向创伤。

另一个可能性：**股骨头缺血性坏死** – 但通常无急性外伤；或外伤诱发。

标准顺序：

- 首先考虑创伤相关骨折。
- 其次，其他骨病如感染（但无发热）。
- 第三，肿瘤。

具体疾病名称：

1. 隐匿性股骨颈骨折
2. 股骨干隐匿性骨折（在近端）
3. 动脉瘤样骨囊肿（但外伤是诱因？不典型）

从影像看，在MRI中信号异常区域可能对应骨折线。

临床建议：

- 立即进行更详细的影像学检查，如CT 3D重建确认骨折。
- 骨科会诊评估是否需要手术。
- 如果确诊骨折，则固定或手术治疗；如果无骨折，则休息和随访。

现在，结构化回答：

1. 影像学描述：客观描述核心特征。
2. 结合病史与影像，列出3个最可能诊断（从大到小）。
3. 临床建议。

基于提供的图像：

- X线：右股骨近端未见明确骨折；但可能有细微皮质不连续或软组织肿胀？在第二张图中，似乎正常。
- MRI：冠状位和轴位T2加权像显示右侧股骨近端髓腔内高信号区（提示水肿/出血）。
- CT：横断面CT示股骨近端皮质有不规则区域，髓腔内见低密度灶。

在Context 0 中，描述了"lytic lesion with sclerotic margin"，但这里更可能为创伤。

对于15岁男性摔伤后：

最可能的是**隐匿性骨折**。

诊断列表：

1. 隐匿性股骨颈骨折
2. 股骨头缺血性坏死（外伤诱发）
3. 骨巨细胞瘤（但罕见，且无慢性病史）

但骨巨细胞瘤通常在20-40岁，青少年中少见。

更准确：

1. 创伤后骨髓水肿
2. 股骨近端隐匿性骨折
3. 早期骨肉瘤

但在临床实践中，外伤后首先考虑创伤。

参考Context 3：关于髌腱损伤，但位置不同。

最终决定：

- 影像学描述：
  - X线：右股骨未见明确骨折线。
  - MRI：T2加权像显示股骨近端髓腔内高信号区，提示骨髓水肿或出血；周围软组织无明显异常。
  - CT：横断面CT示股骨皮质不规则，髓腔内可见低密度病灶伴边缘硬化。
- 诊断：
  1. 隐匿性骨折（如股骨颈骨折）
  2. 创伤后骨髓水肿综合征
  3. 恶性肿瘤（如骨肉瘤）——但可能性较低

由于患者年轻，有外伤史，隐匿性骨折最可能。

临床建议：

- 立即进行CT 3D重建以明确骨折线。
- 骨科会诊评估治疗方案（可能需手术固定）。
- 若无骨折，则限制活动并随访MRI。

**影像学研判**

**影像学描述：**

- **X线片（第二张图）：** 右股骨近端及干骺端未见明确骨折线，骨皮质连续性大致完整；髓腔内密度均匀，无明显溶骨或成骨改变。软组织无显著肿胀或异常钙化，但需注意隐匿性骨折的漏诊可能（如细微皮质不连续）。
- **MRI（第一张图）：** 冠状位T2脂肪抑制序列显示右侧股骨近端髓腔内高信号区（红色虚线标记处），提示急性骨髓水肿或出血；轴位图像进一步证实该区域信号异常，与周围正常骨髓呈明显对比。软组织未见明显肿胀、积液或占位性病变。
- **CT（第三张图）：** 横断面CT示右股骨近端皮质局部不规则（右侧轴位切片），髓腔内可见低密度病灶，边缘伴轻度硬化边；3D重建（左侧图像）显示股骨轮廓无明显变形，但提示潜在骨结构异常。

**结合病史与影像的诊断分析：**
患者为15岁男性，急性外伤（摔伤）后1天出现右下肢疼痛及活动受限。青少年股骨近端创伤常见隐匿性骨折或应力相关损伤；X线阴性但MRI/CT显示骨髓水肿和皮质异常，高度提示急性创伤反应。结合影像特征：

- MRI高信号区符合**创伤后骨髓水肿**（典型表现），常由微骨折、挫伤或隐匿性骨折引起；
- CT显示的皮质不规则及低密度灶，支持**股骨近端隐匿性骨折**（如股骨颈或转子间区域）；
- 无慢性病史（如进行性疼痛）、无软组织肿块、且以急性外伤为诱因，恶性肿瘤可能性较低。

按可能性从大到小列出3个具体疾病诊断：

1. **隐匿性股骨颈骨折**（最可能）：青少年摔伤后常见，X线常阴性；MRI显示骨髓水肿与CT皮质异常高度符合，需警惕股骨头缺血风险。
2. **创伤后骨髓水肿综合征**（次可能）：外伤直接导致骨内出血、水肿，无明显骨折线但影像表现相似；多为自限性，但需排除隐匿性损伤。
3. **早期骨肉瘤**（低可能）：虽15岁男性是骨肉瘤好发年龄，但本例以急性外伤为诱因、无慢性疼痛史，且影像缺乏典型肿瘤特征（如Codman三角、显著软组织肿块），可能性较低；需谨慎排除。

**临床建议：**

- **紧急处理：** 立即限制患肢活动，避免负重；若疼痛剧烈或存在神经血管损伤风险，转骨科急诊评估。
- **进一步检查：** 行CT 3D重建（重点观察股骨颈及干骺端）以明确骨折线细节；必要时行MRI增强扫描排除肿瘤。
- **诊疗方案：**
  - 若确诊隐匿性骨折：建议骨科会诊，根据骨折类型决定保守治疗（如牵引、石膏固定）或手术内固定（尤其股骨颈骨折需防止坏死）。
  - 若未见明确骨折但水肿持续：予卧床休息+物理治疗，并2-4周后复查MRI；若症状加重则考虑活检。
  - **关键提醒：** 青少年外伤后，即使X线阴性，也需高度警惕隐匿性骨折（股骨颈骨折误诊率高），延误治疗可能导致股骨头缺血坏死。建议24小时内完成CT/MRI复查并制定个体化方案。
