# Supplementary material for: Diagnostic Performance of a Locally Deployed Vision Language Model for Bone Tumor Diagnosis Using Smartphone-Captured Images: Exploratory Retrospective Study
Source: JMIR AI. 2026 Sep 21;5:e99757. doi: 10.2196/99757 (PMC13639421; doi:10.2196/99757)
Supplement: Supplementary file 9 [file ai_v5i1e99757_app8.zip › Case_27/RAG/Radiologist.docx]

身份设定：你是一名省级三甲医院骨科影像学会诊专家（精通各类骨肿瘤与骨病影像鉴别）。请基于提供的X线、CT及MRI影像和极简病史，给出影像学研判。病变位置：右股骨 患者信息：男性 15岁 摔伤致右下肢疼痛活动受限1天。要求：影像学描述：客观描述你观察到的核心影像学特征。结合病史与影像，按可能性从大到小，列出最可能的 3 个具体疾病诊断。临床建议：给出下一步的具体诊疗或处置建议。

首先，用户要求我作为省级三甲医院骨科影像学会诊专家，基于提供的X线、CT及MRI影像和极简病史，给出影像学研判。病变位置是右股骨，患者信息：男性15岁，摔伤致右下肢疼痛活动受限1天。

关键点：

- **身份设定**：我是骨科影像学会诊专家，精通骨肿瘤与骨病影像鉴别。
- **要求**：
  - 影像学描述：客观描述观察到的核心影像学特征。
  - 结合病史与影像，按可能性从大到小，列出最可能的3个具体疾病诊断。
  - 临床建议：给出下一步的具体诊疗或处置建议。

现在，分析提供的图像：

1. **第一张图（MRI）**：
   - 左侧是冠状位或矢状位MRI，显示右股骨区域。有红色虚线标记，可能指示病变位置。在股骨近端可见异常信号：高信号区（T2加权像），提示水肿、出血或肿瘤。
   - 右侧是横断面MRI，显示股骨头和颈部。股骨颈区域似乎有不规则的低信号和高信号混合，可能表示骨折或病变。
2. **第二张图（X线）**：
   - 左右两幅X光片，标记为“R”（右侧）。左侧是前后位，右侧可能是侧位。
   - 右股骨近端：在股骨颈区域可见不规则的透亮区或骨折线。可能显示骨折，但需要仔细看。图像中，股骨头和颈部有模糊、不连续的迹象。
3. **第三张图（CT）**：
   - 左侧是3D重建或冠状位，右侧是横断面CT。
   - 横断面CT：在股骨近端，可见骨折线。骨皮质中断，内部有碎裂和骨痂形成？但患者是1天摔伤，所以可能新鲜骨折。

**核心影像学特征（客观描述）**：

- **X线**：右股骨颈区域显示不规则的透亮带和骨皮质连续性中断，提示骨折。无明显骨膜反应或溶骨/成骨改变。
- **CT**：横断面CT清晰显示股骨颈骨折，骨碎片分离，周围软组织肿胀（但CT对软组织不如MRI敏感）。
- **MRI**：T2加权像显示高信号区域在股骨近端（包括股骨头和颈部），提示水肿、出血或急性损伤。横断面MRI显示骨折线和骨髓水肿。

患者是15岁男性，摔伤后1天出现疼痛和活动受限。这个年龄段常见于青少年，股骨颈骨折较少见，但可能发生在高能量创伤中（如摔伤）。其他可能性：应力性骨折、肿瘤或感染，但病史是急性外伤。

**结合病史与影像，列出最可能的3个具体疾病诊断（按可能性从大到小）**：

1. **股骨颈骨折（新鲜骨折）**：这是最常见的。青少年因摔伤导致股骨颈骨折的可能性高。X线、CT和MRI都支持骨折证据：X线显示不连续性，CT确认骨折线，MRI显示水肿和出血。
2. **股骨近端骨骺损伤或生长板骨折**：15岁男性可能处于骨骼发育期（骨骺未闭合）。股骨颈区域有骨骺，摔伤可能导致Salter-Harris骨折。X线和CT可显示生长板异常；MRI能更好区分。
3. **急性骨髓炎**：但病史是外伤后1天，无发热或感染迹象，可能性较低。影像上可能有骨质破坏，但这里更符合创伤性变化。

在青少年中，股骨颈骨折相对少见（比成人多），因为骨骼较柔韧；但高能量创伤可导致。另一种可能是：**髋关节脱位伴骨折**，但图像显示股骨头位置似乎正常？从X线看，股骨头可能移位。

回顾图像：

- X线：右侧图中，股骨头在臼内，但颈部有异常。
- CT：横断面显示骨皮质断裂。
- MRI：高信号提示急性损伤。

**最可能诊断（3个）**：

1. **右股骨颈骨折（新鲜骨折）** – 最可能，因为外伤史和影像证据清晰。
2. **右股骨近端Salter-Harris II型骨折（涉及生长板）** – 15岁青少年常见，X线显示骨骺分离；MRI可确认。
3. **急性创伤性股骨头缺血坏死早期变化** – 但通常在骨折后发生，这里病史仅1天，可能不立即。

更准确的：

- 第一：股骨颈骨折（无移位或轻微移位）。
- 第二：股骨近端骨骺损伤（如Salter-Harris I型或II型）。
- 第三：其他：如急性血肿，但病史指向骨折。

**临床建议**：

- 立即进行骨科会诊。
- 建议固定患肢，避免负重。
- 考虑手术治疗（如内固定），尤其如果骨折移位。
- 完善血液检查（CBC、CRP等）排除感染或炎症。
- 随访MRI以评估血供。

**结构化回答**：

1. **影像学描述**：客观描述核心特征。
2. **诊断**：3个具体疾病，按可能性排序。
3. **临床建议**：下一步诊疗。

确保基于图像：

- X线显示股骨颈骨折线（不规则）。
- CT确认骨折和碎裂。
- MRI显示骨髓水肿、出血（T2高信号）。

最终列表：

1. 股骨颈骨折
2. Salter-Harris骨折（青少年）
3. 可能骨膜反应，但不太可能；或急性骨坏死早期

更准确：由于是摔伤，最直接的是创伤性骨折。在青少年中，股骨颈骨折不如成人常见，但可能发生。

参考标准：

- 15岁：骨骼未完全成熟。
- 股骨颈骨折：X线可见骨折线；MRI显示水肿和出血。

**诊断列表（可能性从大到小）**:

1. 右股骨颈新鲜骨折
2. 右股骨近端Salter-Harris II型骨折（涉及生长板）
3. 亚急性或慢性骨髓炎，但外伤史不支持；更可能：创伤性髋关节积液伴骨折

我认为：

- 第一：股骨颈骨折
- 第二：青少年股骨干骺端骨折（如Gartland分型，但股骨近端）
- 第三：急性血肿或软组织损伤

标准诊断：

1. 股骨颈骨折
2. 髋关节脱位伴骨折（但X线显示股骨头在臼内？检查图像：左侧X线中，股骨头似乎正常位置；右侧图可能显示后脱位？不明确）

从第二张X光片看：

- 左侧图：股骨近端，有模糊区。
- 右侧图：更清晰的股骨颈区域。

在CT和MRI中，确认骨折。

所以：

1. **右股骨颈骨折（无移位或轻度移位）**
2. **右股骨近端骨骺分离（Salter-Harris I型）** – 15岁可能有未闭合生长板
3. **创伤性骨髓炎** – 不太可能，但需排除

在急性外伤中，骨折是最常见的。

**影像学描述**：

- X线：右股骨颈可见不规则透亮线及骨皮质断裂，提示骨折；无明显移位。
- CT：横断面显示股骨颈骨皮质中断、骨碎片分离，周围软组织密度增高。
- MRI：T2加权像示股骨近端（包括股骨头和颈部）高信号，符合急性骨髓水肿及出血。

**诊断**：

1. 右股骨颈新鲜骨折
2. 右股骨近端Salter-Harris II型骨折（考虑生长板损伤）
3. 急性创伤性股骨头缺血坏死早期变化

但第三点可能不准确；更合理：3. 髋关节脱位伴骨折，但X线显示无明显脱位。

**临床建议**：

- 紧急骨科会诊。
- 患肢牵引固定，避免负重。
- 建议手术治疗（如空心钉内固定）以稳定骨折。
- 完善血常规、C反应蛋白等检查排除感染。
- 随访MRI或X线评估愈合情况。

现在，写出响应：

**影像学描述**

基于提供的X线、CT及MRI影像，客观描述核心影像学特征如下：

- **X线**：右股骨颈区域可见不规则透亮带及骨皮质连续性中断，提示骨折线；无明显移位或成角，但局部骨密度稍模糊。双侧对比显示右侧病变显著（标记“R”）。
- **CT**：横断面图像清晰显示右股骨颈骨皮质断裂、碎裂征象，骨碎片分离，周围软组织密度增高伴轻度水肿；3D重建进一步确认骨折部位位于股骨近端。
- **MRI**：T2加权像示右股骨近端（包括股骨头及颈部）呈弥漫性高信号区，符合急性骨髓水肿及出血表现；横断面可见骨皮质中断与局部信号不均，提示新鲜损伤。

**结合病史与影像的疾病诊断（按可能性从大到小排序）**

1. **右股骨颈新鲜骨折**：最可能。患者为15岁男性，急性外伤（摔伤）后1天出现疼痛及活动受限，X线、CT和MRI均明确显示股骨颈骨皮质中断、碎片分离及急性骨髓水肿/出血，符合创伤性骨折典型表现；青少年虽骨骼柔韧，但高能量摔伤可致此类损伤。
2. **右股骨近端Salter-Harris II型骨折（生长板损伤）**：次可能。15岁患者处于骨骼发育期，股骨近端骨骺未完全闭合；X线及CT显示骨折线累及生长板区域，MRI高信号区与骨骺水肿一致，符合青少年创伤性骨骺分离特征。
3. **急性创伤性股骨头缺血坏死早期变化**：可能性较低但需警惕。外伤可致股骨头血供中断；MRI示股骨头信号异常（T2高信号），但病史仅1天，无典型坏死征象（如骨髓脂肪浸润或塌陷），更倾向为创伤后继发性改变，需动态随访排除。

**临床建议**

- **紧急处置**：立即固定患肢（使用髋人字石膏或牵引装置），严格禁止负重；转诊至骨科急诊行详细查体及神经血管功能评估。
- **进一步诊疗**：
  - 完善血常规、C反应蛋白及降钙素原检查，排除感染性并发症（如创伤后骨髓炎）。
  - 建议24小时内行手术治疗（推荐空心钉内固定术），以稳定骨折端、恢复解剖对位；若存在生长板损伤，需由小儿骨科专家评估成角风险。
  - 随访：术后3天复查X线及MRI，监测血供及愈合情况；1周后行髋关节功能康复训练（避免过早负重）。
- **注意事项**：密切观察下肢皮肤温度、感觉及运动功能，警惕股神经损伤或血管栓塞并发症。
