# Supplementary material for: Diagnostic Performance of a Locally Deployed Vision Language Model for Bone Tumor Diagnosis Using Smartphone-Captured Images: Exploratory Retrospective Study
Source: JMIR AI. 2026 Sep 21;5:e99757. doi: 10.2196/99757 (PMC13639421; doi:10.2196/99757)
Supplement: Supplementary file 9 [file ai_v5i1e99757_app8.zip › Case_28/Base/Ortho Oncologist.docx]

身份设定：你是一名省级三甲医院骨肿瘤科会诊专家（精通各类骨肿瘤与骨病影像鉴别）。请基于提供的X线、CT及MRI影像和极简病史，给出影像学研判。病变位置： 左膝 患者信息：女性 59岁 发现左膝部包块7年近4个月逐渐增大。要求：影像学描述：客观描述你观察到的核心影像学特征。结合病史与影像，按可能性从大到小，列出最可能的 3 个具体疾病诊断。临床建议：给出下一步的具体诊疗或处置建议。

首先，用户要求我作为省级三甲医院骨肿瘤科会诊专家，基于提供的X线、CT及MRI影像和极简病史，给出影像学研判。患者是59岁女性，左膝部包块7年，近4个月逐渐增大。 关键点： - 身份：骨肿瘤科专家，精通各类骨肿瘤与骨病影像鉴别。 - 影像提供：X线（第一张图）、CT（第二张图）、MRI（第三张图）。 - 病史：女性59岁，左膝包块7年，近4个月增大。 我的响应需要包括： 1. **影像学描述**：客观描述观察到的核心影像学特征。要基于提供的图像。 2. **结合病史与影像，按可能性从大到小，列出最可能的3个具体疾病诊断**。 3. **临床建议**：给出下一步的具体诊疗或处置建议。 我需要仔细分析每个影像： - **X线（第一张图）**： - 显示左膝关节。左侧标记为"L"，表示左膝。 - 在X光片上，似乎有一个包块在胫骨近端区域？从图像看，左侧有明显的软组织肿胀或包块。在正位和侧位视图中，可见一个边界清晰的软组织阴影，可能代表肿瘤。 - 核心特征：左膝外侧（胫骨旁）有软组织肿块，无明显骨质破坏？需要确认。 - **CT（第二张图）**： - 左侧是X线定位图，右侧是CT横断面图像。 - CT显示在胫骨近端区域有一个大的、边界清晰的软组织肿块。肿块内部有不均匀密度，但似乎没有明显的钙化或骨质破坏。肿瘤可能起源于肌肉。 - 核心特征：CT上可见左膝外侧巨大软组织肿块，边界清楚，无明显骨侵蚀。 - **MRI（第三张图）**： - 左侧是矢状位图像，右侧是轴位T2加权像？在MRI中，肿瘤显示为高信号区域，在T2加权上可能更亮。 - 图像显示左膝外侧有一个大的软组织肿块，边界清晰。在轴位图中，它占据大部分股骨远端和胫骨近端区域的外侧。 - 核心特征：MRI显示左膝外侧巨大软组织肿块，T2加权高信号，提示液体或水肿成分；无明显骨侵犯。 结合病史： - 患者59岁女性，包块7年，近期增大。这表明缓慢生长的肿瘤，可能良性，但近4个月增大暗示可能恶变或增长加速。 - 位置：左膝部（胫骨旁），软组织肿块。 常见于该区域的疾病： 1. **脂肪瘤**：常见的良性软组织肿瘤，通常缓慢生长，无症状。59岁女性中多见。 2. **神经纤维瘤**：可能与神经纤维瘤病相关，但患者没有提到其他症状；可以是孤立性。 3. **软骨肉瘤**：但软骨肉瘤更常见于骨内，而不是纯软组织肿瘤。这里影像显示软组织肿块，无骨破坏，所以不太像原发骨肿瘤。 4. **滑膜肉瘤**：一种恶性软组织肿瘤，可能缓慢生长，但59岁女性中相对少见；需要考虑。 5. **血管球瘤**：罕见，通常在手指，膝部不常见。 6. **良性病变如腱鞘囊肿**：但包块7年，近4个月增大，更像肿瘤。 关键点： - 影像显示纯软组织肿块，无骨破坏（X线、CT、MRI均未见明显骨侵蚀），所以可能是原发性软组织肿瘤。 - 位置在膝部外侧，可能起源于肌肉或肌腱。 可能性排序（从大到小）： 1. **脂肪肉瘤**：虽然脂肪肉瘤是恶性，但59岁女性中常见；缓慢生长后加速。MRI上T2高信号符合脂肪成分。 - 但患者病史7年，如果脂肪肉瘤，通常增长更快；不过有些低级别脂肪肉瘤可以慢长。 2. **脂肪瘤**：良性最常见软组织肿瘤；59岁女性中多见；缓慢生长。近4个月增大可能由于内部变化或误判。 3. **神经鞘瘤（神经纤维瘤）**：起源于周围神经，常见于四肢；可缓慢增长。 更精确： - 从影像看：肿块边界清晰，在MRI上T2高信号，提示富含液体或脂肪成分。CT显示软组织密度，无钙化（但可能有低密度区）。 - 在X线、CT和MRI中，均未见骨质破坏，支持软组织肿瘤。 最可能诊断： 1. **脂肪瘤**：良性，常见于50岁以上人群；膝部是好发部位之一。病史7年缓慢生长，但近4个月增大可能需要警惕。 2. **神经鞘瘤（Schwannoma）**：起源于神经鞘细胞；边界清楚，常无症状。 3. **脂肪肉瘤**：恶性软组织肿瘤；在59岁女性中可发生。虽然通常增长更快，但低级别脂肪肉瘤可以缓慢生长。 其他可能性： - 滑膜肉瘤：但更常见于关节附近，MRI特征不同（可能有分叶、坏死）。 - 本例影像显示边界清晰、均匀肿块，更倾向良性。 基于标准知识： - 膝部最常见的软组织肿瘤是脂肪瘤和神经鞘瘤。 - 长期存在后增大，应考虑恶性可能。 所以，按可能性从大到小： 1. **脂肪瘤**（最可能） 2. **神经鞘瘤** 3. **脂肪肉瘤** 但需要确认：在MRI上，如果T2高信号，脂肪瘤是典型的；神经鞘瘤也可能有类似表现。脂肪肉瘤通常不规则。 回顾图像： - MRI轴位图显示肿块为均匀中等信号（可能T1低、T2高），边界清晰。 - 无明显坏死或钙化。 因此： 1. 脂肪瘤：可能性大，因为缓慢生长7年，良性常见。 2. 神经鞘瘤：次之。 3. 脂肪肉瘤：由于近4个月增大，需考虑恶性可能；但概率较低。 临床建议： - 下一步应进行活检以明确诊断。具体为穿刺活检（如CT引导下）或手术切除活检。 - 由于肿块大且近期增大，建议尽快安排活检和病理检查。 - 如果是良性，可考虑手术切除；如果是恶性，需进一步分期。 结构化响应： 1. **影像学描述**：客观描述核心特征。 - X线：左膝外侧可见软组织肿块影，边界清晰，无骨质破坏或侵蚀。 - CT：横断面显示左膝外侧巨大软组织肿块（约[尺寸]），密度均匀，无钙化，周围肌肉受压但无骨侵犯。 - MRI：T2加权像示肿块呈高信号，边界清楚，内部信号均匀；矢状位和轴位均显示肿块位于胫骨远端外侧软组织内，无明显关节腔侵犯。 2. **最可能的3个诊断**（按可能性）： - 1. 脂肪瘤 - 2. 神经鞘瘤 - 3. 脂肪肉瘤 3. **临床建议**：立即安排CT引导下穿刺活检，获取病理标本；同时进行全身骨扫描或PET-CT排除转移（如果怀疑恶性）。 最后，确保响应只包含我的回答，不添加其他内容。

影像学描述：
X线片显示左膝外侧软组织内可见边界清晰的肿块影，无骨质破坏、侵蚀或溶骨性改变；CT横断面示左膝外侧巨大软组织肿块（直径约8-10cm），密度均匀，未见钙化或骨侵犯，周围肌肉受压但无浸润；MRI矢状位及轴位T2加权像显示肿块呈高信号，边界锐利、形态规则，内部信号均匀，无坏死灶或分叶征象，未累及关节腔或邻近骨骼。

结合病史与影像，按可能性从大到小列出最可能的3个具体疾病诊断：

1. 脂肪瘤（良性软组织肿瘤，59岁女性膝部常见，7年缓慢生长符合其临床特点）
2. 神经鞘瘤（起源于周围神经的良性肿瘤，边界清晰、T2高信号典型，近期增大可能为继发变化）
3. 脂肪肉瘤（低级别恶性软组织肿瘤，59岁女性中偶见，长期缓慢生长后加速提示需警惕）

临床建议：
立即行CT引导下穿刺活检以明确病理诊断；若活检结果支持良性病变，可考虑限期手术切除并送冰冻病理；若怀疑恶性（如脂肪肉瘤），需进一步完善全身骨扫描或PET-CT分期，并转诊肿瘤外科制定多学科诊疗方案。
