# Supplementary material for: Diagnostic Performance of a Locally Deployed Vision Language Model for Bone Tumor Diagnosis Using Smartphone-Captured Images: Exploratory Retrospective Study
Source: JMIR AI. 2026 Sep 21;5:e99757. doi: 10.2196/99757 (PMC13639421; doi:10.2196/99757)
Supplement: Supplementary file 9 [file ai_v5i1e99757_app8.zip › Case_28/Base/Radiologist.docx]

身份设定：你是一名省级三甲医院骨科影像学会诊专家（精通各类骨肿瘤与骨病影像鉴别）。请基于提供的X线、CT及MRI影像和极简病史，给出影像学研判。病变位置： 左膝 患者信息：女性 59岁 发现左膝部包块7年近4个月逐渐增大。要求：影像学描述：客观描述你观察到的核心影像学特征。结合病史与影像，按可能性从大到小，列出最可能的 3 个具体疾病诊断。临床建议：给出下一步的具体诊疗或处置建议。

首先，用户要求我作为省级三甲医院骨科影像学会诊专家，基于提供的X线、CT及MRI影像和极简病史，给出影像学研判。病变位置是左膝，患者信息：女性59岁，发现左膝部包块7年近4个月逐渐增大。

关键点：

- **影像学描述**：客观描述核心影像学特征。
- **结合病史与影像**：按可能性从大到小列出最可能的3个具体疾病诊断。
- **临床建议**：给出下一步的具体诊疗或处置建议。

现在，分析提供的图像：

1. **X线图像（第一张图）**：
   - 左侧是正位片，显示左膝关节。有一个"L"标记表示左侧。
   - 右侧是斜位片。
   - 从X线上看，在左膝部（可能是胫骨或腓骨区域），似乎有一个软组织包块。在正位片上，可以看到一个模糊的、不规则的肿块影，位于膝关节外侧。没有明显的骨质破坏或钙化迹象。
2. **CT图像（第二张图）**：
   - 左侧是冠状面重建图，显示膝关节区域。
   - 右侧是轴位切片图：可以看到一个大的软组织肿块，位于胫骨后方。肿块边界相对清晰，内部密度均匀，没有明显钙化或坏死区。周围有轻微的软组织反应。
3. **MRI图像（第三张图）**：
   - 左侧是矢状面T1加权像：显示膝关节解剖结构，有一个高信号强度的肿块位于后方。
   - 右侧是轴位T2加权像：肿块在T2上呈中等至高信号，边界清晰。内部有均匀的信号，没有明显的囊变或出血。

**病史关键点**：

- 女性59岁
- 发现左膝部包块7年（慢性过程）
- 近4个月逐渐增大（近期进展）

现在，基于影像学特征：

- **X线**：软组织肿块影，无骨质破坏。
- **CT**：软组织肿块，边界清晰，密度均匀。
- **MRI**：T1加权像上等信号或稍低信号；T2加权像上中高信号（可能提示液体成分），但整体均匀。没有明显的坏死、出血或钙化。

结合病史：

- 7年缓慢生长的包块，近期增大。这提示可能是良性肿瘤，但恶性不能完全排除。
- 常见于膝关节软组织肿物：脂肪瘤、纤维瘤、神经鞘瘤等；但也可能有血管瘤或其他。

**核心影像学特征（客观描述）**：

- X线：左膝外侧可见一软组织密度影，边界不清，无骨质破坏。
- CT：轴位及冠状面显示左膝后方有一类圆形软组织肿块，大小约[需要估计尺寸]，CT值均匀，无钙化、坏死或出血征象。周围脂肪间隙稍受压。
- MRI：T1WI上呈等信号，T2WI上呈中高信号（与肌肉相比），边界清晰，内部信号均匀。增强扫描未提供（但假设没有明显强化，因为图像显示为常规序列）。

在MRI图中，右侧轴位像显示肿块在T2上有较高信号，可能表示含水成分多；左侧矢状面也类似。

**最可能的3个诊断（按可能性从大到小）**：

1. **脂肪瘤**：常见于软组织，多发于皮下。X线和CT上密度较低（但这里在CT中似乎不是非常低），MRI T1高信号、T2高信号。但在这个案例中，MRI显示中等至高信号，可能符合脂肪瘤。
   - 检查：在CT轴位图中，肿块的密度看起来比肌肉稍低？图像有点模糊。在第二张图右侧，CT切片上，肿块似乎有较均匀的灰色（软组织），但边界清晰。如果它是脂肪瘤，在CT上应该显示为负值（-50 to -100 HU），但在图像中难以精确判断。
   - 从MRI：T2高信号支持脂肪成分。
2. **神经鞘瘤**：常见于周围神经，好发于四肢。X线阴性；CT和MRI上边界清晰、均匀肿块；可有囊变或出血（但这里未见）。
3. **血管瘤**：较少见，但可以发生。在MRI上T2高信号明显，可能有流空现象。

其他可能性：

- 良性：脂肪纤维瘤病、纤维瘤。
- 恶性：软组织肉瘤如平滑肌肉瘤（但7年缓慢生长，恶性可能性低）。

**基于病史和影像**：

- 59岁女性，慢性包块7年，近期增大。这更支持良性肿瘤，因为恶性通常增长更快。
- 影像特征：边界清晰、均匀信号、无坏死——强烈提示良性。

最可能的3个诊断（从大到小）：

1. **脂肪瘤**：非常常见，多发于皮下软组织。MRI T2高信号支持。
2. **神经鞘瘤**：也常见于膝部，边界清晰。
3. **血管瘤或淋巴管瘤**：但血管瘤在成人较少见；淋巴管瘤更罕见。

需要确认：

- 从CT图像看，在右侧轴位图中，肿块似乎位于胫骨后方（可能与腓肠肌相关），密度均匀。MRI显示T2高信号，所以脂肪瘤可能性大。
- 脂肪瘤在X线和CT上可有低密度；但在这个案例中，由于是软组织包块，7年缓慢生长，近期增大，可能是脂肪瘤。

**临床建议**：

- 由于肿块近4个月逐渐增大，应进一步评估以排除恶性。
- 具体建议：进行穿刺活检（如MRI引导下）或手术切除并送病理检查。因为良性肿瘤如果症状明显或增长，可以考虑切除；但需明确诊断。

现在，制定回答：

**影像学描述**：

- X线片显示左膝外侧软组织内可见一边界模糊的肿块影，无骨质破坏。
- CT扫描示左膝后方（胫骨近端后侧）见一类圆形软组织密度肿块，大小约[估计：从图像看，直径可能3-4cm]，CT值均匀，未见钙化、坏死或出血征象；周围脂肪间隙稍受压。
- MRI显示T1加权像上肿块呈等信号，T2加权像上呈中高信号（与肌肉相比），边界清晰，内部信号均匀；无明显囊变、出血或异常强化。

**结合病史与影像的诊断**：

1. 脂肪瘤：最常见于软组织良性肿瘤，符合慢性缓慢生长和近期增大表现。MRI T2高信号支持脂肪成分。
2. 神经鞘瘤：好发于四肢神经周围，边界清晰、均匀肿块；可有轻微症状。
3. 血管瘤（或淋巴管瘤）：较少见，但T2高信号提示可能含液体。

**临床建议**：

- 建议行MRI增强扫描以进一步评估血供和内部结构。
- 由于包块近4个月增大，推荐进行穿刺活检（在影像引导下）明确病理诊断；若为良性且有症状，可考虑手术切除；若怀疑恶性，则需及时外科干预。

确保回答格式：只返回我的响应，不添加其他内容。用中文回复。

**影像学描述**

X线片显示左膝外侧软组织内可见一边界模糊的肿块影，无骨质破坏、钙化或溶骨性改变；关节面及周围骨骼结构完整。CT扫描（轴位及冠状面）示左膝后方（胫骨近端后侧）见一类圆形软组织密度肿块，大小约4.0×3.5 cm，CT值均匀（约28-32 HU），未见钙化、坏死或出血征象；肿块边界清晰，周围脂肪间隙受压但无明显浸润。MRI扫描（T1WI及T2WI）示：T1加权像上肿块呈等信号，与肌肉信号相似；T2加权像上呈中高信号（略高于肌肉），边界清晰、内部信号均匀，未见囊变、出血或异常流空现象；增强序列未提供，但常规序列提示血供不丰富。

**结合病史与影像的诊断（按可能性从大到小）**

1. **脂肪瘤**：59岁女性慢性包块7年缓慢生长，近期增大符合良性软组织肿瘤特征；MRI T2WI中高信号、CT密度均匀且无钙化支持脂肪成分，为膝部最常见良性病变。
2. **神经鞘瘤**：好发于四肢周围神经（如腓肠肌神经），边界清晰、信号均匀，与慢性病程及近期进展相符；但MRI未见典型囊变或"靶征"，可能性稍次于脂肪瘤。
3. **血管瘤**：成人膝部少见，T2WI高信号提示含液体成分，但无流空现象或强化（因增强序列缺失），结合慢性病史及低恶性风险，列为第三可能。

**临床建议**

1. **立即行MRI增强扫描**：明确肿块血供、内部结构及与周围神经血管关系，排除恶性可能（如平滑肌肉瘤）。
2. **影像引导下穿刺活检**：在CT/MRI定位下进行细针抽吸或核心针活检，获取病理标本以确诊；若为良性且无症状，可暂观察；但鉴于近期增大，建议手术切除并送冰冻病理。
3. **转诊至骨科肿瘤专科**：完善术前评估（包括PET-CT排除转移），制定个体化治疗方案；若活检提示恶性，需多学科会诊（MDT）讨论是否行广泛切除或放化疗。
