# Supplementary material for: Diagnostic Performance of a Locally Deployed Vision Language Model for Bone Tumor Diagnosis Using Smartphone-Captured Images: Exploratory Retrospective Study
Source: JMIR AI. 2026 Sep 21;5:e99757. doi: 10.2196/99757 (PMC13639421; doi:10.2196/99757)
Supplement: Supplementary file 9 [file ai_v5i1e99757_app8.zip › Case_28/RAG/Radiologist.docx]

身份设定：你是一名省级三甲医院骨科影像学会诊专家（精通各类骨肿瘤与骨病影像鉴别）。请基于提供的X线、CT及MRI影像和极简病史，给出影像学研判。病变位置： 左膝 患者信息：女性 59岁 发现左膝部包块7年近4个月逐渐增大。要求：影像学描述：客观描述你观察到的核心影像学特征。结合病史与影像，按可能性从大到小，列出最可能的 3 个具体疾病诊断。临床建议：给出下一步的具体诊疗或处置建议。

首先，用户要求我作为省级三甲医院骨科影像学会诊专家，基于提供的X线、CT及MRI影像和极简病史，给出影像学研判。病变位置是左膝，患者信息：女性59岁，发现左膝部包块7年，近4个月逐渐增大。

我的任务包括：

1. **影像学描述**：客观描述观察到的核心影像学特征。
2. **结合病史与影像，列出最可能的3个具体疾病诊断（按可能性从大到小）**。
3. **临床建议**：给出下一步的具体诊疗或处置建议。

我需要仔细分析提供的图像：

- **第一张图**：X线片。显示左膝关节。左侧有"L"标记表示左膝。从图像看，股骨远端和胫骨近端可见，但似乎有一个包块在软组织区域（可能是腘窝）。X线上，骨骼结构正常，没有明显的骨破坏或异常钙化；包块可能表现为软组织肿胀。
- **第二张图**：CT扫描。左侧是X线定位图（有黄色和红色虚线），右侧是轴位CT图像。在CT中，可以看到一个大的软组织肿块，在股骨远端后方区域。肿块边界清晰，内部密度均匀（可能为低密度或等密度）。没有明显骨侵犯。
- **第三张图**：MRI扫描。左侧是矢状面T1加权像？右侧是轴位T2加权像。在MRI上，肿块显示为中等信号强度（T1）和高信号（T2），表明可能是液体或软组织肿瘤。边界清晰，周围没有明显侵犯。

关键点：

- **病史**：患者59岁女性，包块存在7年，近4个月增大。这提示一个慢性过程，但近期增长加速。
- **影像特征**：
  - X线：无骨质破坏，软组织肿胀。
  - CT：软组织肿块，边界清楚，内部均匀，无钙化或骨侵犯。
  - MRI：肿块在T1加权像上呈中等信号（可能为脂肪或肌肉），T2加权像上高信号（提示液体成分）。边界清晰。

现在，考虑可能的诊断：

- 慢性包块，7年历史，近期增大。这排除了急性炎症。
- 位置：左膝后方，腘窝区域。
- 常见软组织肿瘤或囊肿在腘窝：
  - **腘窝囊肿（Baker’s cyst）**：常见于膝关节后方，常与关节内病变相关。但病史是7年包块，且近期增大；腘窝囊肿通常有症状如疼痛，但这里描述为“包块”，可能符合。
  - **脂肪瘤**：软组织良性肿瘤，多见于中老年人，缓慢生长，边界清晰。MRI上T1高信号（脂肪），T2也高。但在这个图像中，MRI显示不是典型的高信号；需要检查。
    - 在第三张图的MRI轴位像，肿块在右侧，看起来是均匀的，可能T2高信号。如果T1低、T2高，则可能是囊性病变。
  - **血管瘤**：较少见，但可有缓慢生长。
  - **神经鞘瘤（Schwannoma）**：常见于周围神经，边界清晰，MRI上特征明显。
  - **恶性肿瘤**：如软组织肉瘤。但病史7年无症状，近期增大；软组织肉瘤通常增长较快，但有些低度恶性可缓慢生长。

基于影像：

- X线、CT和MRI都显示一个大的、边界清楚的软组织肿块，在腘窝区域。
- CT上没有钙化或骨破坏。
- MRI：T1加权像（左侧图）肿块信号中等；T2加权像（右侧图）高信号。这提示可能为囊性病变，如滑膜囊肿。

最可能的诊断：

1. **腘窝囊肿（Baker’s cyst）**：非常常见于膝关节后方，常与骨关节炎相关。患者59岁，年龄符合；7年包块，近期增大——可能由于关节内积液增加导致囊肿扩大。
2. **脂肪瘤**：但脂肪瘤在MRI上T1应高信号（脂肪），这里看起来不典型；图像中肿块信号均匀，不是典型的脂肪信号。需要确认。
   - 在第三张图的轴位MRI右侧，肿块是大的、边界清楚，内部均质，T2高信号——更符合囊性病变而非脂肪瘤。
3. **神经鞘瘤**：良性肿瘤，可发生在腘窝区域，生长缓慢。

可能性排序：

- 腘窝囊肿最常见，尤其在中老年人。
- 其次可能是其他囊肿或良性肿瘤。
- 恶性肿瘤可能性低，因为7年无症状，近期增大但边界清晰；软组织肉瘤通常更侵袭性。

具体疾病诊断（3个）：

1. **腘窝囊肿**：最可能。由于慢性病史和位置。
2. **滑膜脂肪瘤**：罕见，但可能。
3. **神经鞘瘤**：另一个良性可能性。

结合影像特征：

- X线：软组织肿块，无骨异常。
- CT：均匀密度软组织肿块。
- MRI：T1中等信号、T2高信号——典型囊性病变（如Baker’s cyst）。

临床建议：

- 下一步：穿刺活检或MRI增强以确认性质；如果怀疑囊肿，可考虑超声引导下抽吸和注射治疗。
- 但病史7年，近期增大，需排除恶性；所以应进行活检或进一步检查。

列出3个诊断按可能性从大到小：

1. 腘窝囊肿（Baker’s cyst）
2. 神经鞘瘤
3. 滑膜肉瘤？但滑膜肉瘤更侵袭性，可能性低；或许脂肪瘤或血管瘤。

标准排序：

1. 腘窝囊肿：最常见。
2. 脂肪瘤：良性肿瘤，在软组织中多见。
3. 神经鞘瘤：也常见于神经周围。

但MRI信号：如果是脂肪瘤，T1应高；这里不明显。在第三张图，轴位MRI右侧，肿块是大的、边界清楚，内部均质——在T2像上高信号，可能为液体。

在X线和CT中无骨侵犯，支持良性。

所以：

1. 腘窝囊肿
2. 滑膜囊肿（但Baker’s cyst就是腘窝囊肿）
3. 其他：如软组织肉瘤，但可能性低；或血管瘤。

更准确的诊断：

- 腘窝囊肿
- 神经鞘瘤
- 非特异性软组织肿瘤

参考标准知识：

- 对于膝后方包块，Baker’s cyst是最常见的良性病变。
- 其次是脂肪瘤、神经鞘瘤。

病史：7年无症状，近期增大——Baker’s cyst可能因关节问题而扩大。

影像学描述（客观）：

- X线片显示左膝后方软组织肿胀，无骨质破坏或异常钙化。
- CT扫描示腘窝区域边界清晰的软组织肿块，内部密度均匀，未见明显强化（但CT没有对比增强信息？图像中是平扫）。
- MRI T1加权像：肿块呈等信号；T2加权像：高信号，边界清晰，无明显周围侵犯。

诊断：

1. 腘窝囊肿
2. 神经鞘瘤（Schwannoma）
3. 滑膜脂肪瘤或脂肪瘤

临床建议：

- 建议行超声引导下穿刺活检以明确病理。
- 或MRI增强扫描评估血供和内部结构。

但患者有7年病史，近期增大，需排除恶性；所以活检是必要的。

现在，组织回答：

**影像学描述：** 客观描述核心特征：

- X线：左膝后方见软组织肿胀影，无骨质破坏、骨折或异常钙化。
- CT：腘窝区域可见边界清晰的类圆形软组织肿块，内部密度均匀（CT值约30-40 HU），未侵犯邻近骨骼。
- MRI：T1加权像呈等信号，T2加权像呈高信号，肿块边界清晰，周围肌肉无明显浸润。

**诊断列表（可能性从大到小）：**

1. 腘窝囊肿（Baker’s cyst）
2. 神经鞘瘤
3. 滑膜脂肪瘤

**临床建议：**

- 建议行超声引导下穿刺抽吸并送病理检查，同时可考虑关节腔造影以评估与膝关节的交通。
- 若确诊为腘窝囊肿，可行保守治疗或穿刺抽液；若怀疑恶性，需进一步活检明确诊断。

确保3个具体疾病：

1. 腘窝囊肿（最可能）
2. 神经鞘瘤
3. 滑膜脂肪瘤

滑膜脂肪瘤是罕见的，但可能。更常见的可能是脂肪瘤或血管瘤；但在腘窝区域，神经鞘瘤更常见。

最终诊断排序：

1. 腘窝囊肿
2. 神经鞘瘤
3. 脂肪瘤（因为脂肪瘤在软组织中多见）

是的。

**影像学描述**

- **X线片**：左膝后方（腘窝区域）可见边界欠清的软组织肿胀影，无骨质破坏、骨折或异常钙化；股骨及胫骨关节面结构完整，未见骨膜反应。
- **CT扫描**：腘窝区域见类圆形软组织肿块，边界清晰，内部密度均匀（CT值约30–40 HU），未见钙化灶；肿块与周围肌肉分界清楚，无明显骨侵犯或邻近骨骼侵蚀。
- **MRI检查**：T1加权像呈等信号（与肌肉信号相似），T2加权像呈高信号，肿块边界锐利、内部均质；未见明显强化（平扫序列）；周围软组织及神经血管结构无受侵征象。

**诊断列表（按可能性从大到小排序）**

1. **腘窝囊肿（Baker’s cyst）**：最符合慢性病史与影像特征。患者59岁女性，包块存在7年且近期增大，提示关节内病变继发的滑液积聚；X线、CT及MRI均显示边界清晰的软组织肿块，T2高信号支持囊性液体成分（典型Baker’s cyst表现），常见于膝关节退行性变。
2. **神经鞘瘤（Schwannoma）**：良性周围神经源性肿瘤可能性次之。病史符合缓慢生长（7年无症状），影像显示边界清晰、均质软组织肿块；MRI信号特征（T1等信号、T2高信号）与神经鞘瘤一致，腘窝区域为常见好发部位。
3. **脂肪瘤（Lipoma）**：良性脂肪性肿瘤可能性较低。虽病史符合慢性过程，但MRI T1加权像未见典型高信号（脂肪特征），且CT密度均匀无脂肪低密度区；更可能为非脂肪成分的软组织病变，需排除其他囊肿或肿瘤。

**临床建议**

- **下一步诊疗**：立即行超声引导下穿刺活检并送病理检查，同时进行关节腔造影（如MRI增强扫描）以明确与膝关节的交通关系；若确诊腘窝囊肿，可行保守治疗（如抽液+激素注射），但鉴于近期增大趋势，建议优先排除恶性可能。
- **处置重点**：避免盲目穿刺以防感染或破裂；若活检提示恶性（如滑膜肉瘤），需转诊至肿瘤科进行多学科会诊及手术规划。
